# Supplementary material for: Wave patterns organize cellular protrusions and control cortical dynamics
Source: Mol Syst Biol. 2019 Mar 12;15(3):e8585. doi: 10.15252/msb.20188585 (PMC6413885; doi:10.15252/msb.20188585)
Supplement: Supplementary file 1 — Appendix [file MSB-15-e8585-s001.pdf]

Appendix

Wave patterns organize cellular protrusions and control cortical dynamics

Yuchuan Miao*, Sayak Bhattacharya*, Tatsat Banerjee, Bedri Abubaker-Sharif, Yu Long, Takanari Inoue, Pablo A. Iglesias, and Peter N. Devreotes

Table of Content

-Appendix Figures

Appendix Figure S1. Biosensors in cortical waves.

Appendix Figure S2. F-actin dependencies of biosensor activities.

Appendix Figure S3. Computational modeling of waves.

Appendix Figure S4. Effects on waves after STEN threshold is lowered by CID.

Appendix Figure S5. Effects of recruiting PKBA by CID.

Appendix Figure S6. Effects on waves after PKB activities were increased by CID.

Appendix Figure S7. Effects on waves after RacGEF1ΔN recruitment by CID.

-Appendix Tables

Appendix Table S1. Table of simulation parameters.

-Descriptions of Movies EV 1-15

Appendix Figure S1

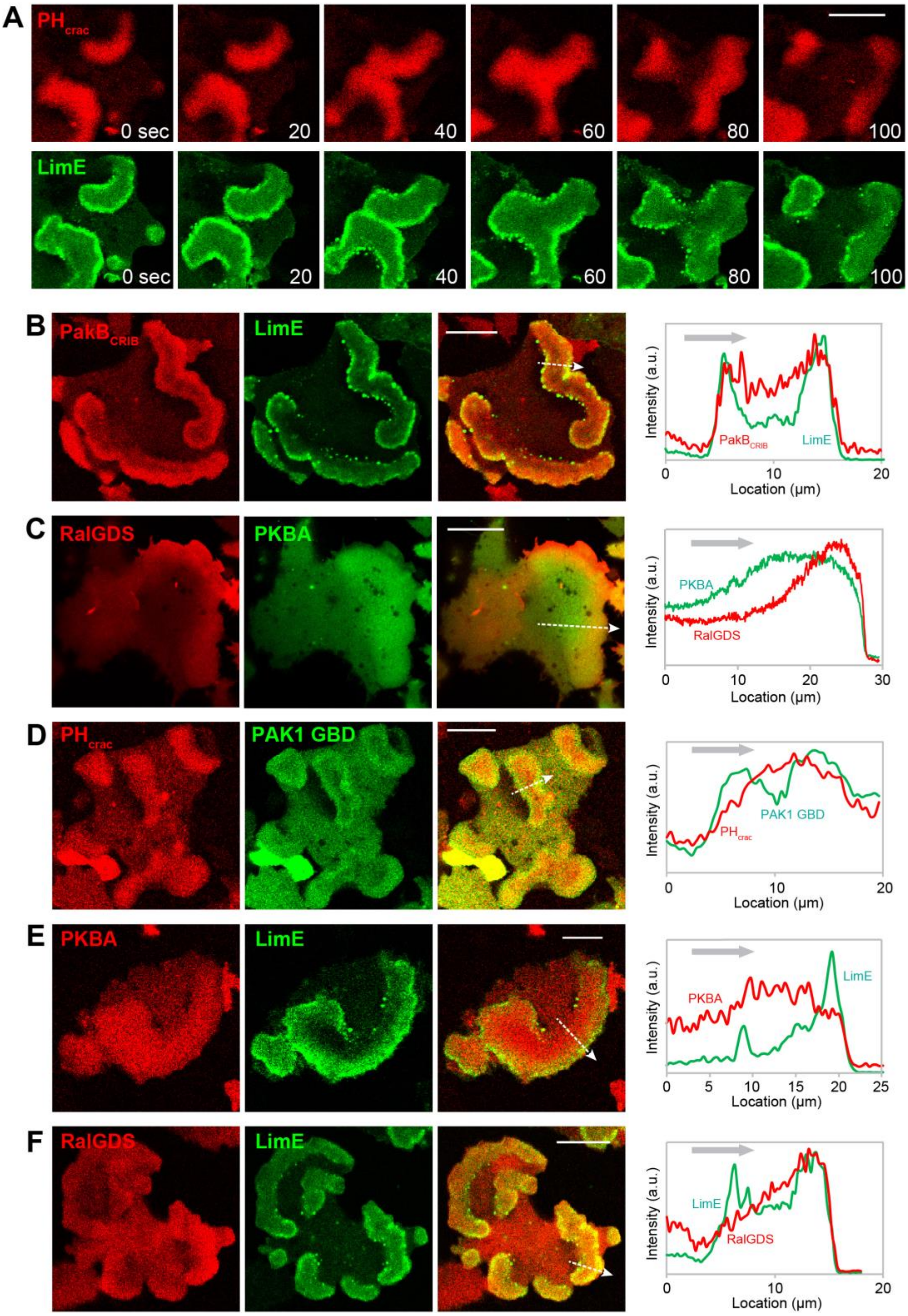

Appendix Figure S1. Biosensors in cortical waves.

(A) Time lapse confocal images of PH_{crac} (top) and LimE (bottom) at the basal surface of the same cell. **(B-F)** Confocal images of different biosensors (left) and intensity plots (right) across the white dotted arrows. Scale bars represent 20 µm.

Appendix Figure S2

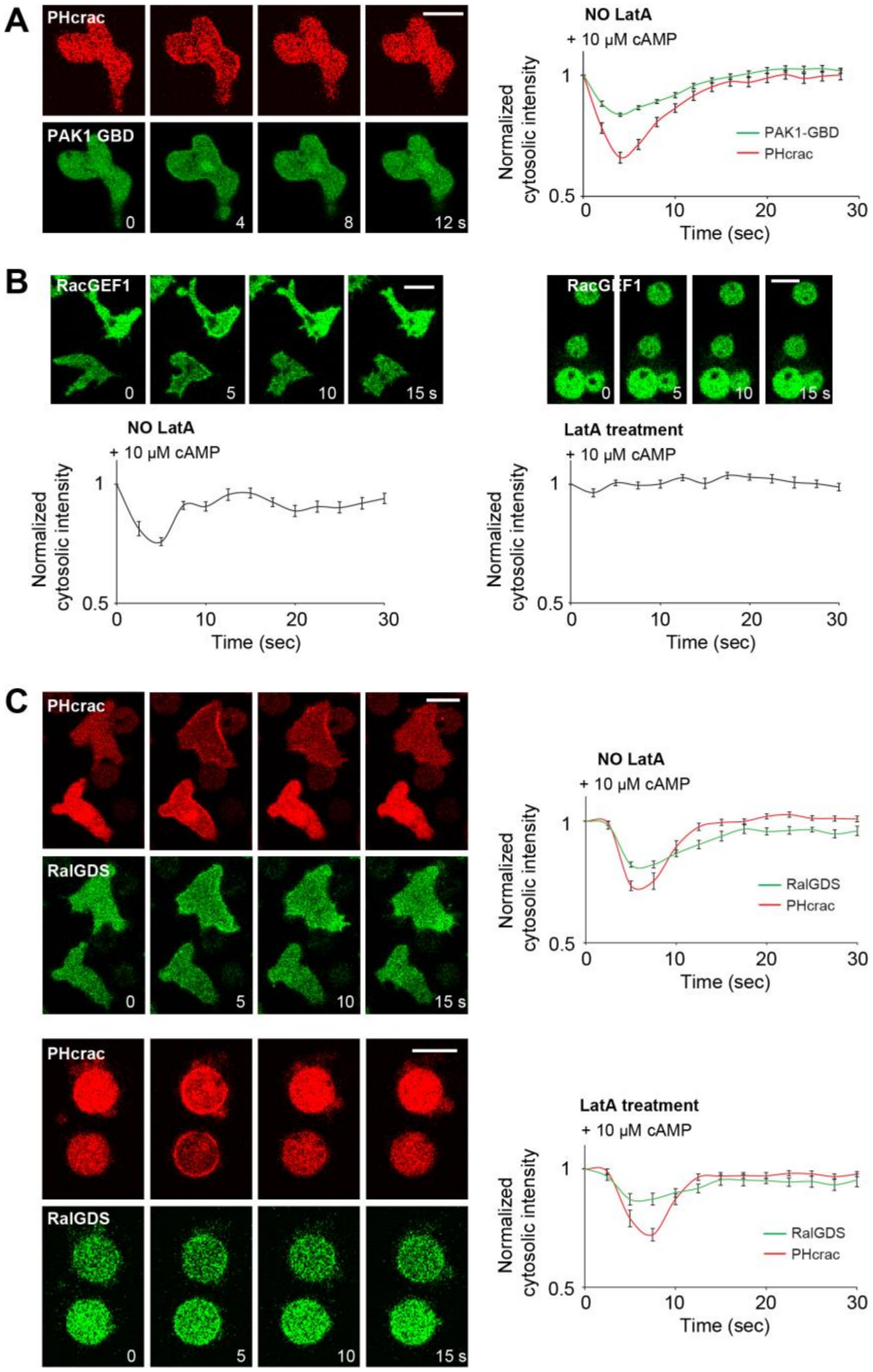

Appendix Figure S2. F-actin dependencies of biosensor activities.

(A) Time lapse confocal images of PH_{crac} and PAK1-GBD in the same cell (left) and temporal profiles of normalized cytosolic intensities (right) in response to 10 µM cAMP addition at time 0 (mean±s.e.m., n=18 cells). **(B)** Time lapse confocal images of PH_{crac} and RalGDS in the same cells (left) and temporal profiles of normalized cytosolic intensities (right) in response to 10 µM cAMP addition at time 0, with (bottom) or without (top) LatA treatment (mean±s.e.m., n=15 cells each). **(C)** Time lapse confocal images of RacGEF1 (Top) and temporal profiles of normalized cytosolic intensities (bottom) in response to 10 µM cAMP addition at time 0, with (right) or without (left) LatA treatment (mean±s.e.m., n=10 cells each). All scale bars represent 10 µm.

Appendix Figure S3

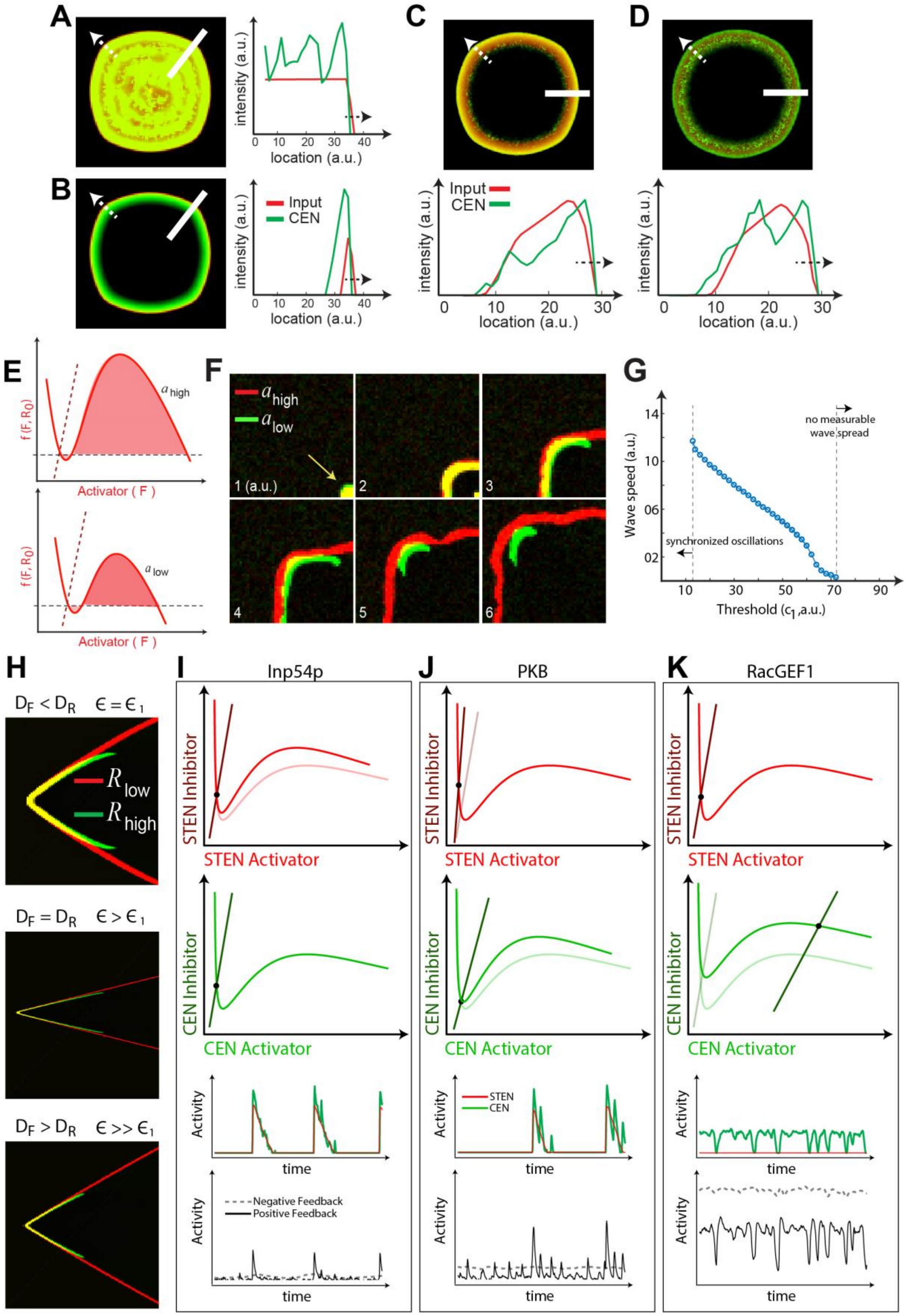

Appendix Figure S3. Computational modeling of waves.

(A) (left) A two-dimensional (2D) simulation showing the CEN output (green) when a step input is applied. The input is deterministically triggered at the center, causing CEN to spread outward into space. The input is overlaid in red, thus giving the simulation a yellow color during overlap. The line scan corresponding to the white line is shown to the right, with the dashed arrow indicating the direction of propagation of the wave. **(B)** Same simulation scheme as **A**, but with a pulse input. **(C, D)** (left) Two sample input profiles (red lines) generated by modifying the dynamics of the STEN profile. These were then applied to CEN to generate the wave snapshot shown on top. Line scans taken from the wave snapshots are shown below. **(E)** Plots showing how the nullclines are altered when the positive feedback parameter of the activator is changed. **(F)** A wave race corresponding to the two parameters in **E**. The higher positive feedback wave is in red, while the lower one is in green. **(G)** Wave speeds plotted for a range of the negative feedback parameter. **(H)** Three examples of threshold wave control, in order to illustrate the independence from particular diffusion coefficient values. The high threshold wave is shown in green while the low threshold is in red. The curving in of the high threshold wave from the low threshold trajectory indicates a slower wave speed. **(I-K)** Phase-plane plots for all three perturbations showing how the corresponding STEN and CEN nullclines are altered. The corresponding activity level along with the positive and negative CEN-to-STEN feedback levels are shown in the plots below.

Appendix Figure S4

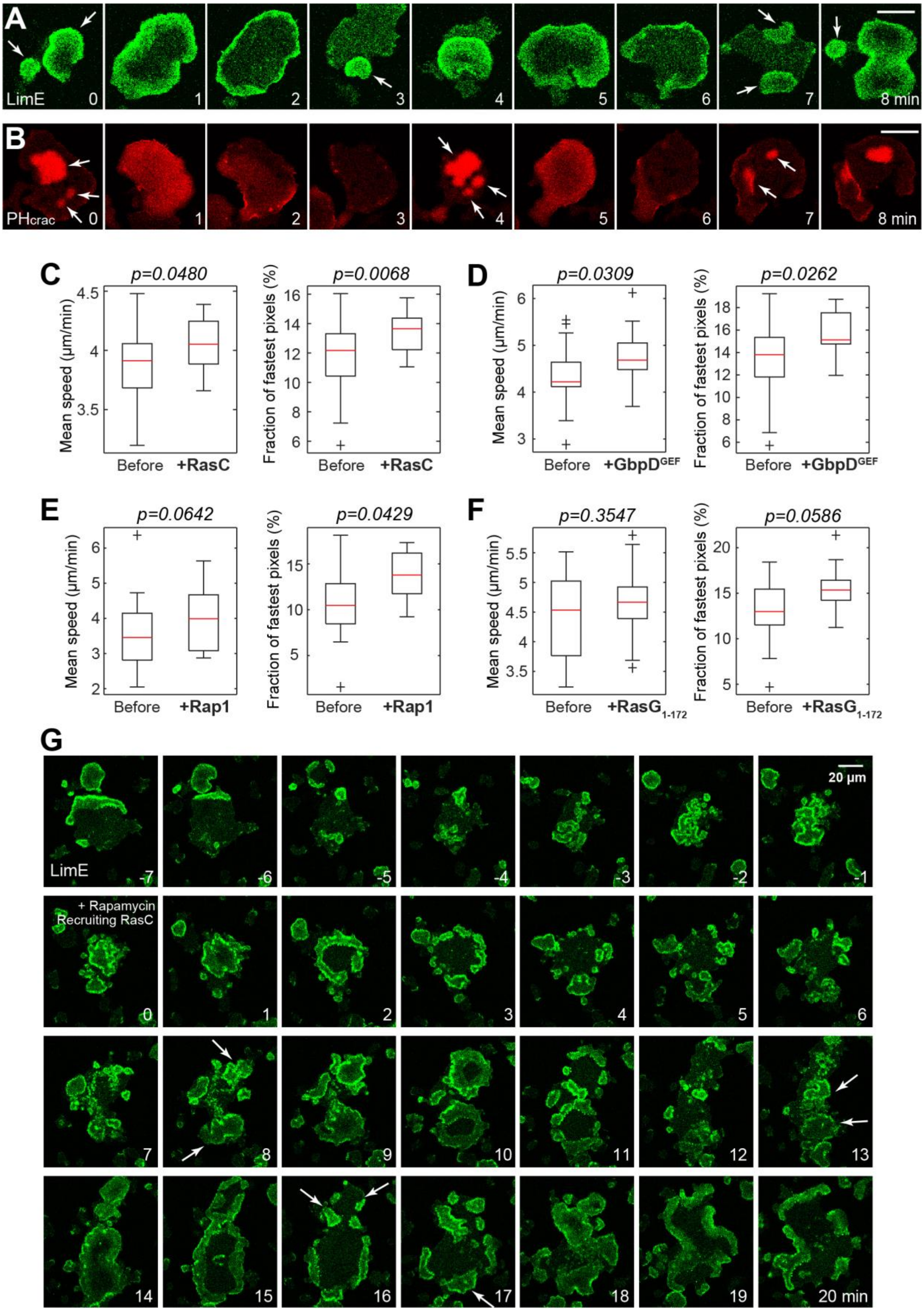

Appendix Figure S4. Effects on waves after STEN threshold is lowered by CID.

(A and **B)** Time lapse confocal images of LimE (**A**) and PH_{crac} (**B**) in a giant cell after steady Inp54p recruitment. White arrows point to nascent waves. **(C-F)** Box plots of mean wave speed (left) and fraction of fastest pixels (right) before and after recruiting: (**C**) $RasC^{Q62L}$ (2-185 amino acids, ΔCAAX) (n=22 cells), (**D**) the GEF domain (1-587 amino acids) of GbpD (n=19 cells), (**E**) $Rap1^{G12V}$ (1-182 amino acids, ΔCAAX) (n=15 cells), and (**F**) $RasG^{Q61L}$ (1-172 amino acids, more C-terminal residues other than the CAAX were deleted due to problems with expression and basal activities) (n=16 cells). Red bars indicate median. **(G)** Time lapse confocal images of LimE at the basal surface of a giant cell, which recruits $RasC^{Q62L, \Delta CAAX}$ to membrane induced by rapamycin at time 0. Arrows point to nascent waves. Scale bar represents 20 μm.

Appendix Figure S5

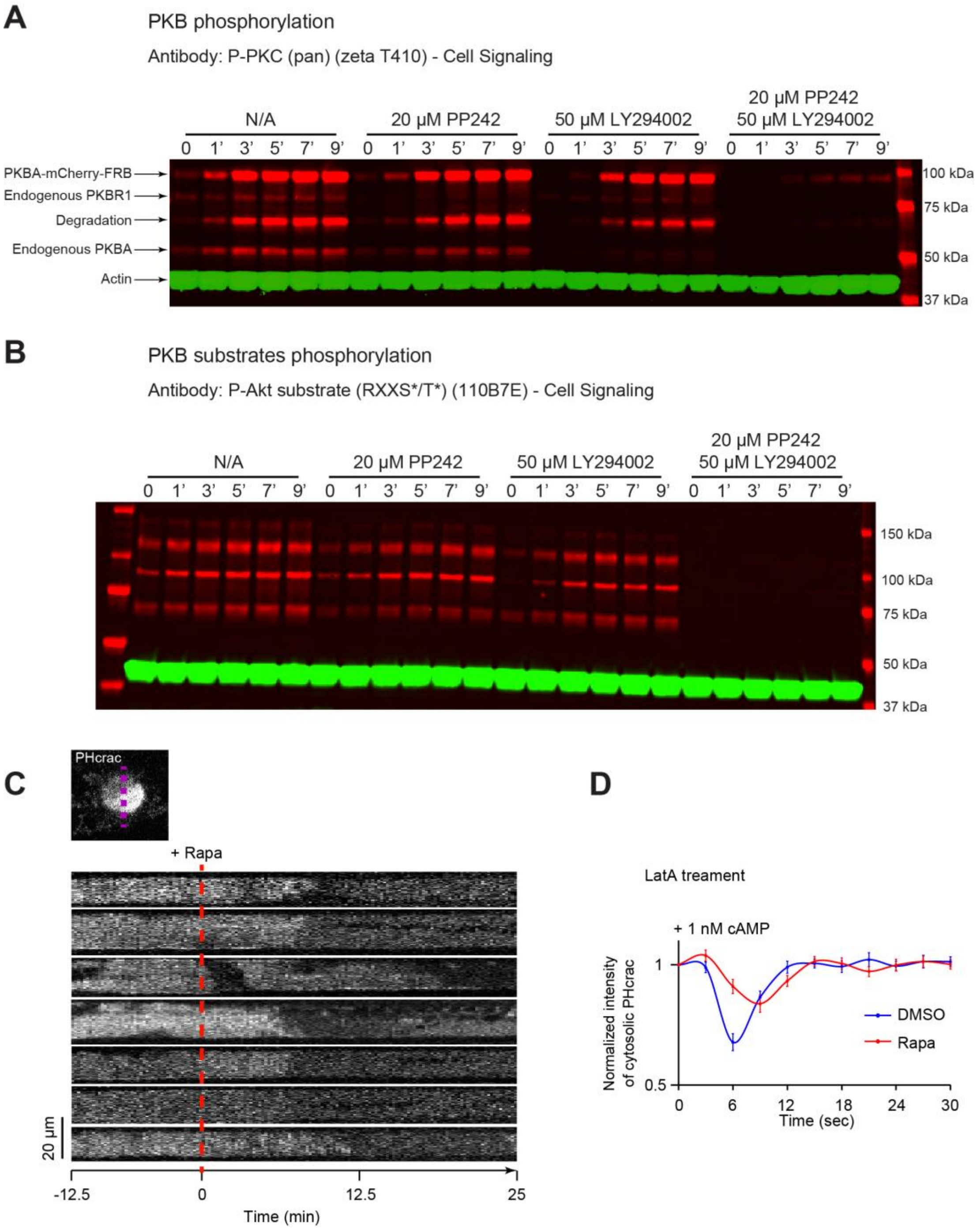

Appendix Figure S5. Effects of recruiting PKBA by CID.

(A and B) Western blots detecting PKB phosphorylation (**A**) and Akt substrates (**B**) following PKBA recruitment with or without treatment of PP242 (TorC2 inhibitor) and LY294002 (PI3K inhibitor). **(C)** Kymographs of PH_{crac} in 7 cells treated with LatA before and after PKBA recruitment induced by Rapamycin at time zero. The image on top is just an example showing the magenta dotted line from which the kymographs are made. **(D)** Temporal profiles of normalized cytosolic intensities of PH_{crac} with LatA treatment in response to 1 nM cAMP stimulation at time 0, with (red) or without (blue) PKBA recruitment (mean±s.e.m., n=30 cells each).

Appendix Figure S6

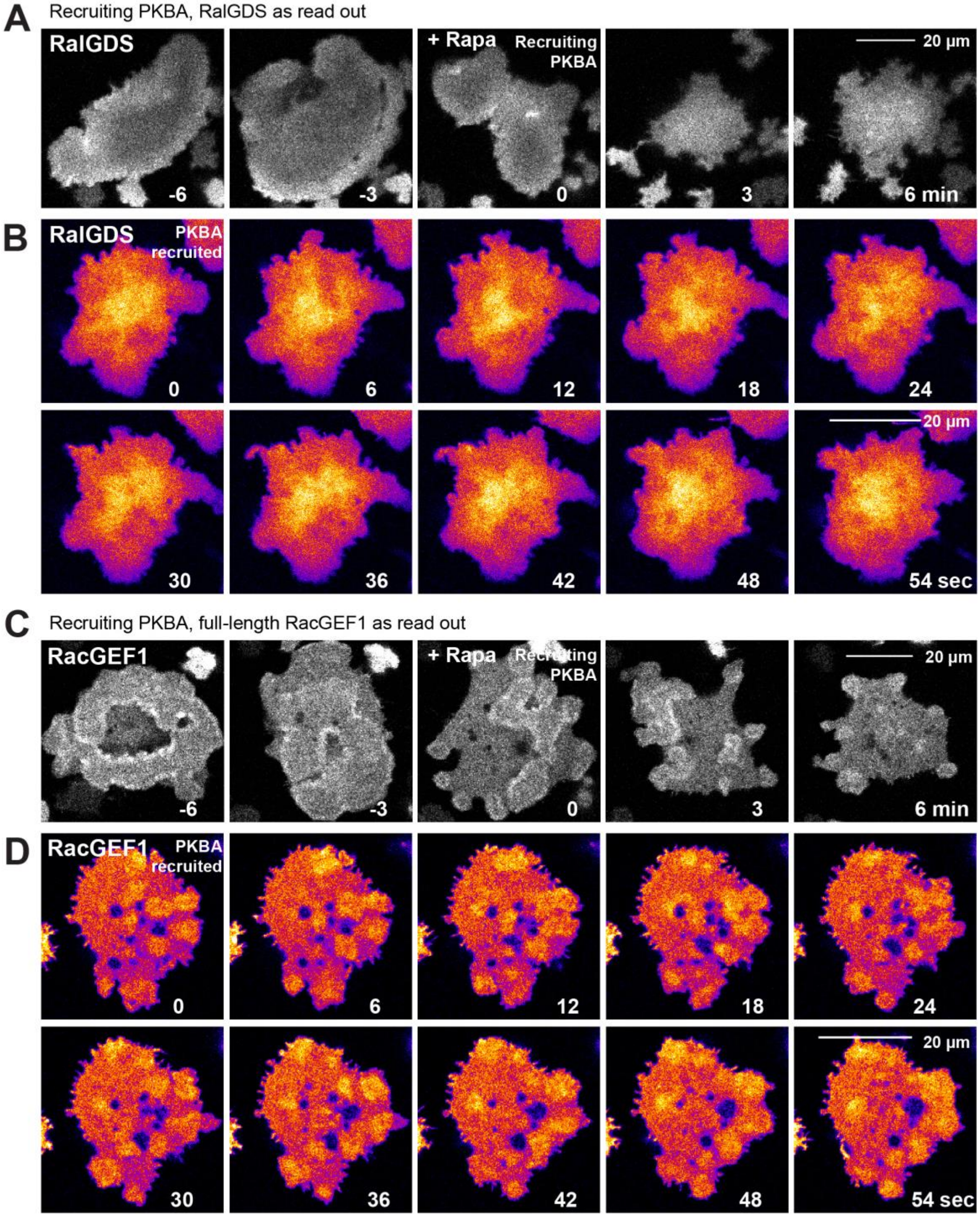

Appendix Figure S6. Effects on waves after PKB activities were increased by CID.

(A) Time lapse confocal images of RalGDS at the basal surface of a giant cell, which recruits PKBA to membrane induced by rapamycin at time 0. **(B)** Time lapse confocal images of RalGDS after PKBA recruitment as shown with pseudo colors. **(C)** Time lapse confocal images of RacGEF1 at the basal surface of a giant cell, which recruits PKBA to membrane induced by rapamycin at time 0. **(D)** Time lapse confocal images of RacGEF1 after PKBA recruitment as shown with pseudo colors. Scale bars represent 20 μm.

Appendix Figure S7

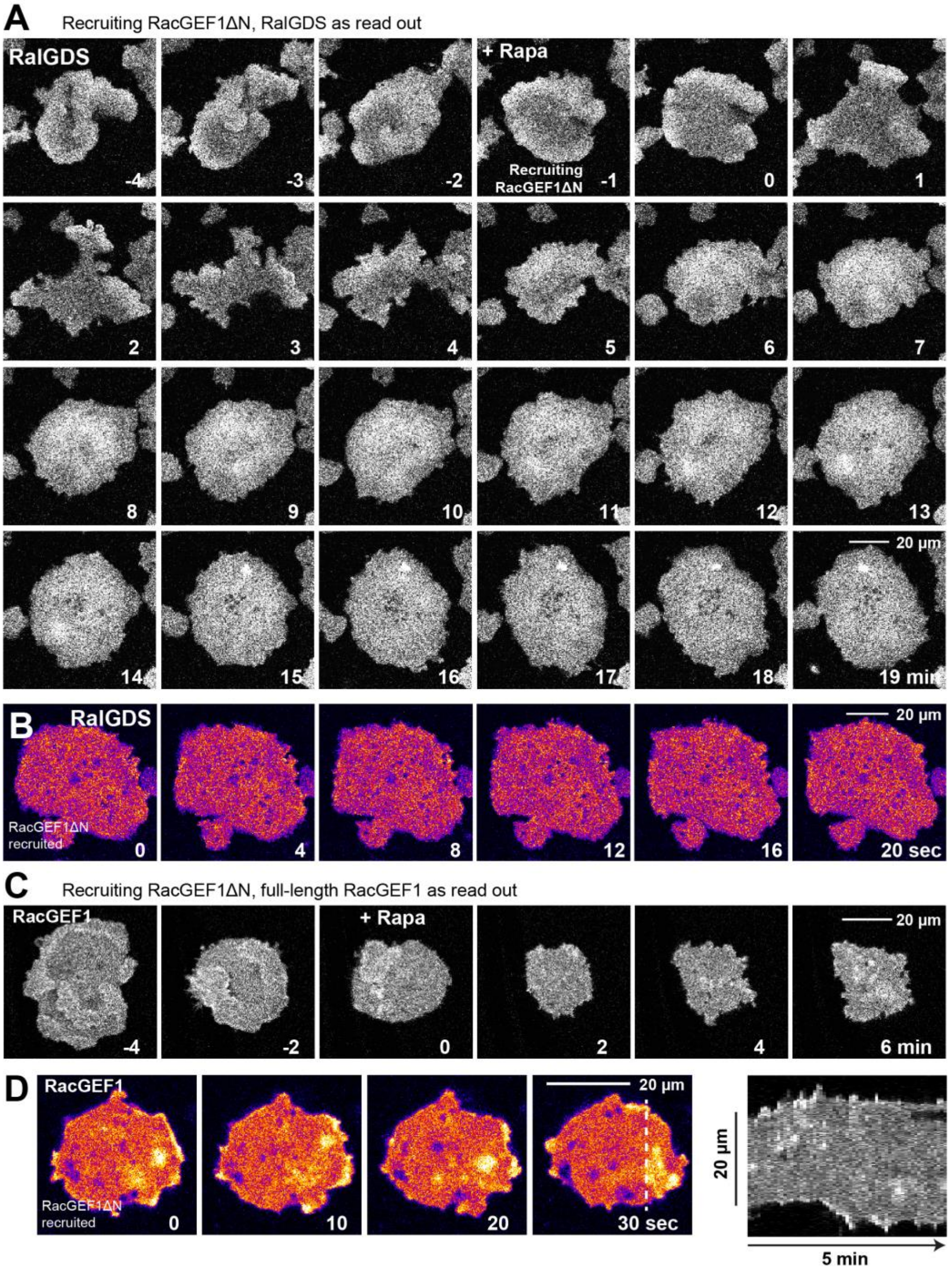

Appendix Figure S7. Effects on waves after RacGEF1$^{\Delta N}$ recruitment by CID.

(A) Time lapse confocal images of RalGDS at the basal surface of a giant cell, which recruits RacGEF1$^{\Delta N}$ to membrane induced by rapamycin at time 0. **(B)** Time lapse confocal images of RalGDS after RacGEF1$^{\Delta N}$ recruitment as shown with pseudo colors. **(C)** Time lapse confocal images of full-length RacGEF1 at the basal surface of a giant cell, which recruits RacGEF1$^{\Delta N}$ to membrane induced by rapamycin at time 0. **(D)** Left, time lapse confocal images of full-length RacGEF1 after RacGEF1$^{\Delta N}$ recruitment as shown with pseudo colors. Right, kymograph of full-length RacGEF1 across the white dotted line in image. Scale bars represent 20 µm.

Appendix Table S1. Table of simulation parameters.

Parameter values for simulations

**Excitable Signal Transduction Network**			
D_{Fs}	2.2	D_{Rs}	1.5
a_{1s}	0.167	ε_s	0.1
a_{2s}	16.67	c_{1s}	43
a_{3s}	167	a_{3s} (Inp54p)	182
a_{4s}	1.44	c_{1s} (PKBA)	43.7
a_{5s}	1.47		
U_b	0.3		
**Wave Characteristics (Fig. 3)**			
D_{Fs}	0.1	c_{1s} (R_{high})	60
ε_s	0.1	c_{1s} (R_{low})	55
D_{Rs}	0.4	a_{3s} (high)	1.1
D_{Fs} (2D)	0.1	a_{3s} (low)	0.98
D_{Rs} (2D)	0.1		
**Cytoskeletal Excitable Network**			
D_{Fc}	1	D_{Rc}	1
a_{1c}	0.167	ε_c	0.3
a_{2c}	16.67	c_{1c}	40
a_{3c}	167	a_{3c} (PKBA)	300
a_{4c}	1.44	c_{1c} (RacGEF1)	5.8
a_{5c}	1.47	a_{1c} (RacGEF1)	-49.8
**CEN - STEN Interconnections**			
D_{Zc}	1	p_3	0.24
p_1	2	p_4	0.48
p_2	2	s_c	2
**Level Set Model**			
K	0.098	D	0.064
B	6.09		

Movie EV1. Time-lapse confocal videos of PH_{crac} (red) and RalGDS (green) on the basal surface of a giant cell. Images were acquired every 5 sec and the videos are shown at 10 frame/sec. Related to Figure 1.

Movie EV2. Time-lapse simulation videos of CEN (left) and STEN (right) showing wave propagation in 2D space before and after the perturbation (increasing positive feedback of STEN, Inp54p-type) is introduced. Related to Figure 4.

Movie EV3. Time-lapse confocal videos of LimE on the basal surface of giant cells before and after recruiting Inp54p. Rapamycin was added at time 07:30 min:sec. Images were acquired every 15 sec and the videos are shown at 20 frame/sec. Related to Figure 4.

Movie EV4. Time-lapse confocal videos of PH_{crac} on the basal surface of giant cells before and after recruiting Inp54p. Rapamycin was added at time 06:15 min:sec. Images were acquired every 15 sec and the videos are shown at 20 frame/sec. Related to Figure 4.

Movie EV5. Time-lapse confocal videos of Inp54p (left) and PH_{crac} (right) on the basal surface of giant cells before and after recruiting Inp54p, under the treatment of LatrunculinA. Rapamycin was added at time 12:30 min:sec. Images were acquired every 15 sec and the videos are shown at 20 frame/sec. Related to Figure 4.

Movie EV6. Time-lapse confocal videos of LimE on the basal surface of giant cells before and after recruiting PKBA. Rapamycin was added at time 07:45 min:sec. Images were acquired every 15 sec and the videos are shown at 20 frame/sec. Related to Figure 5.

Movie EV7. Time-lapse confocal videos of LimE (green) and PH_{crac} (red) on the basal surface of a giant cell before and after recruiting PKBA. Rapamycin was added at time 07:45 min:sec. Images were acquired every 15 sec and the videos are shown at 15 frame/sec. Related to Figure 5.

Movie EV8. Time-lapse confocal videos of Inp54p (left) and PH_{crac} (right) on the basal surface of giant cells before and after recruiting PKBA, under the treatment of LatrunculinA. Rapamycin was added at time 12:30 min:sec. The cell labeled with “No recruitment” does not have PKBA recruitment thus can serve as internal control. Images were acquired every 15 sec and the videos are shown at 20 frame/sec. Related to Figure 4.

Movie EV9. Time-lapse simulation videos of CEN (left) and STEN (right) showing wave propagation in 2D space before and after the perturbation (lowering threshold of CEN and increasing threshold of STEN, PKBA-type) is introduced. Related to Figure 5.

Movie EV10. Time-lapse confocal videos of LimE in single cells before and after recruiting RacGEF1$^{\Delta N}$. Rapamycin was added at time 06:15 min:sec. Images were acquired every 15 sec and the video is shown at 15 frame/sec. Related to Figure 6.

Movie EV11. Time-lapse confocal videos of LimE on the basal surface of giant cells before and after recruiting RacGEF1$^{\Delta N}$. Rapamycin was added at time 07:30 min:sec. Images were acquired every 15 sec and the video is shown at 15 frame/sec. Related to Figure 6.

Movie EV12. Time-lapse simulation videos of CEN (left) and STEN (right) showing wave propagation in 2D space before and after the perturbation (increasing positive feedback of CEN and inhibiting STEN, RacGEF1-type) is introduced. Related to Figure 6.

Movie EV13. Time-lapse confocal videos of PH_{crac} on the basal surface of giant cells before and after recruiting RacGEF1$^{\Delta N}$. Rapamycin was added at time 07:30 min:sec. Images were acquired every 15 sec and the video is shown at 15 frame/sec. Related to Figure 7.

Movie EV14. Time-lapse confocal videos of LimE in cells after recruiting PKBA (left) or RacGEF1$^{\Delta N}$ (right). Images were acquired every 2 sec and the videos are shown at 15 frame/sec. Related to Figure 8.

Movie EV15. Level set simulations of cell morphology changes before and after the different perturbations are introduced. The corresponding perturbation is indicated in the title. Fluorescent green denotes higher levels of activity on the membrane. Related to Figure 8.
