# Supplementary figures and images for: Wave patterns organize cellular protrusions and control cortical dynamics (part 1 of 2)
Source: Mol Syst Biol. 2019 Mar 12;15(3):e8585. doi: 10.15252/msb.20188585 (PMC6413885; doi:10.15252/msb.20188585)

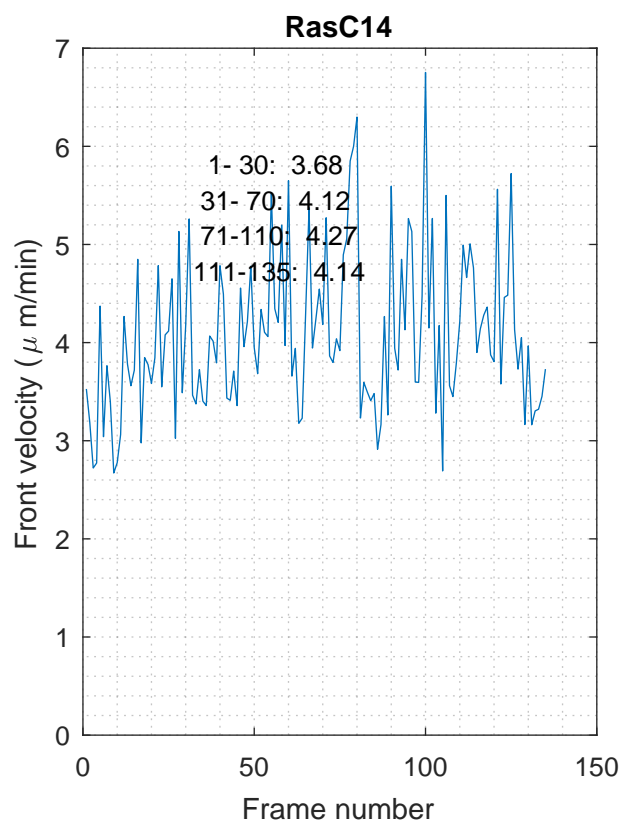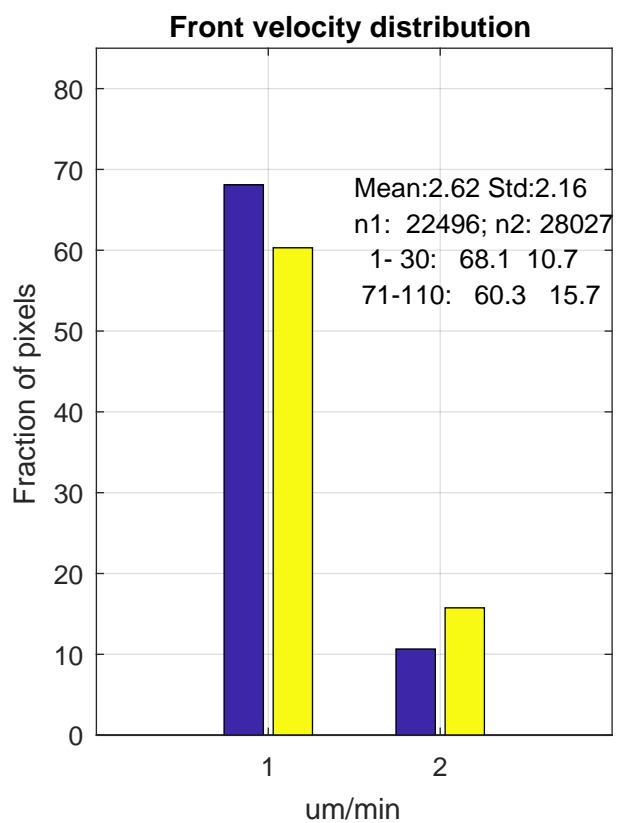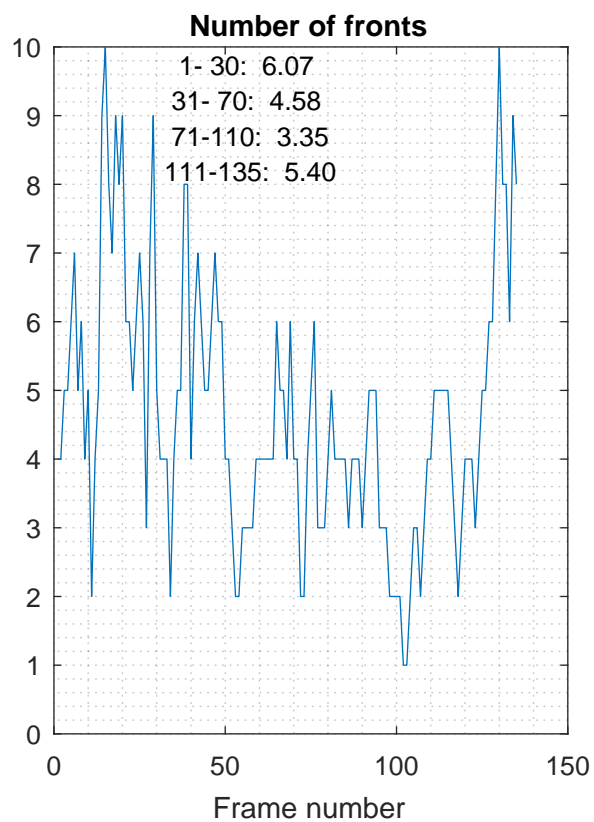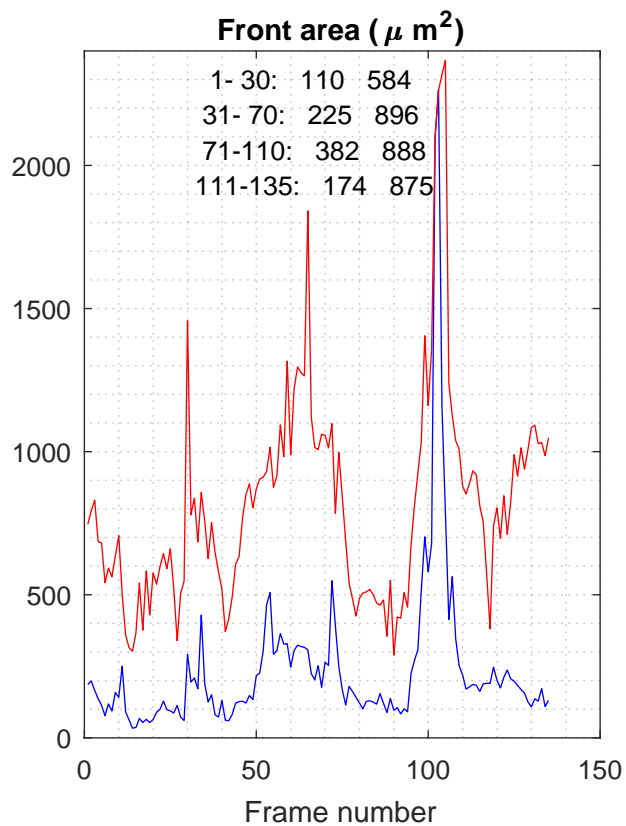

Supplement: Supplementary file 17 — Source Data for Appendix [file MSB-15-e8585-s025.zip › Source_data_for_Appendix/Appendix_Fig_S4/S4C/RasC14.pdf]

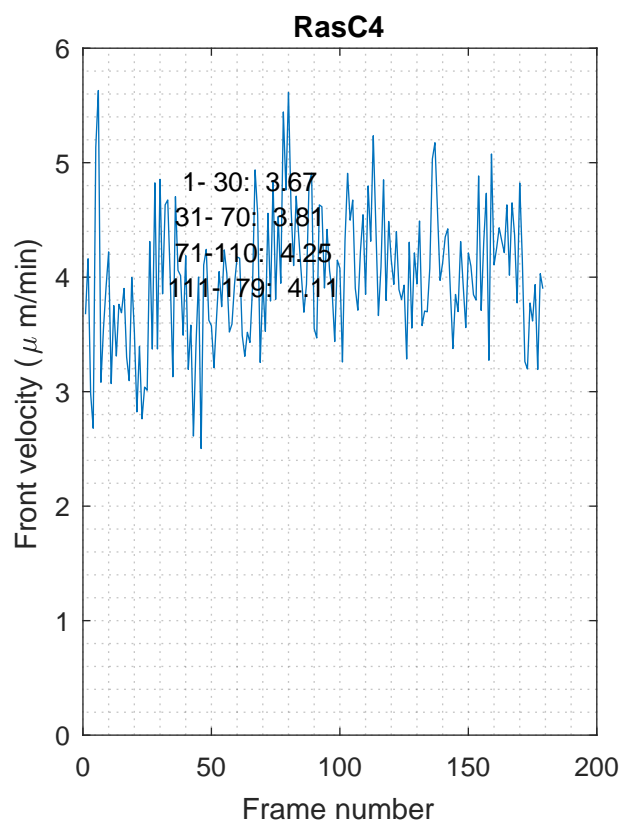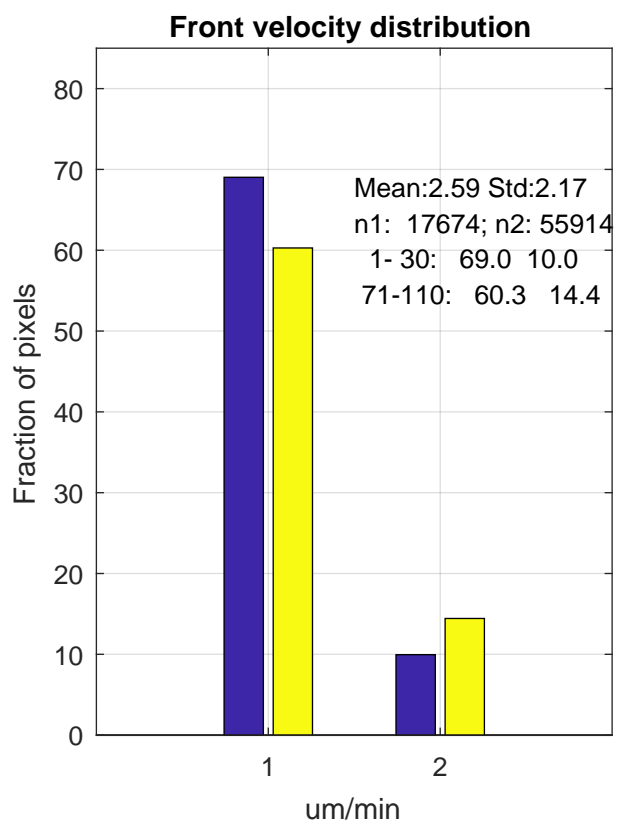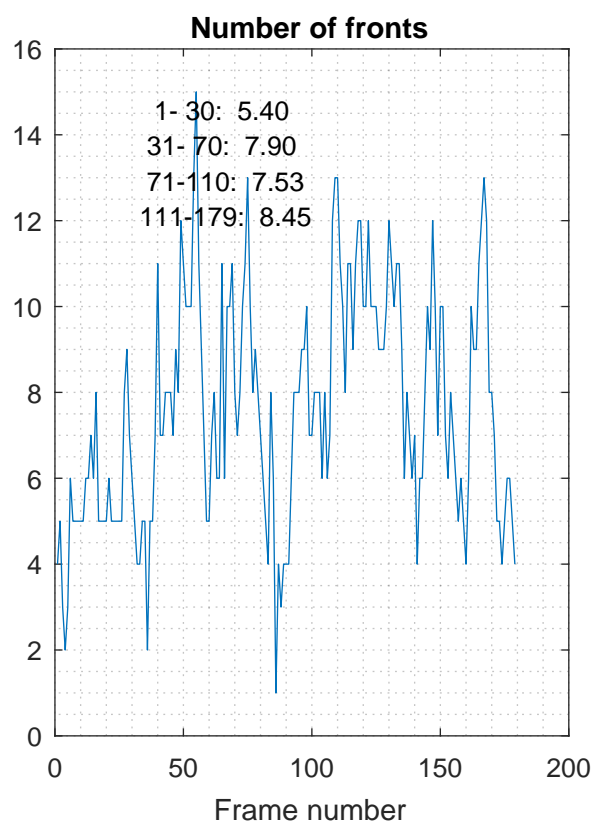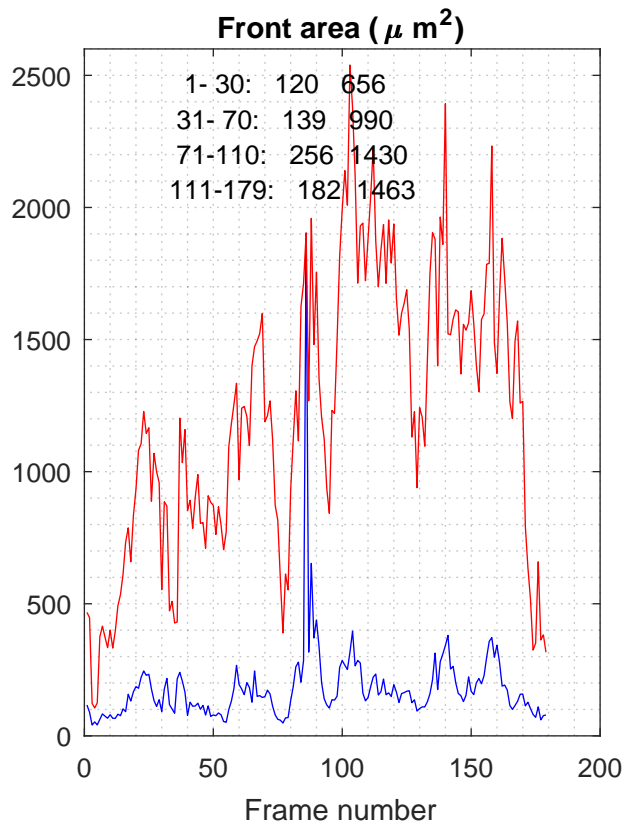

Supplement: Supplementary file 17 — Source Data for Appendix [file MSB-15-e8585-s025.zip › Source_data_for_Appendix/Appendix_Fig_S4/S4C/RasC4.pdf]

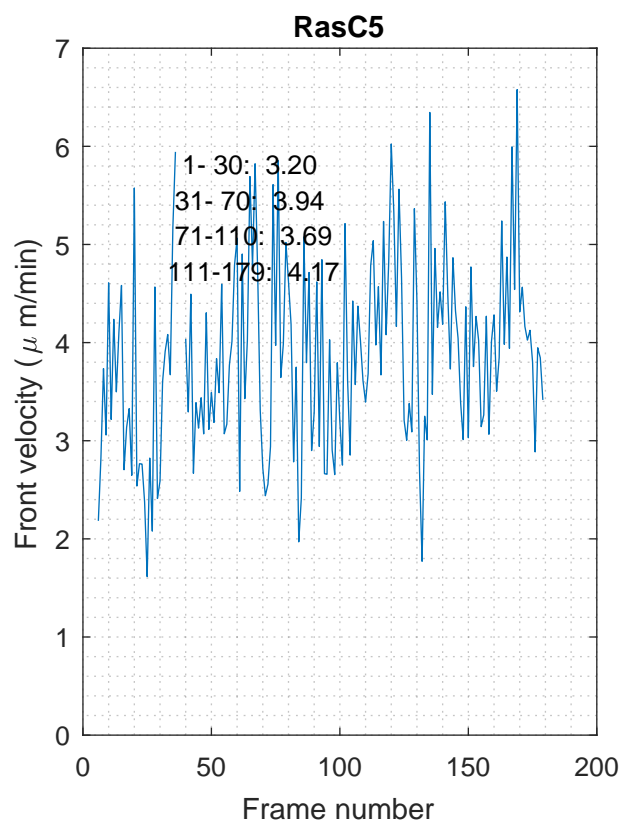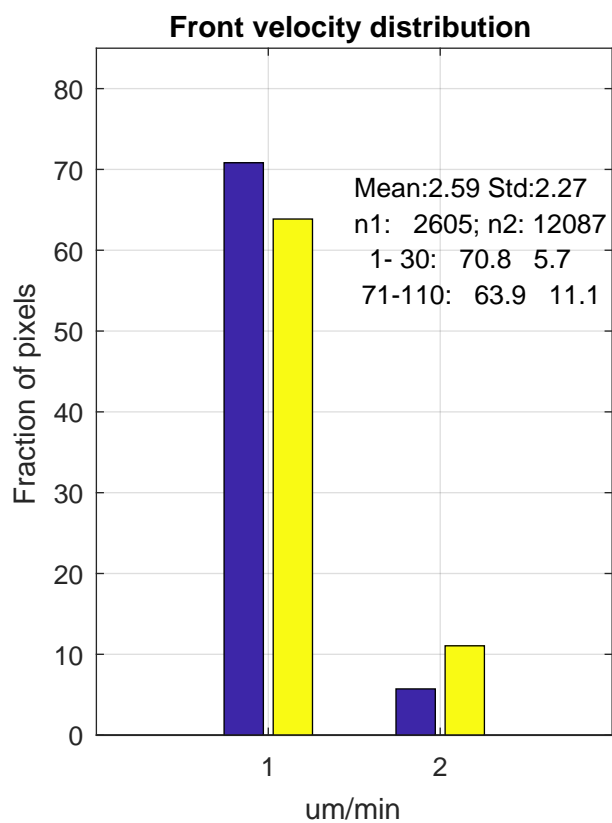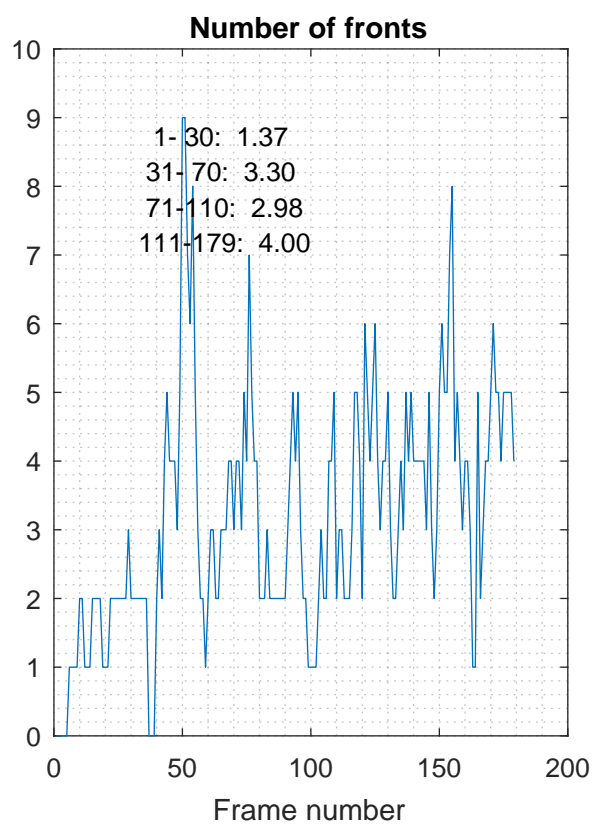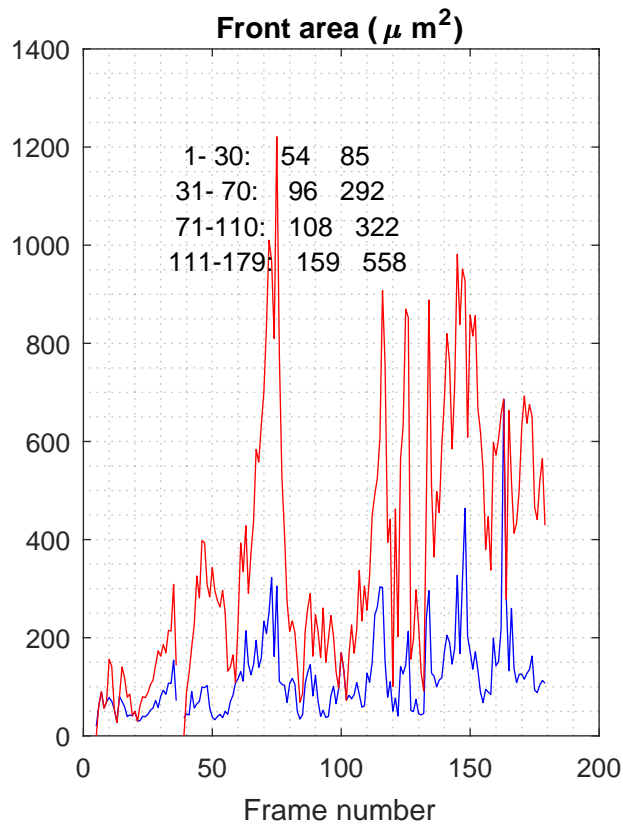

Supplement: Supplementary file 17 — Source Data for Appendix [file MSB-15-e8585-s025.zip › Source_data_for_Appendix/Appendix_Fig_S4/S4C/RasC5.pdf]

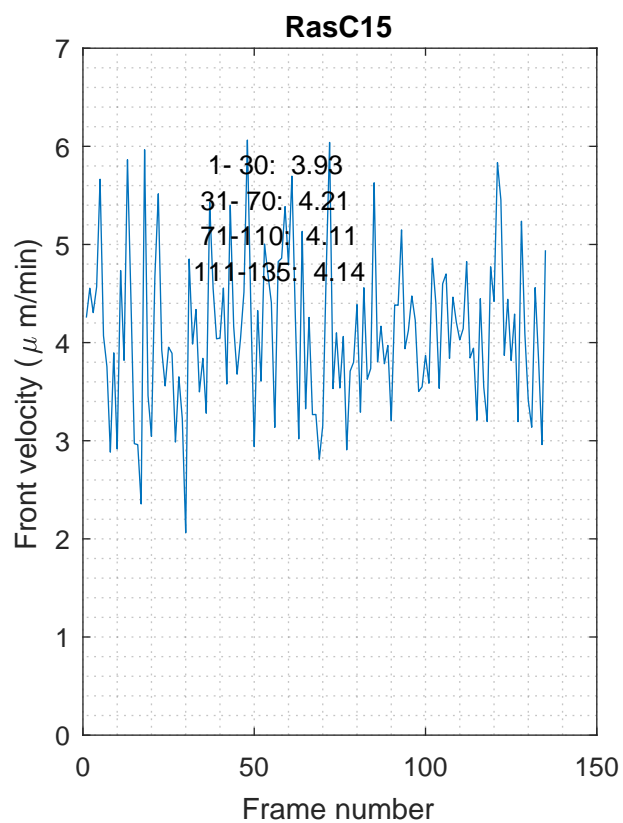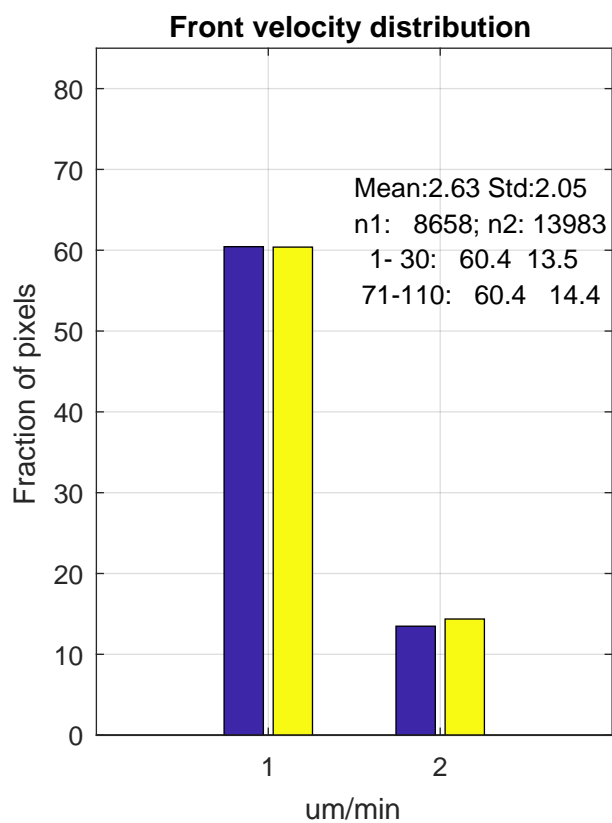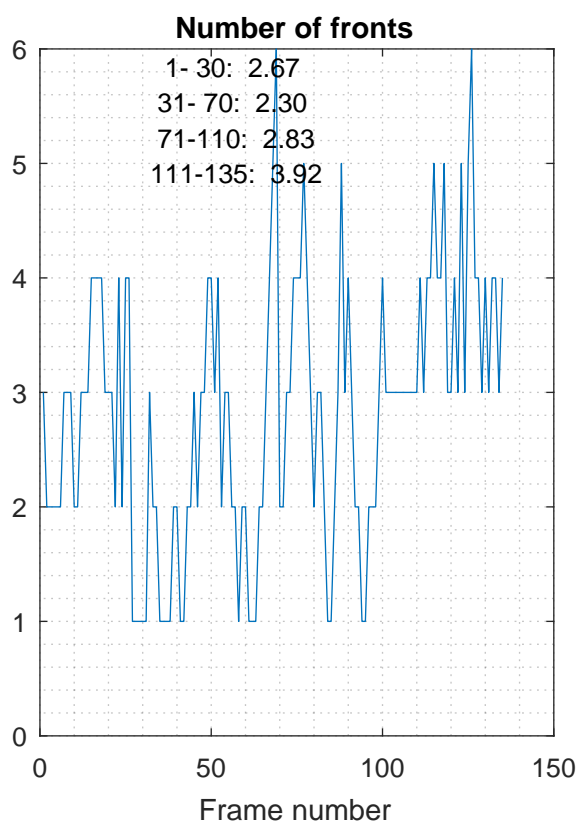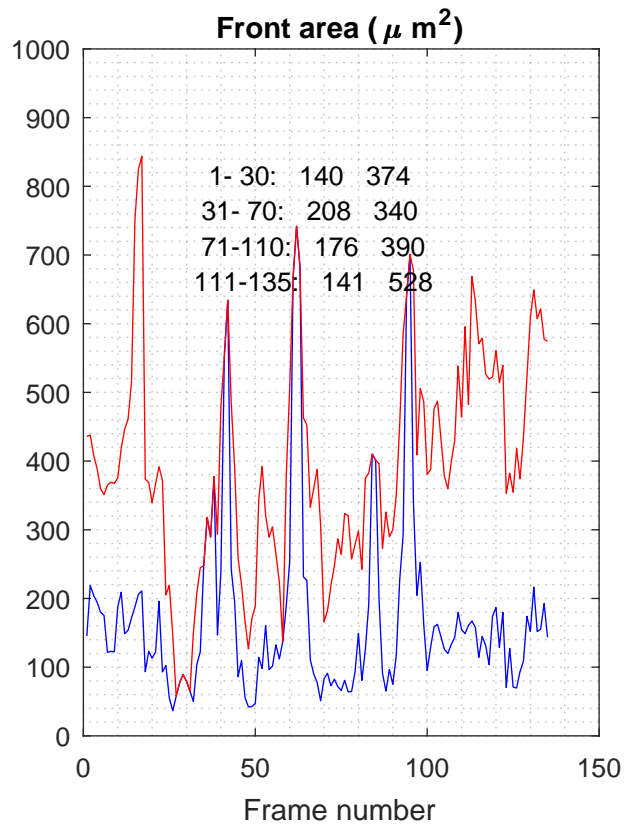

Supplement: Supplementary file 17 — Source Data for Appendix [file MSB-15-e8585-s025.zip › Source_data_for_Appendix/Appendix_Fig_S4/S4C/RasC15.pdf]

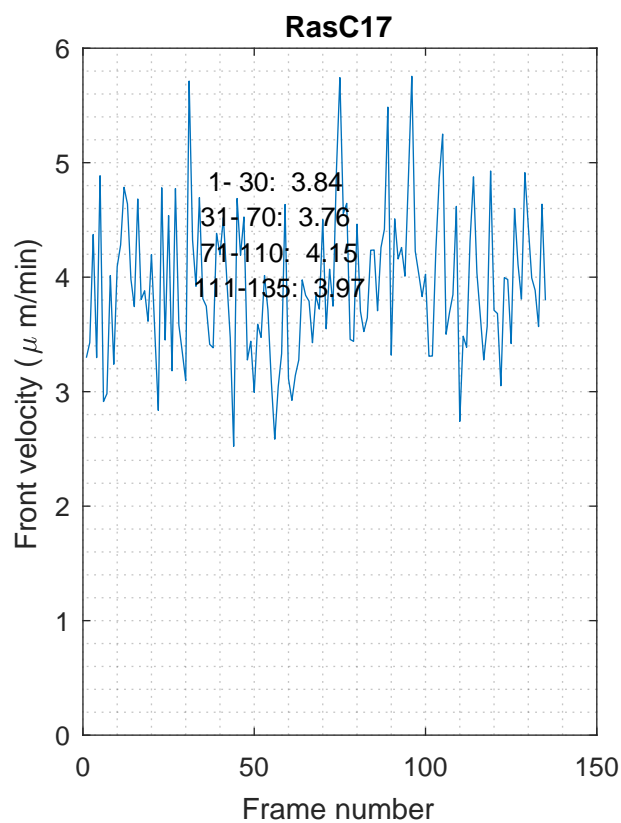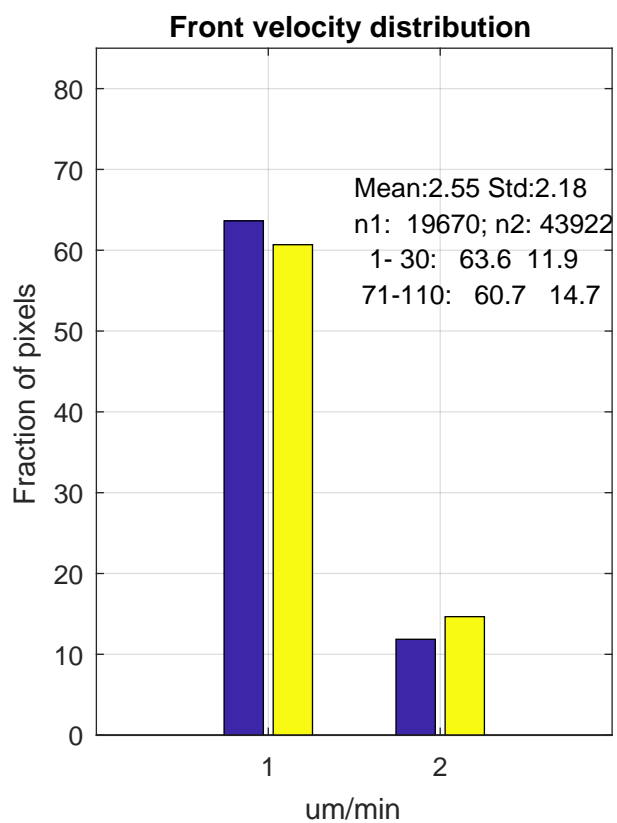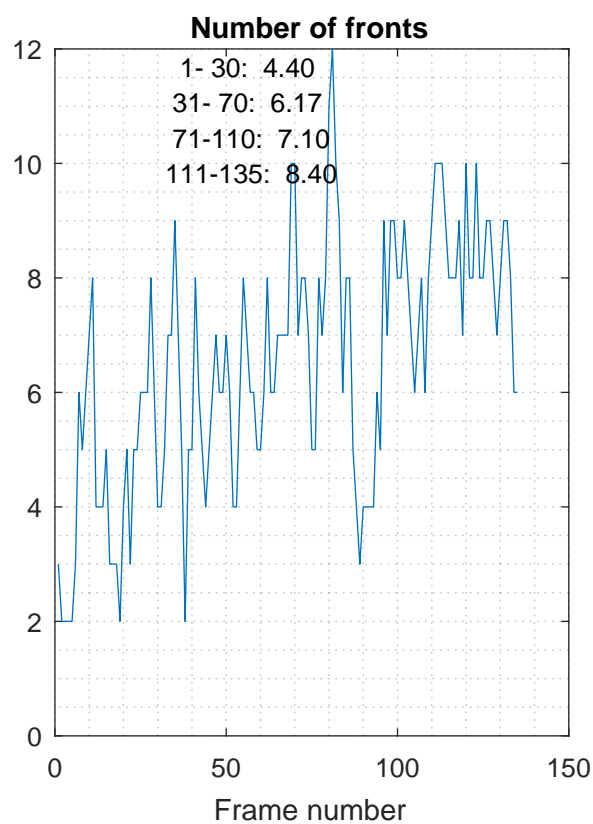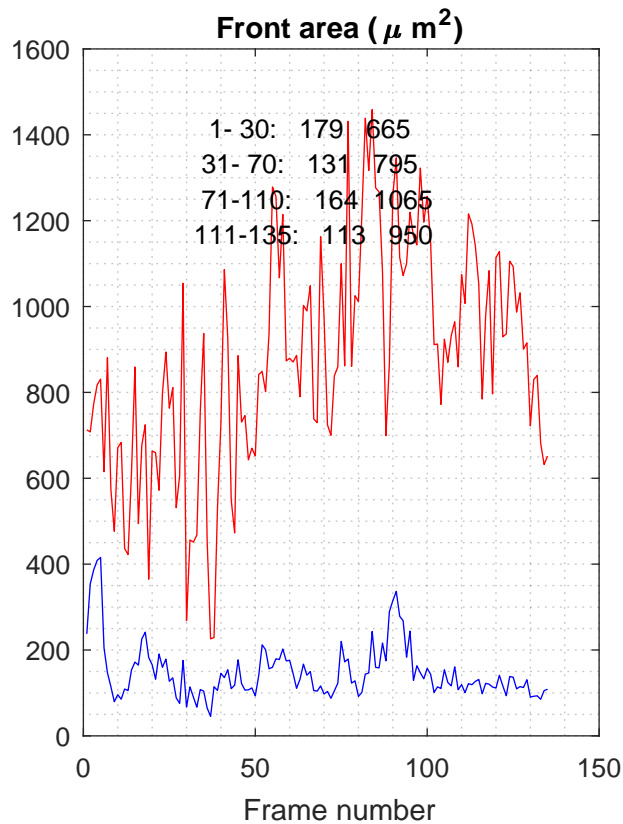

Supplement: Supplementary file 17 — Source Data for Appendix [file MSB-15-e8585-s025.zip › Source_data_for_Appendix/Appendix_Fig_S4/S4C/RasC17.pdf]

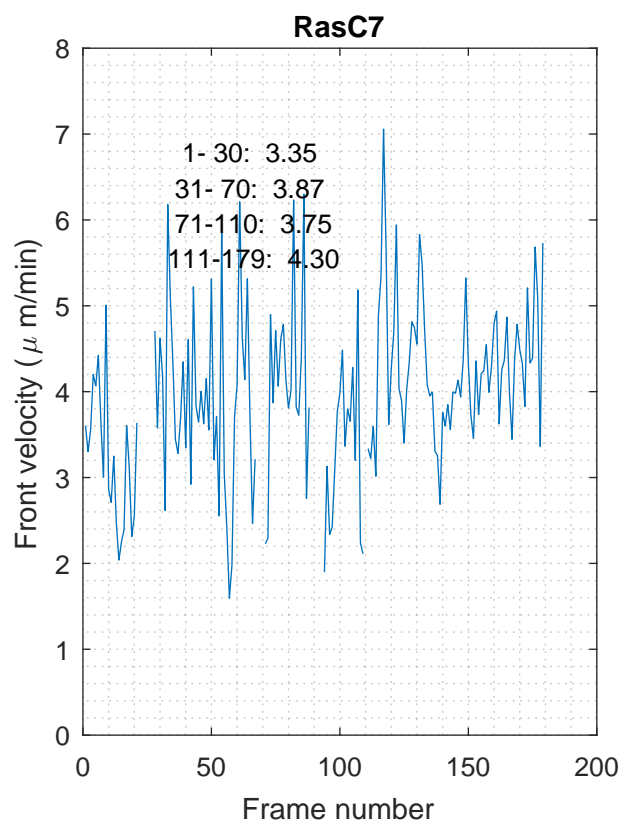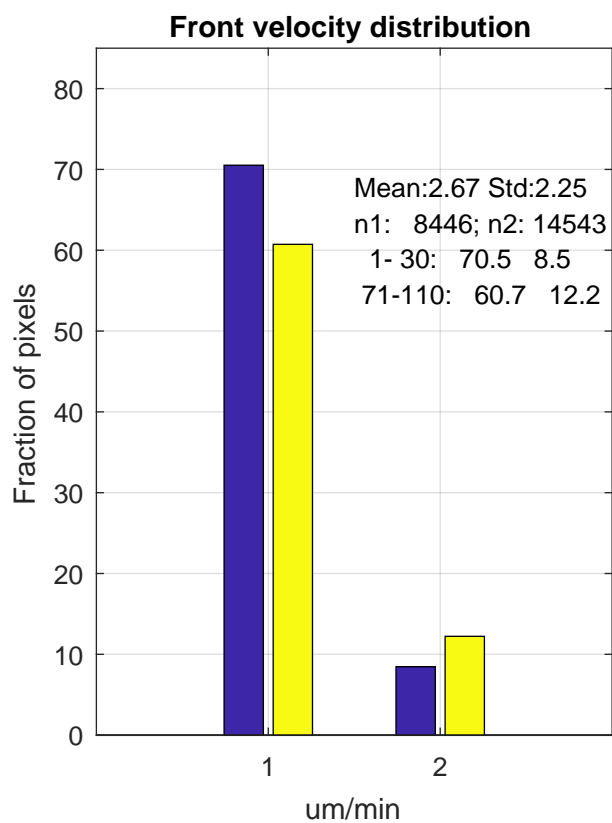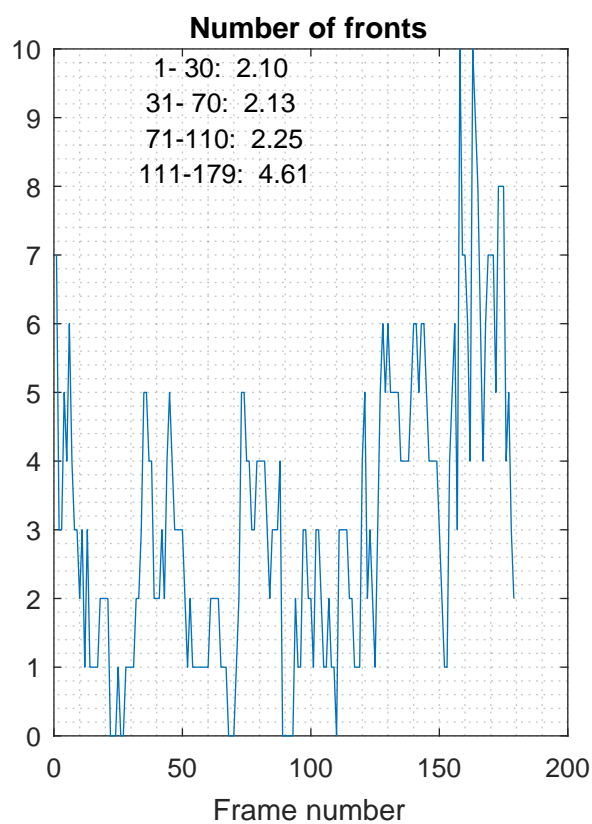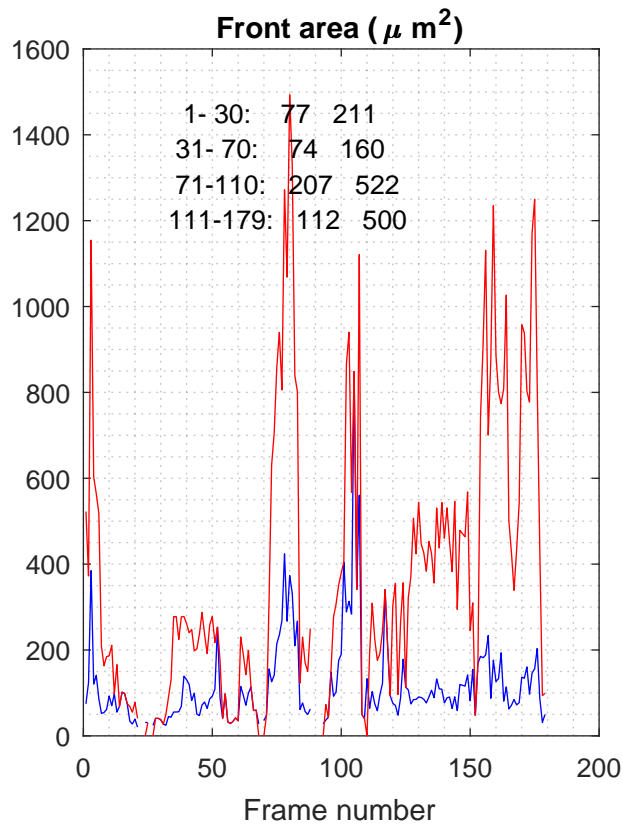

Supplement: Supplementary file 17 — Source Data for Appendix [file MSB-15-e8585-s025.zip › Source_data_for_Appendix/Appendix_Fig_S4/S4C/RasC7.pdf]

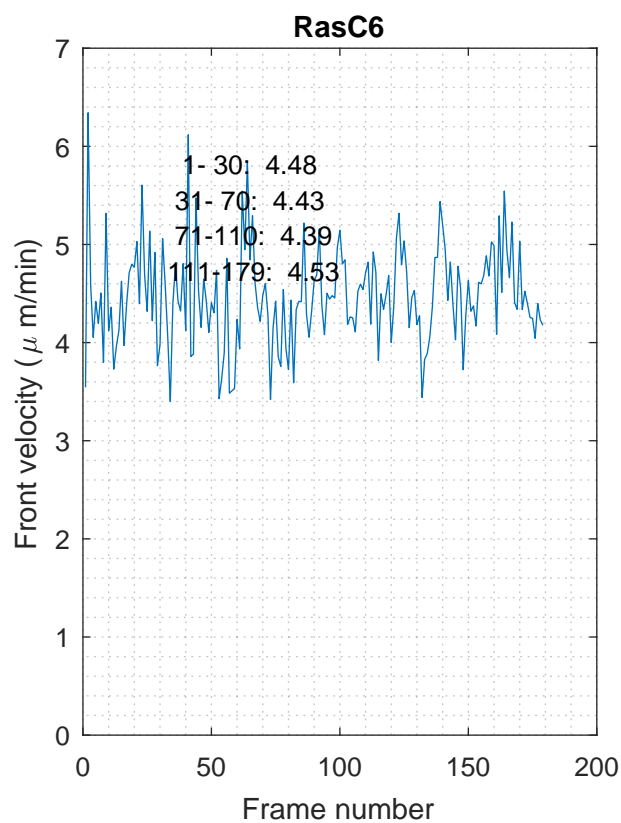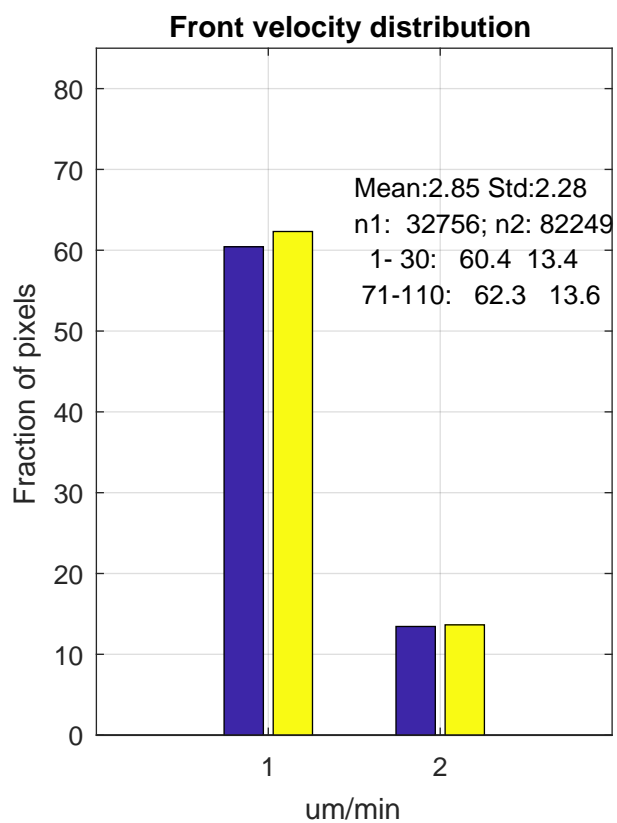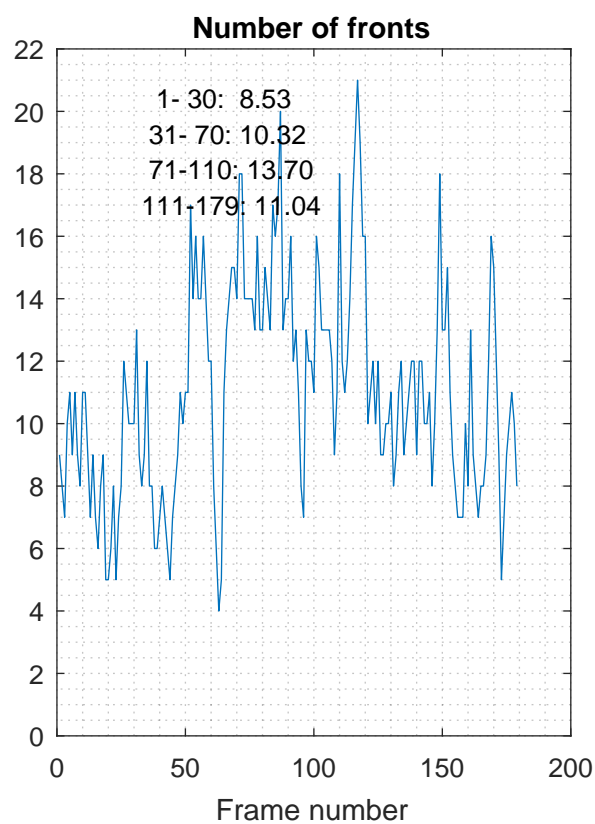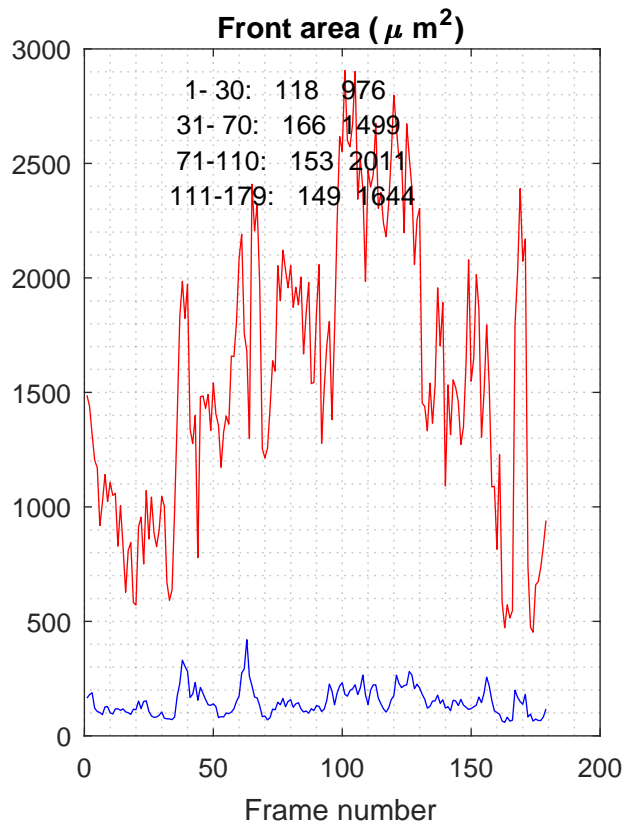

Supplement: Supplementary file 17 — Source Data for Appendix [file MSB-15-e8585-s025.zip › Source_data_for_Appendix/Appendix_Fig_S4/S4C/RasC6.pdf]

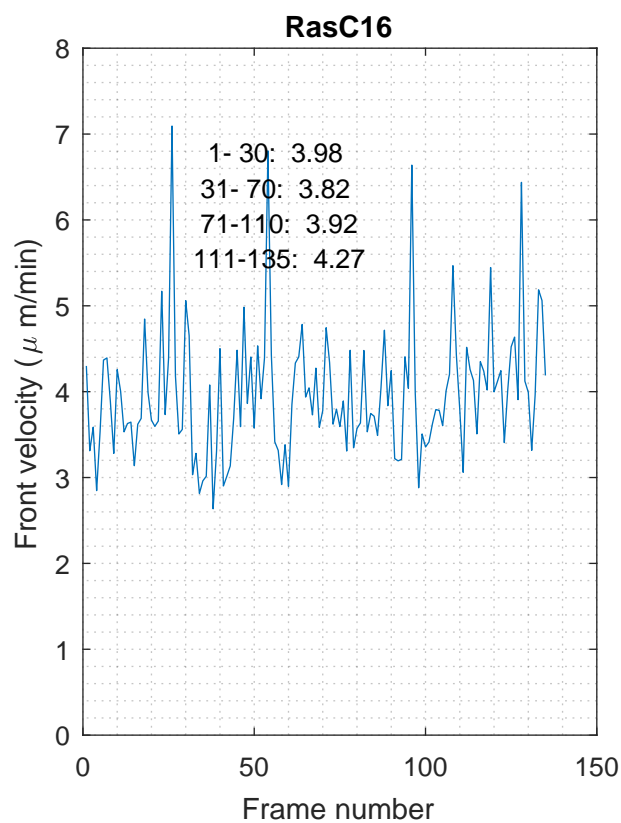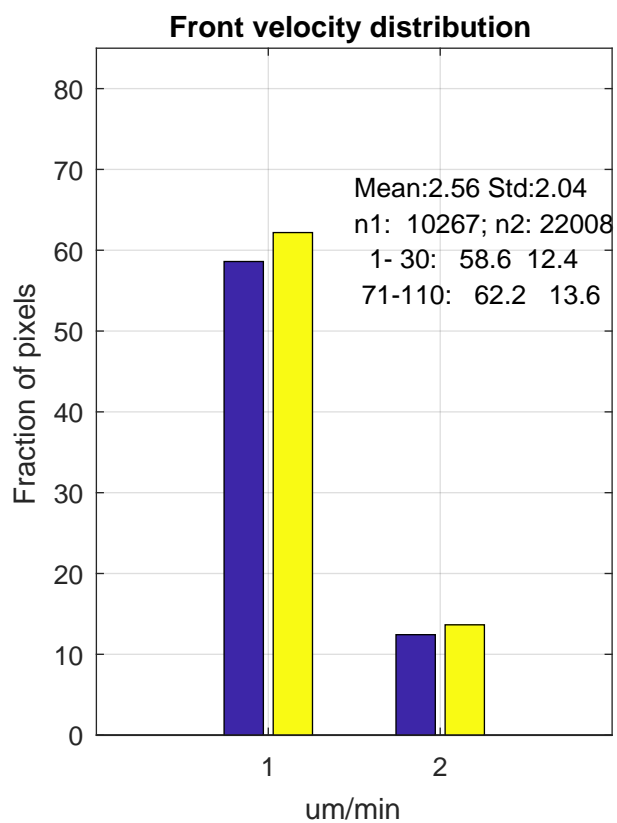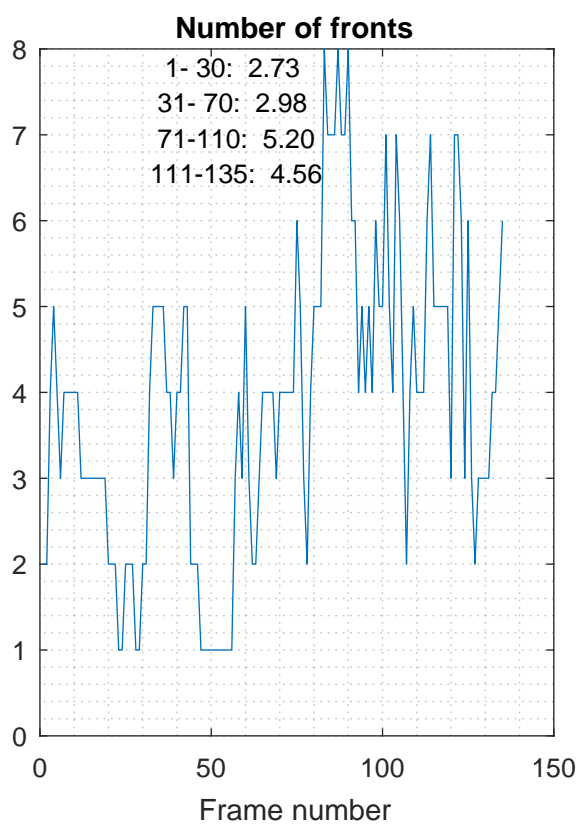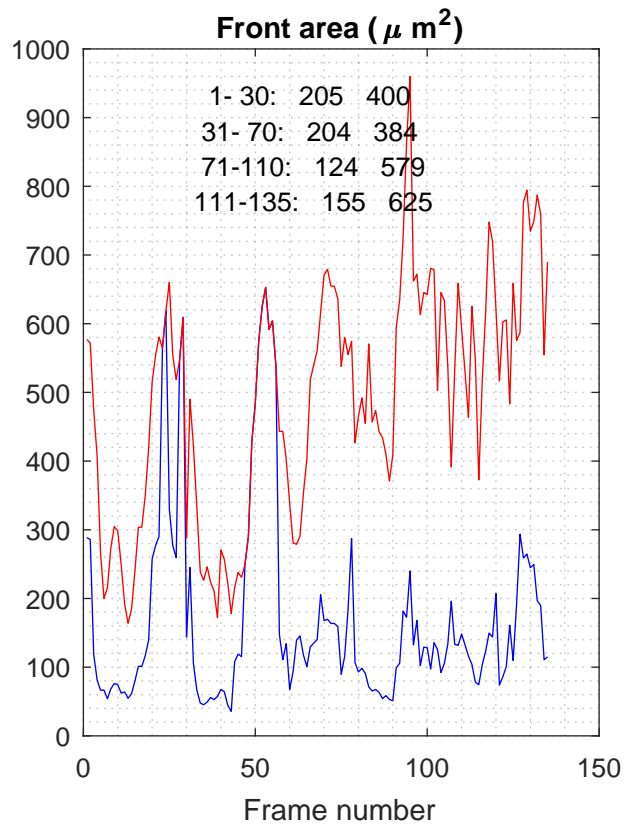

Supplement: Supplementary file 17 — Source Data for Appendix [file MSB-15-e8585-s025.zip › Source_data_for_Appendix/Appendix_Fig_S4/S4C/RasC16.pdf]

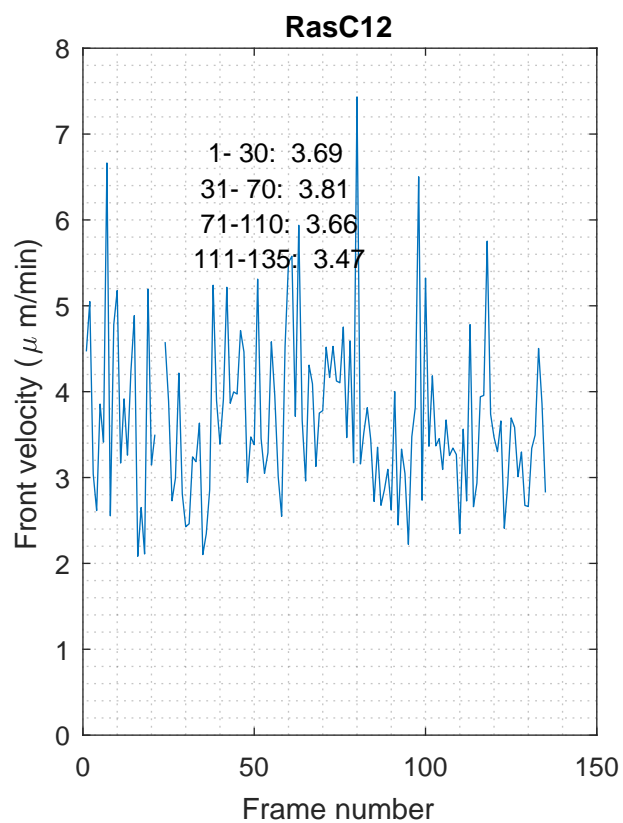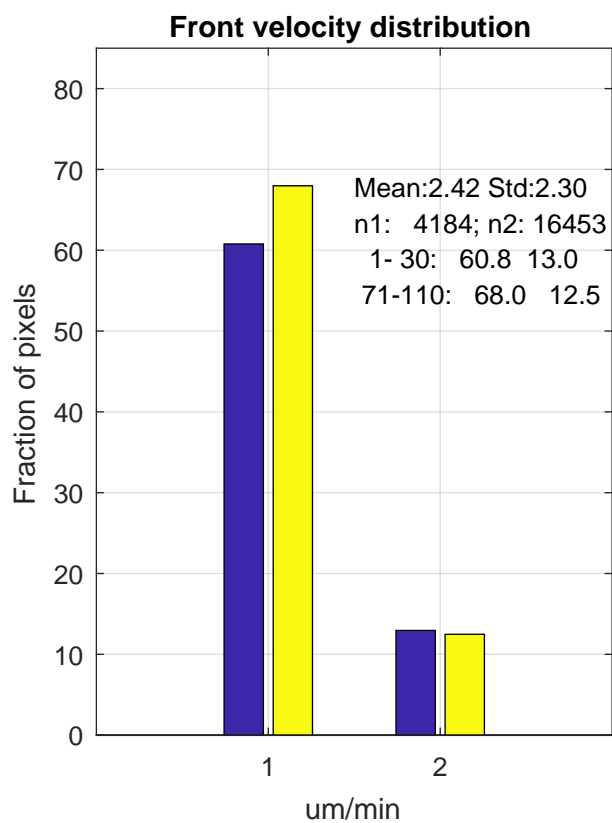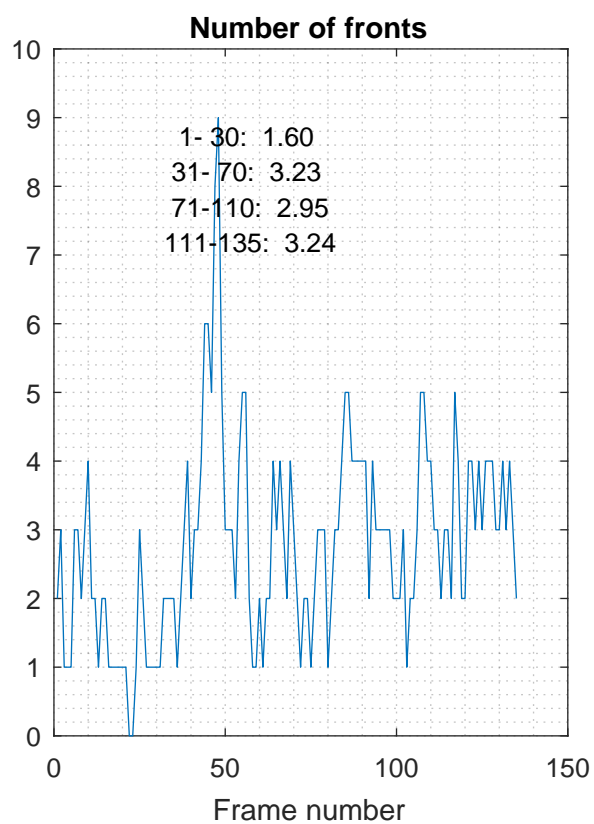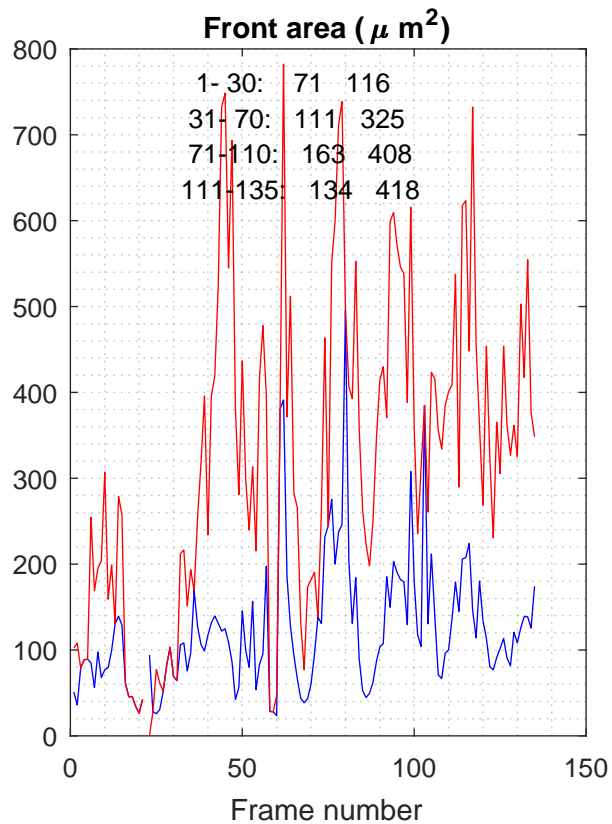

Supplement: Supplementary file 17 — Source Data for Appendix [file MSB-15-e8585-s025.zip › Source_data_for_Appendix/Appendix_Fig_S4/S4C/RasC12.pdf]

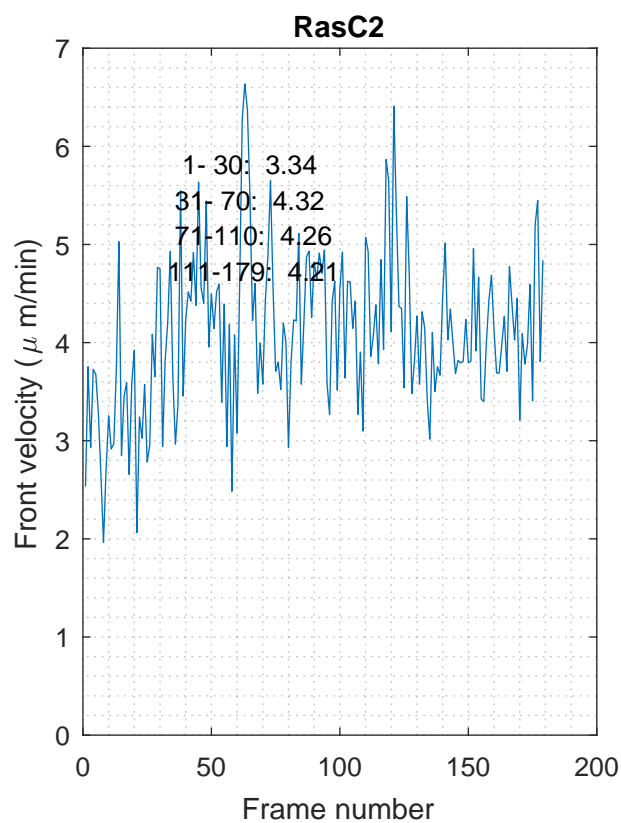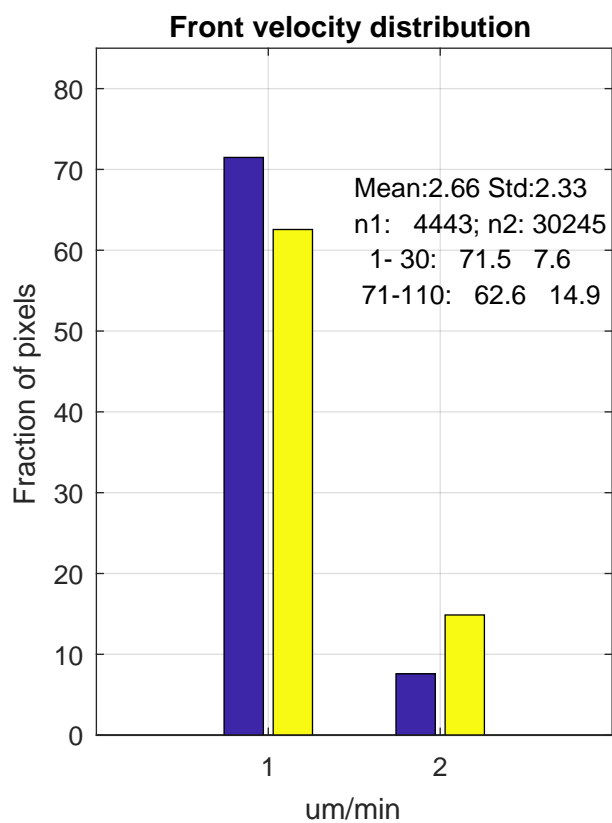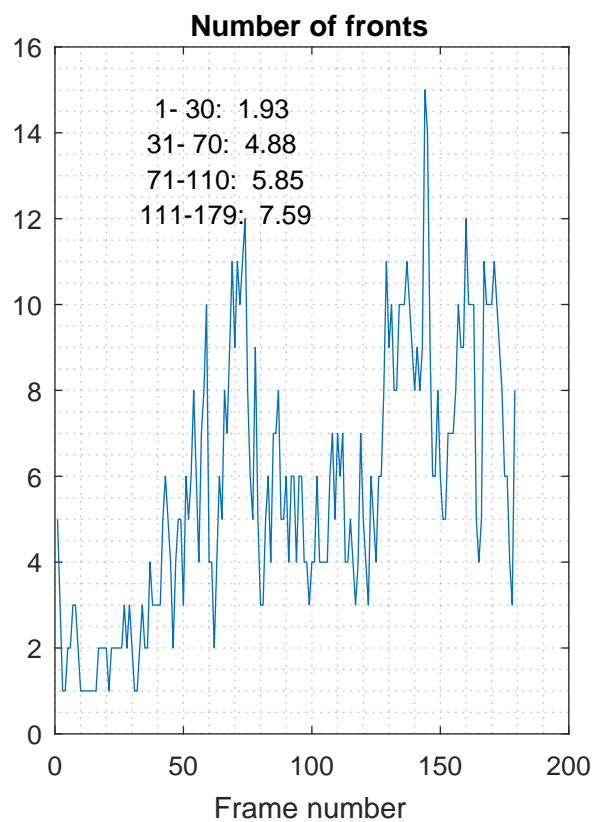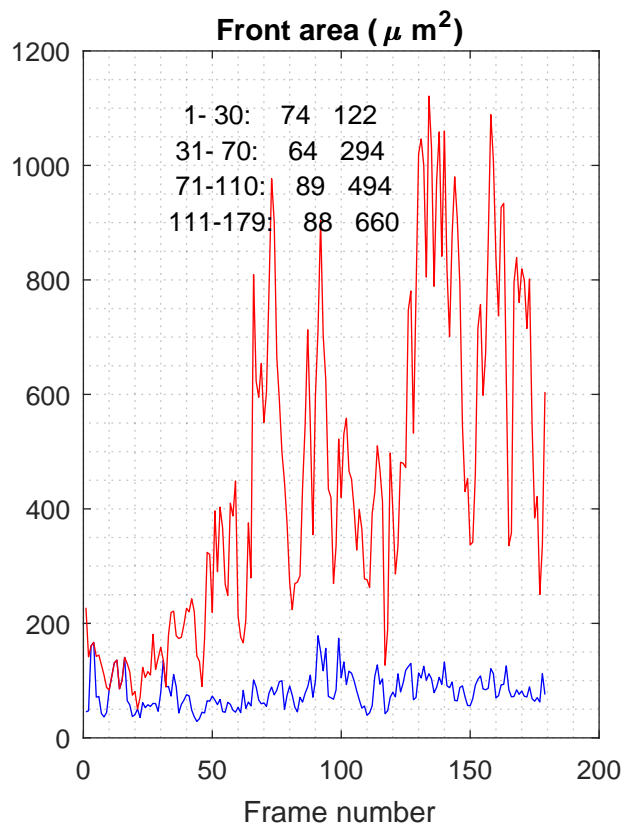

Supplement: Supplementary file 17 — Source Data for Appendix [file MSB-15-e8585-s025.zip › Source_data_for_Appendix/Appendix_Fig_S4/S4C/RasC2.pdf]

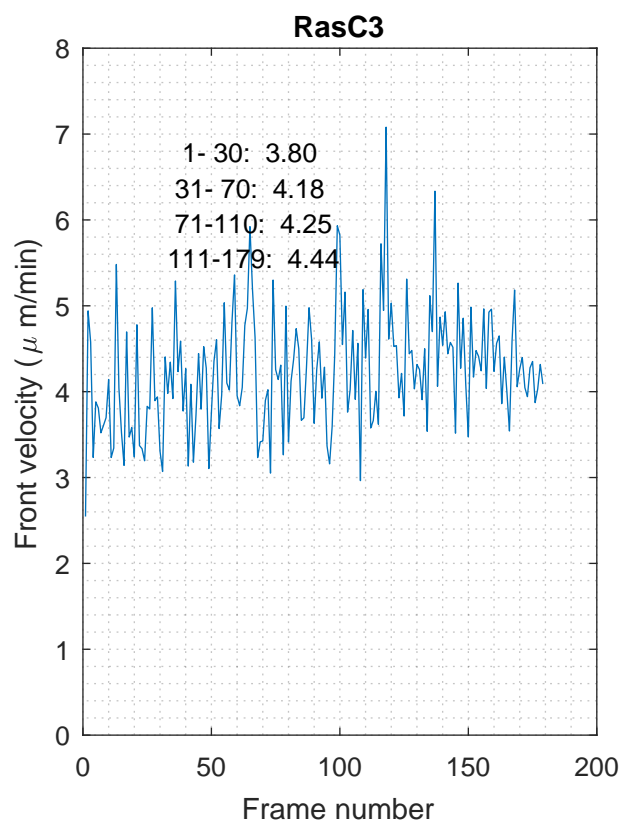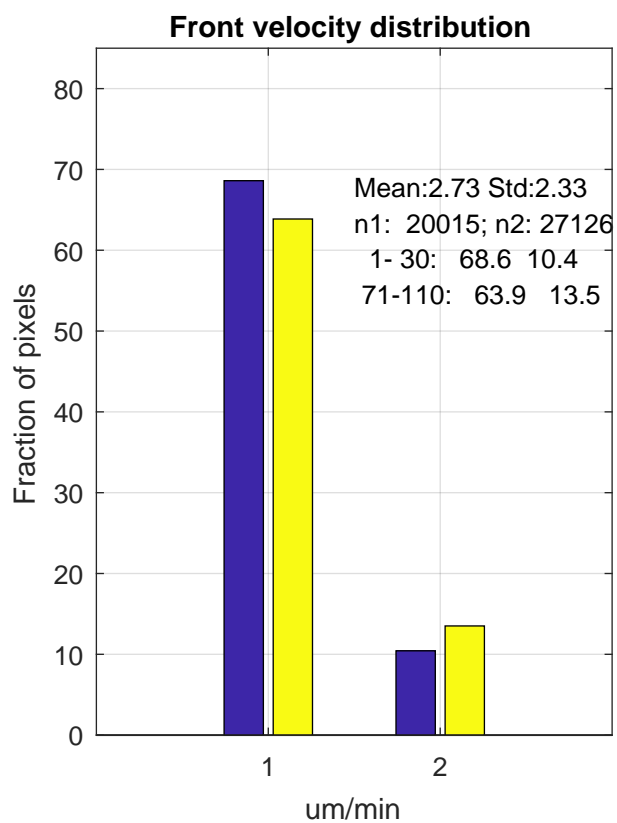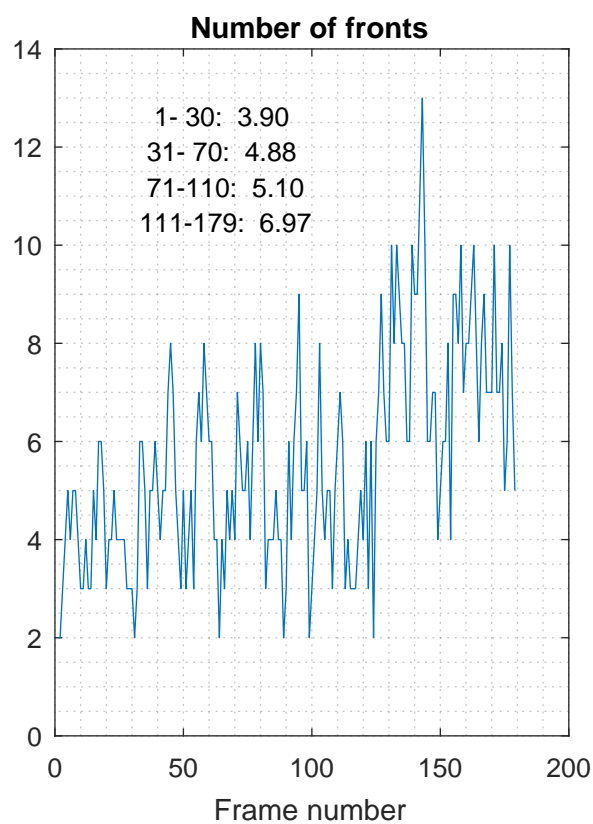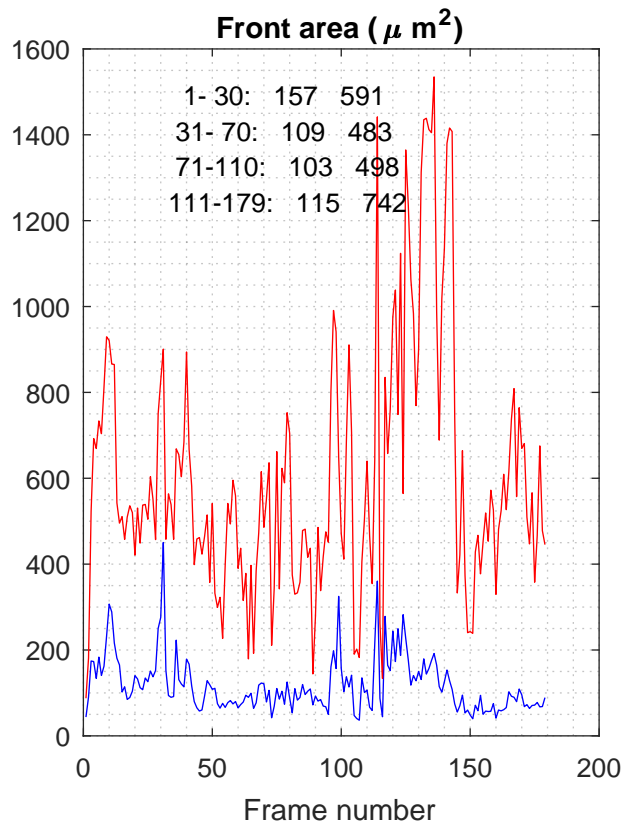

Supplement: Supplementary file 17 — Source Data for Appendix [file MSB-15-e8585-s025.zip › Source_data_for_Appendix/Appendix_Fig_S4/S4C/RasC3.pdf]

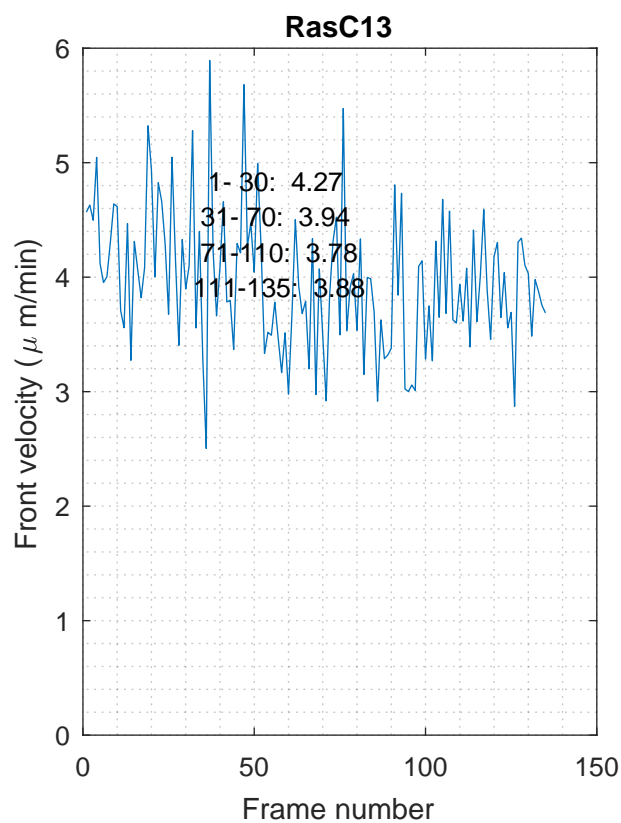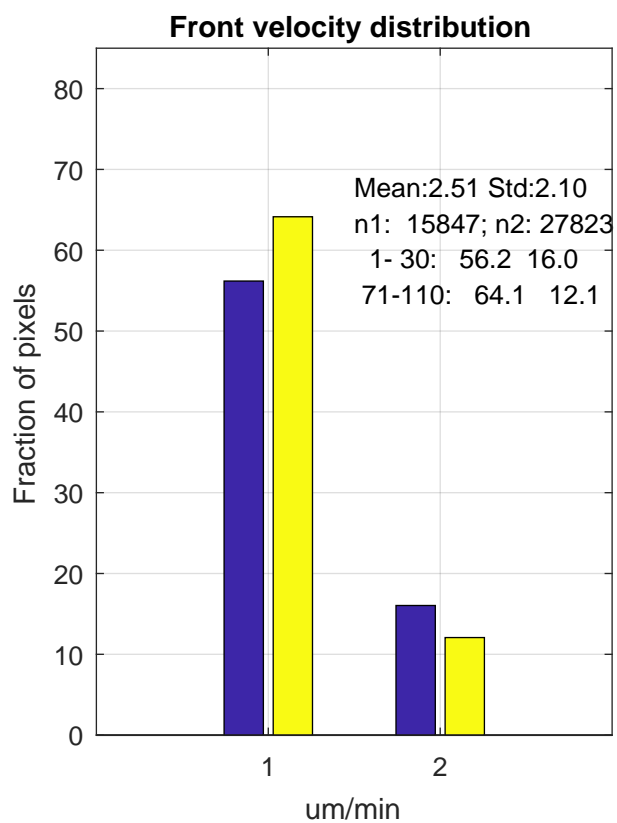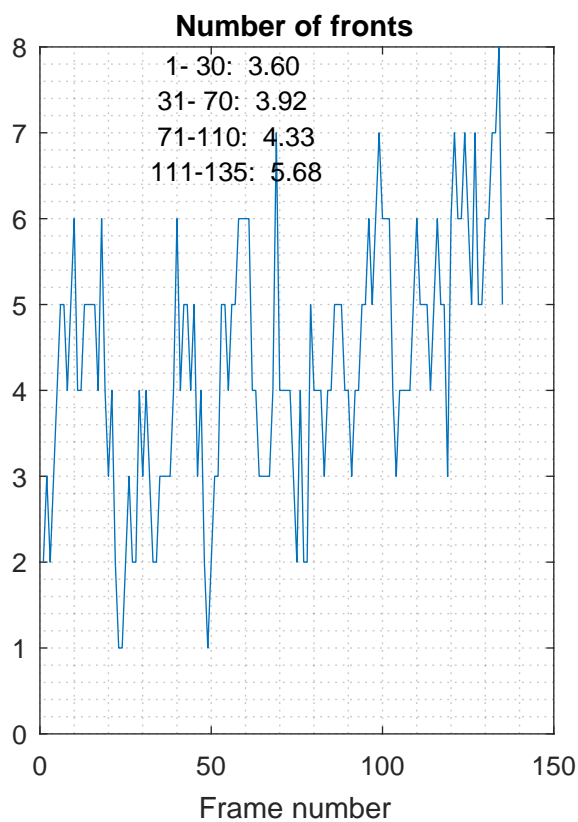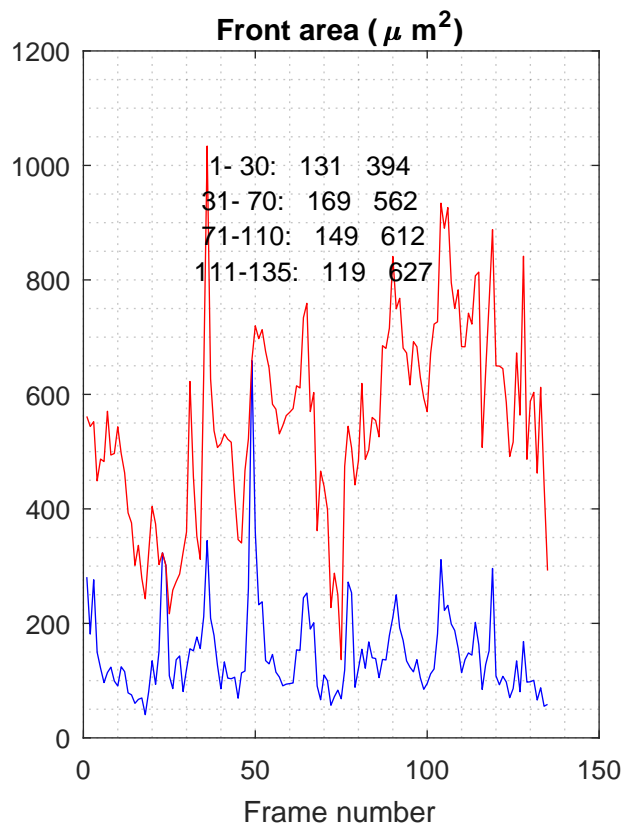

Supplement: Supplementary file 17 — Source Data for Appendix [file MSB-15-e8585-s025.zip › Source_data_for_Appendix/Appendix_Fig_S4/S4C/RasC13.pdf]

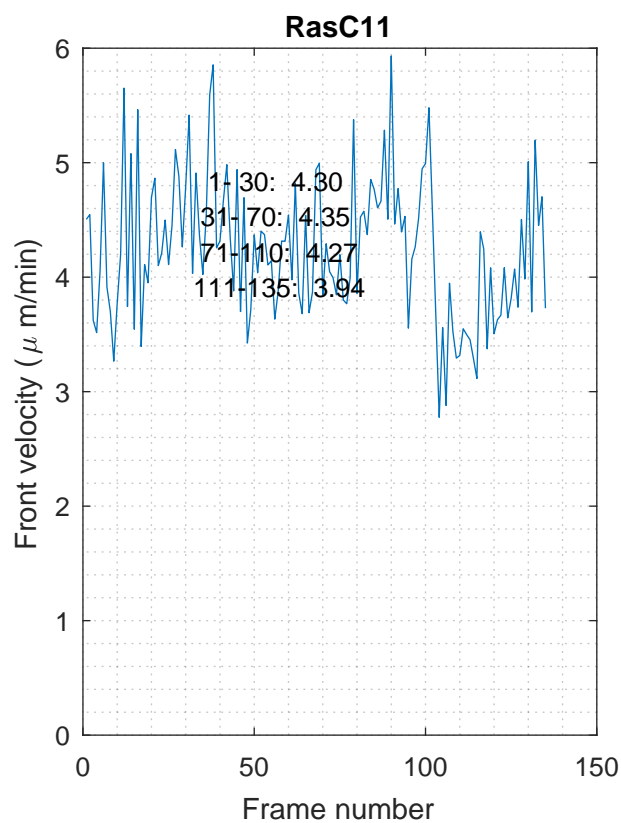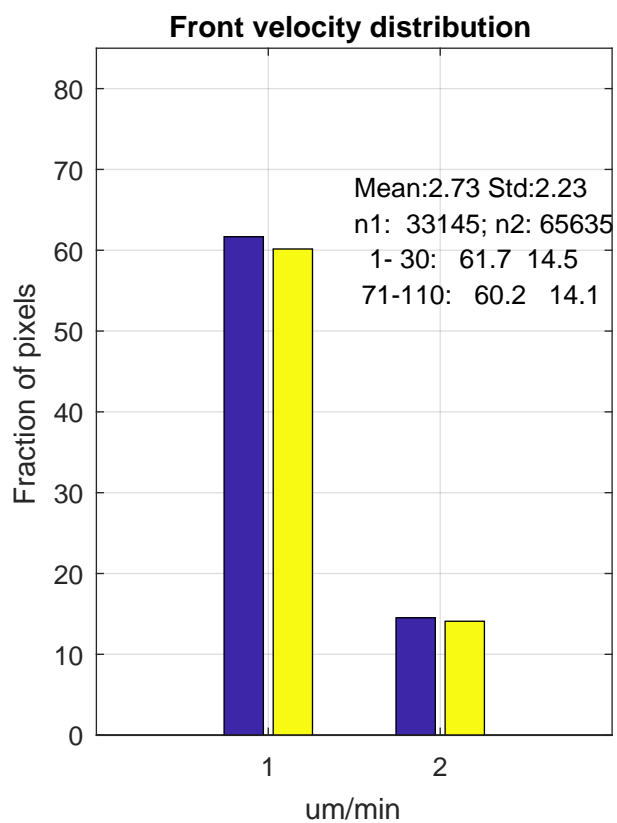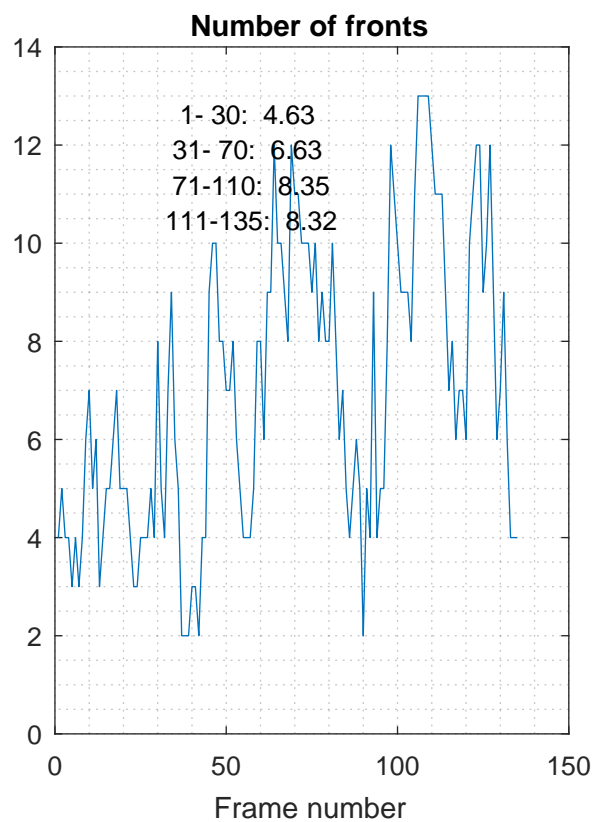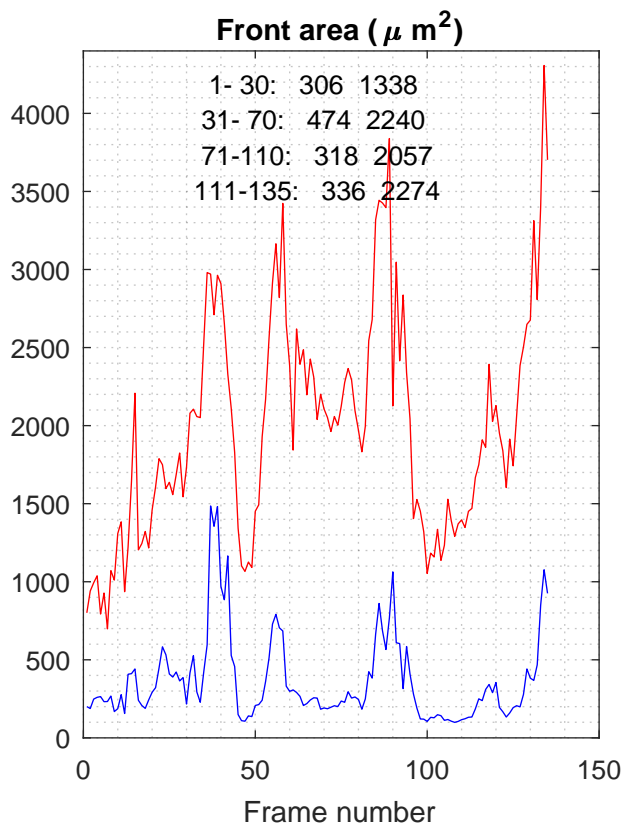

Supplement: Supplementary file 17 — Source Data for Appendix [file MSB-15-e8585-s025.zip › Source_data_for_Appendix/Appendix_Fig_S4/S4C/RasC11.pdf]

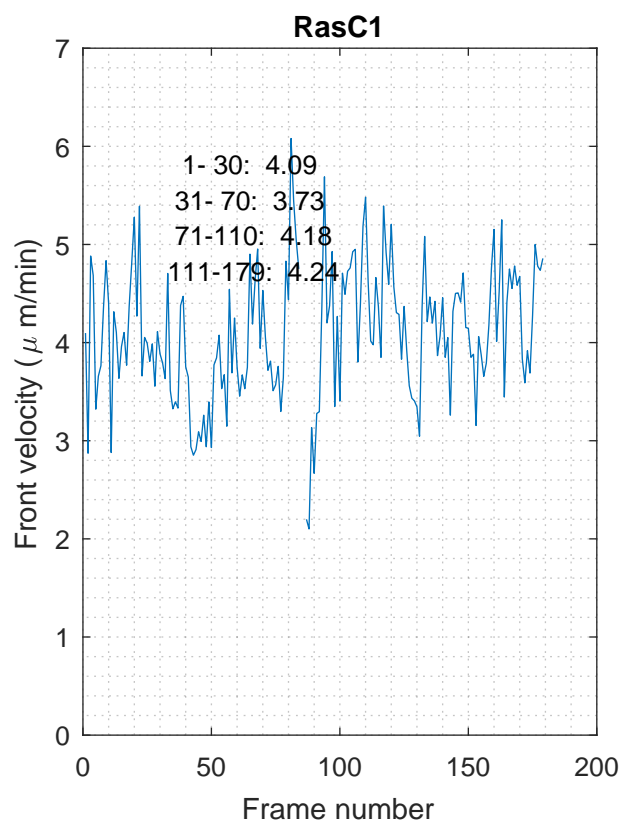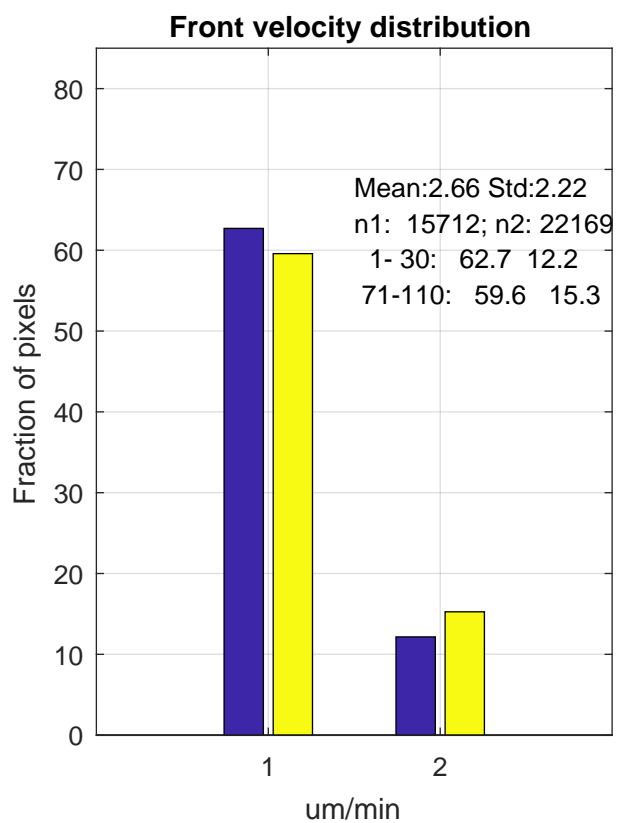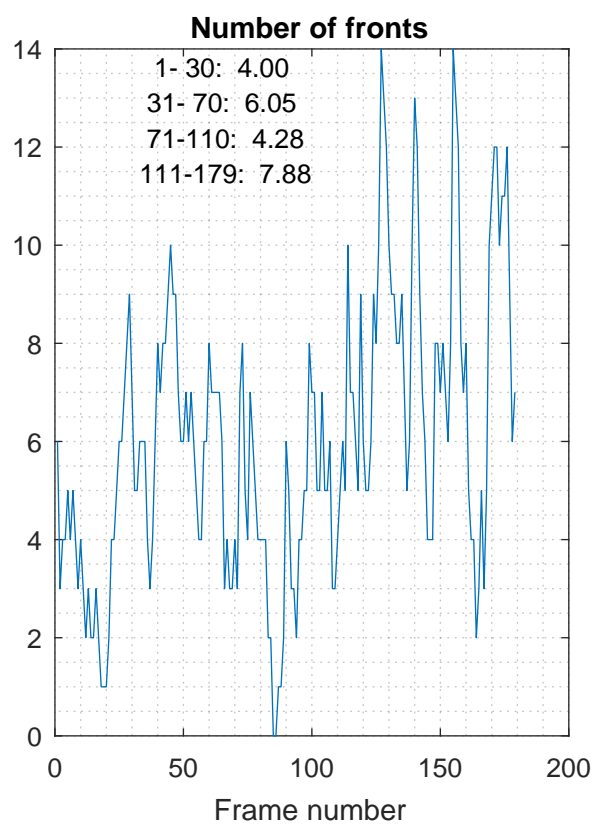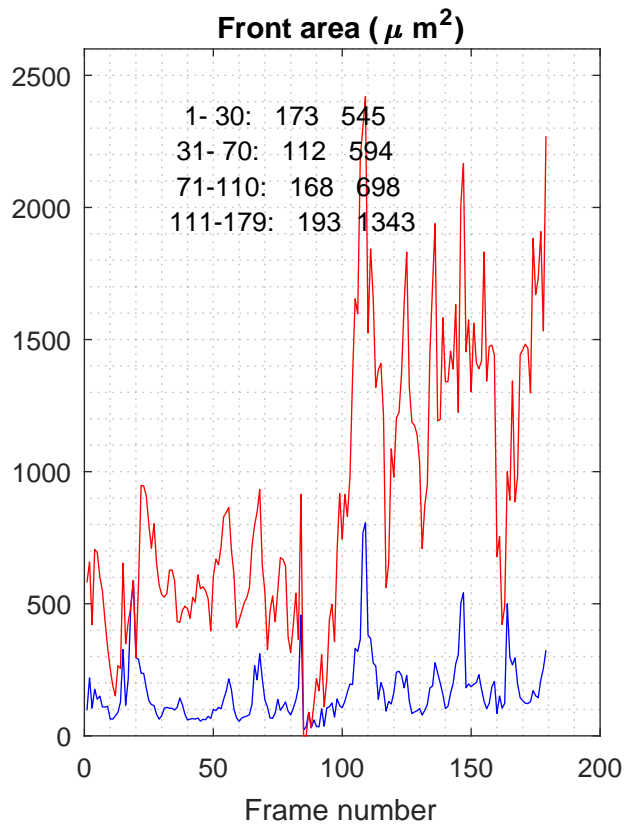

Supplement: Supplementary file 17 — Source Data for Appendix [file MSB-15-e8585-s025.zip › Source_data_for_Appendix/Appendix_Fig_S4/S4C/RasC1.pdf]

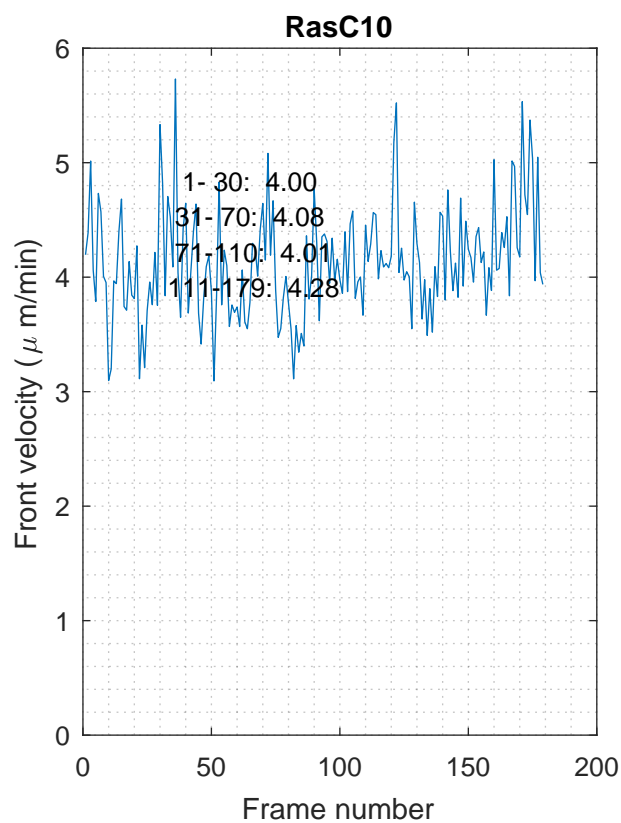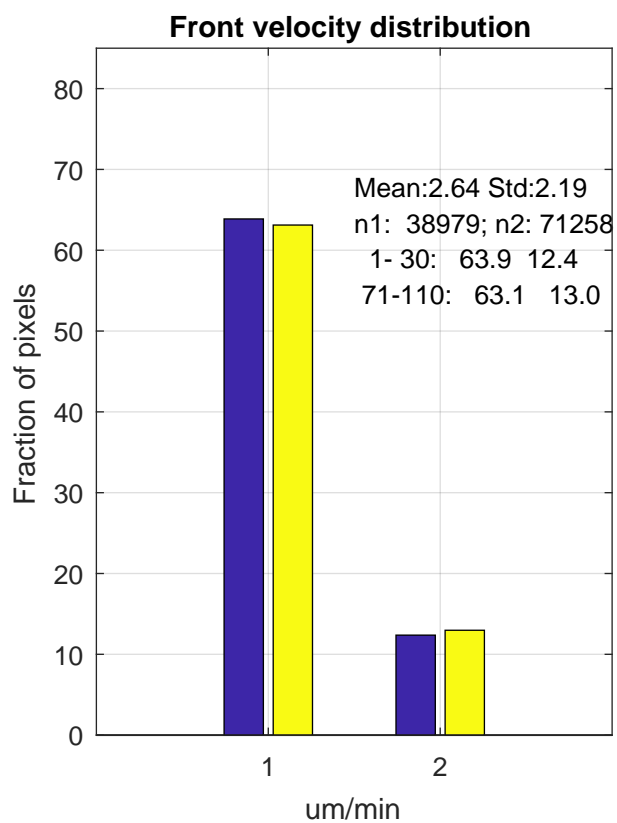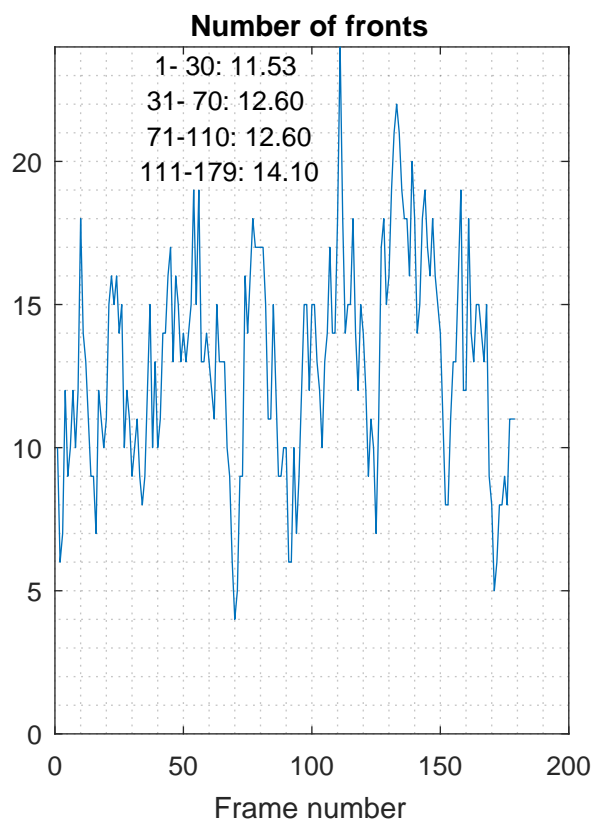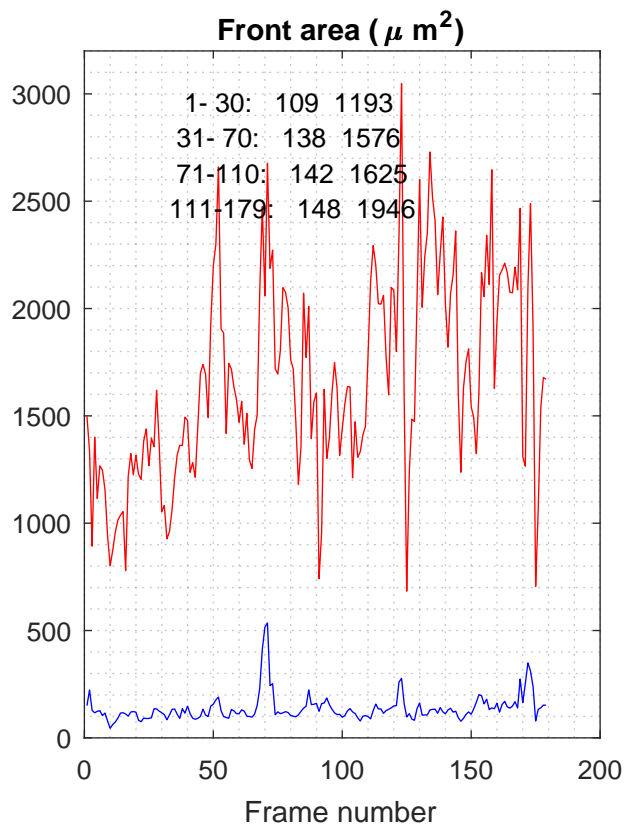

Supplement: Supplementary file 17 — Source Data for Appendix [file MSB-15-e8585-s025.zip › Source_data_for_Appendix/Appendix_Fig_S4/S4C/RasC10.pdf]

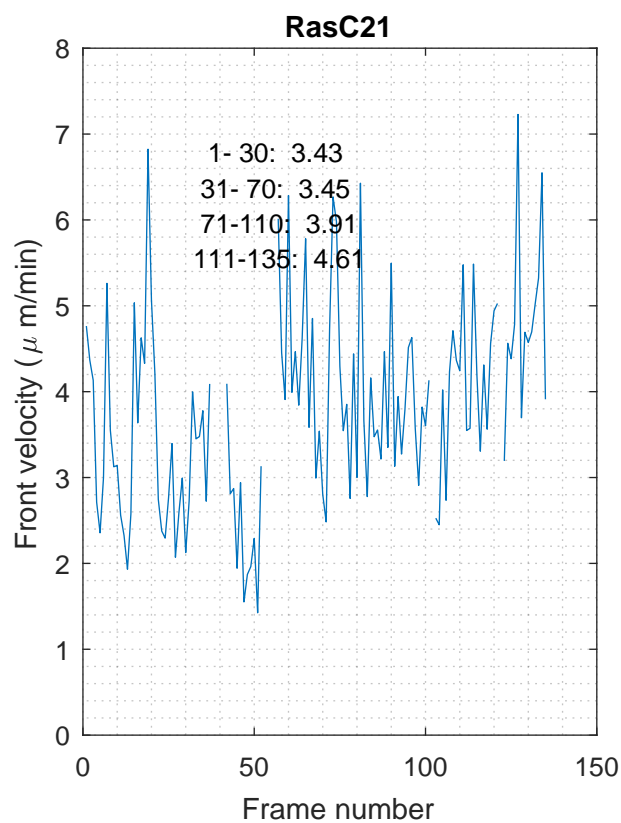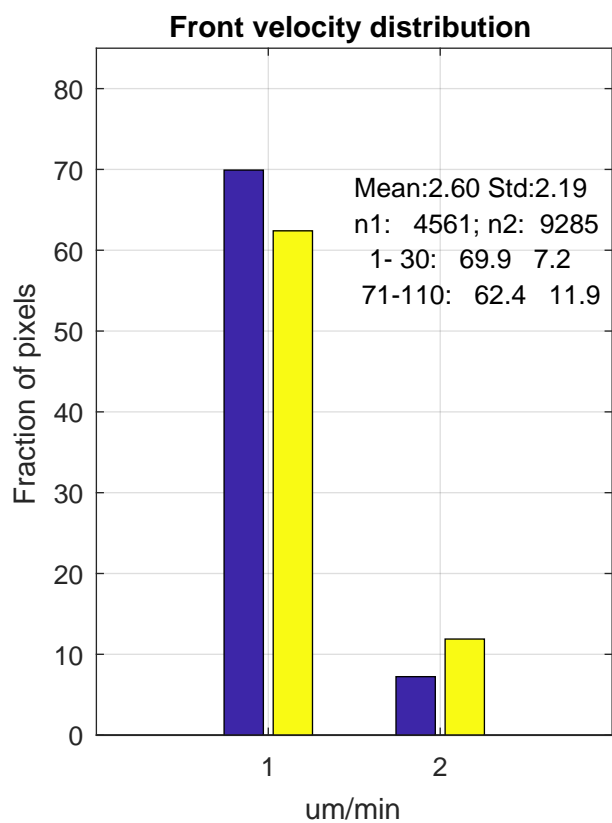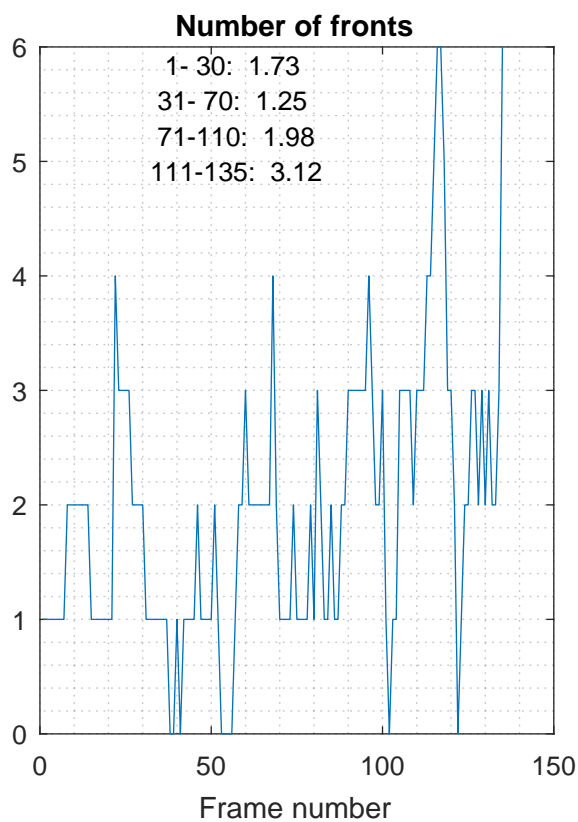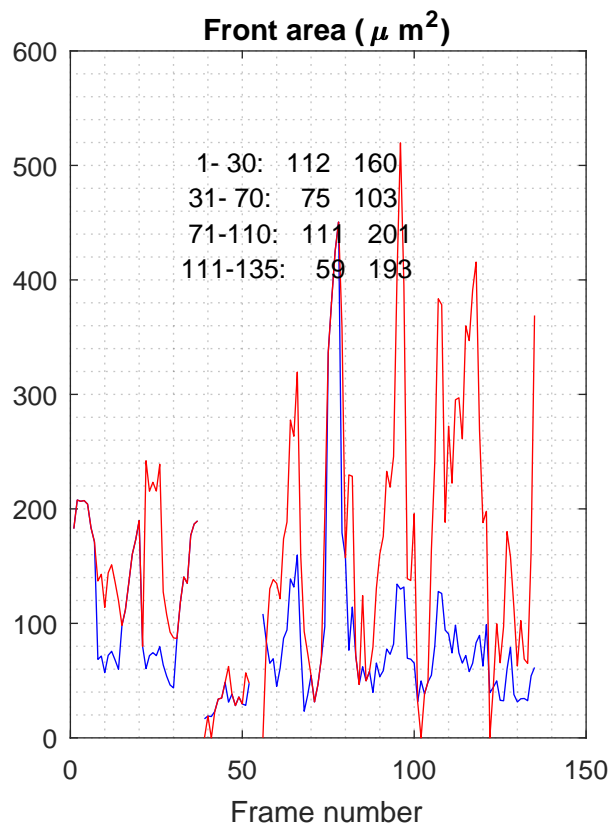

Supplement: Supplementary file 17 — Source Data for Appendix [file MSB-15-e8585-s025.zip › Source_data_for_Appendix/Appendix_Fig_S4/S4C/RasC21.pdf]

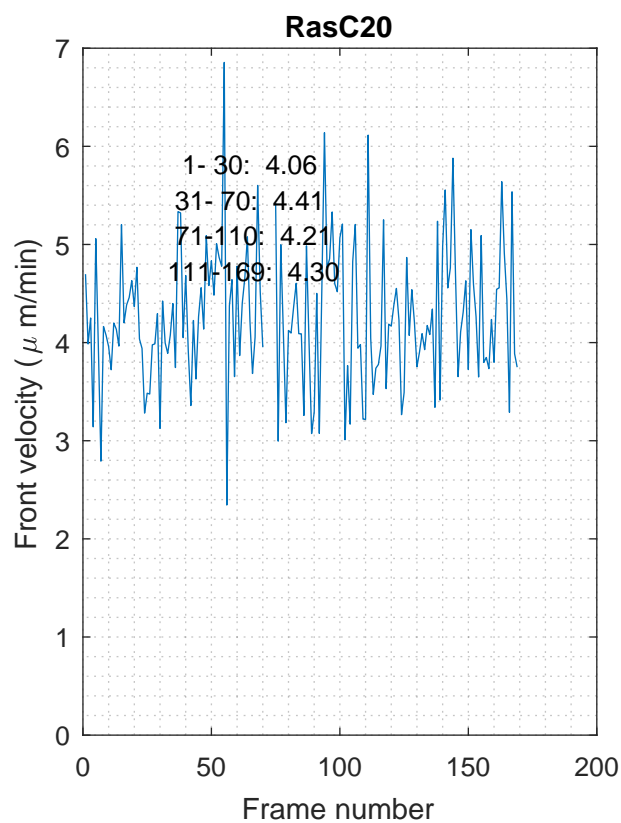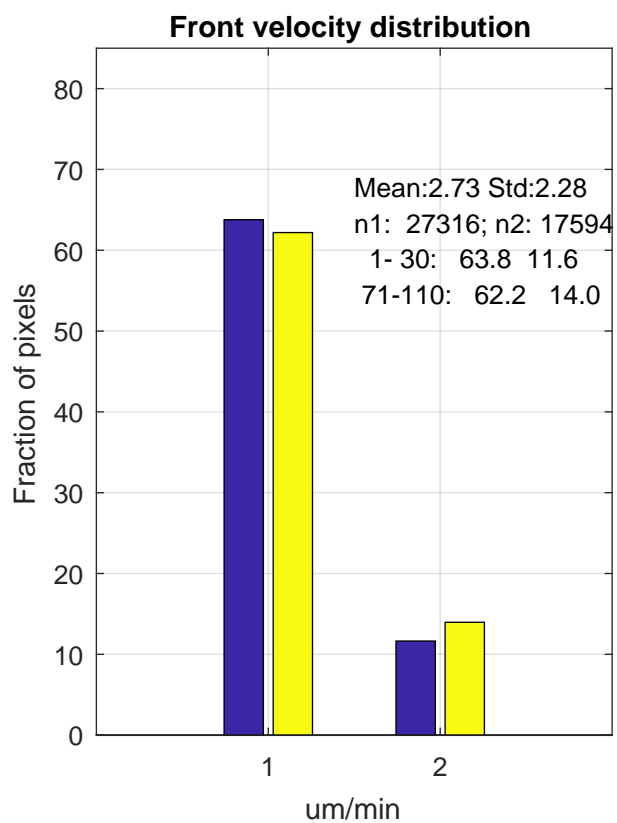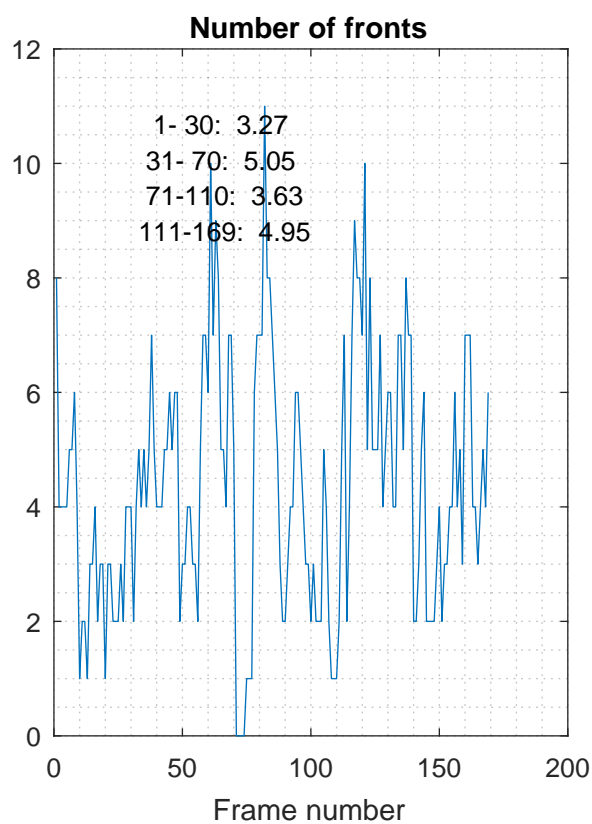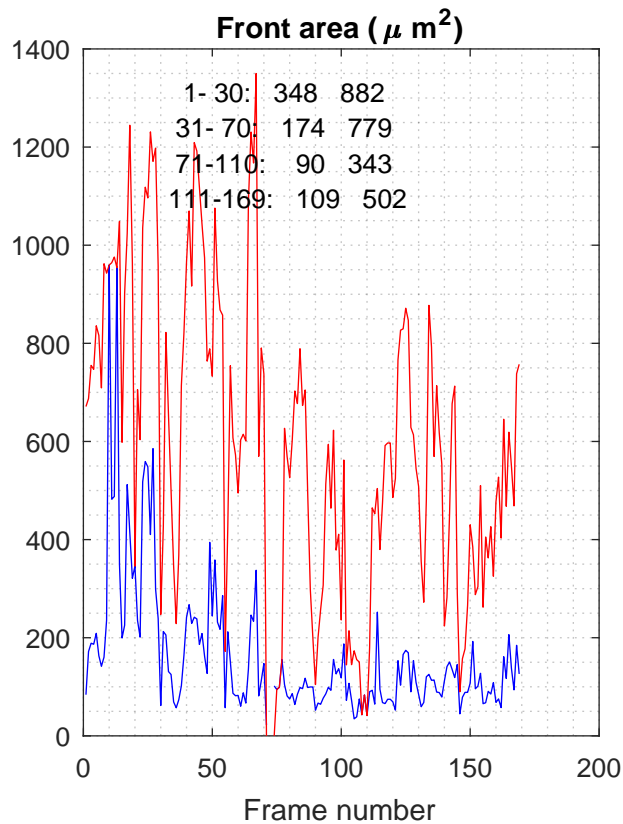

Supplement: Supplementary file 17 — Source Data for Appendix [file MSB-15-e8585-s025.zip › Source_data_for_Appendix/Appendix_Fig_S4/S4C/RasC20.pdf]

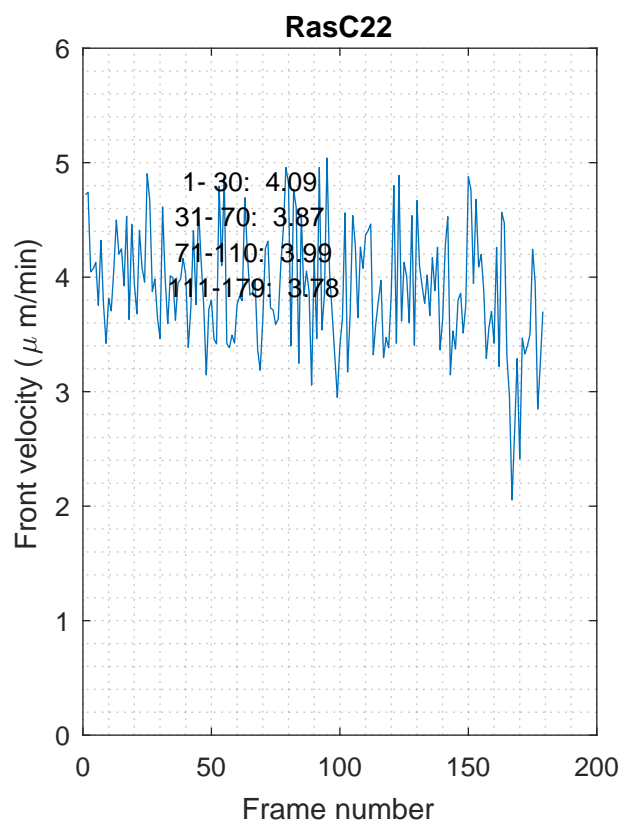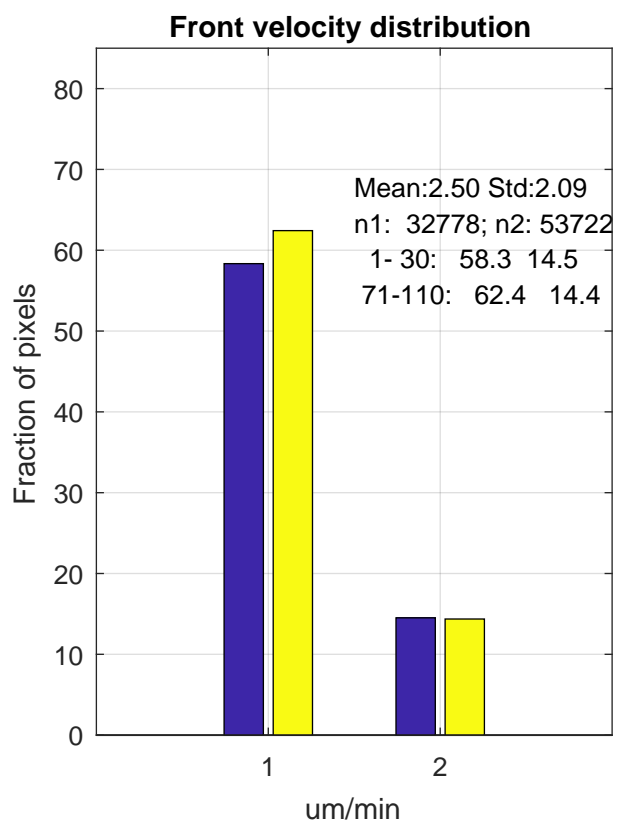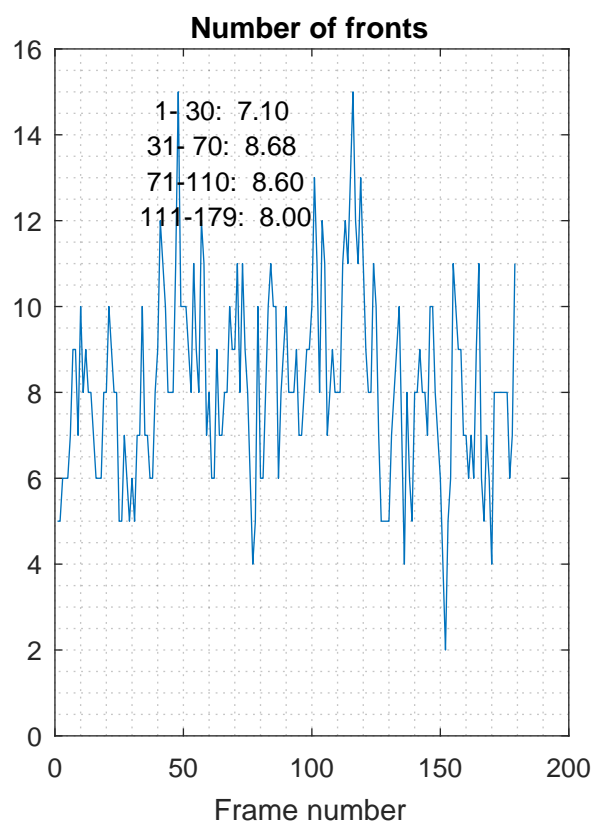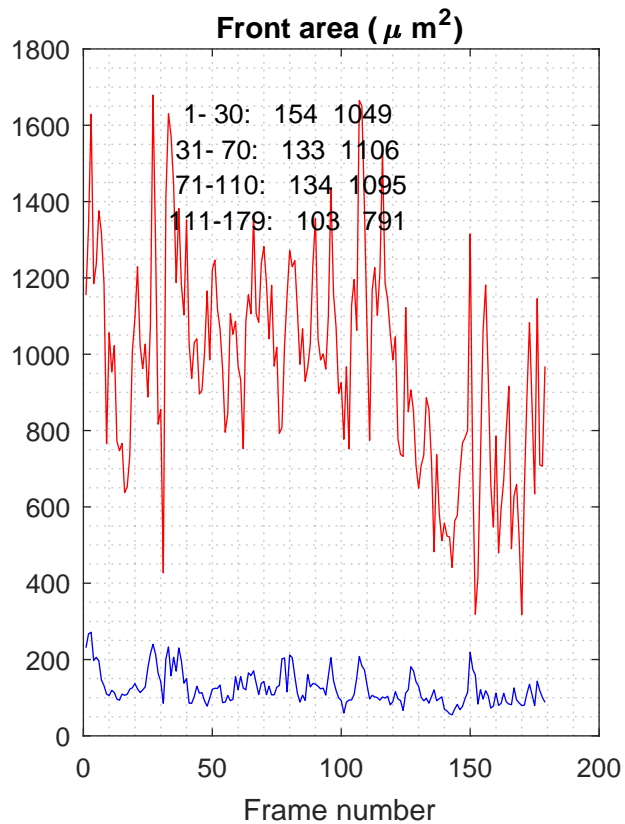

Supplement: Supplementary file 17 — Source Data for Appendix [file MSB-15-e8585-s025.zip › Source_data_for_Appendix/Appendix_Fig_S4/S4C/RasC22.pdf]

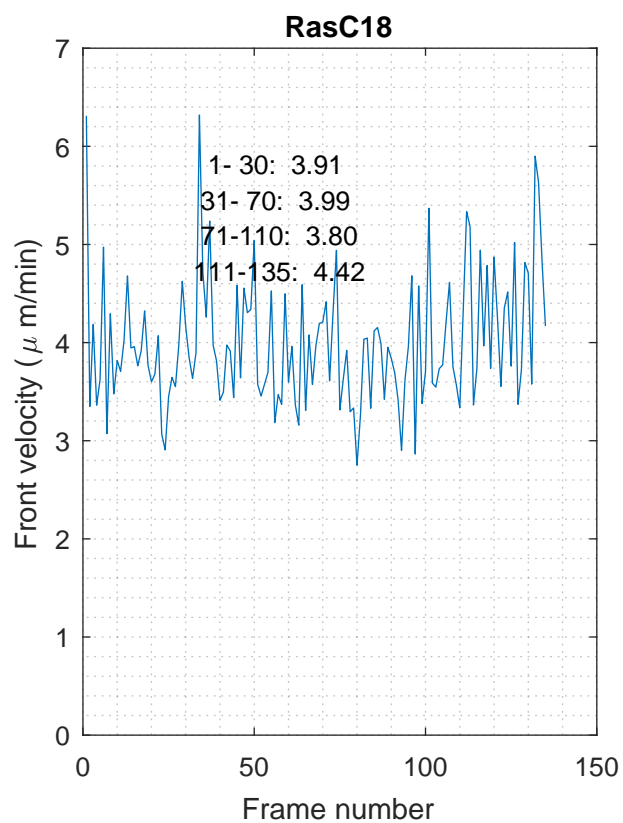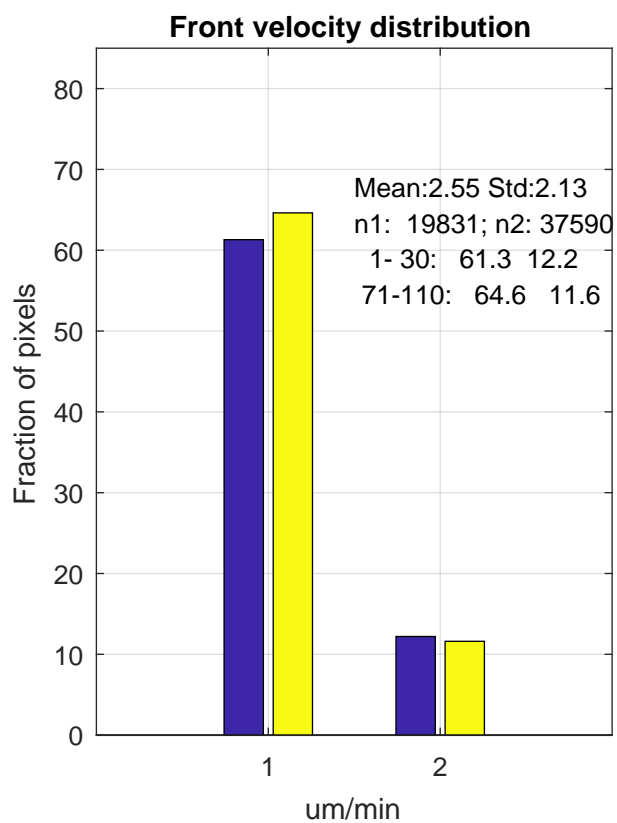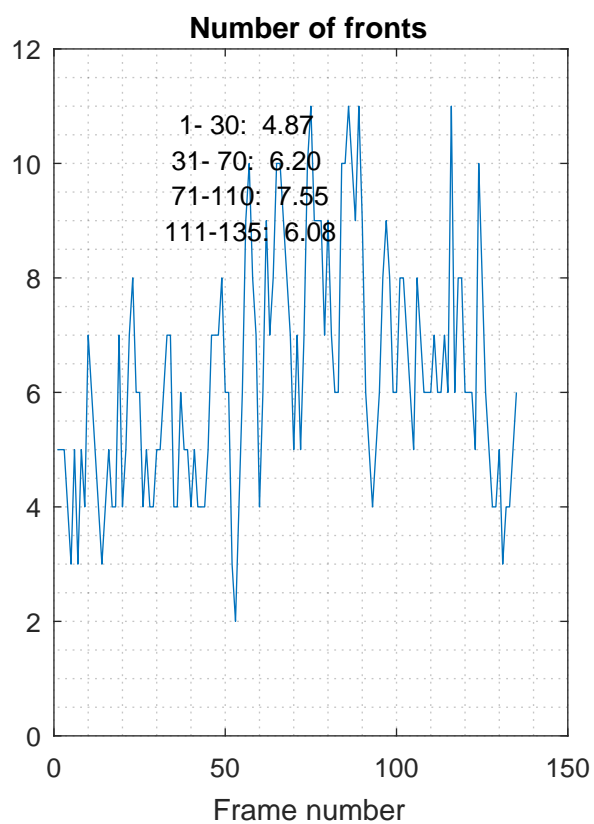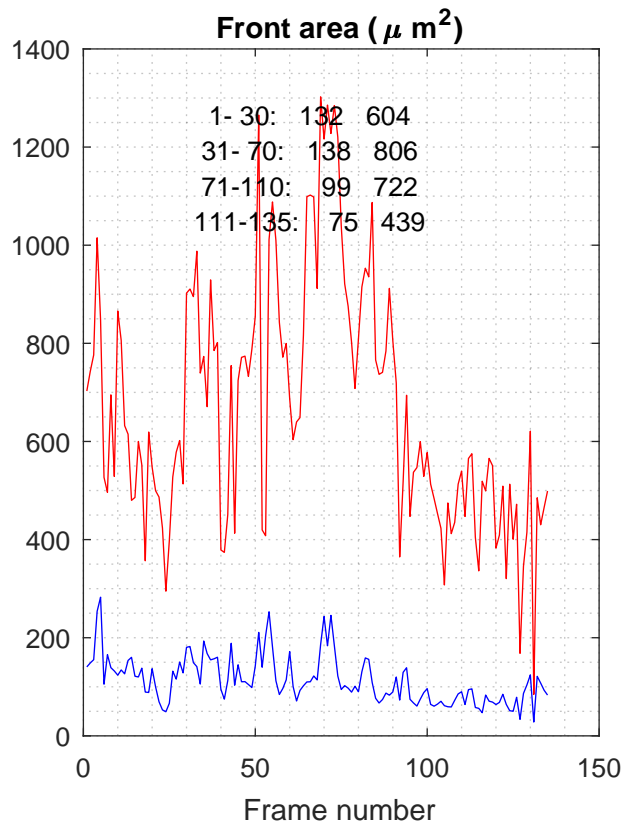

Supplement: Supplementary file 17 — Source Data for Appendix [file MSB-15-e8585-s025.zip › Source_data_for_Appendix/Appendix_Fig_S4/S4C/RasC18.pdf]

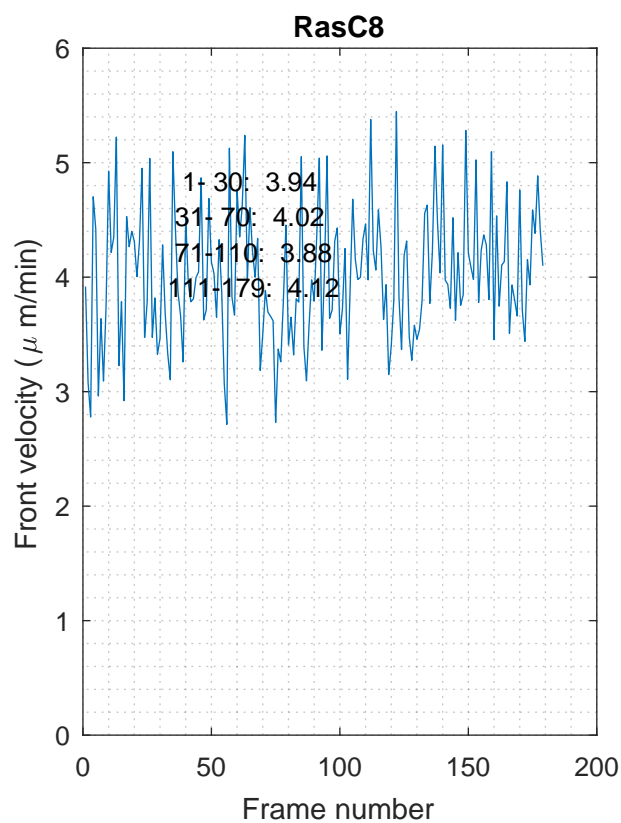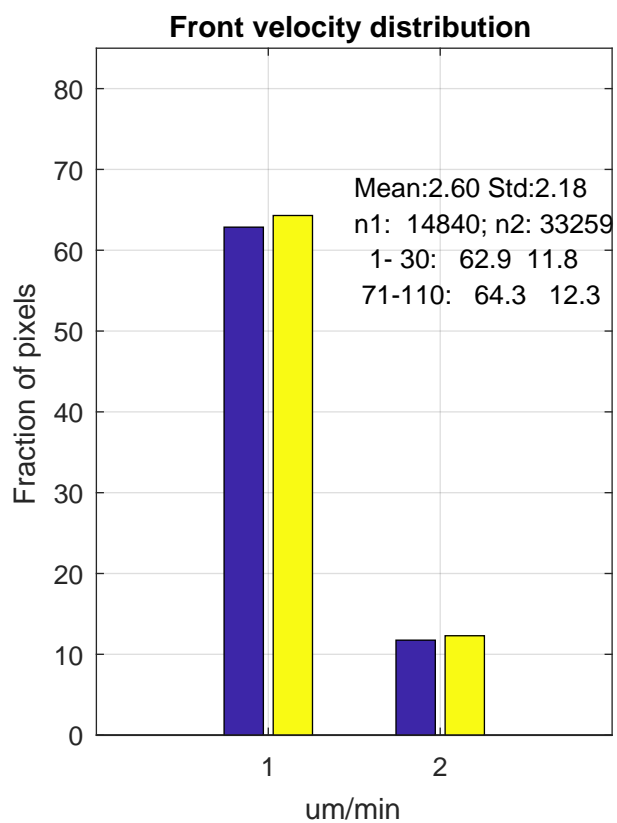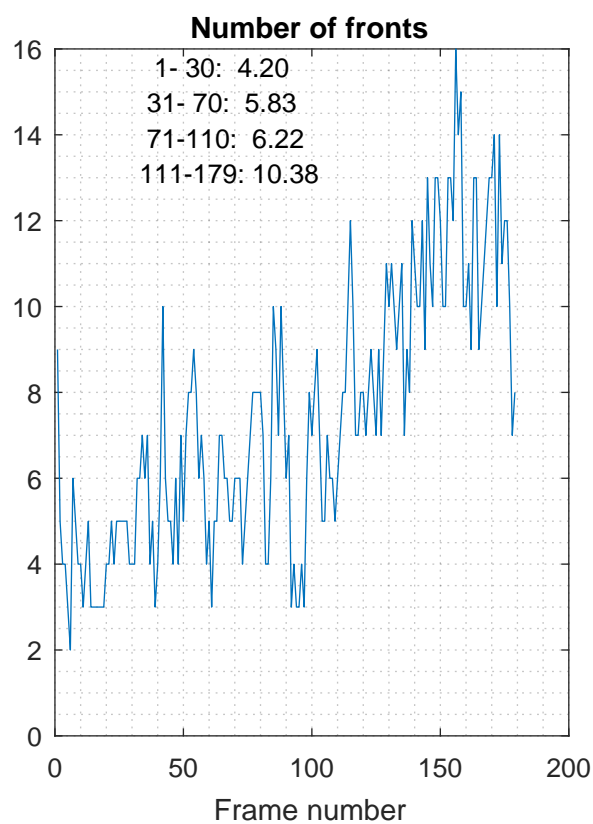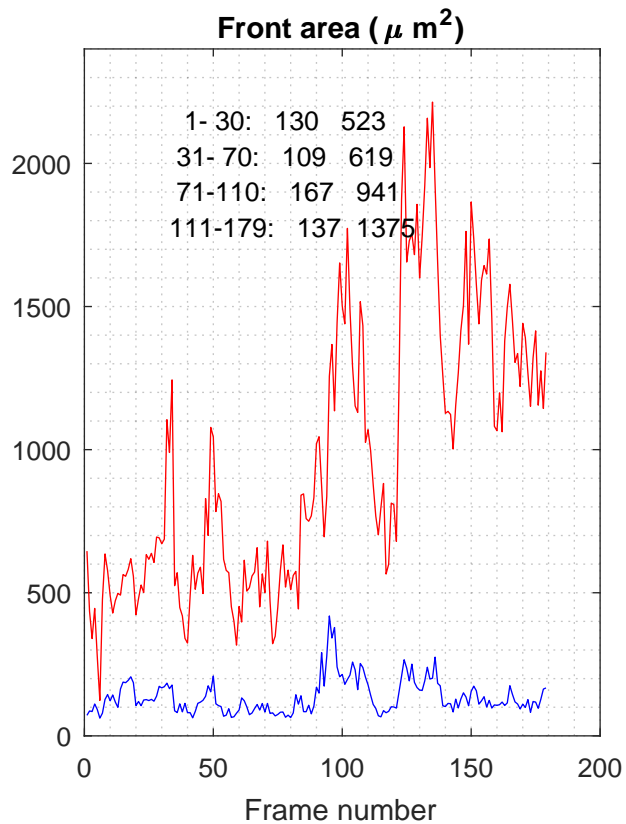

Supplement: Supplementary file 17 — Source Data for Appendix [file MSB-15-e8585-s025.zip › Source_data_for_Appendix/Appendix_Fig_S4/S4C/RasC8.pdf]

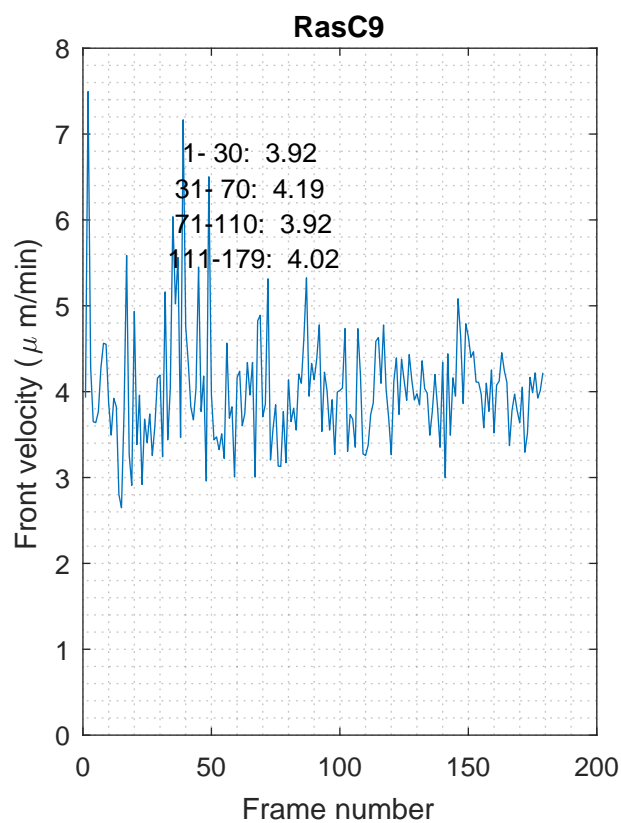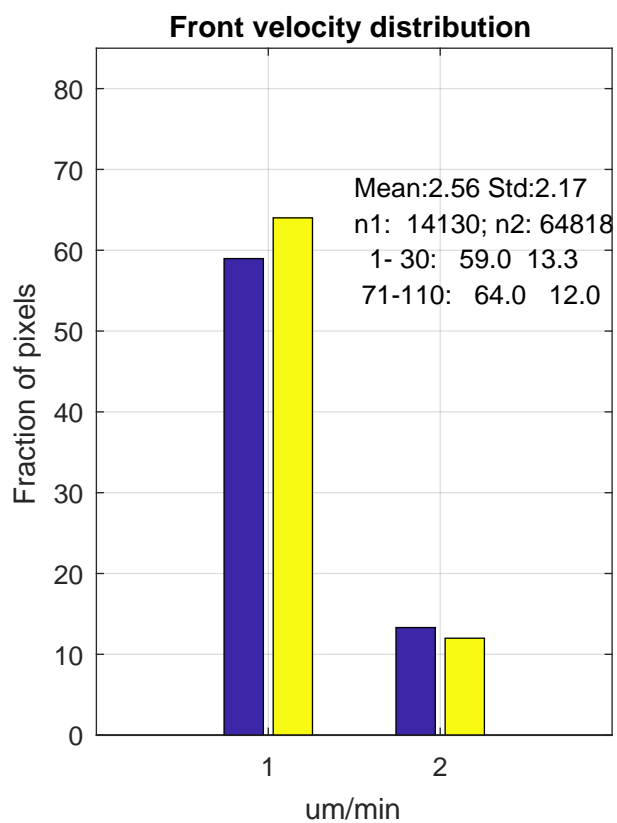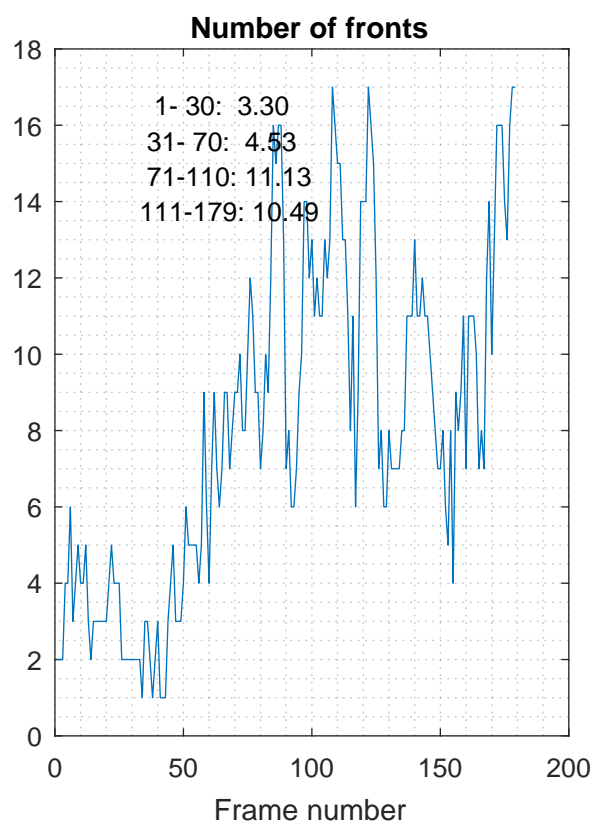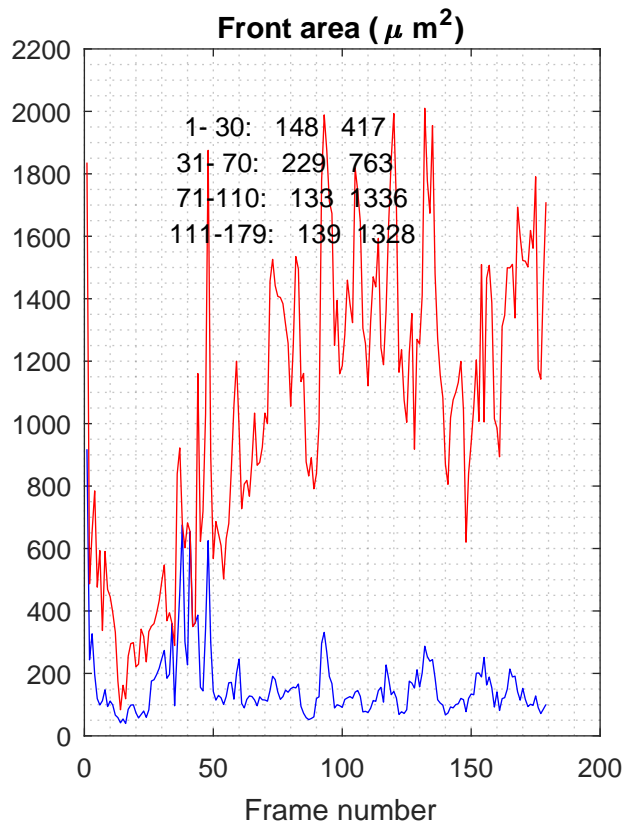

Supplement: Supplementary file 17 — Source Data for Appendix [file MSB-15-e8585-s025.zip › Source_data_for_Appendix/Appendix_Fig_S4/S4C/RasC9.pdf]

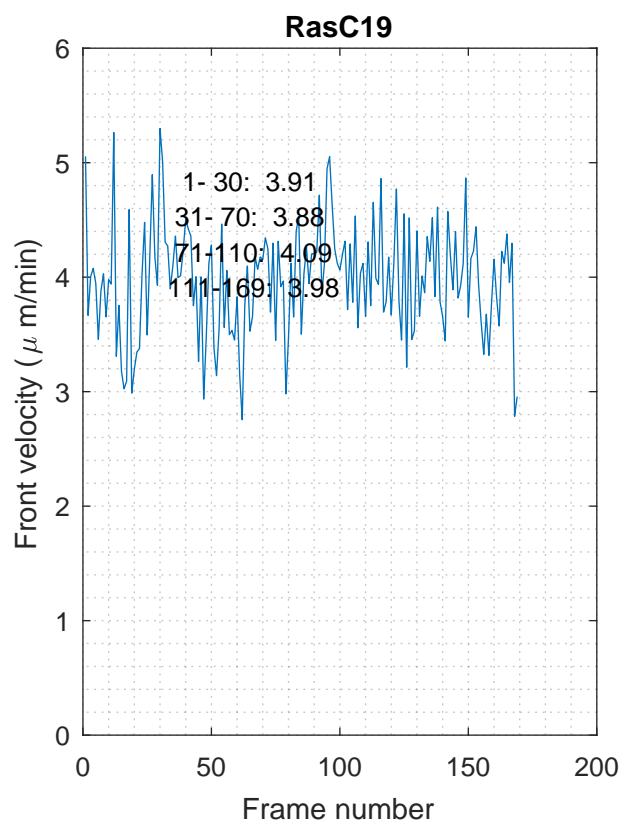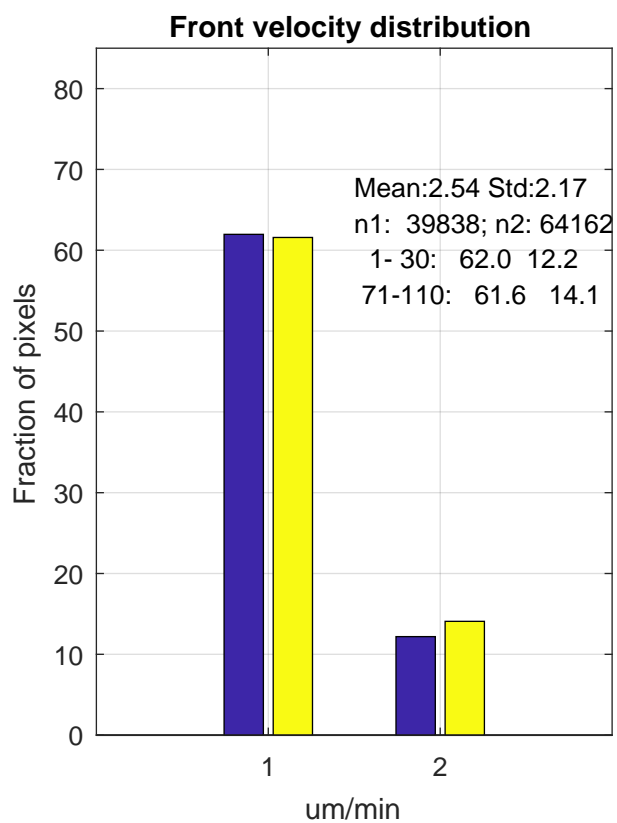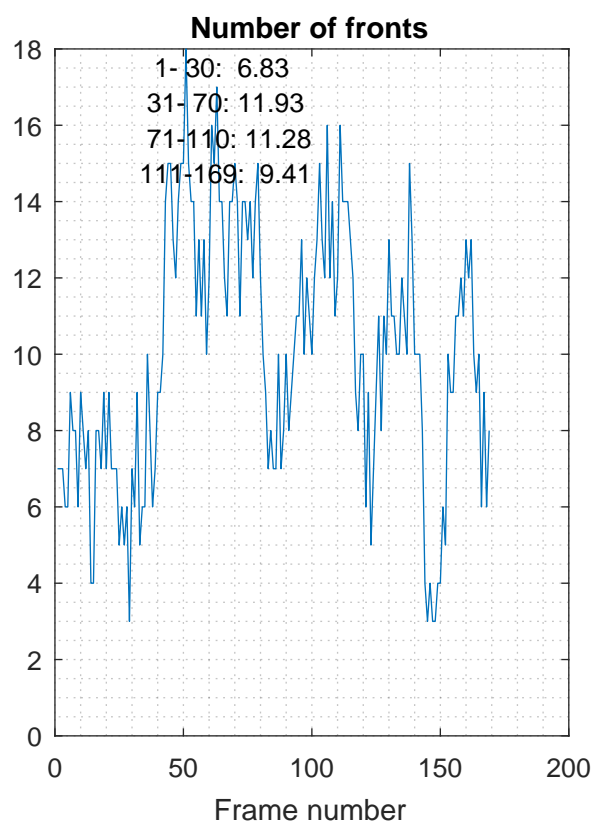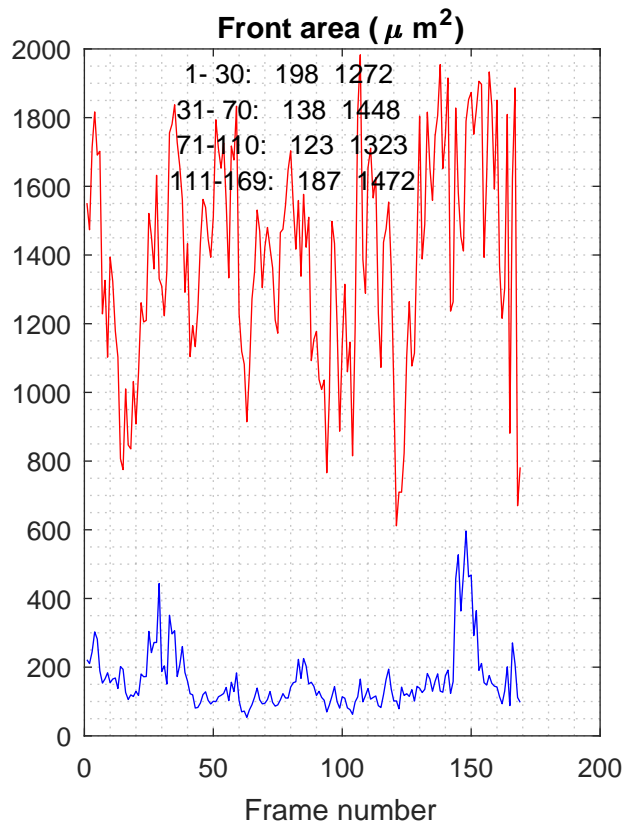

Supplement: Supplementary file 17 — Source Data for Appendix [file MSB-15-e8585-s025.zip › Source_data_for_Appendix/Appendix_Fig_S4/S4C/RasC19.pdf]

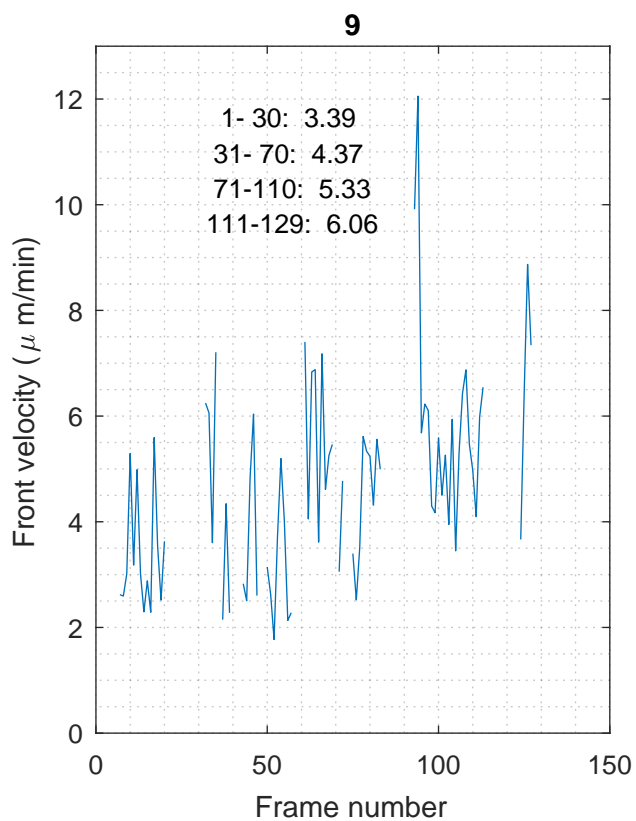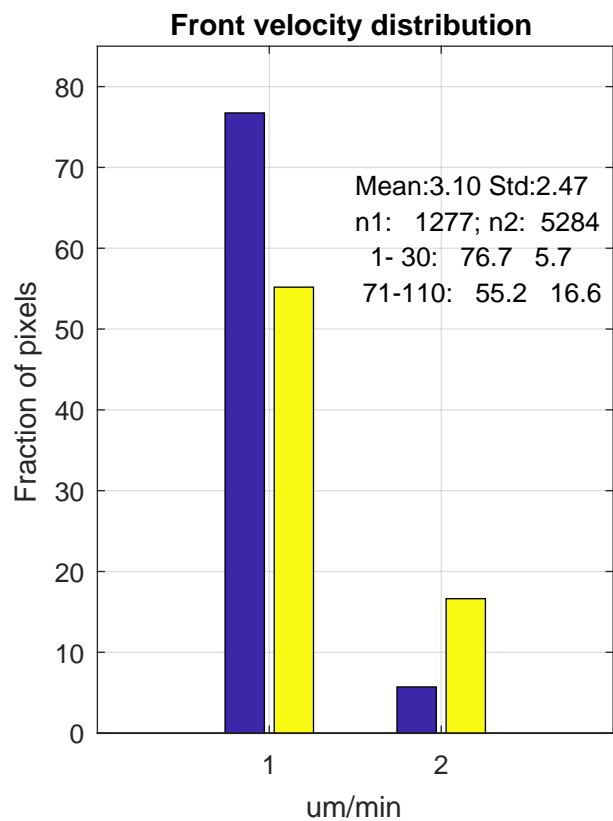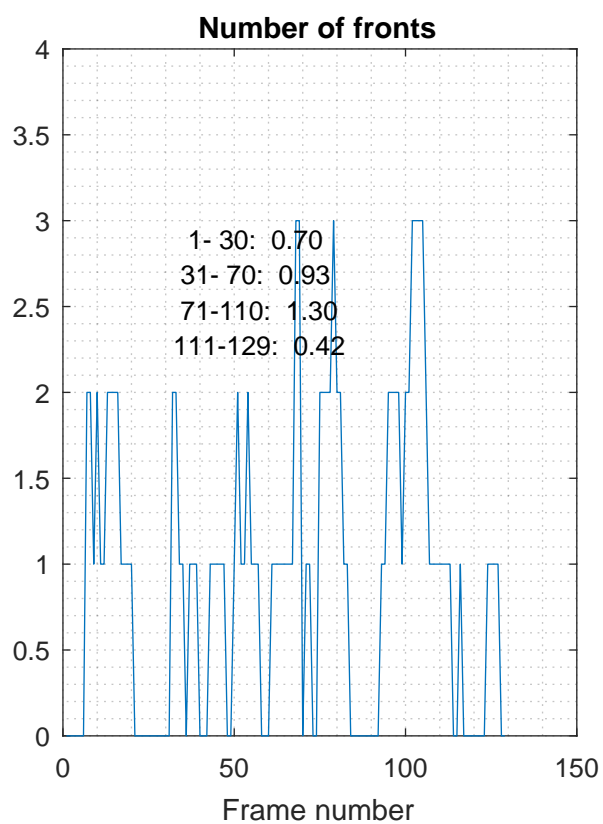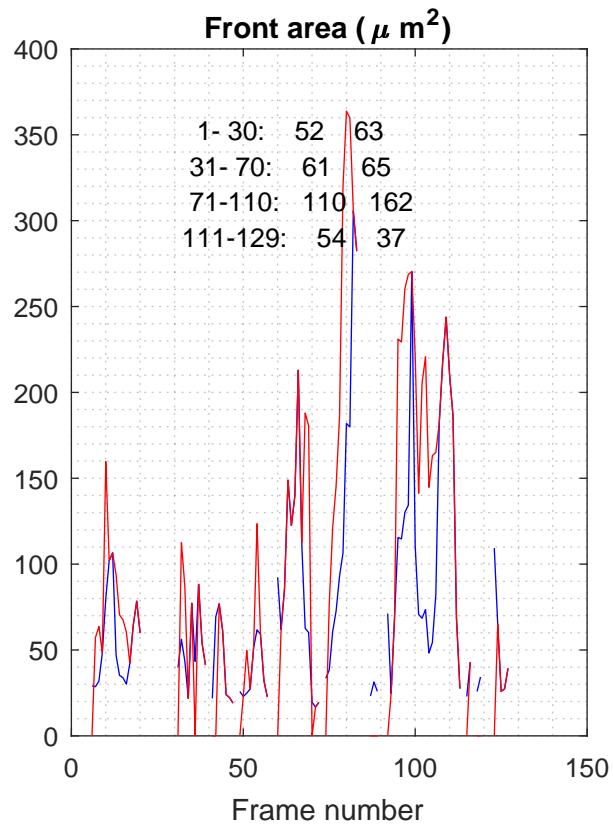

Supplement: Supplementary file 17 — Source Data for Appendix [file MSB-15-e8585-s025.zip › Source_data_for_Appendix/Appendix_Fig_S4/S4D/9.pdf]

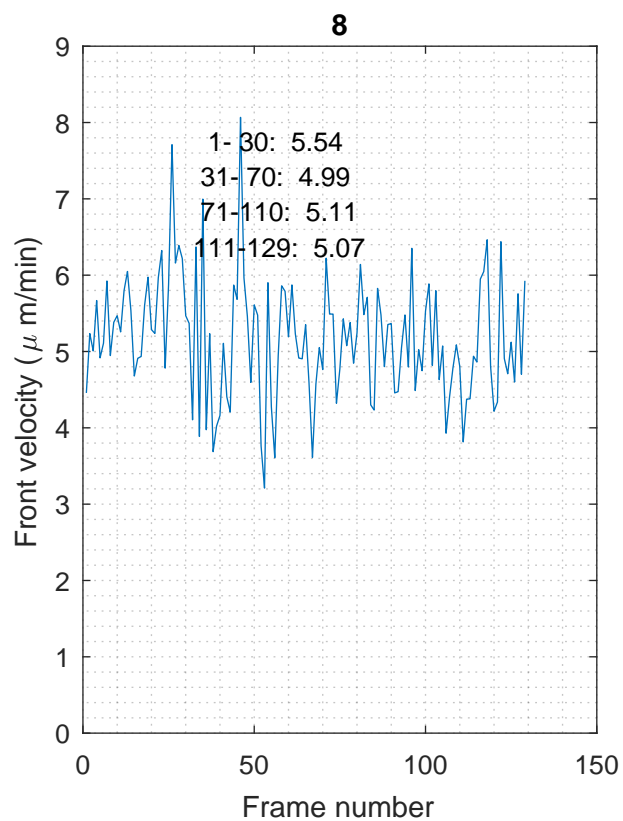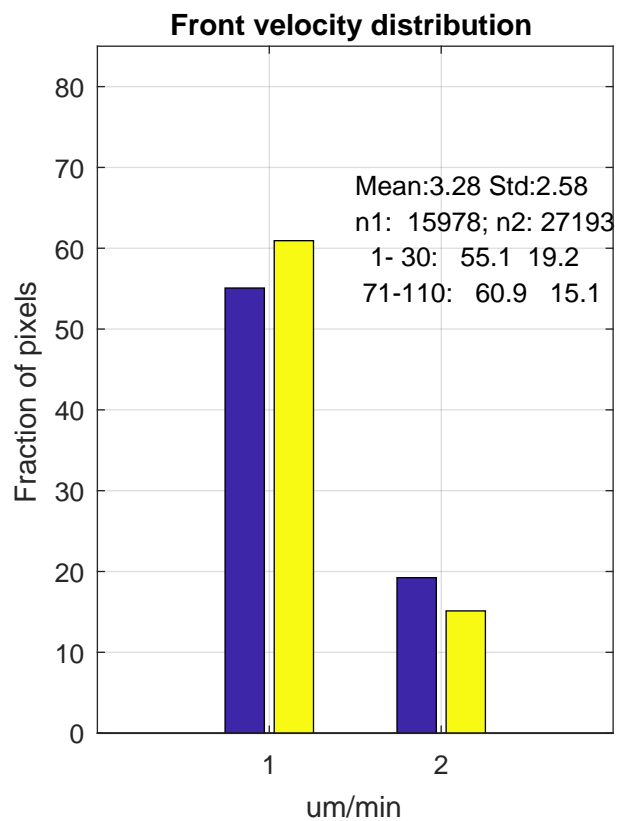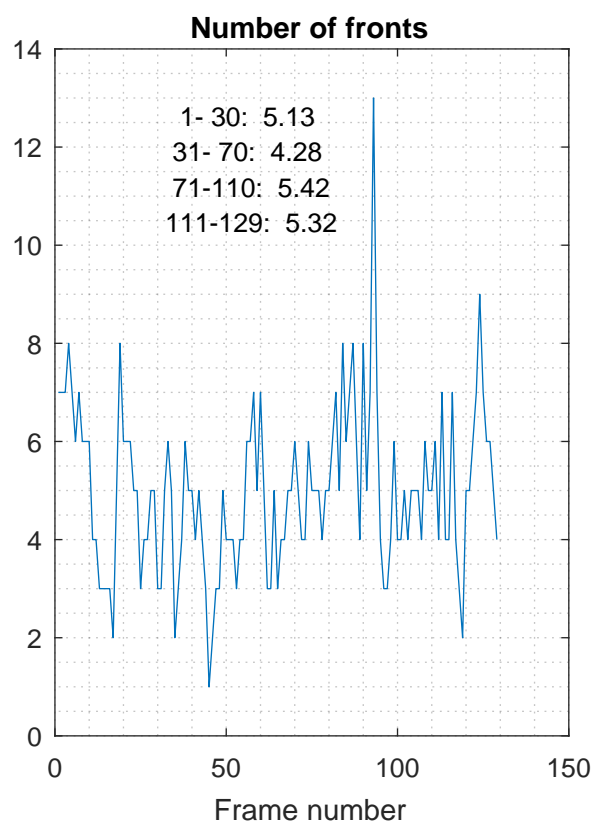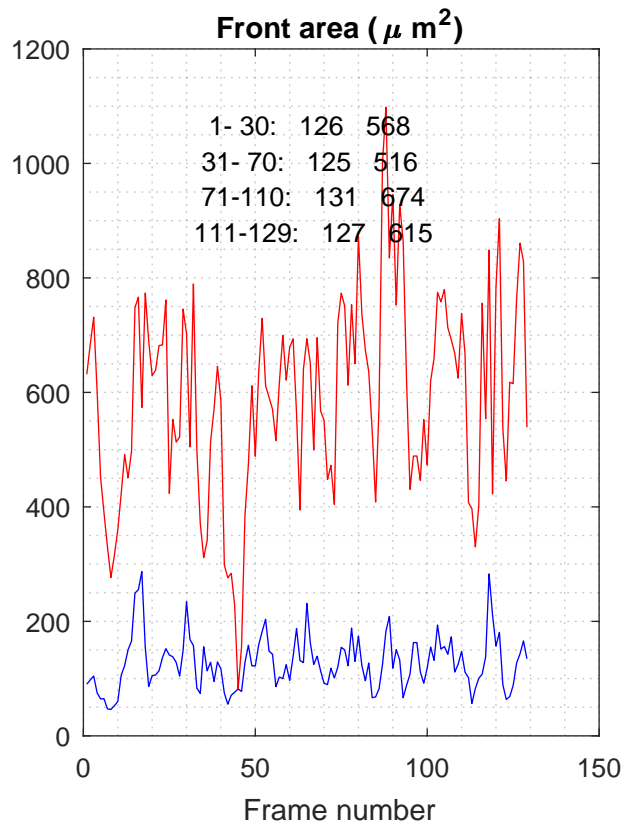

Supplement: Supplementary file 17 — Source Data for Appendix [file MSB-15-e8585-s025.zip › Source_data_for_Appendix/Appendix_Fig_S4/S4D/8.pdf]

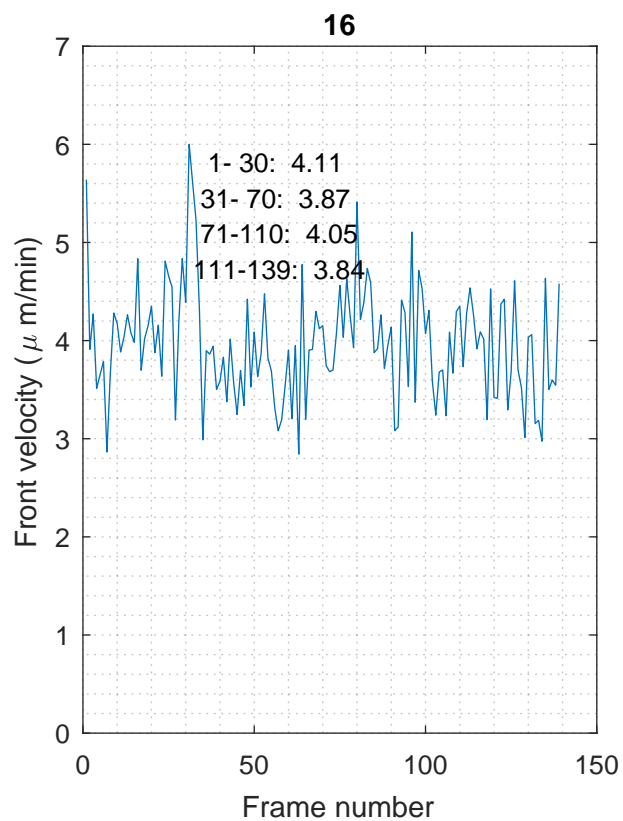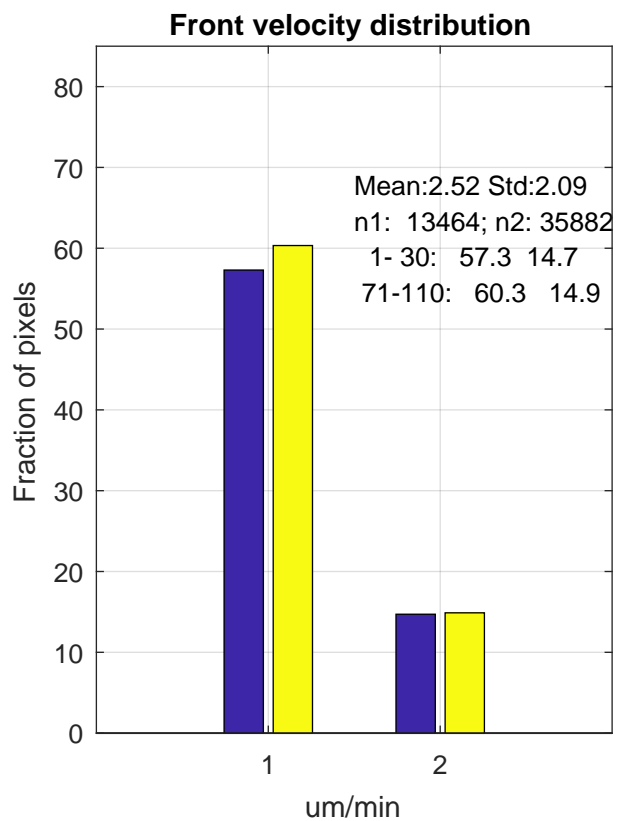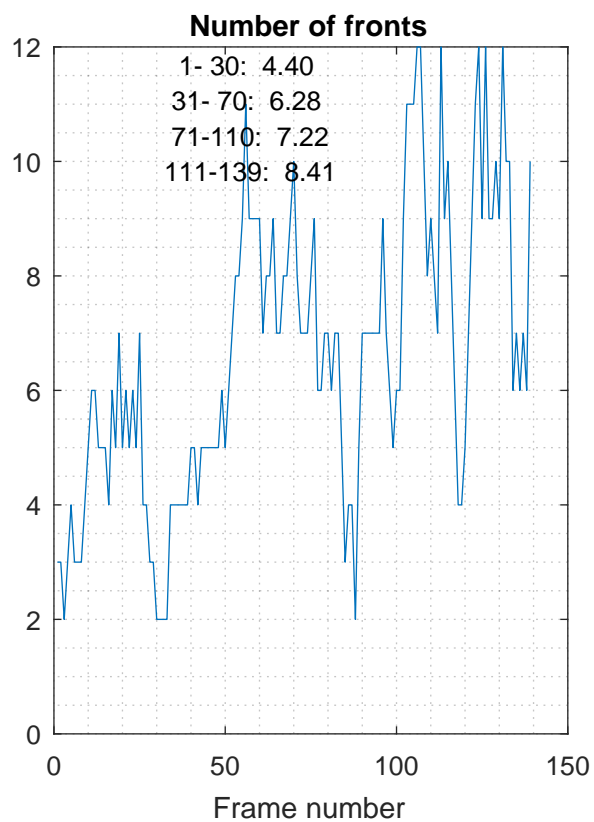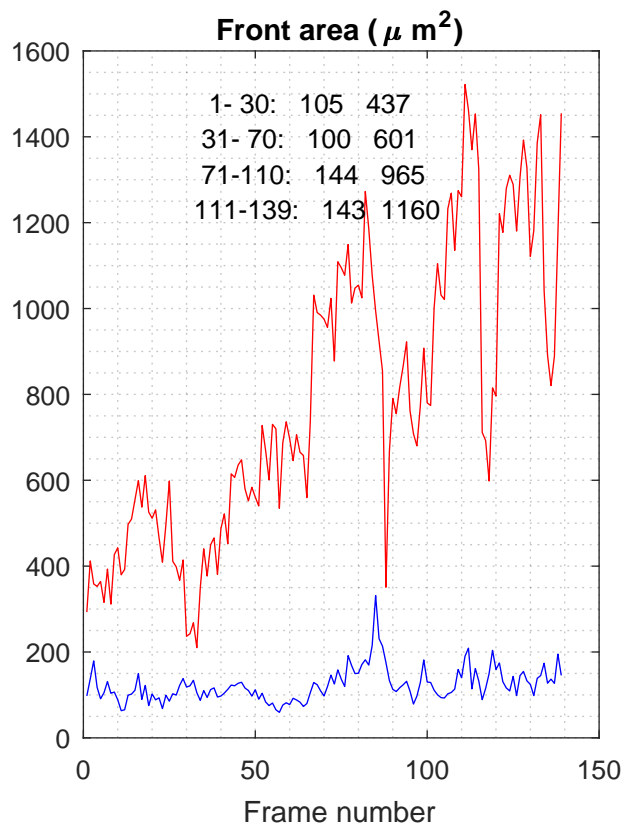

Supplement: Supplementary file 17 — Source Data for Appendix [file MSB-15-e8585-s025.zip › Source_data_for_Appendix/Appendix_Fig_S4/S4D/16.pdf]

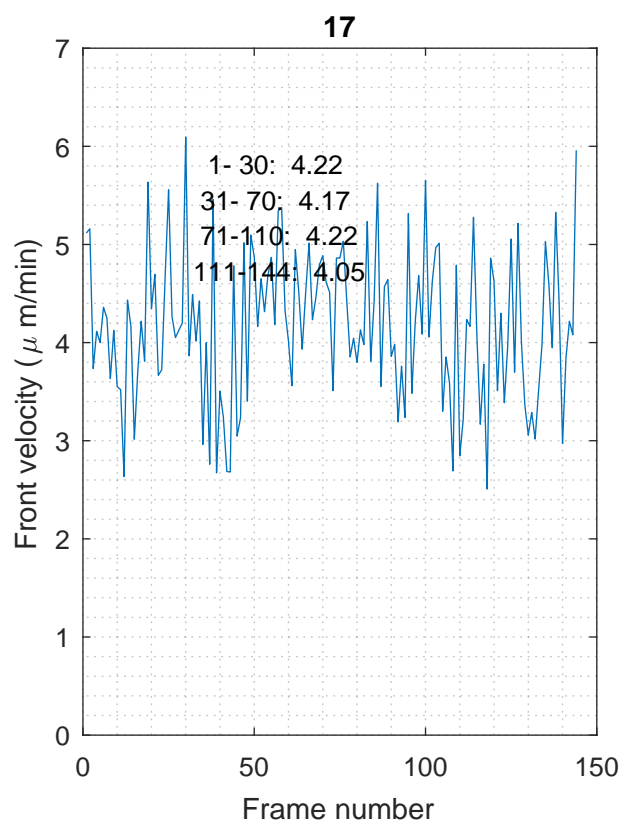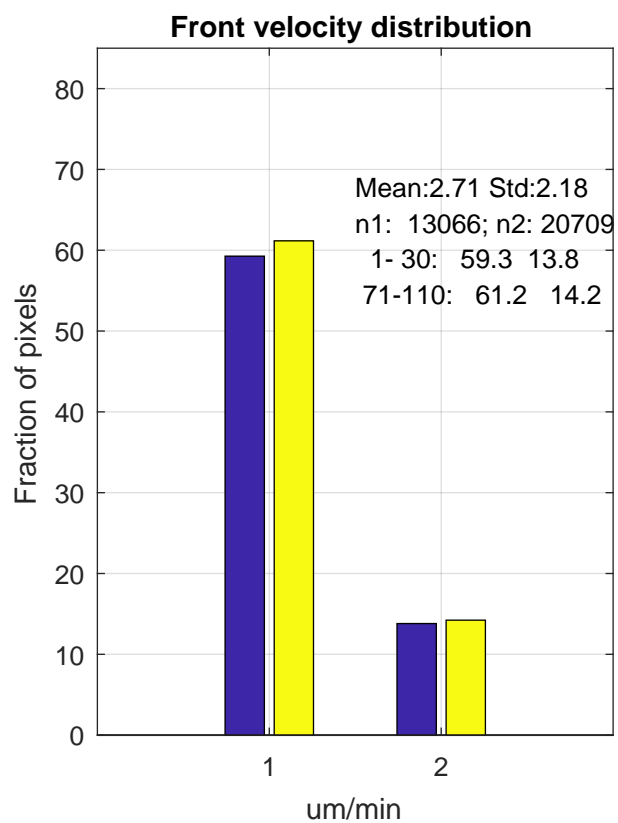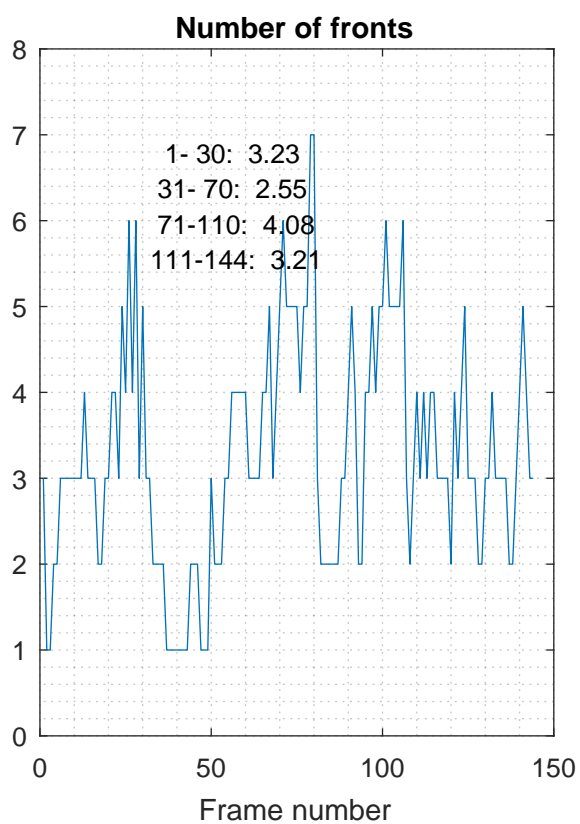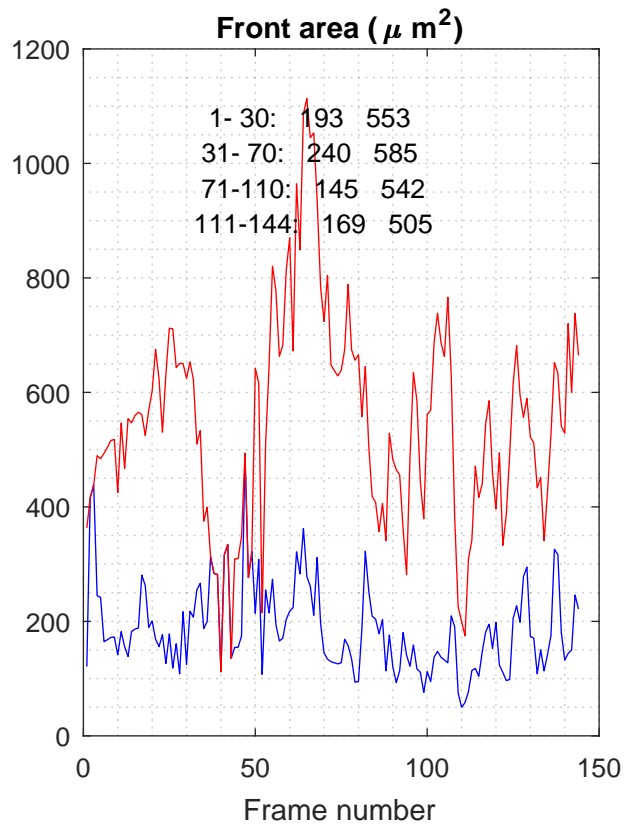

Supplement: Supplementary file 17 — Source Data for Appendix [file MSB-15-e8585-s025.zip › Source_data_for_Appendix/Appendix_Fig_S4/S4D/17.pdf]

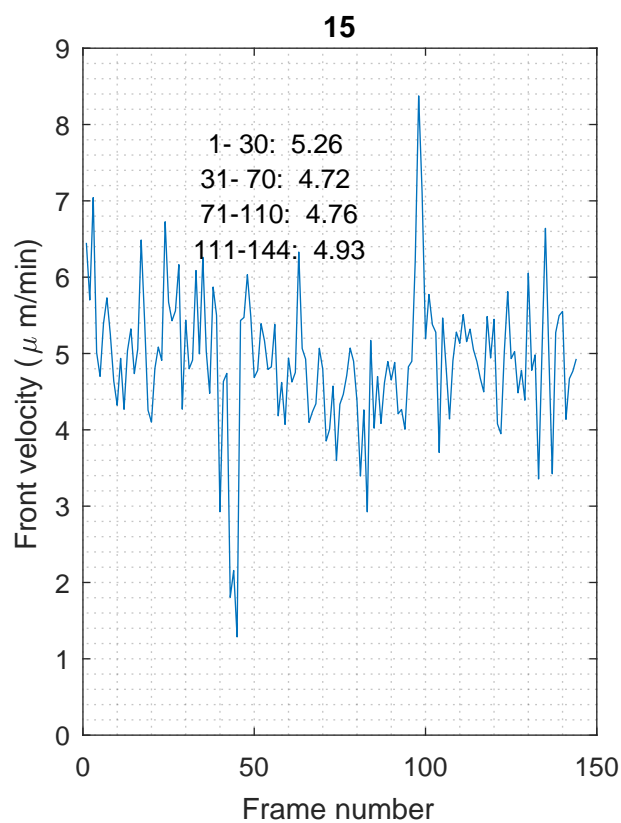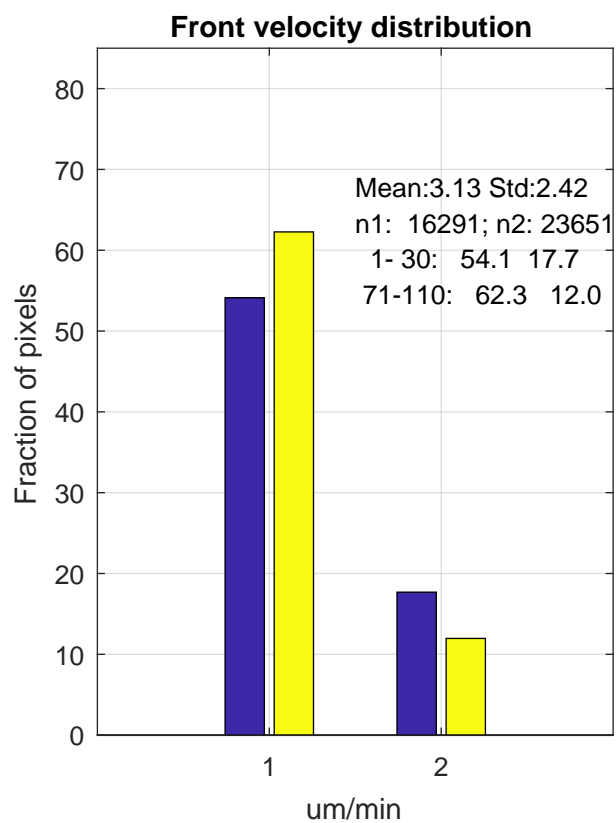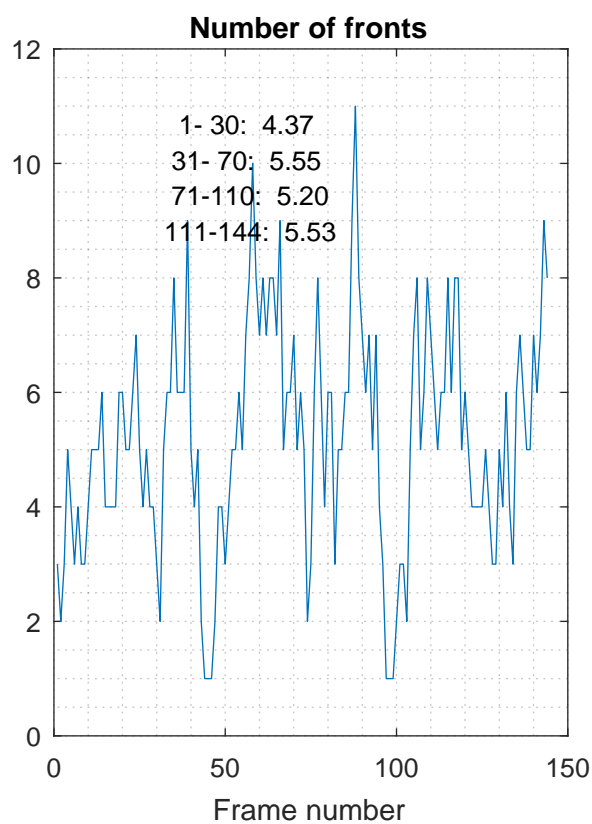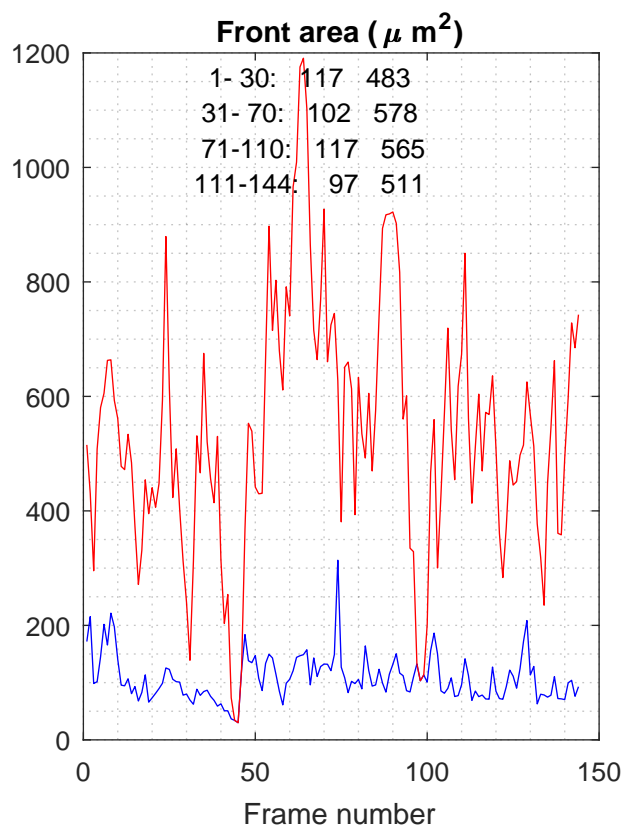

Supplement: Supplementary file 17 — Source Data for Appendix [file MSB-15-e8585-s025.zip › Source_data_for_Appendix/Appendix_Fig_S4/S4D/15.pdf]

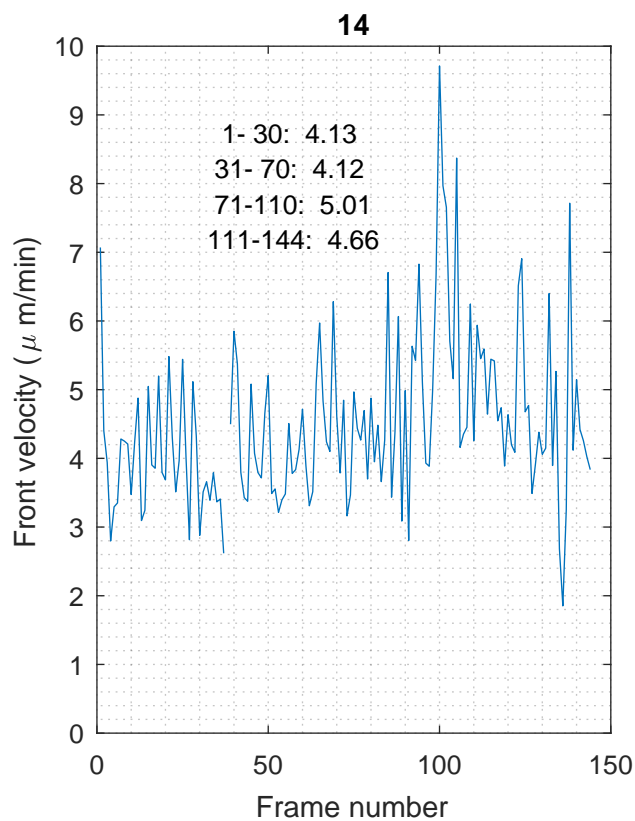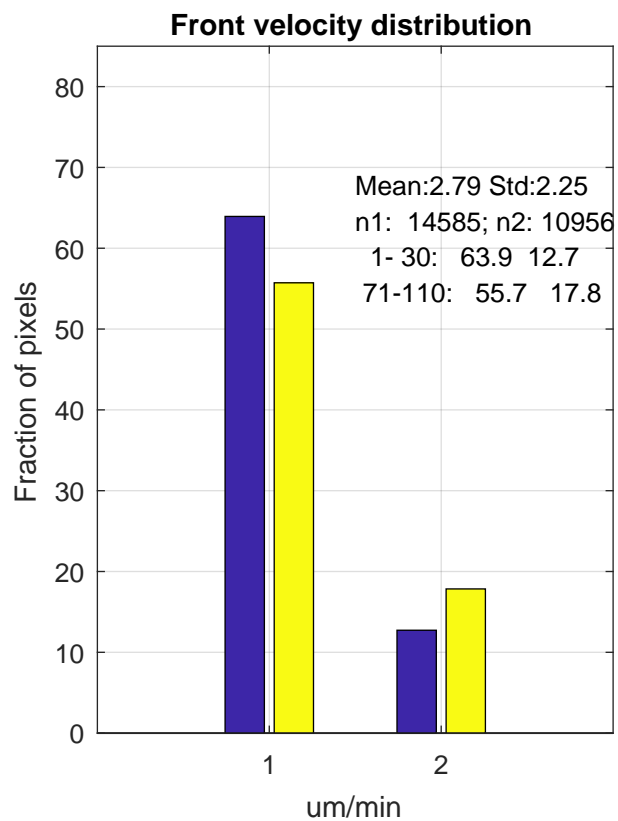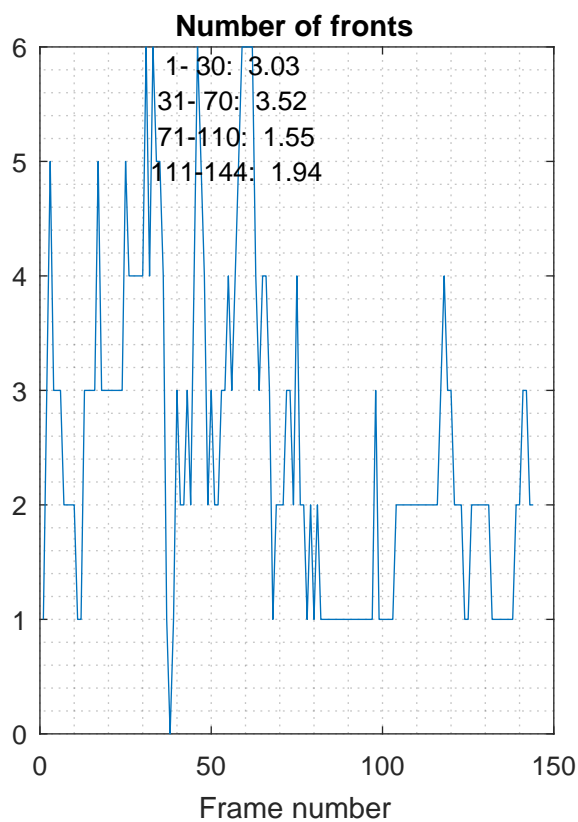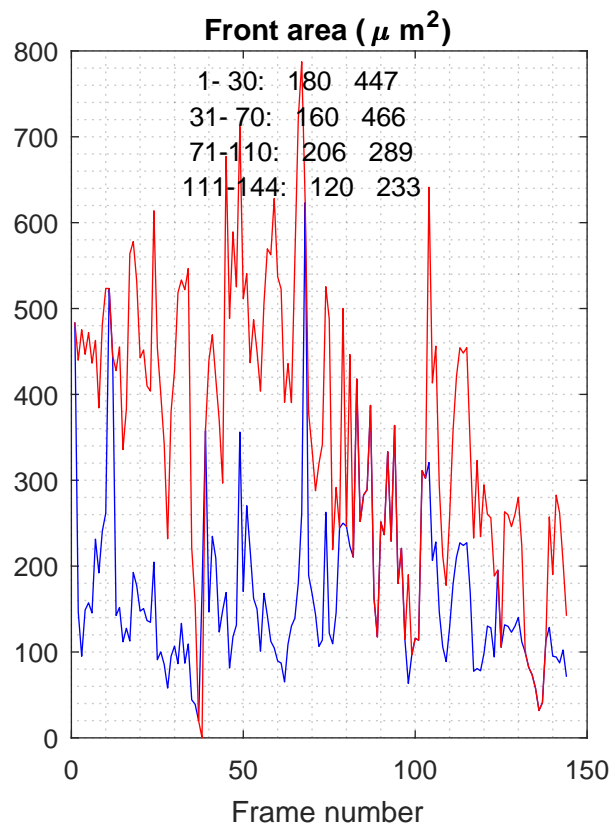

Supplement: Supplementary file 17 — Source Data for Appendix [file MSB-15-e8585-s025.zip › Source_data_for_Appendix/Appendix_Fig_S4/S4D/14.pdf]

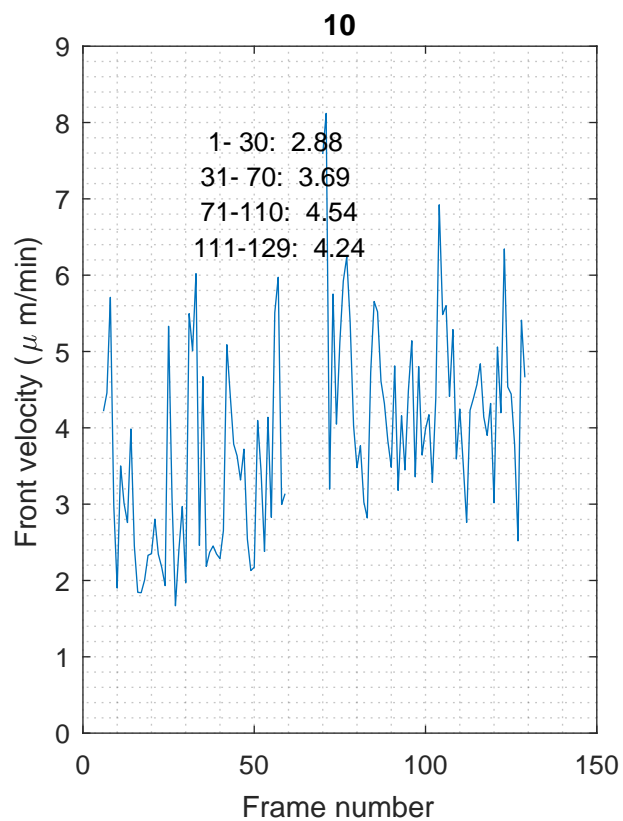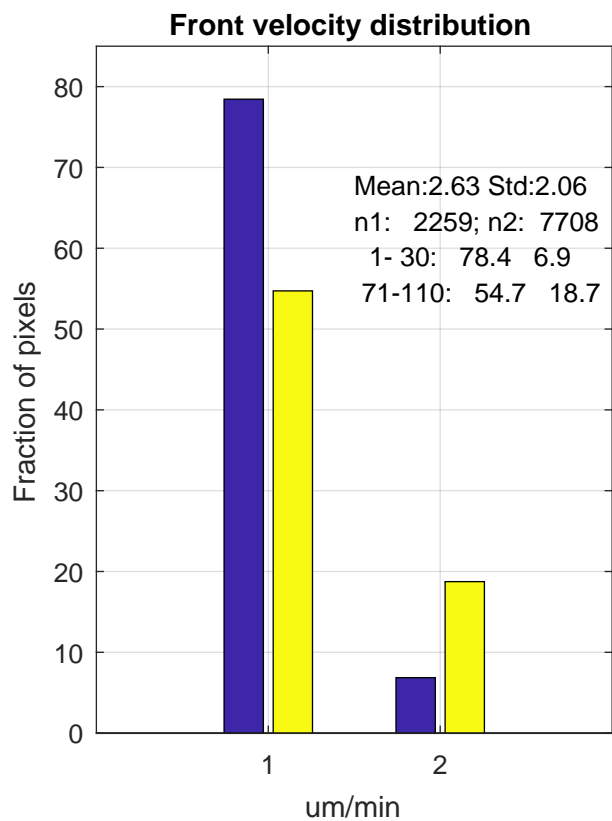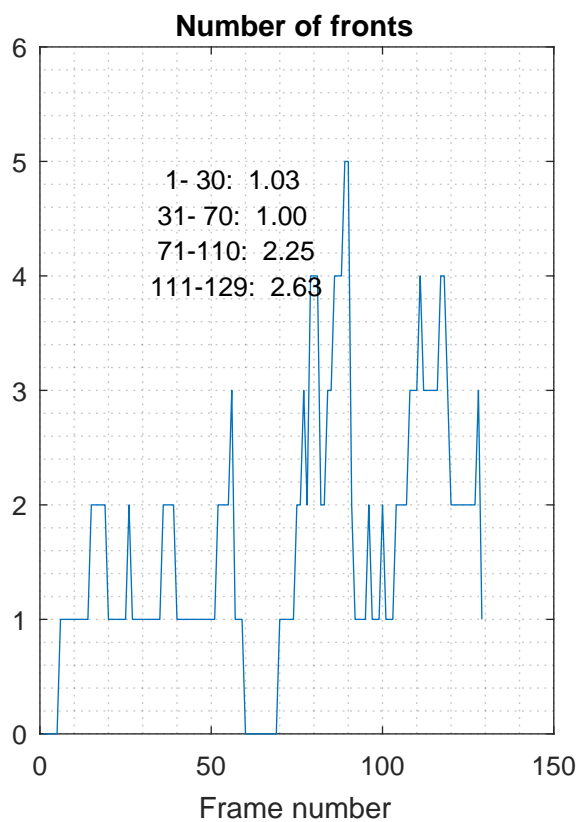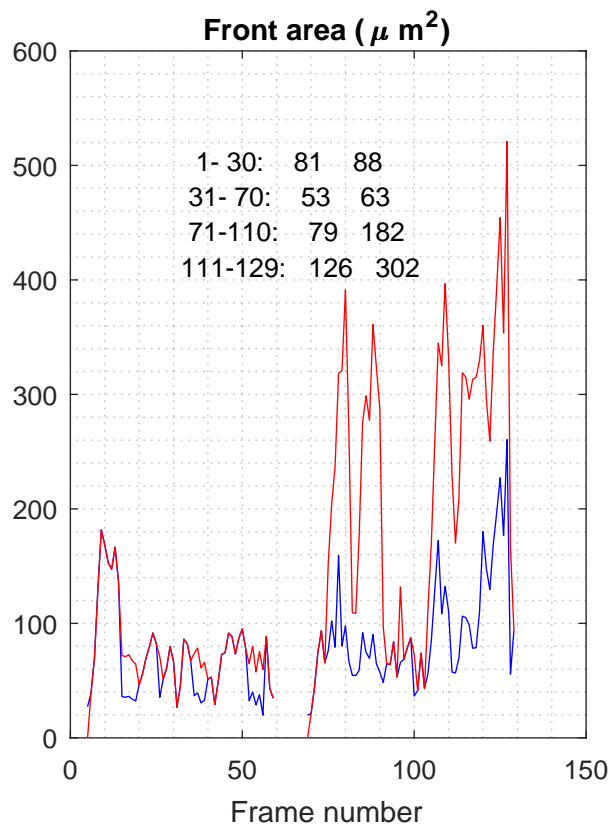

Supplement: Supplementary file 17 — Source Data for Appendix [file MSB-15-e8585-s025.zip › Source_data_for_Appendix/Appendix_Fig_S4/S4D/10.pdf]

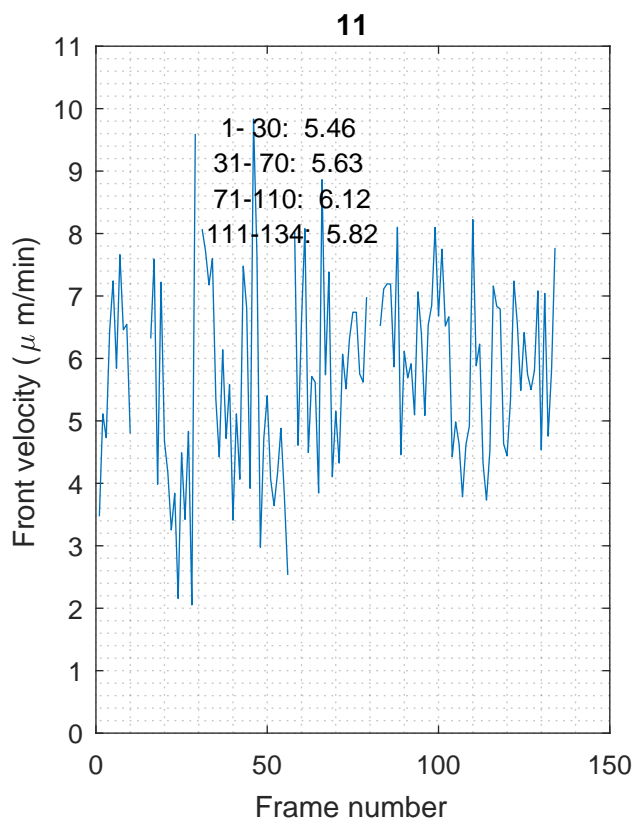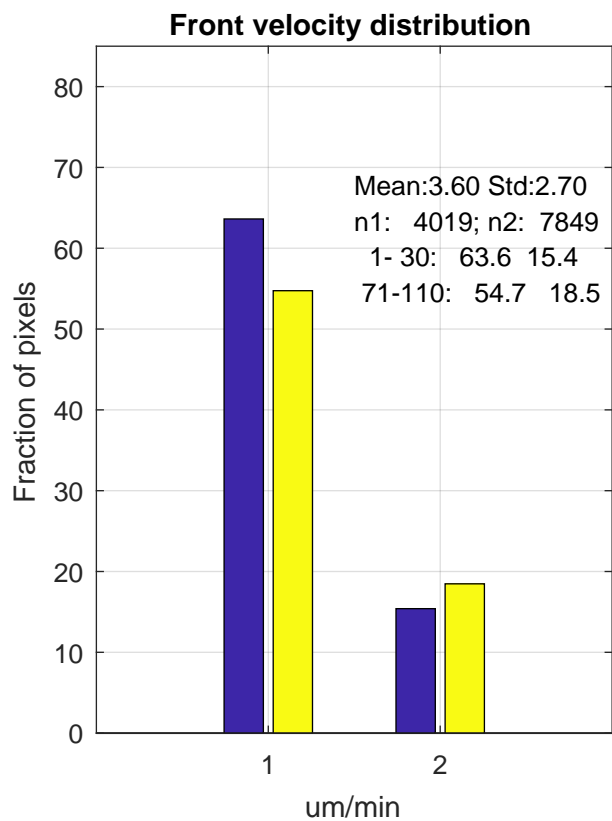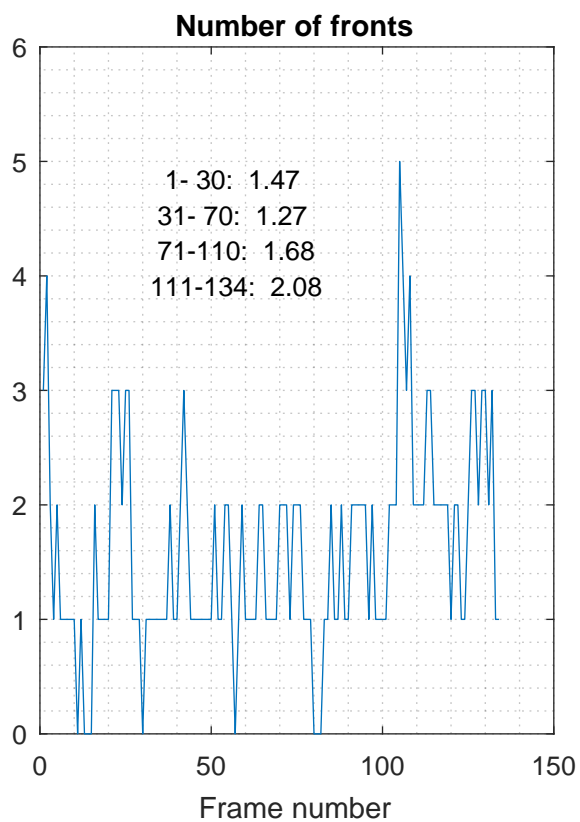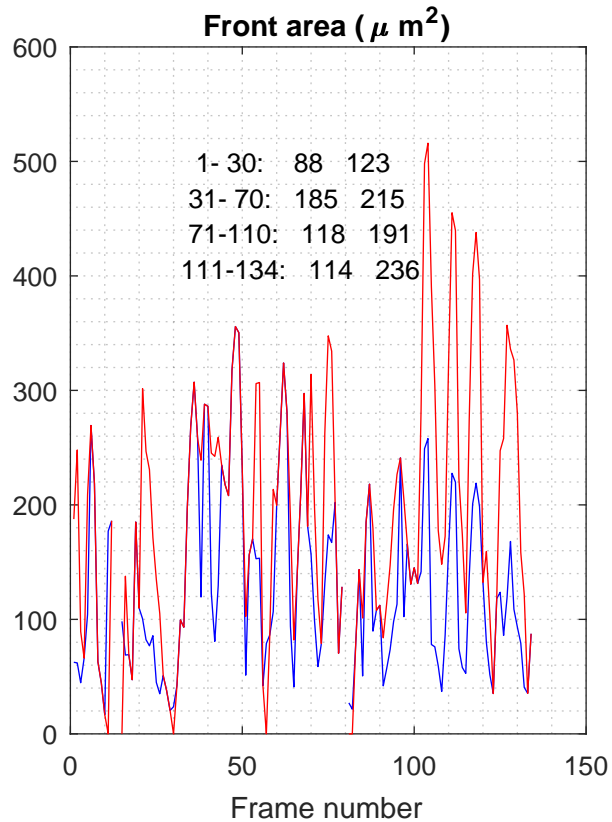

Supplement: Supplementary file 17 — Source Data for Appendix [file MSB-15-e8585-s025.zip › Source_data_for_Appendix/Appendix_Fig_S4/S4D/11.pdf]

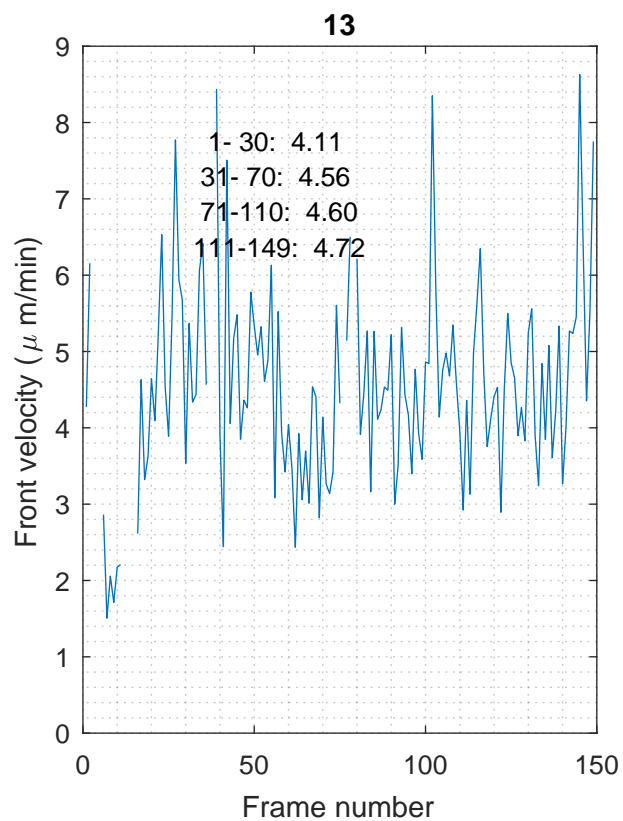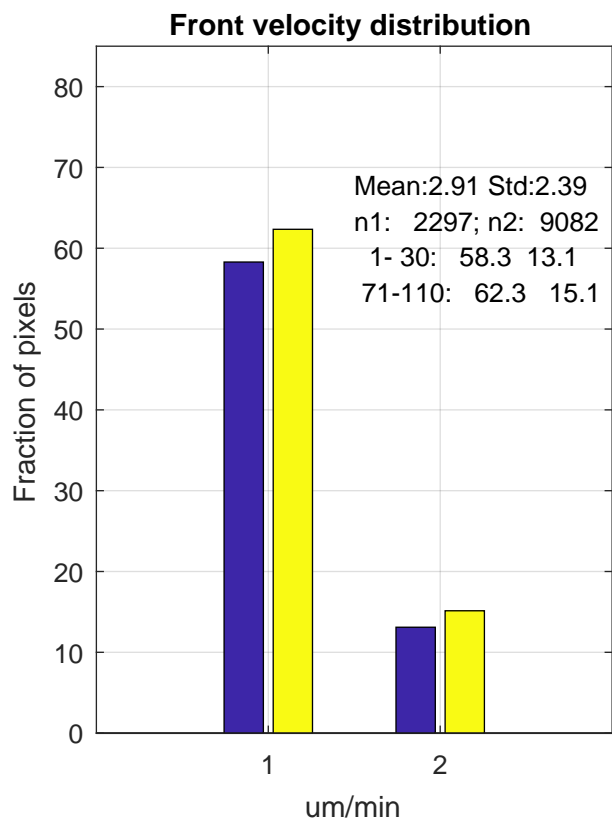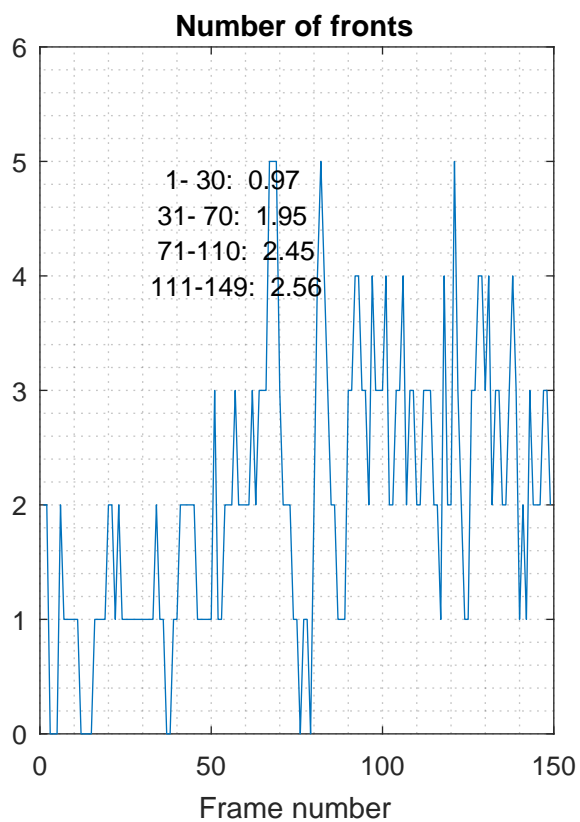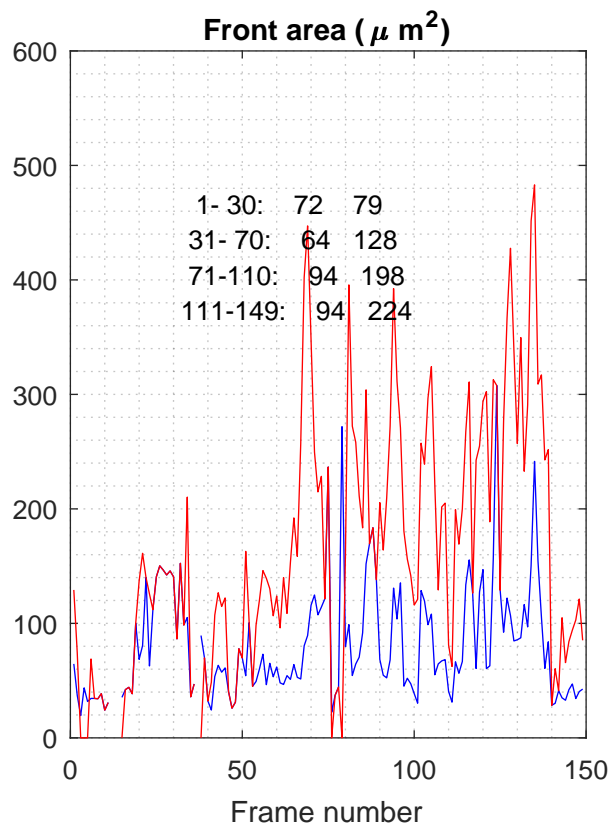

Supplement: Supplementary file 17 — Source Data for Appendix [file MSB-15-e8585-s025.zip › Source_data_for_Appendix/Appendix_Fig_S4/S4D/13.pdf]

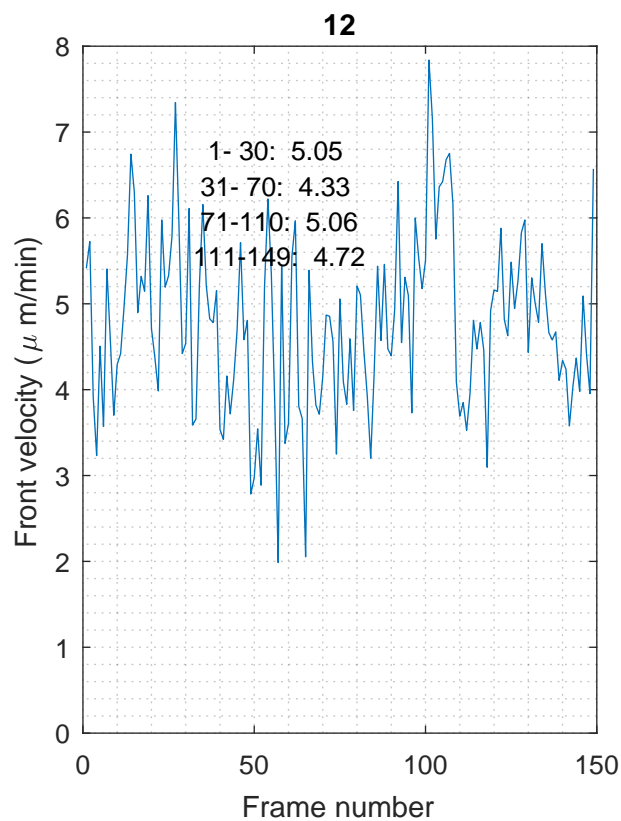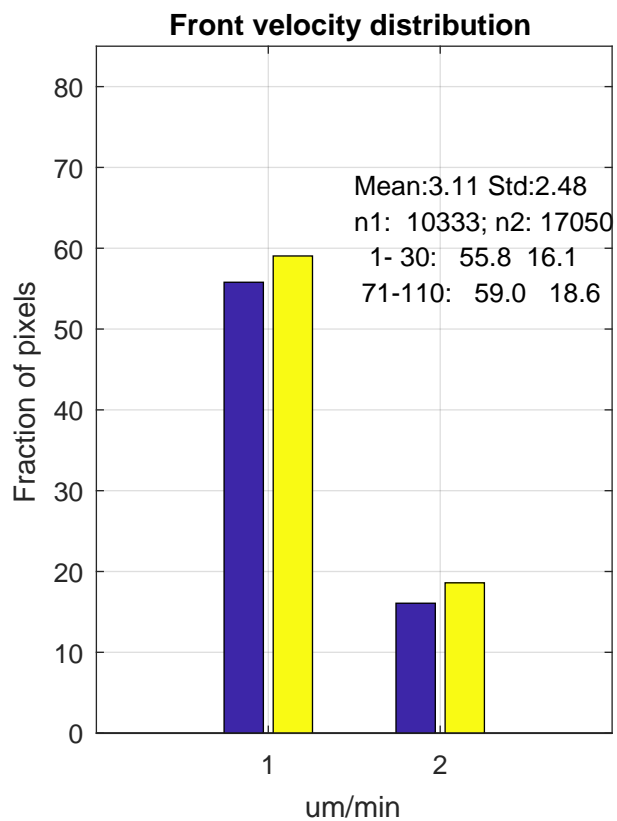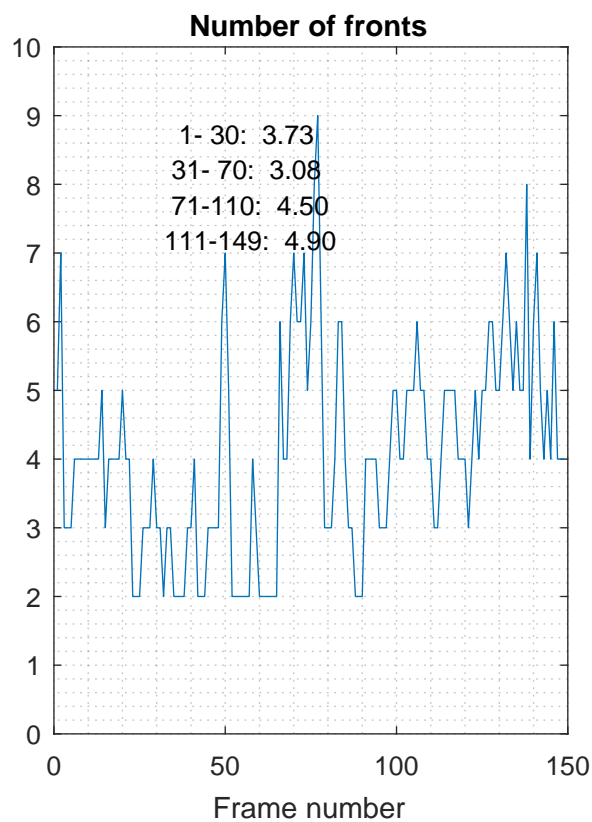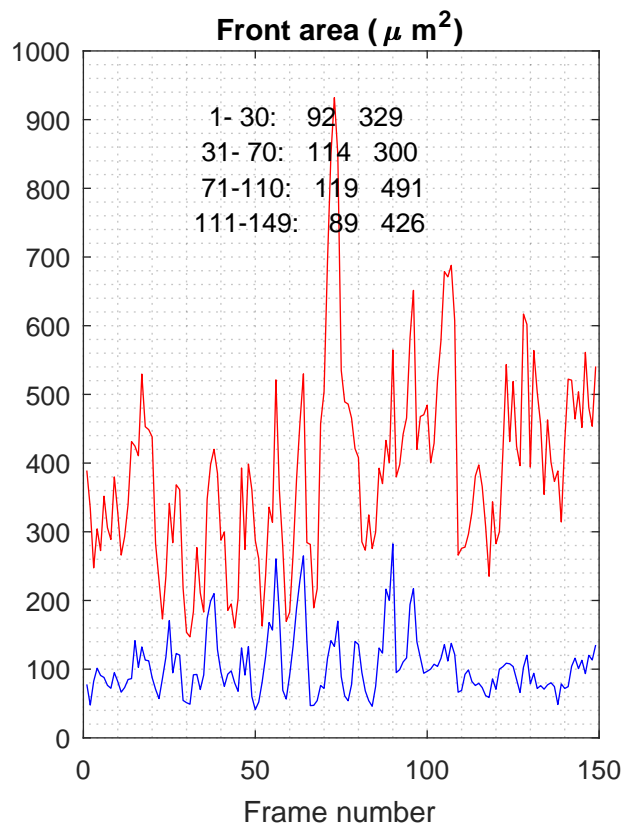

Supplement: Supplementary file 17 — Source Data for Appendix [file MSB-15-e8585-s025.zip › Source_data_for_Appendix/Appendix_Fig_S4/S4D/12.pdf]

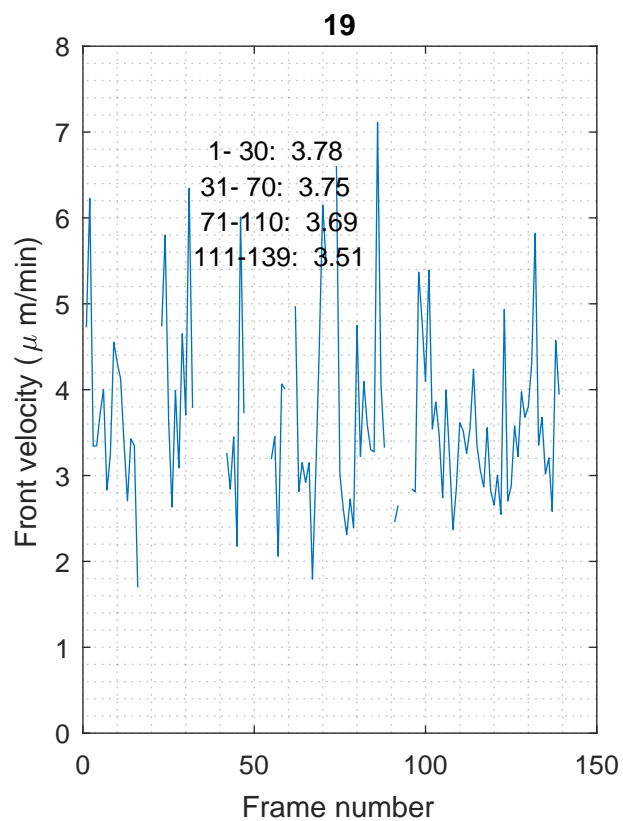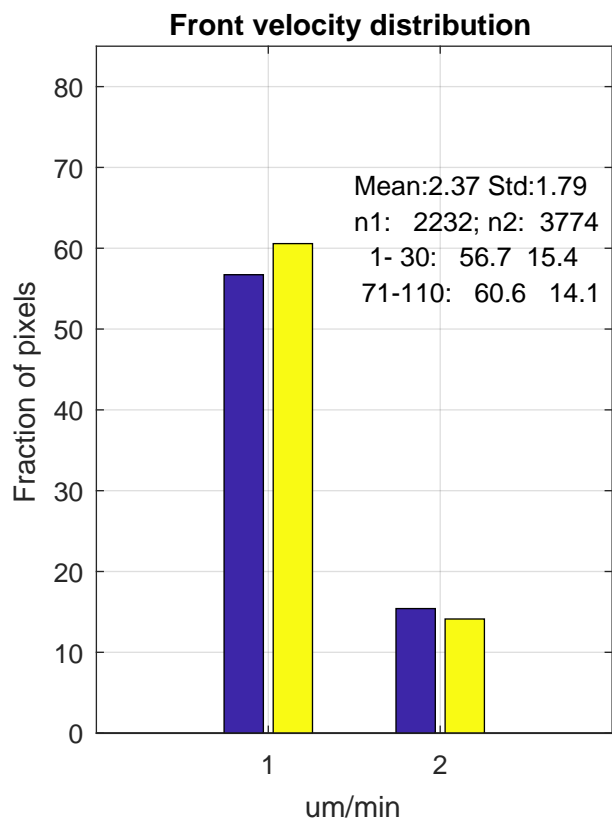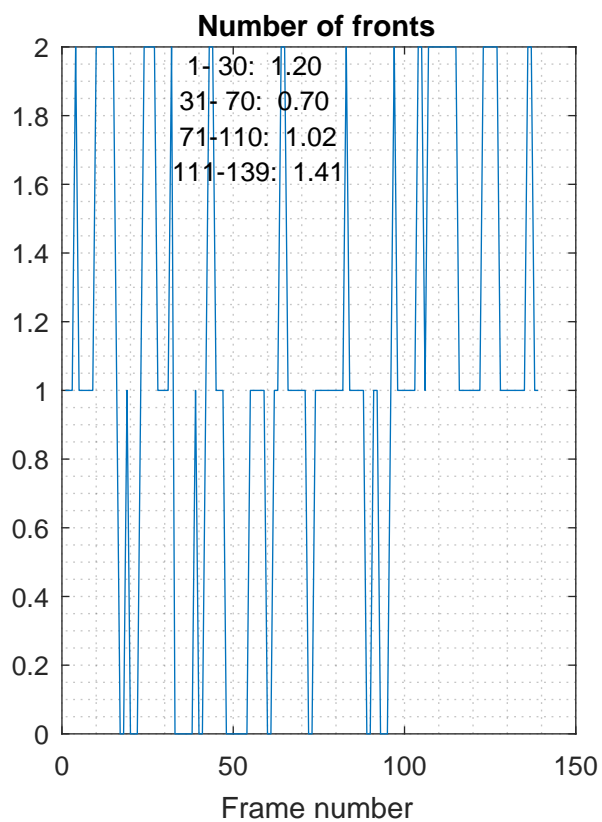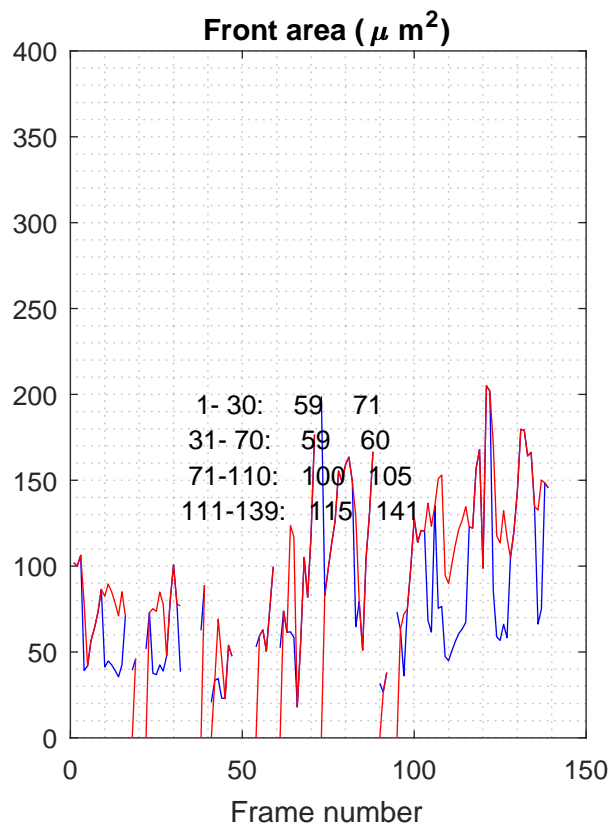

Supplement: Supplementary file 17 — Source Data for Appendix [file MSB-15-e8585-s025.zip › Source_data_for_Appendix/Appendix_Fig_S4/S4D/19.pdf]

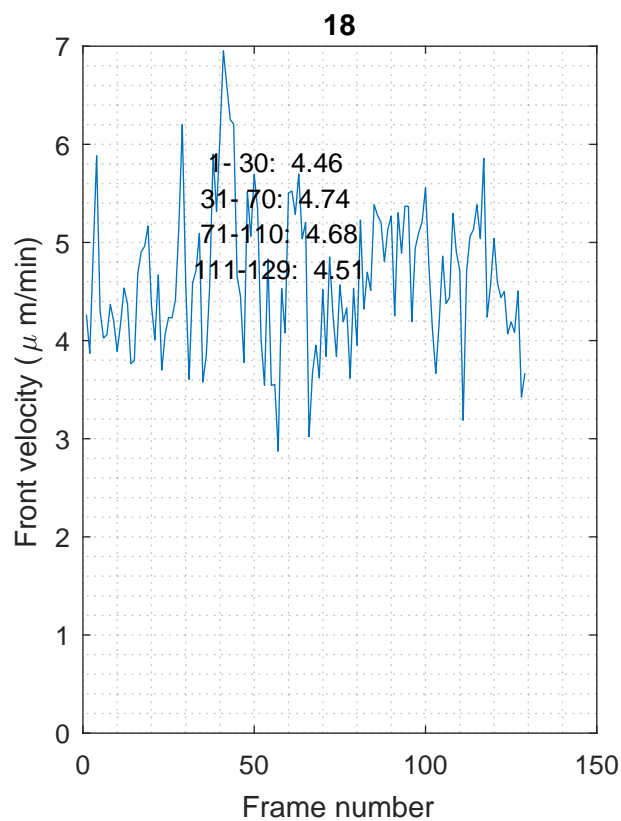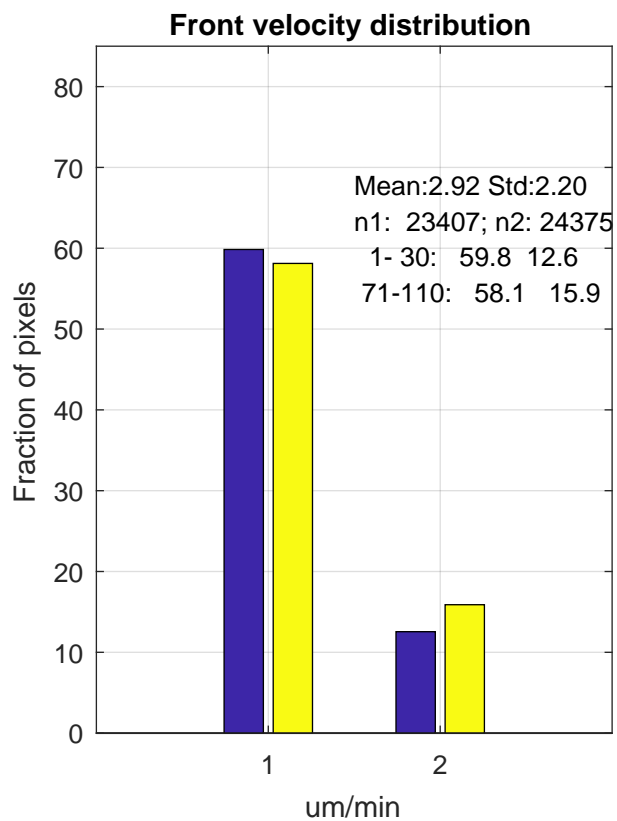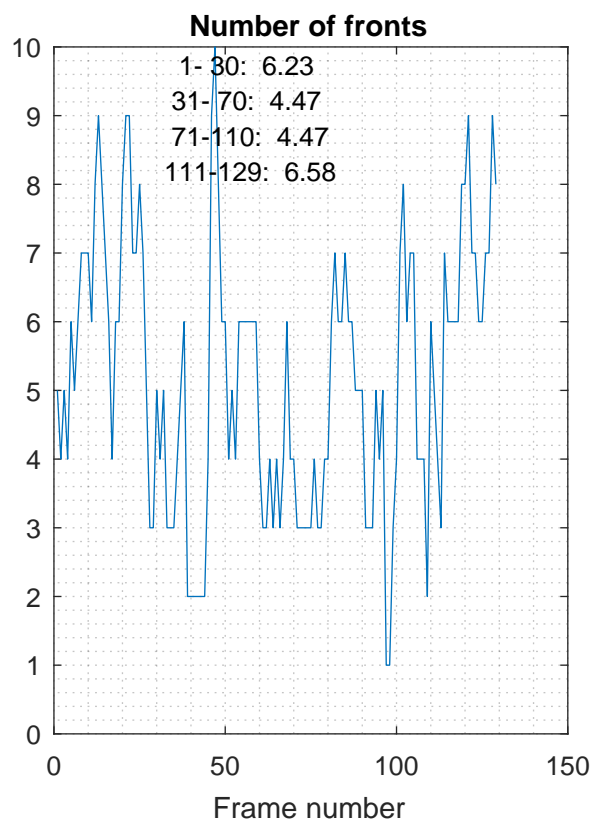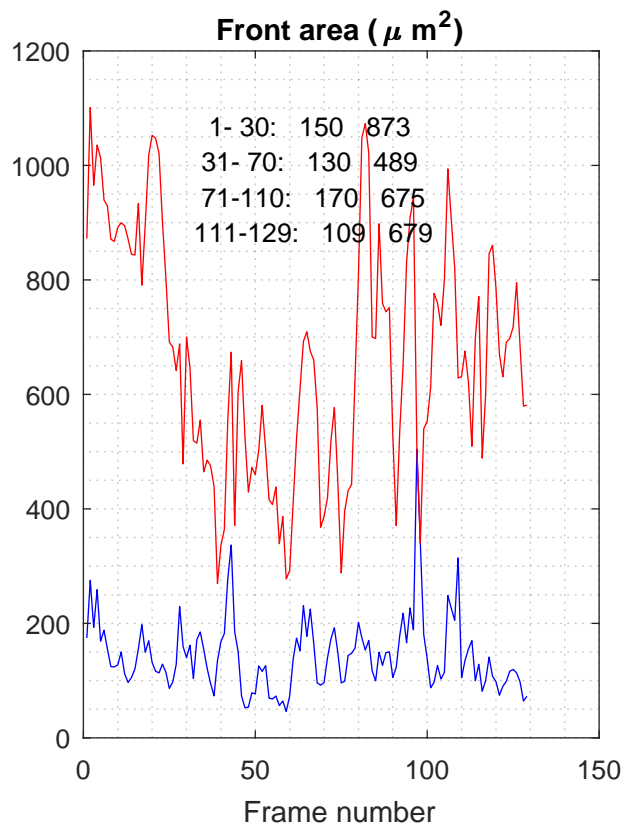

Supplement: Supplementary file 17 — Source Data for Appendix [file MSB-15-e8585-s025.zip › Source_data_for_Appendix/Appendix_Fig_S4/S4D/18.pdf]

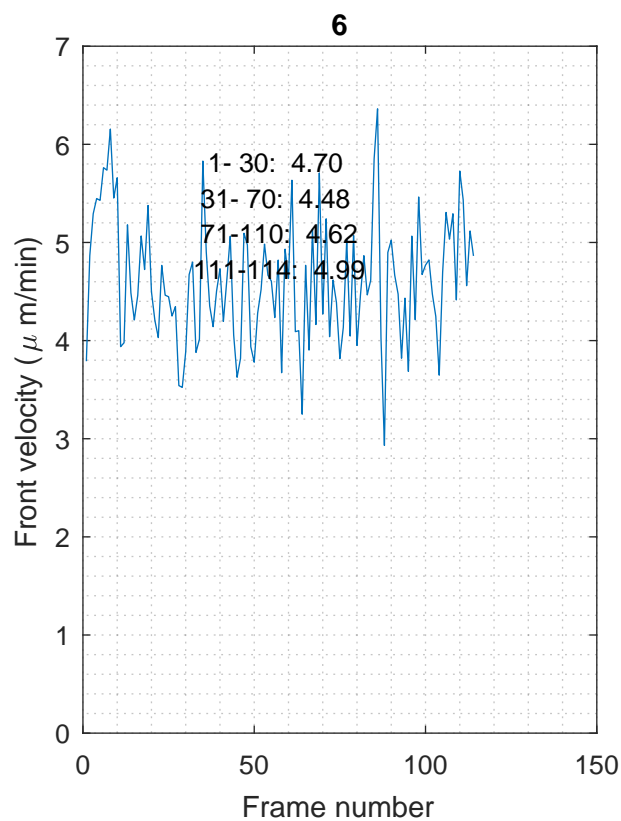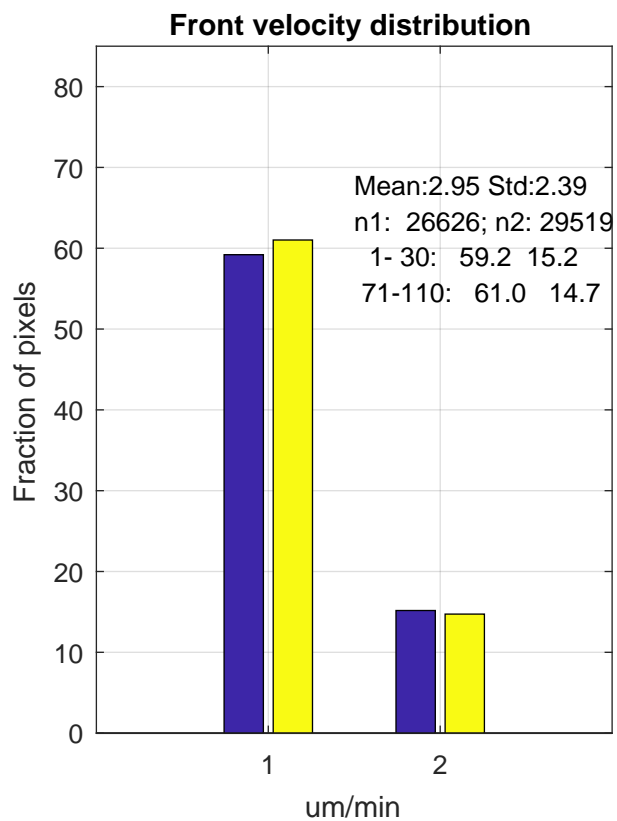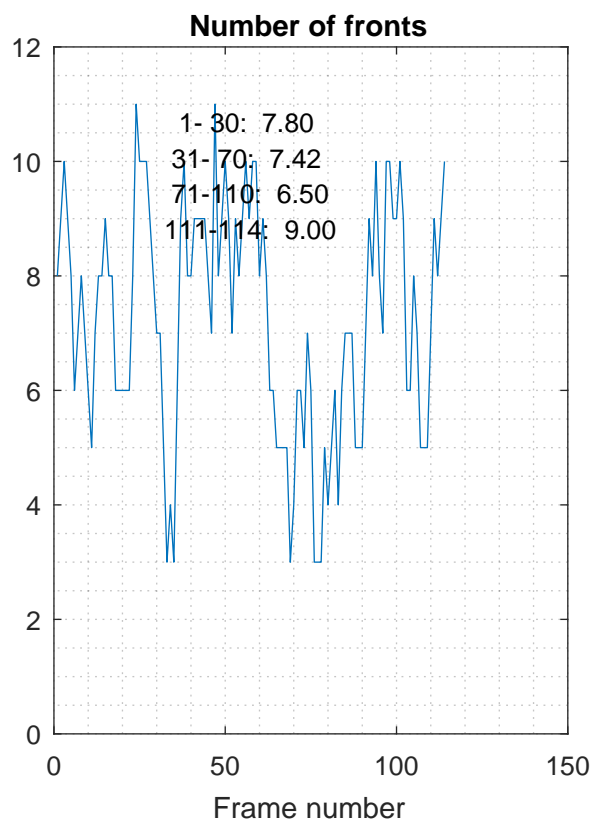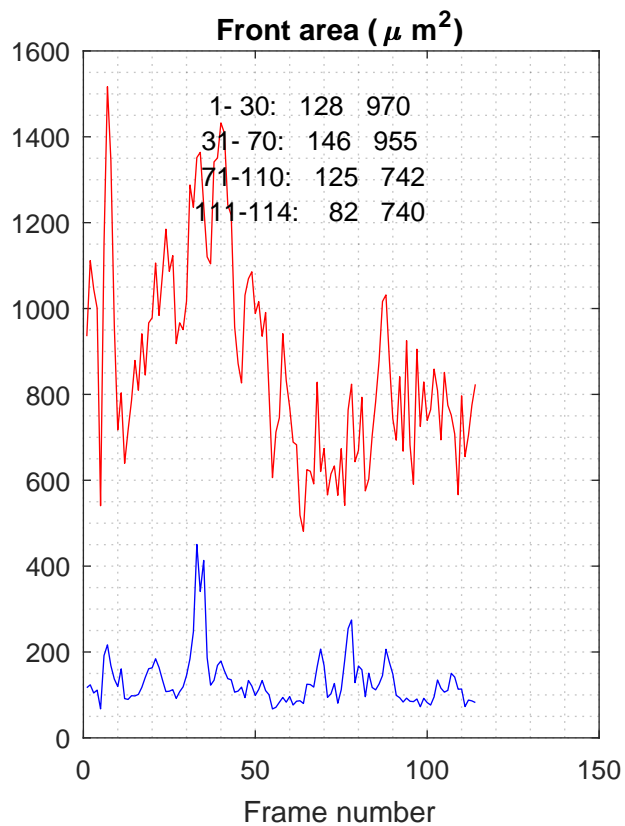

Supplement: Supplementary file 17 — Source Data for Appendix [file MSB-15-e8585-s025.zip › Source_data_for_Appendix/Appendix_Fig_S4/S4D/6.pdf]

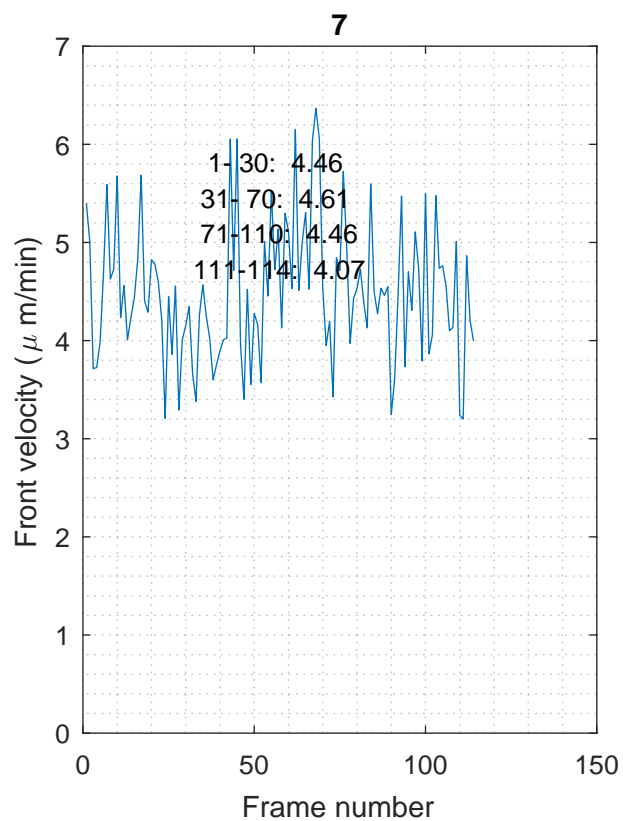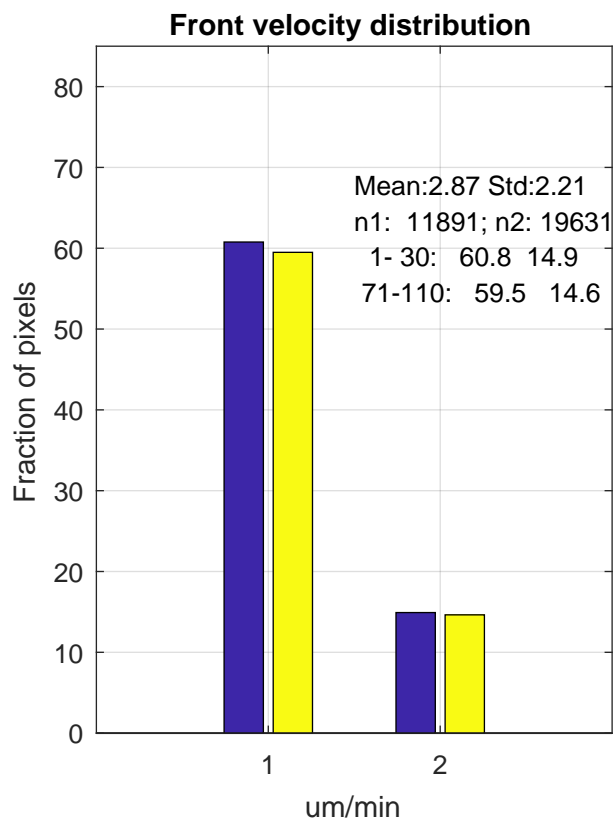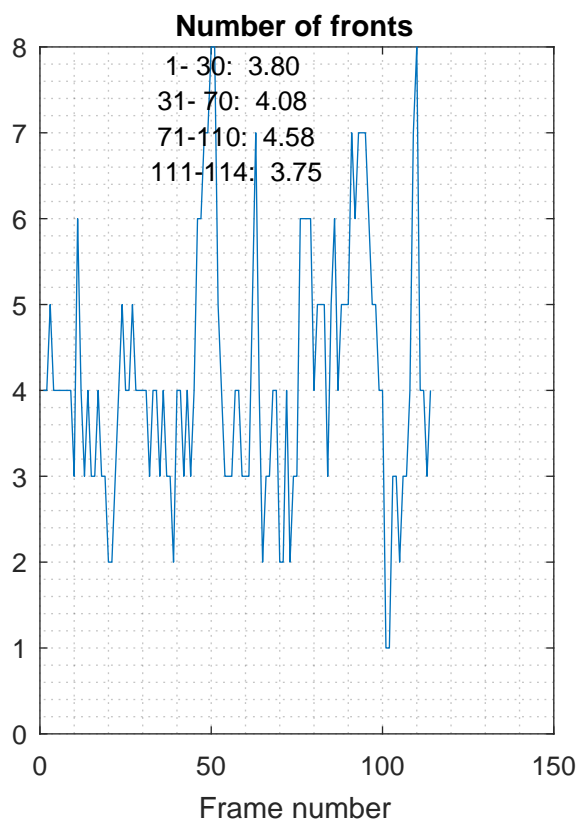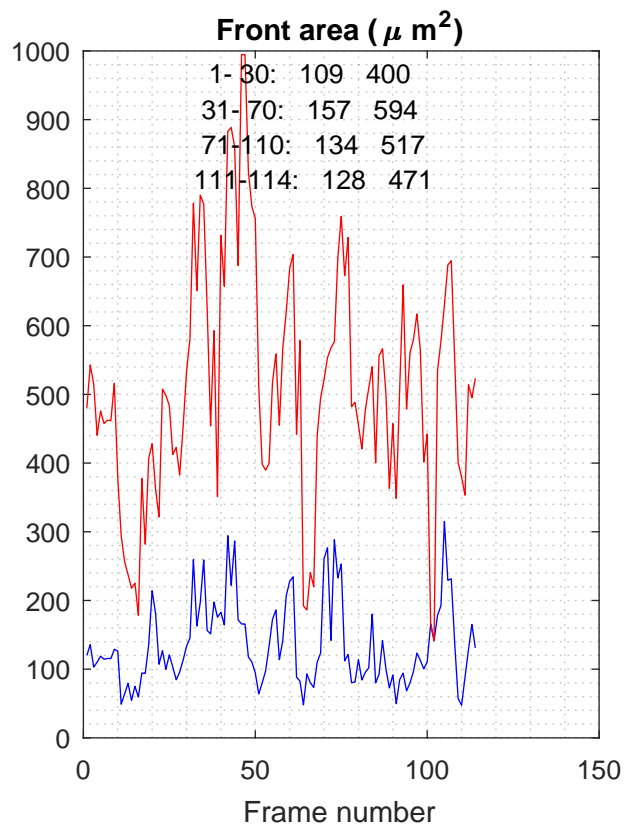

Supplement: Supplementary file 17 — Source Data for Appendix [file MSB-15-e8585-s025.zip › Source_data_for_Appendix/Appendix_Fig_S4/S4D/7.pdf]

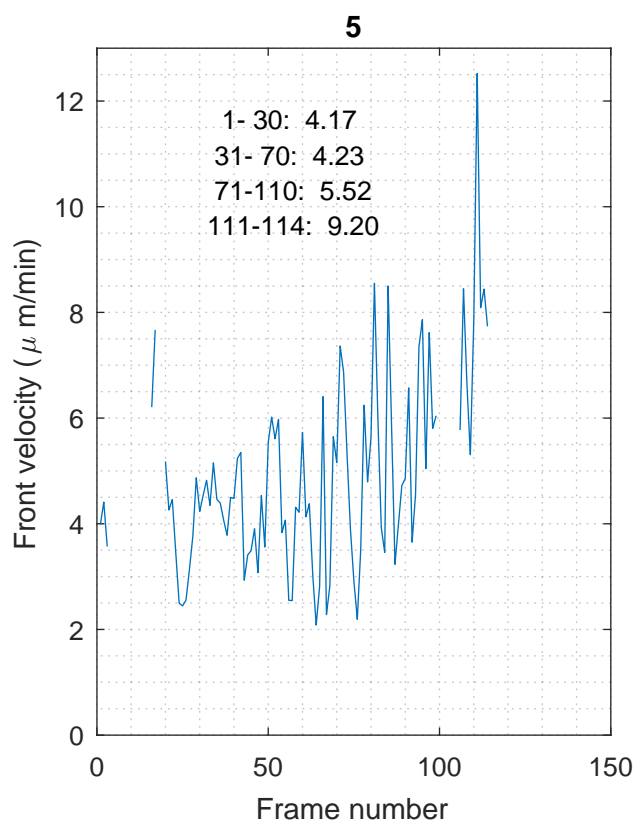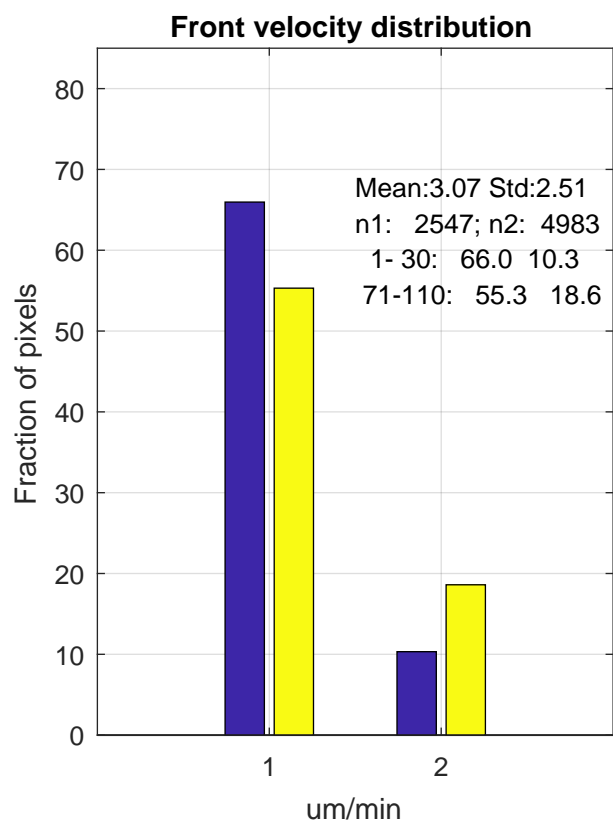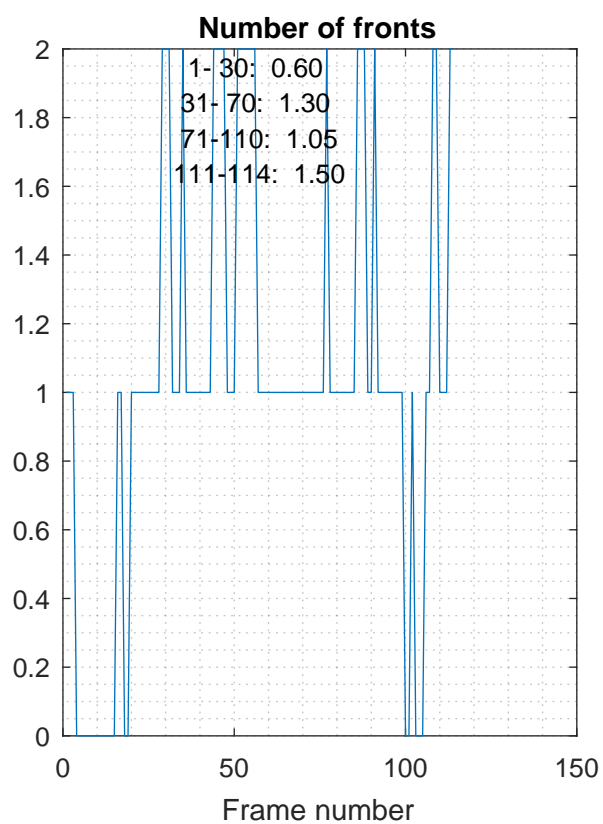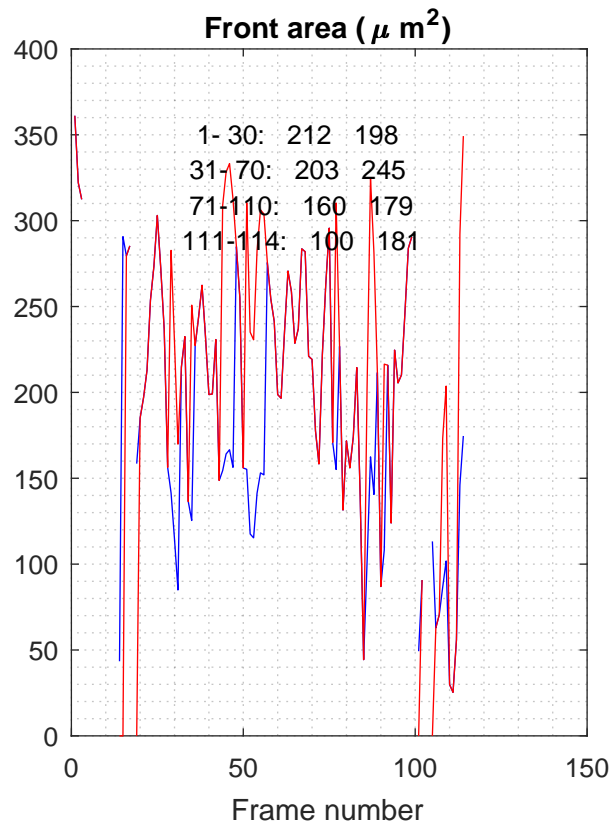

Supplement: Supplementary file 17 — Source Data for Appendix [file MSB-15-e8585-s025.zip › Source_data_for_Appendix/Appendix_Fig_S4/S4D/5.pdf]

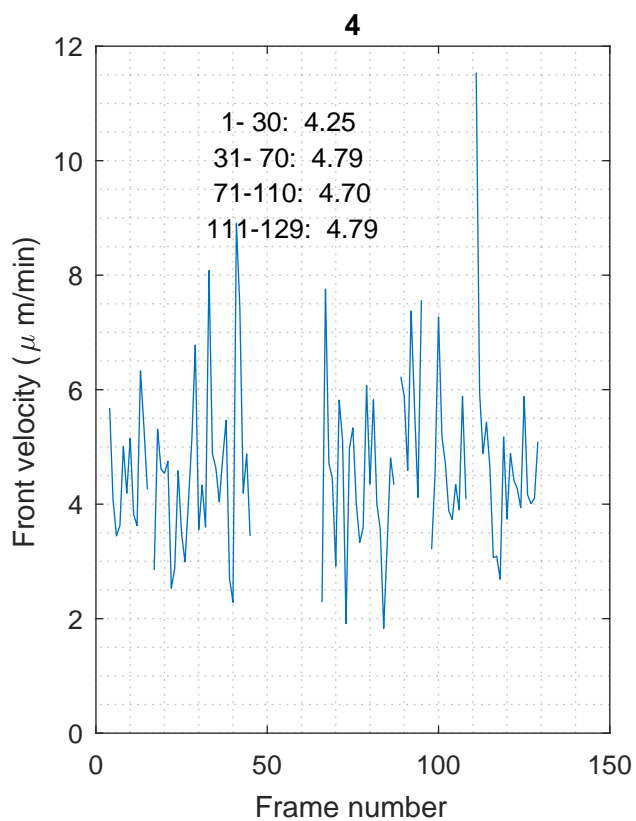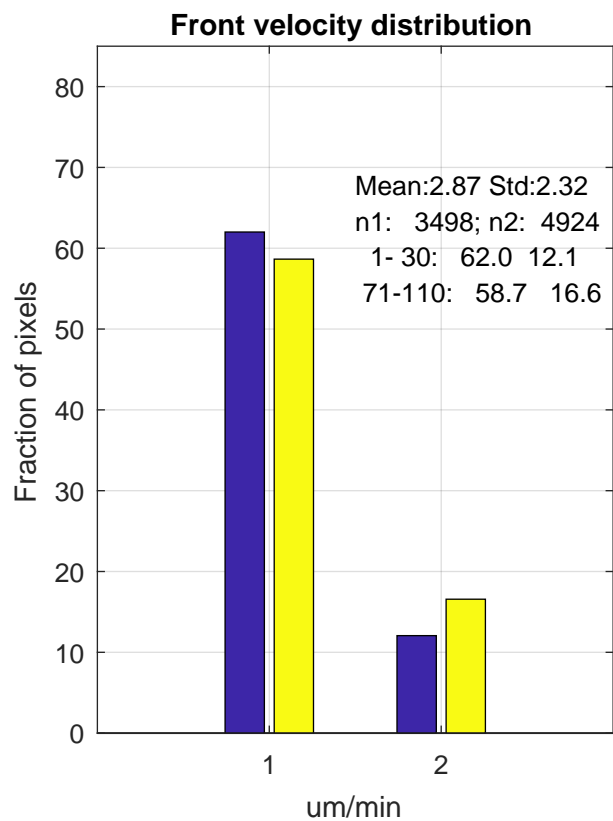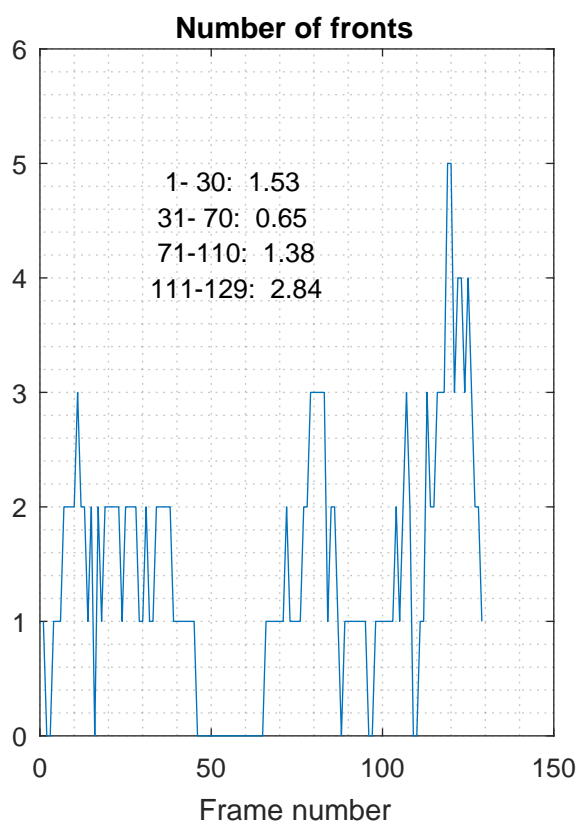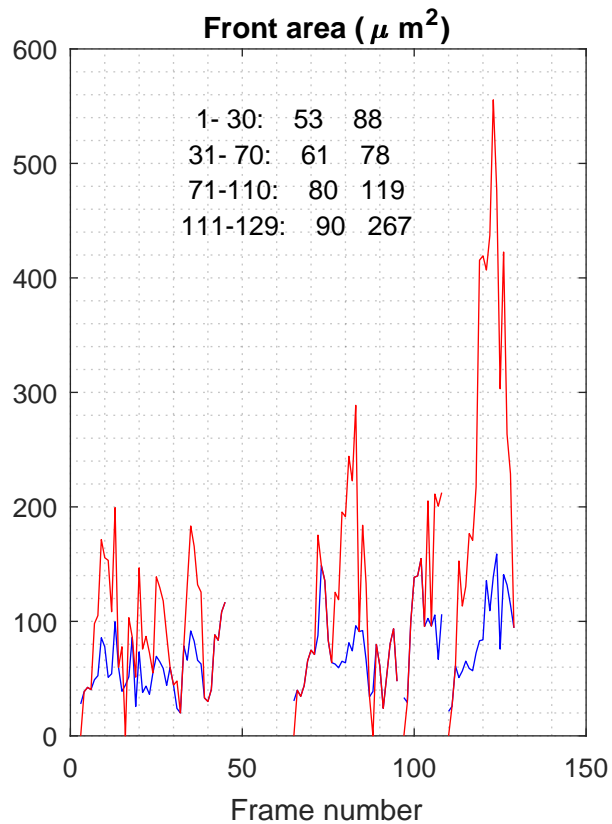

Supplement: Supplementary file 17 — Source Data for Appendix [file MSB-15-e8585-s025.zip › Source_data_for_Appendix/Appendix_Fig_S4/S4D/4.pdf]

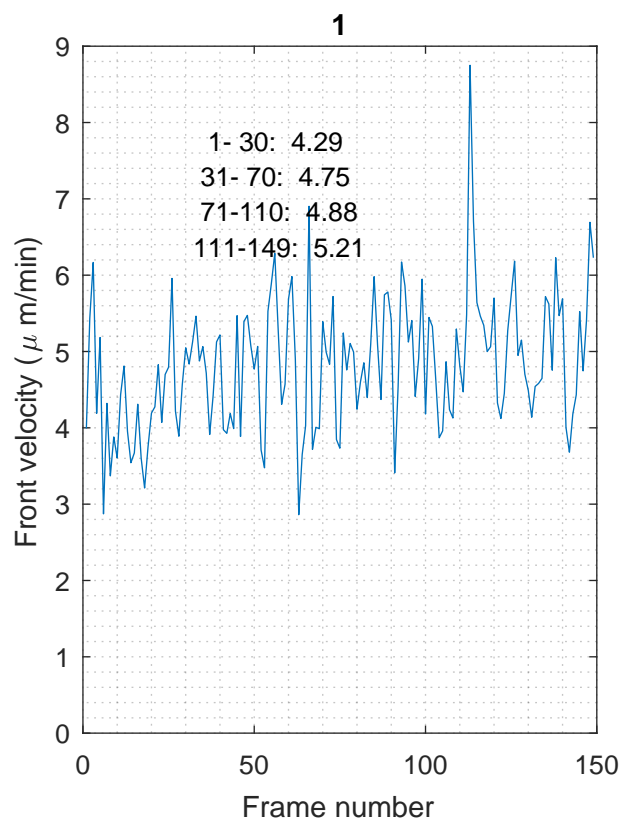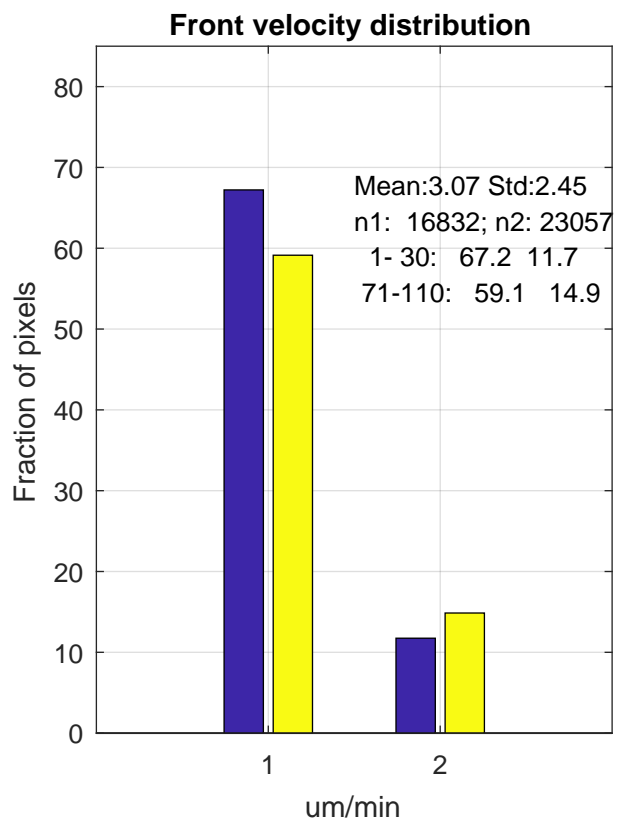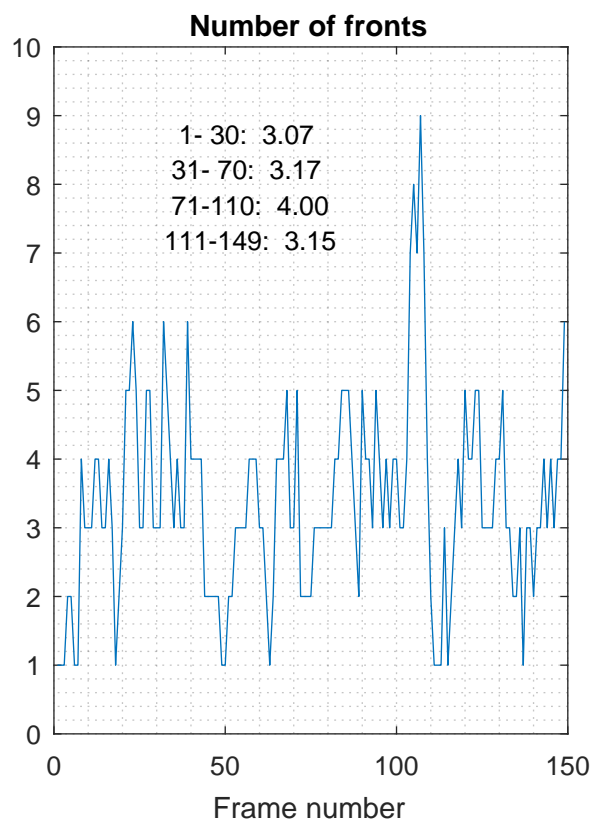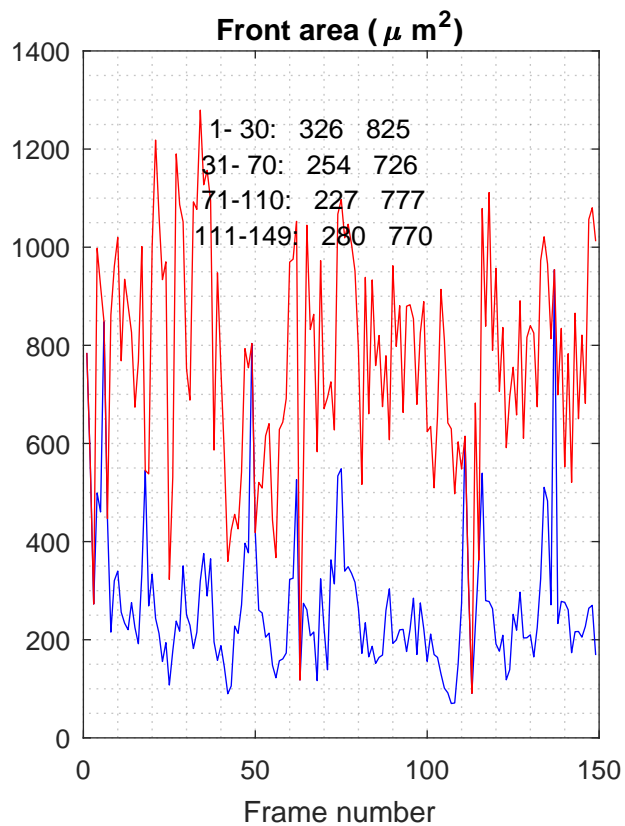

Supplement: Supplementary file 17 — Source Data for Appendix [file MSB-15-e8585-s025.zip › Source_data_for_Appendix/Appendix_Fig_S4/S4D/1.pdf]

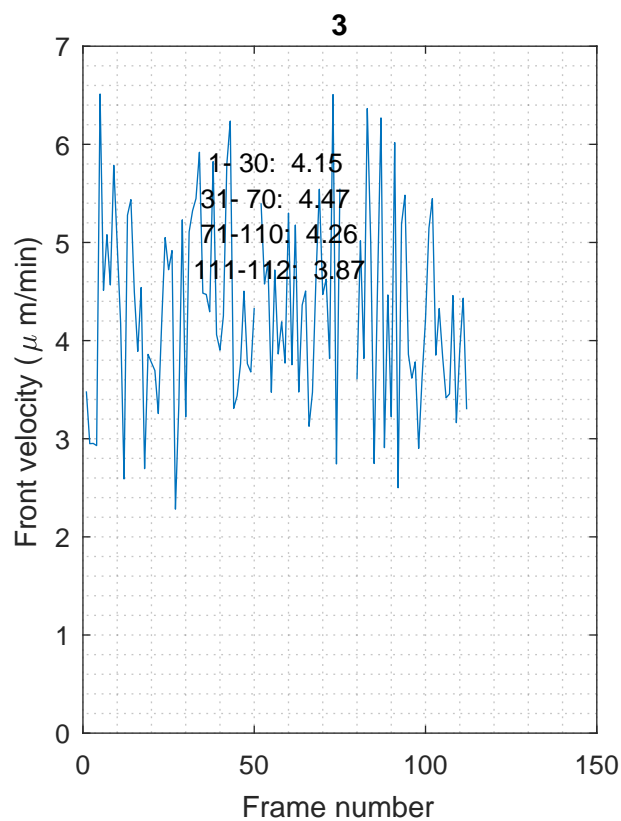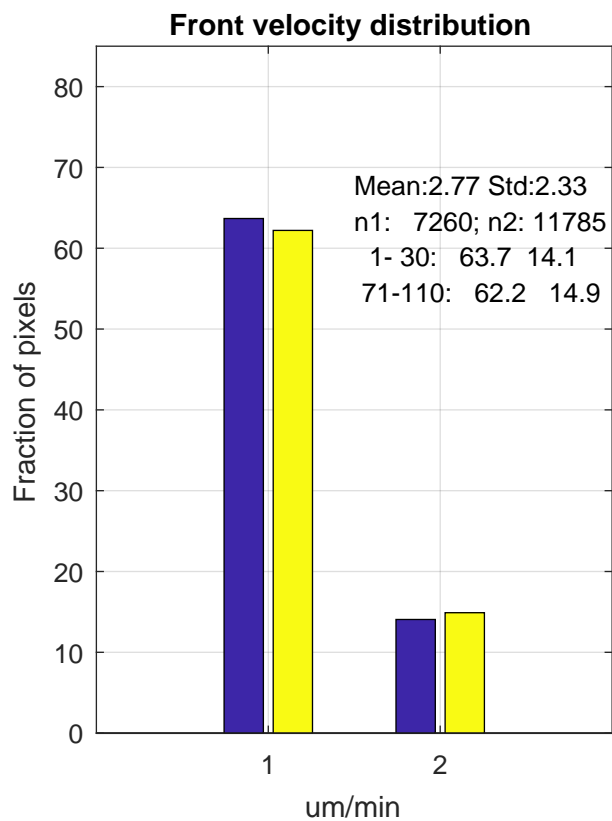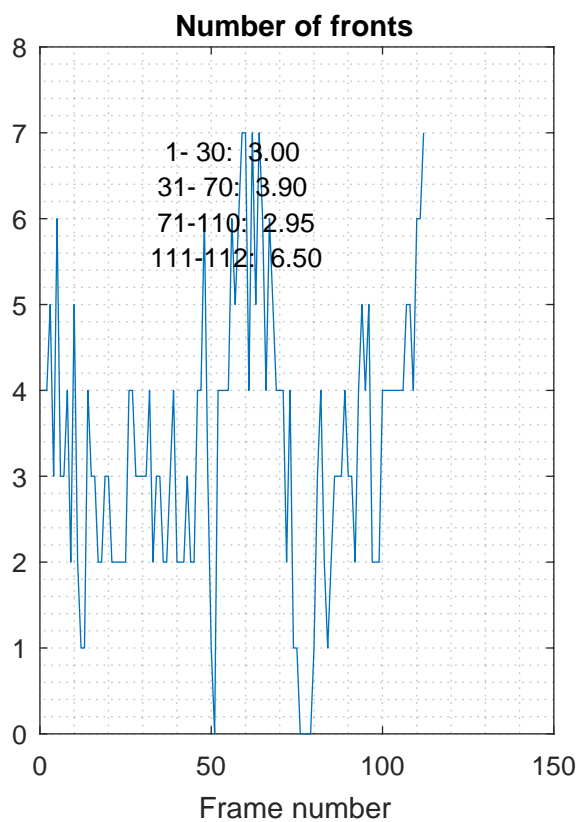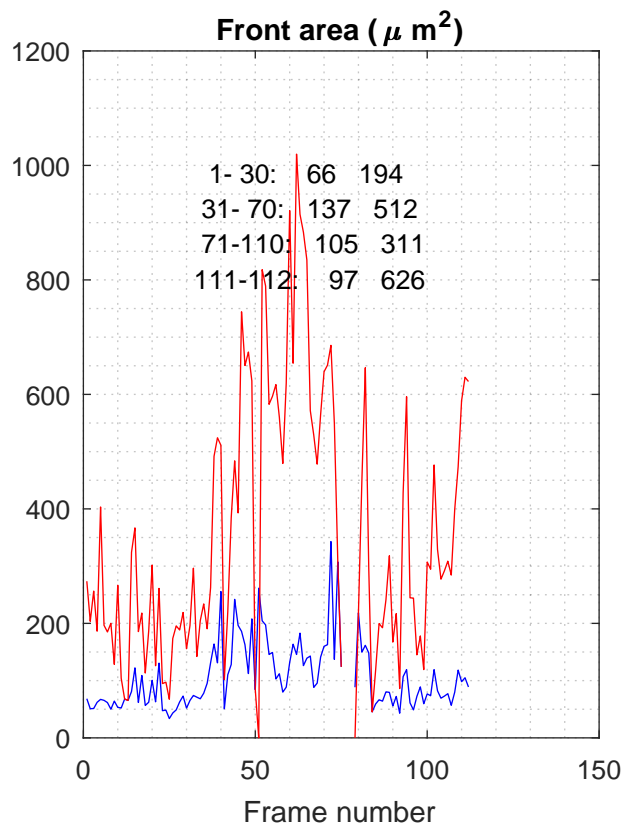

Supplement: Supplementary file 17 — Source Data for Appendix [file MSB-15-e8585-s025.zip › Source_data_for_Appendix/Appendix_Fig_S4/S4D/3.pdf]

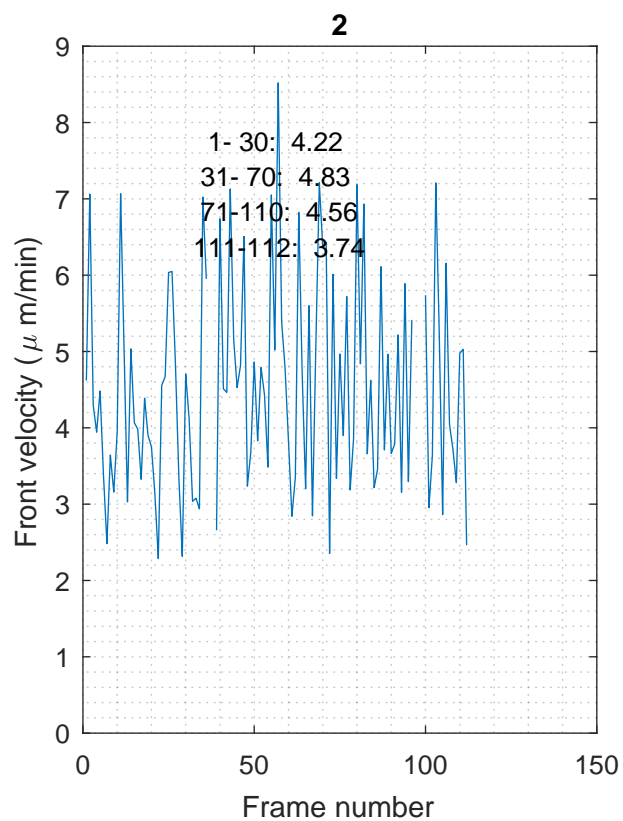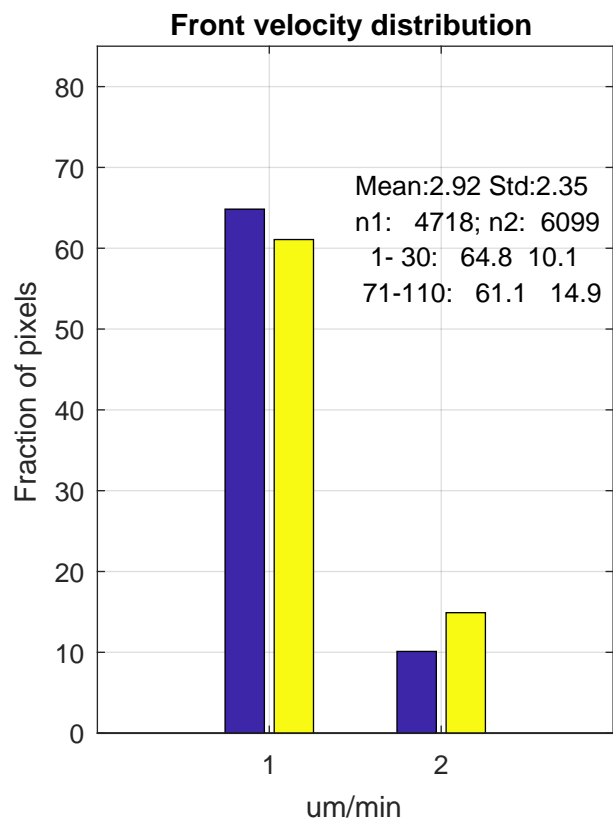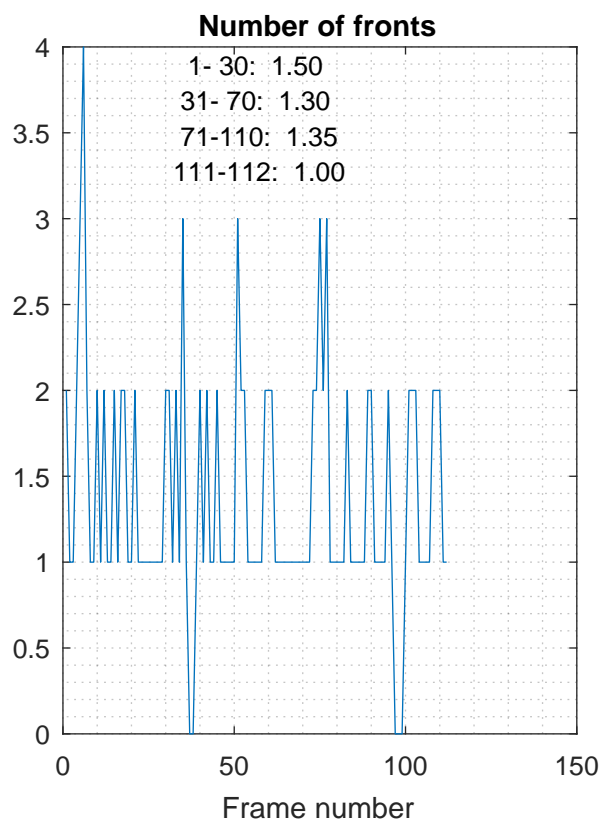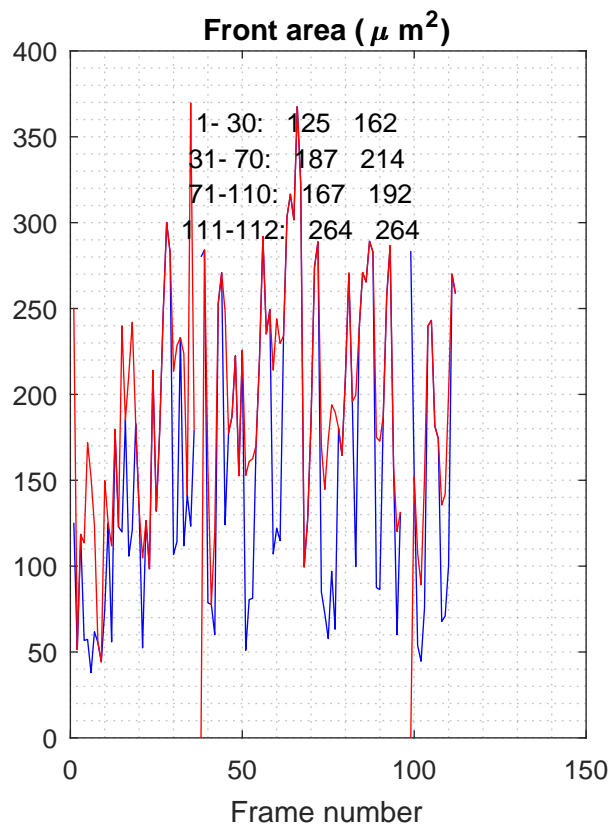

Supplement: Supplementary file 17 — Source Data for Appendix [file MSB-15-e8585-s025.zip › Source_data_for_Appendix/Appendix_Fig_S4/S4D/2.pdf]

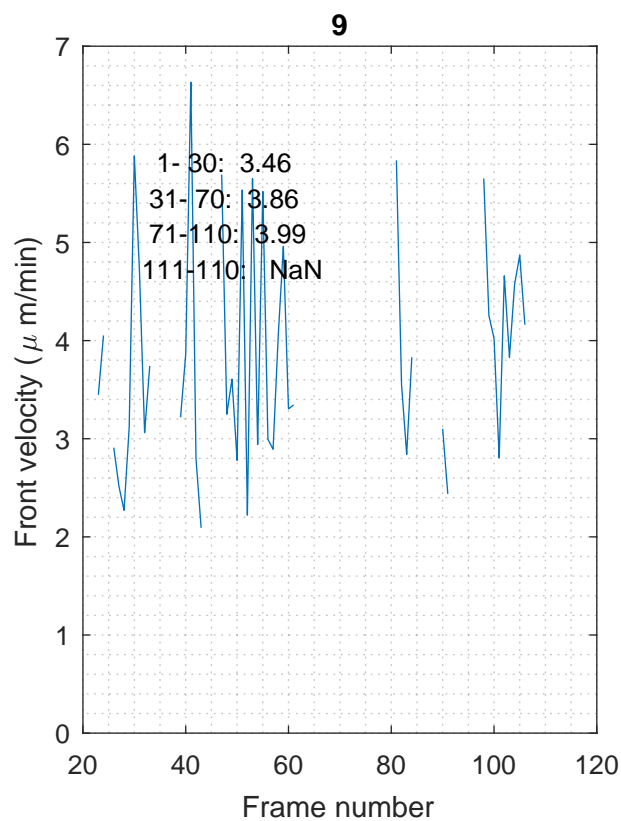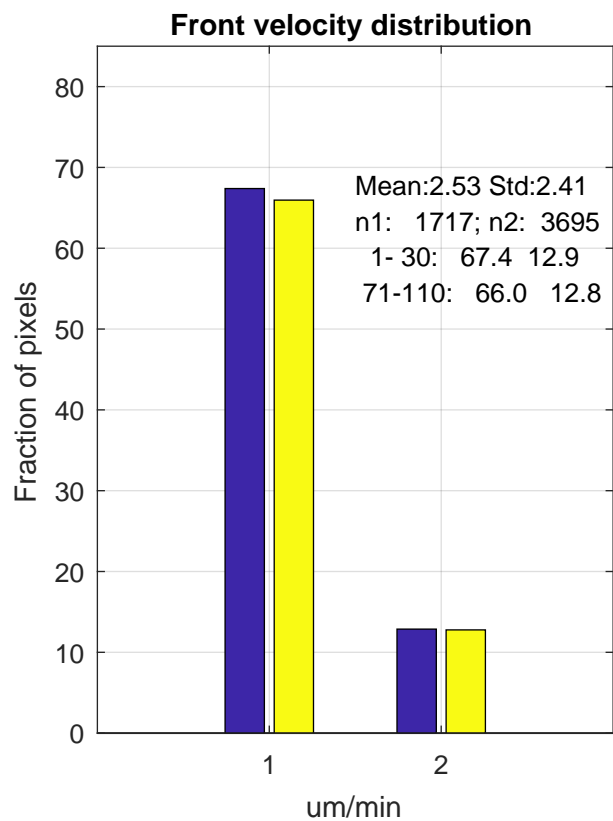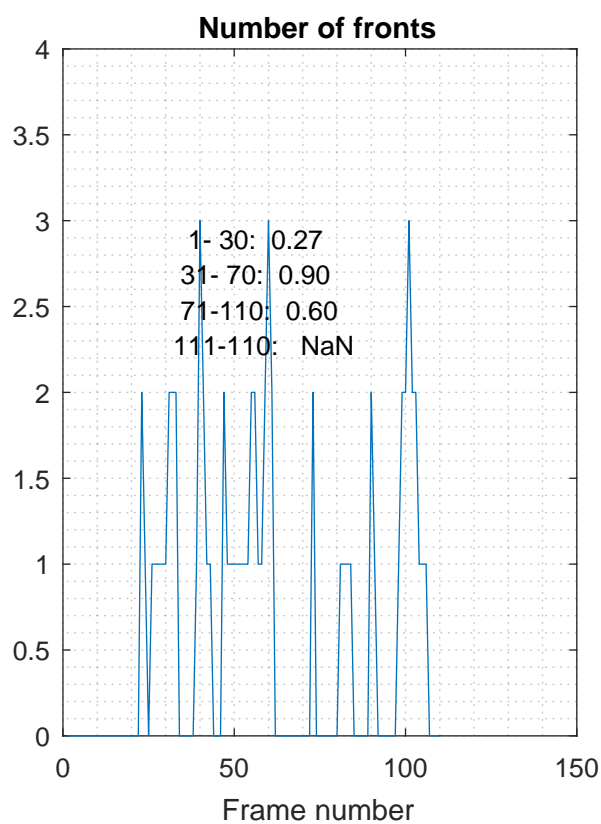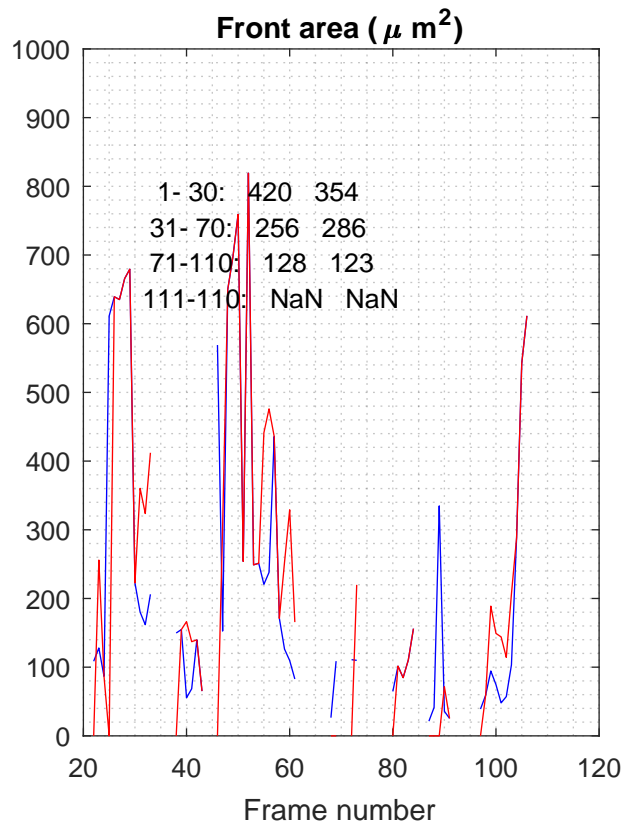

Supplement: Supplementary file 17 — Source Data for Appendix [file MSB-15-e8585-s025.zip › Source_data_for_Appendix/Appendix_Fig_S4/S4E/9.pdf]

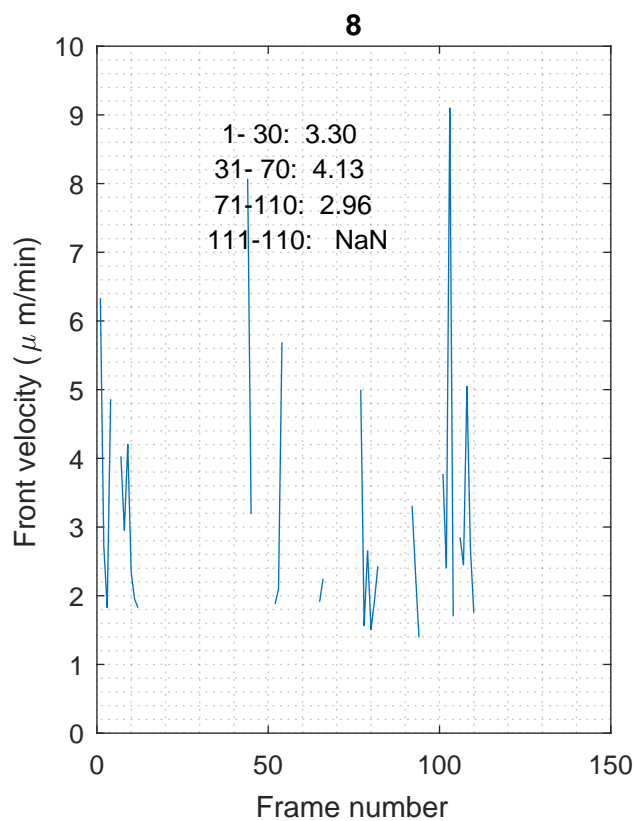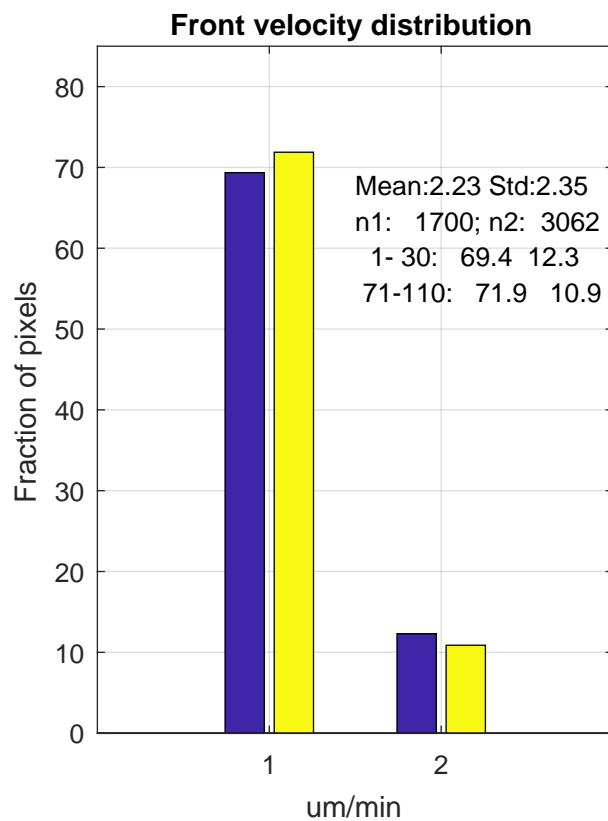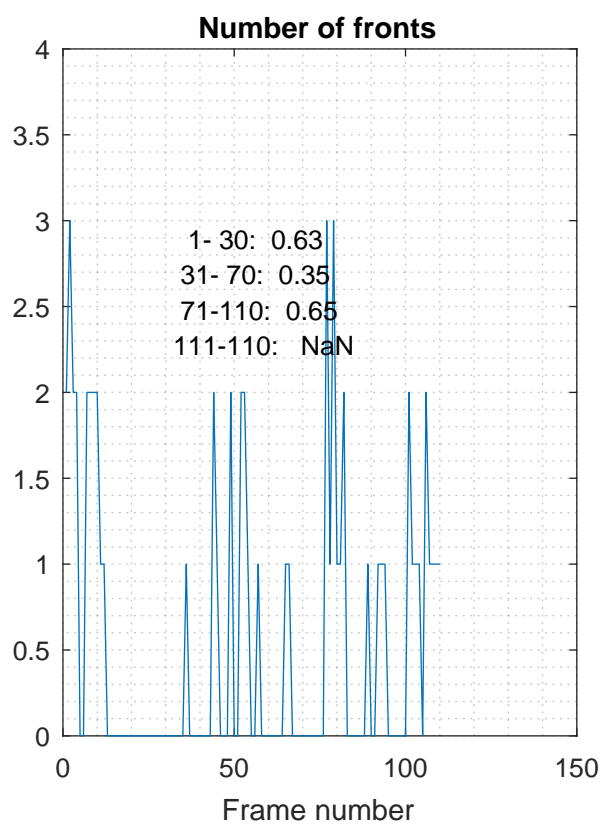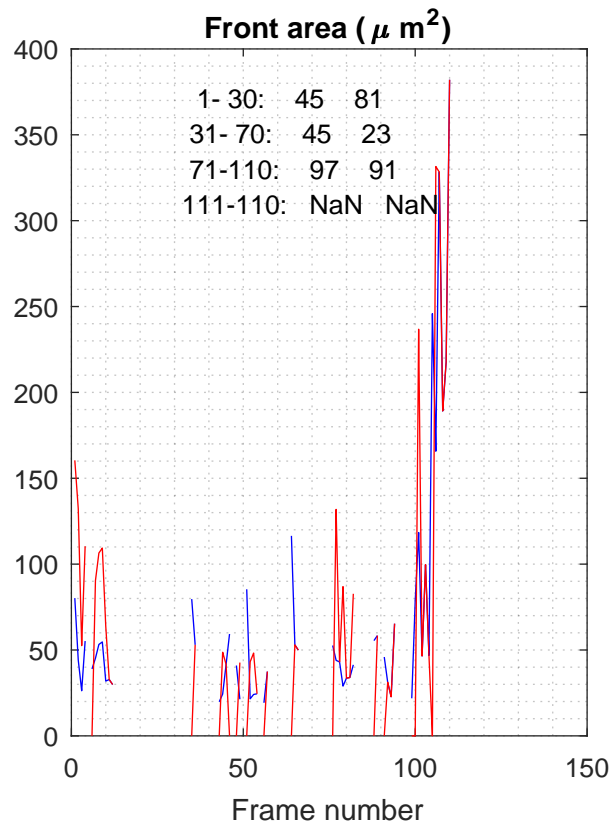

Supplement: Supplementary file 17 — Source Data for Appendix [file MSB-15-e8585-s025.zip › Source_data_for_Appendix/Appendix_Fig_S4/S4E/8.pdf]

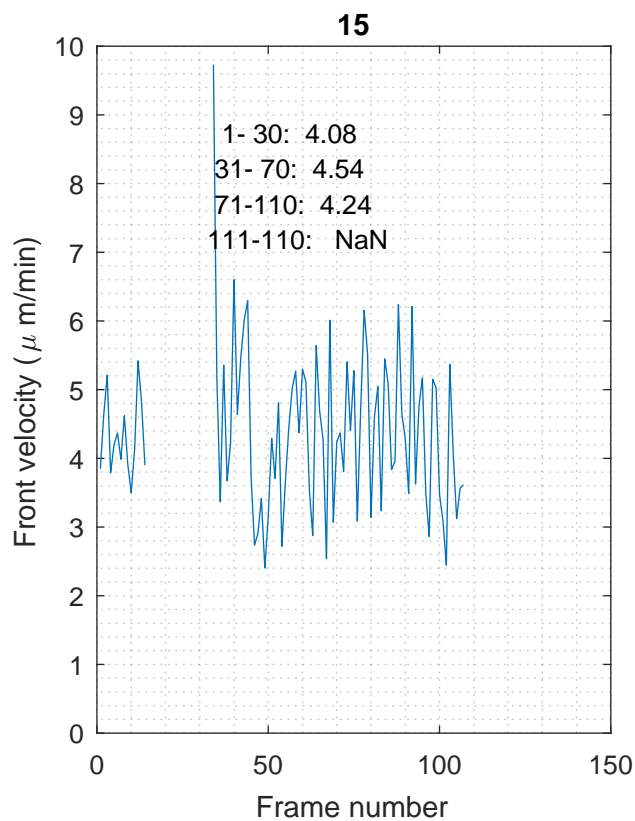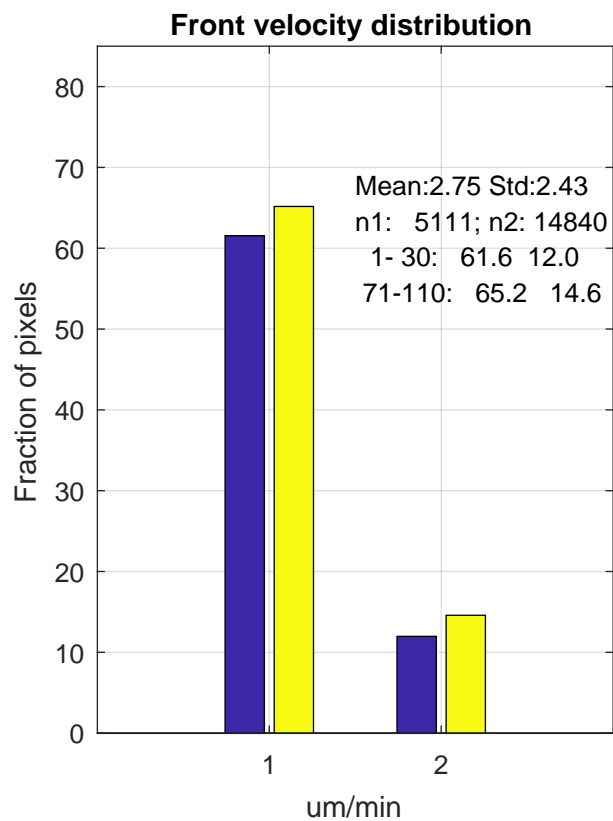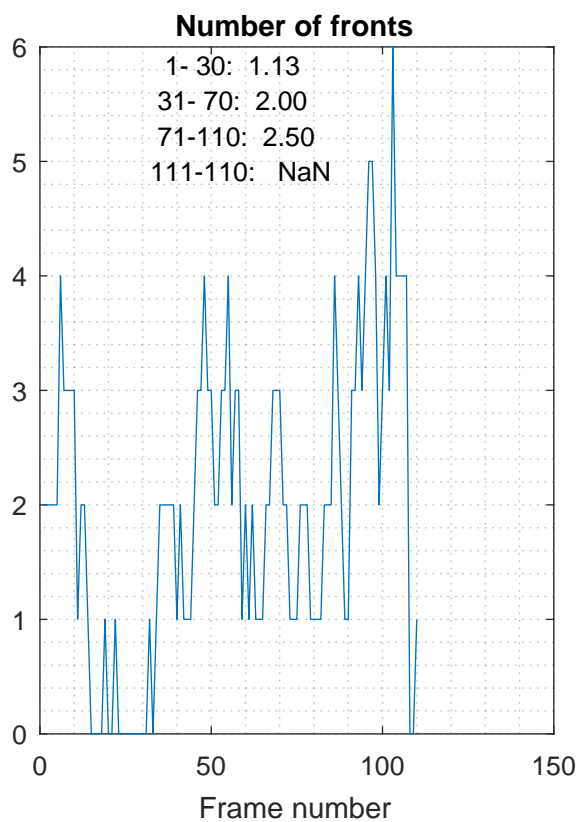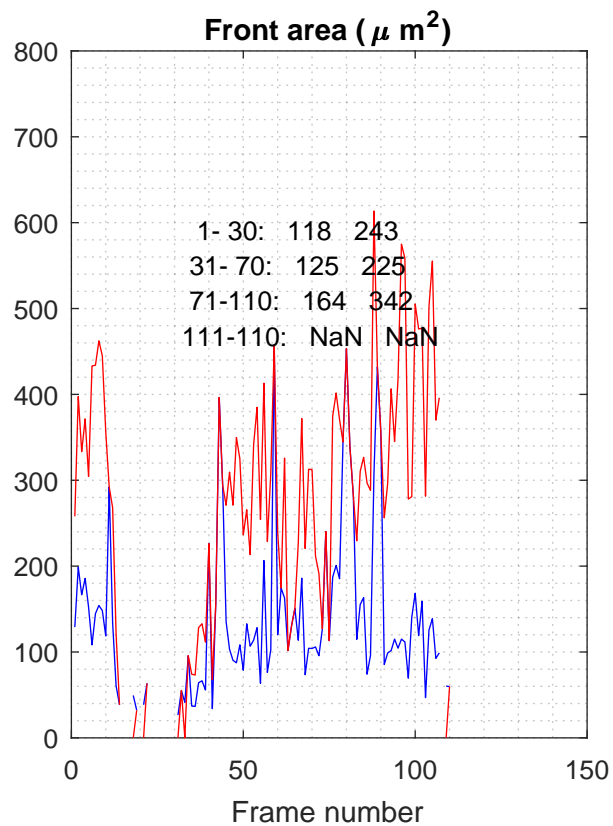

Supplement: Supplementary file 17 — Source Data for Appendix [file MSB-15-e8585-s025.zip › Source_data_for_Appendix/Appendix_Fig_S4/S4E/15.pdf]

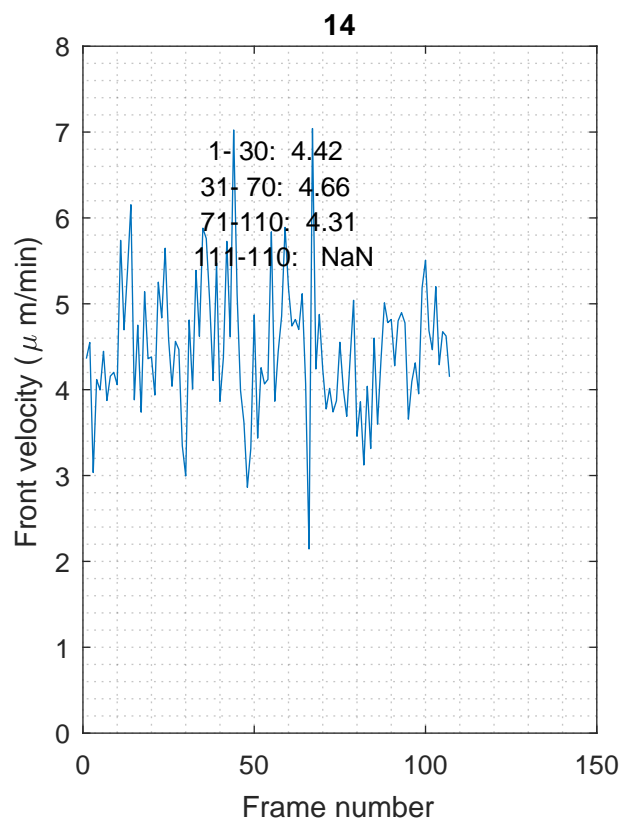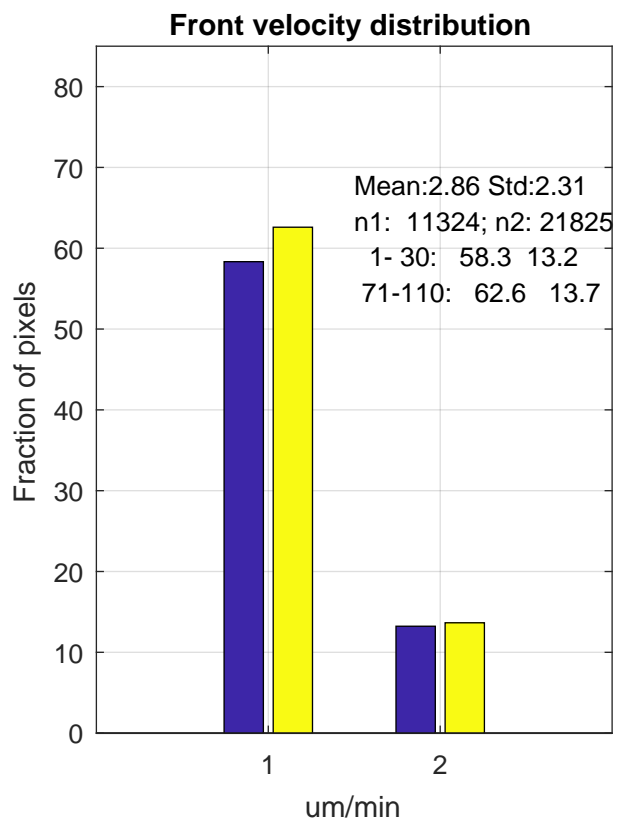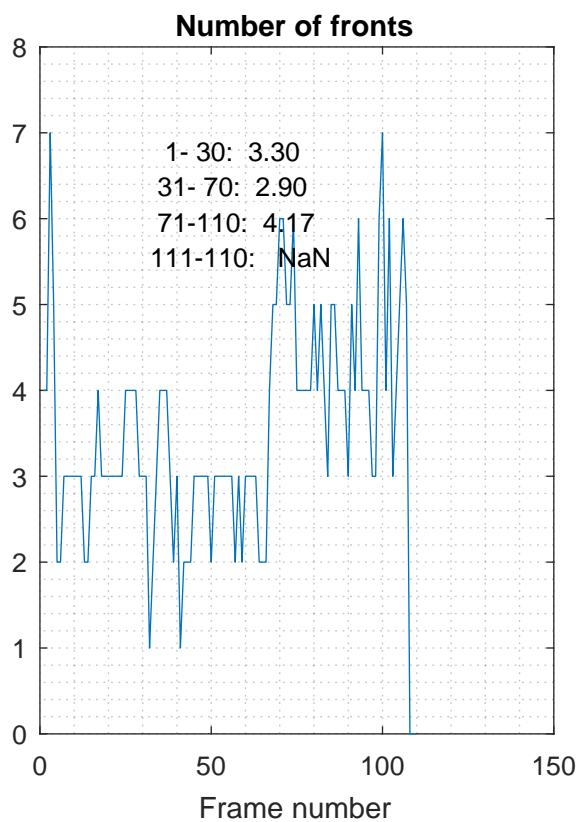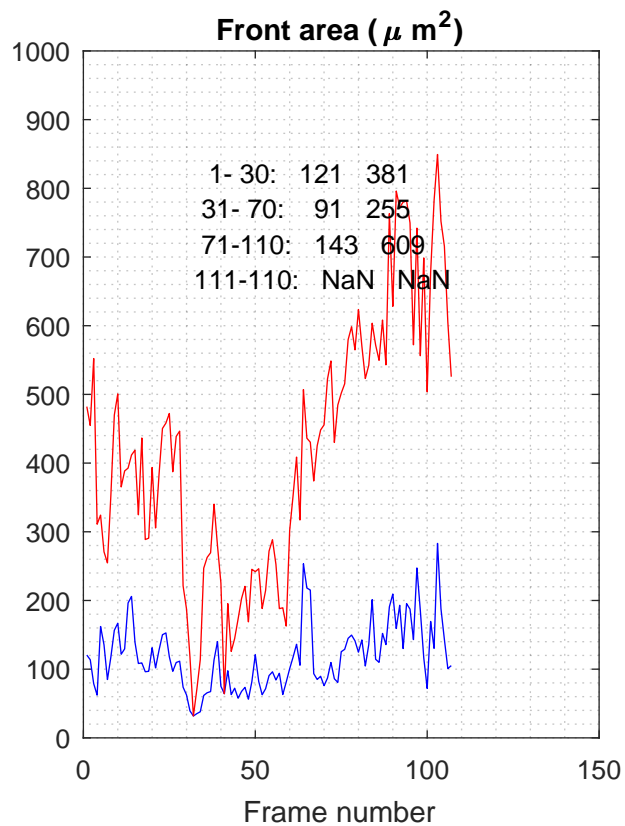

Supplement: Supplementary file 17 — Source Data for Appendix [file MSB-15-e8585-s025.zip › Source_data_for_Appendix/Appendix_Fig_S4/S4E/14.pdf]

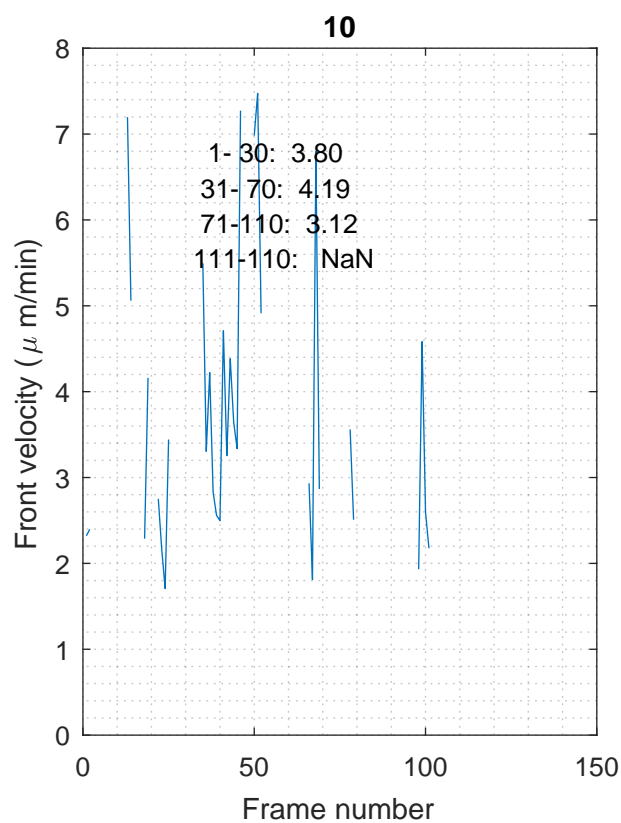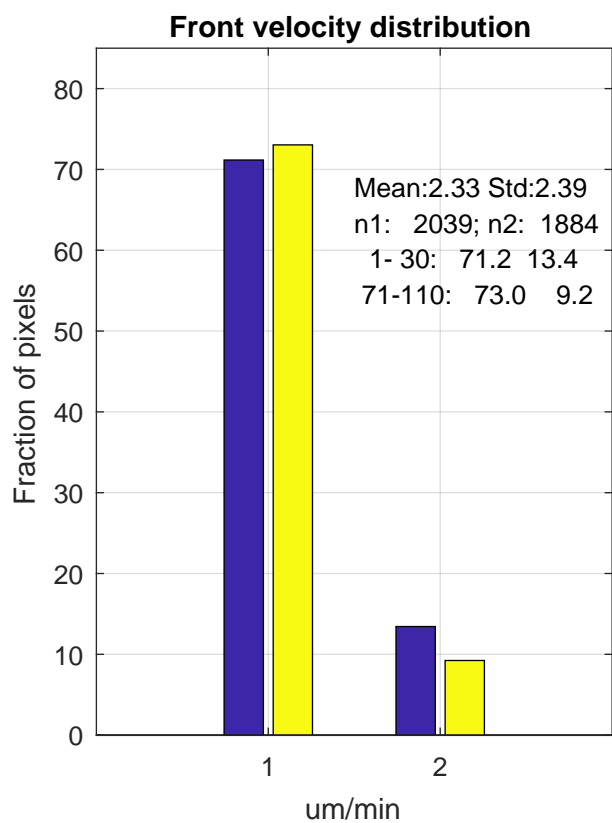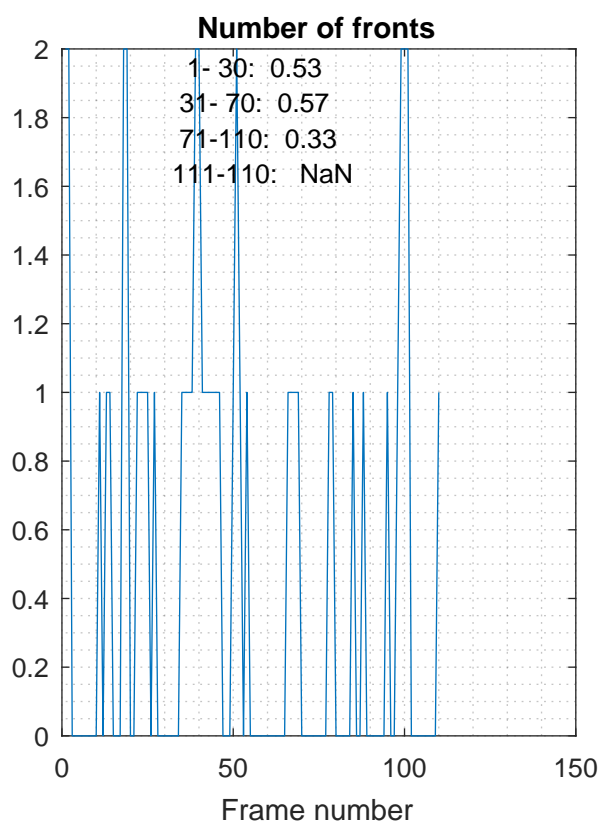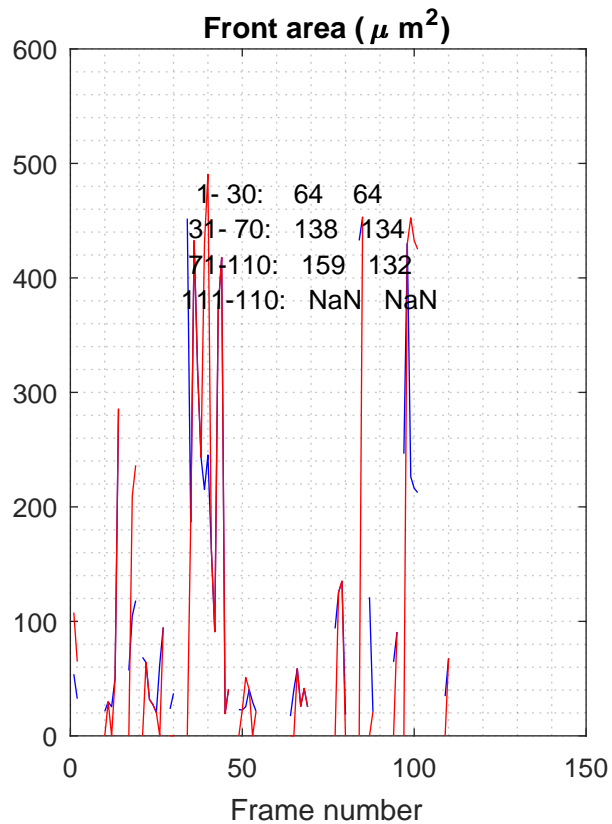

Supplement: Supplementary file 17 — Source Data for Appendix [file MSB-15-e8585-s025.zip › Source_data_for_Appendix/Appendix_Fig_S4/S4E/10.pdf]

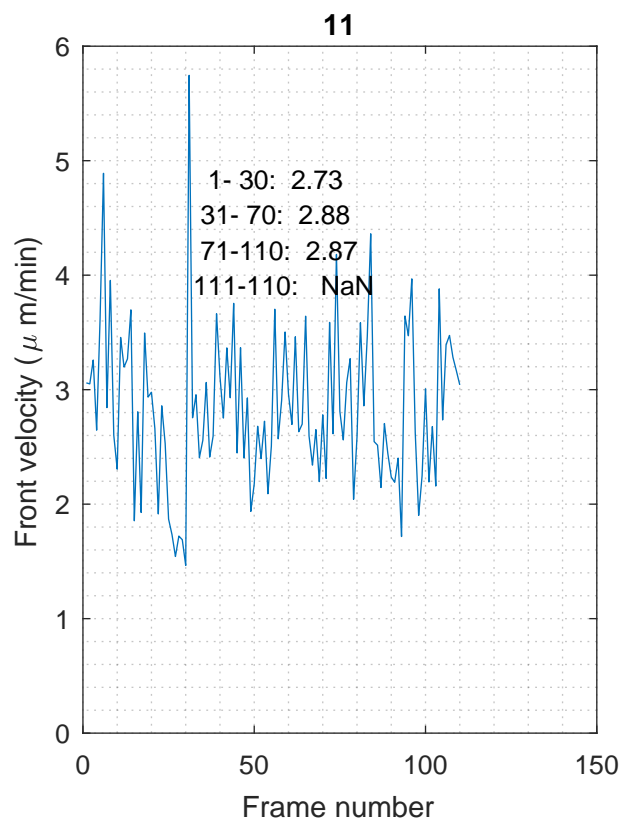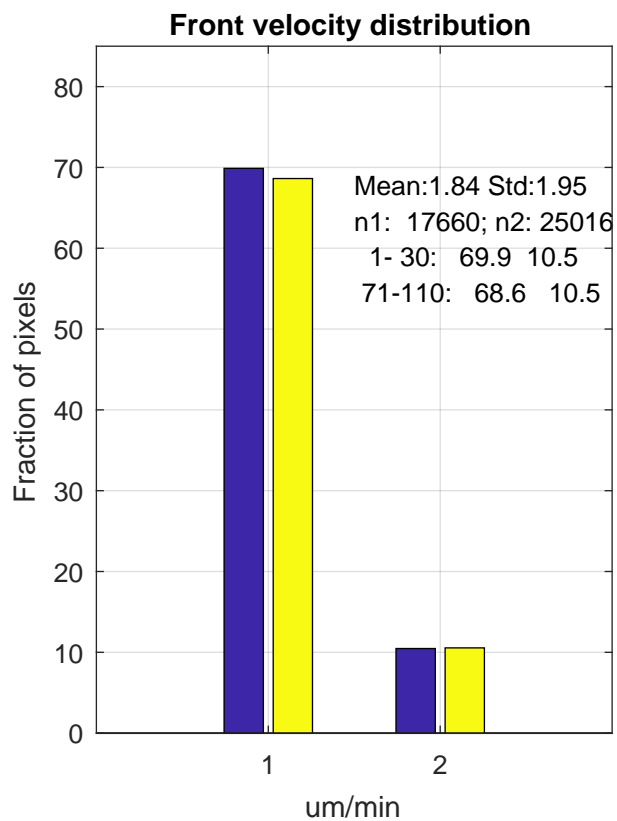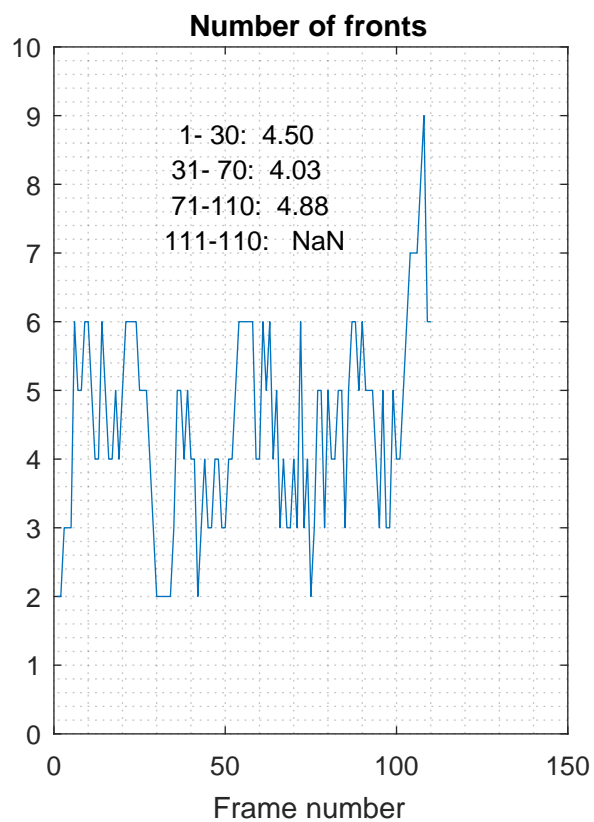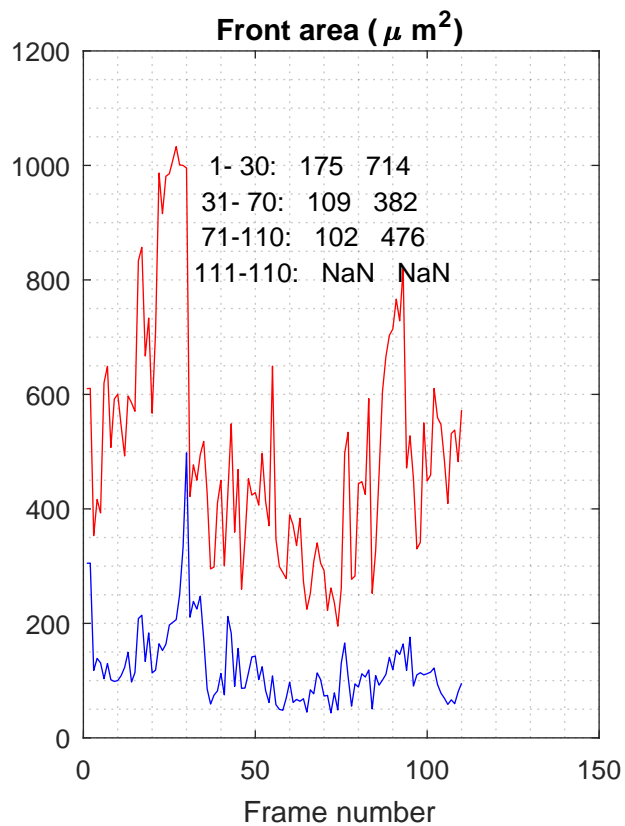

Supplement: Supplementary file 17 — Source Data for Appendix [file MSB-15-e8585-s025.zip › Source_data_for_Appendix/Appendix_Fig_S4/S4E/11.pdf]

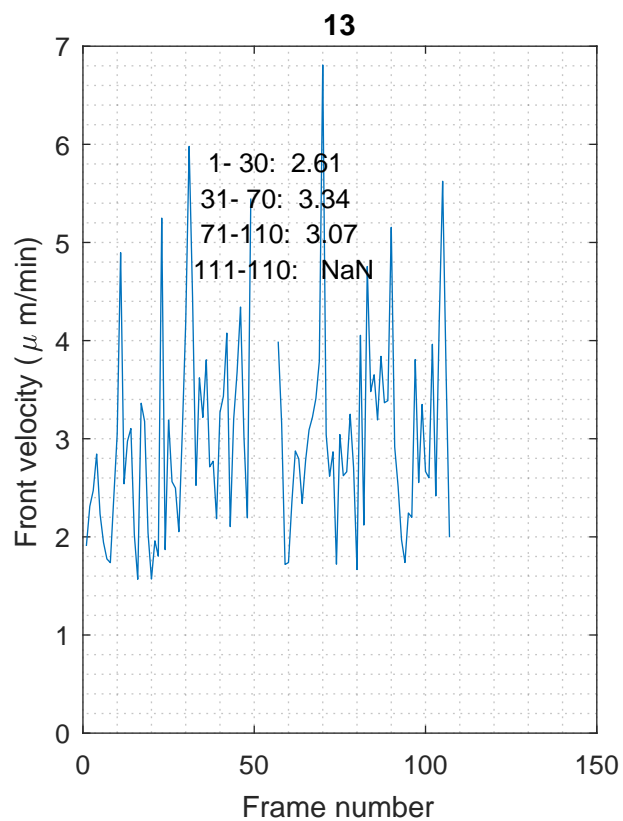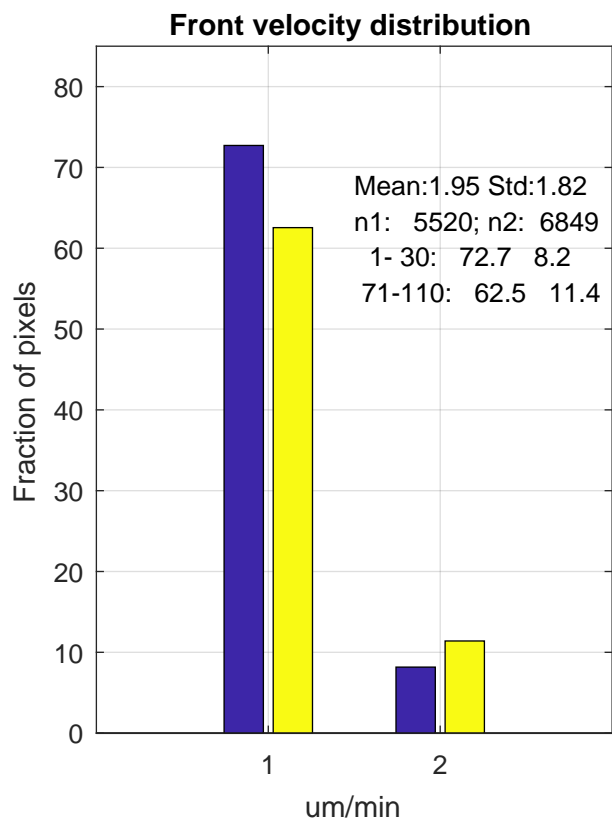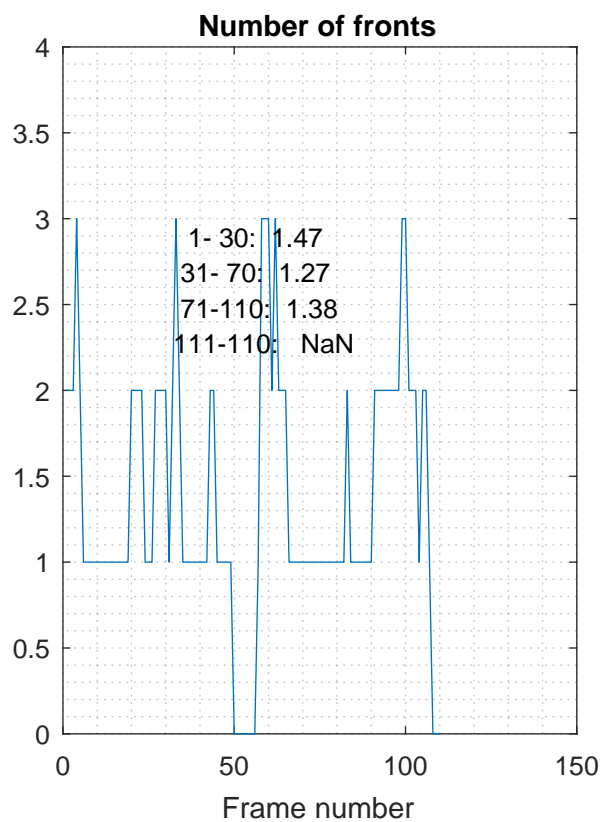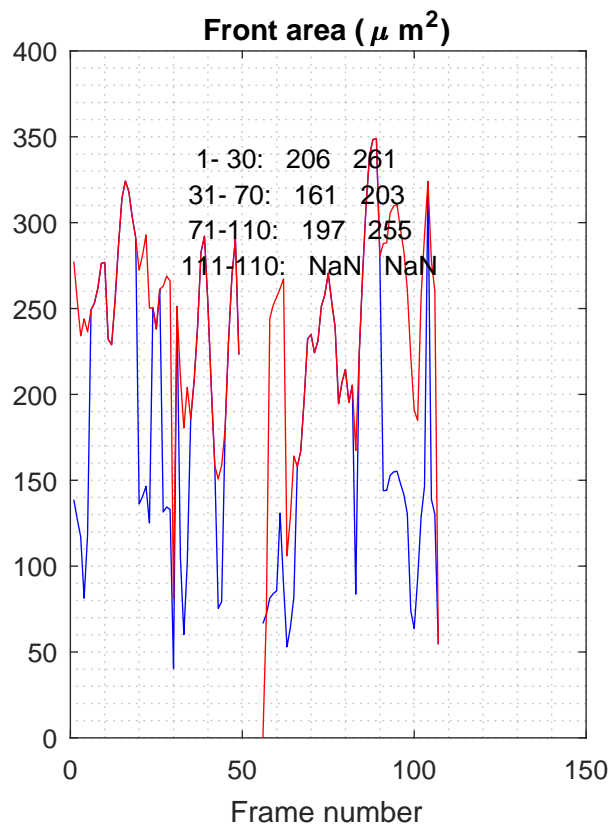

Supplement: Supplementary file 17 — Source Data for Appendix [file MSB-15-e8585-s025.zip › Source_data_for_Appendix/Appendix_Fig_S4/S4E/13.pdf]

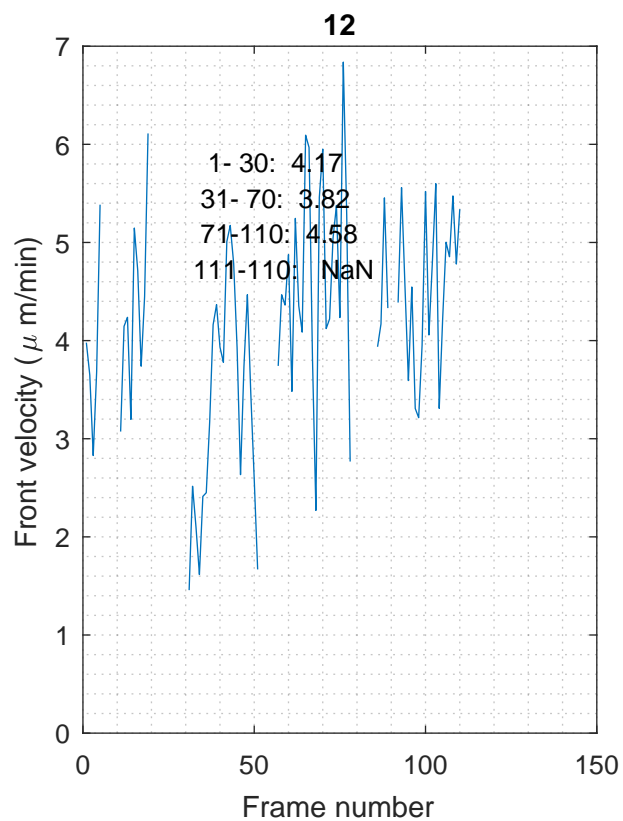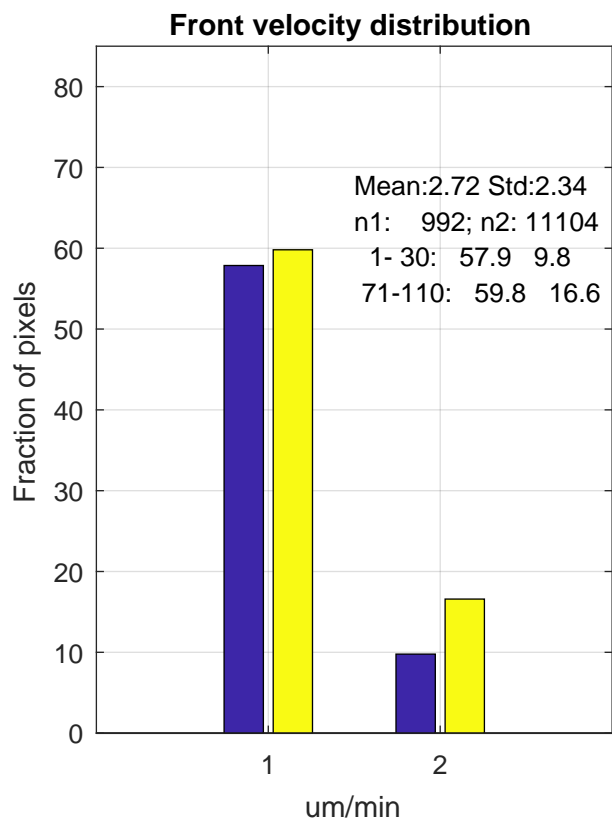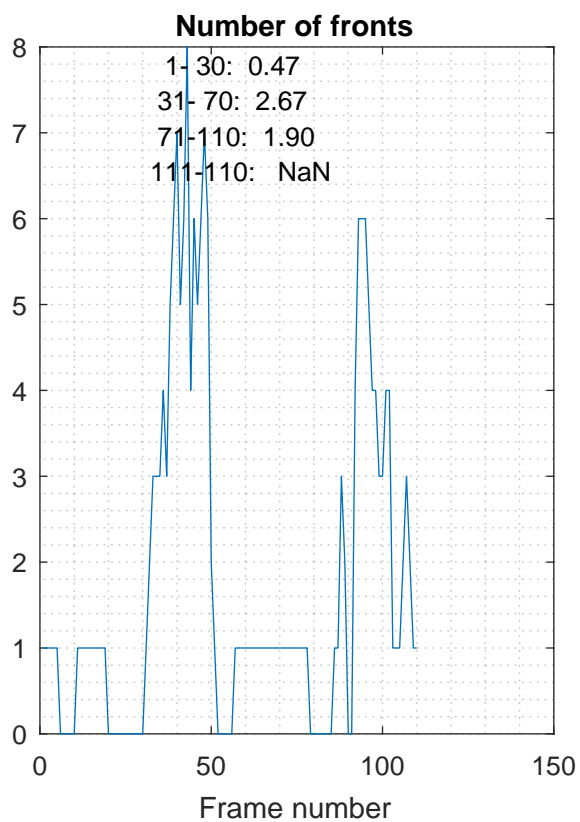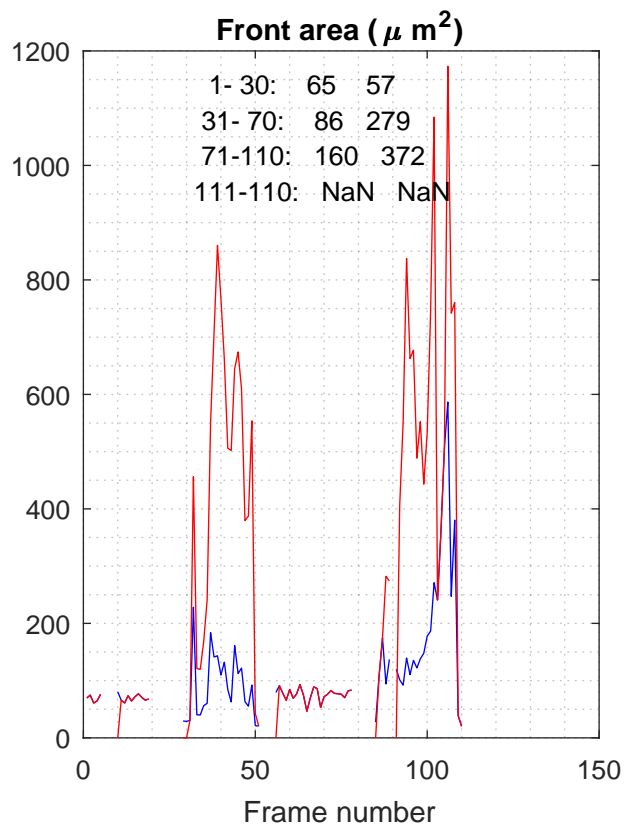

Supplement: Supplementary file 17 — Source Data for Appendix [file MSB-15-e8585-s025.zip › Source_data_for_Appendix/Appendix_Fig_S4/S4E/12.pdf]

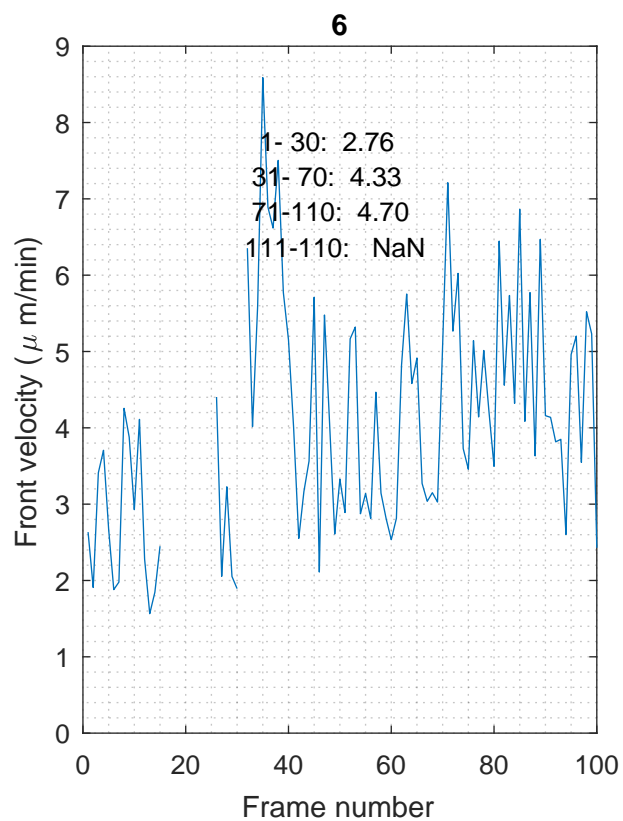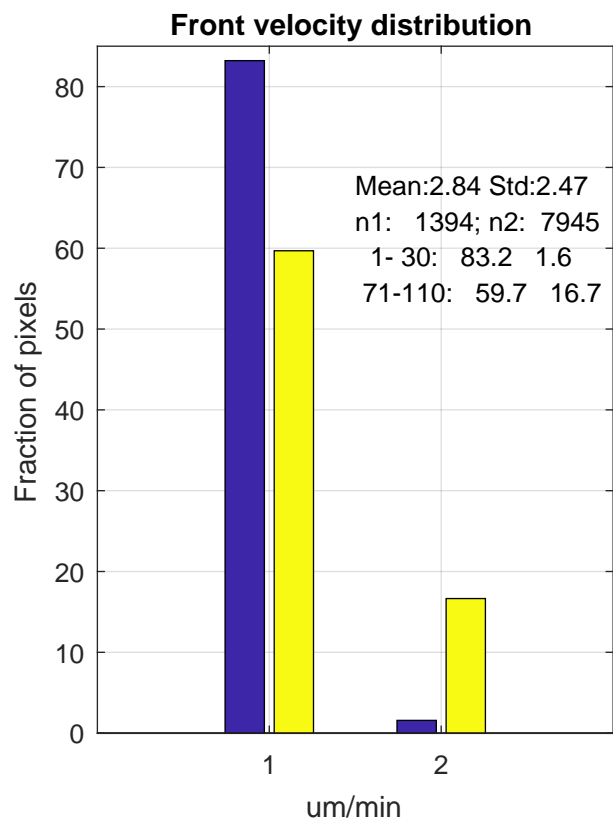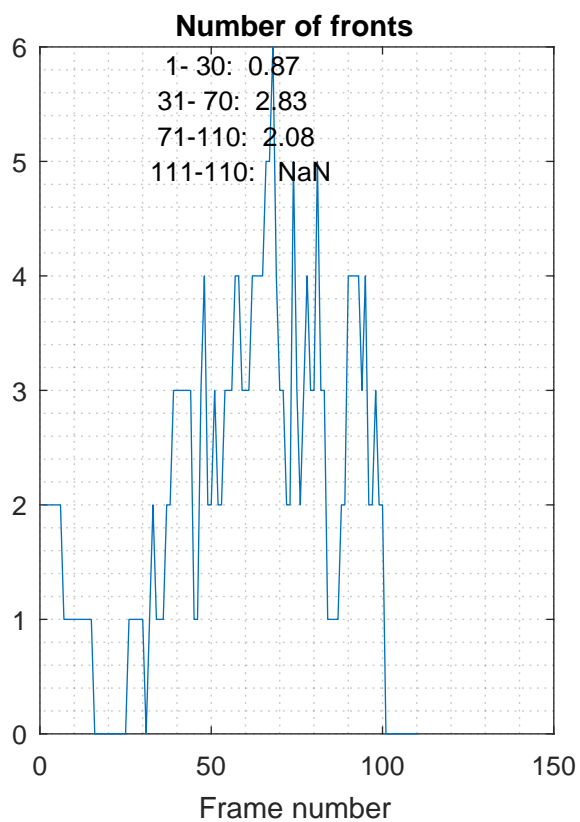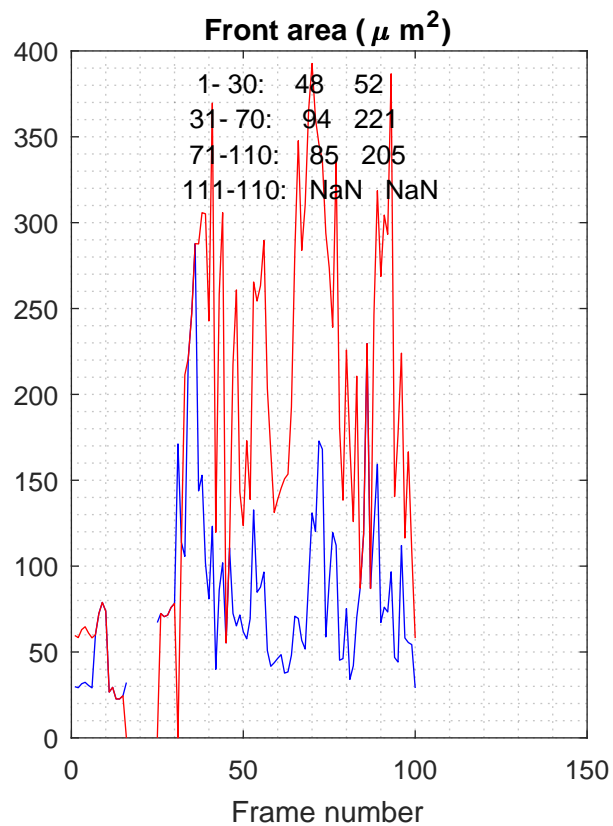

Supplement: Supplementary file 17 — Source Data for Appendix [file MSB-15-e8585-s025.zip › Source_data_for_Appendix/Appendix_Fig_S4/S4E/6.pdf]

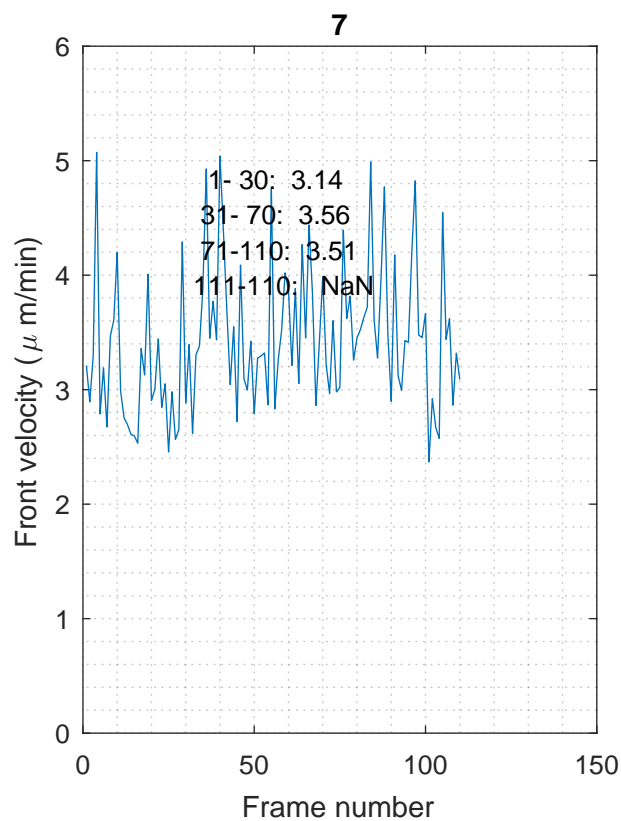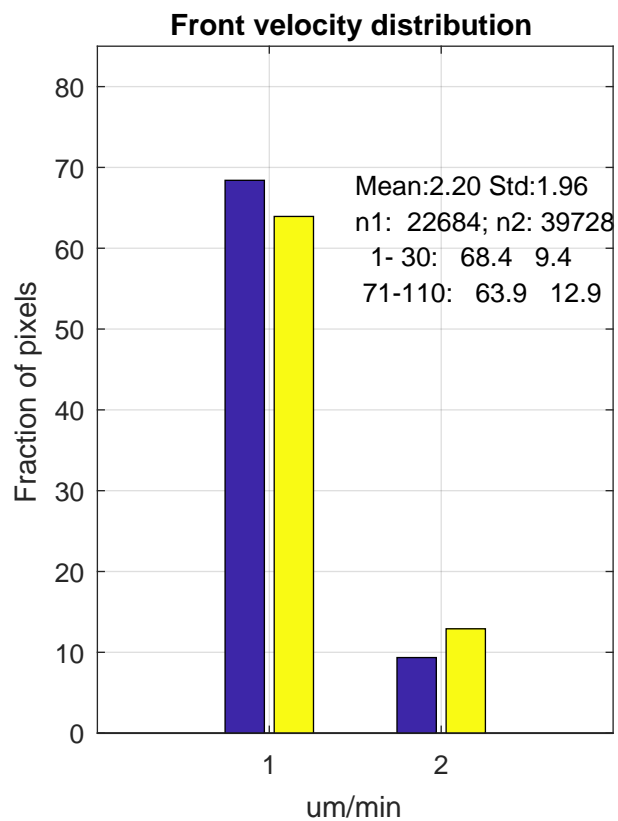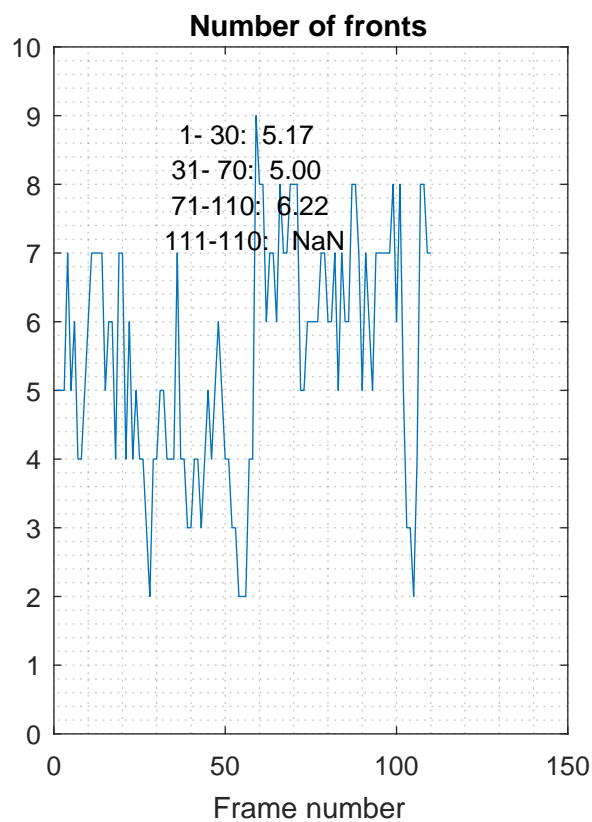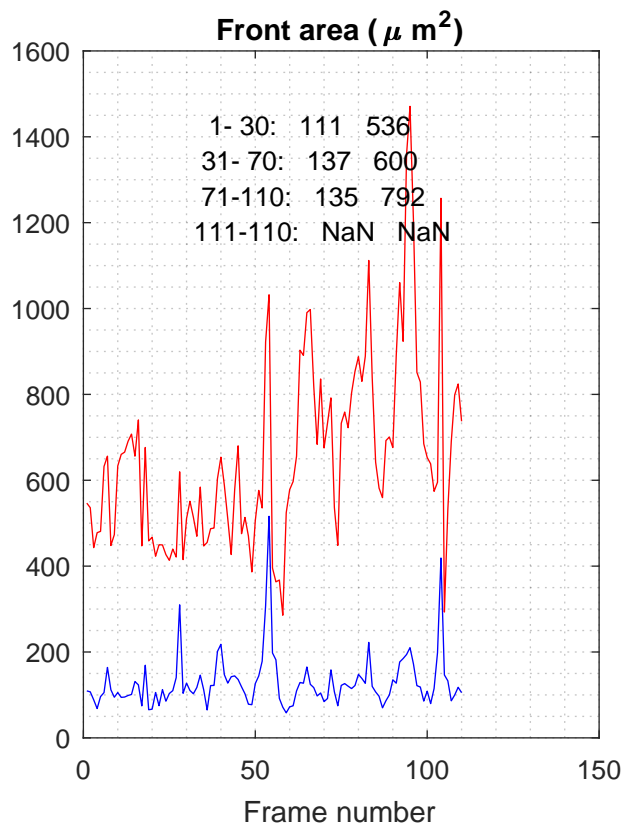

Supplement: Supplementary file 17 — Source Data for Appendix [file MSB-15-e8585-s025.zip › Source_data_for_Appendix/Appendix_Fig_S4/S4E/7.pdf]

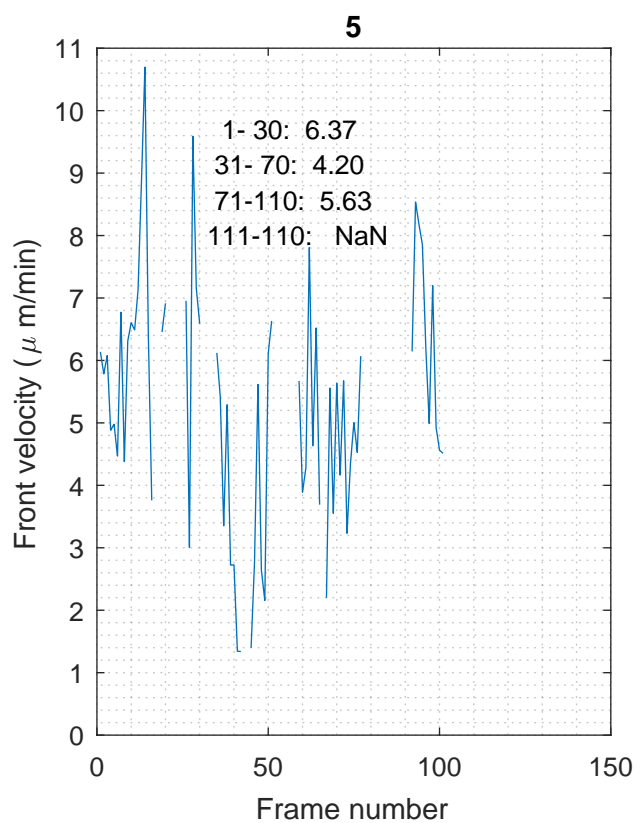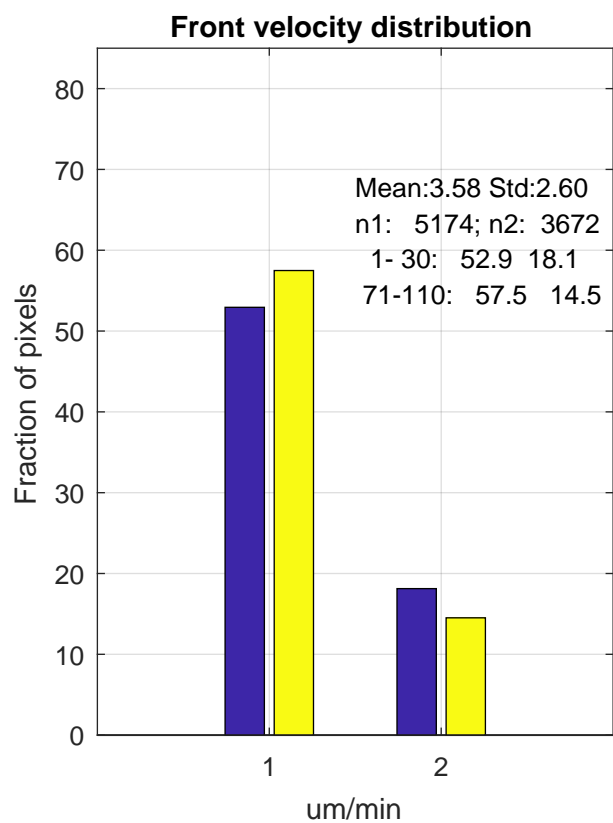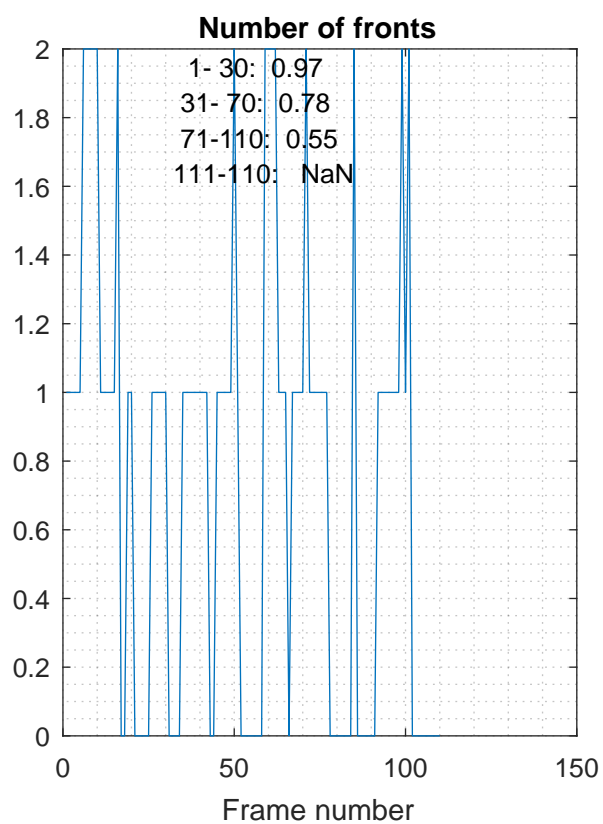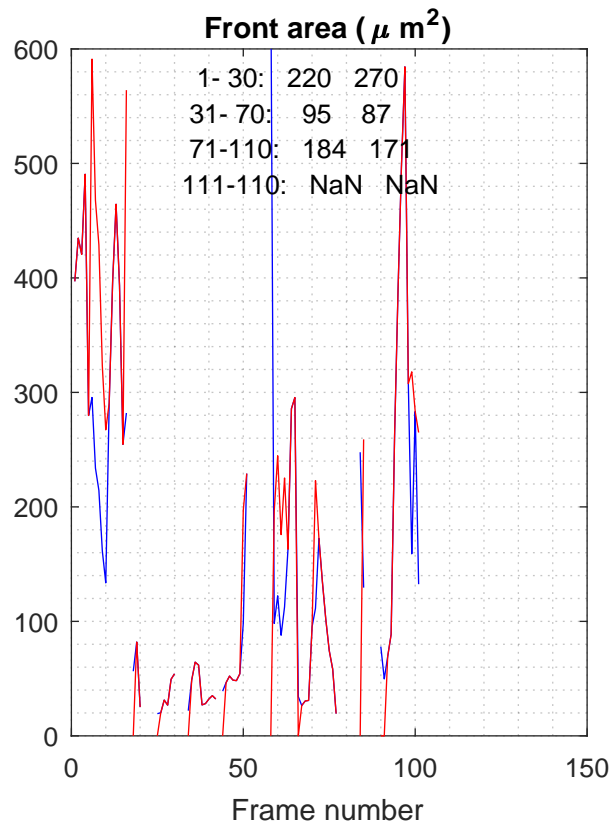

Supplement: Supplementary file 17 — Source Data for Appendix [file MSB-15-e8585-s025.zip › Source_data_for_Appendix/Appendix_Fig_S4/S4E/5.pdf]

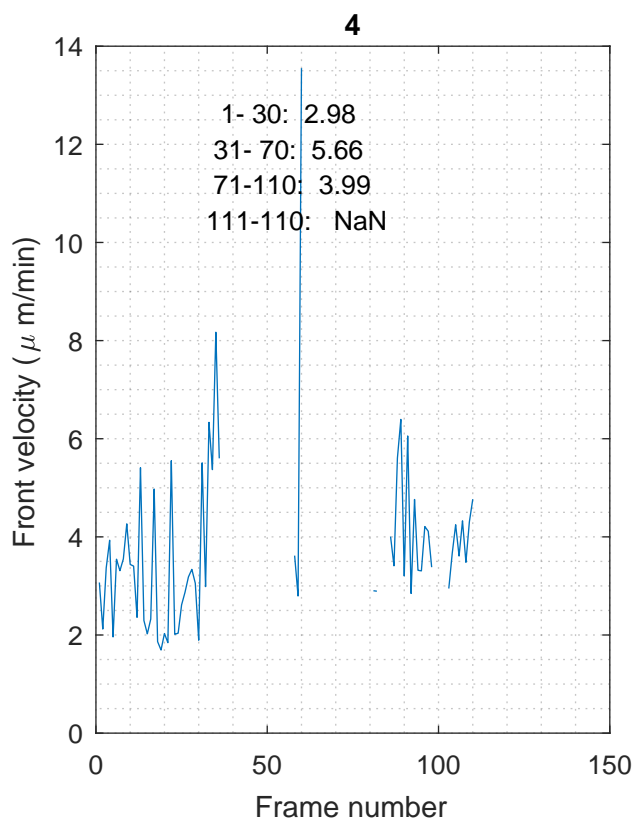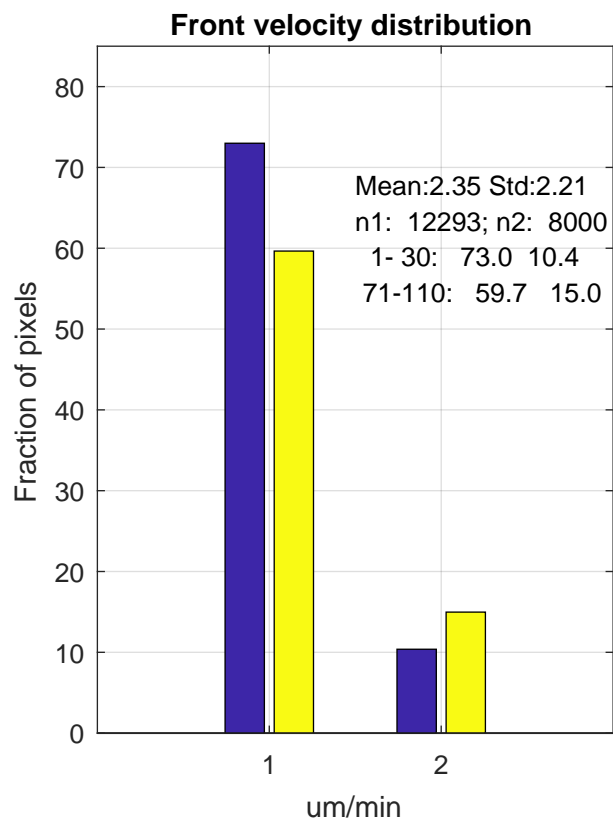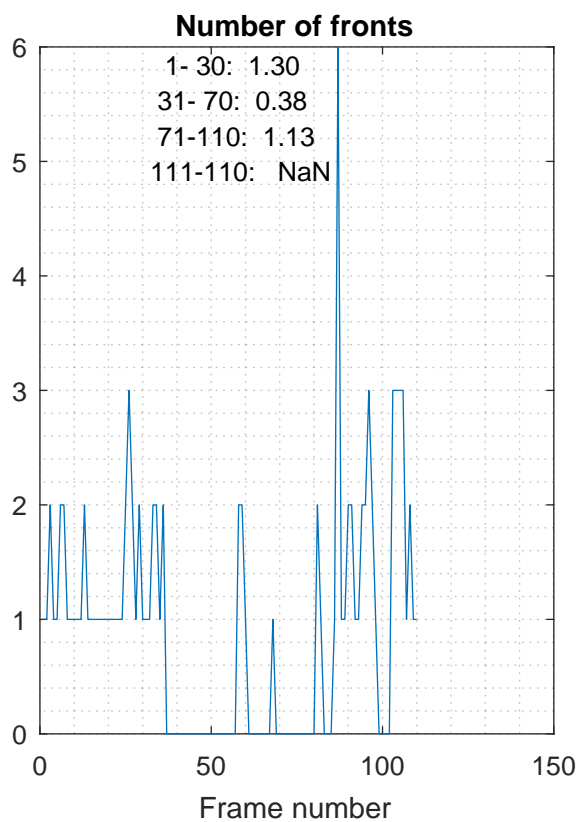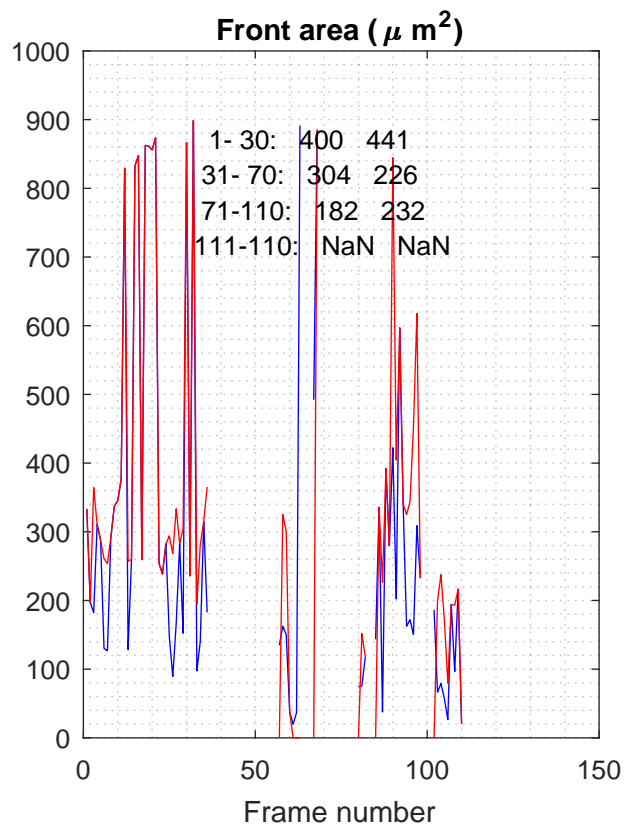

Supplement: Supplementary file 17 — Source Data for Appendix [file MSB-15-e8585-s025.zip › Source_data_for_Appendix/Appendix_Fig_S4/S4E/4.pdf]

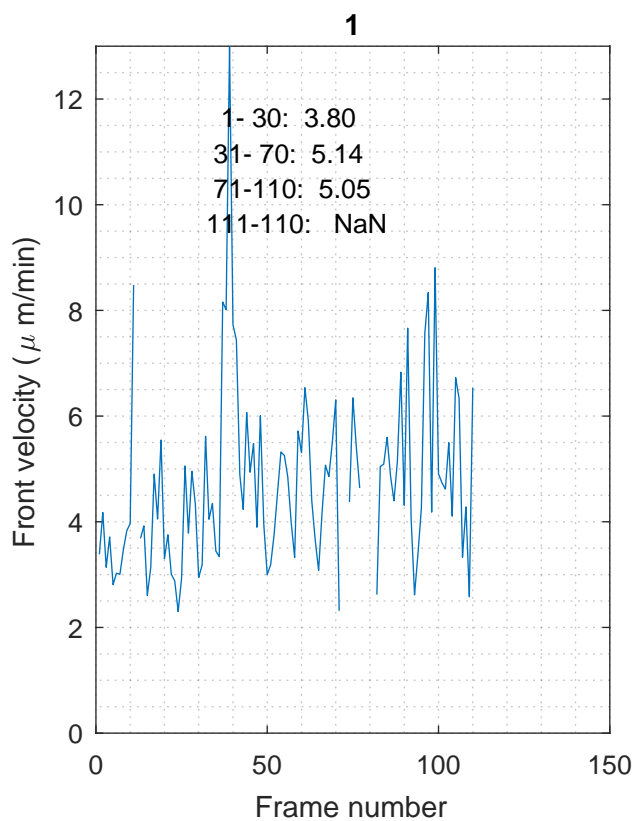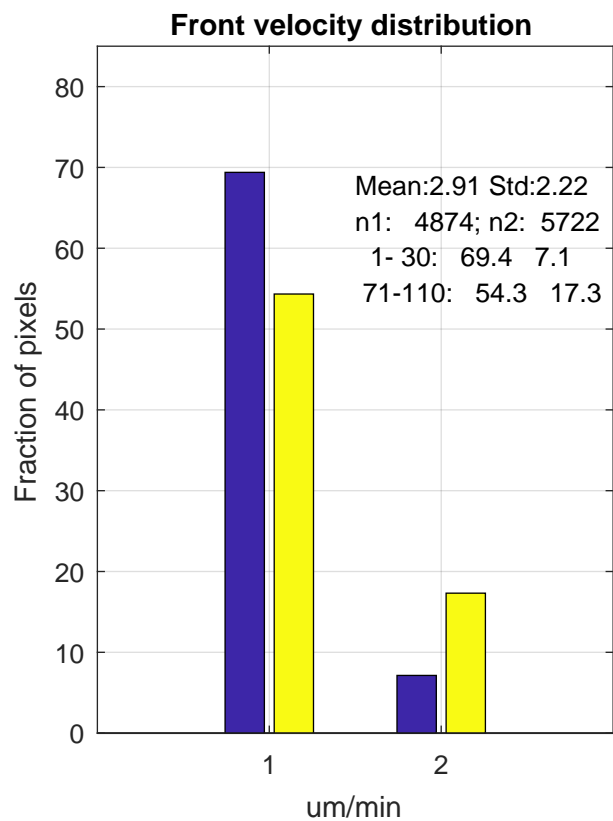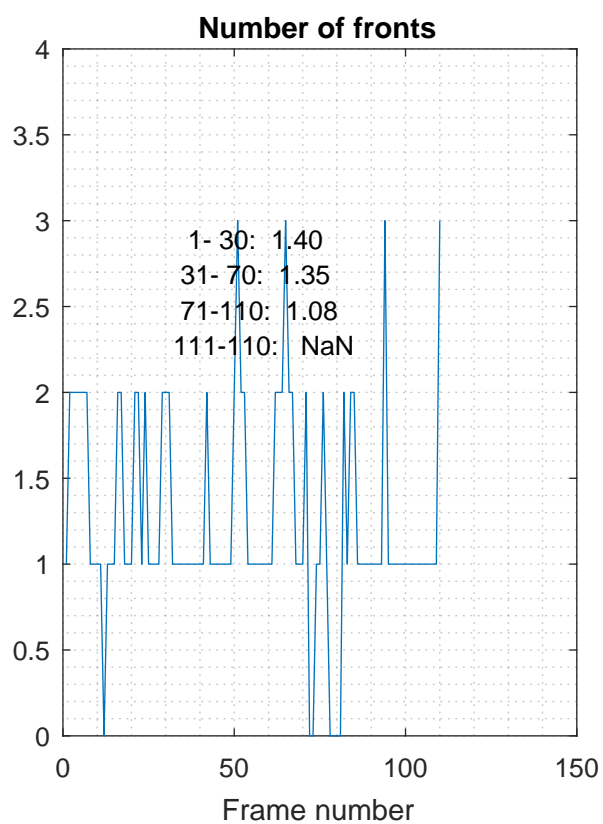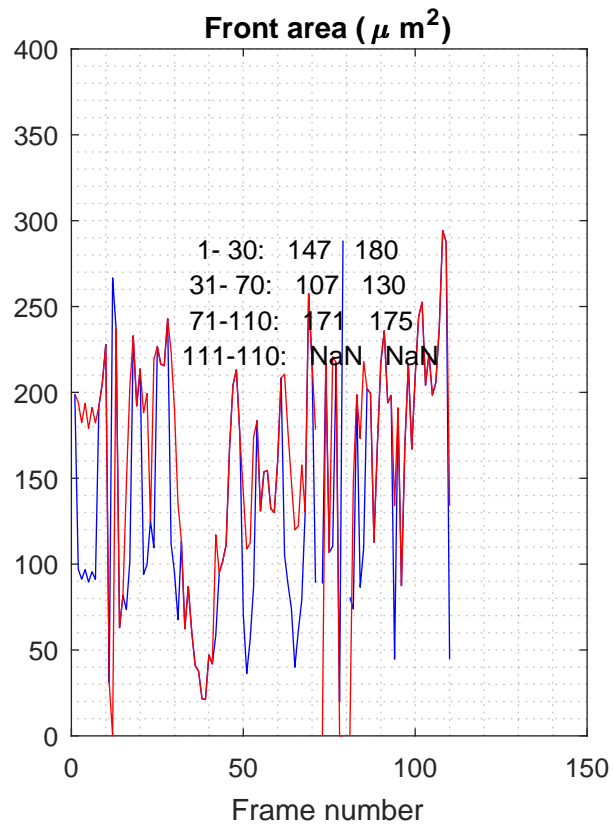

Supplement: Supplementary file 17 — Source Data for Appendix [file MSB-15-e8585-s025.zip › Source_data_for_Appendix/Appendix_Fig_S4/S4E/1.pdf]

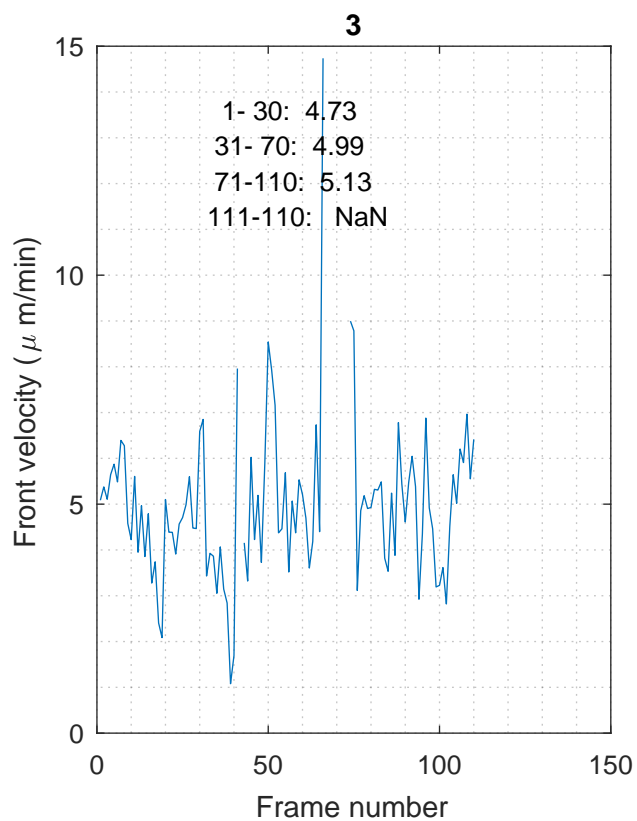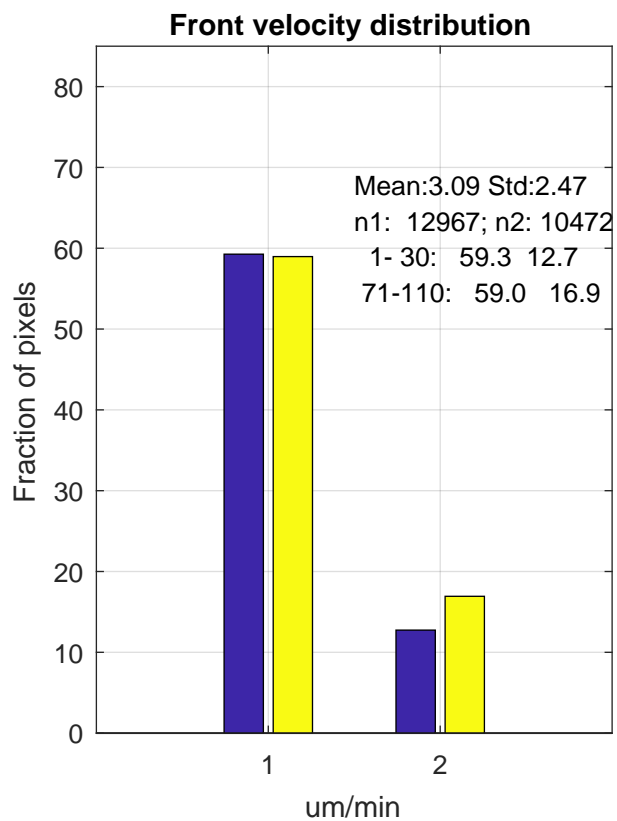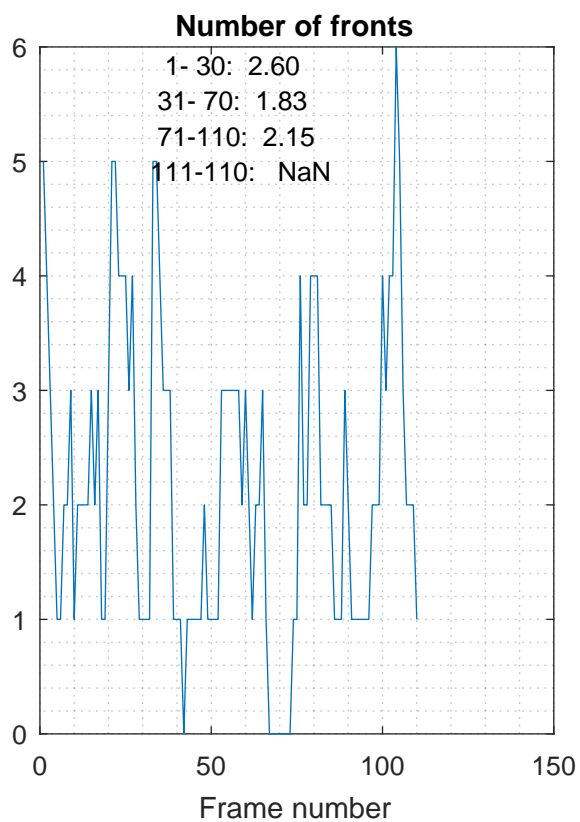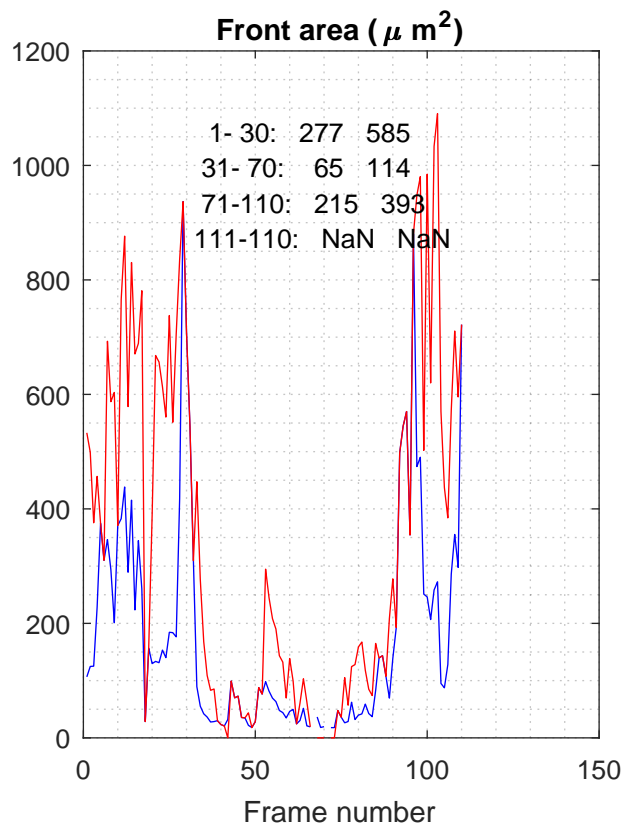

Supplement: Supplementary file 17 — Source Data for Appendix [file MSB-15-e8585-s025.zip › Source_data_for_Appendix/Appendix_Fig_S4/S4E/3.pdf]

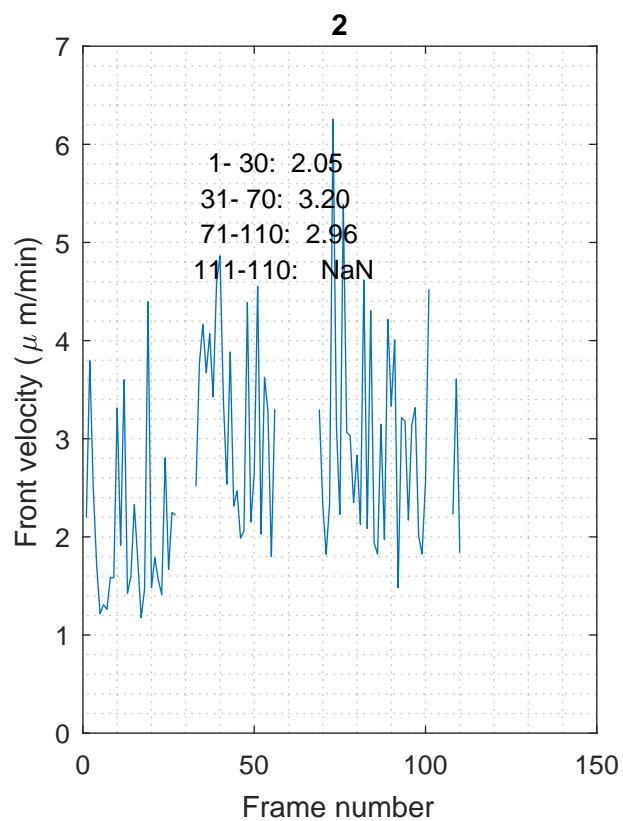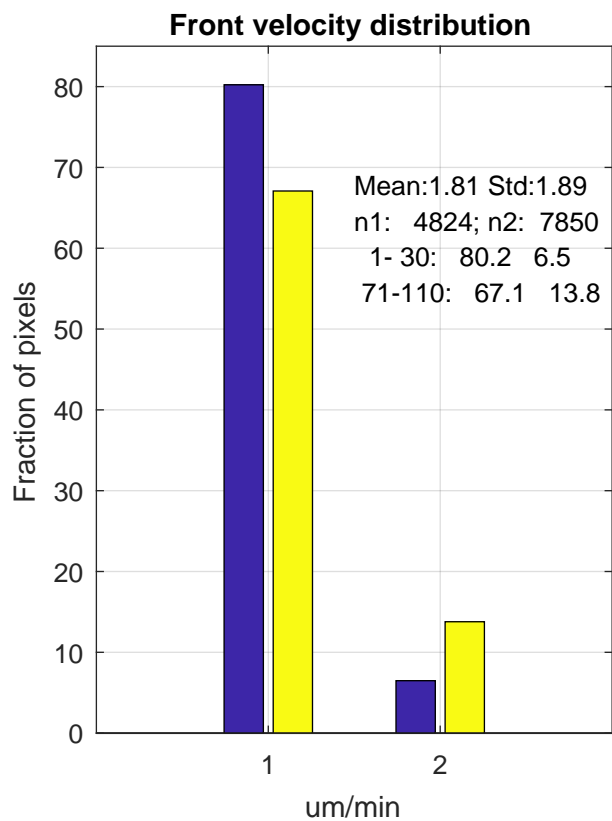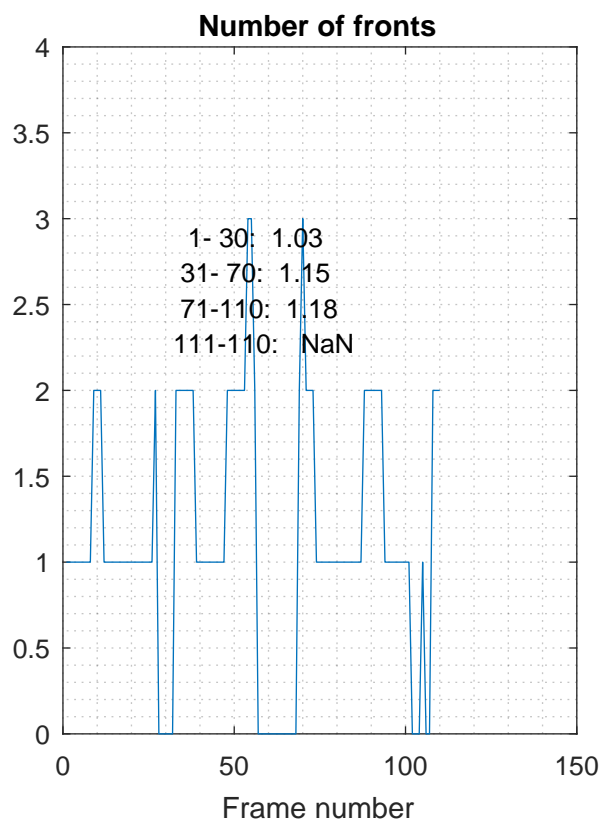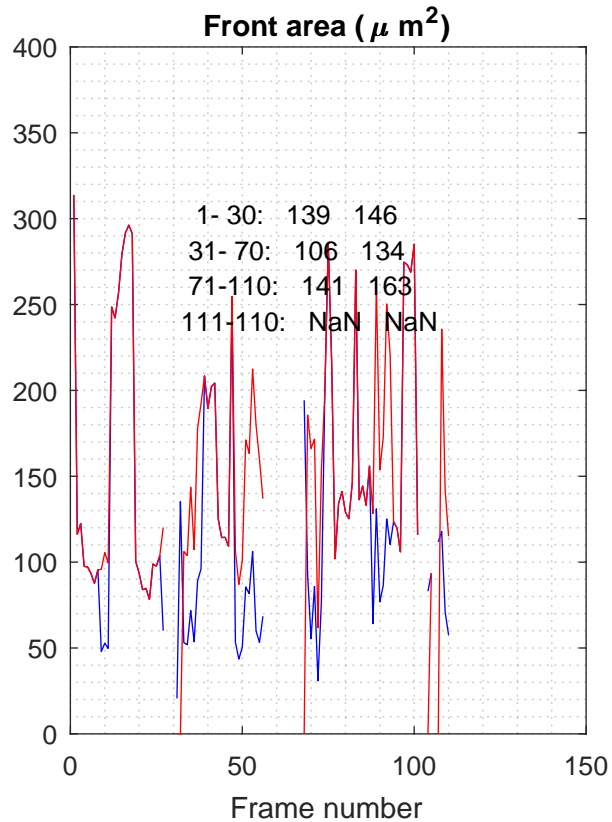

Supplement: Supplementary file 17 — Source Data for Appendix [file MSB-15-e8585-s025.zip › Source_data_for_Appendix/Appendix_Fig_S4/S4E/2.pdf]

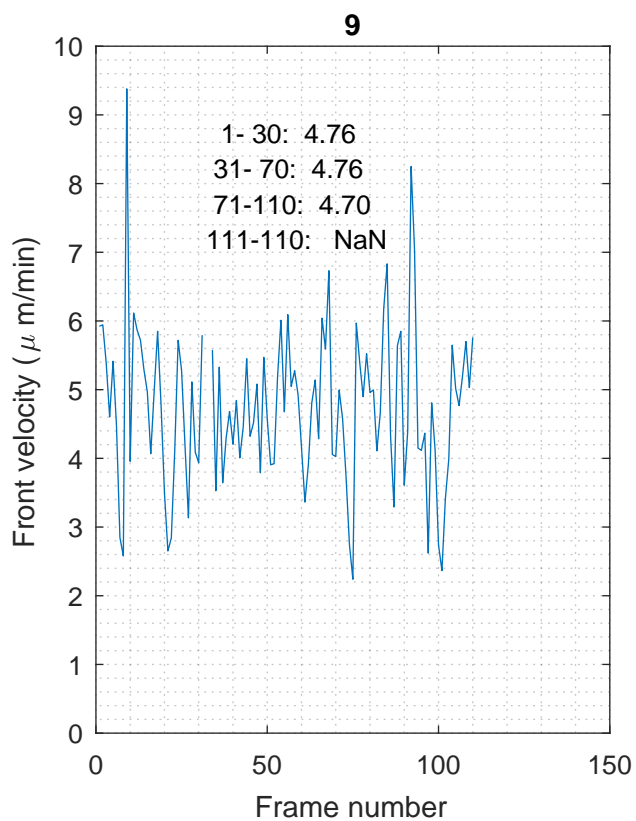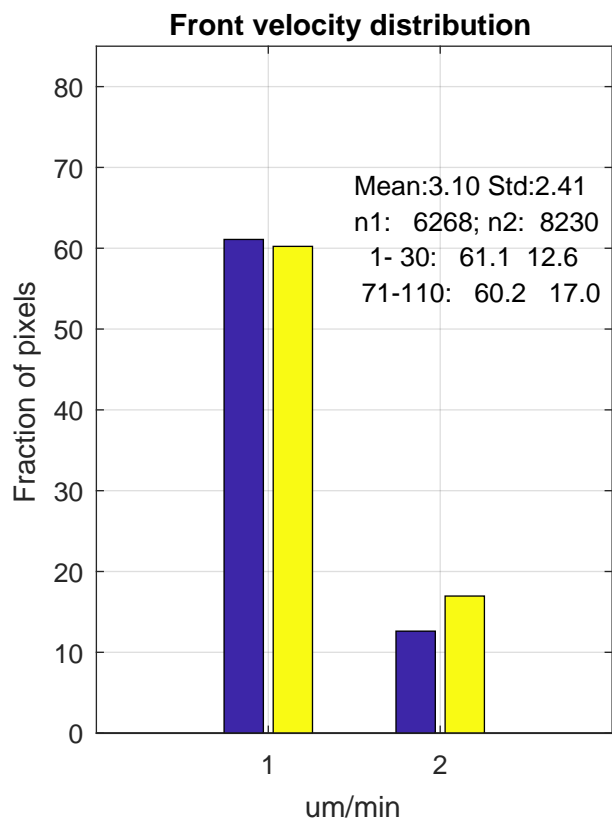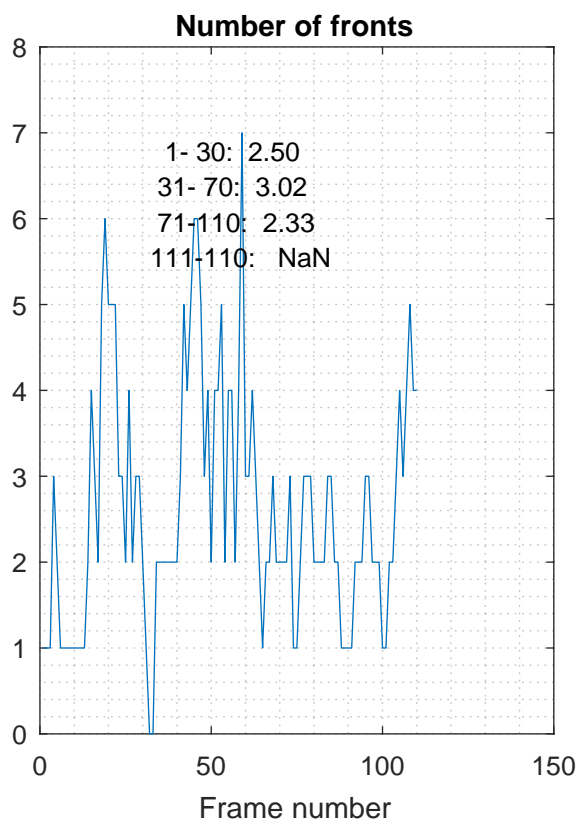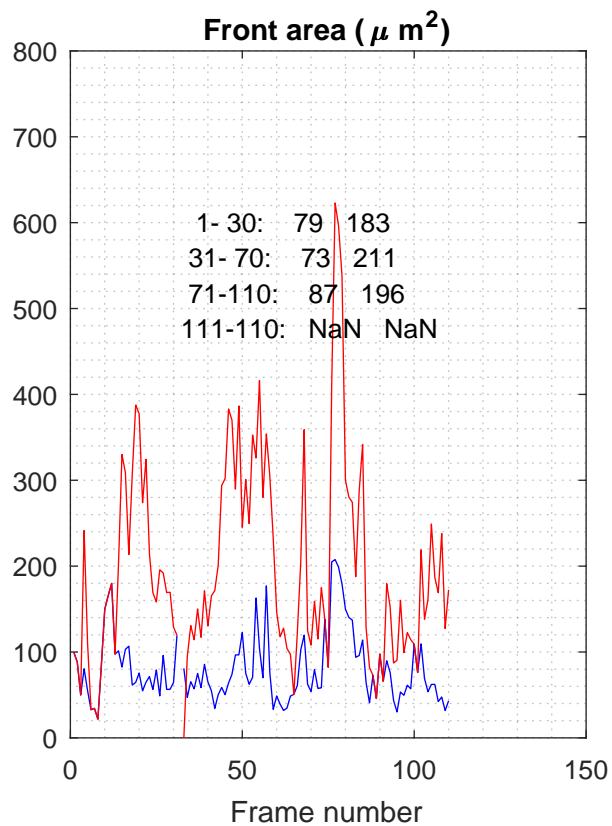

Supplement: Supplementary file 17 — Source Data for Appendix [file MSB-15-e8585-s025.zip › Source_data_for_Appendix/Appendix_Fig_S4/S4F/9.pdf]

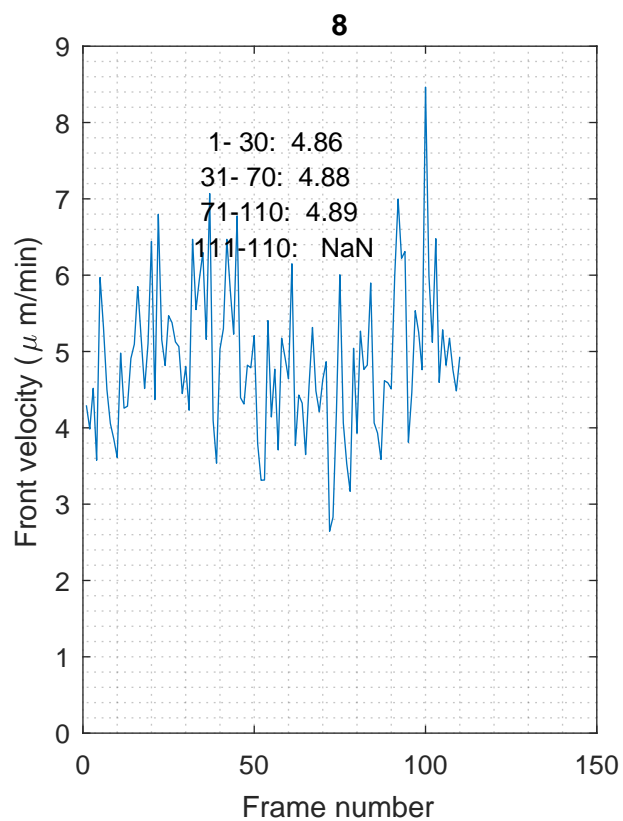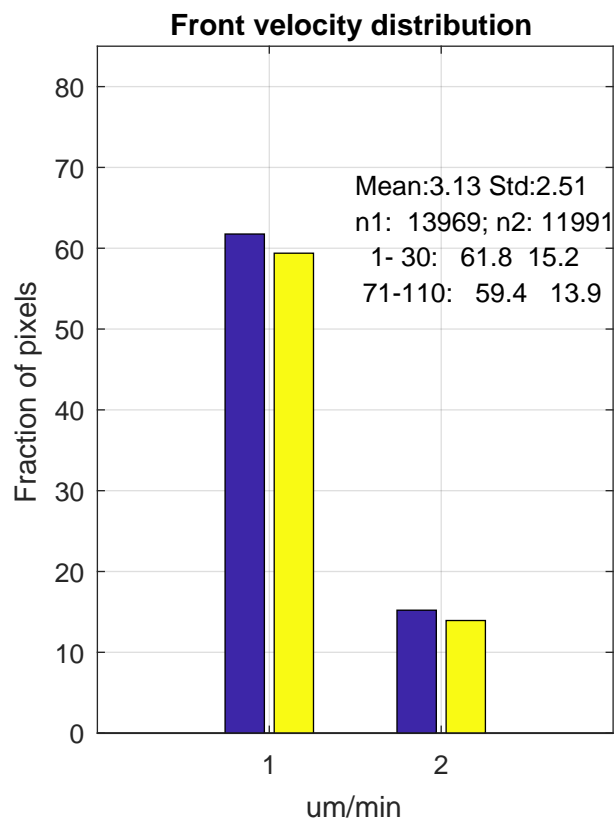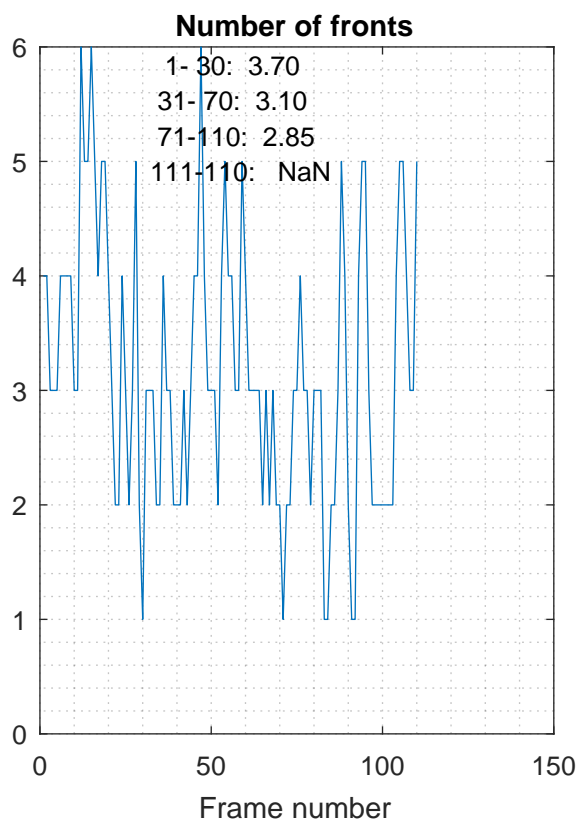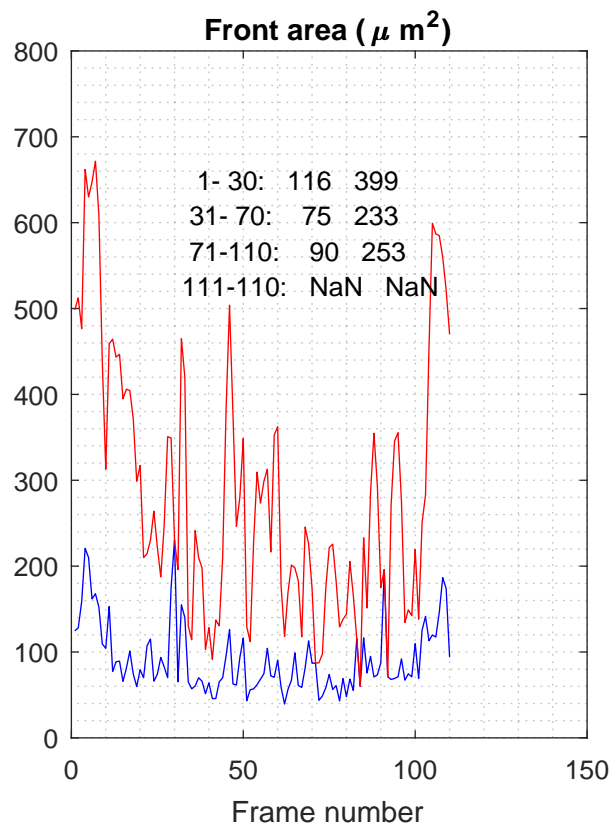

Supplement: Supplementary file 17 — Source Data for Appendix [file MSB-15-e8585-s025.zip › Source_data_for_Appendix/Appendix_Fig_S4/S4F/8.pdf]

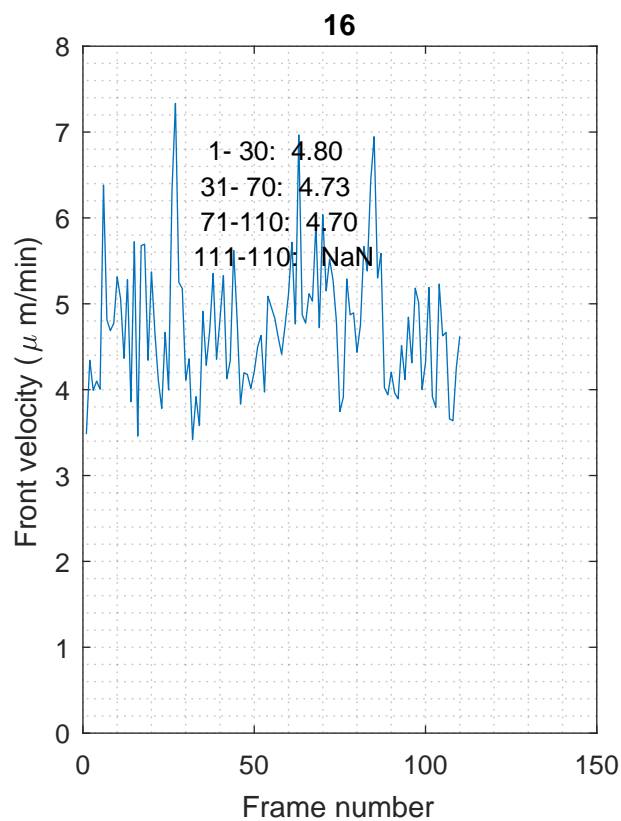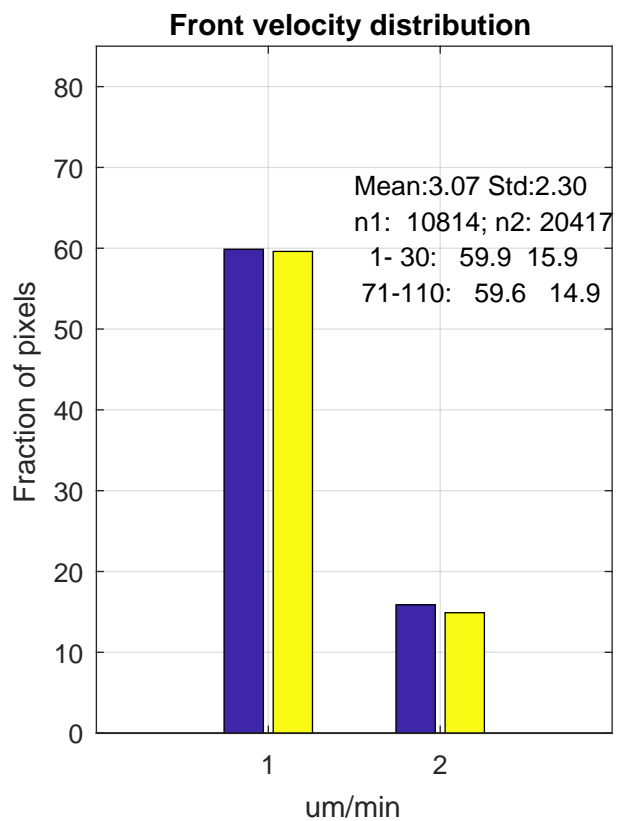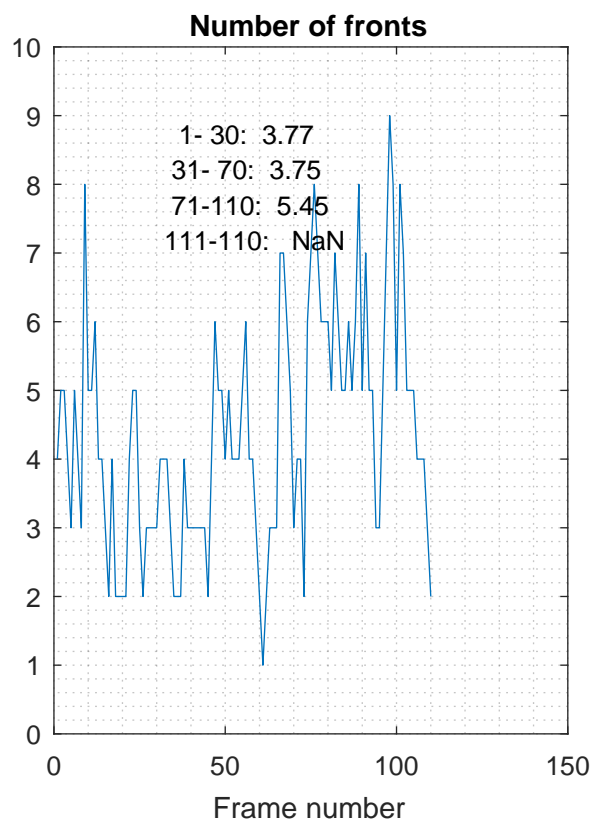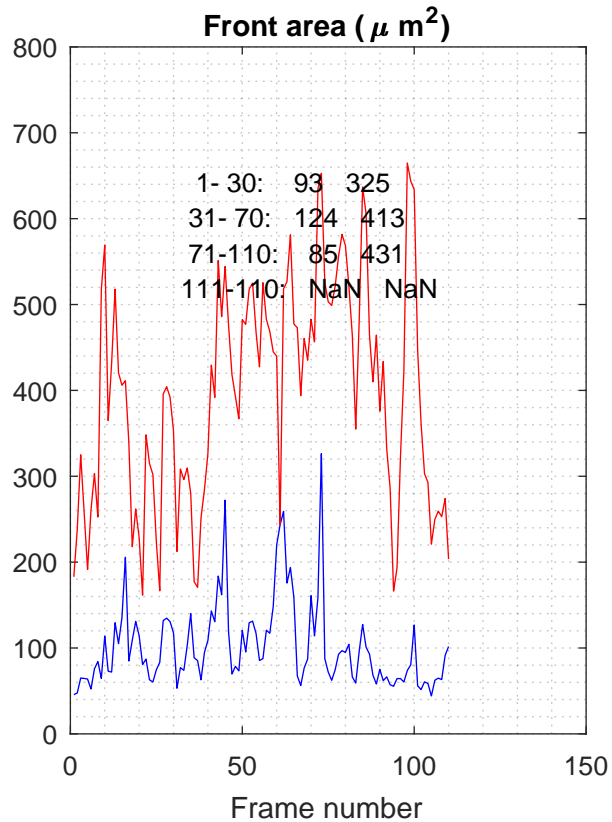

Supplement: Supplementary file 17 — Source Data for Appendix [file MSB-15-e8585-s025.zip › Source_data_for_Appendix/Appendix_Fig_S4/S4F/16.pdf]

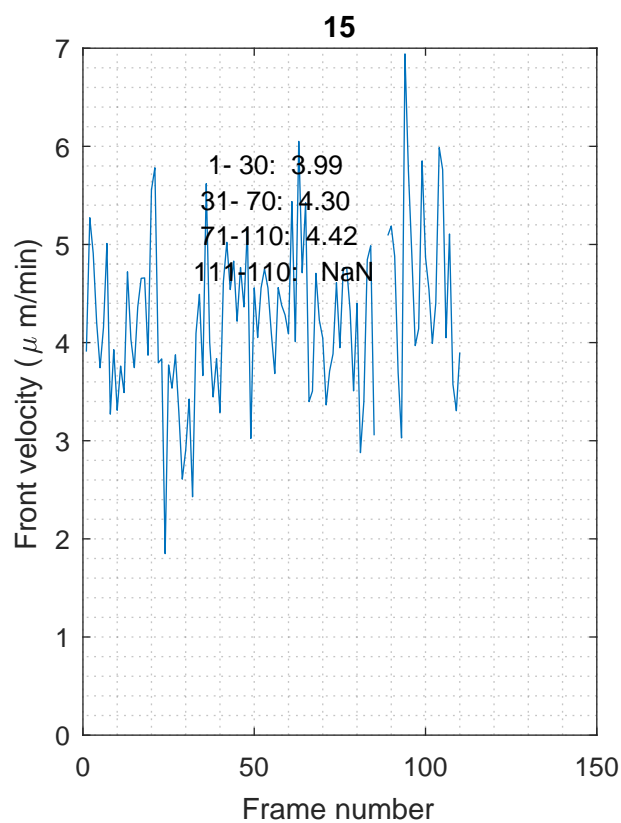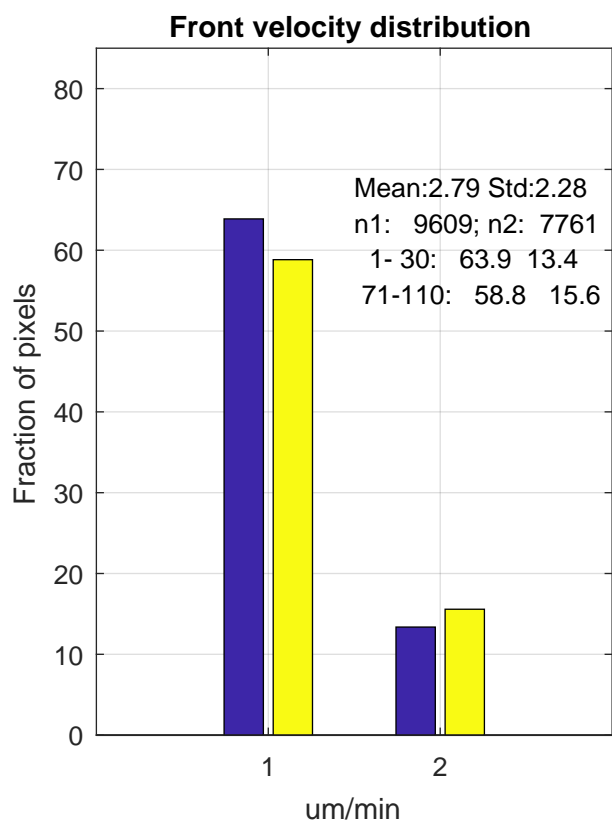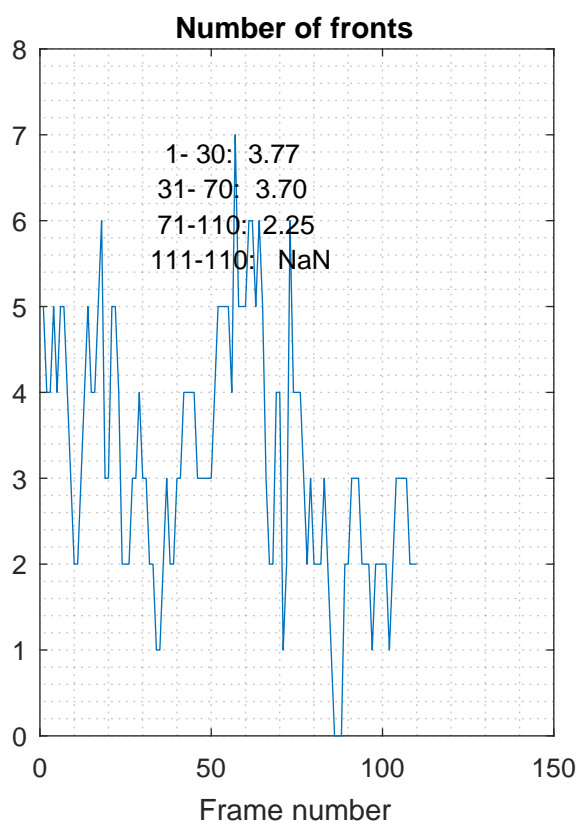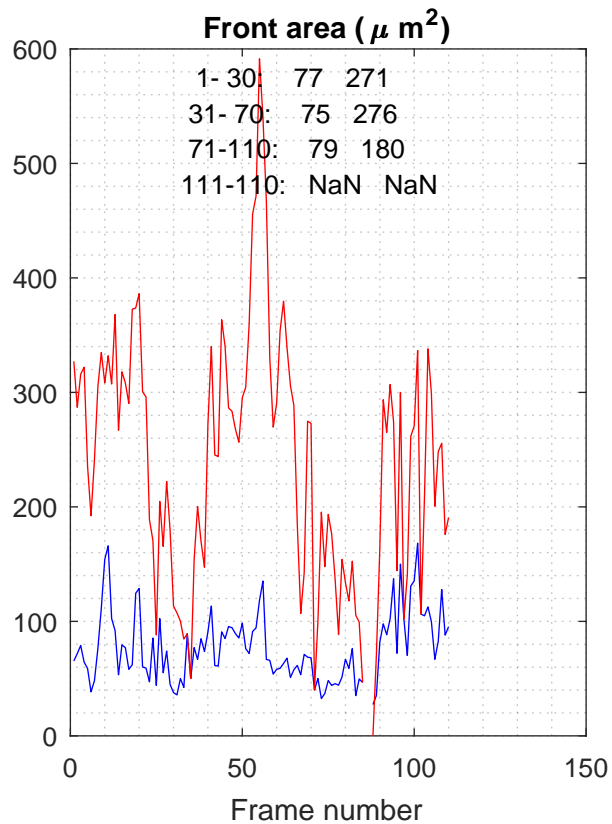

Supplement: Supplementary file 17 — Source Data for Appendix [file MSB-15-e8585-s025.zip › Source_data_for_Appendix/Appendix_Fig_S4/S4F/15.pdf]

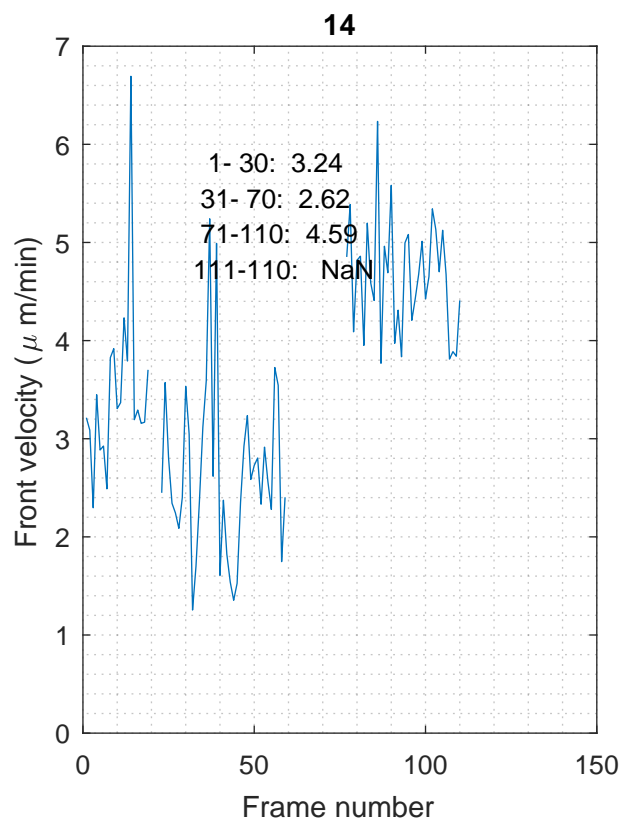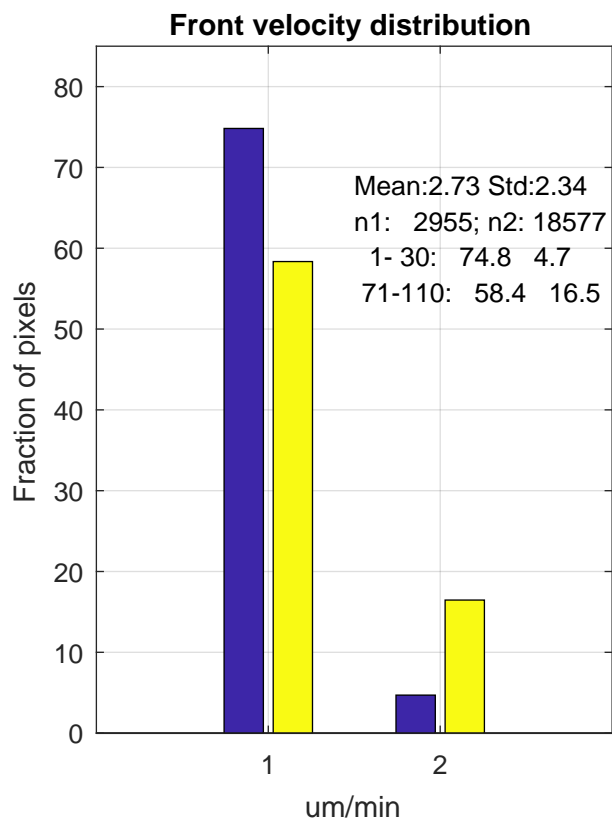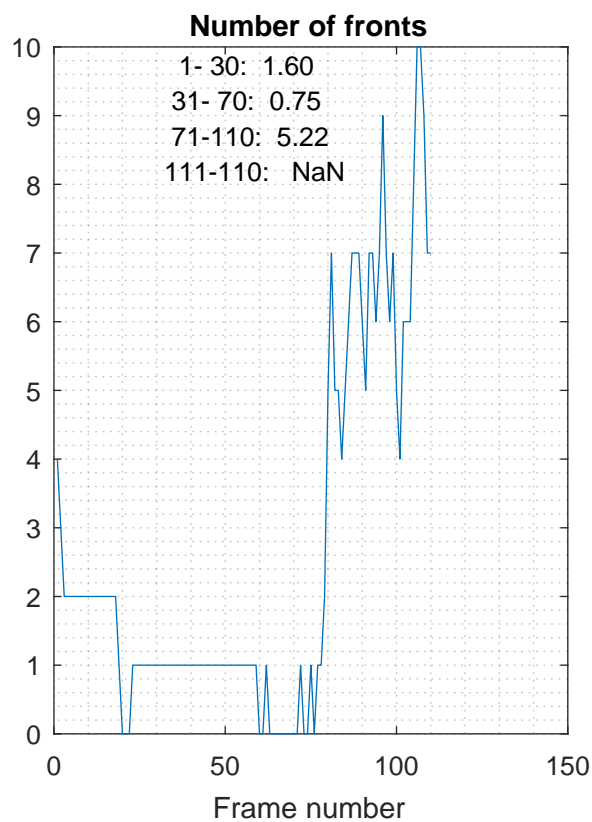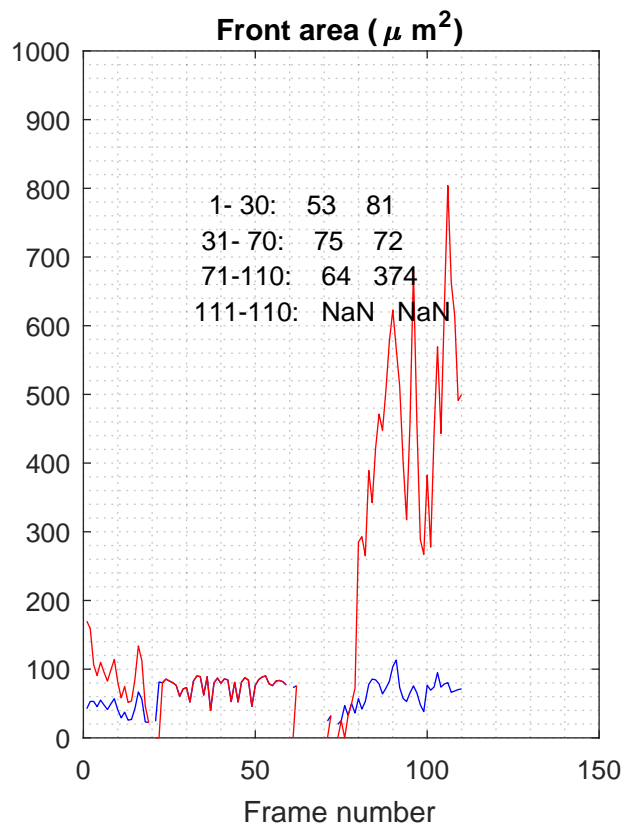

Supplement: Supplementary file 17 — Source Data for Appendix [file MSB-15-e8585-s025.zip › Source_data_for_Appendix/Appendix_Fig_S4/S4F/14.pdf]

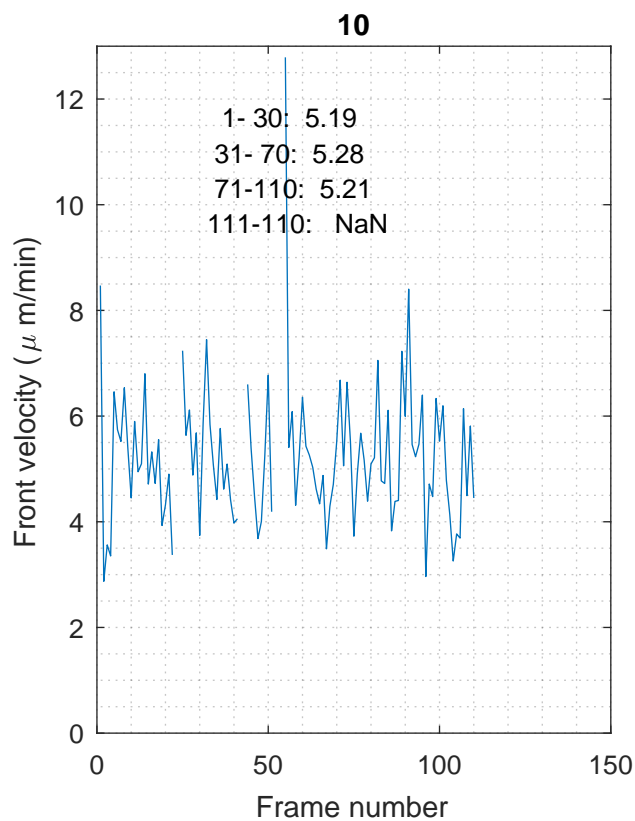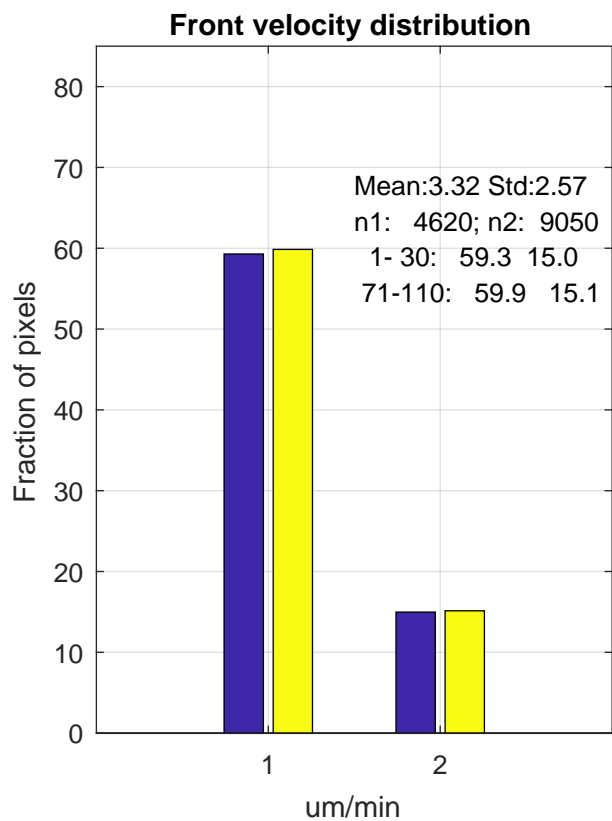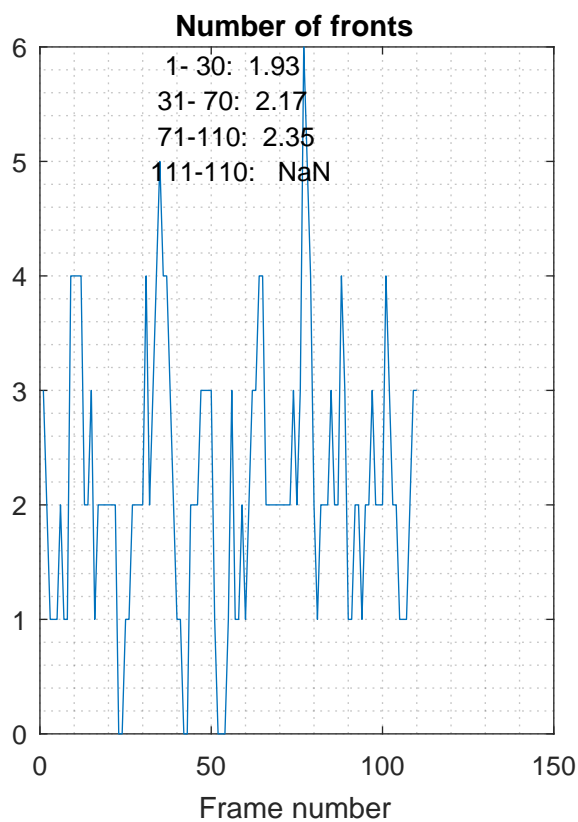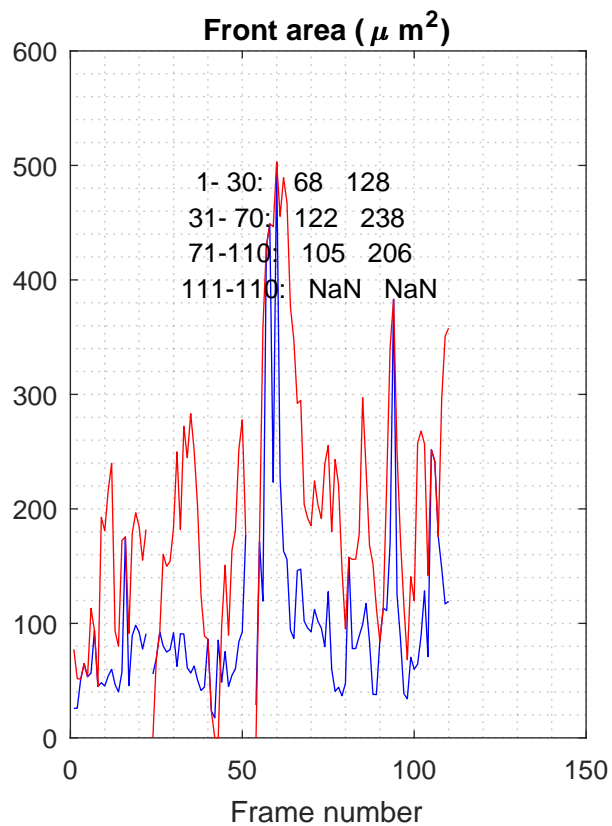

Supplement: Supplementary file 17 — Source Data for Appendix [file MSB-15-e8585-s025.zip › Source_data_for_Appendix/Appendix_Fig_S4/S4F/10.pdf]

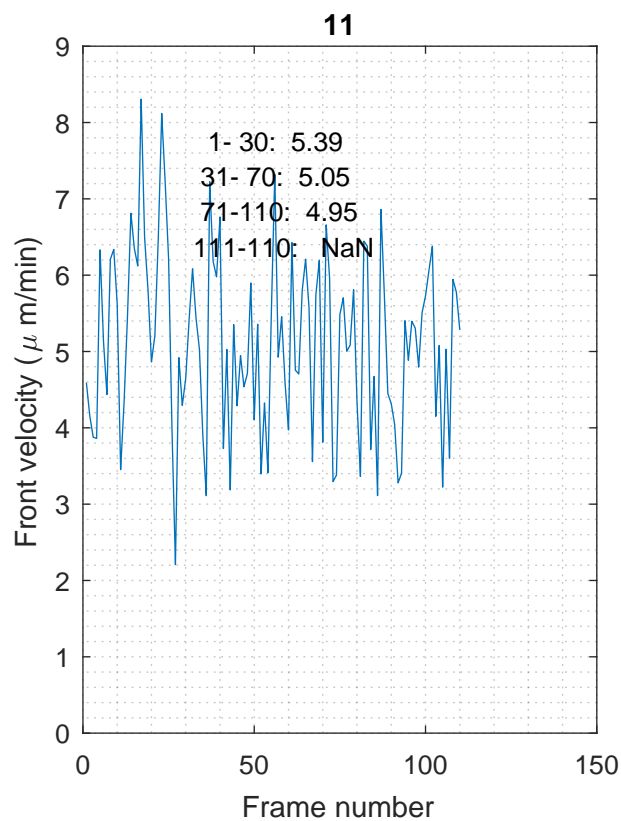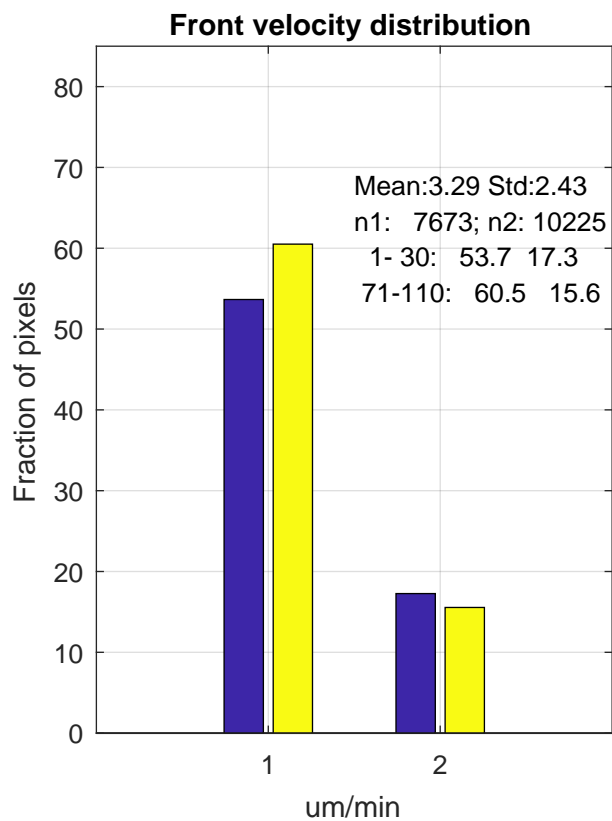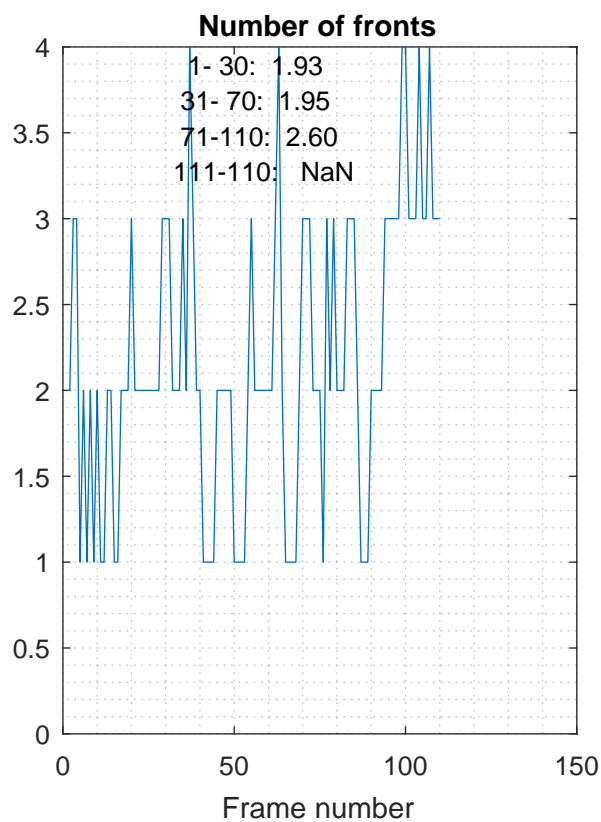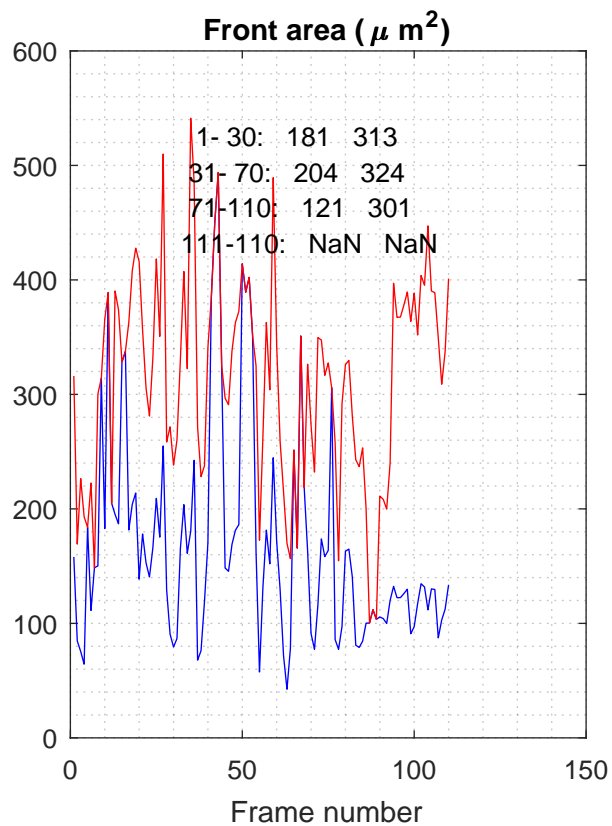

Supplement: Supplementary file 17 — Source Data for Appendix [file MSB-15-e8585-s025.zip › Source_data_for_Appendix/Appendix_Fig_S4/S4F/11.pdf]

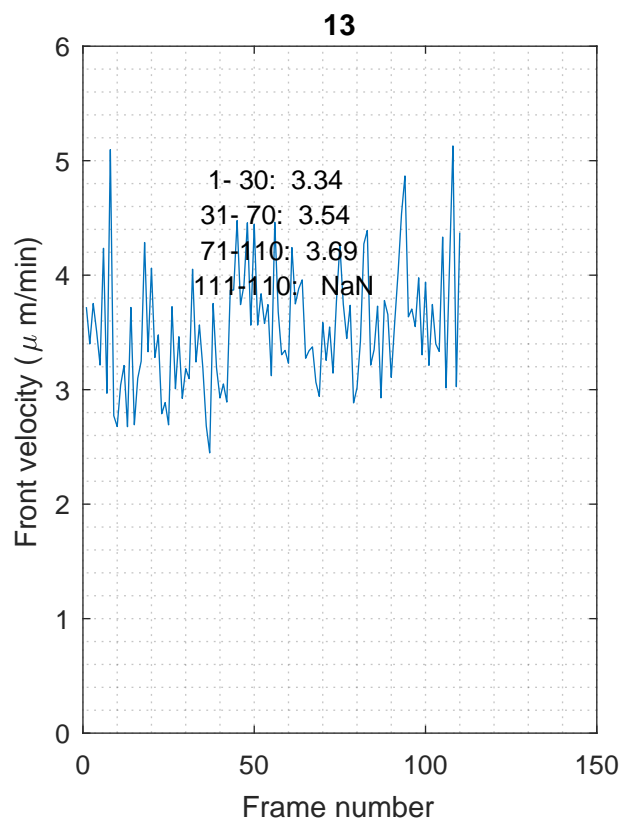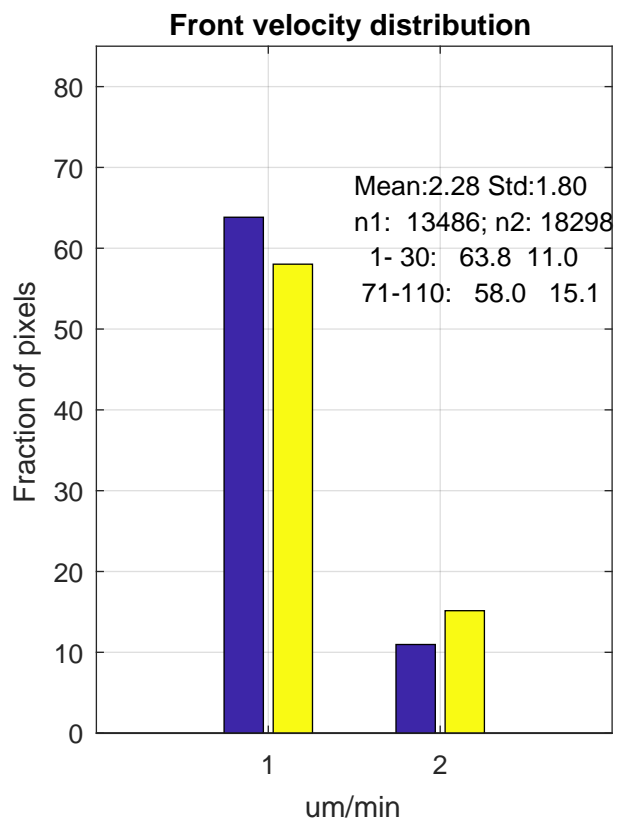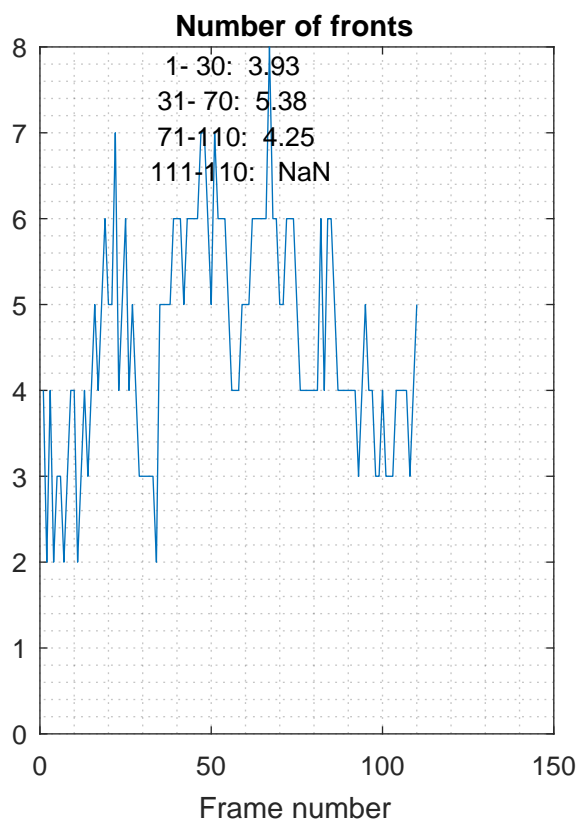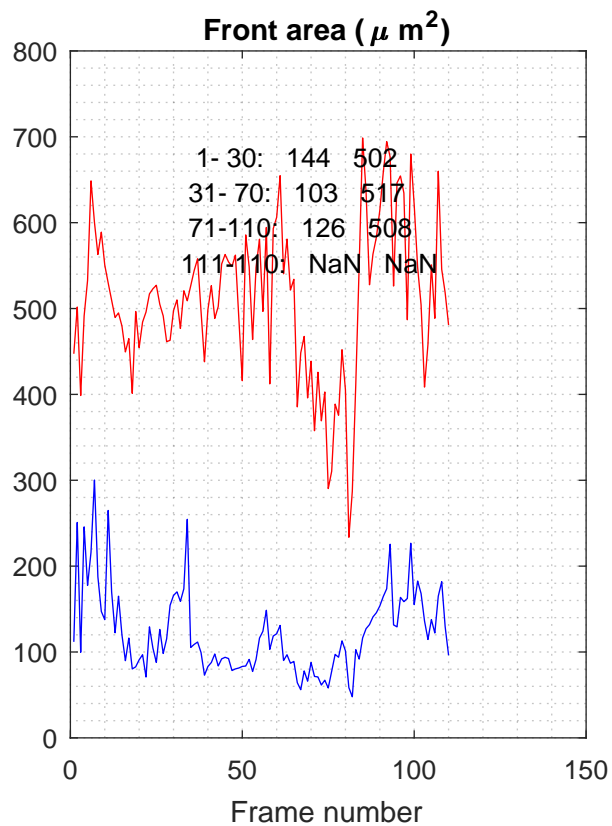

Supplement: Supplementary file 17 — Source Data for Appendix [file MSB-15-e8585-s025.zip › Source_data_for_Appendix/Appendix_Fig_S4/S4F/13.pdf]

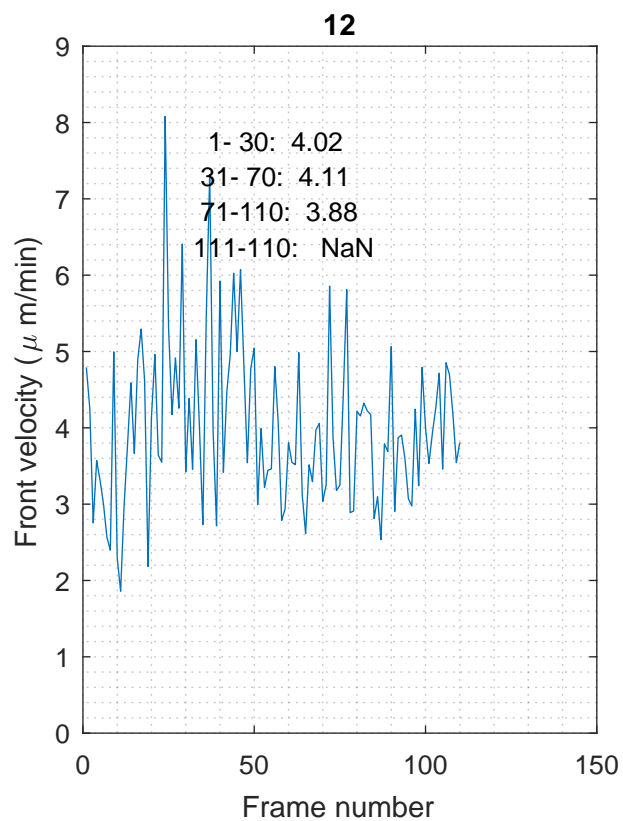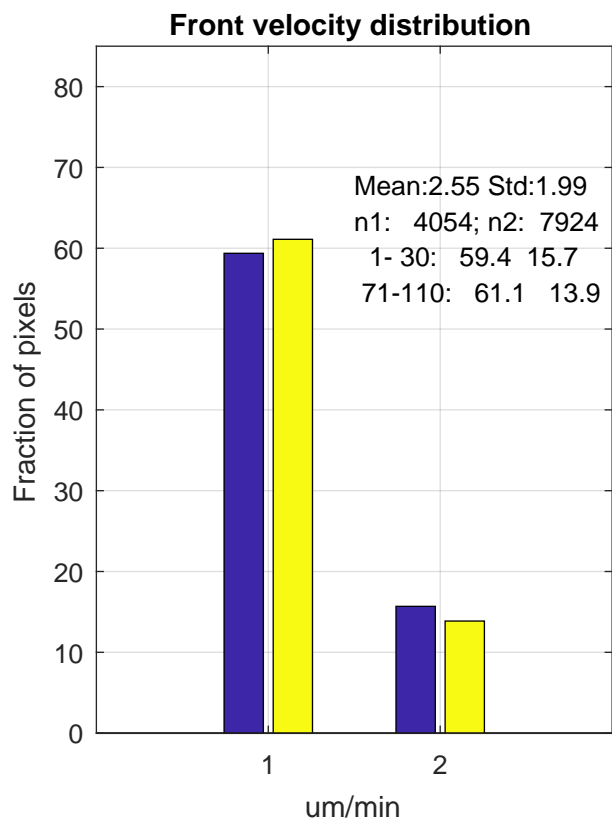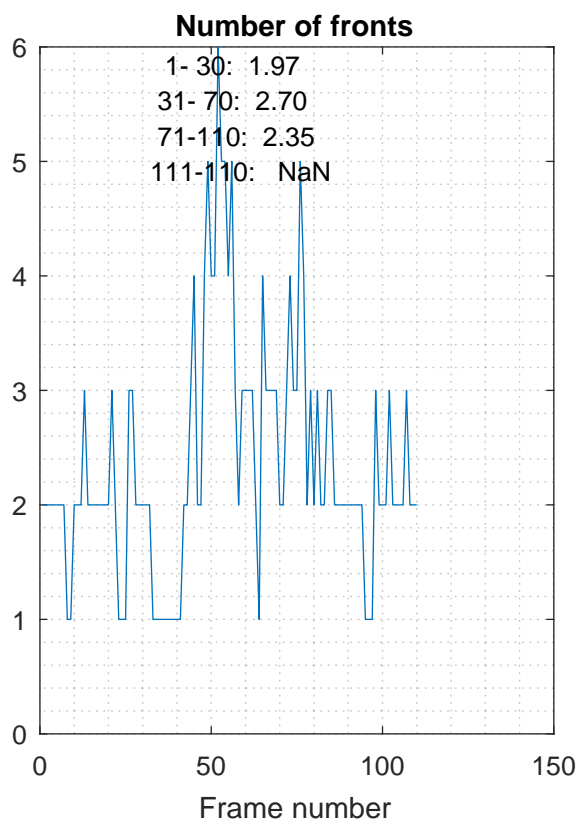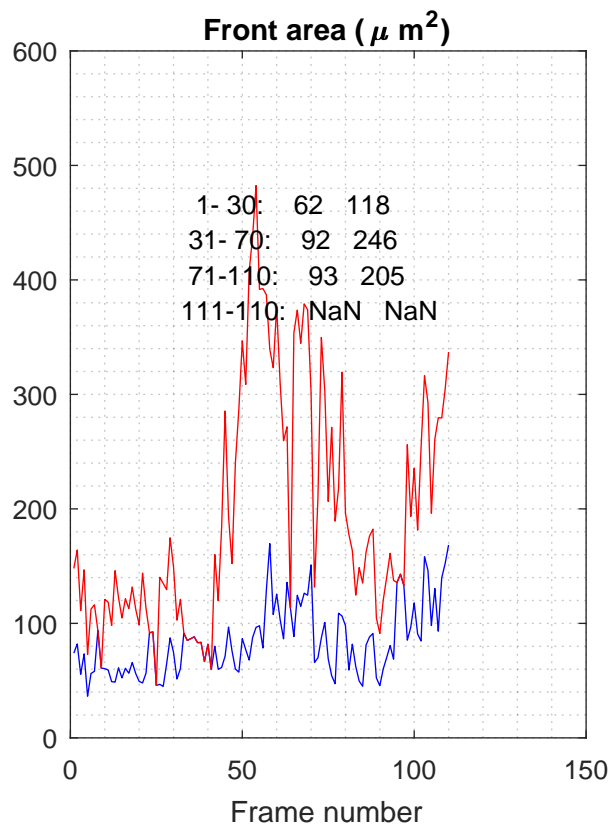

Supplement: Supplementary file 17 — Source Data for Appendix [file MSB-15-e8585-s025.zip › Source_data_for_Appendix/Appendix_Fig_S4/S4F/12.pdf]

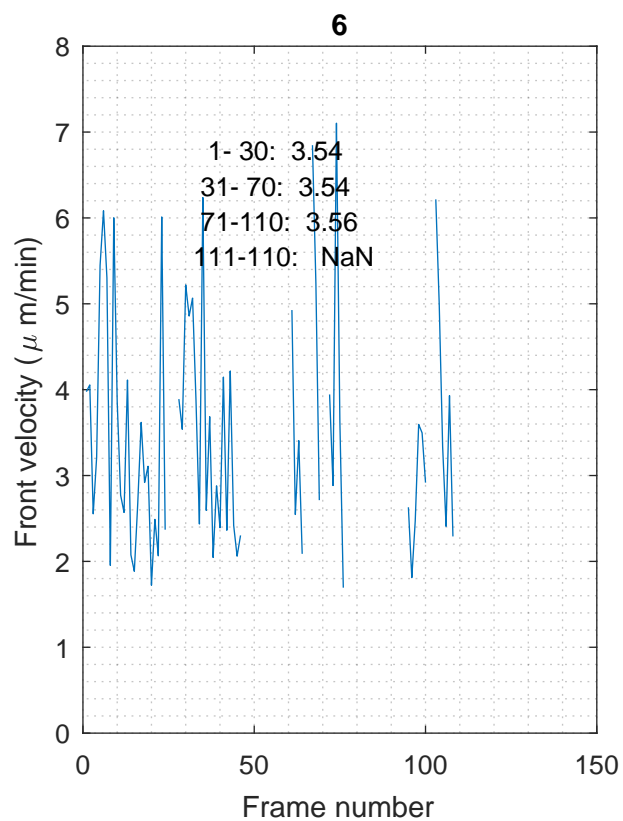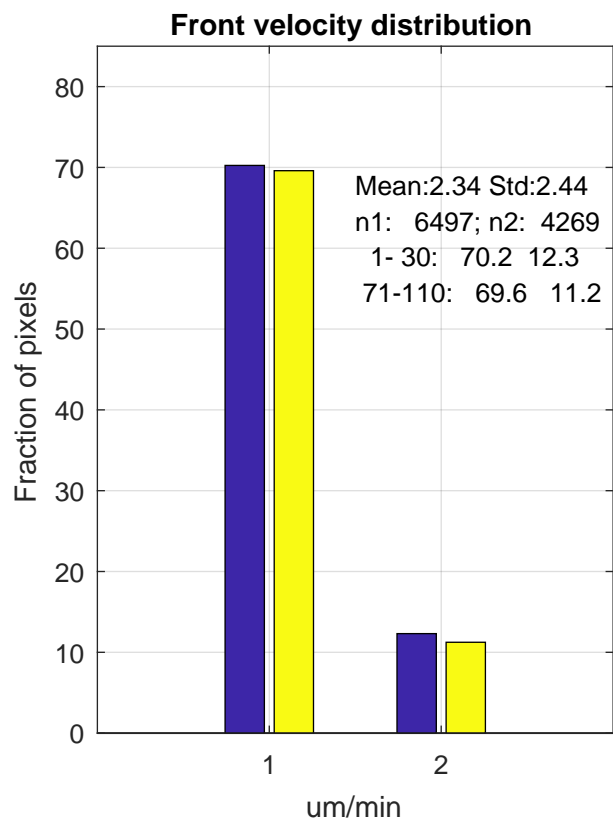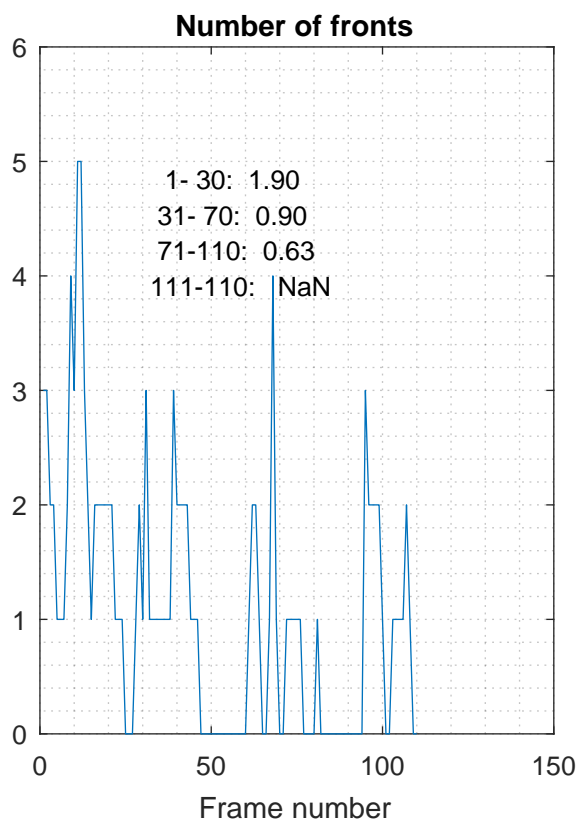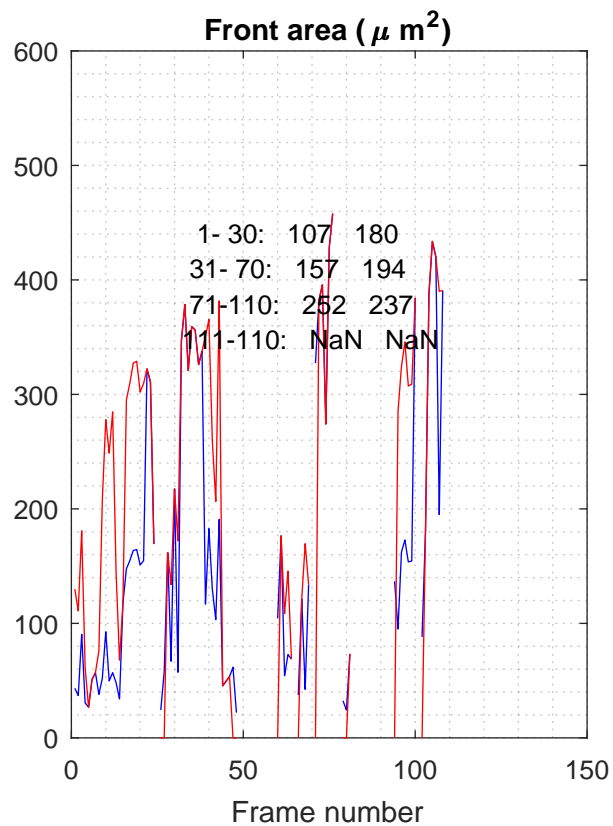

Supplement: Supplementary file 17 — Source Data for Appendix [file MSB-15-e8585-s025.zip › Source_data_for_Appendix/Appendix_Fig_S4/S4F/6.pdf]

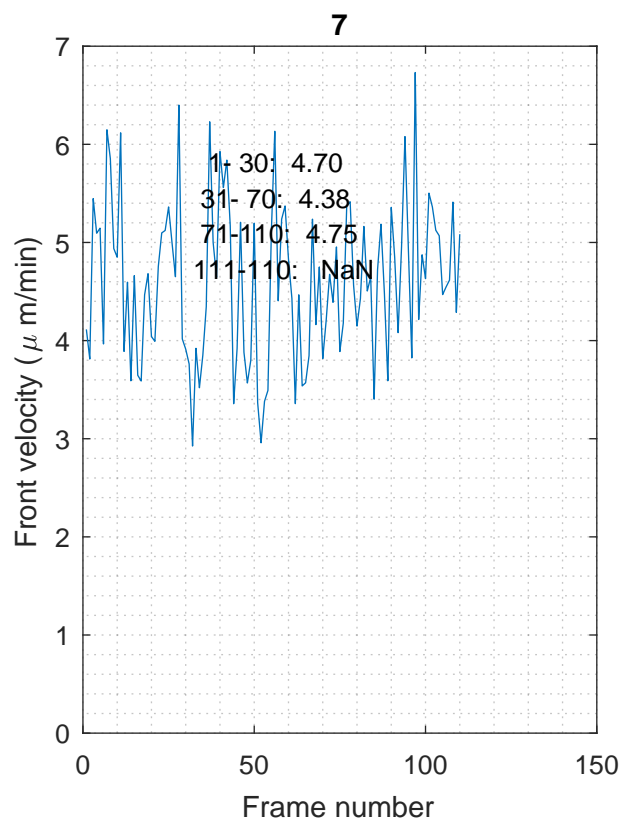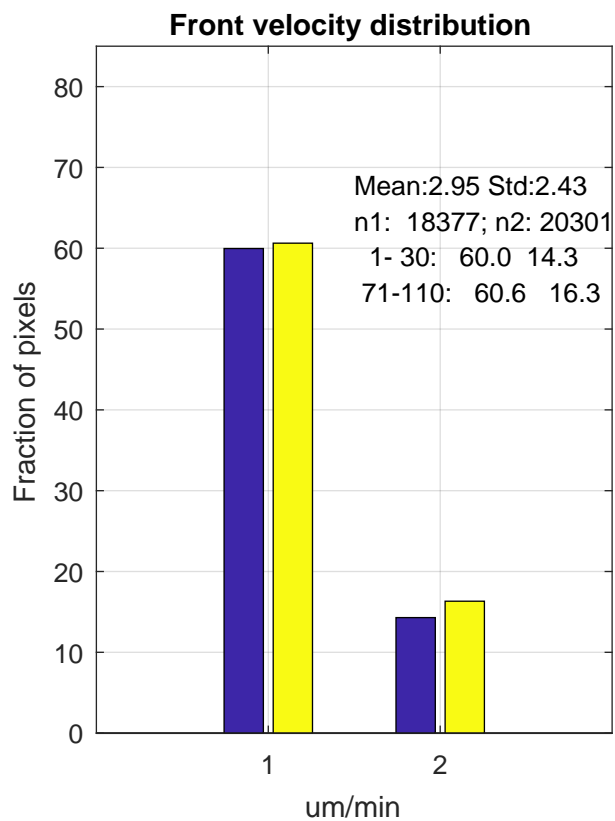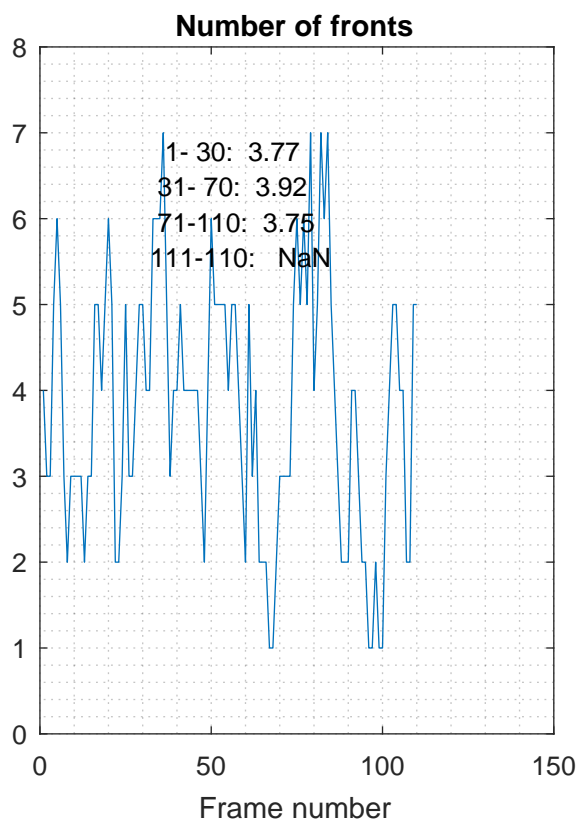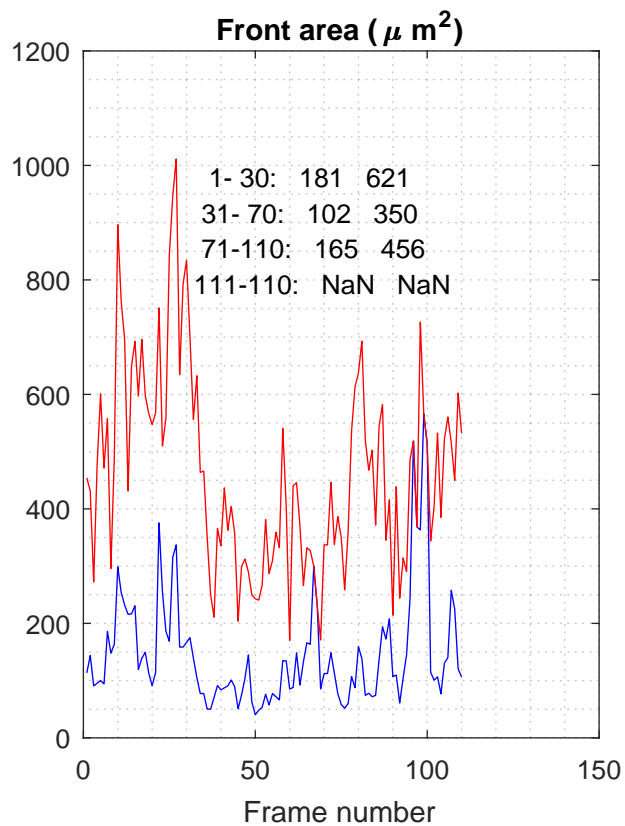

Supplement: Supplementary file 17 — Source Data for Appendix [file MSB-15-e8585-s025.zip › Source_data_for_Appendix/Appendix_Fig_S4/S4F/7.pdf]

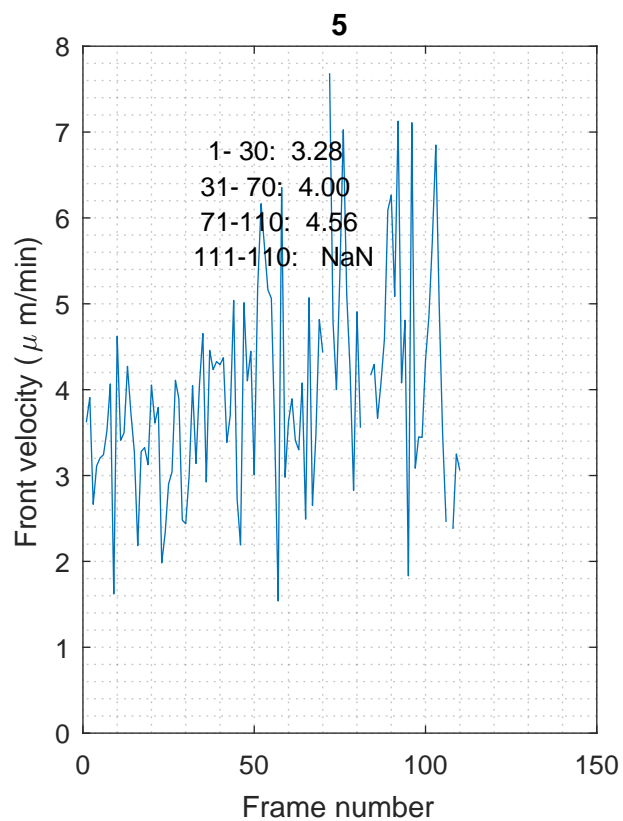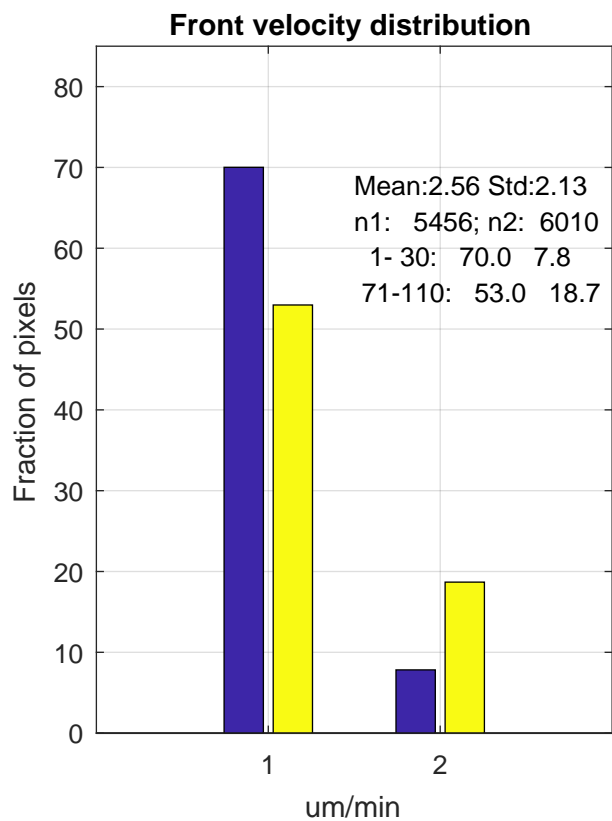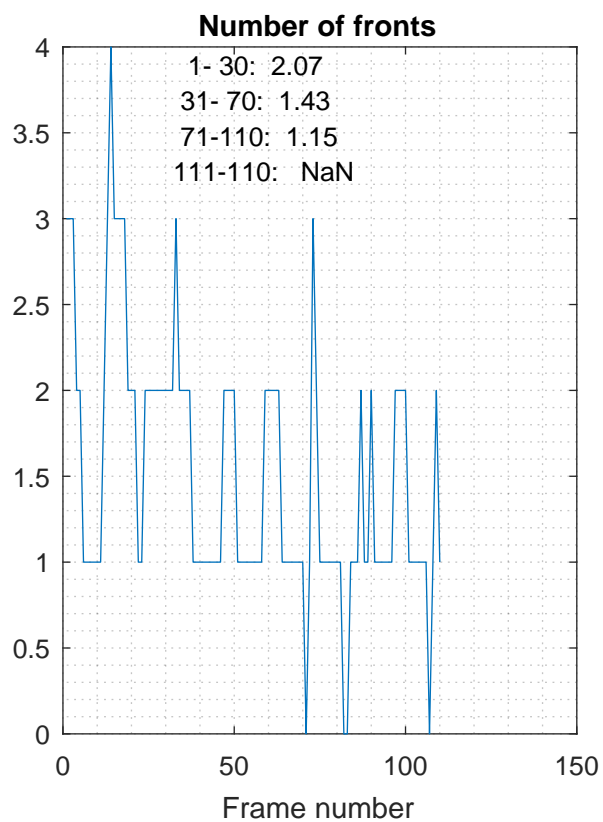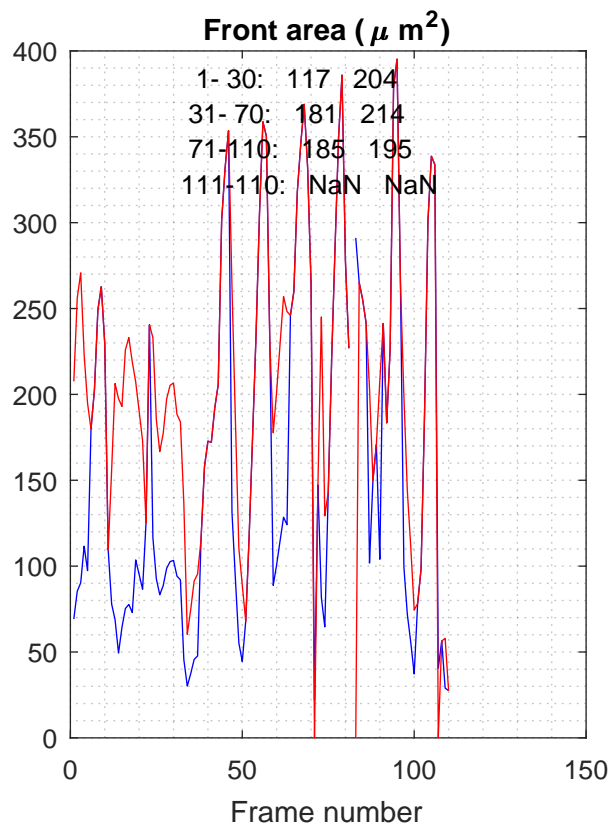

Supplement: Supplementary file 17 — Source Data for Appendix [file MSB-15-e8585-s025.zip › Source_data_for_Appendix/Appendix_Fig_S4/S4F/5.pdf]

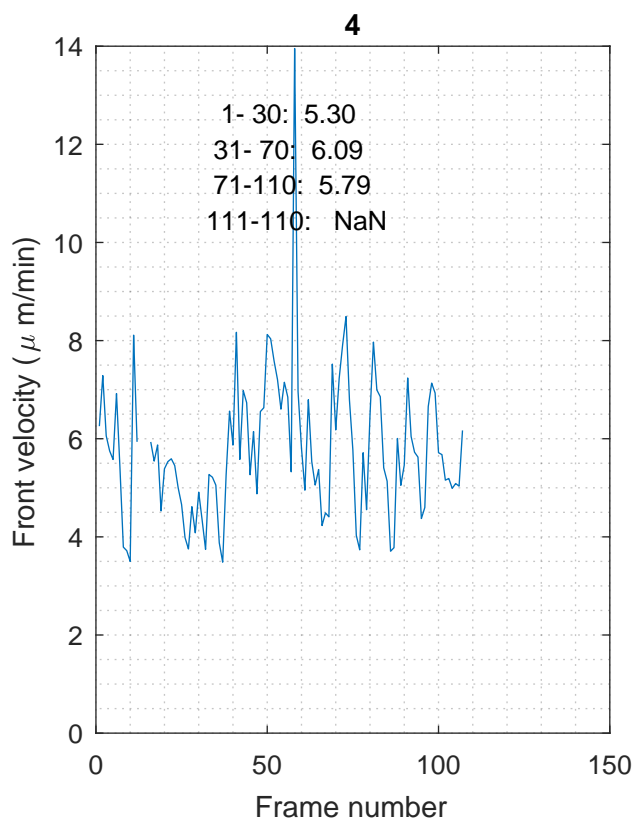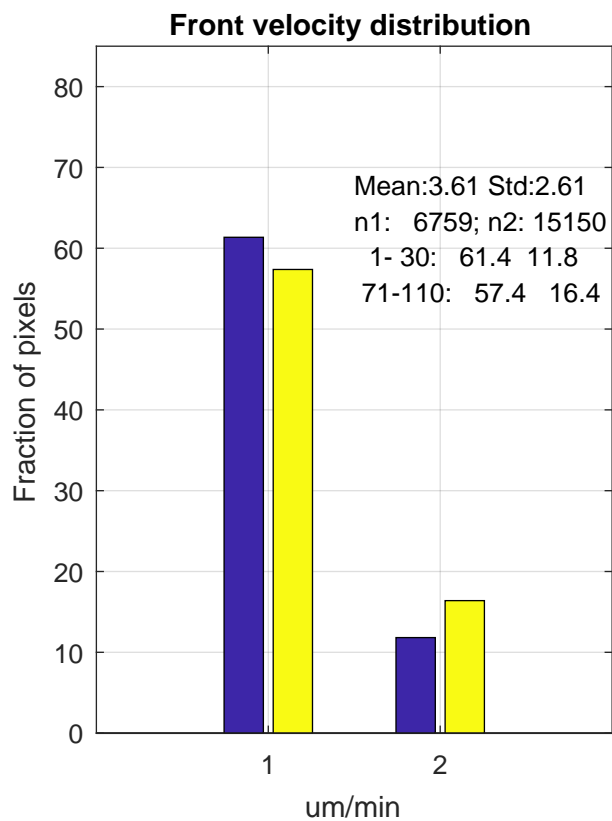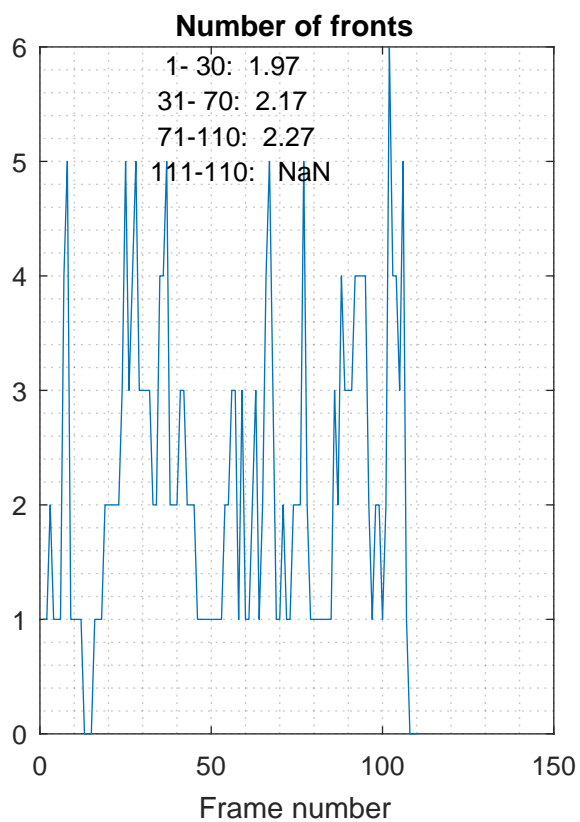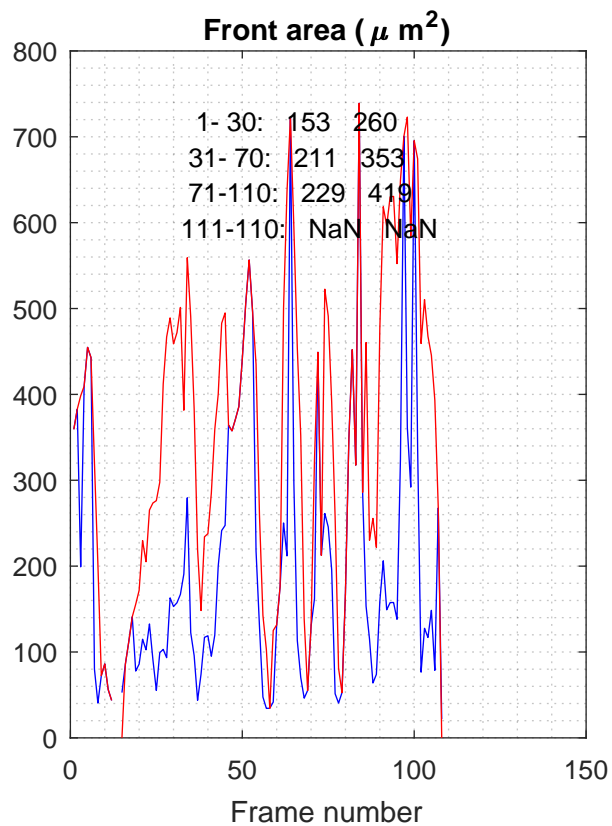

Supplement: Supplementary file 17 — Source Data for Appendix [file MSB-15-e8585-s025.zip › Source_data_for_Appendix/Appendix_Fig_S4/S4F/4.pdf]

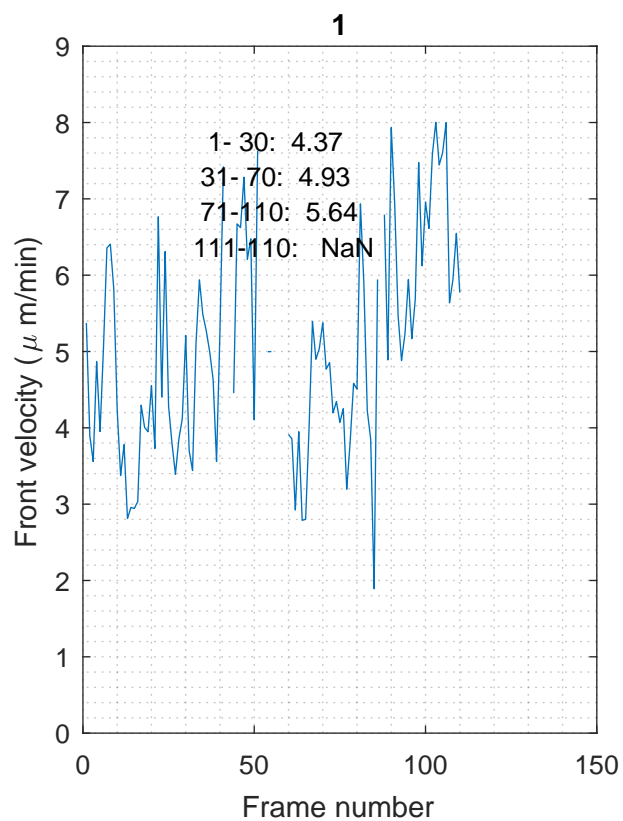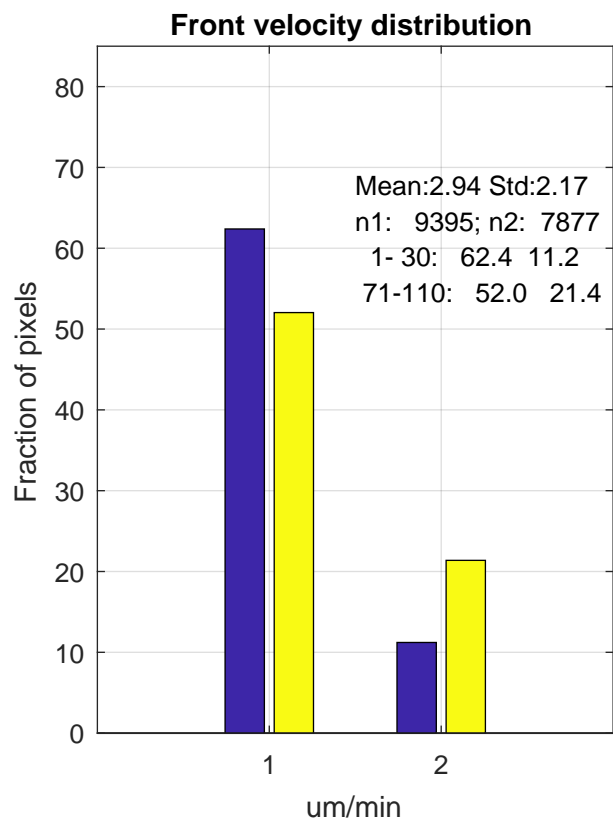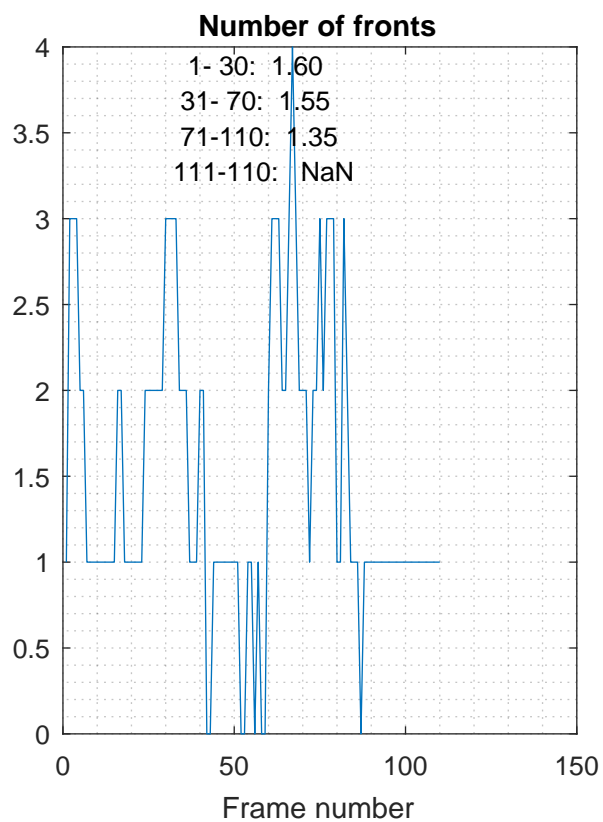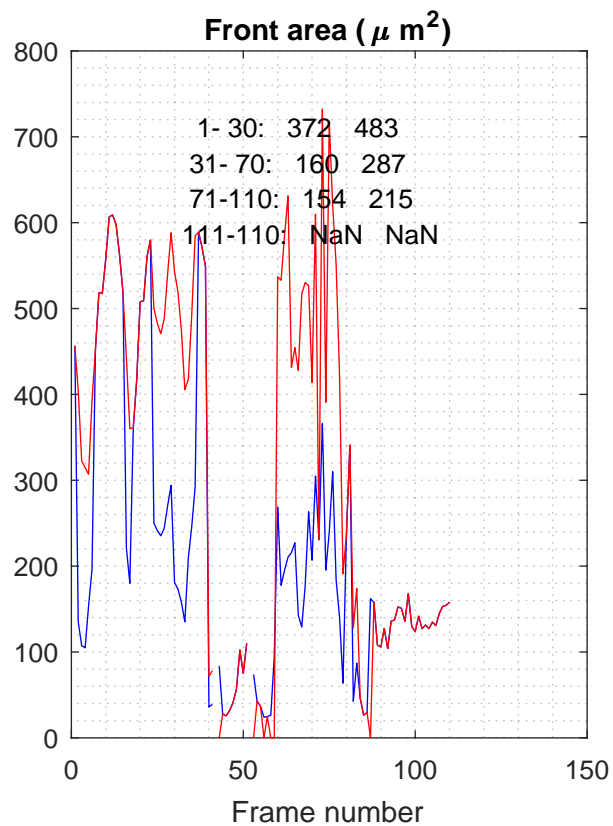

Supplement: Supplementary file 17 — Source Data for Appendix [file MSB-15-e8585-s025.zip › Source_data_for_Appendix/Appendix_Fig_S4/S4F/1.pdf]

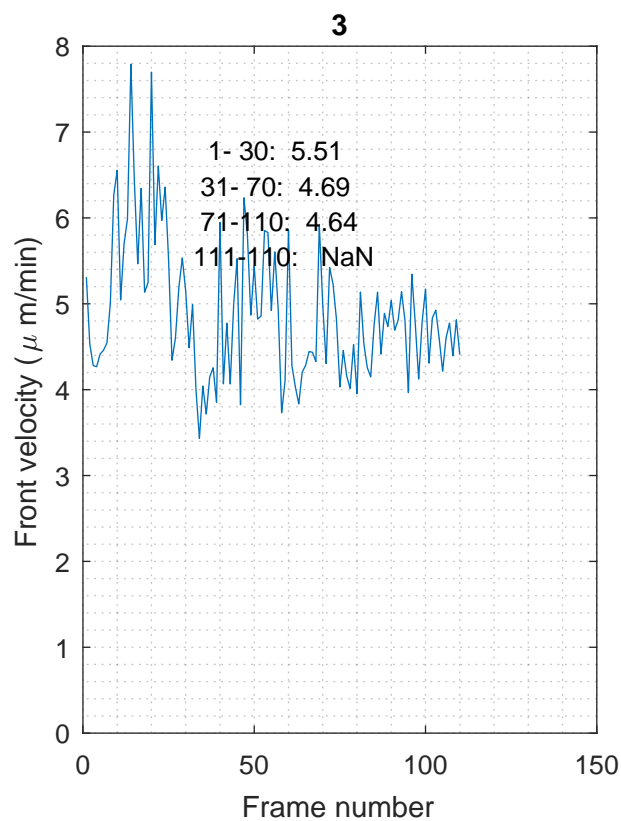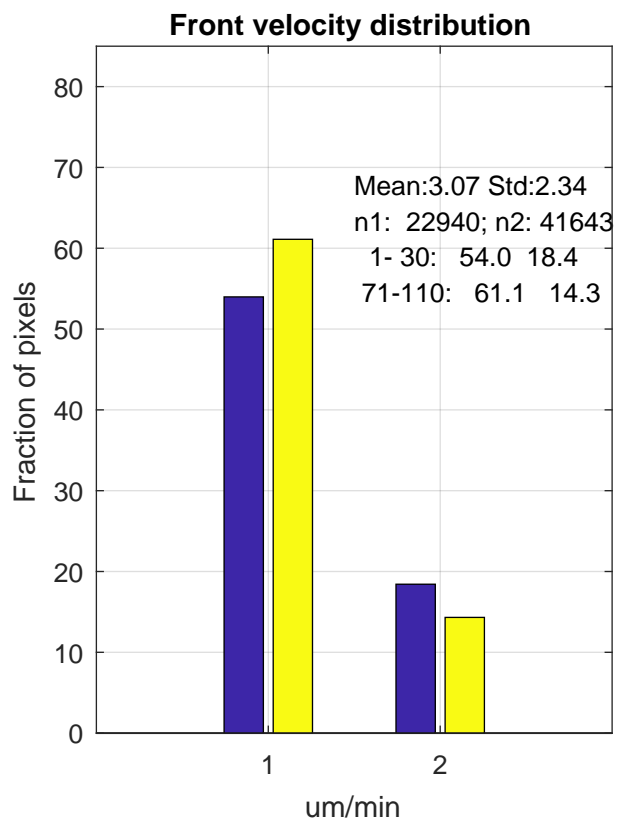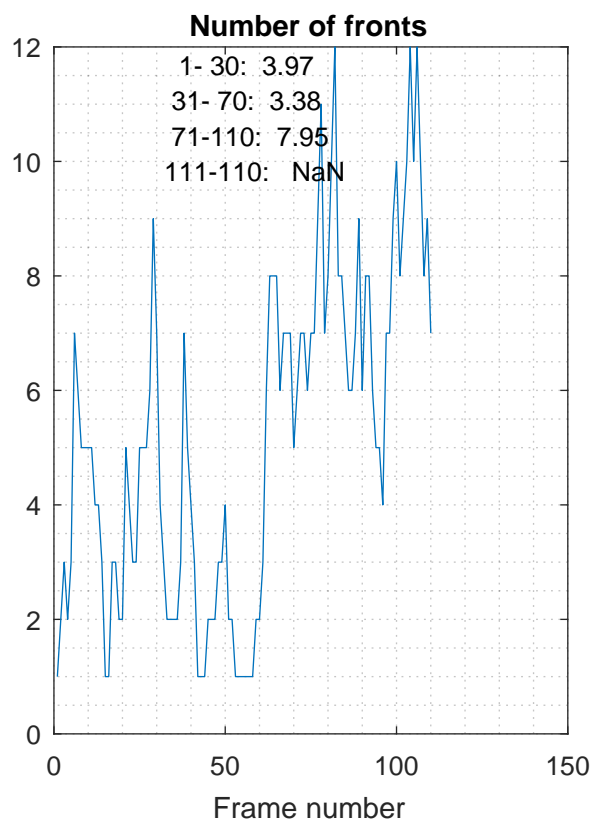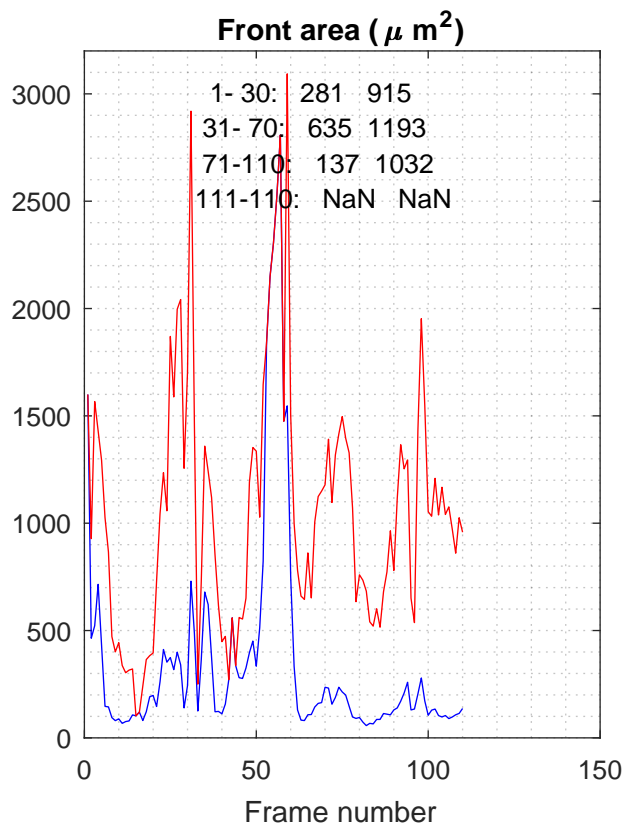

Supplement: Supplementary file 17 — Source Data for Appendix [file MSB-15-e8585-s025.zip › Source_data_for_Appendix/Appendix_Fig_S4/S4F/3.pdf]

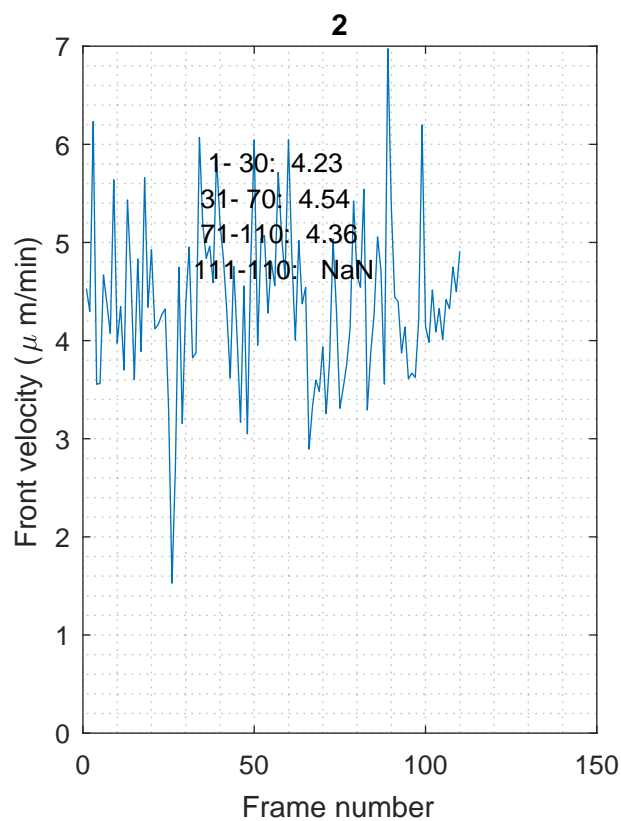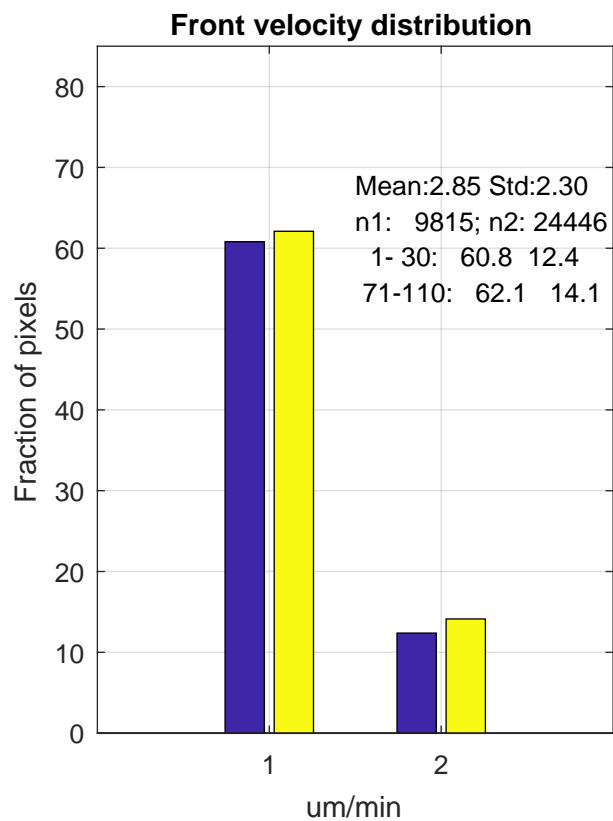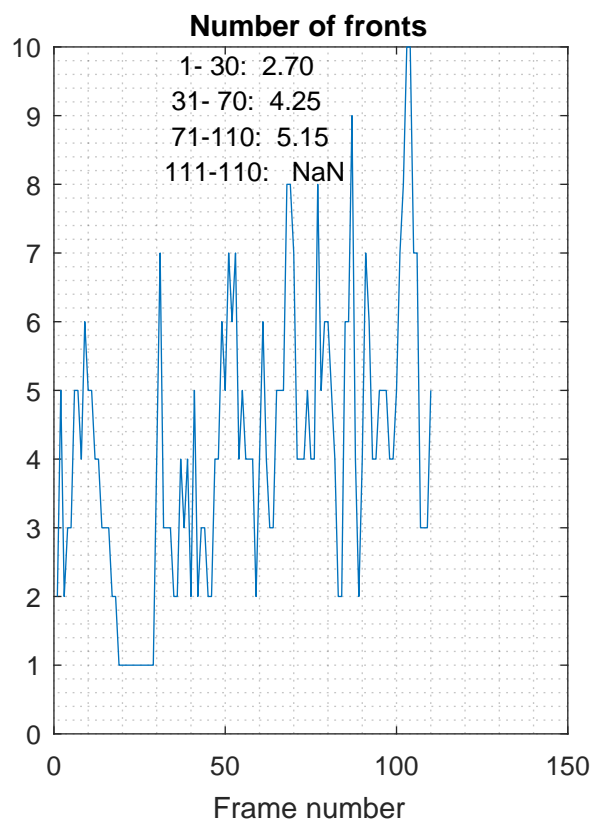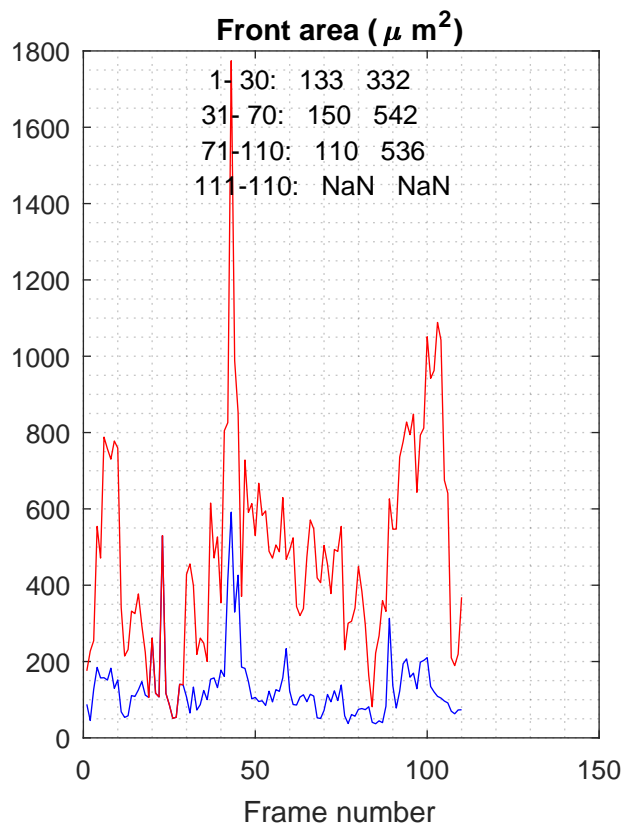

Supplement: Supplementary file 17 — Source Data for Appendix [file MSB-15-e8585-s025.zip › Source_data_for_Appendix/Appendix_Fig_S4/S4F/2.pdf]

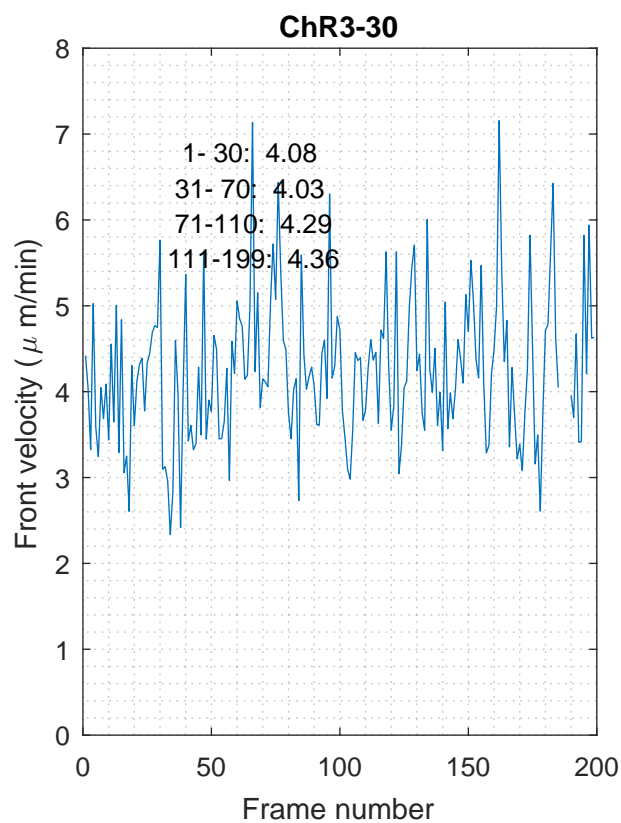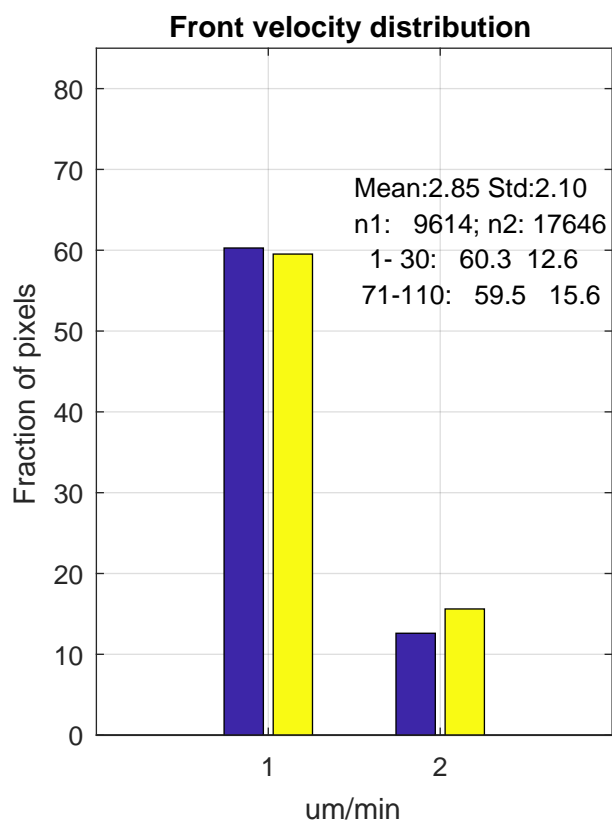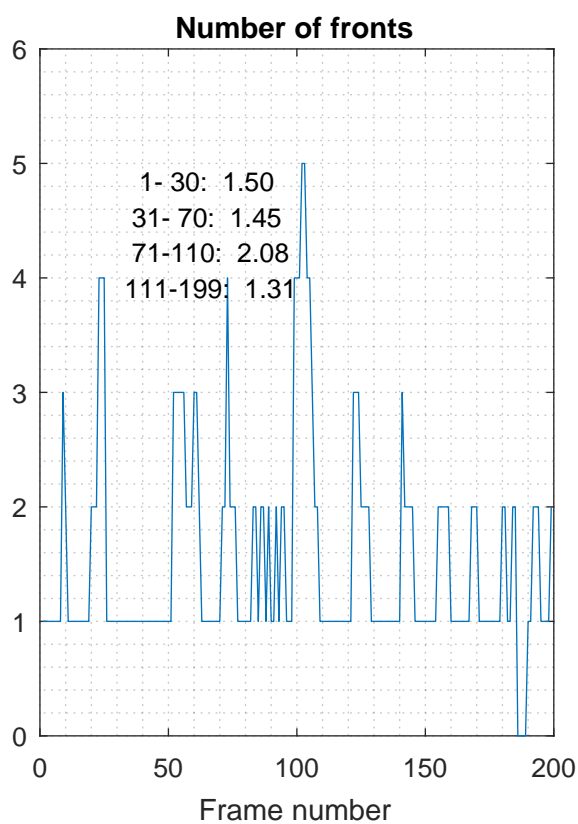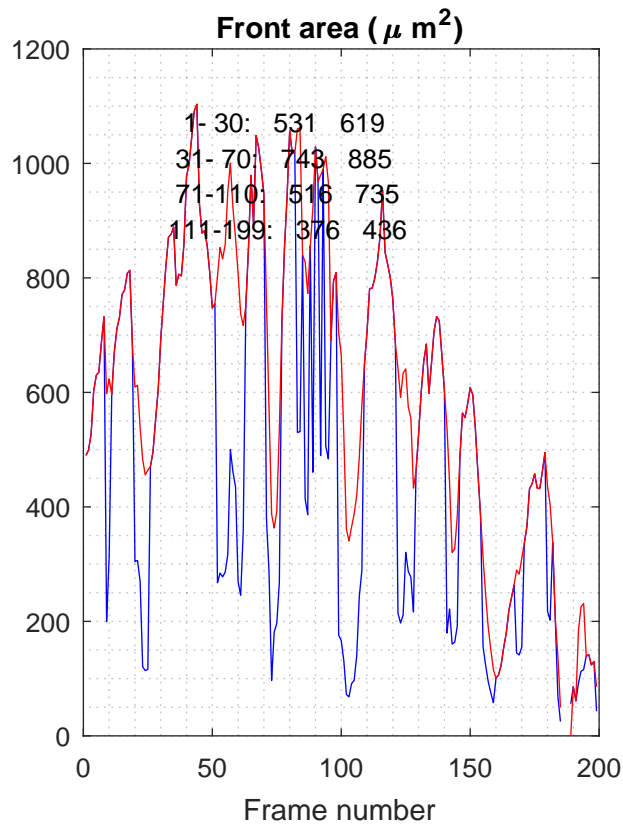

Supplement: Supplementary file 22 — Source Data for Figure 4 [file MSB-15-e8585-s020.zip › Source_data_for_Figure_4/Fig_4I/ChR3-30.pdf]

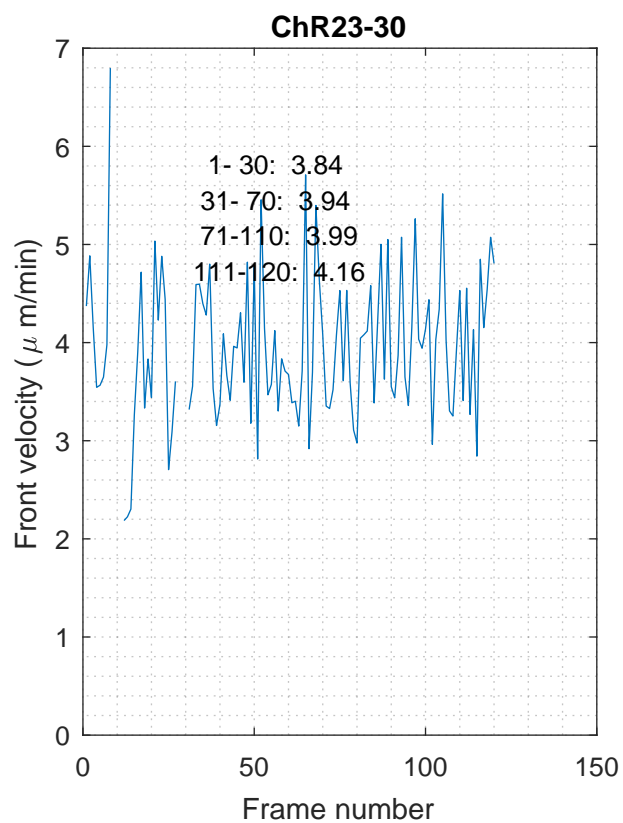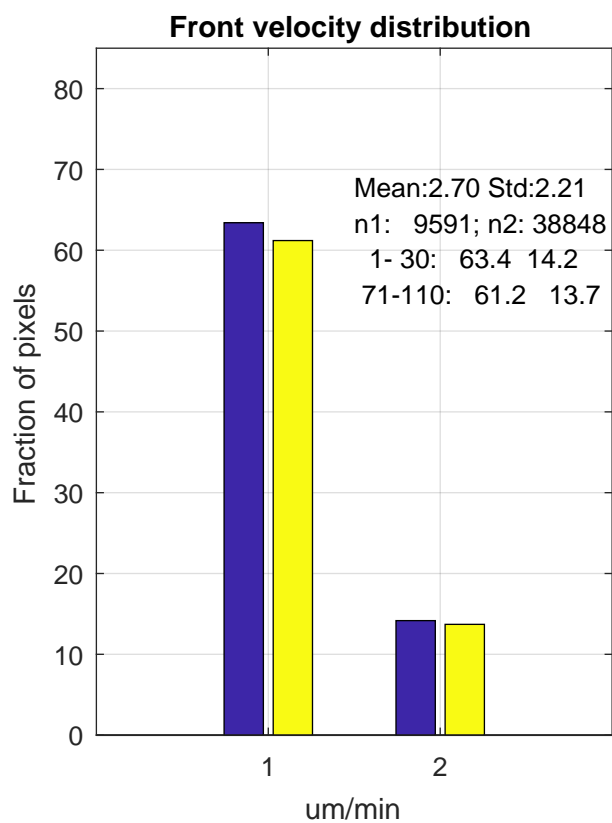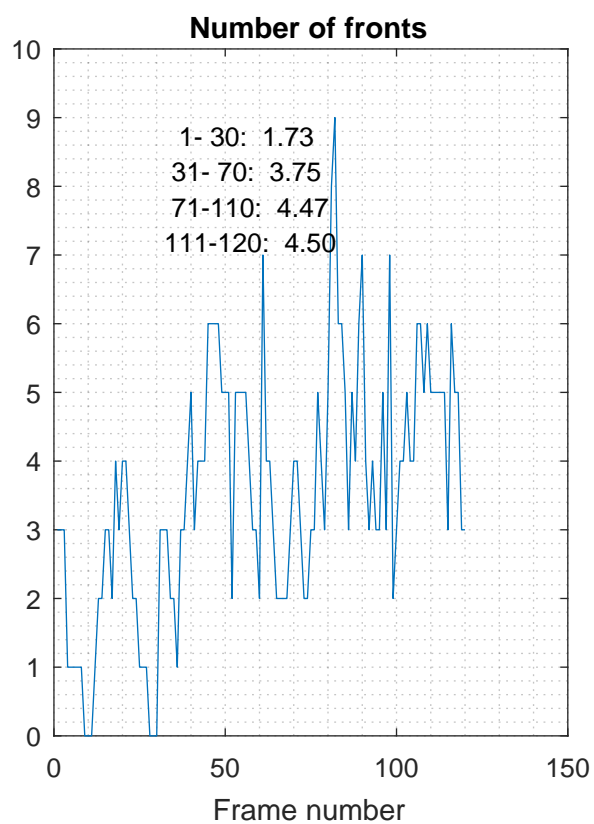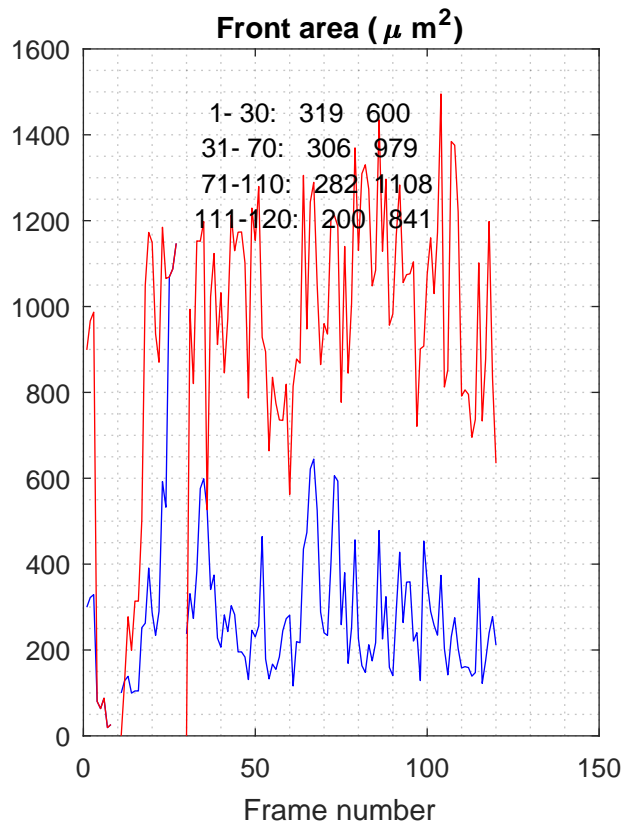

Supplement: Supplementary file 22 — Source Data for Figure 4 [file MSB-15-e8585-s020.zip › Source_data_for_Figure_4/Fig_4I/ChR23-30.pdf]

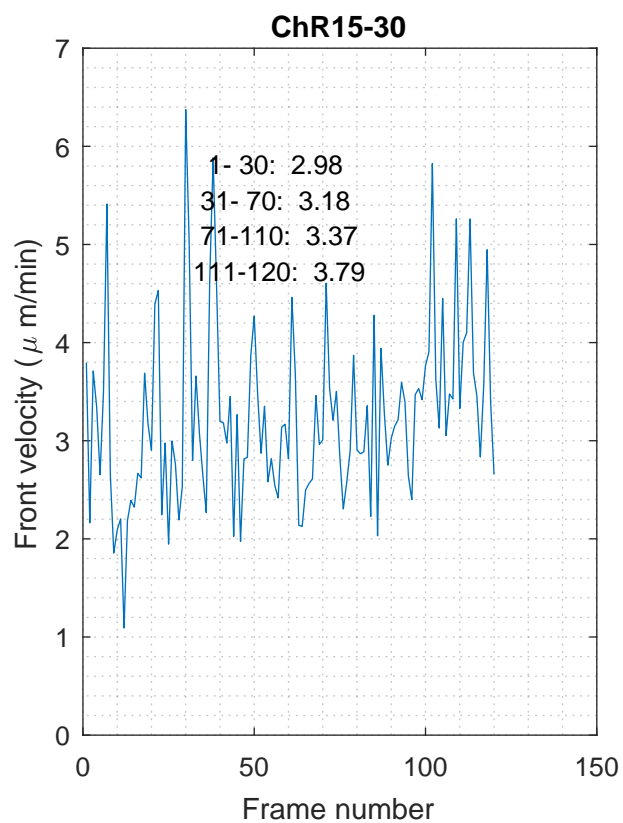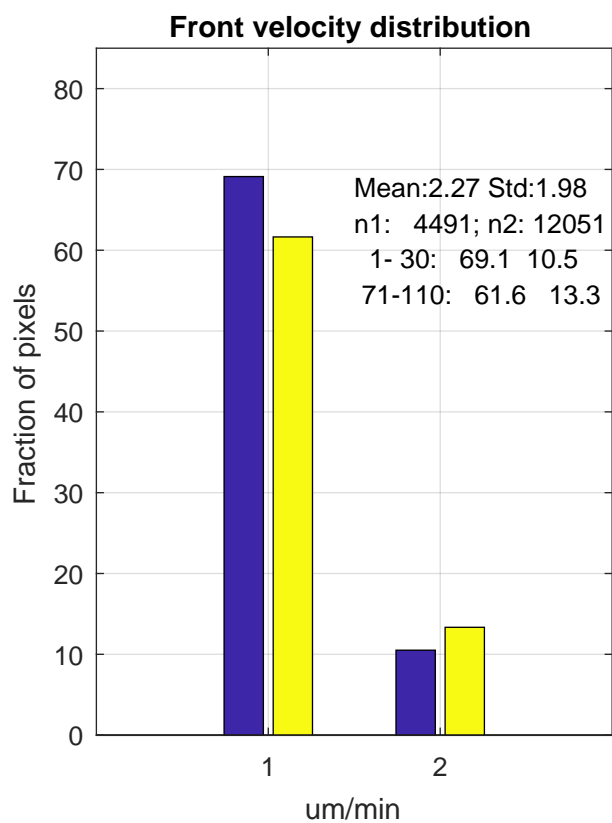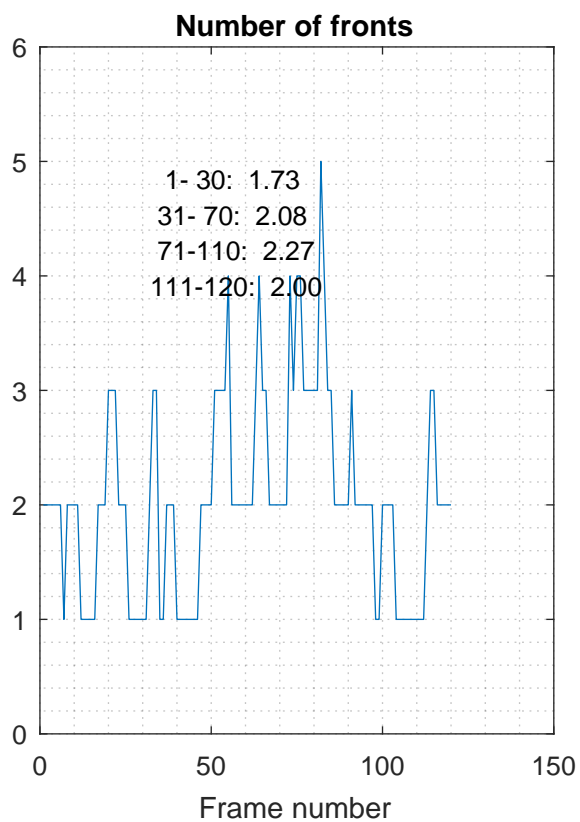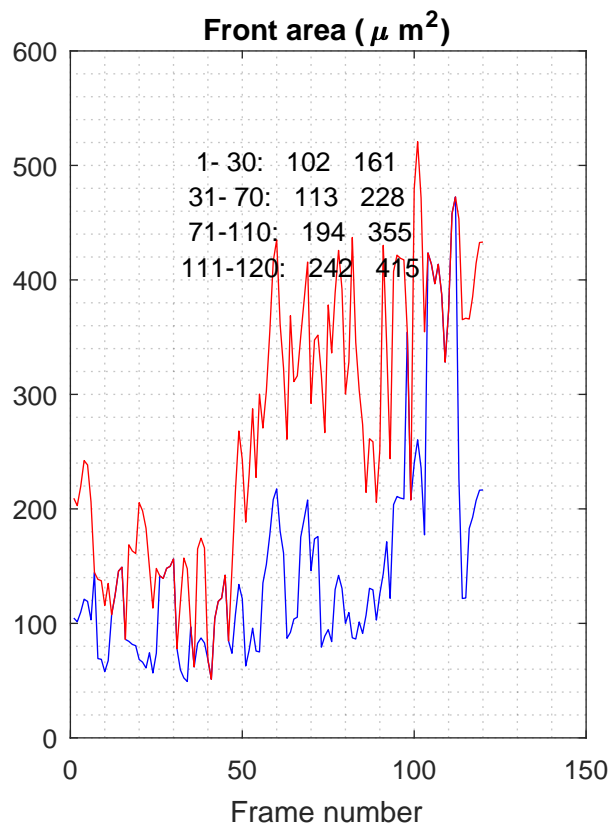

Supplement: Supplementary file 22 — Source Data for Figure 4 [file MSB-15-e8585-s020.zip › Source_data_for_Figure_4/Fig_4I/ChR15-30.pdf]

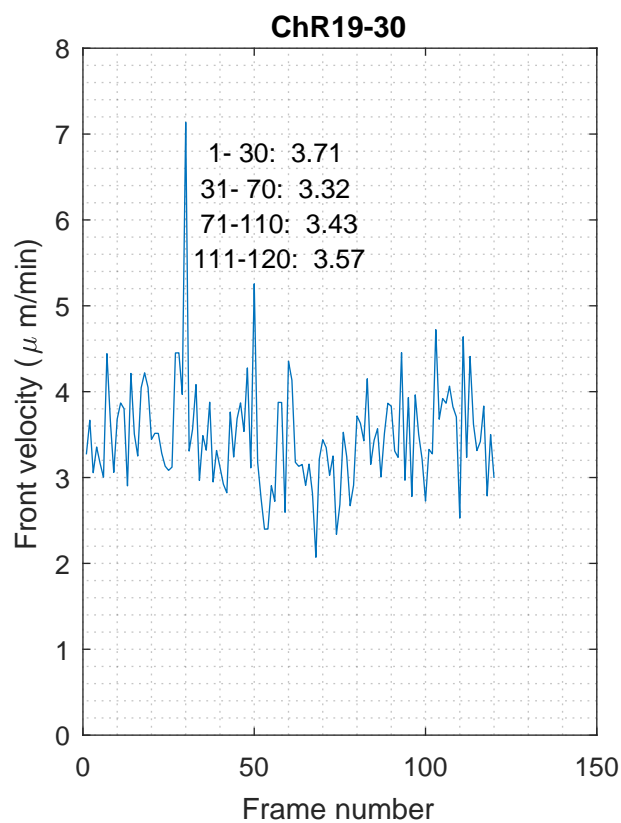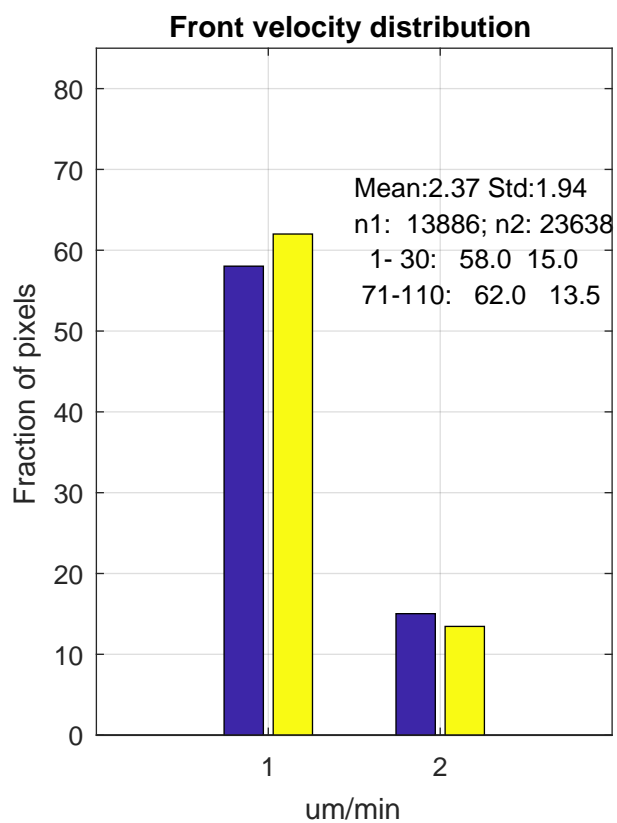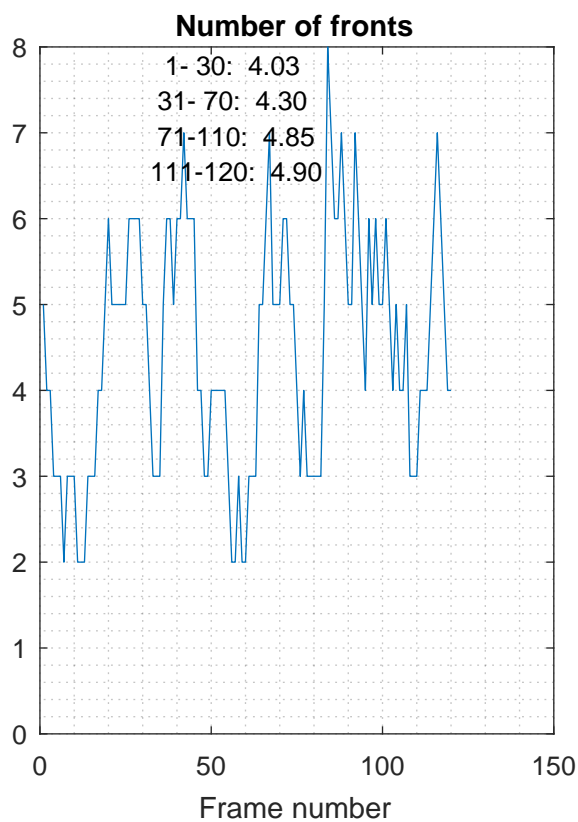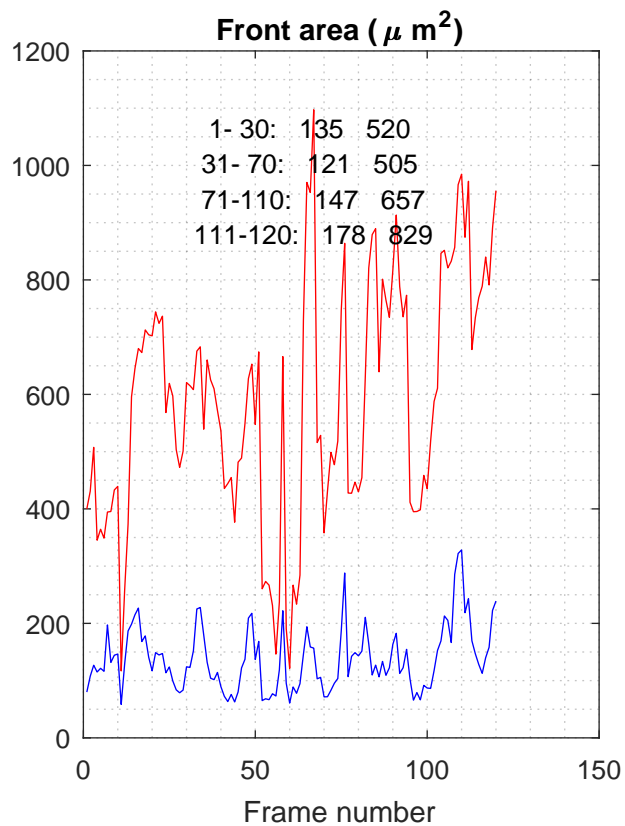

Supplement: Supplementary file 22 — Source Data for Figure 4 [file MSB-15-e8585-s020.zip › Source_data_for_Figure_4/Fig_4I/ChR19-30.pdf]

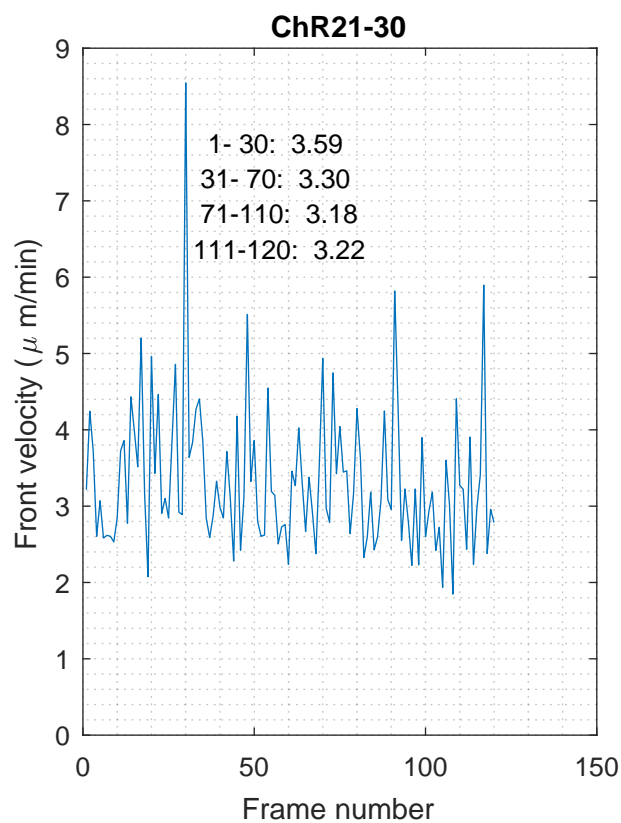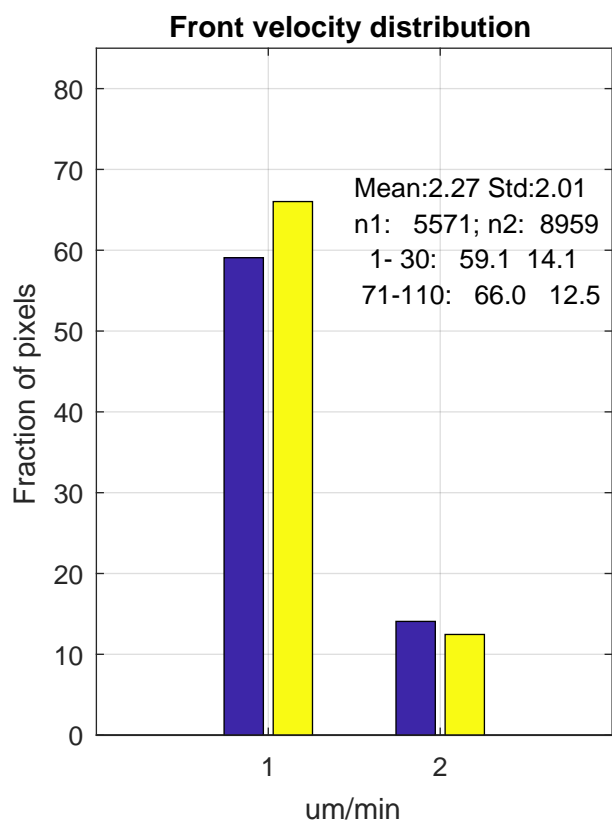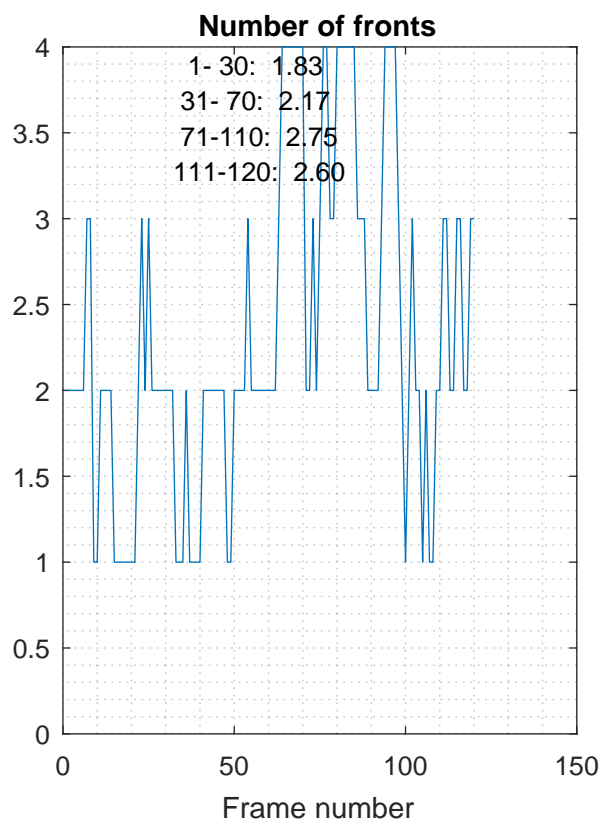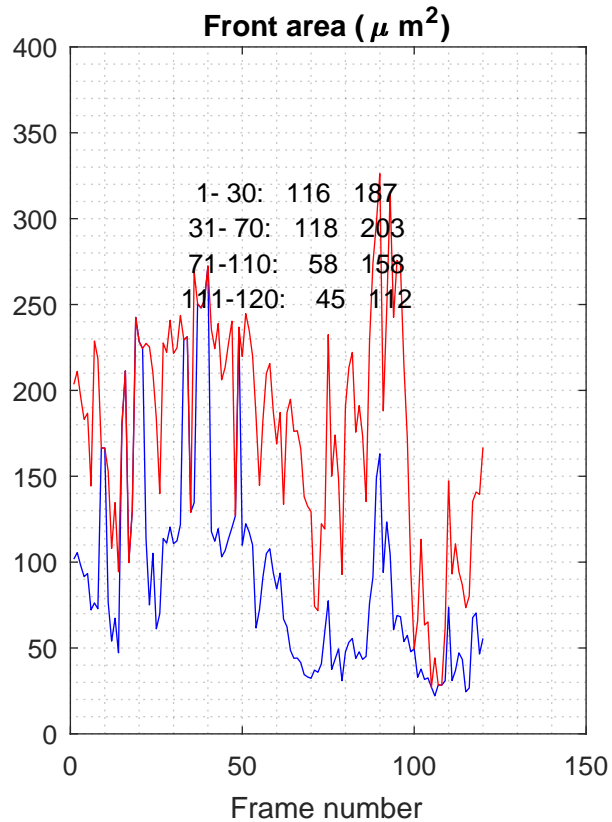

Supplement: Supplementary file 22 — Source Data for Figure 4 [file MSB-15-e8585-s020.zip › Source_data_for_Figure_4/Fig_4I/ChR21-30.pdf]

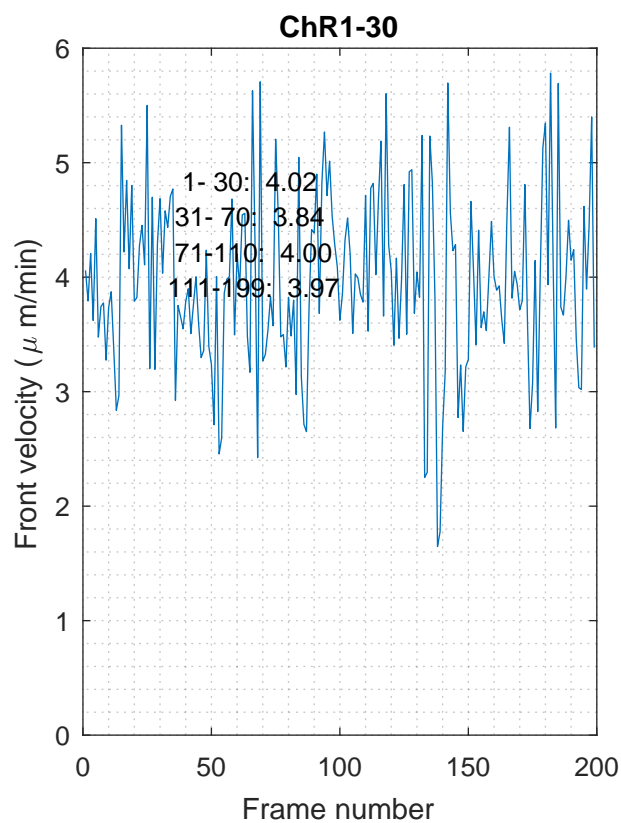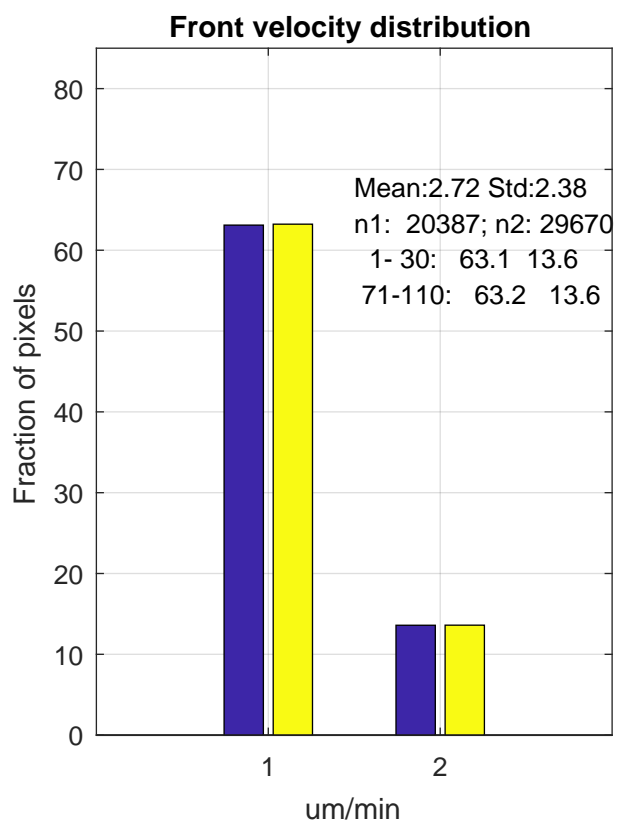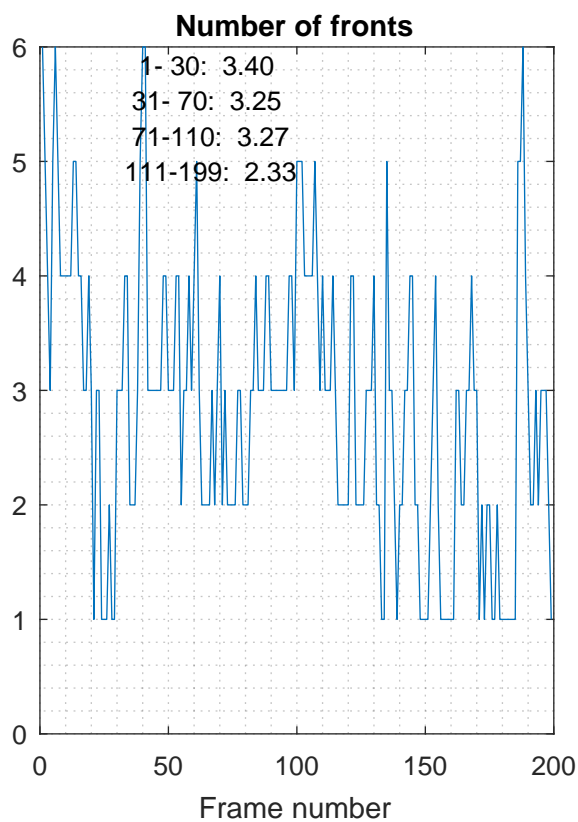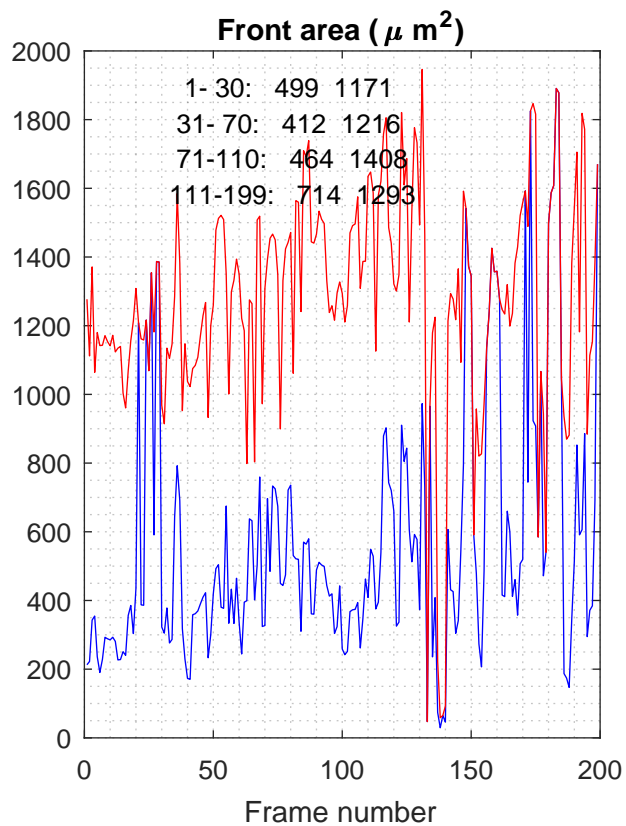

Supplement: Supplementary file 22 — Source Data for Figure 4 [file MSB-15-e8585-s020.zip › Source_data_for_Figure_4/Fig_4I/ChR1-30.pdf]

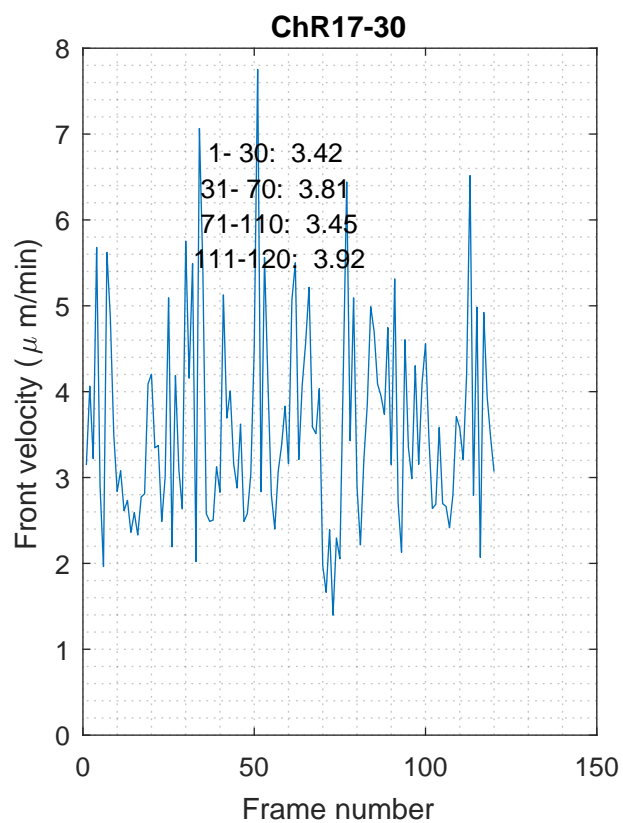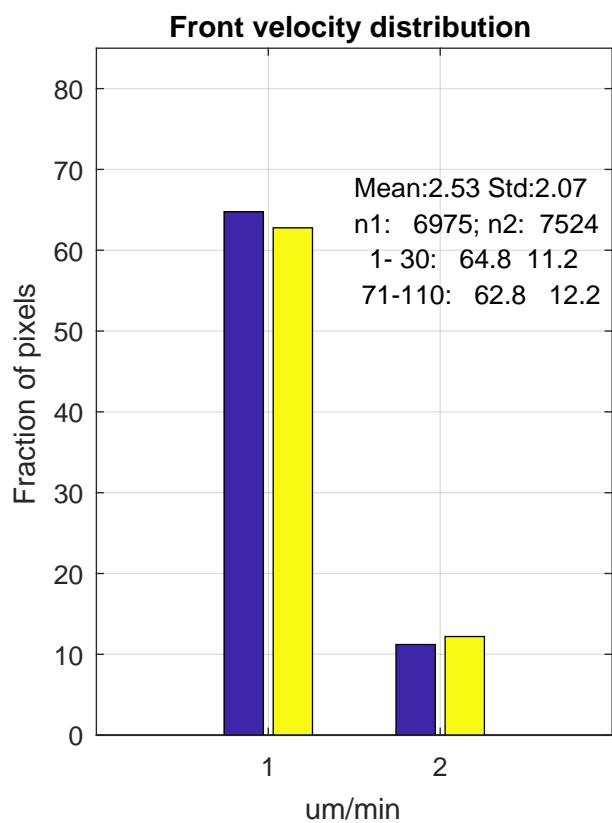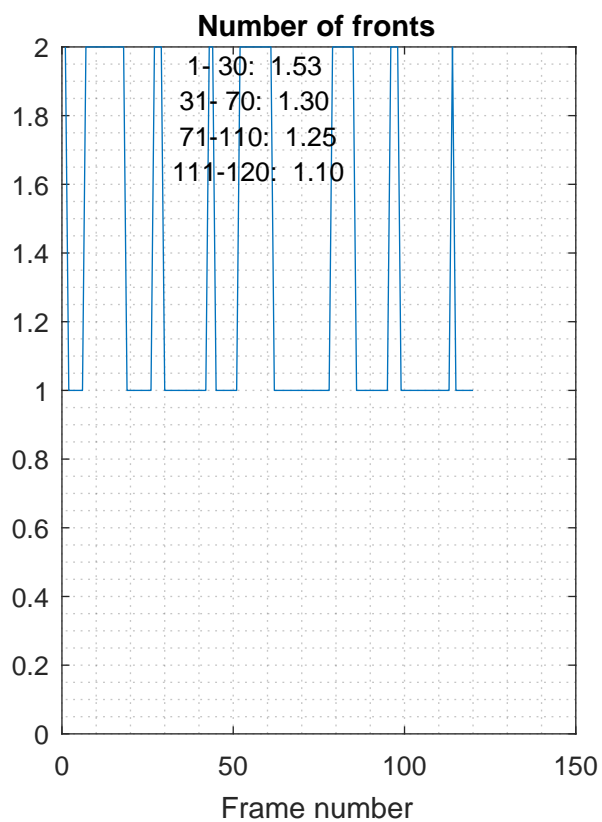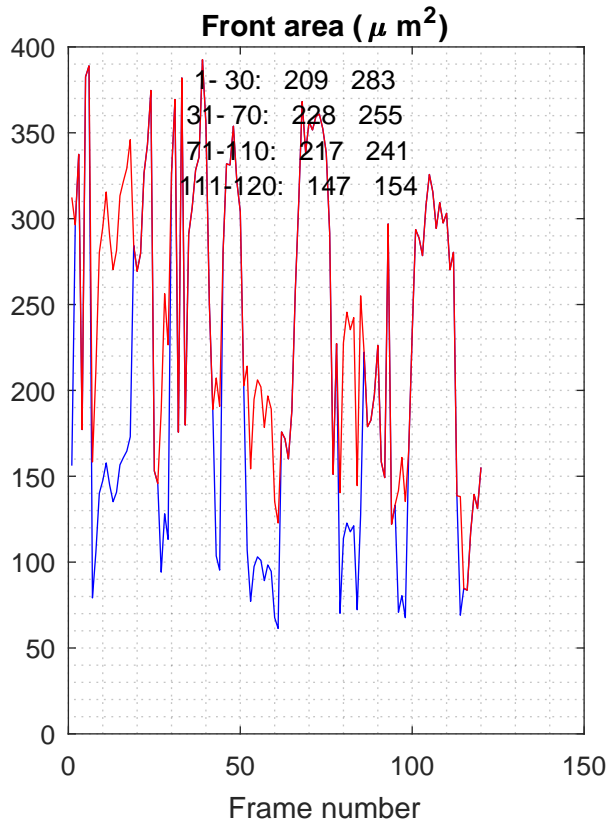

Supplement: Supplementary file 22 — Source Data for Figure 4 [file MSB-15-e8585-s020.zip › Source_data_for_Figure_4/Fig_4I/ChR17-30.pdf]

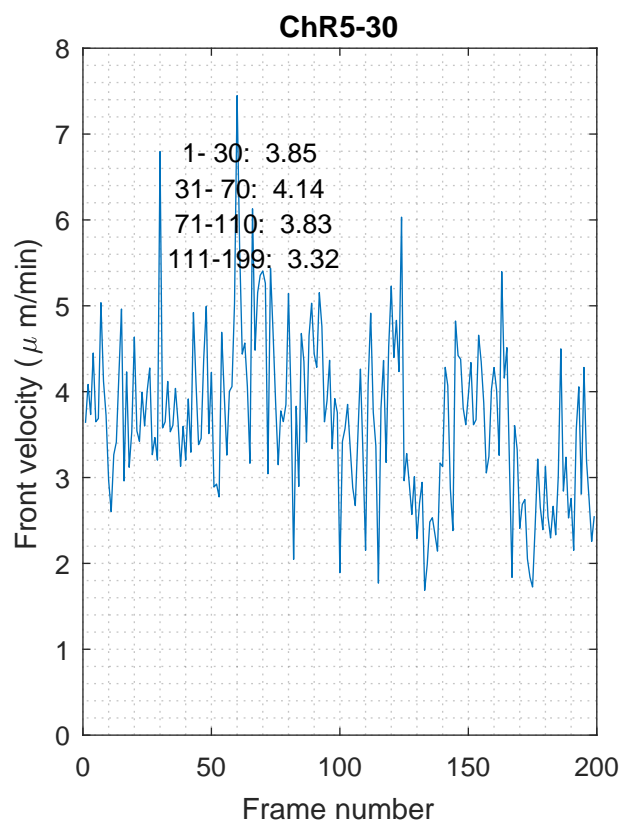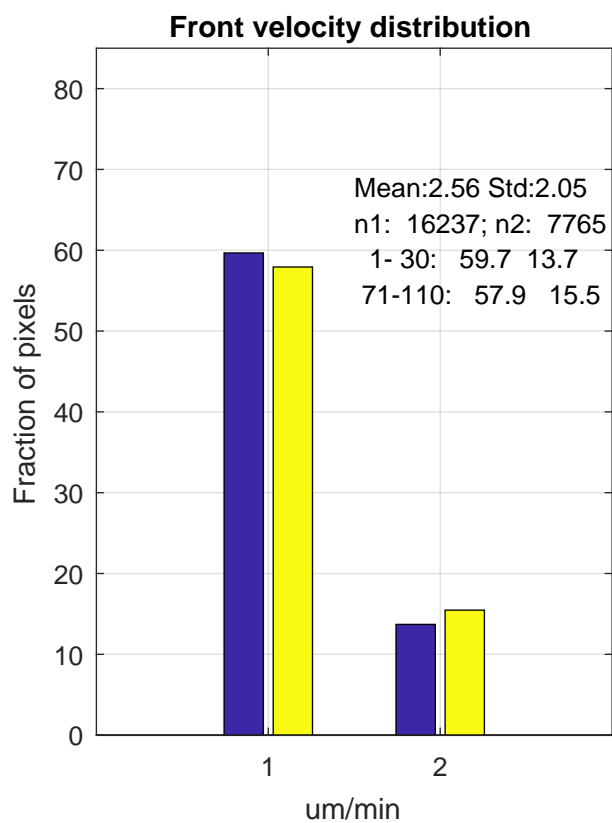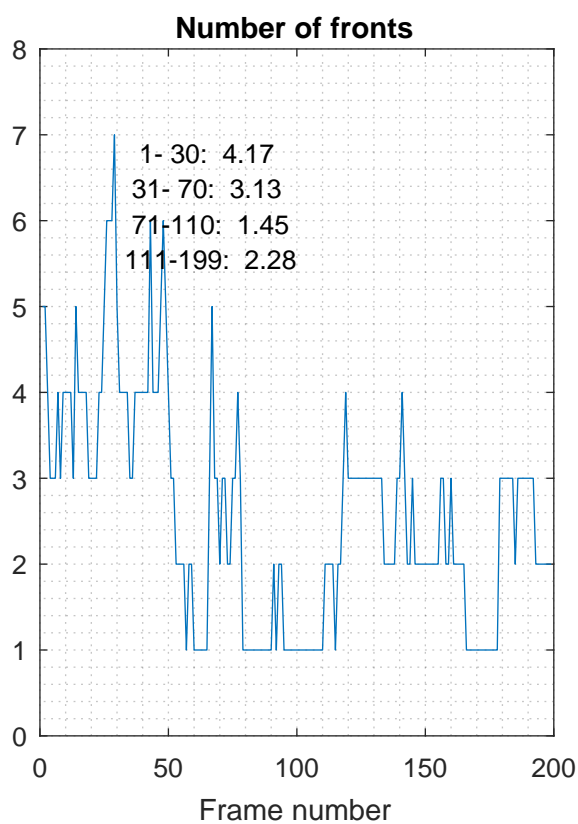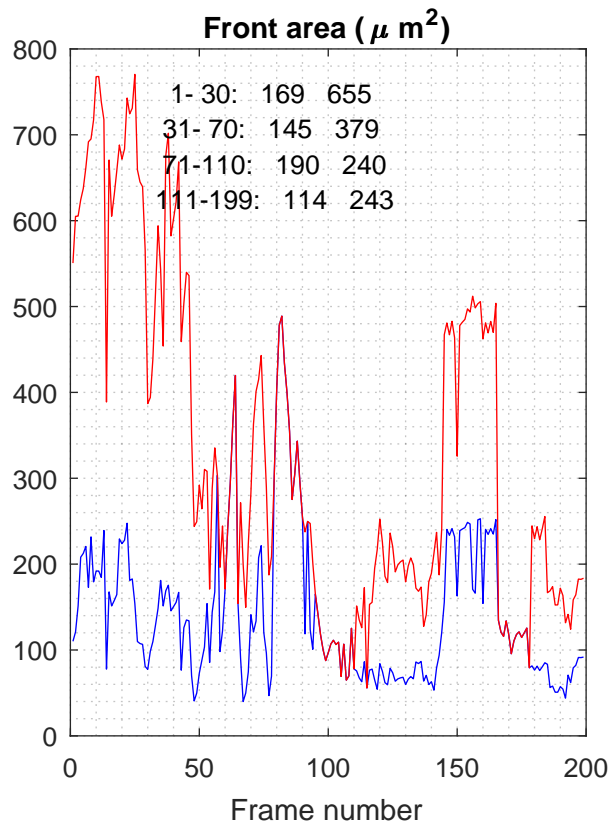

Supplement: Supplementary file 22 — Source Data for Figure 4 [file MSB-15-e8585-s020.zip › Source_data_for_Figure_4/Fig_4I/ChR5-30.pdf]

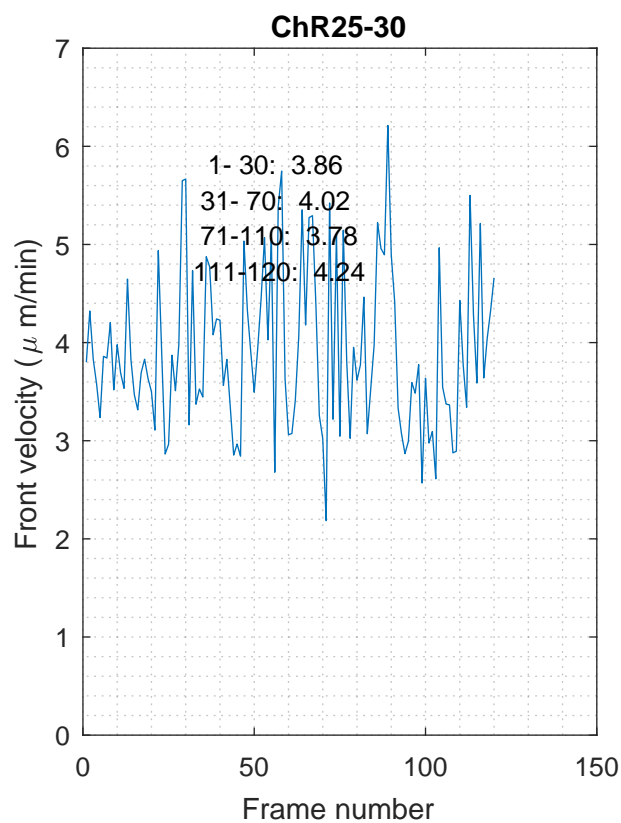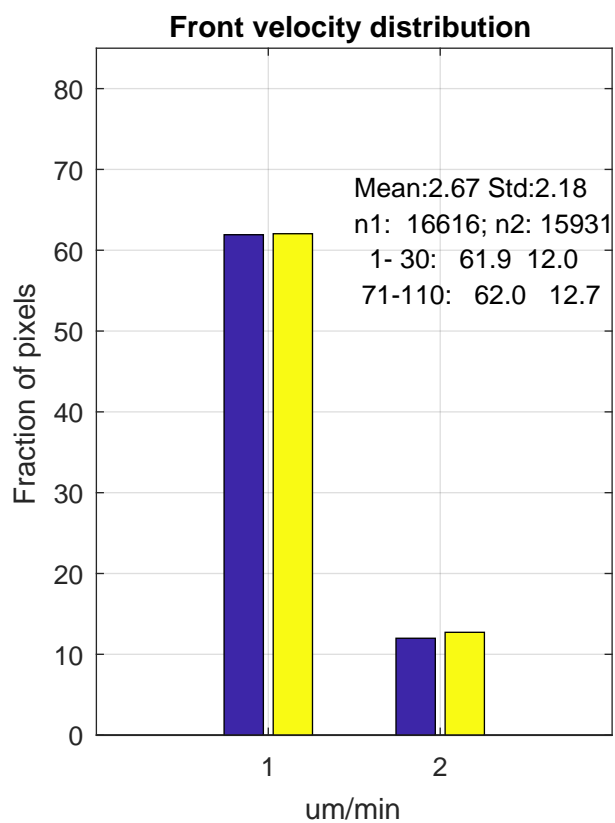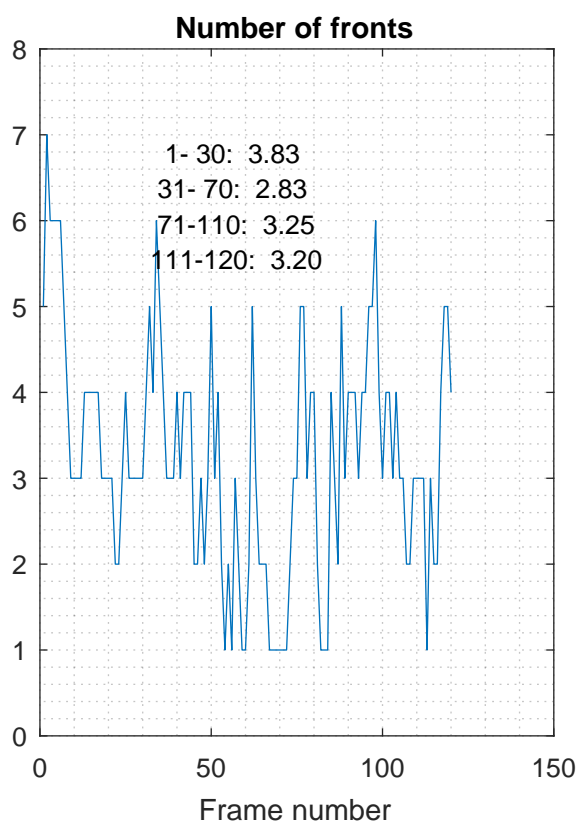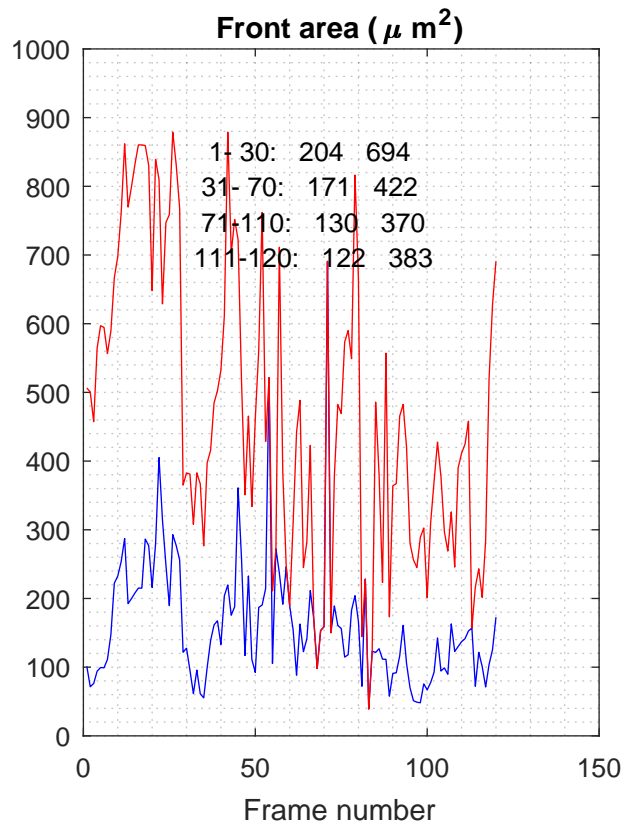

Supplement: Supplementary file 22 — Source Data for Figure 4 [file MSB-15-e8585-s020.zip › Source_data_for_Figure_4/Fig_4I/ChR25-30.pdf]

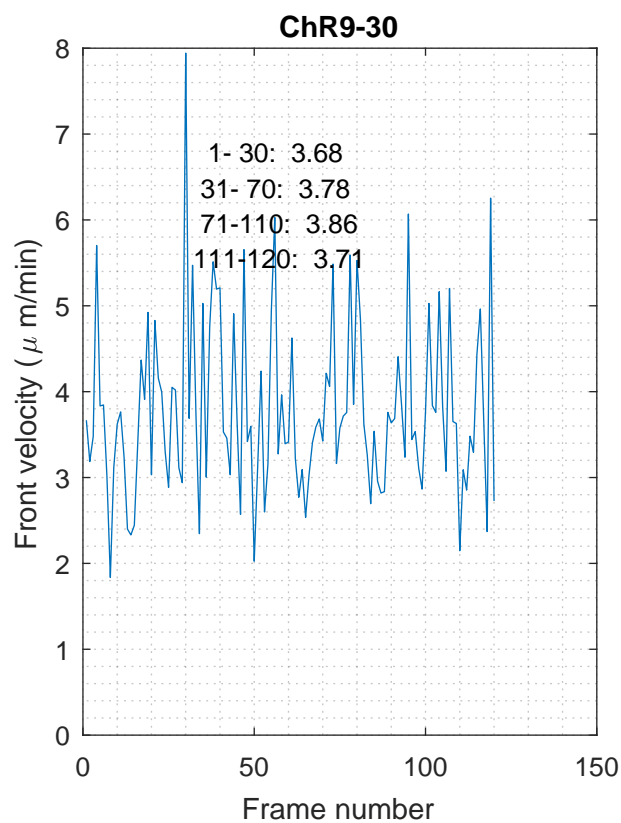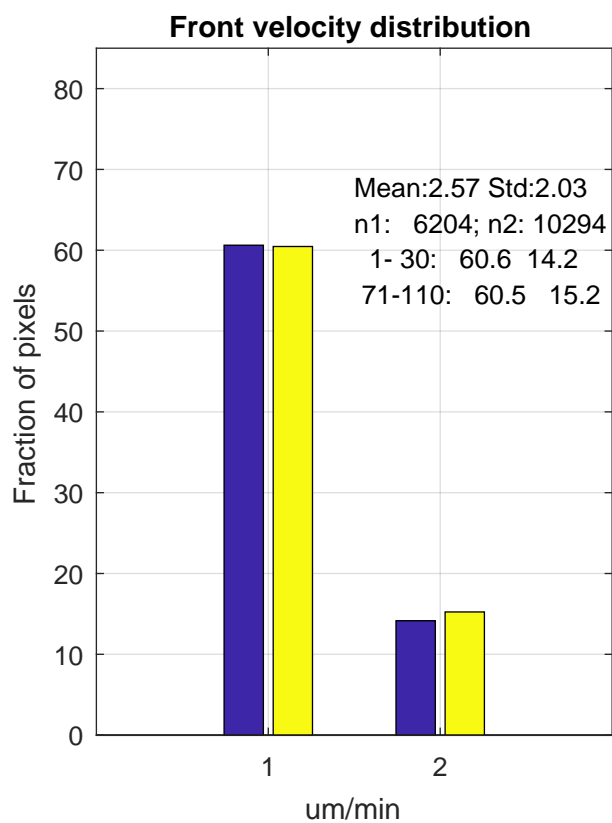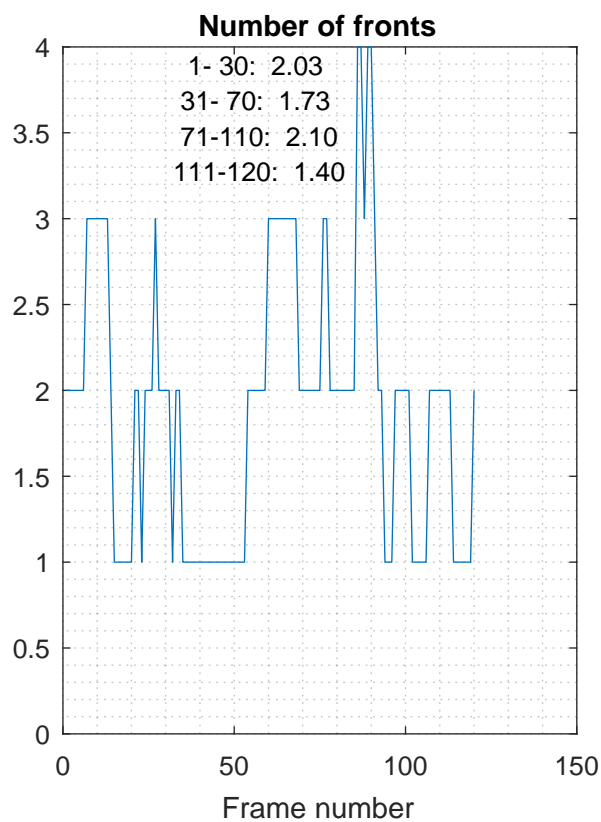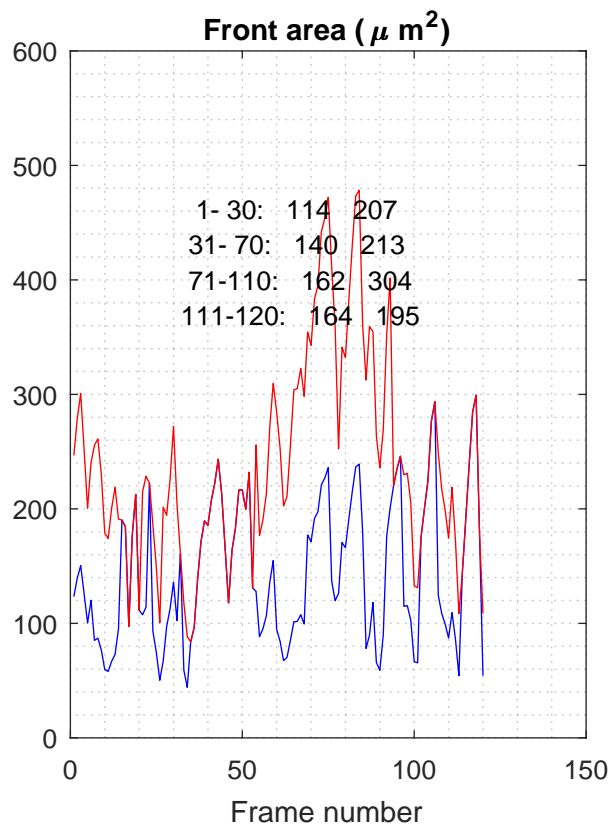

Supplement: Supplementary file 22 — Source Data for Figure 4 [file MSB-15-e8585-s020.zip › Source_data_for_Figure_4/Fig_4I/ChR9-30.pdf]

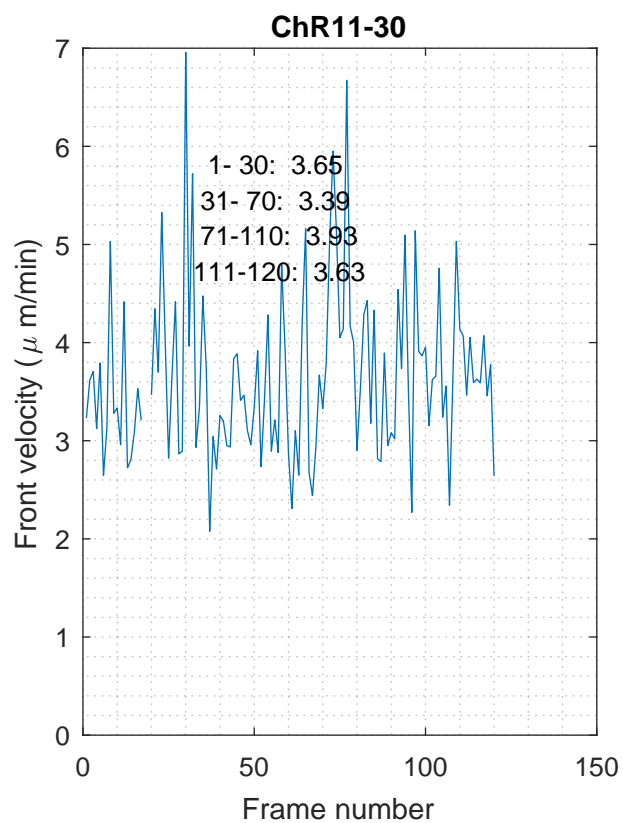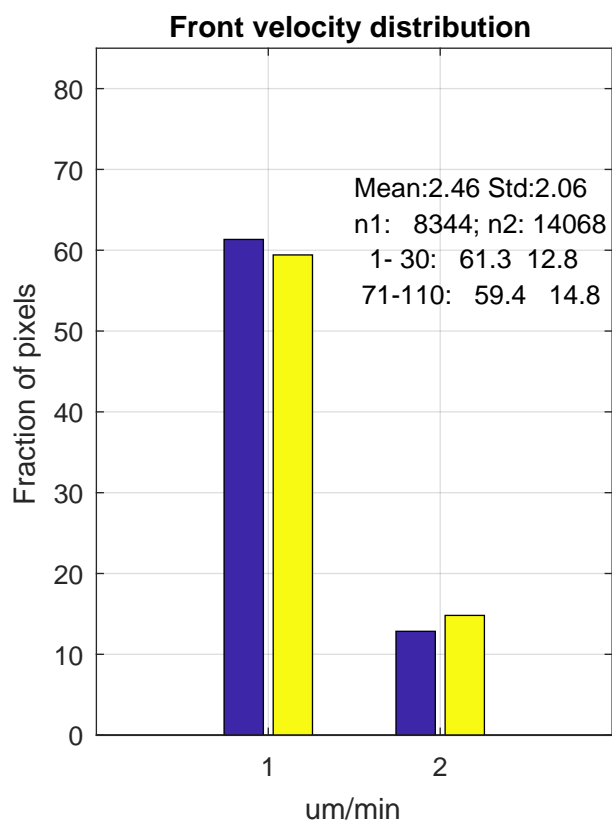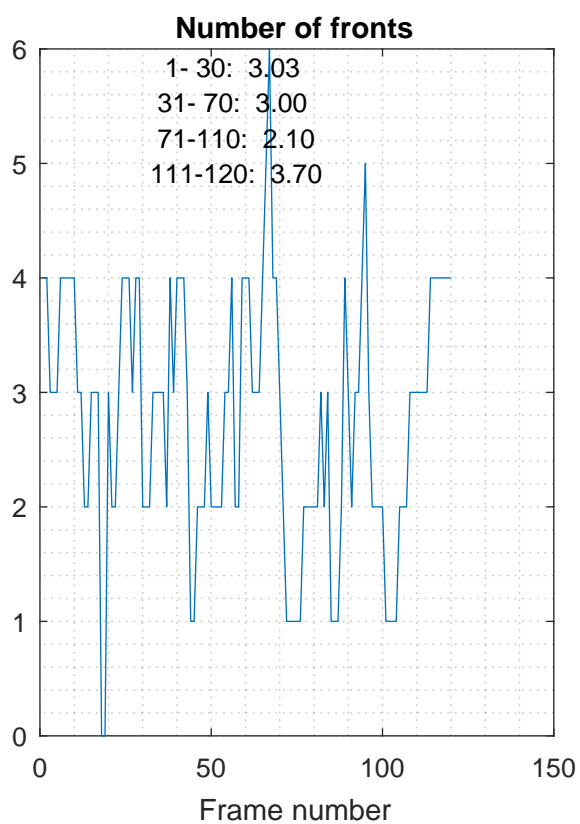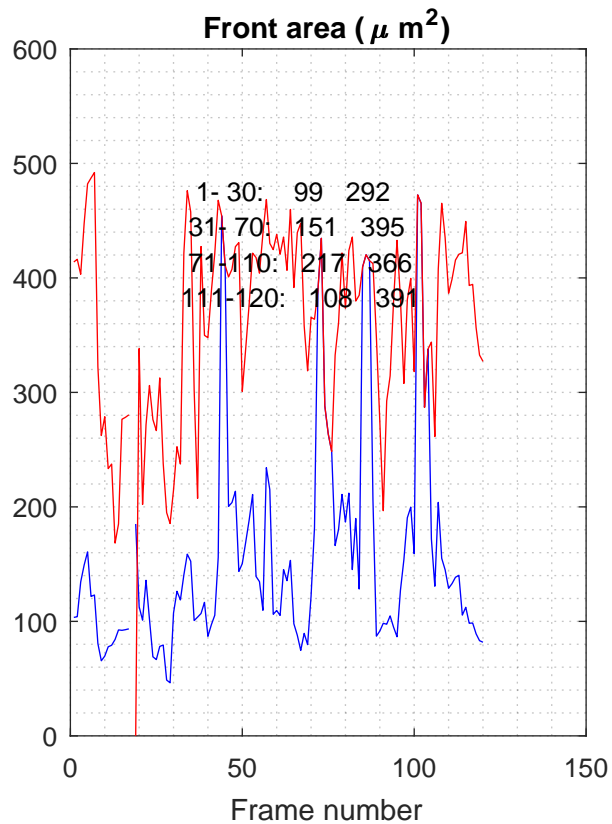

Supplement: Supplementary file 22 — Source Data for Figure 4 [file MSB-15-e8585-s020.zip › Source_data_for_Figure_4/Fig_4I/ChR11-30.pdf]

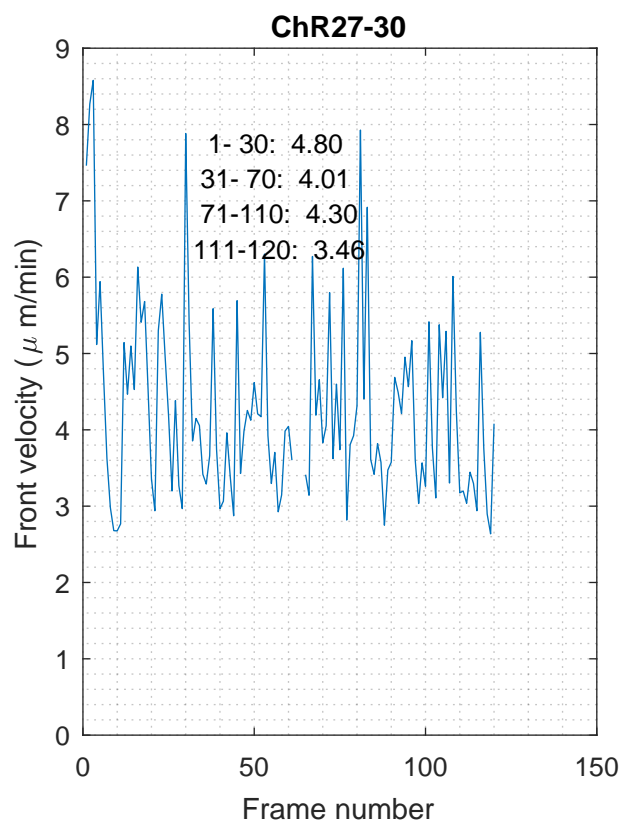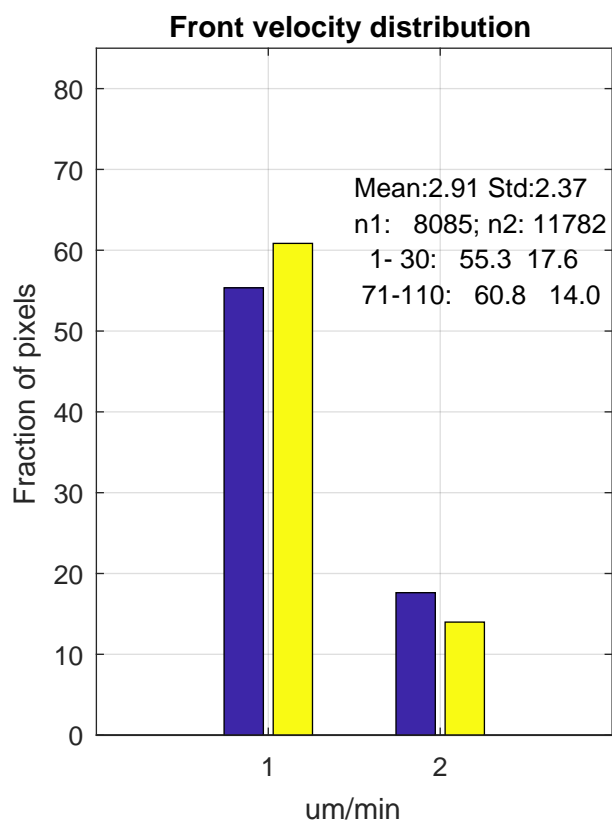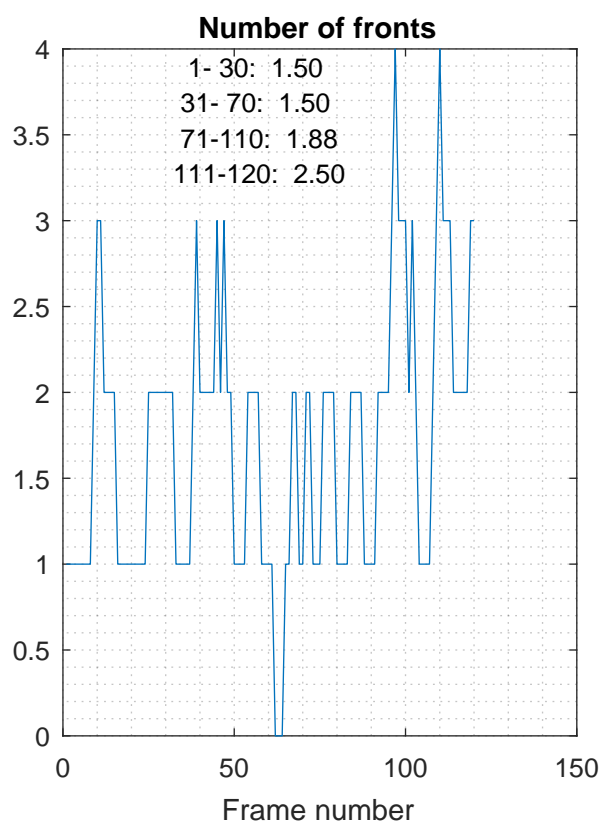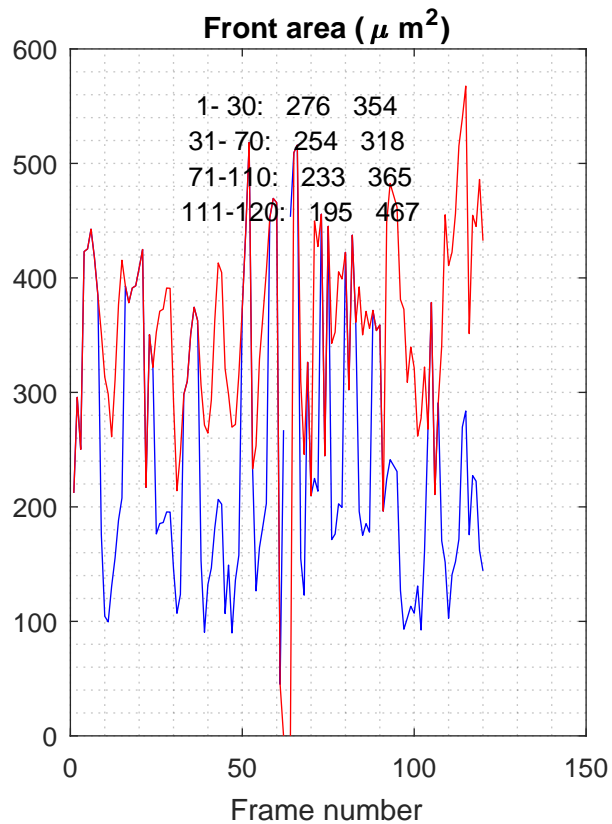

Supplement: Supplementary file 22 — Source Data for Figure 4 [file MSB-15-e8585-s020.zip › Source_data_for_Figure_4/Fig_4I/ChR27-30.pdf]

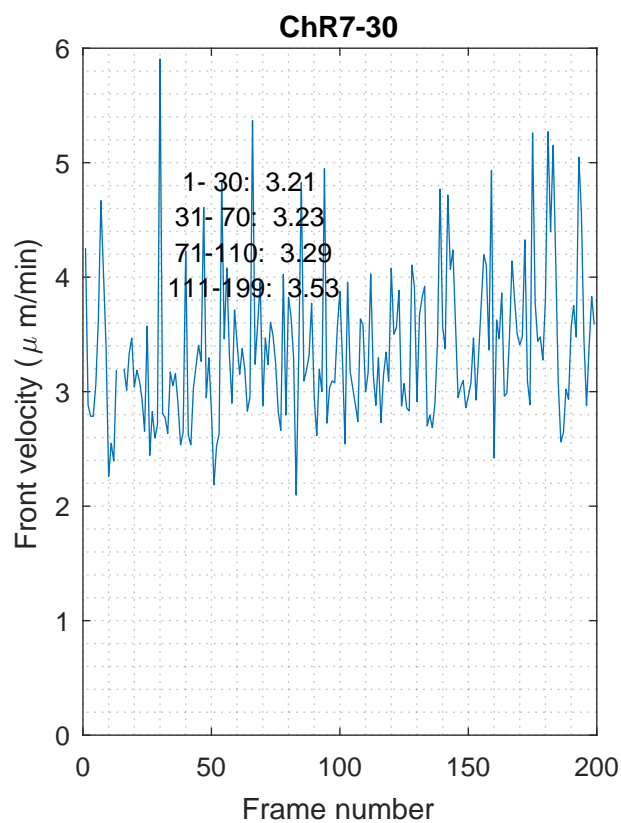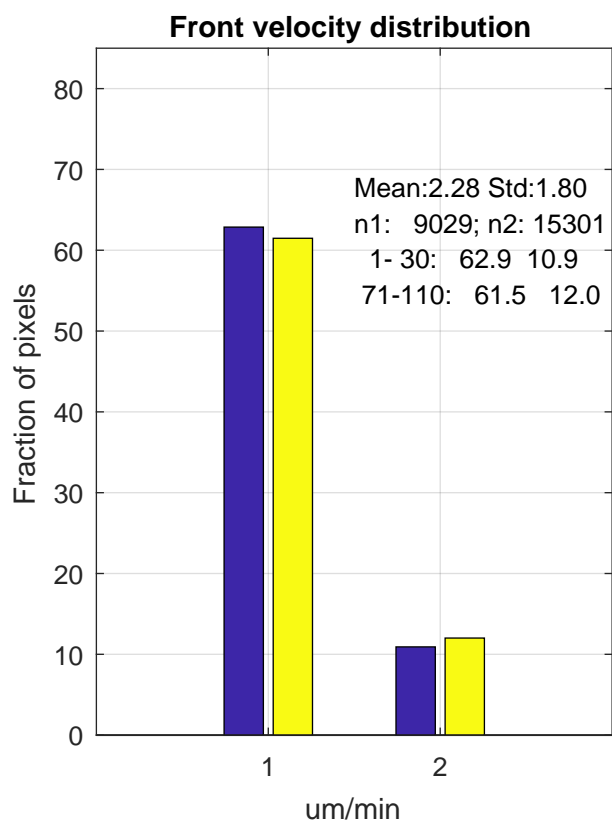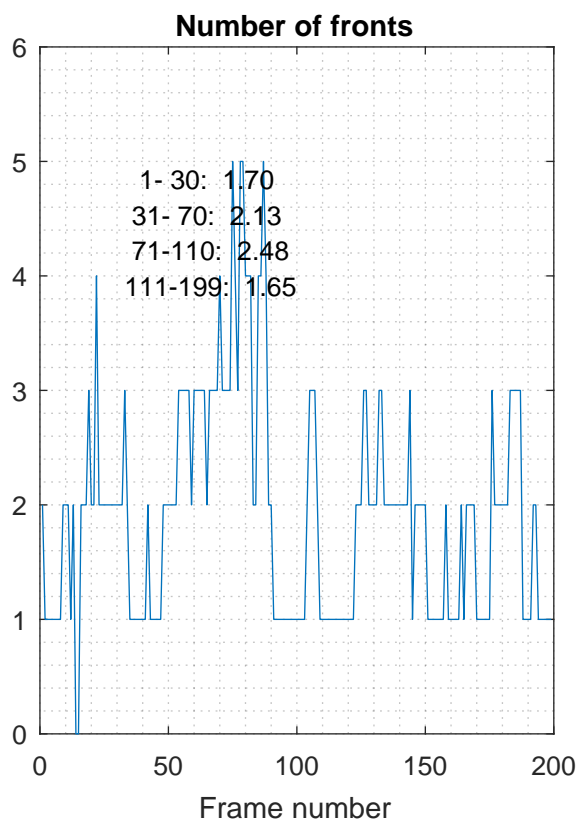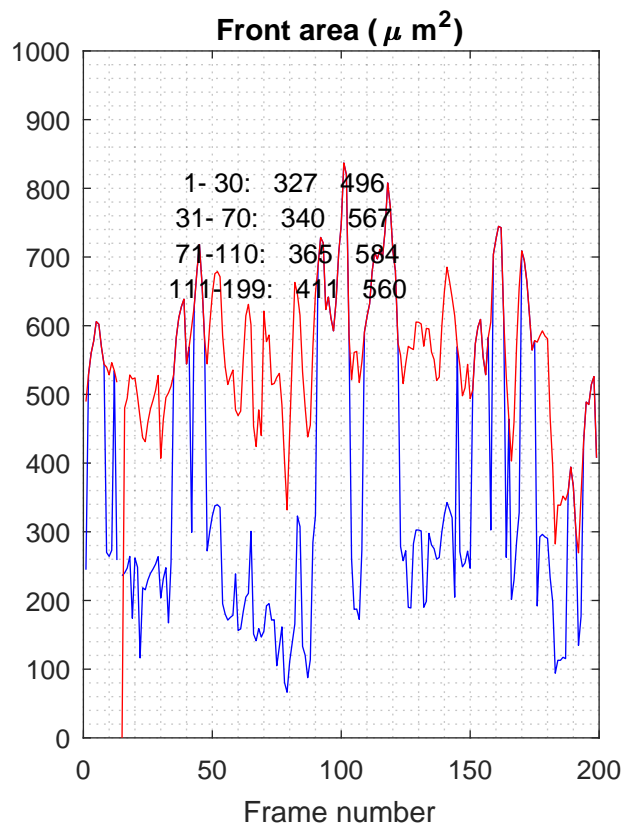

Supplement: Supplementary file 22 — Source Data for Figure 4 [file MSB-15-e8585-s020.zip › Source_data_for_Figure_4/Fig_4I/ChR7-30.pdf]

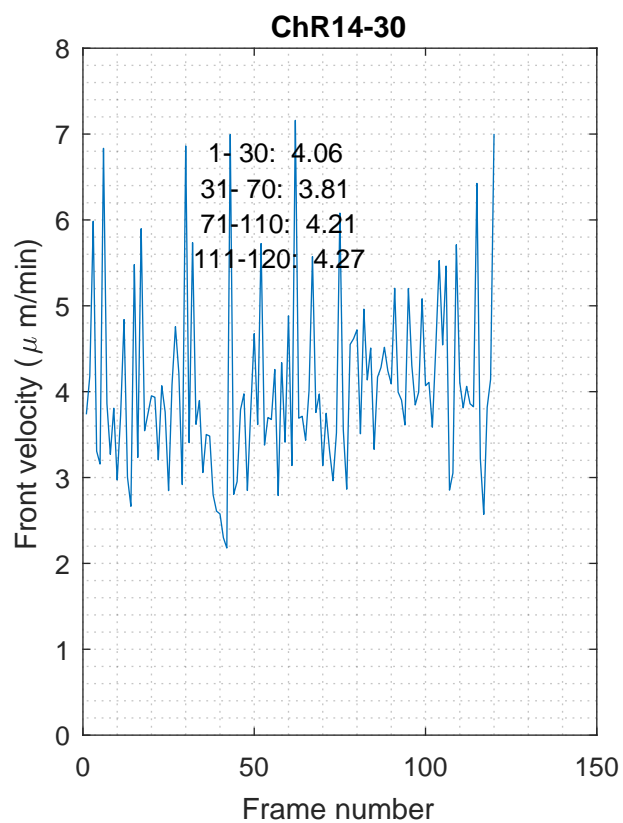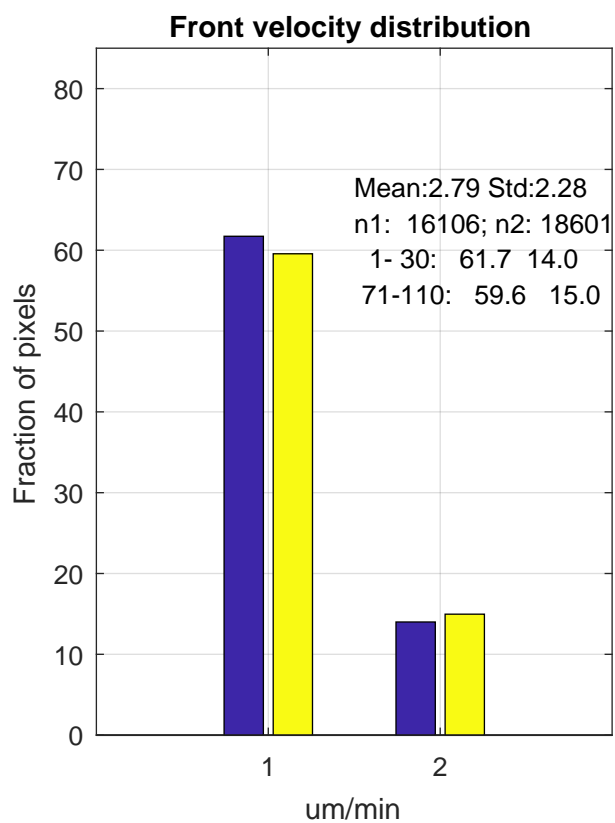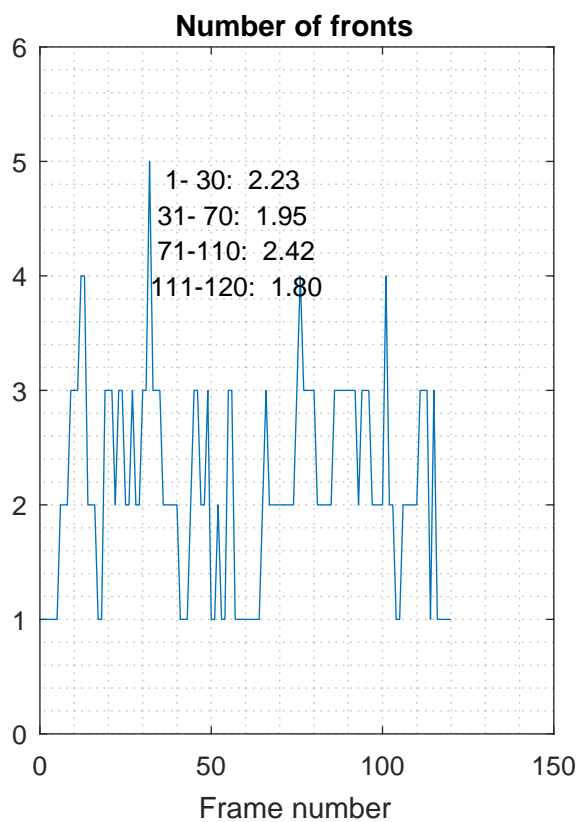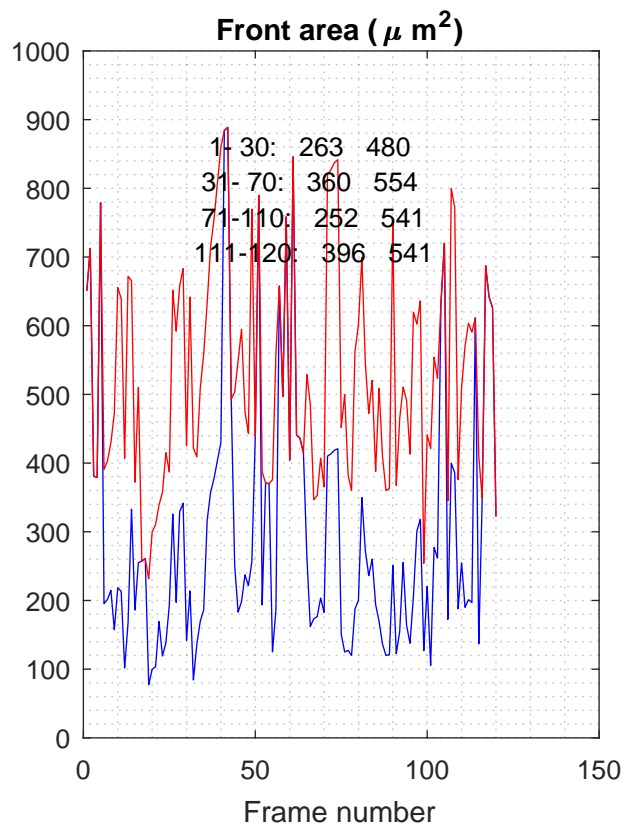

Supplement: Supplementary file 22 — Source Data for Figure 4 [file MSB-15-e8585-s020.zip › Source_data_for_Figure_4/Fig_4I/ChR14-30.pdf]

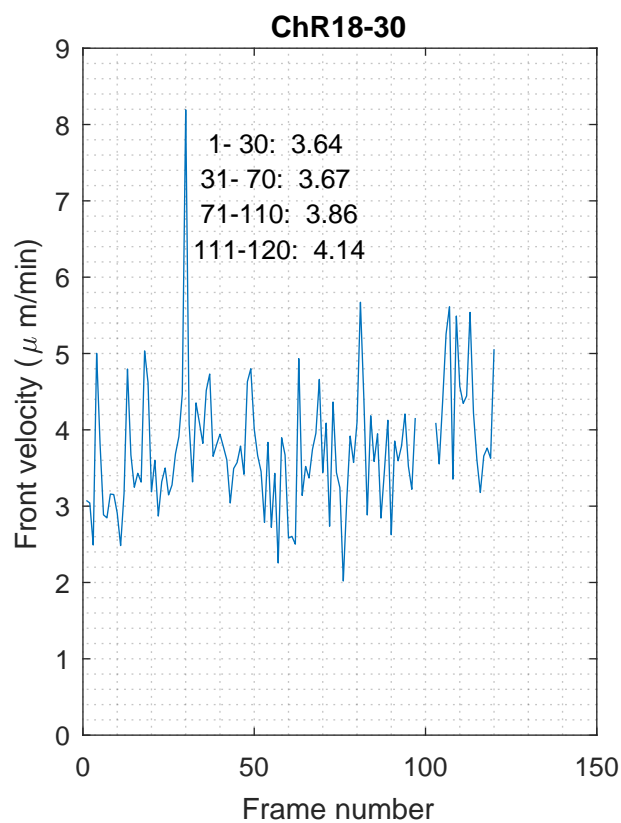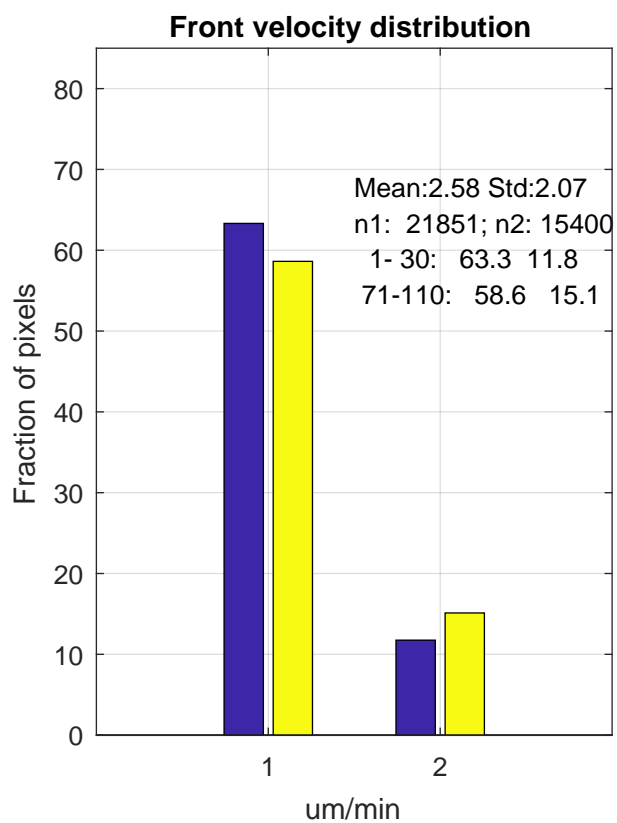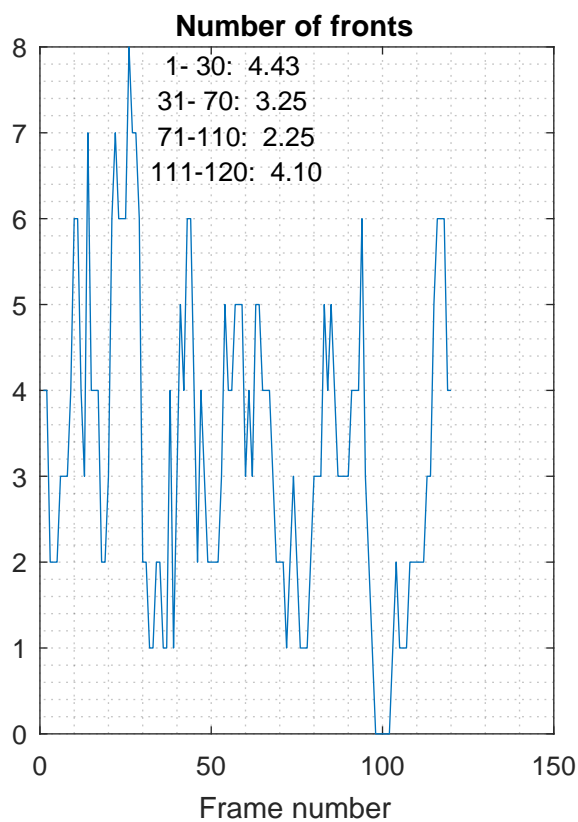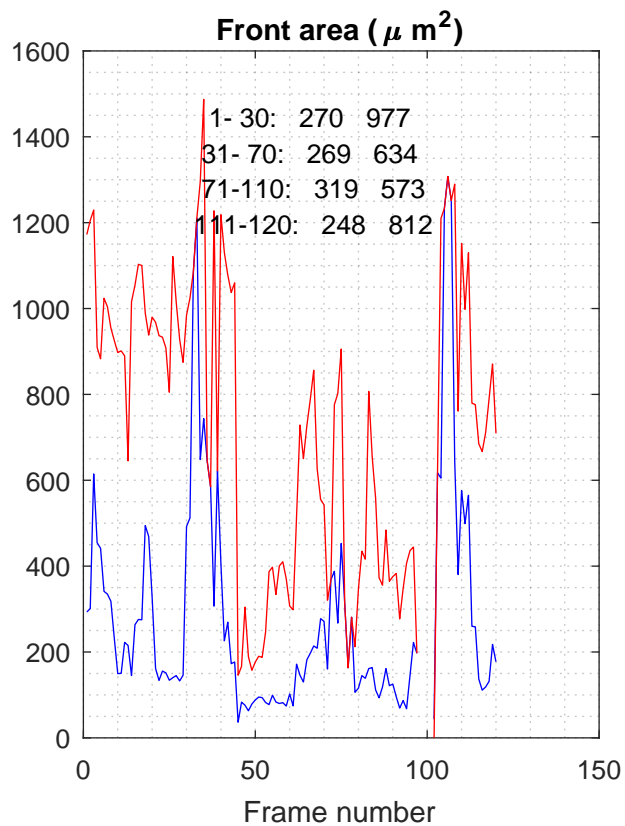

Supplement: Supplementary file 22 — Source Data for Figure 4 [file MSB-15-e8585-s020.zip › Source_data_for_Figure_4/Fig_4I/ChR18-30.pdf]

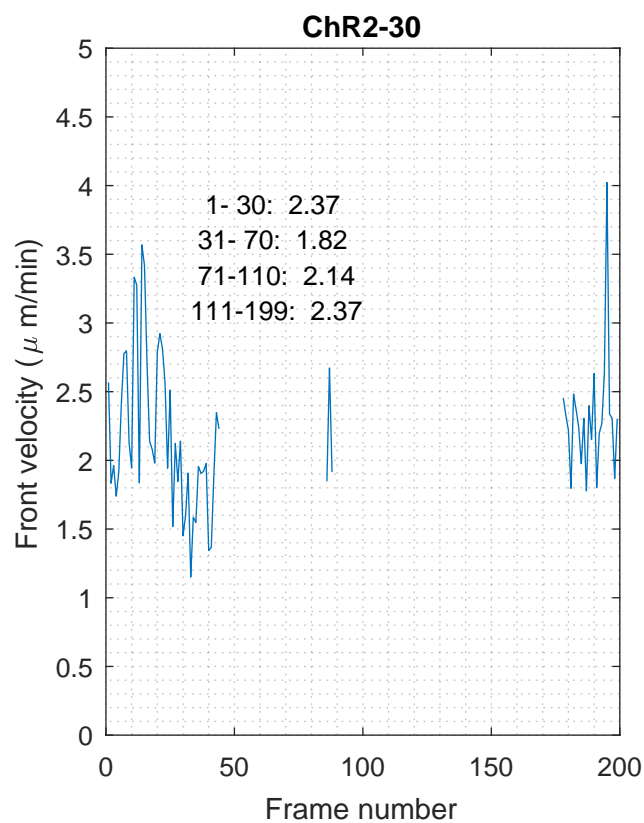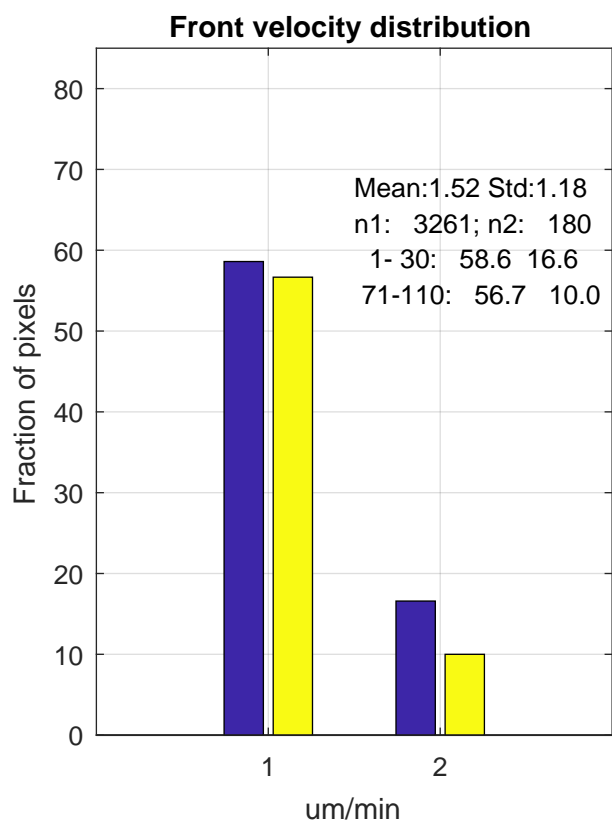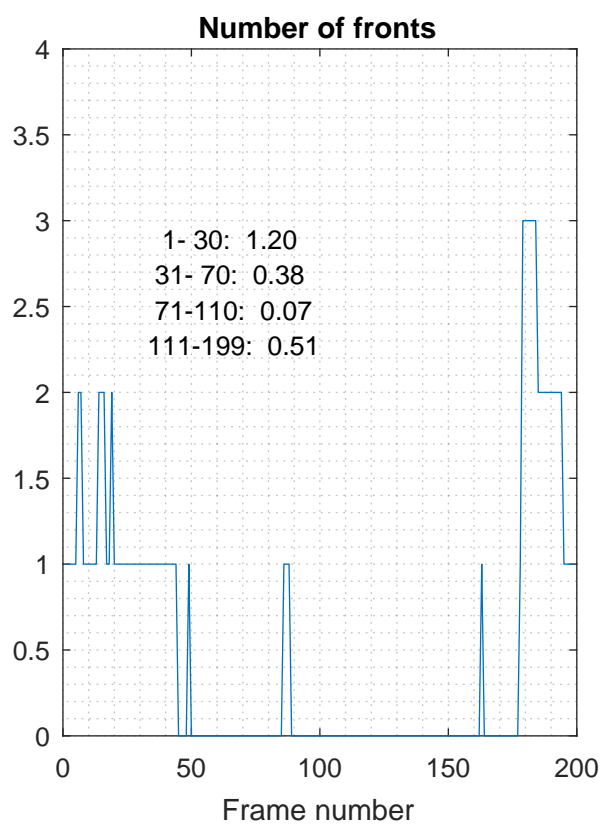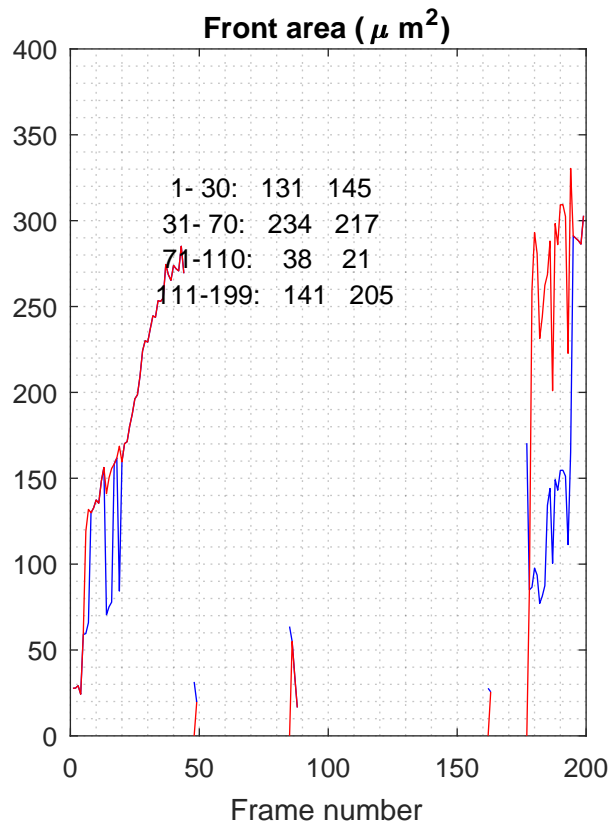

Supplement: Supplementary file 22 — Source Data for Figure 4 [file MSB-15-e8585-s020.zip › Source_data_for_Figure_4/Fig_4I/ChR2-30.pdf]

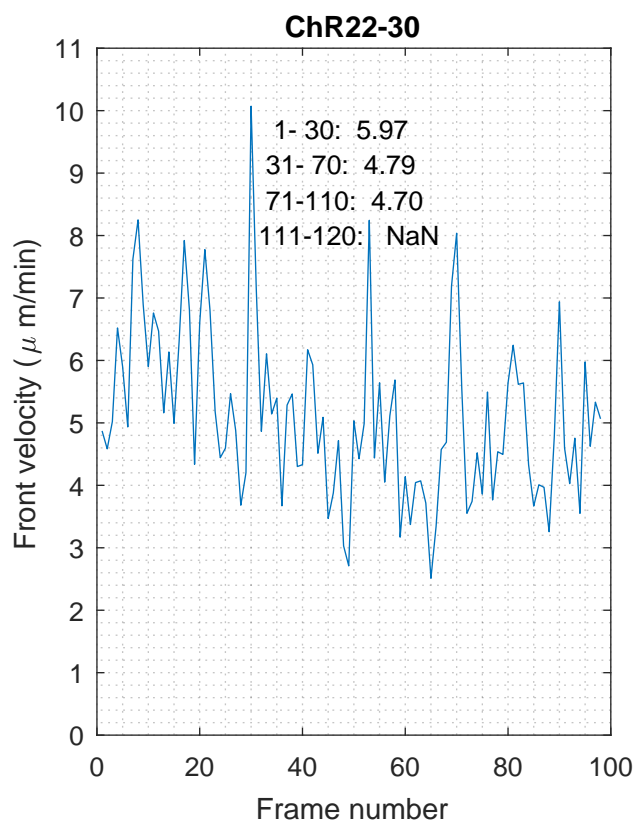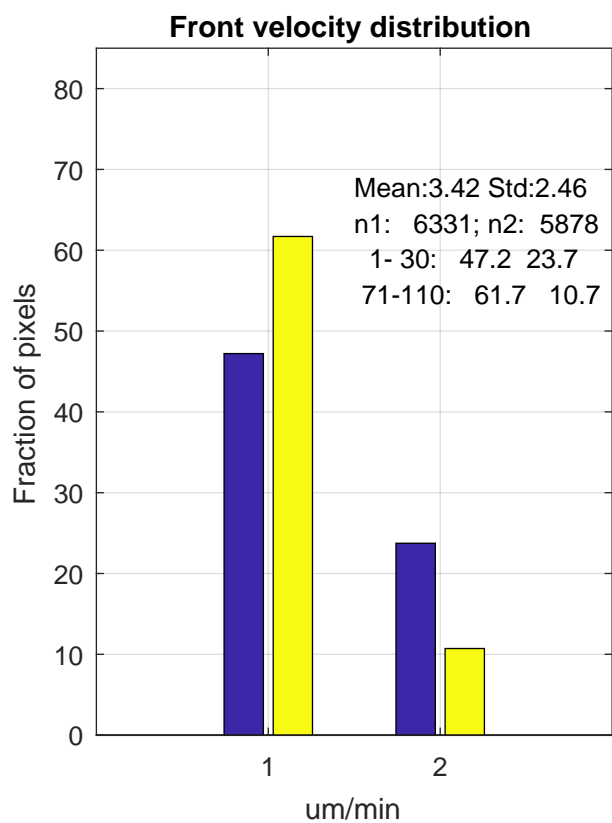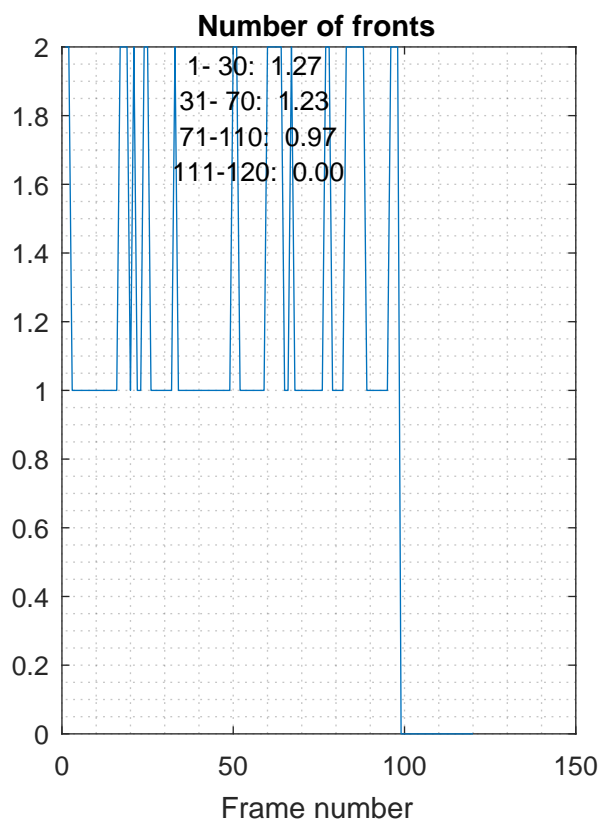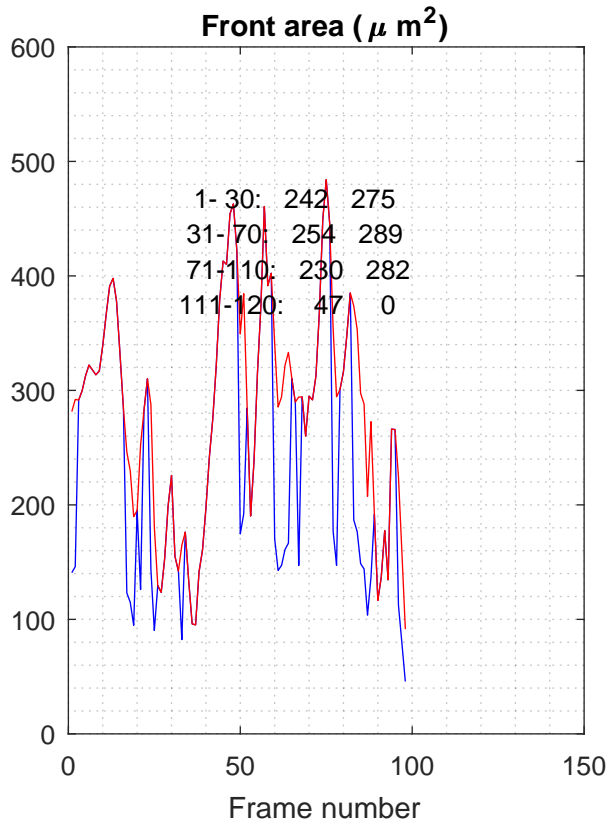

Supplement: Supplementary file 22 — Source Data for Figure 4 [file MSB-15-e8585-s020.zip › Source_data_for_Figure_4/Fig_4I/ChR22-30.pdf]

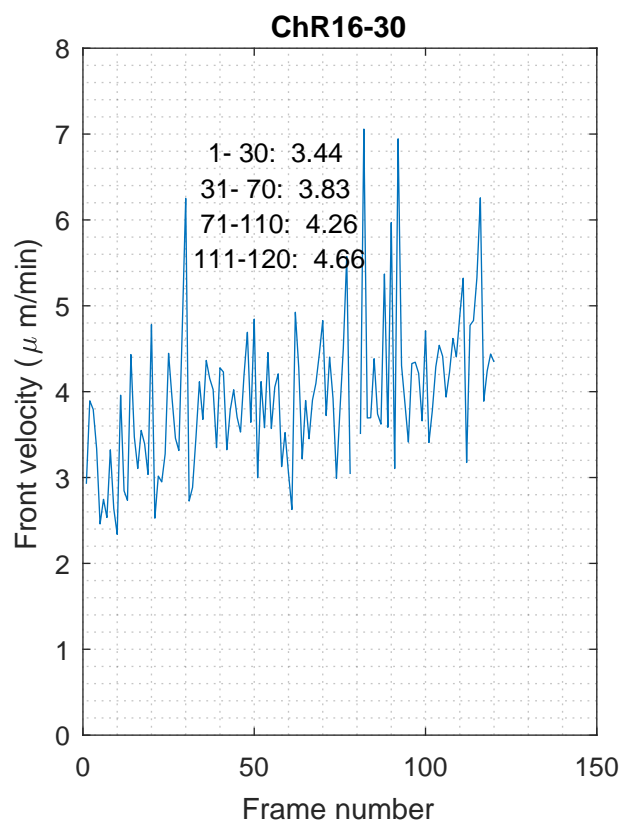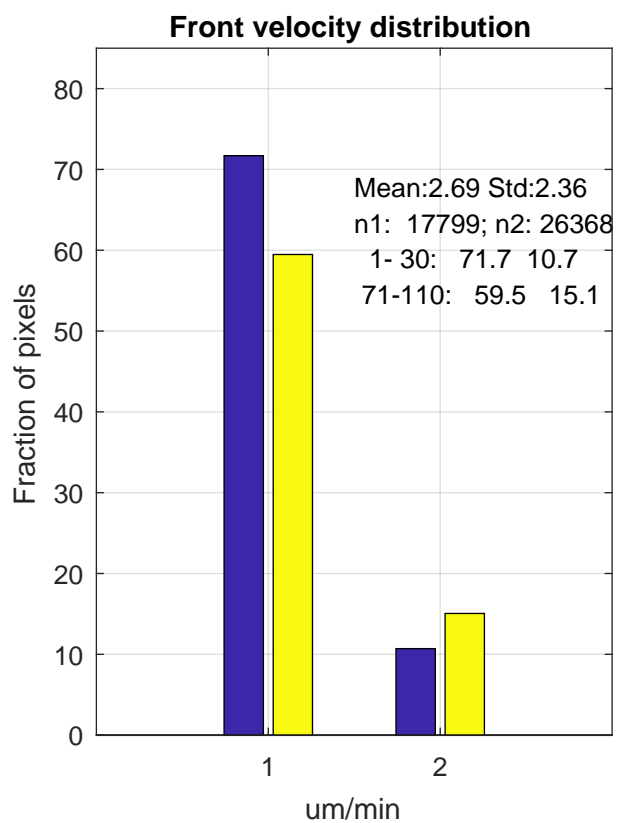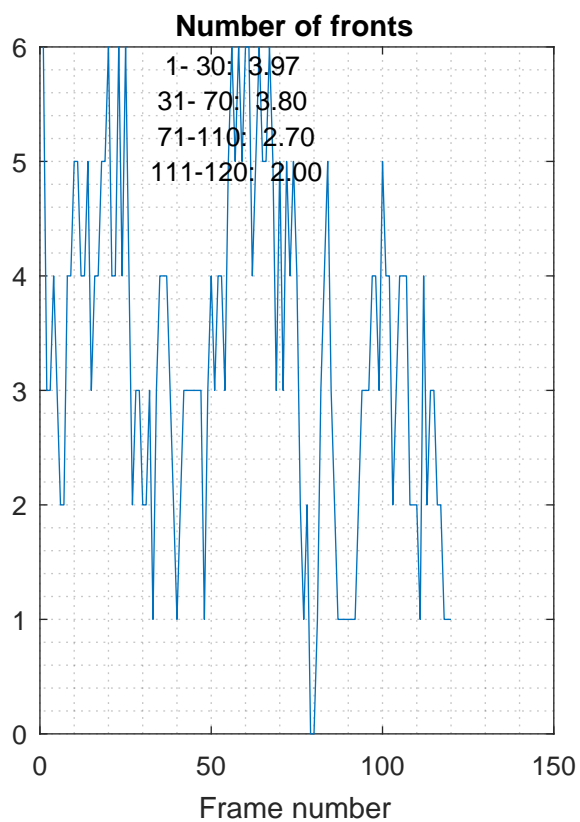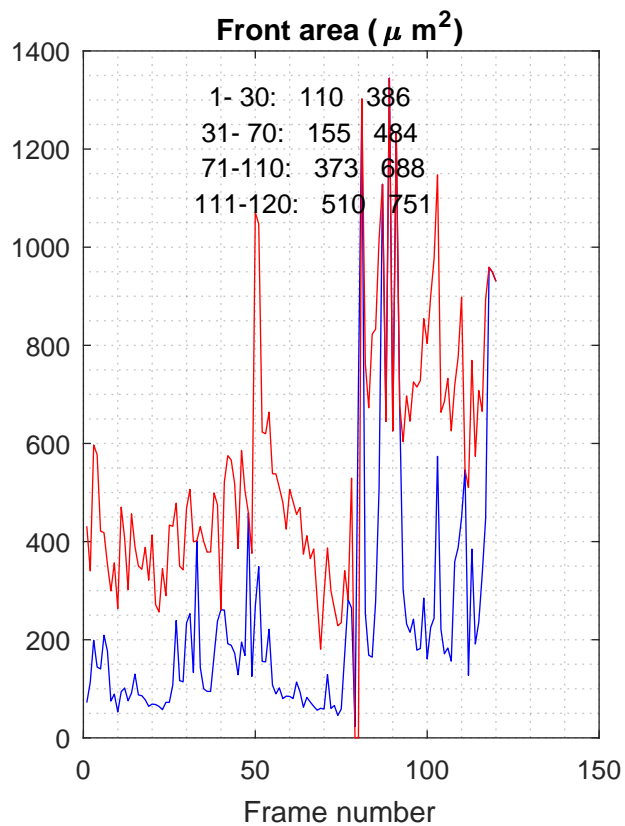

Supplement: Supplementary file 22 — Source Data for Figure 4 [file MSB-15-e8585-s020.zip › Source_data_for_Figure_4/Fig_4I/ChR16-30.pdf]

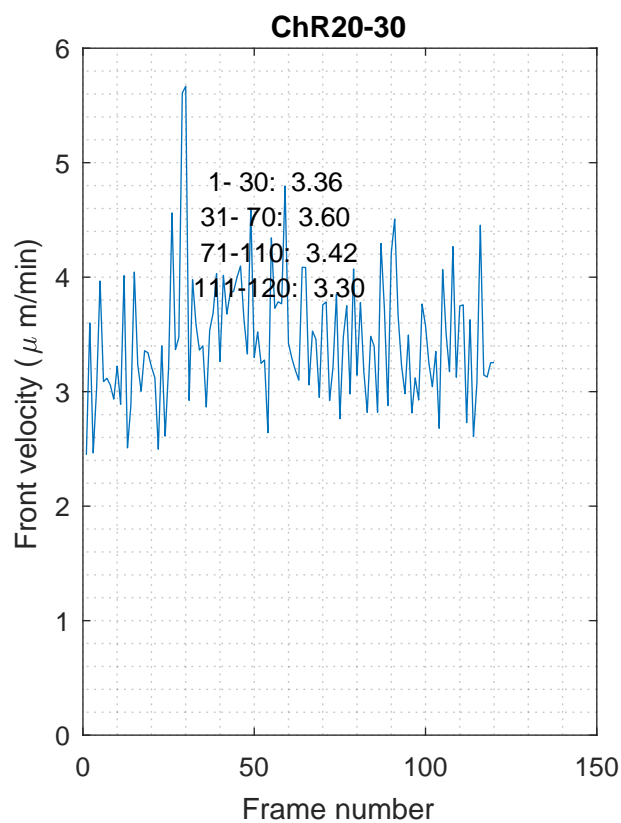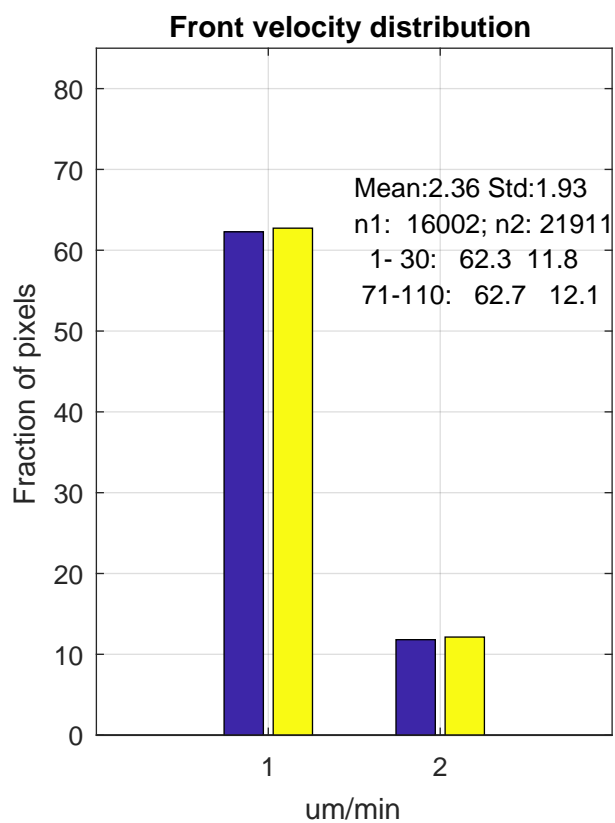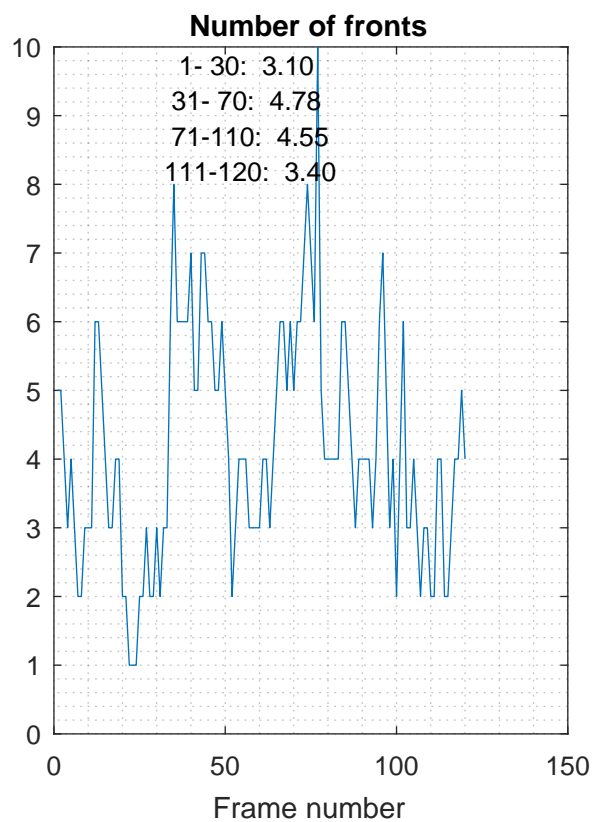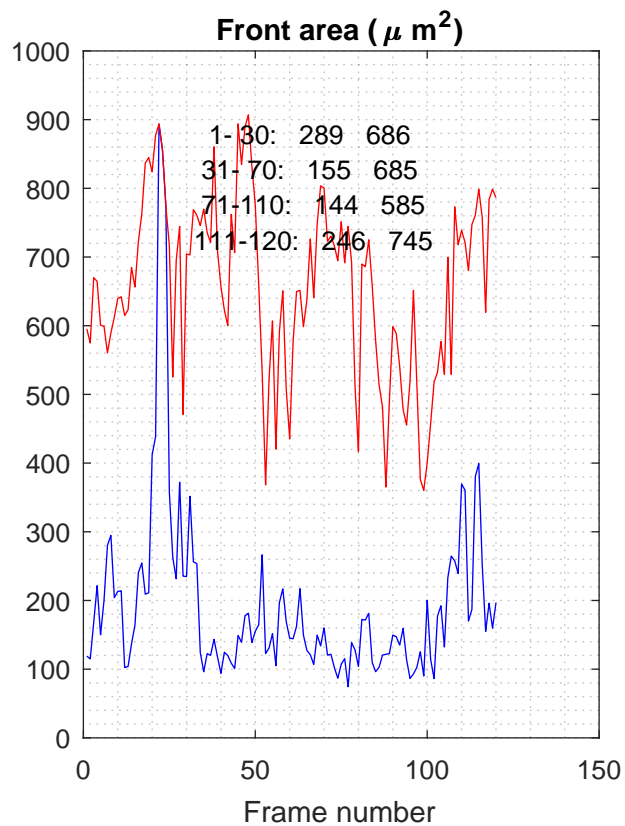

Supplement: Supplementary file 22 — Source Data for Figure 4 [file MSB-15-e8585-s020.zip › Source_data_for_Figure_4/Fig_4I/ChR20-30.pdf]

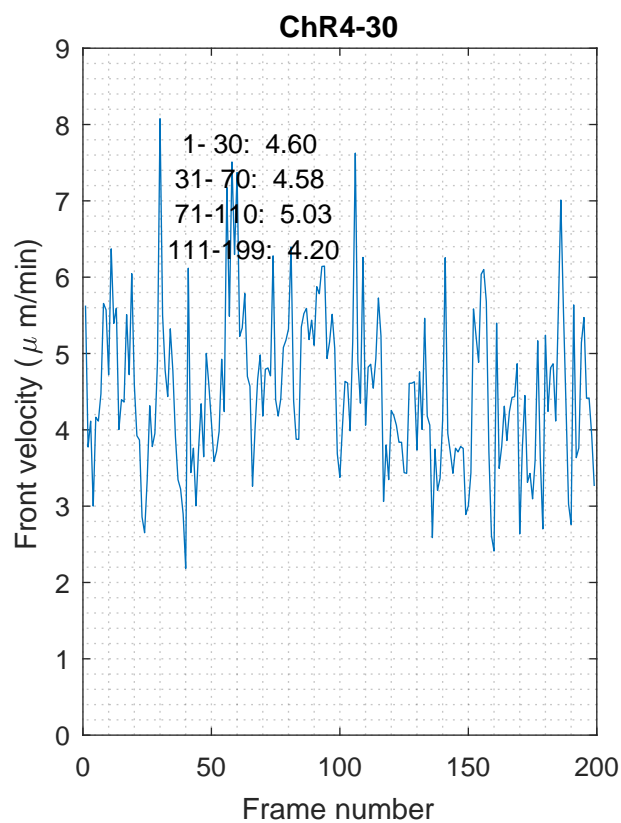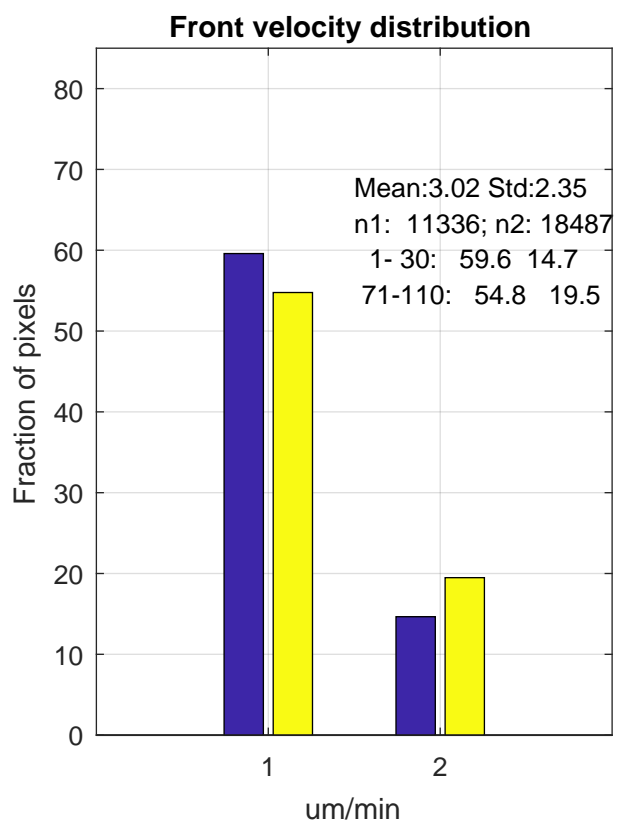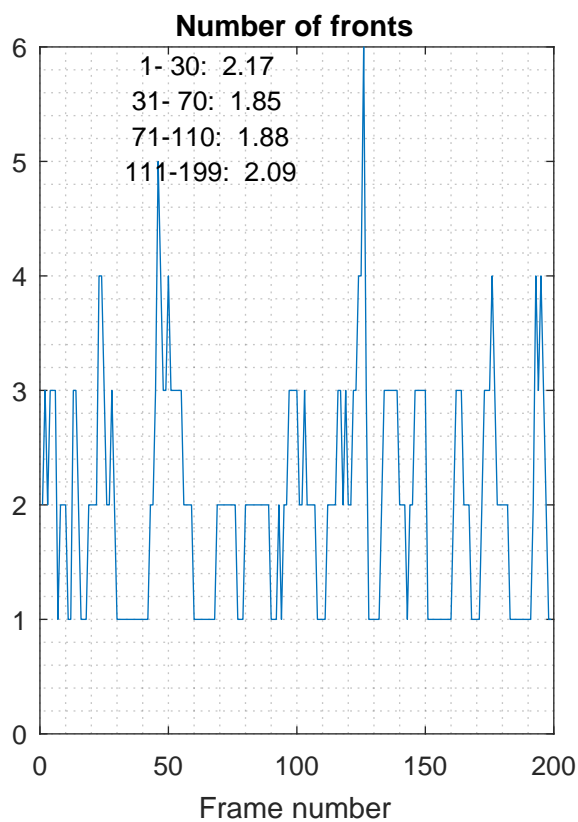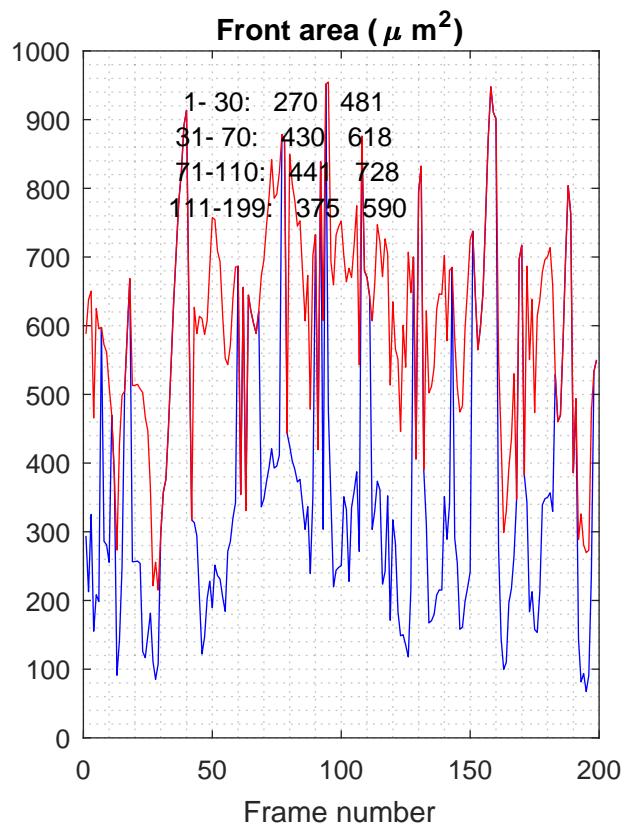

Supplement: Supplementary file 22 — Source Data for Figure 4 [file MSB-15-e8585-s020.zip › Source_data_for_Figure_4/Fig_4I/ChR4-30.pdf]

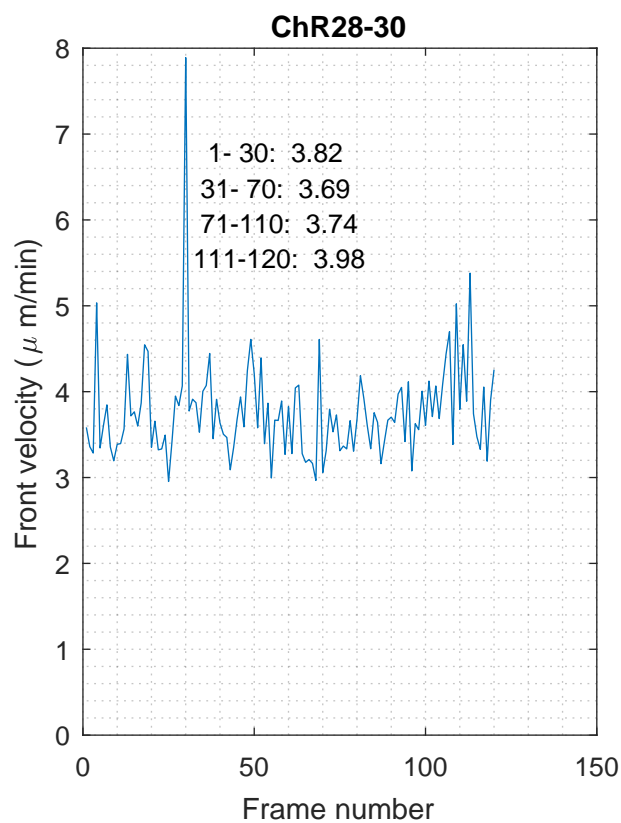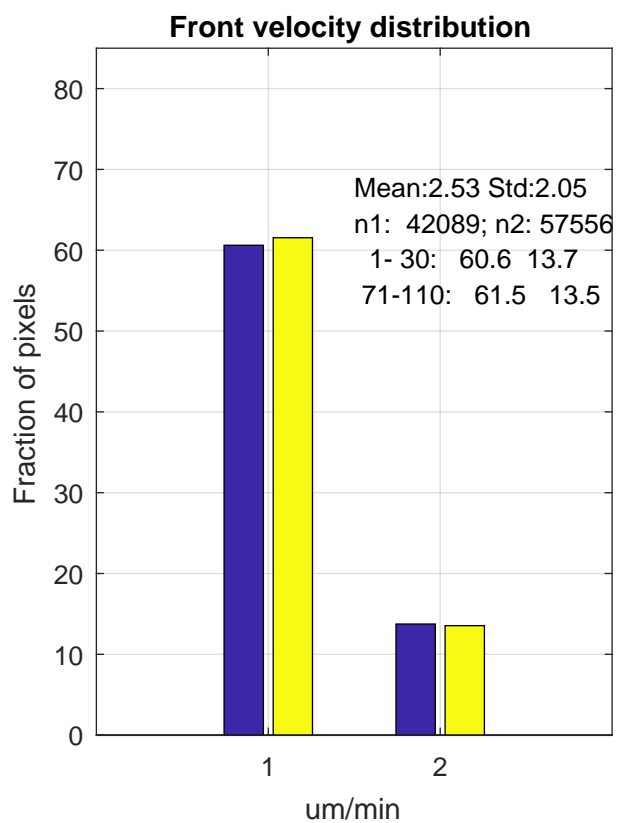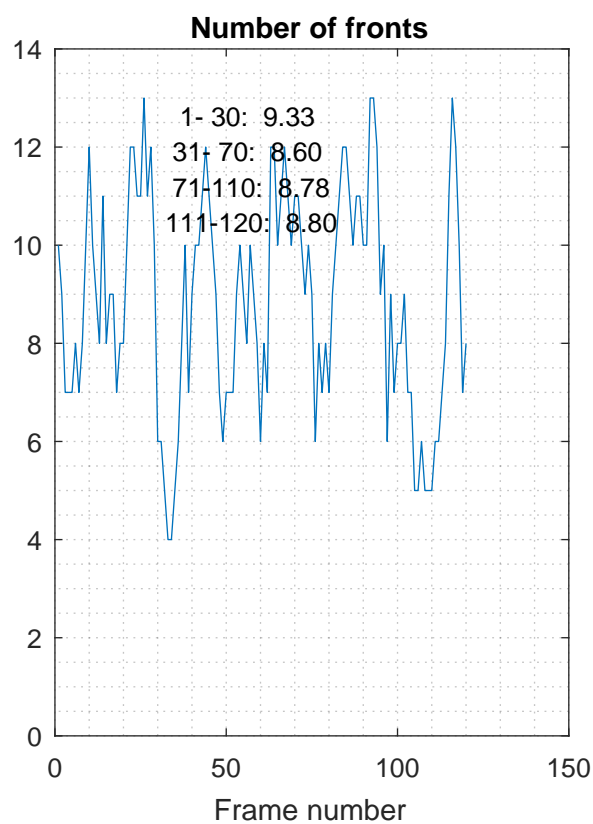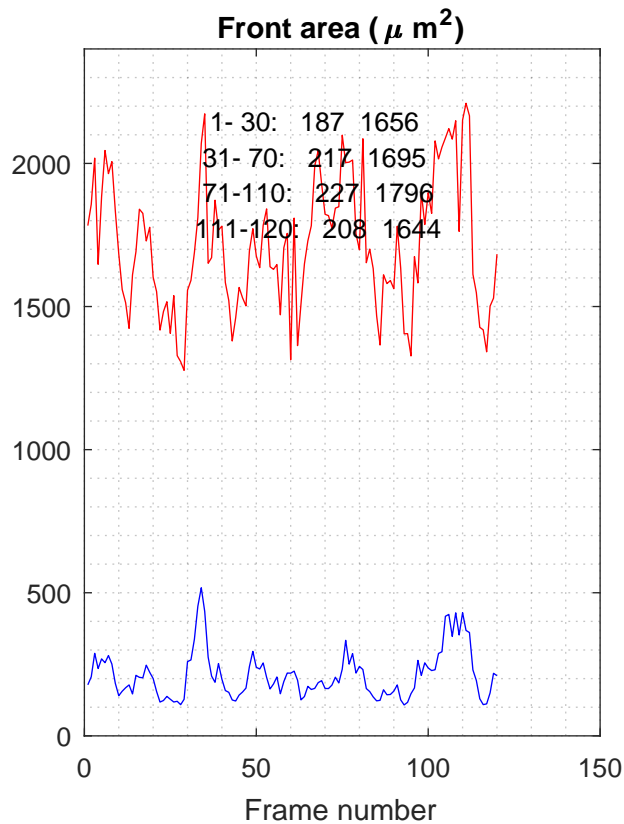

Supplement: Supplementary file 22 — Source Data for Figure 4 [file MSB-15-e8585-s020.zip › Source_data_for_Figure_4/Fig_4I/ChR28-30.pdf]

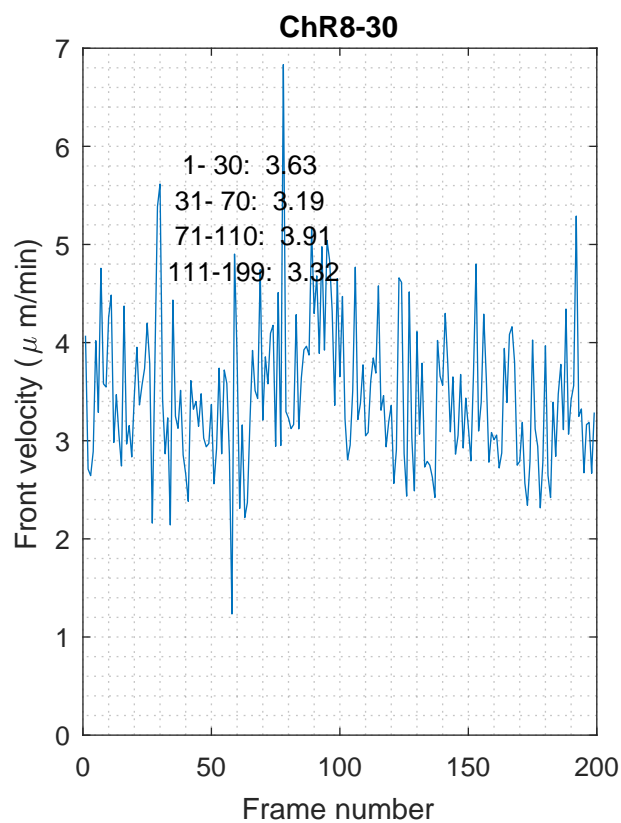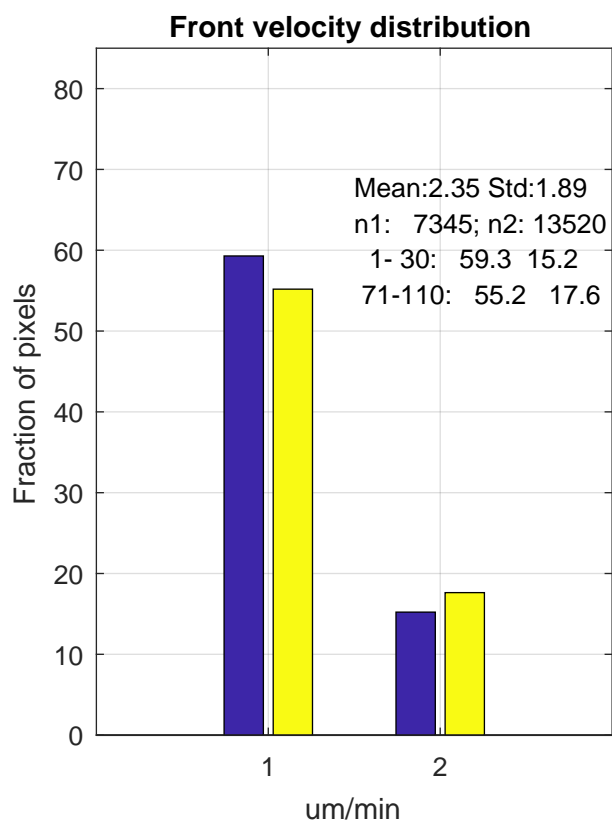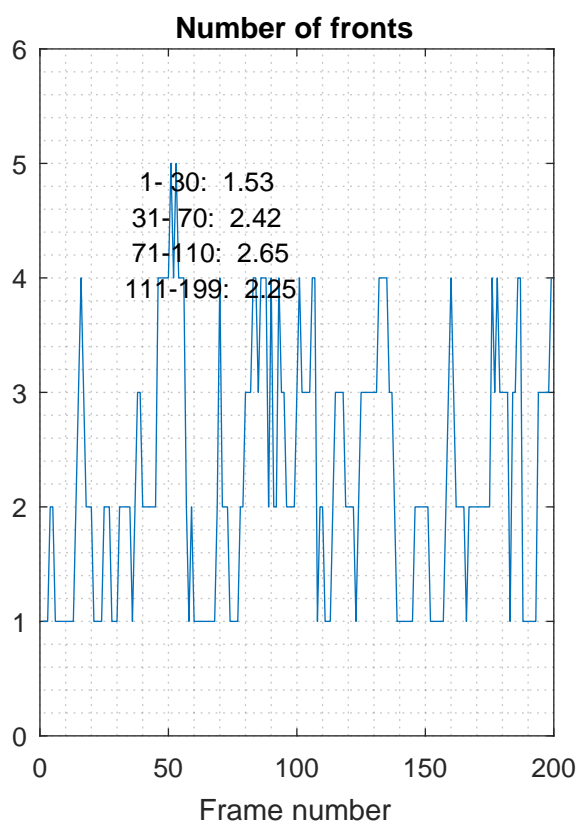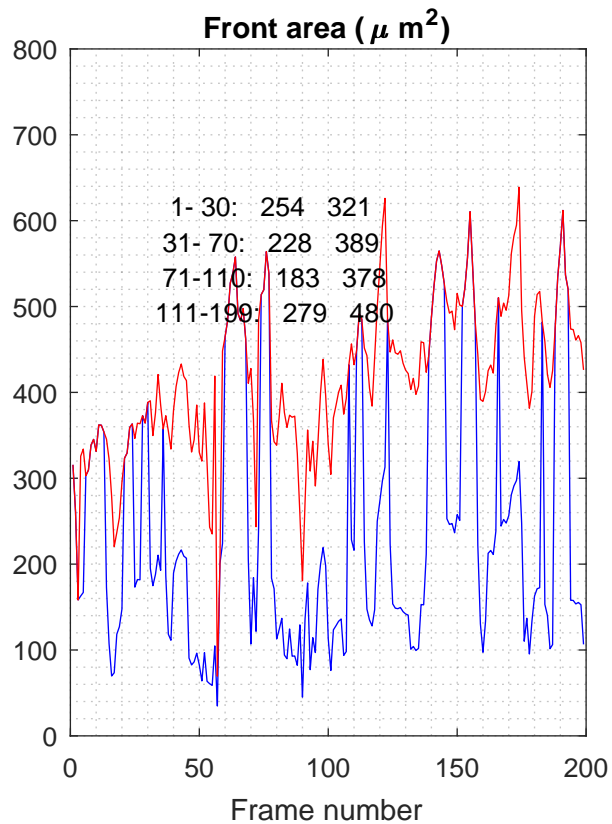

Supplement: Supplementary file 22 — Source Data for Figure 4 [file MSB-15-e8585-s020.zip › Source_data_for_Figure_4/Fig_4I/ChR8-30.pdf]

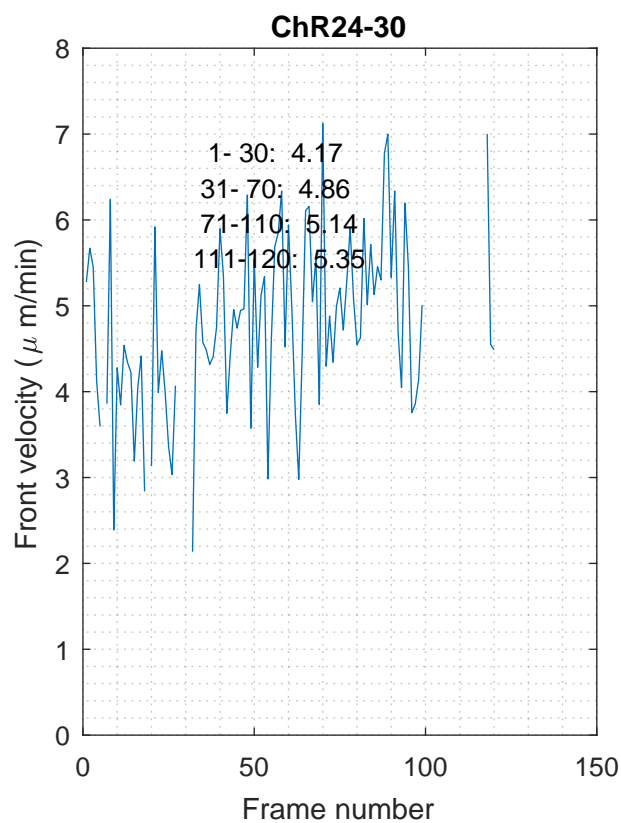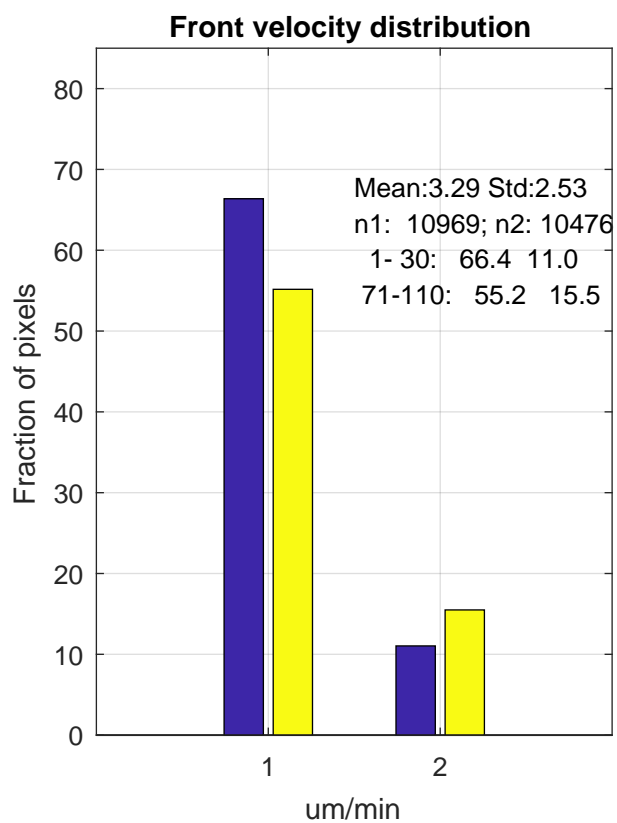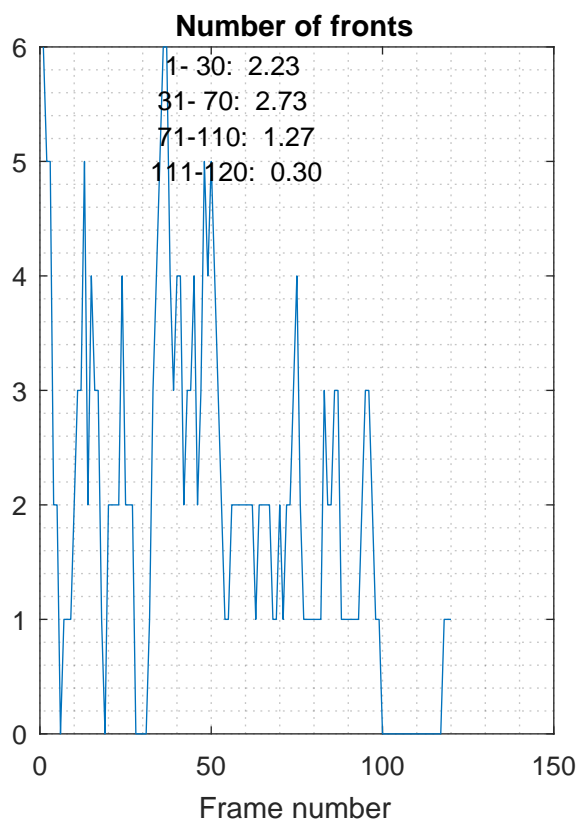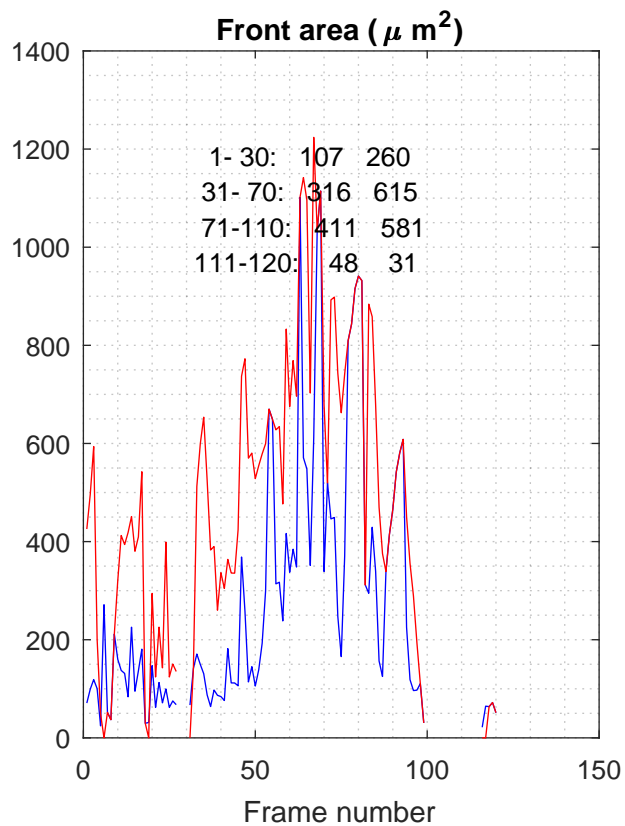

Supplement: Supplementary file 22 — Source Data for Figure 4 [file MSB-15-e8585-s020.zip › Source_data_for_Figure_4/Fig_4I/ChR24-30.pdf]

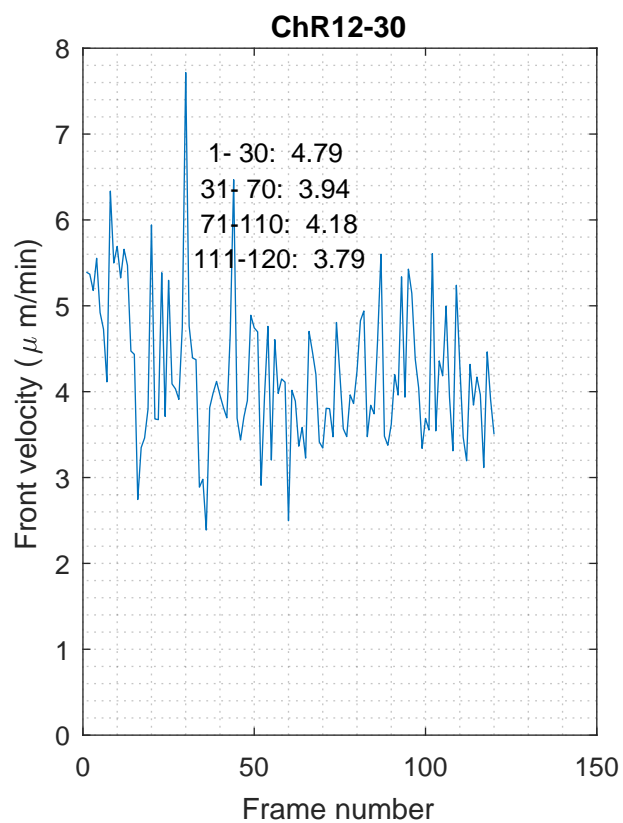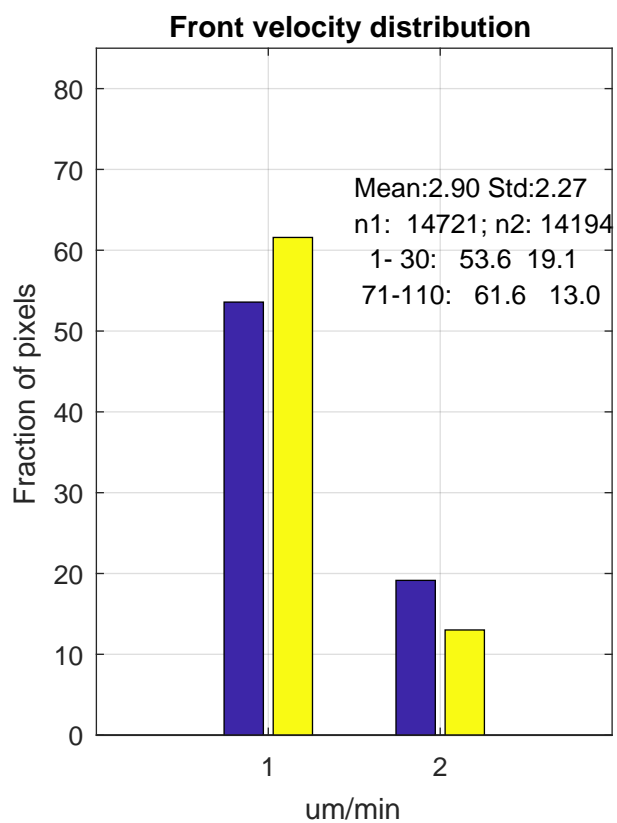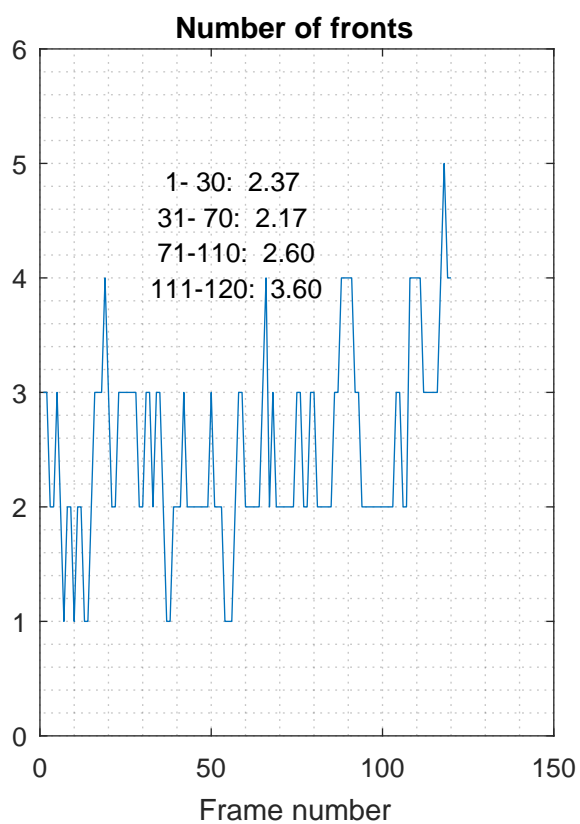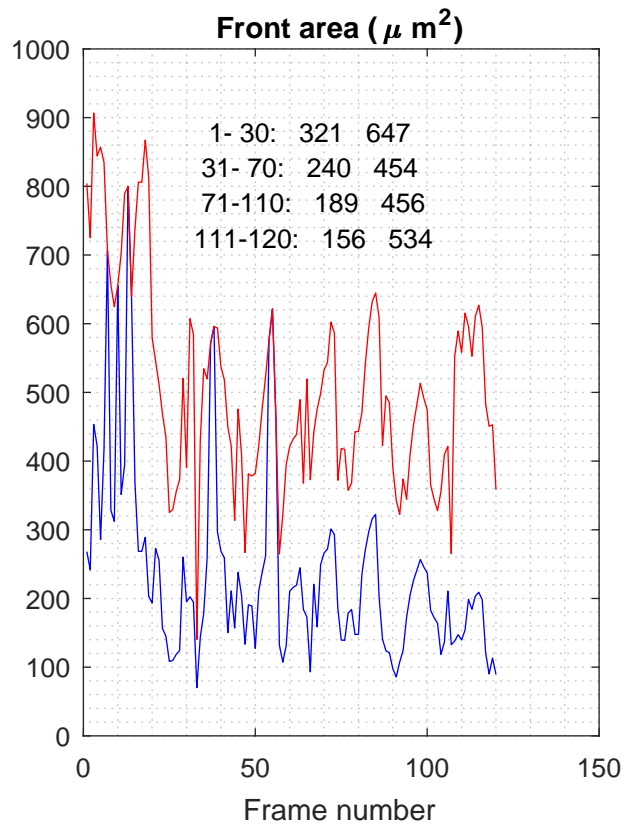

Supplement: Supplementary file 22 — Source Data for Figure 4 [file MSB-15-e8585-s020.zip › Source_data_for_Figure_4/Fig_4I/ChR12-30.pdf]

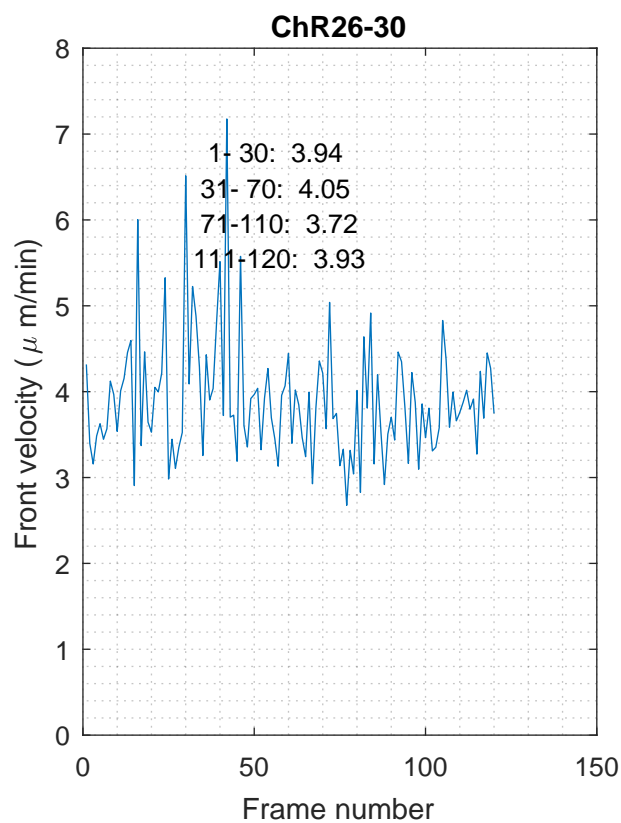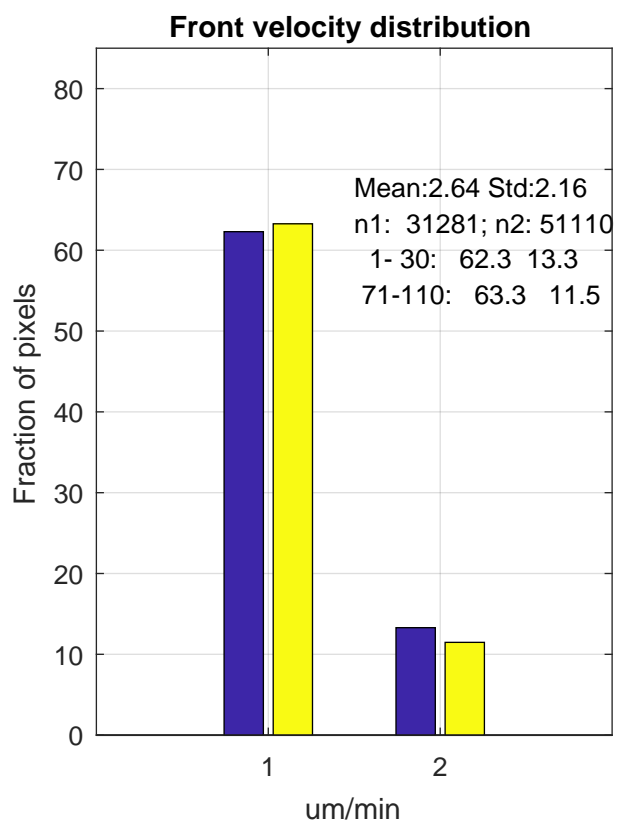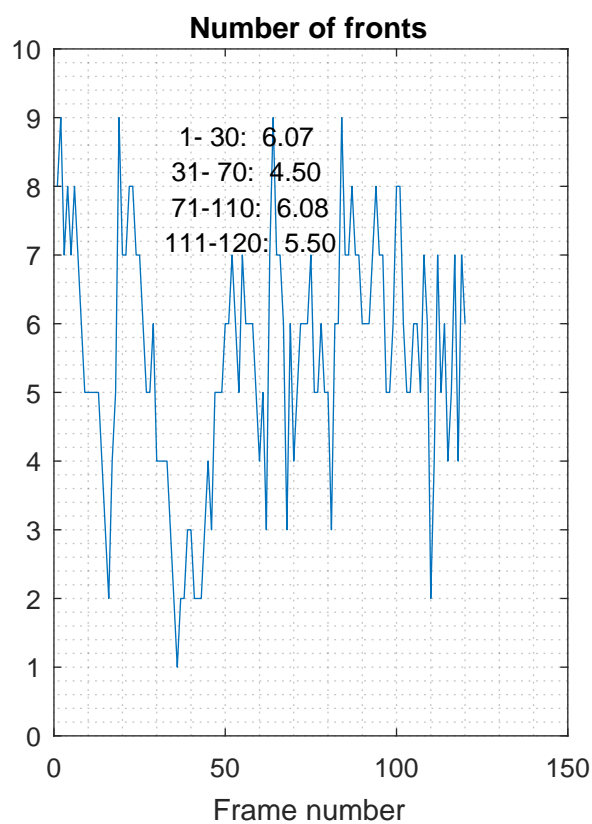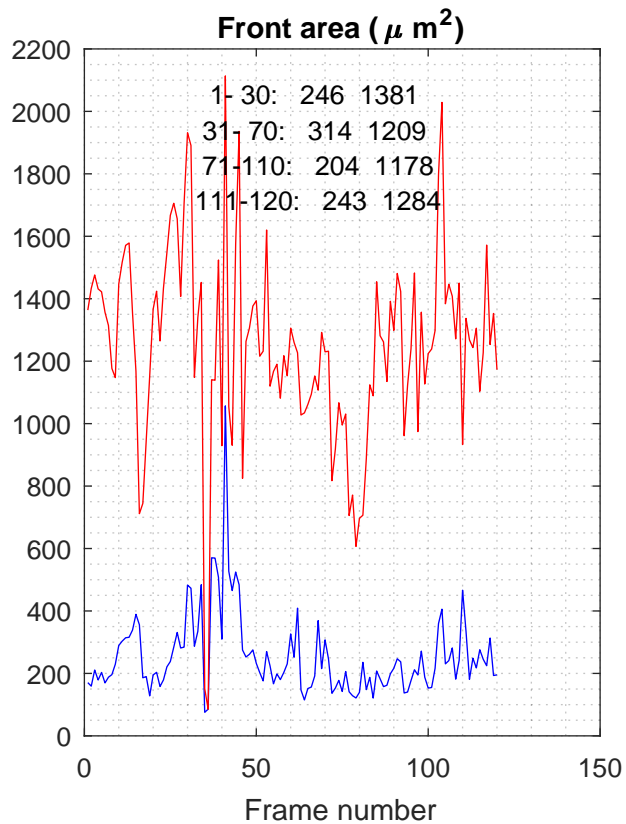

Supplement: Supplementary file 22 — Source Data for Figure 4 [file MSB-15-e8585-s020.zip › Source_data_for_Figure_4/Fig_4I/ChR26-30.pdf]

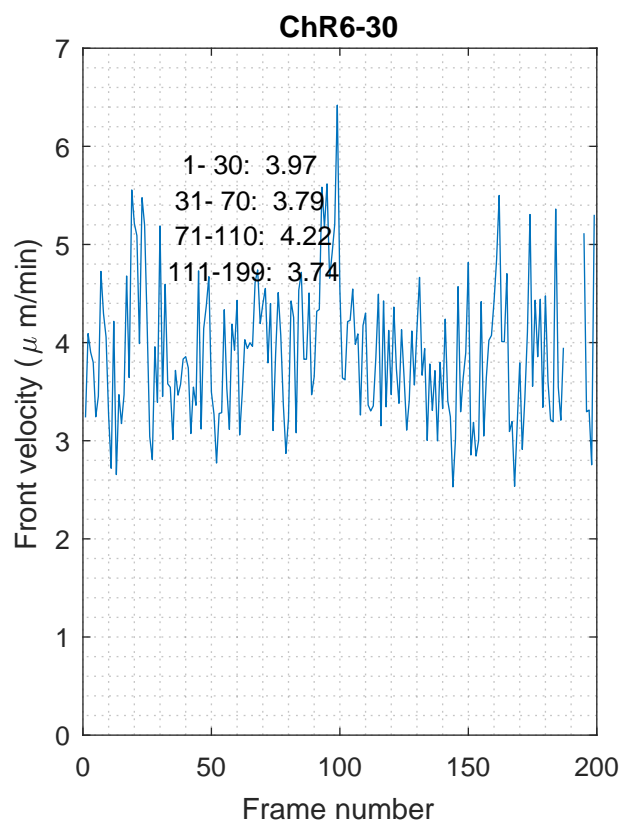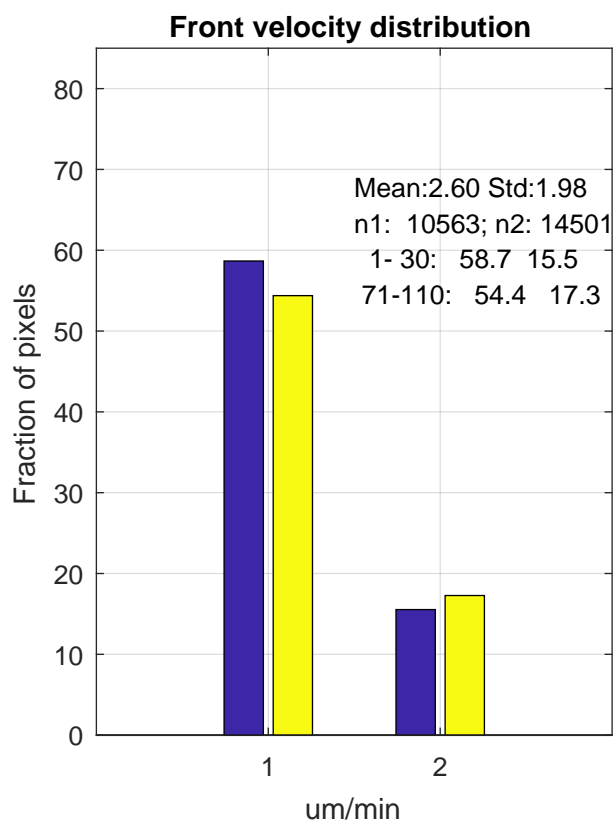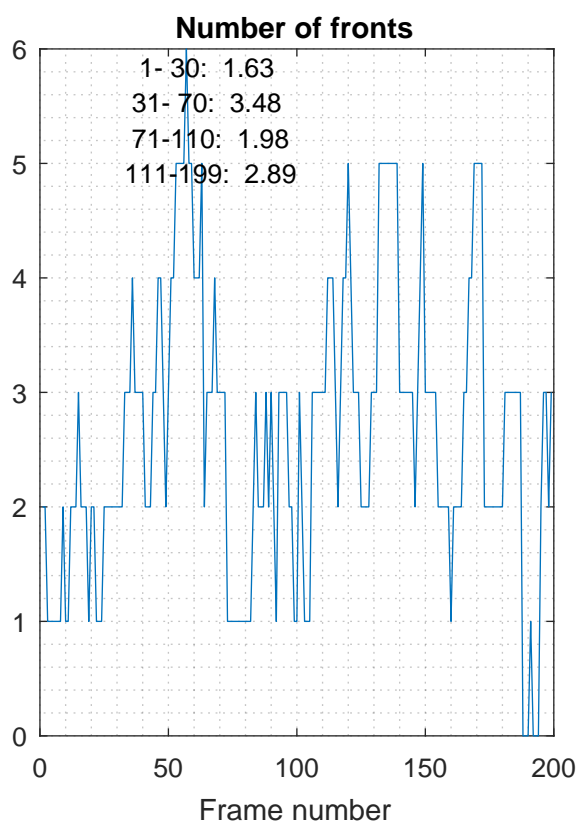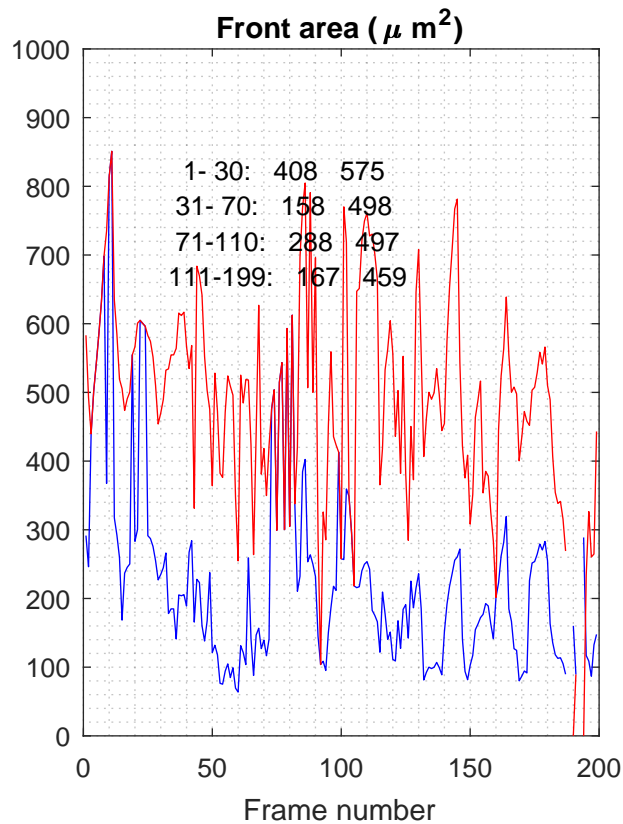

Supplement: Supplementary file 22 — Source Data for Figure 4 [file MSB-15-e8585-s020.zip › Source_data_for_Figure_4/Fig_4I/ChR6-30.pdf]

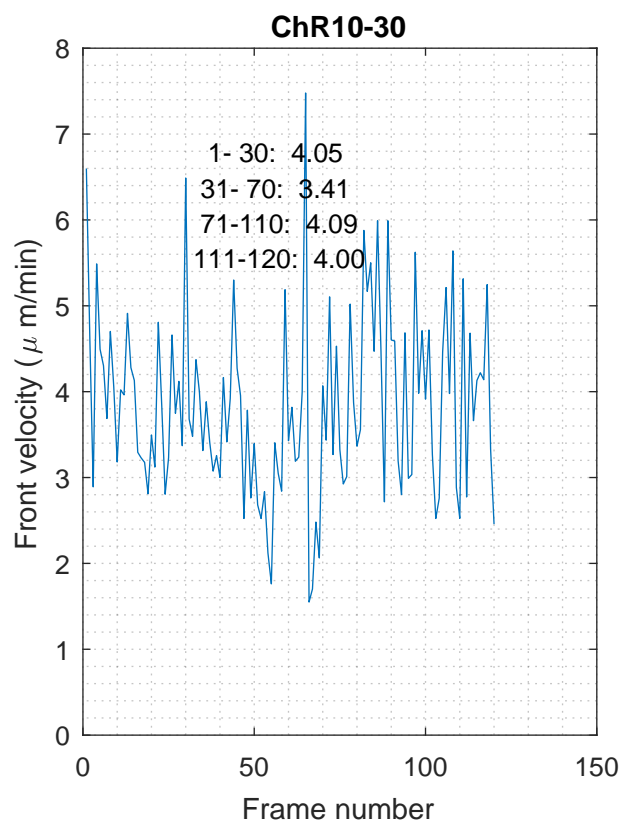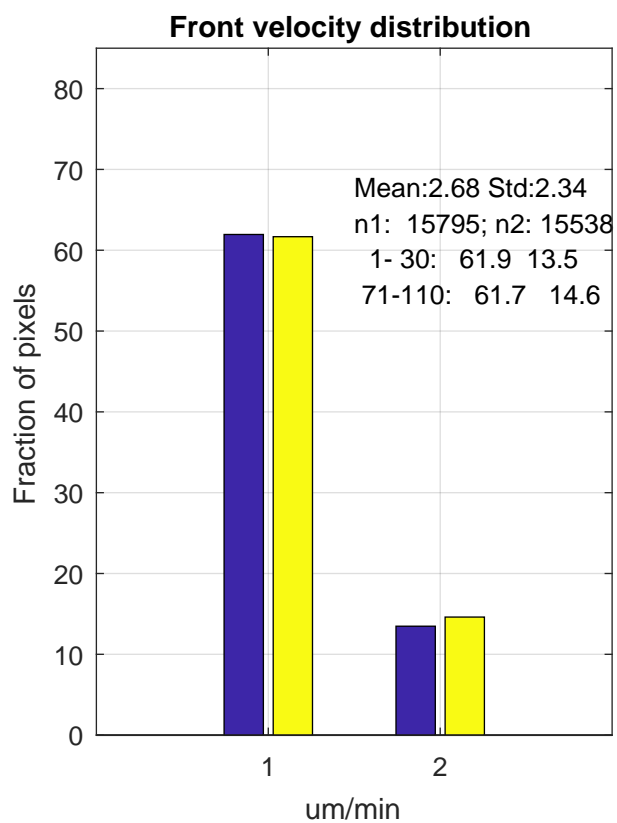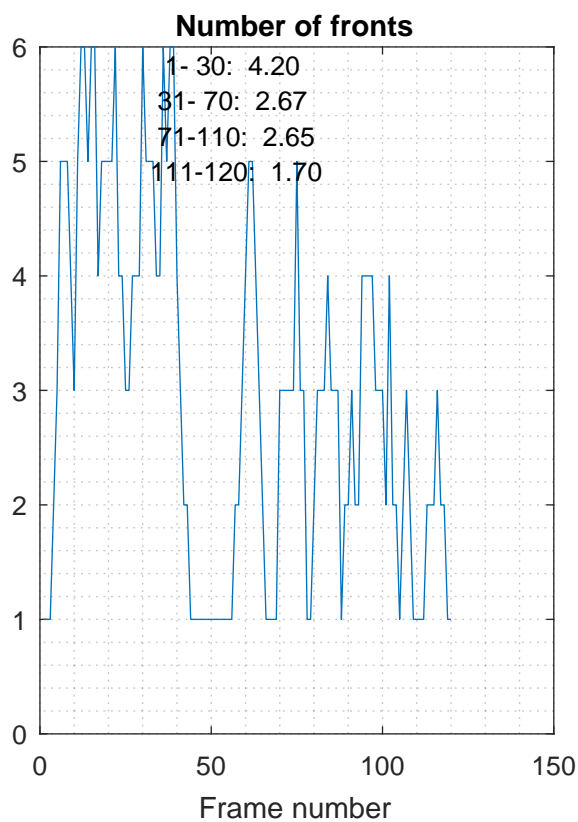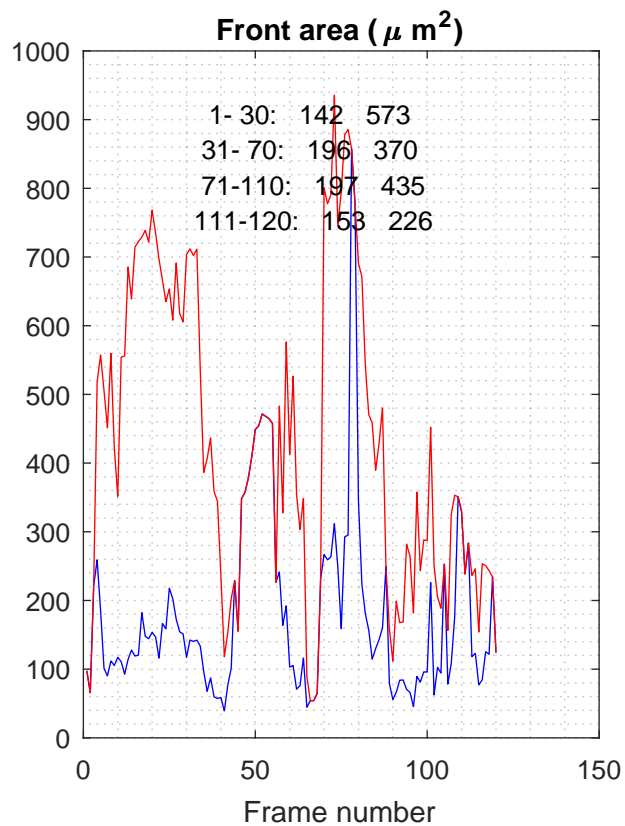

Supplement: Supplementary file 22 — Source Data for Figure 4 [file MSB-15-e8585-s020.zip › Source_data_for_Figure_4/Fig_4I/ChR10-30.pdf]

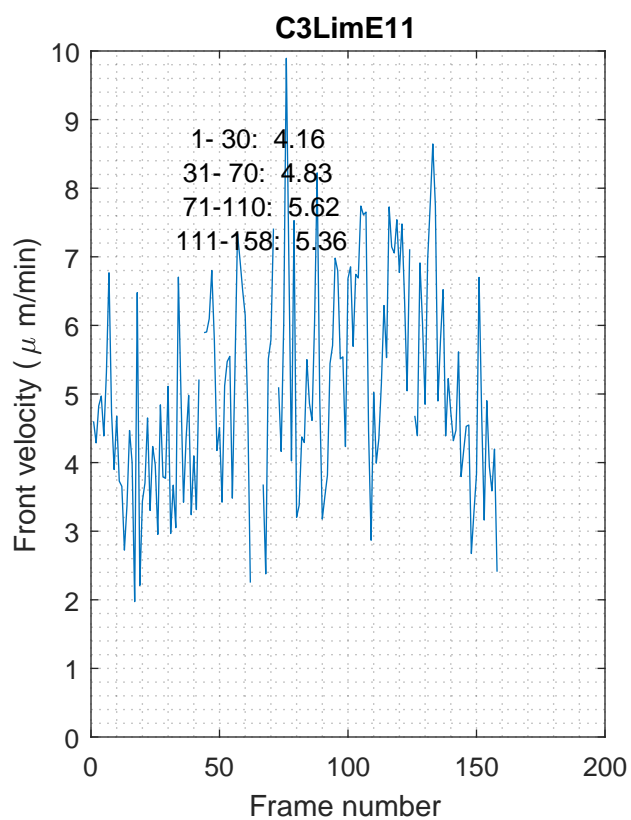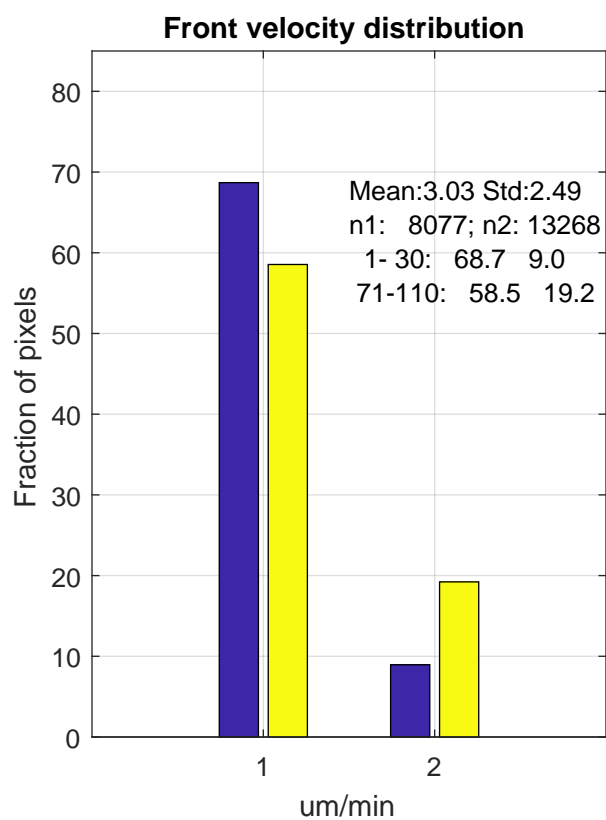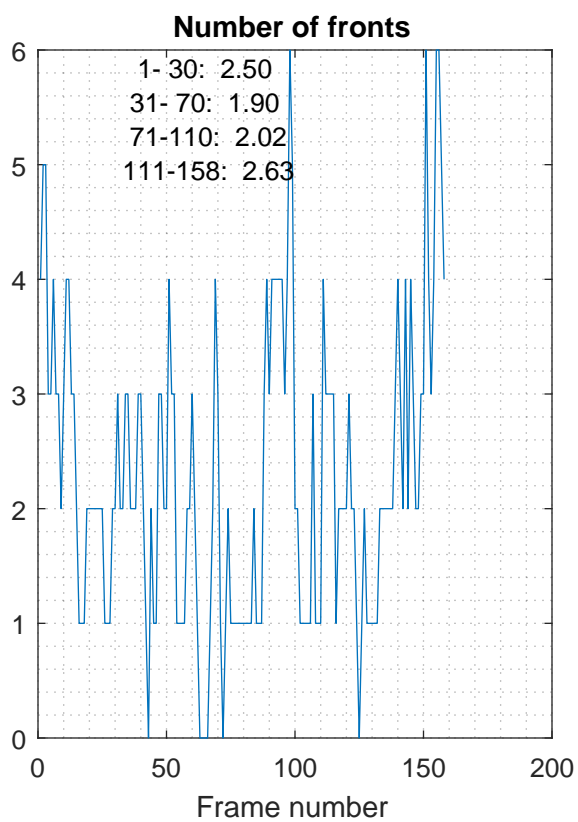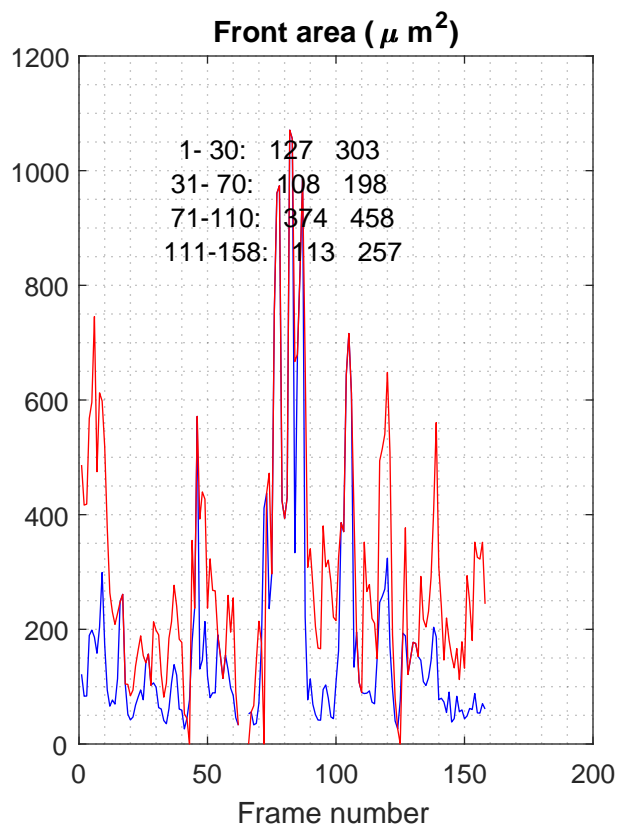

Supplement: Supplementary file 22 — Source Data for Figure 4 [file MSB-15-e8585-s020.zip › Source_data_for_Figure_4/Fig_4H/9.pdf]
