# Supplementary figures and images for: Wave patterns organize cellular protrusions and control cortical dynamics (part 2 of 2)
Source: Mol Syst Biol. 2019 Mar 12;15(3):e8585. doi: 10.15252/msb.20188585 (PMC6413885; doi:10.15252/msb.20188585)

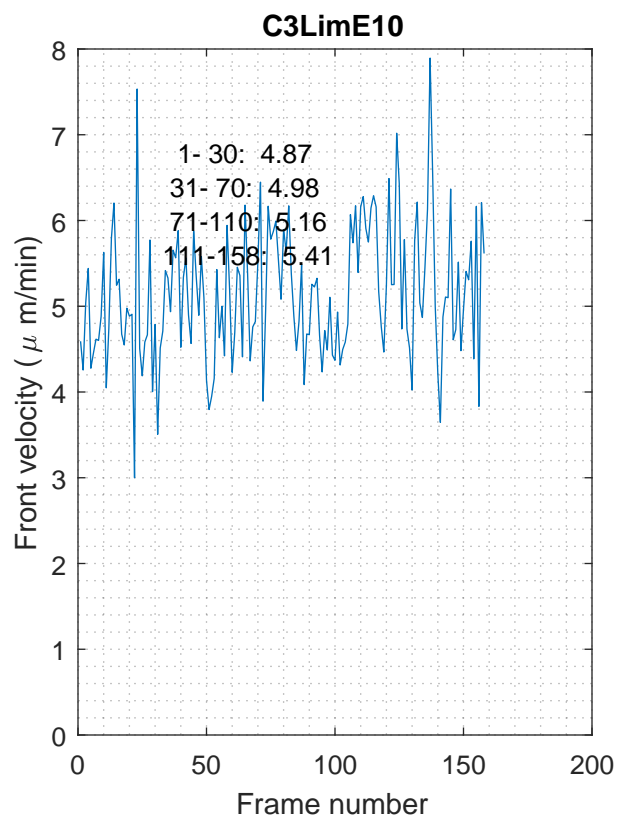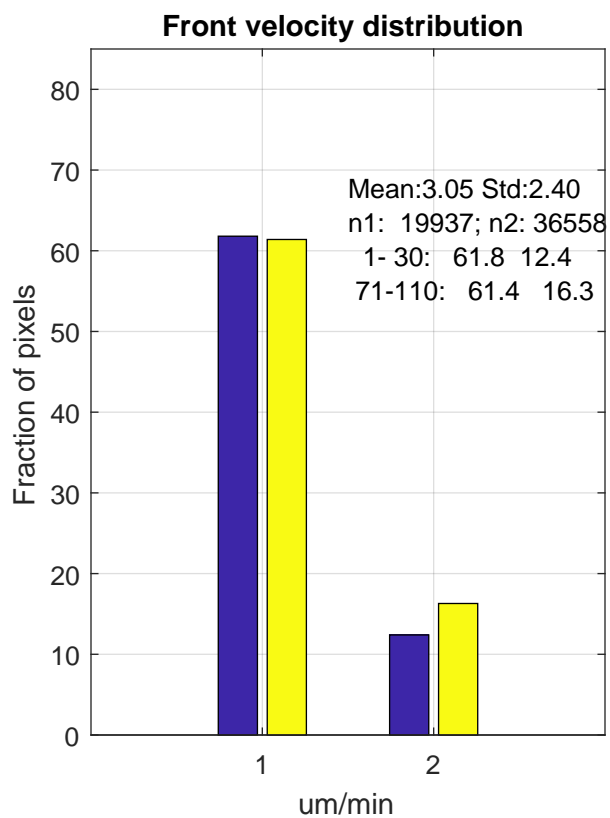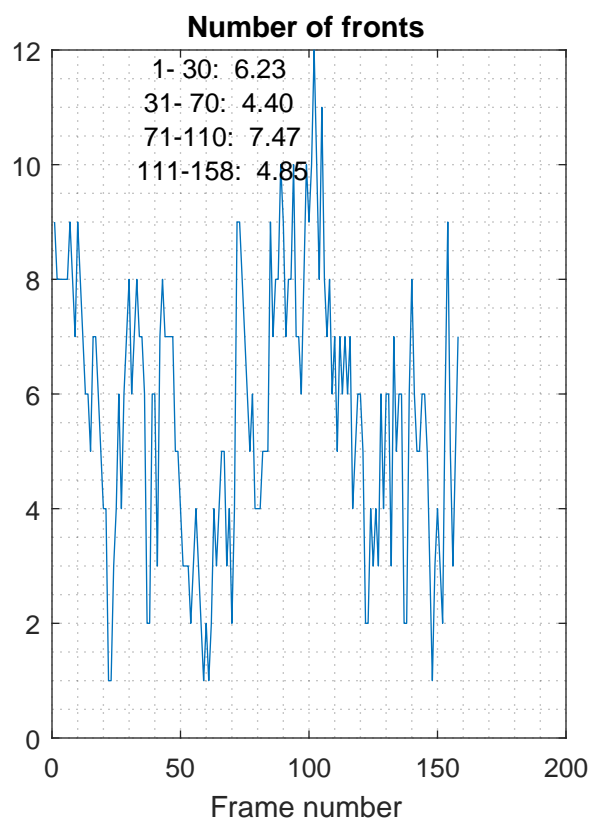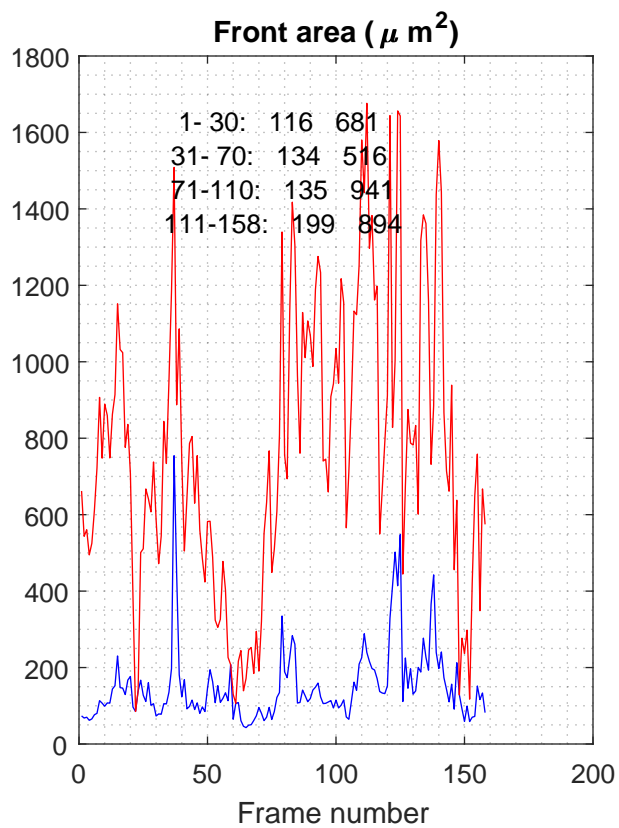

Supplement: Supplementary file 22 — Source Data for Figure 4 [file MSB-15-e8585-s020.zip › Source_data_for_Figure_4/Fig_4H/8.pdf]

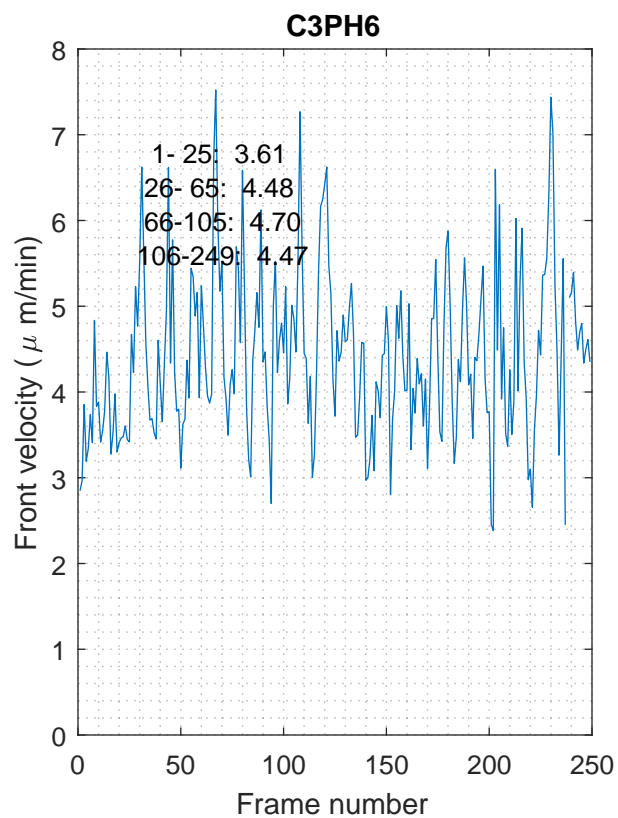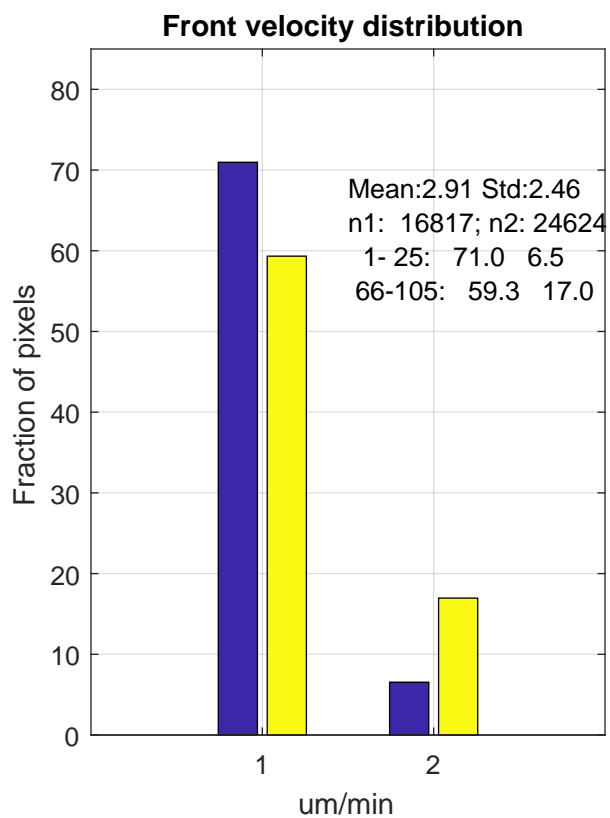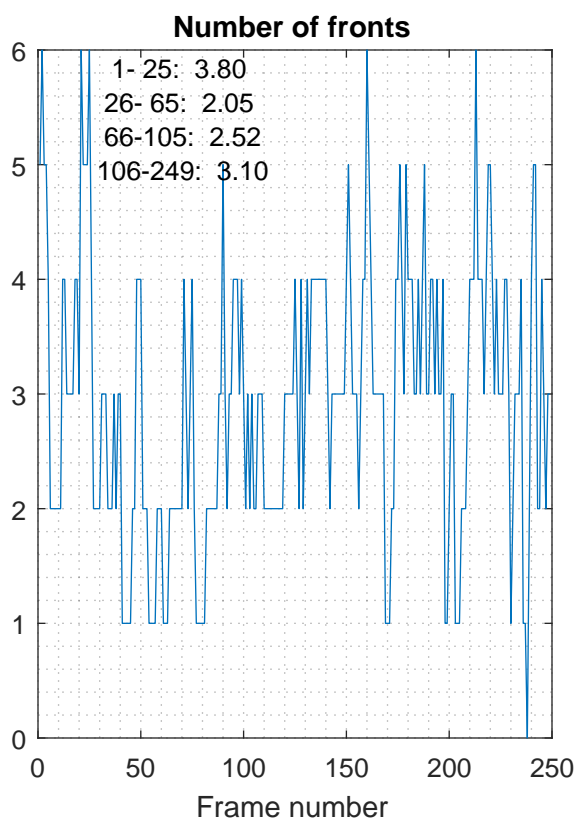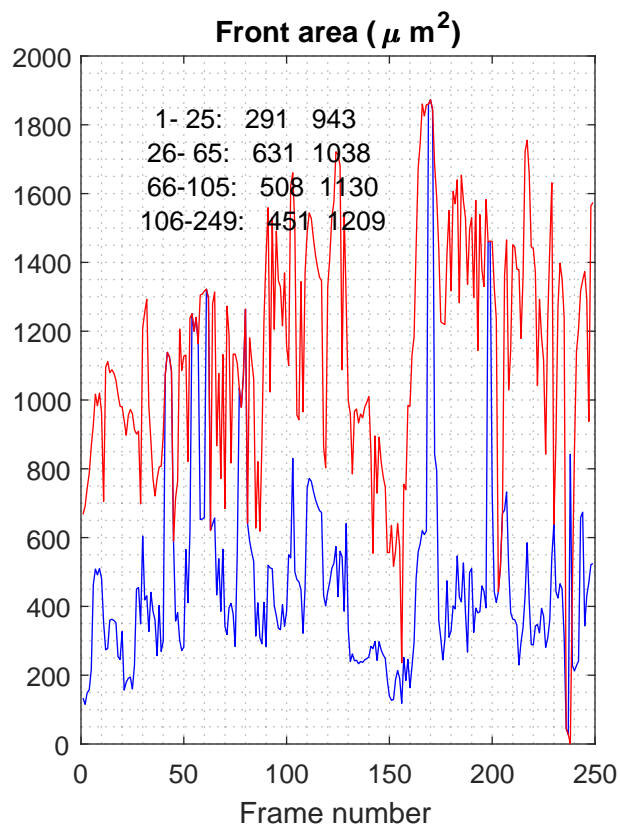

Supplement: Supplementary file 22 — Source Data for Figure 4 [file MSB-15-e8585-s020.zip › Source_data_for_Figure_4/Fig_4H/16.pdf]

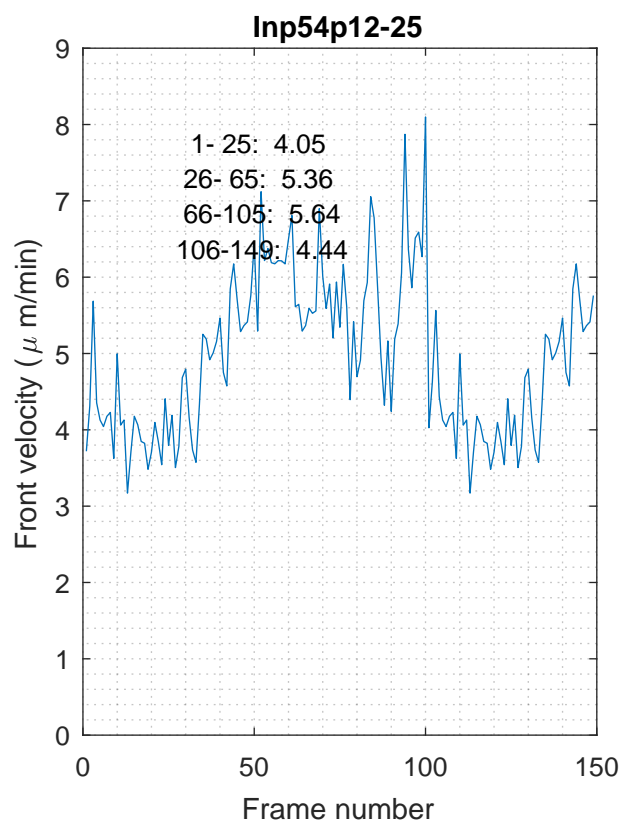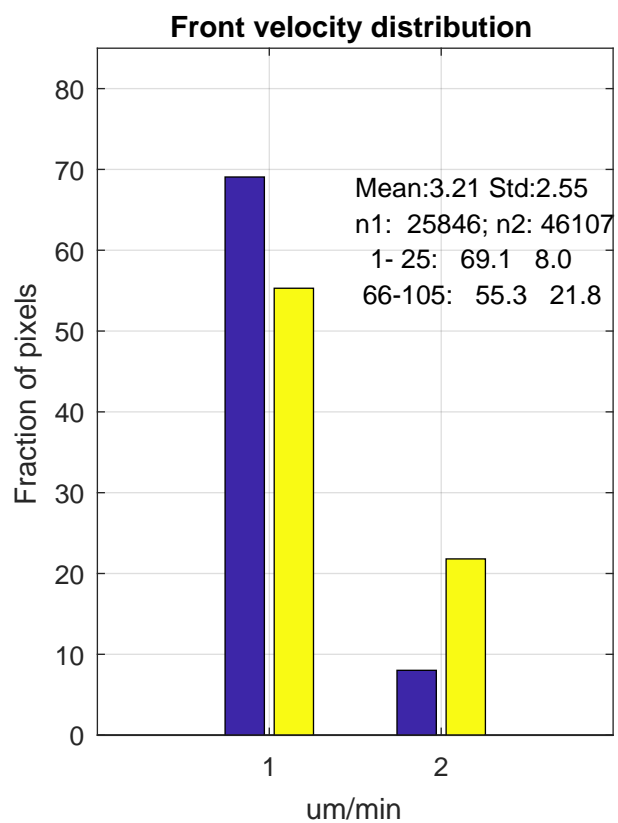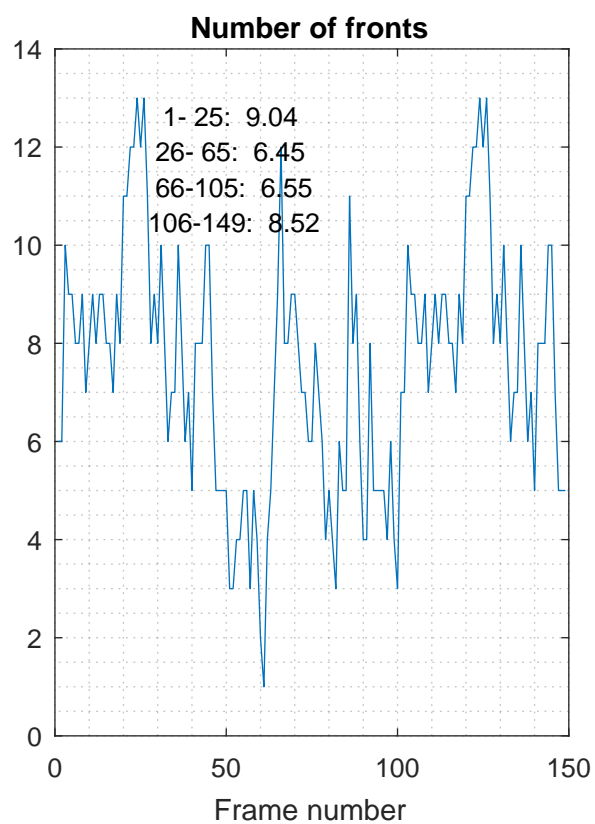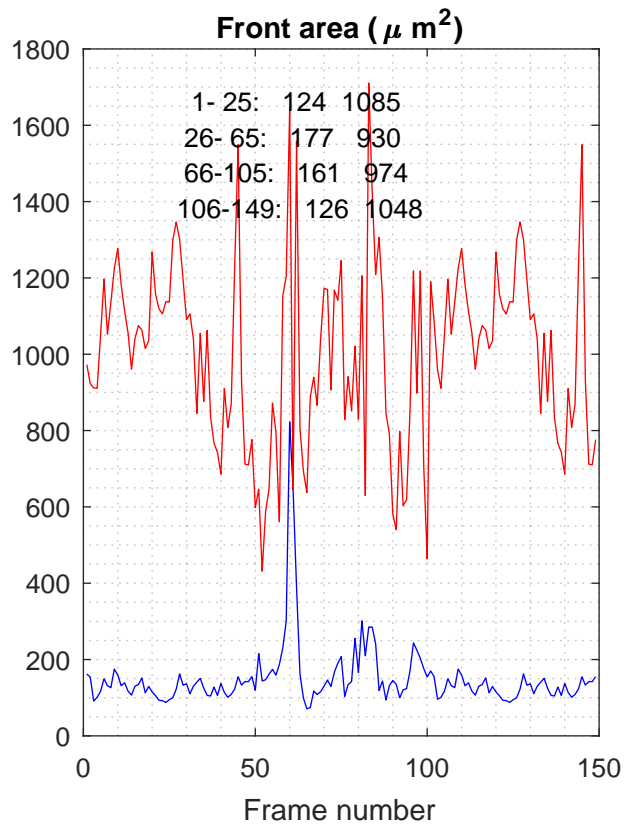

Supplement: Supplementary file 22 — Source Data for Figure 4 [file MSB-15-e8585-s020.zip › Source_data_for_Figure_4/Fig_4H/17.pdf]

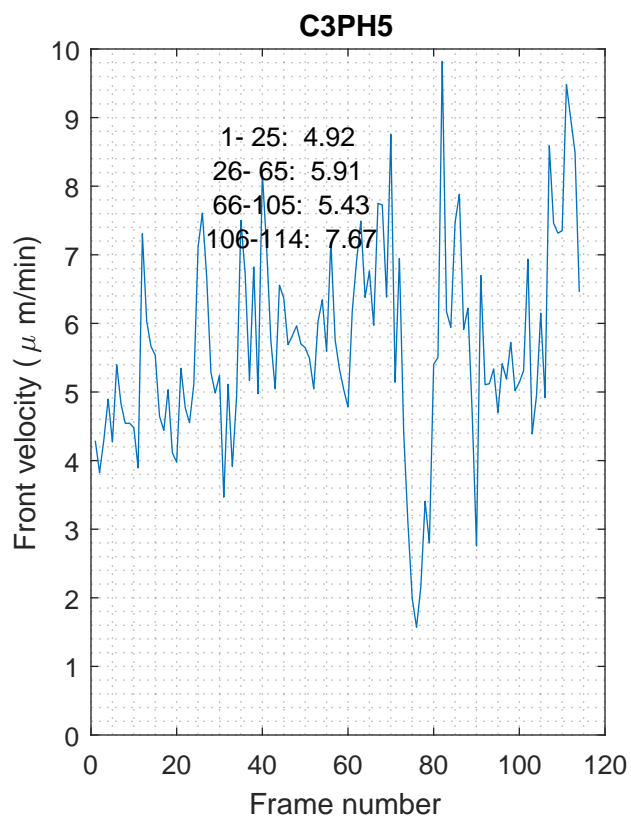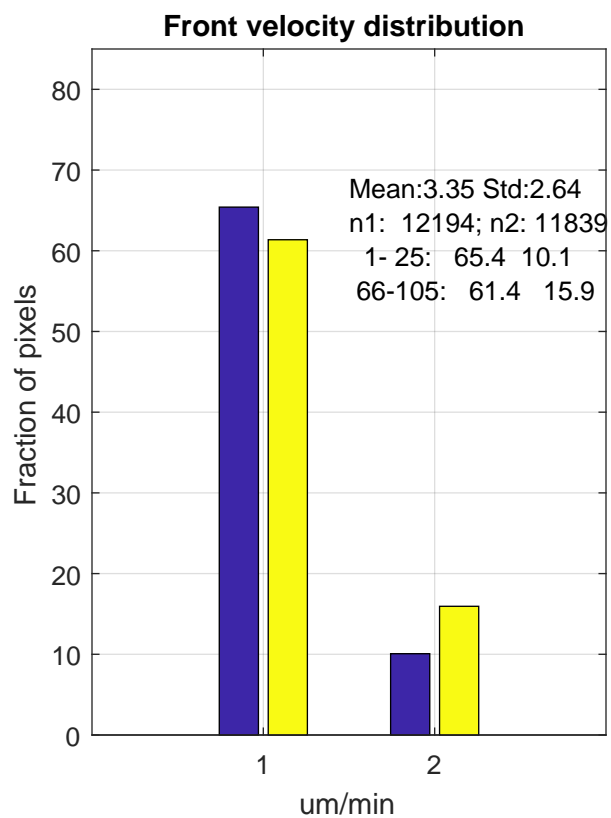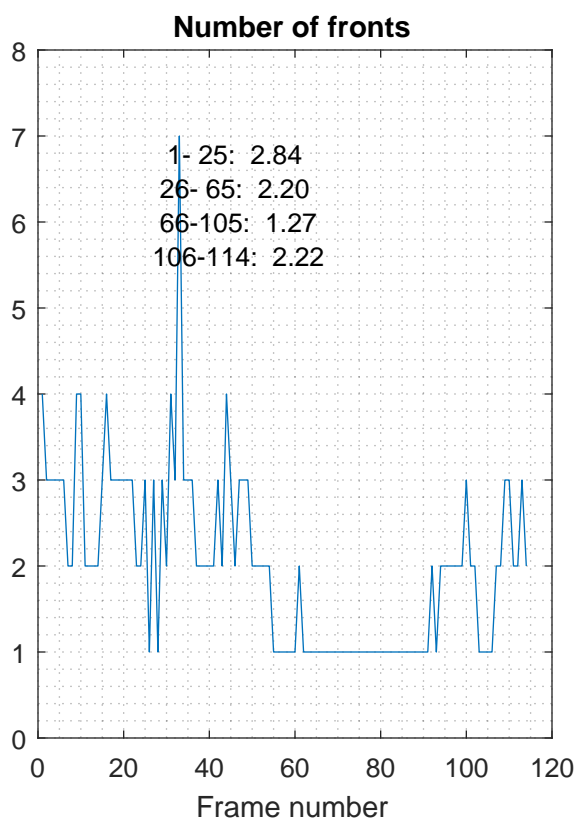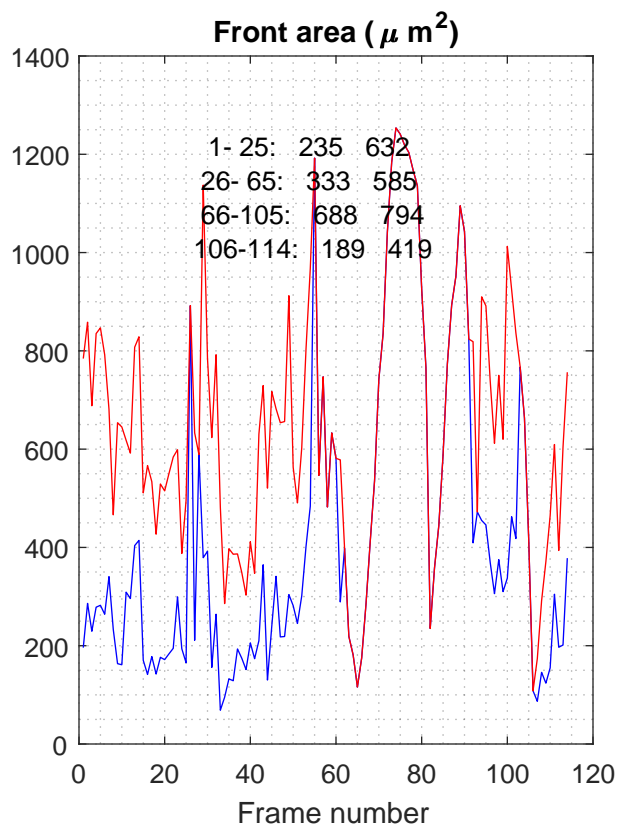

Supplement: Supplementary file 22 — Source Data for Figure 4 [file MSB-15-e8585-s020.zip › Source_data_for_Figure_4/Fig_4H/15.pdf]

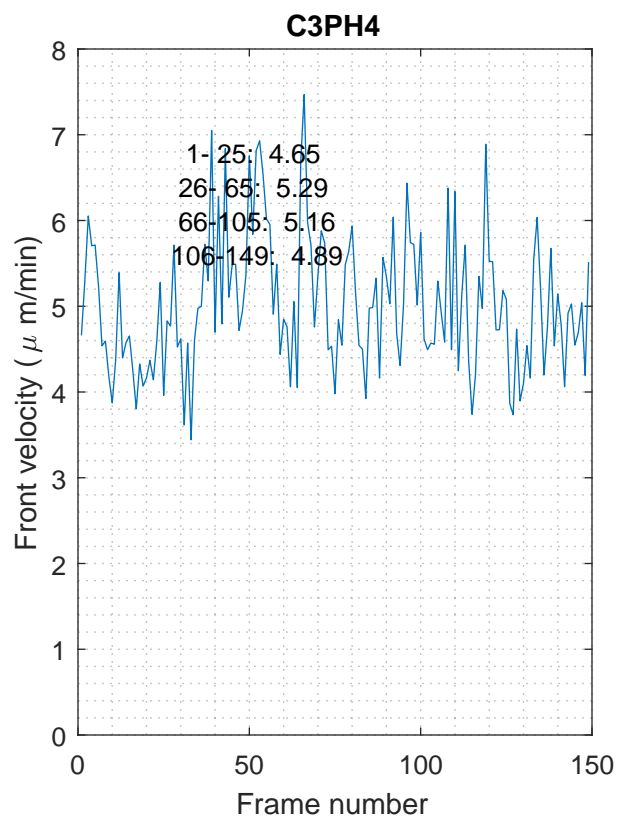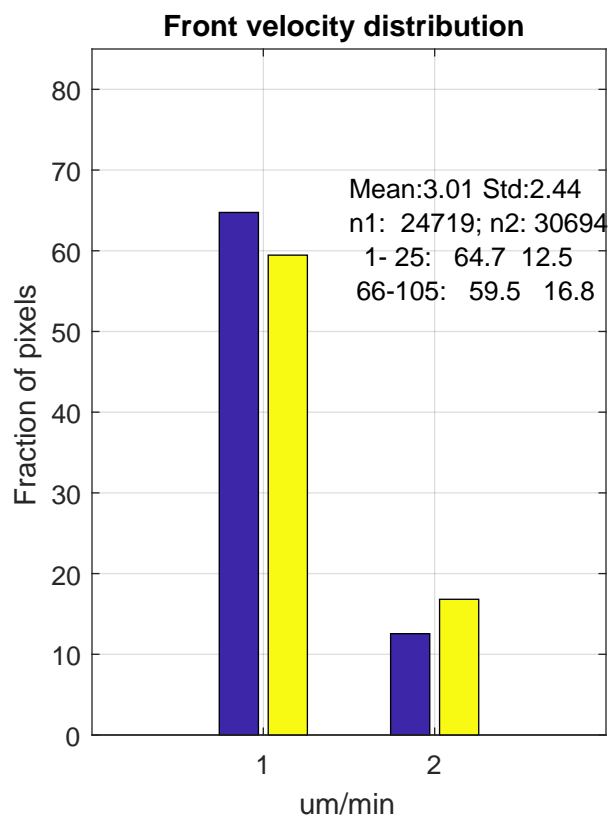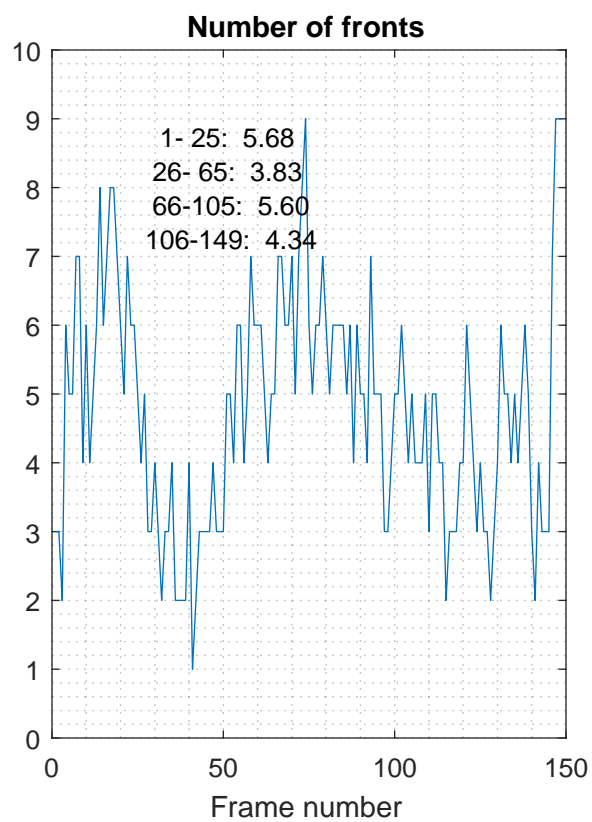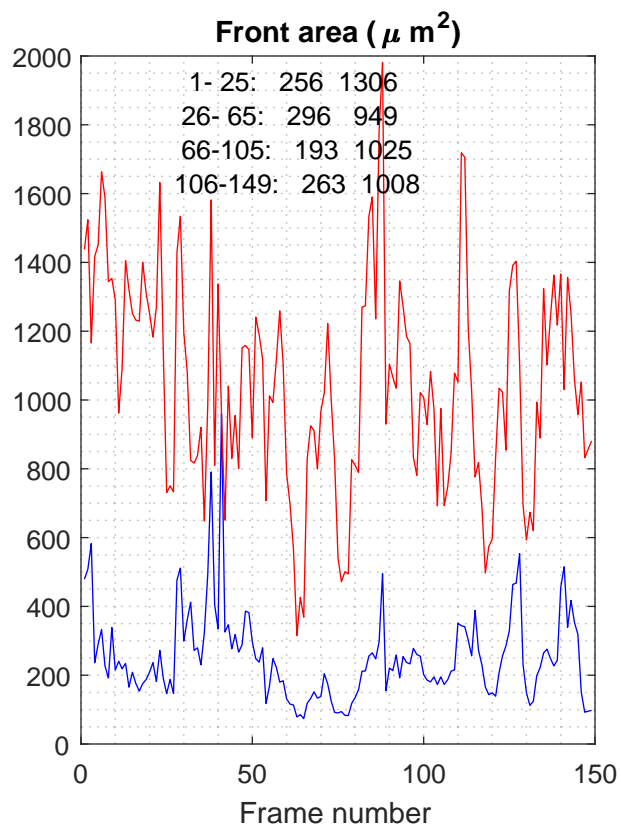

Supplement: Supplementary file 22 — Source Data for Figure 4 [file MSB-15-e8585-s020.zip › Source_data_for_Figure_4/Fig_4H/14.pdf]

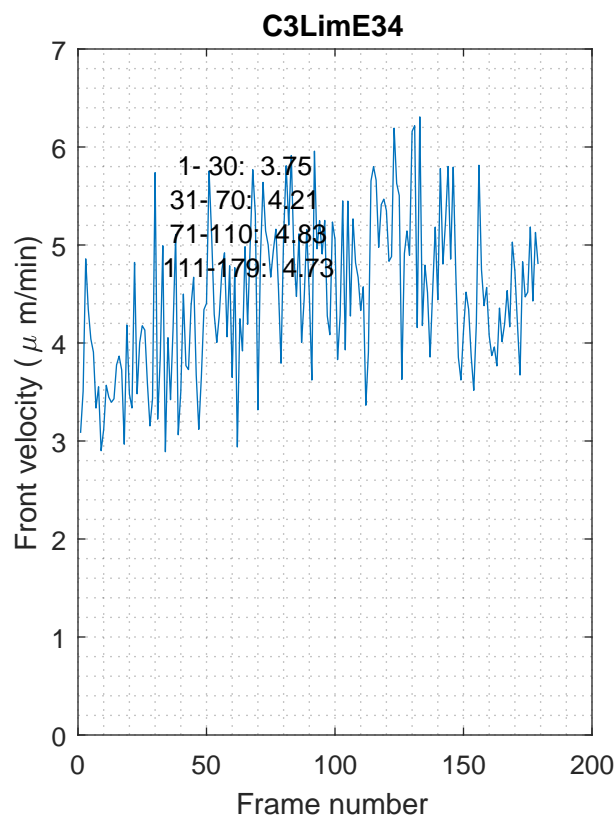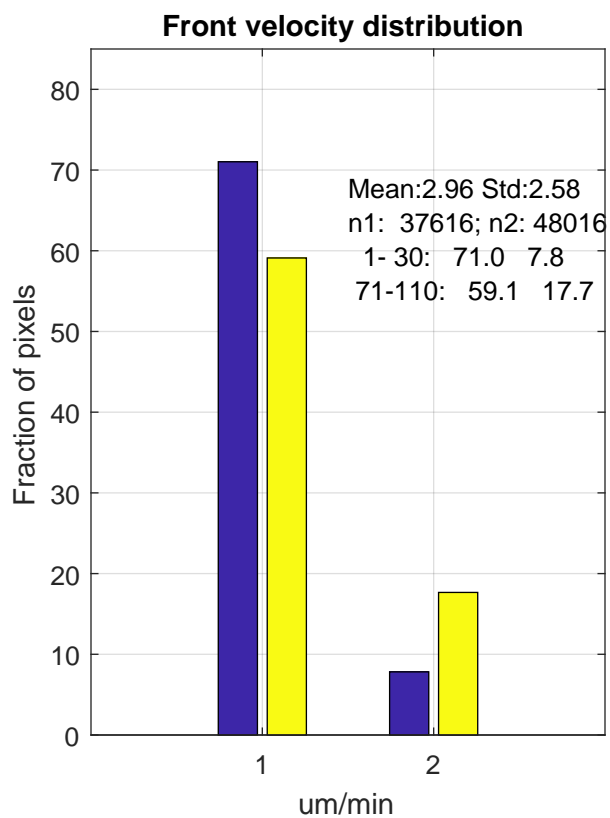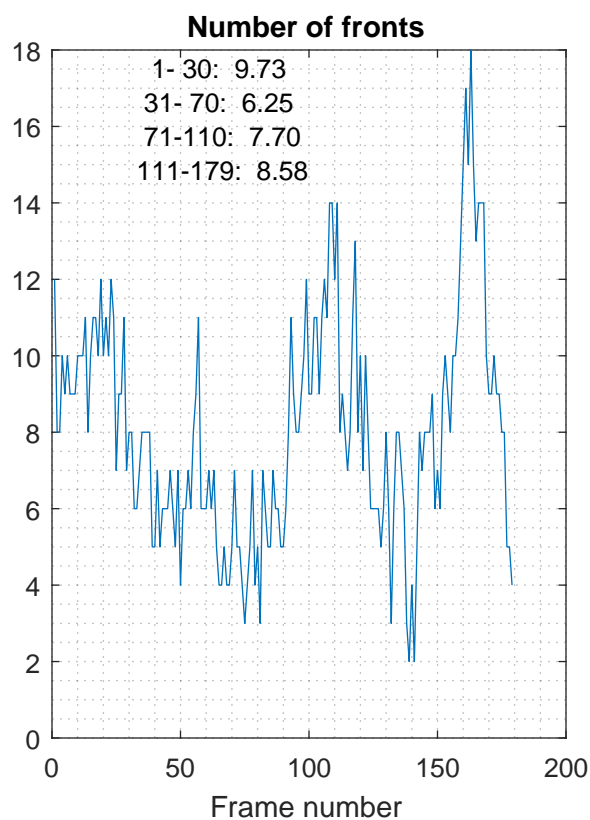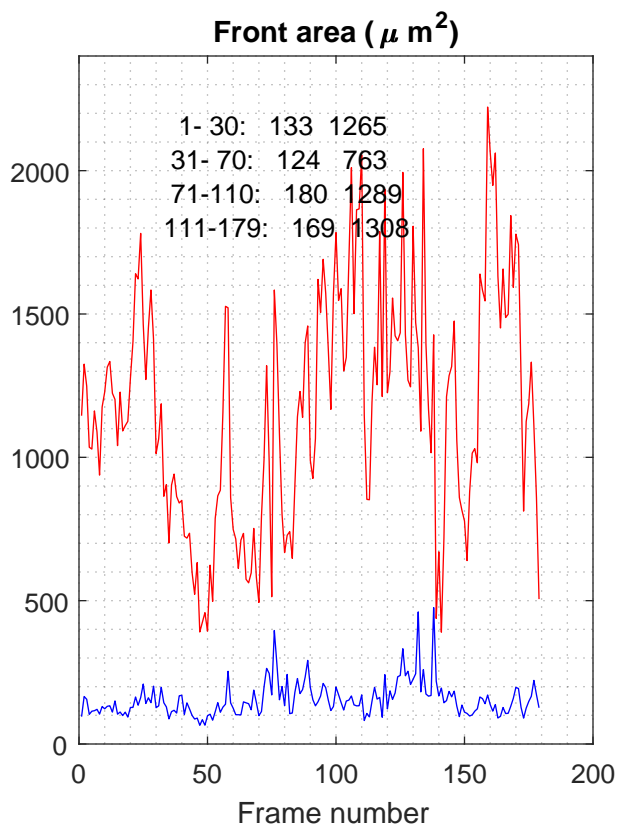

Supplement: Supplementary file 22 — Source Data for Figure 4 [file MSB-15-e8585-s020.zip › Source_data_for_Figure_4/Fig_4H/10.pdf]

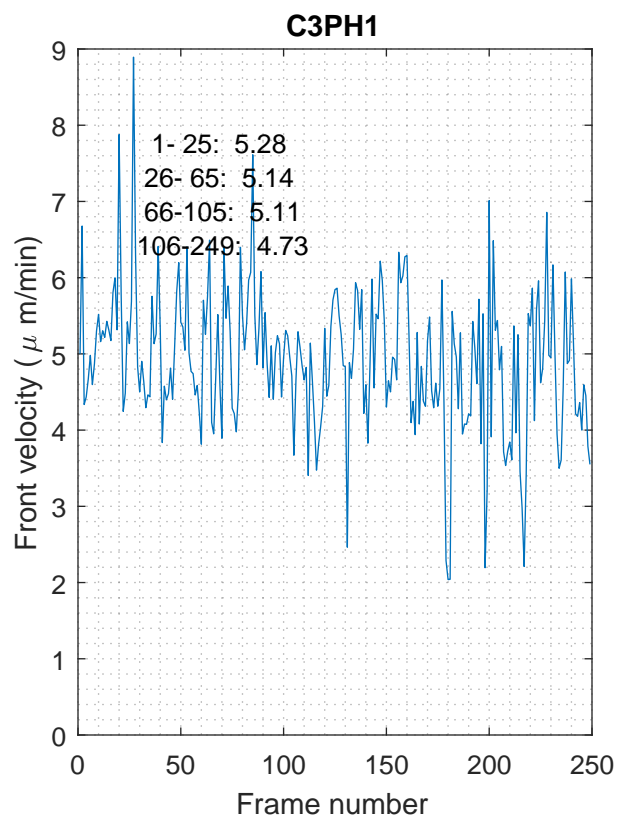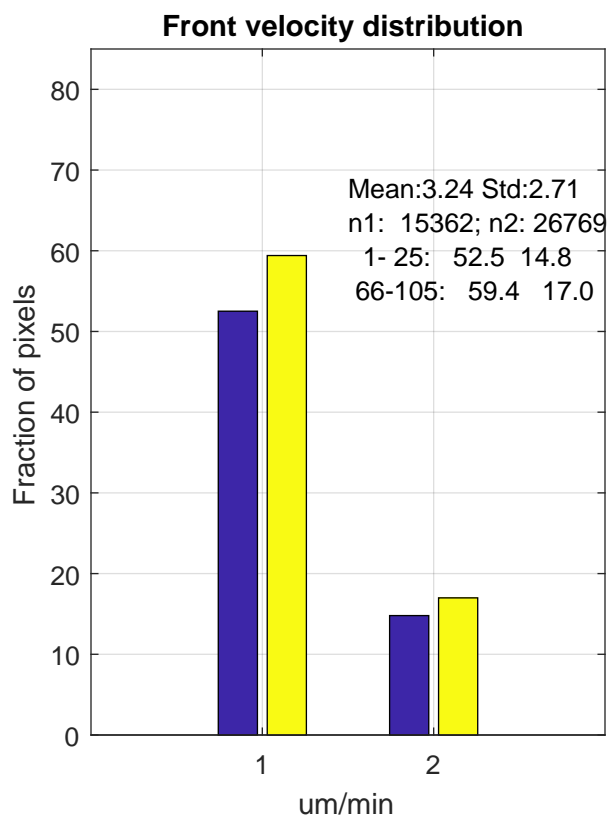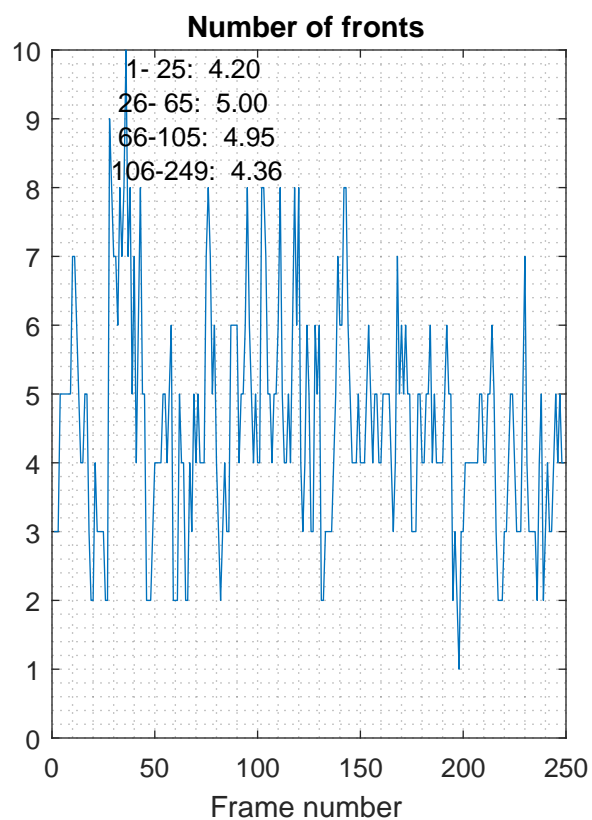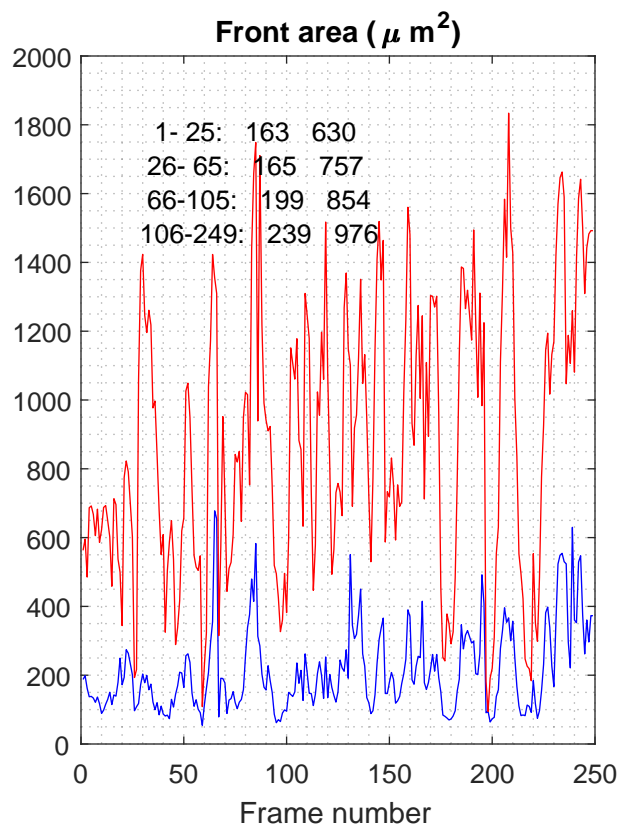

Supplement: Supplementary file 22 — Source Data for Figure 4 [file MSB-15-e8585-s020.zip › Source_data_for_Figure_4/Fig_4H/11.pdf]

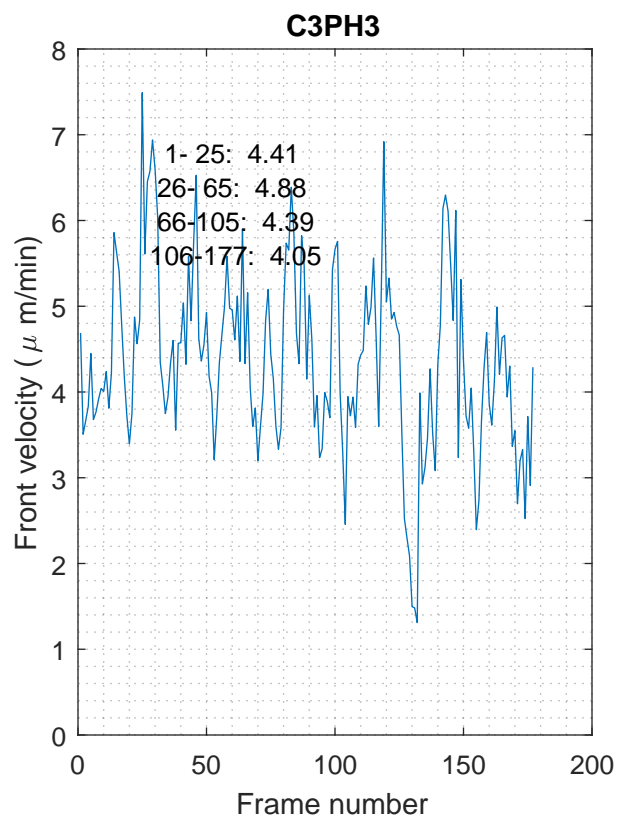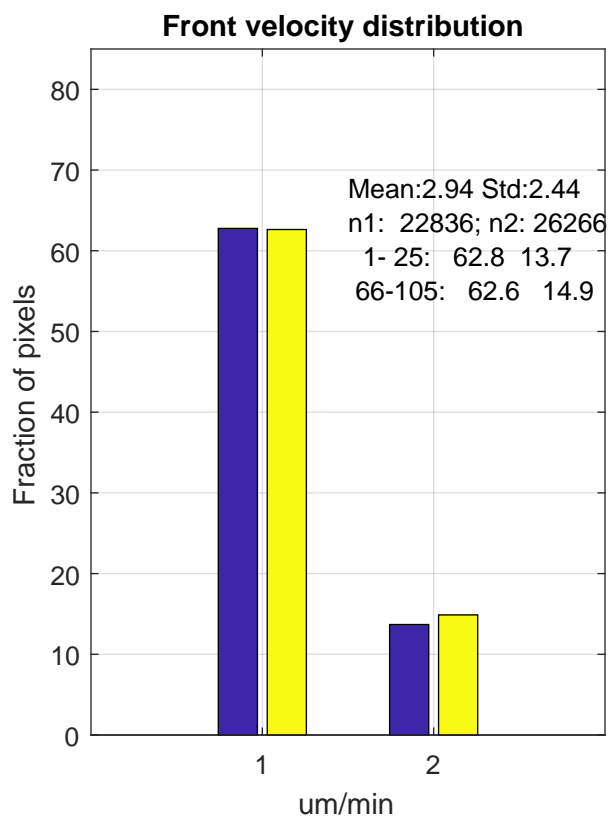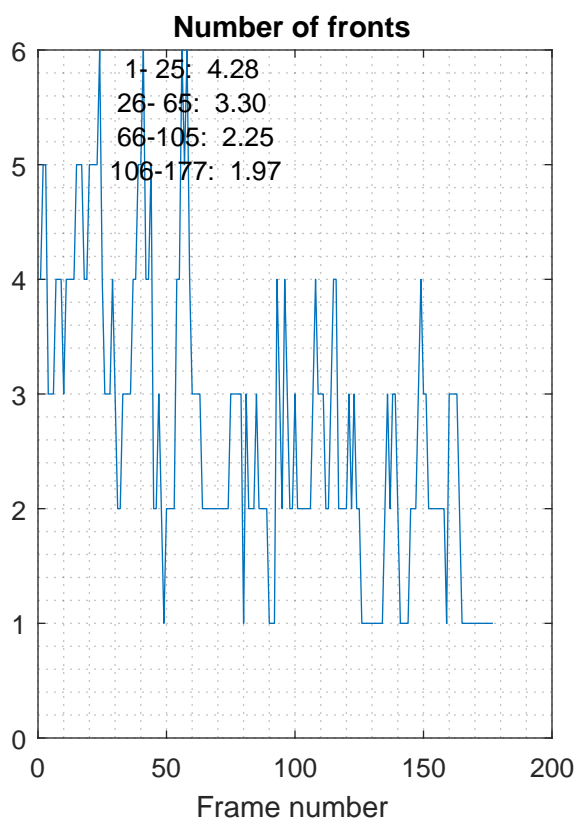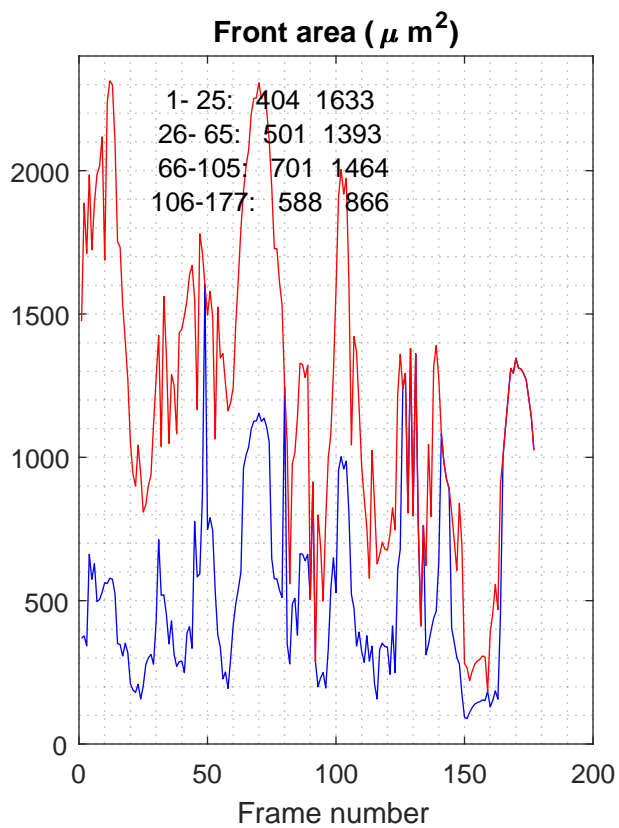

Supplement: Supplementary file 22 — Source Data for Figure 4 [file MSB-15-e8585-s020.zip › Source_data_for_Figure_4/Fig_4H/13.pdf]

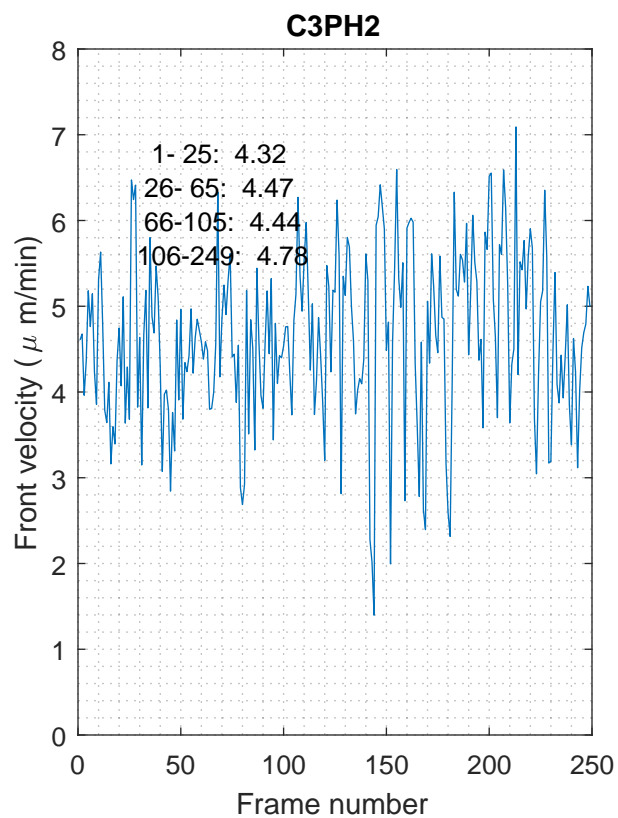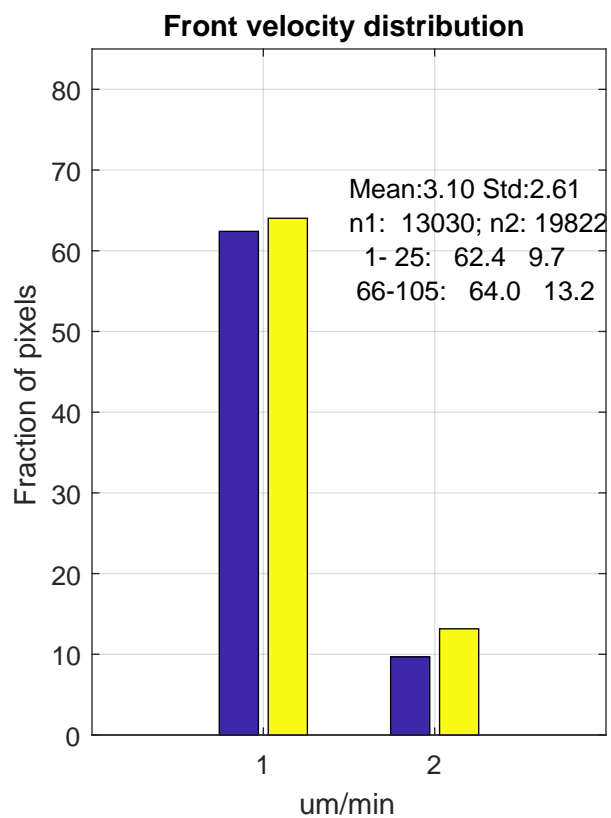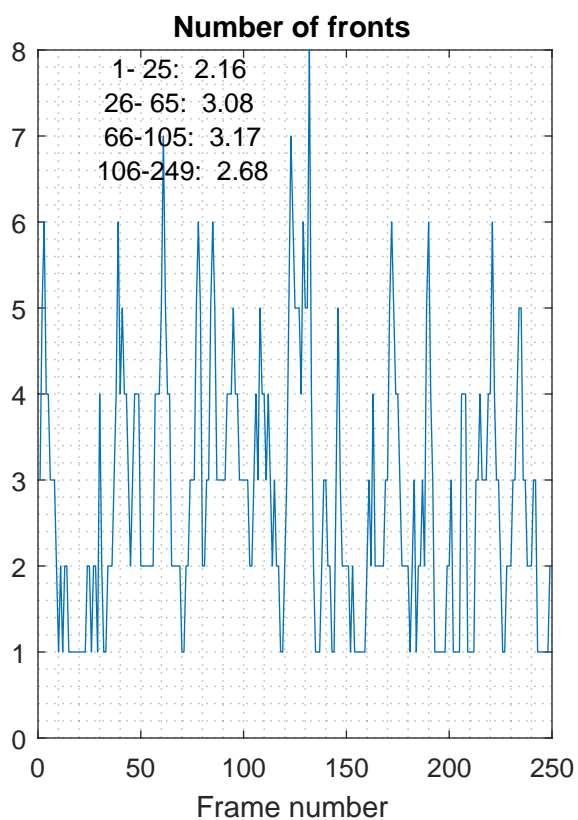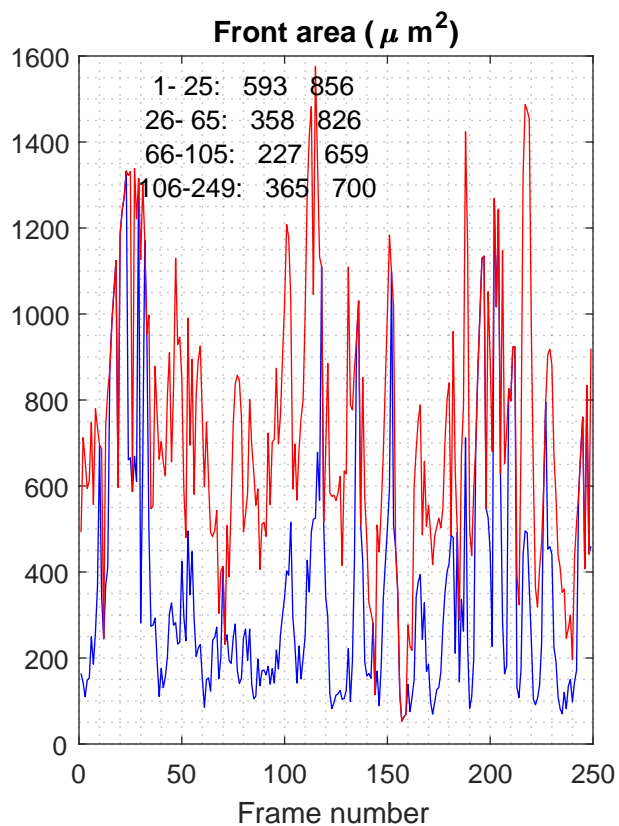

Supplement: Supplementary file 22 — Source Data for Figure 4 [file MSB-15-e8585-s020.zip › Source_data_for_Figure_4/Fig_4H/12.pdf]

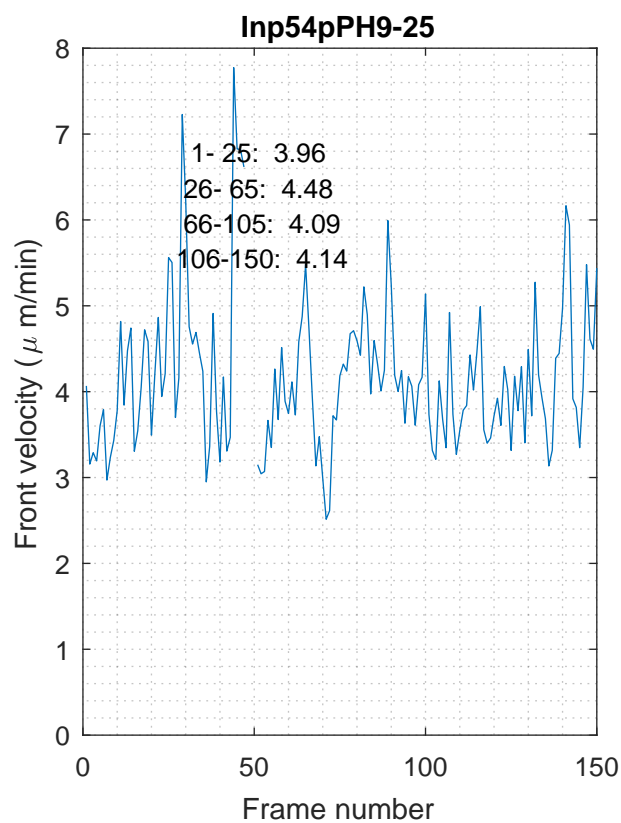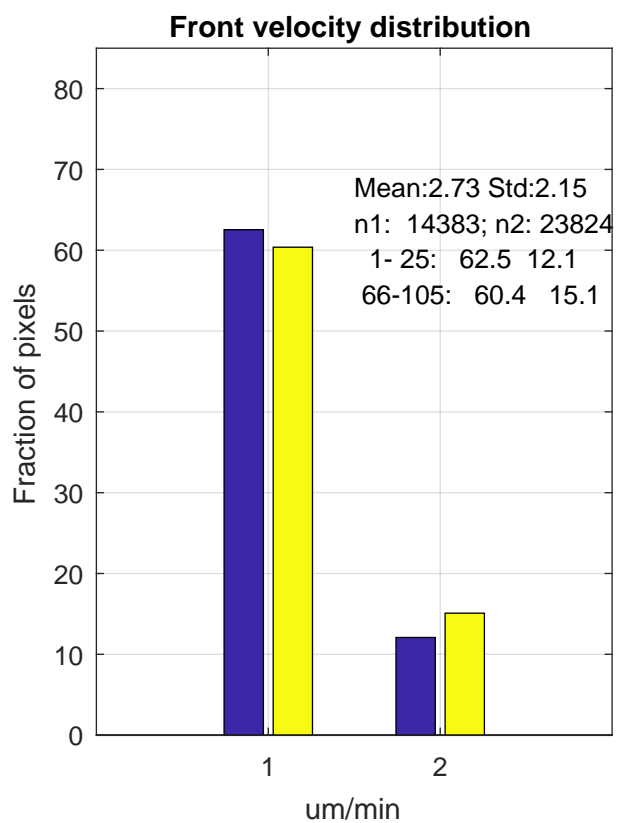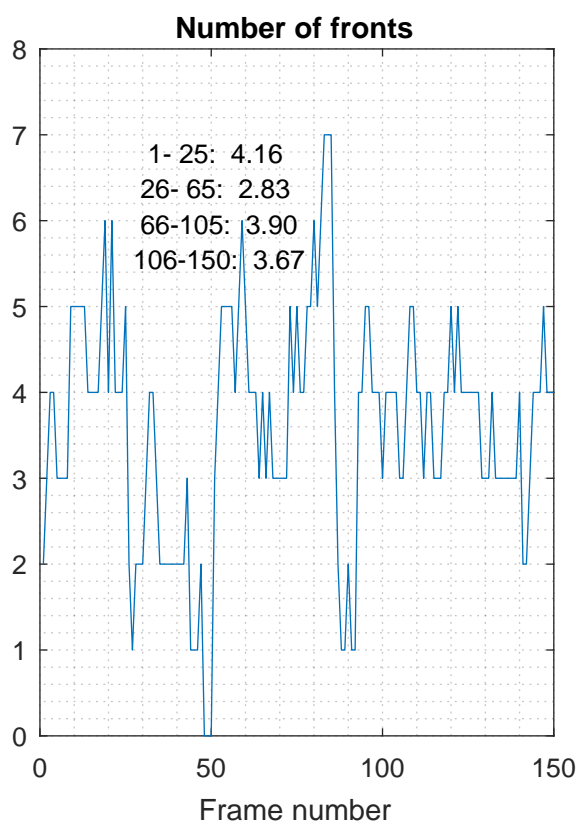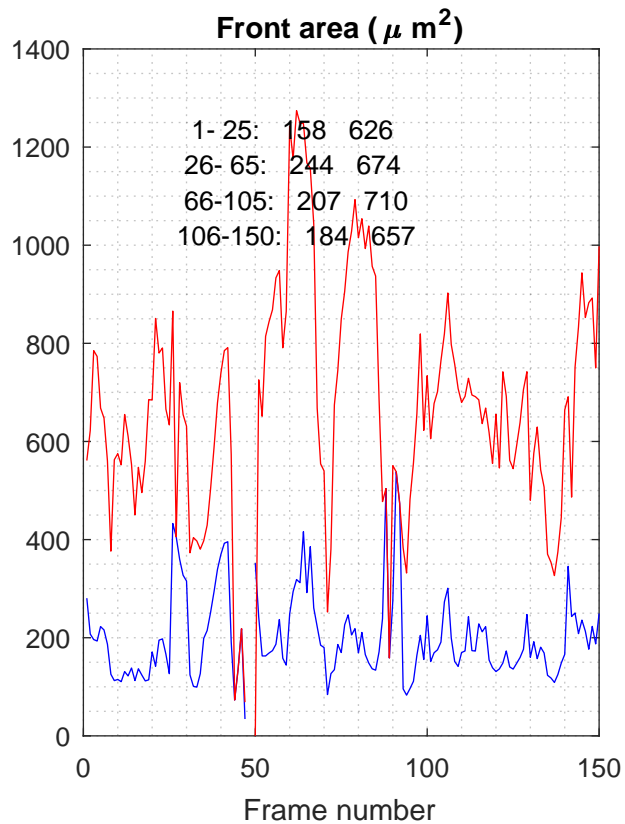

Supplement: Supplementary file 22 — Source Data for Figure 4 [file MSB-15-e8585-s020.zip › Source_data_for_Figure_4/Fig_4H/23.pdf]

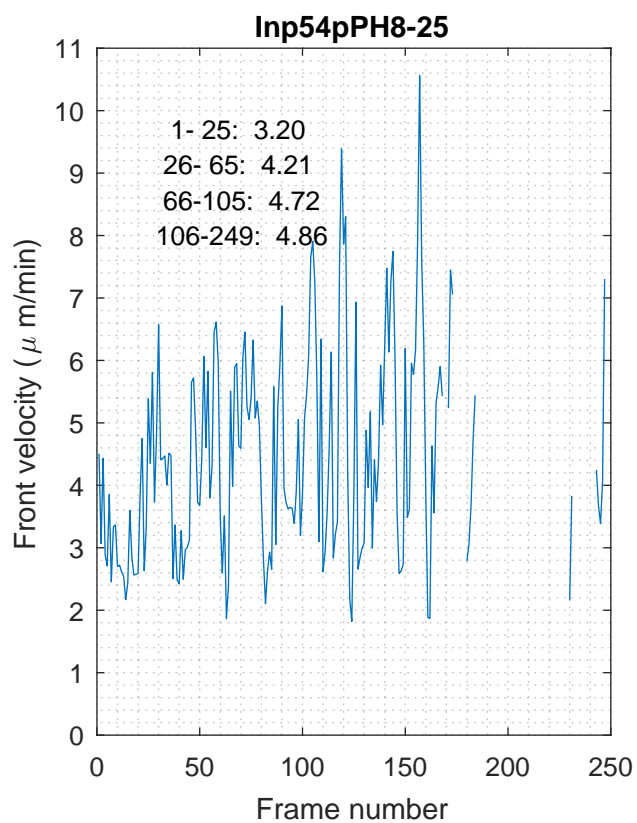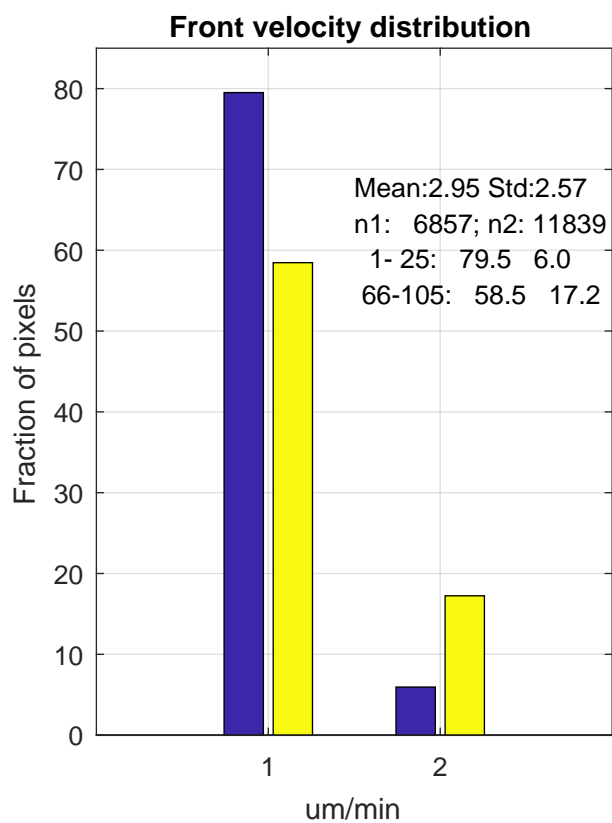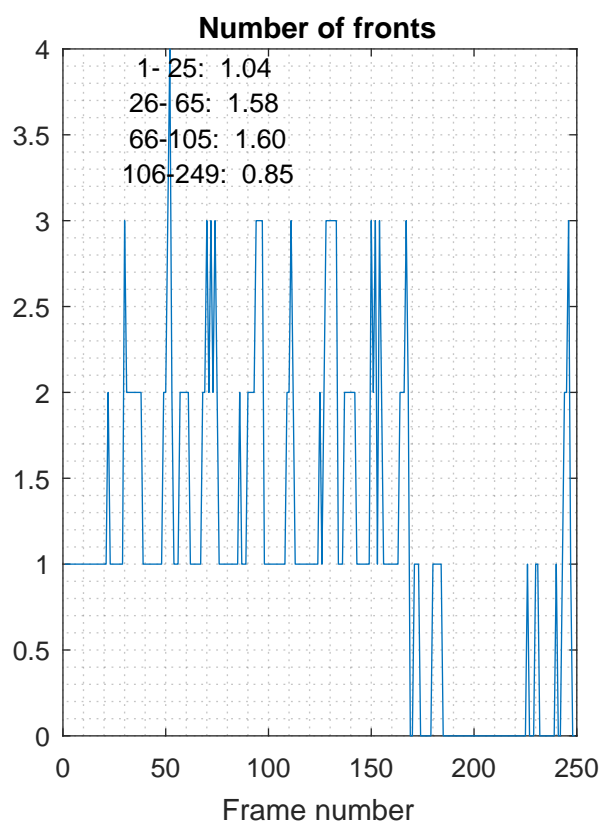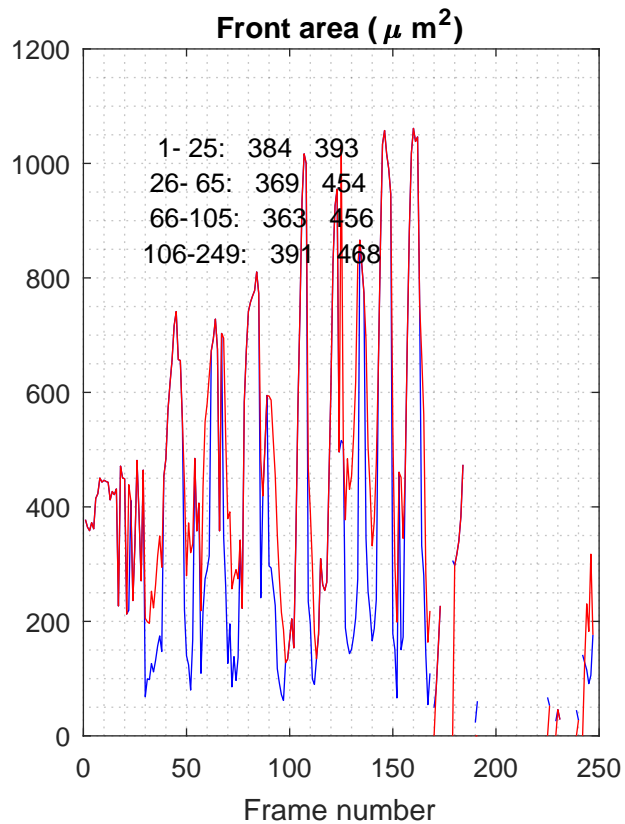

Supplement: Supplementary file 22 — Source Data for Figure 4 [file MSB-15-e8585-s020.zip › Source_data_for_Figure_4/Fig_4H/22.pdf]

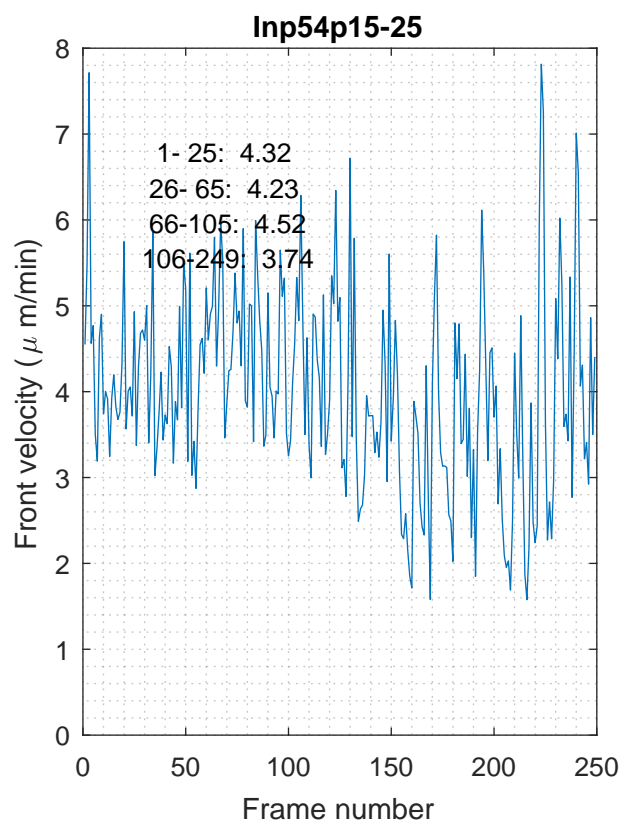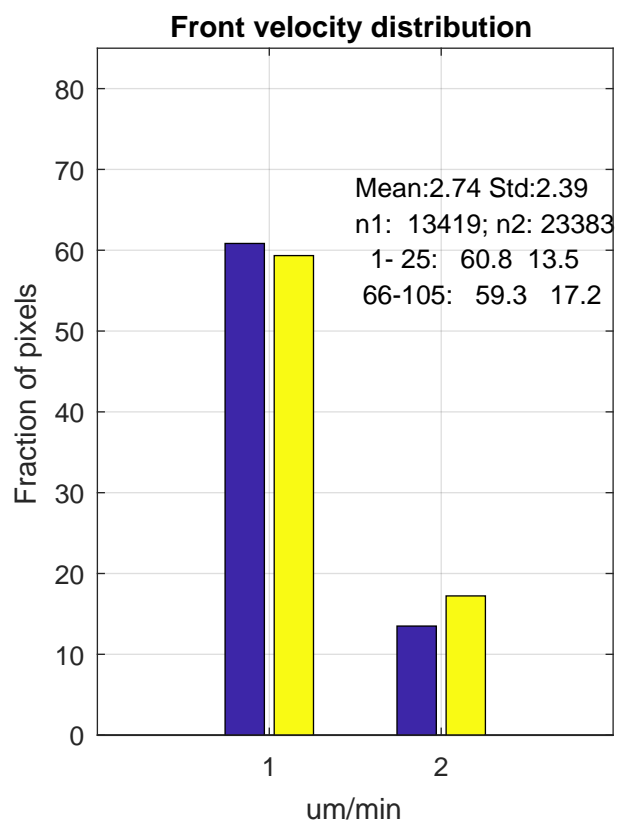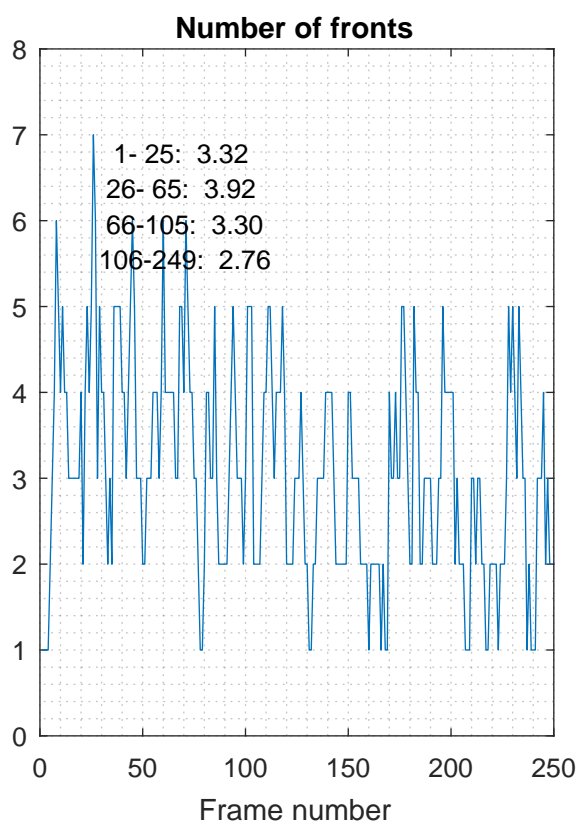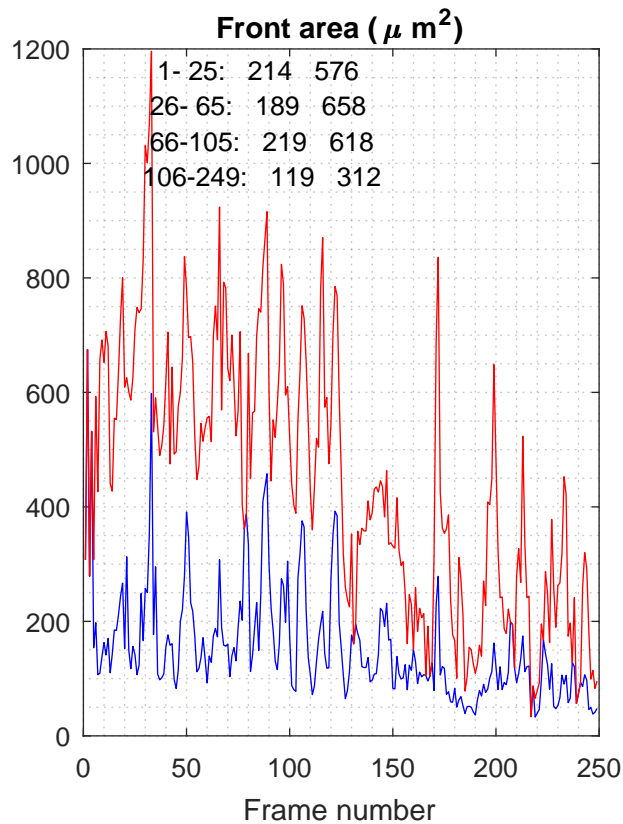

Supplement: Supplementary file 22 — Source Data for Figure 4 [file MSB-15-e8585-s020.zip › Source_data_for_Figure_4/Fig_4H/20.pdf]

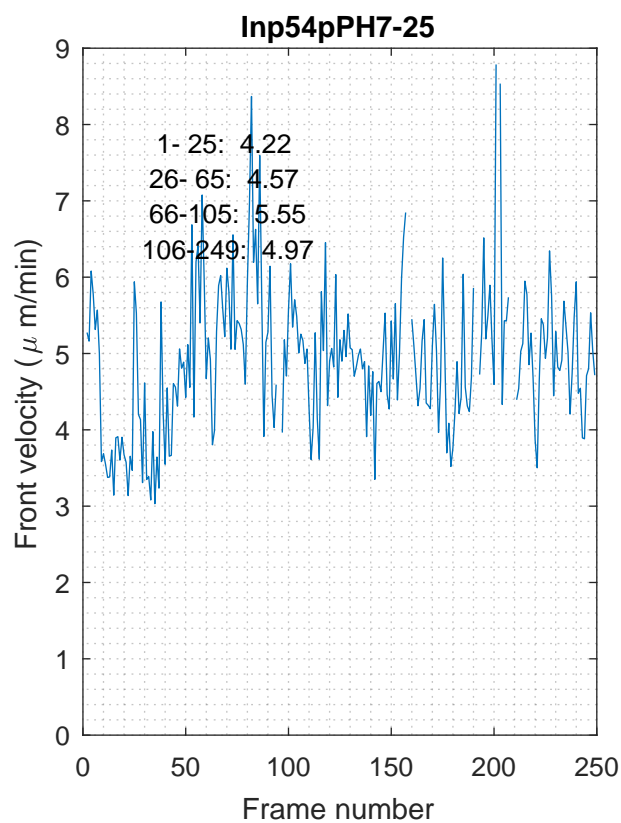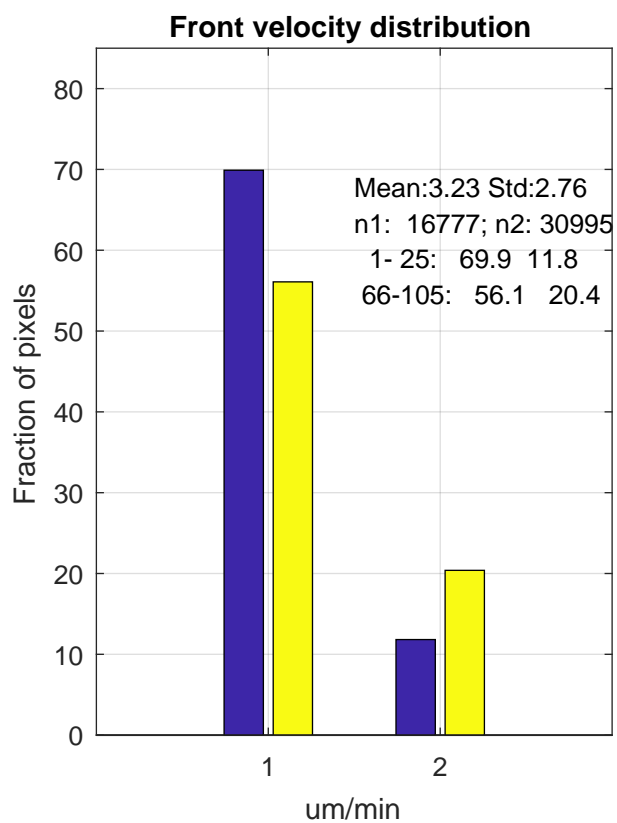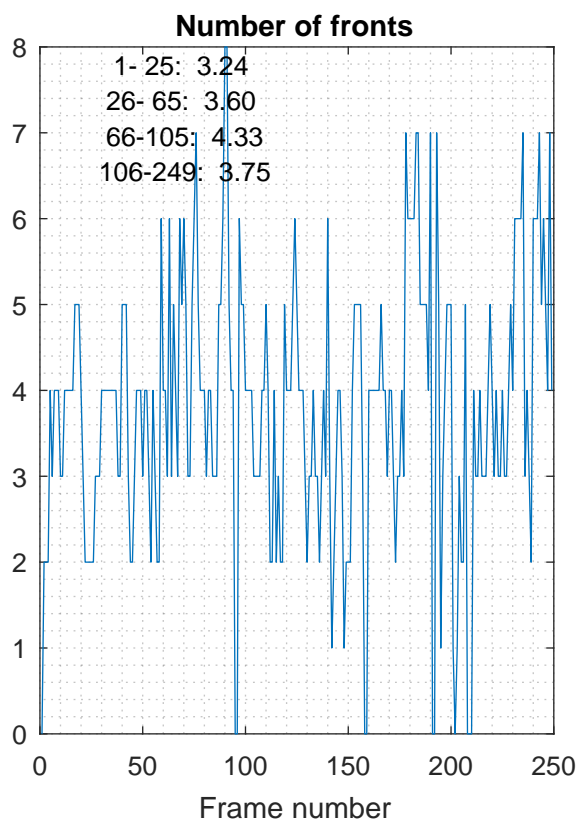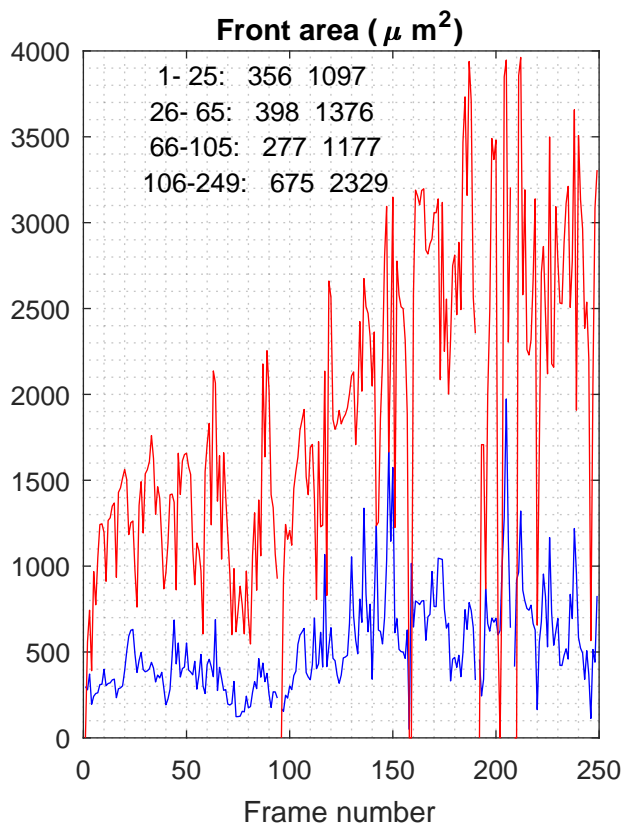

Supplement: Supplementary file 22 — Source Data for Figure 4 [file MSB-15-e8585-s020.zip › Source_data_for_Figure_4/Fig_4H/21.pdf]

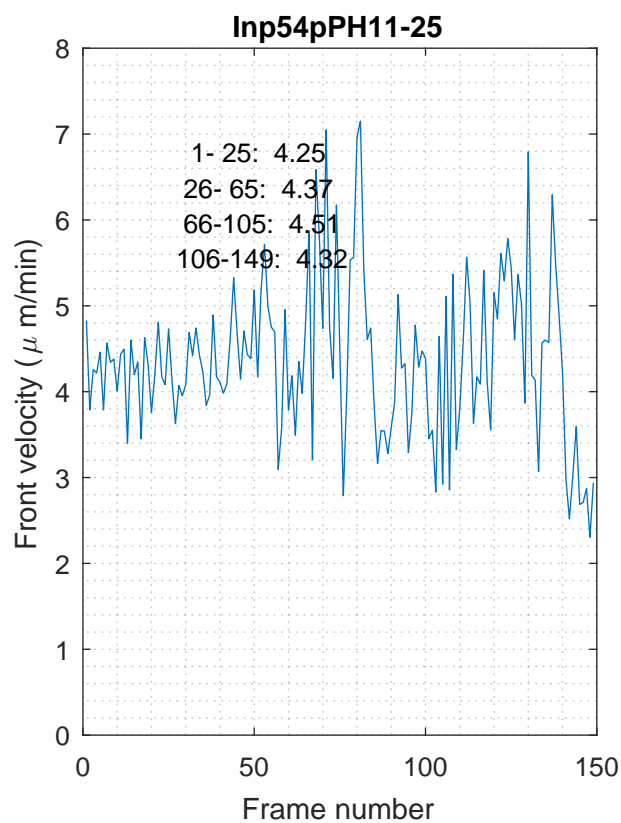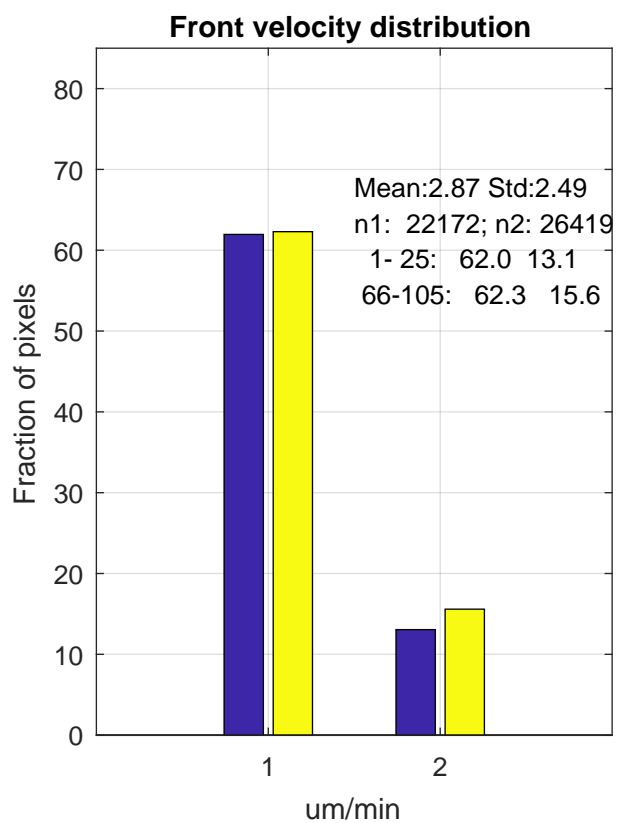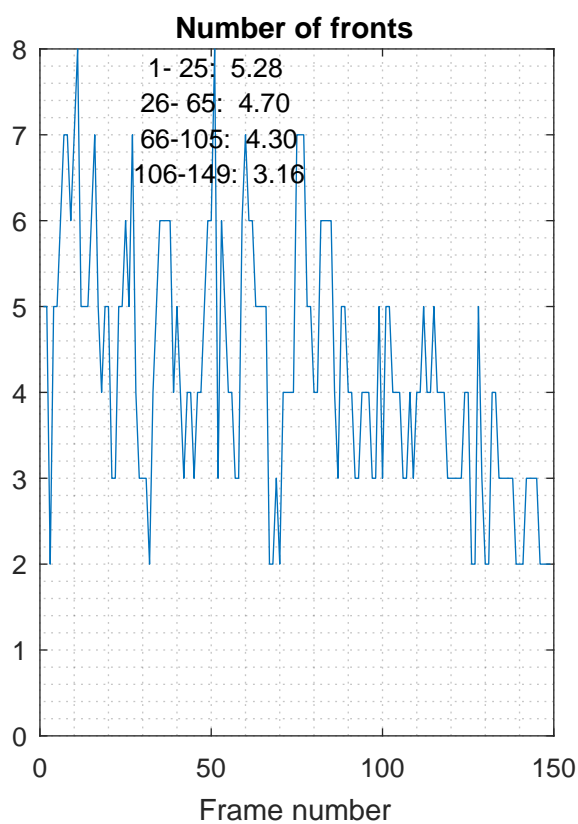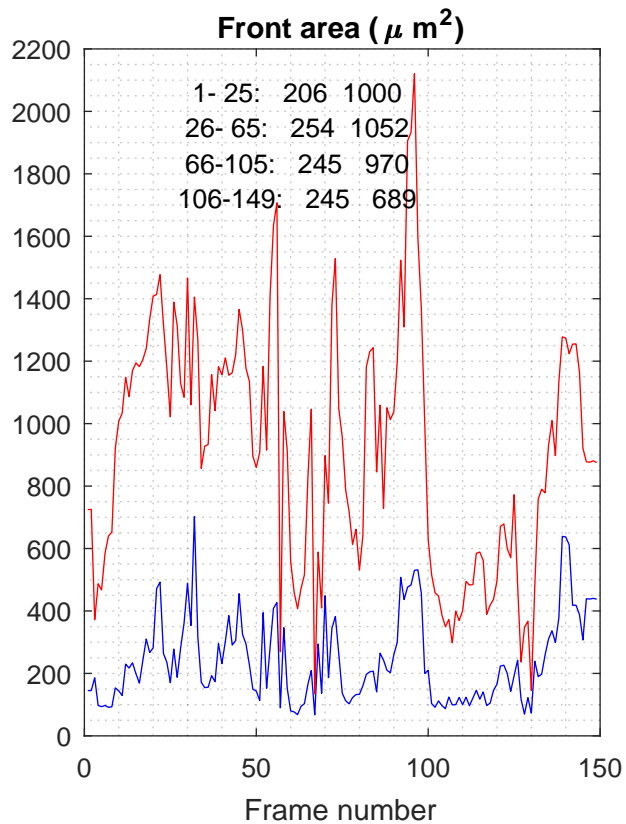

Supplement: Supplementary file 22 — Source Data for Figure 4 [file MSB-15-e8585-s020.zip › Source_data_for_Figure_4/Fig_4H/25.pdf]

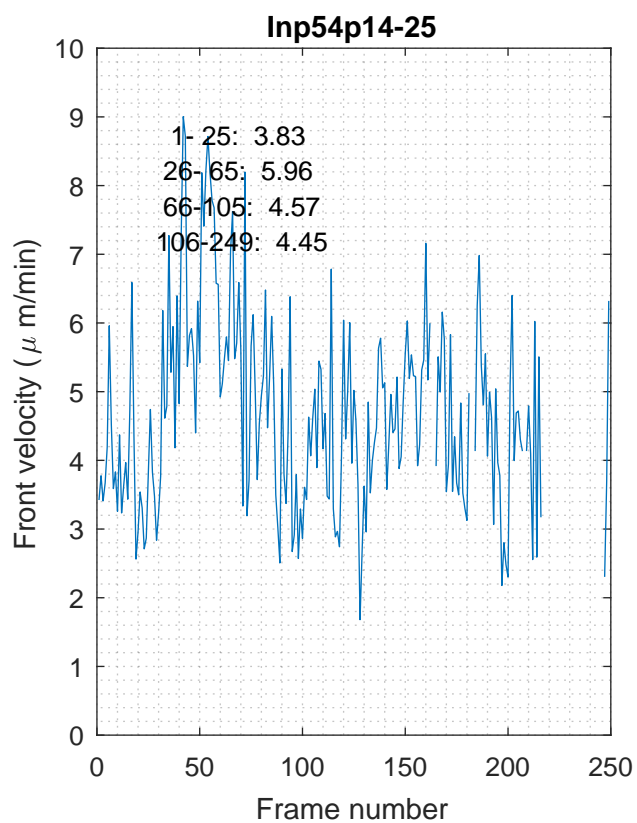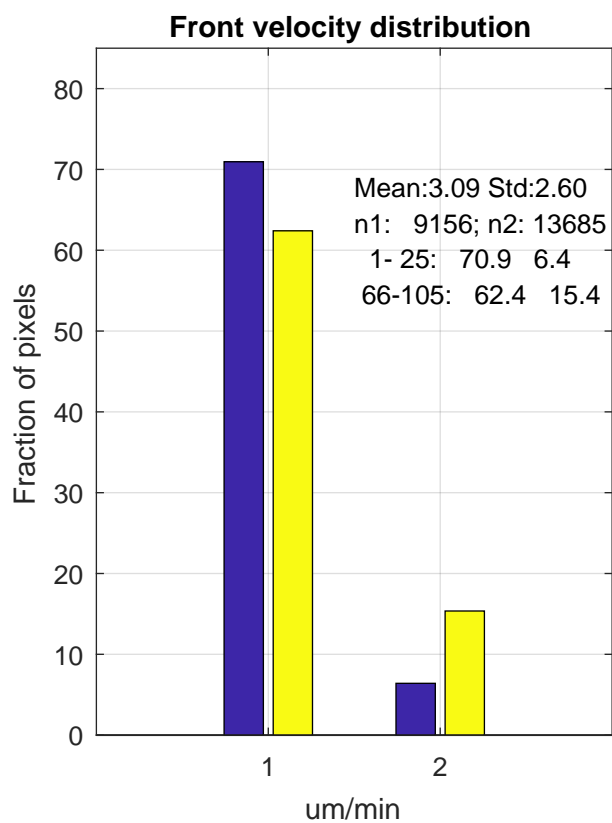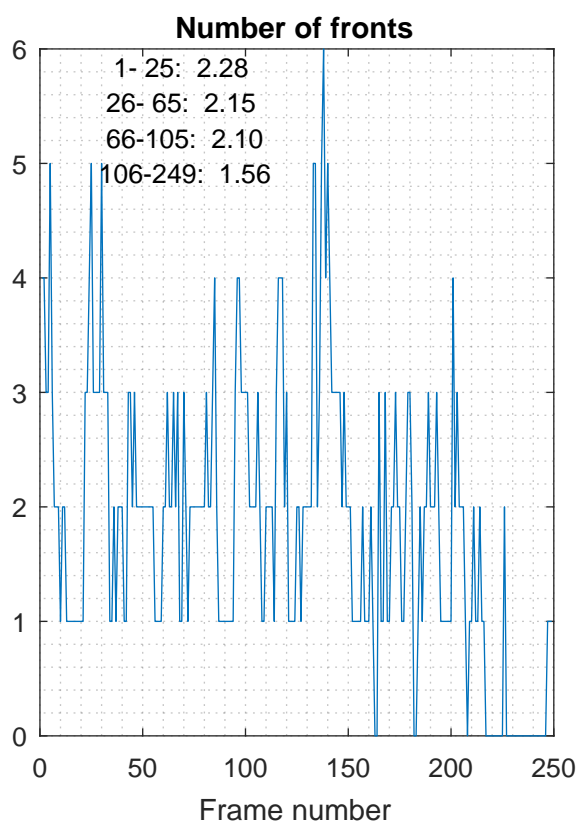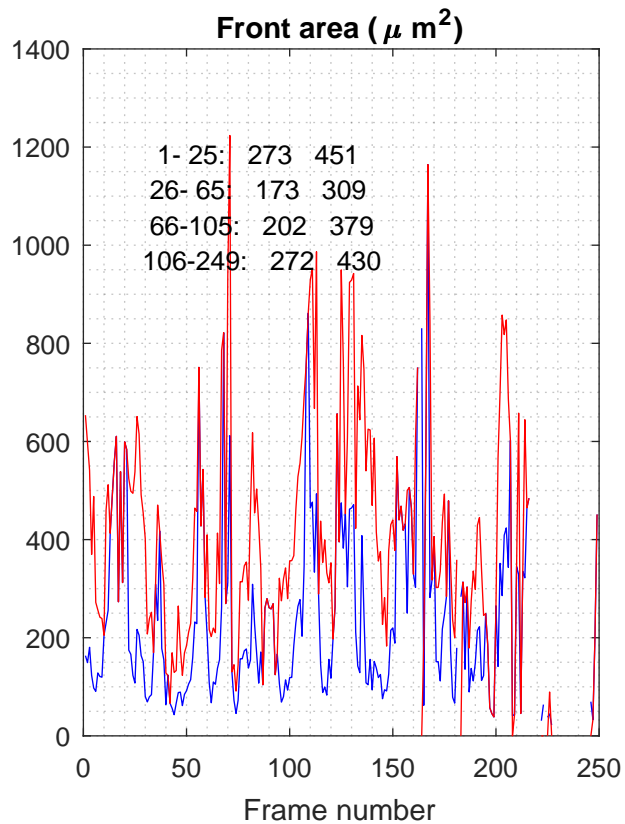

Supplement: Supplementary file 22 — Source Data for Figure 4 [file MSB-15-e8585-s020.zip › Source_data_for_Figure_4/Fig_4H/19.pdf]

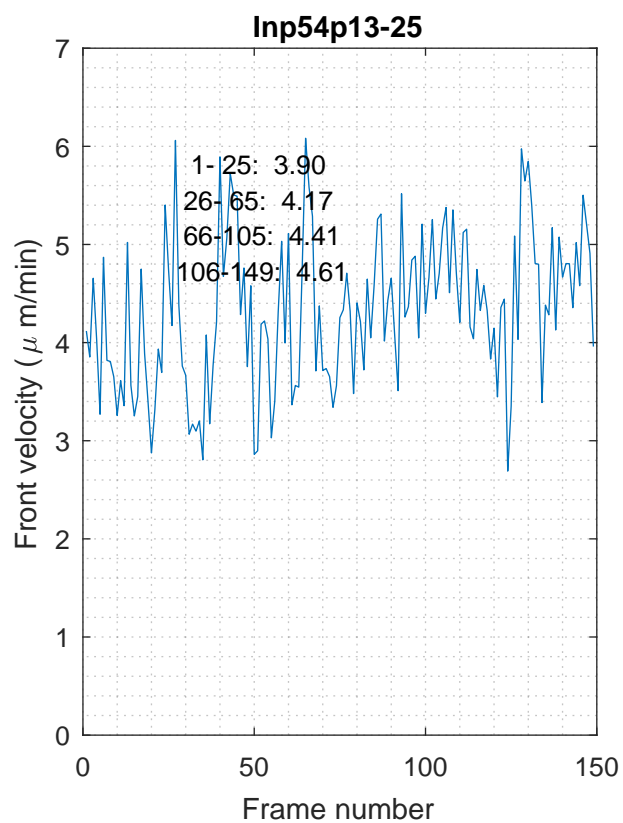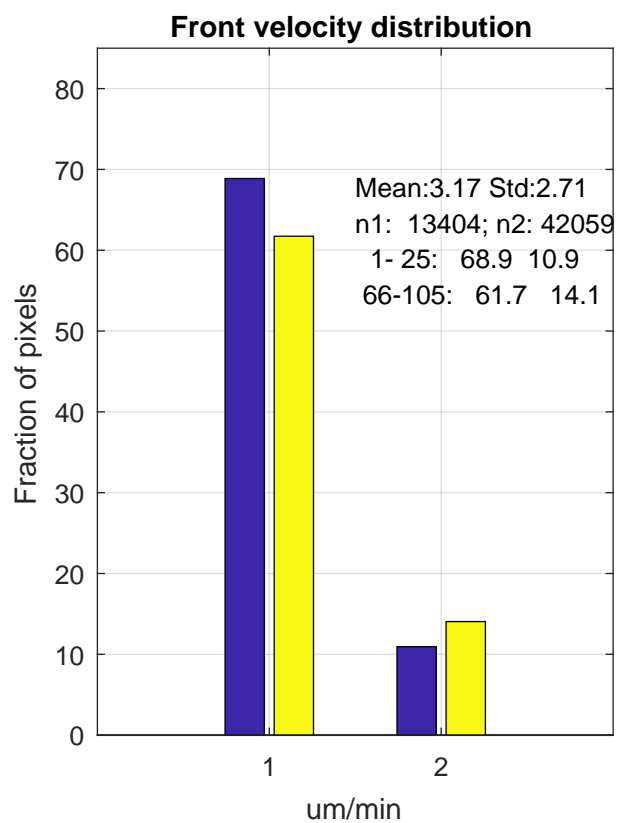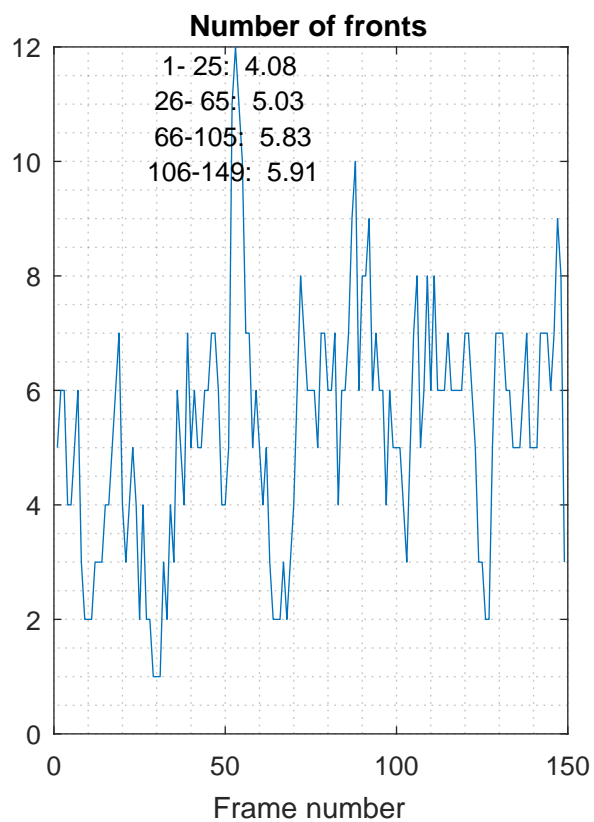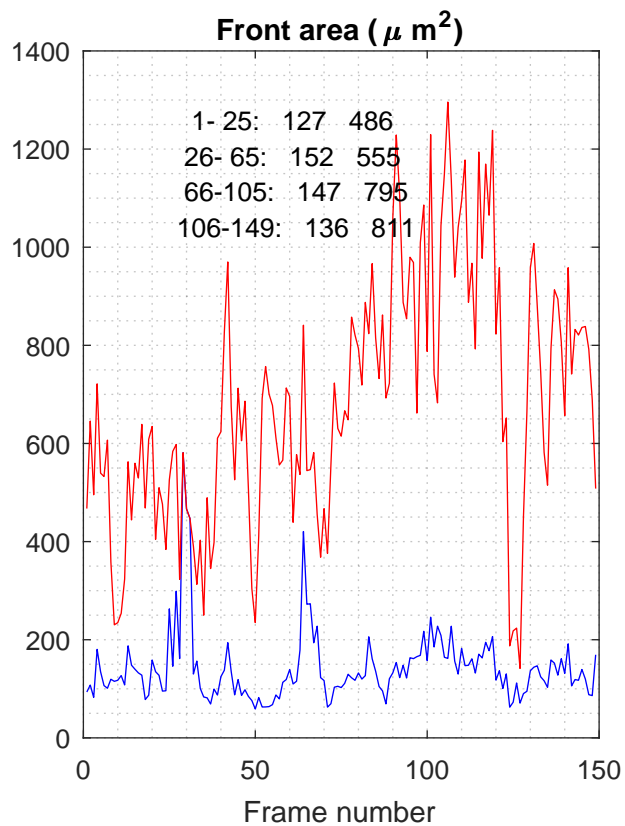

Supplement: Supplementary file 22 — Source Data for Figure 4 [file MSB-15-e8585-s020.zip › Source_data_for_Figure_4/Fig_4H/18.pdf]

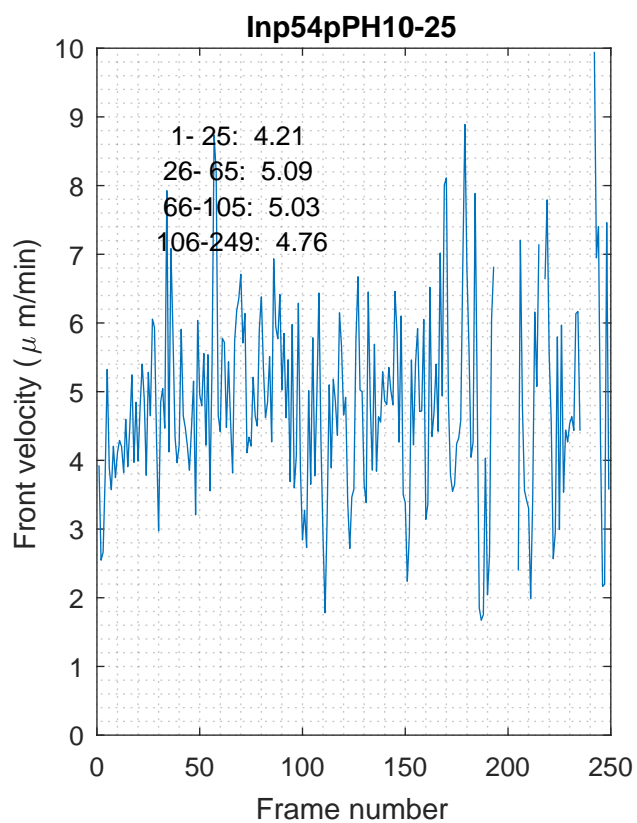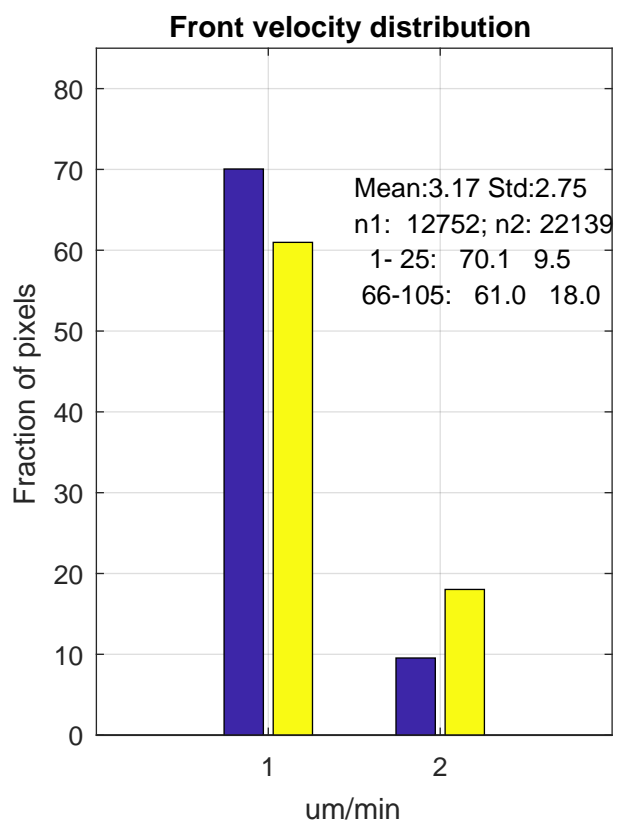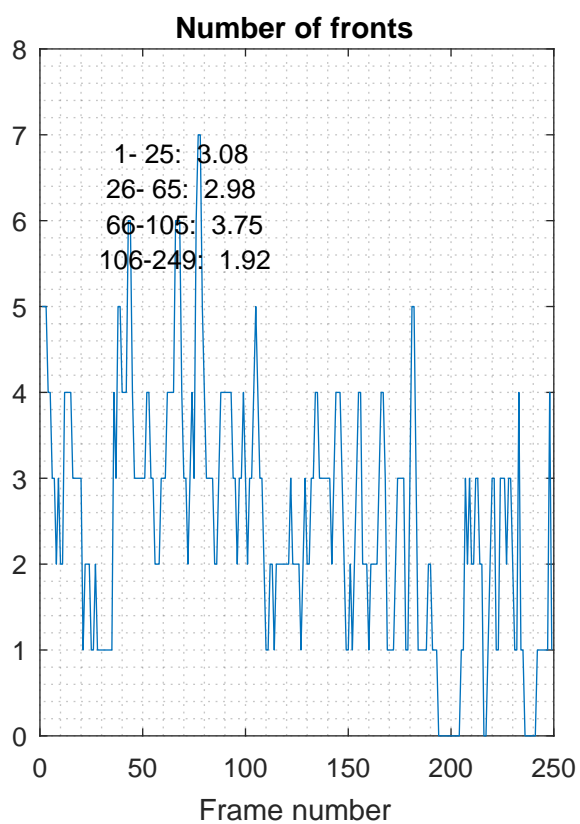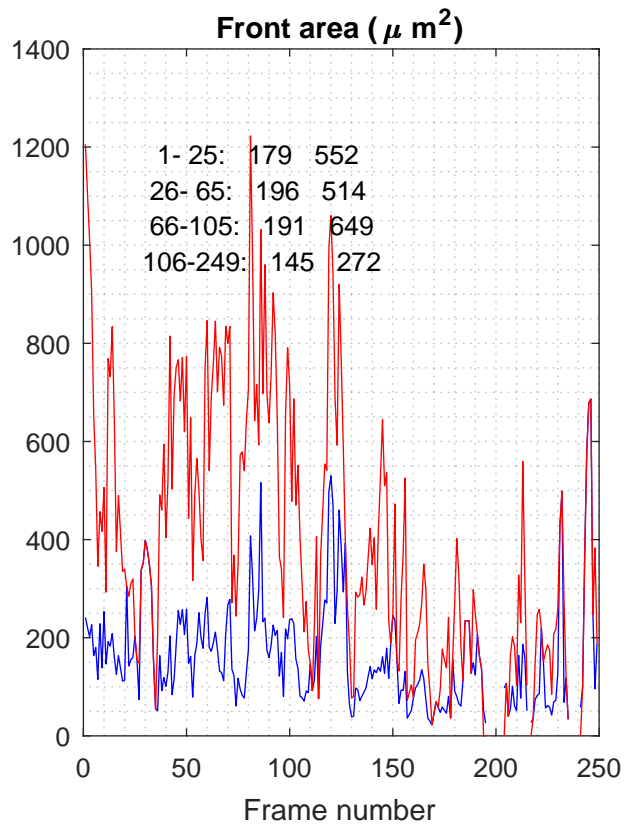

Supplement: Supplementary file 22 — Source Data for Figure 4 [file MSB-15-e8585-s020.zip › Source_data_for_Figure_4/Fig_4H/24.pdf]

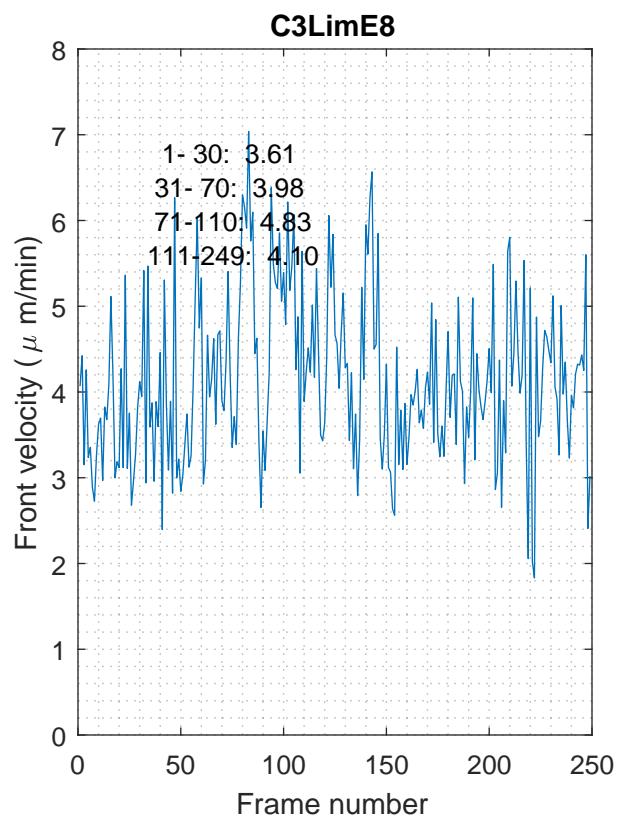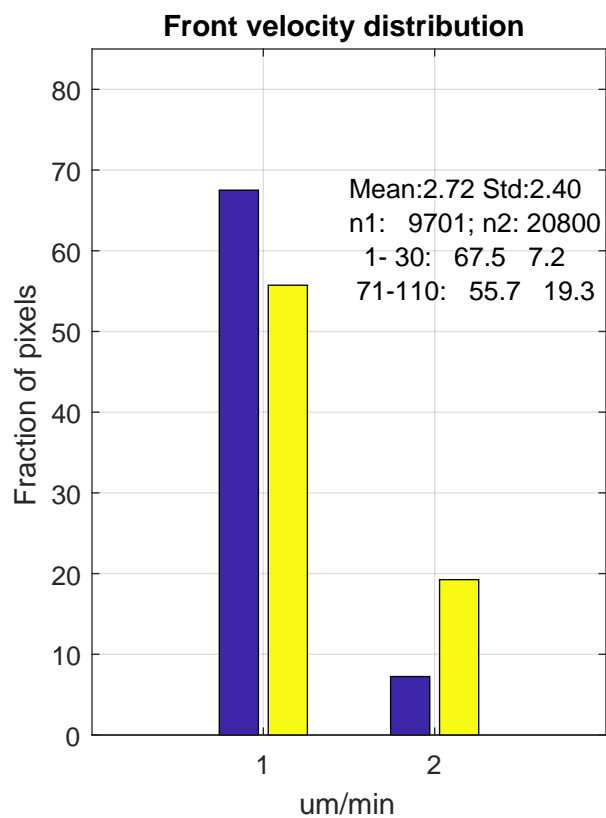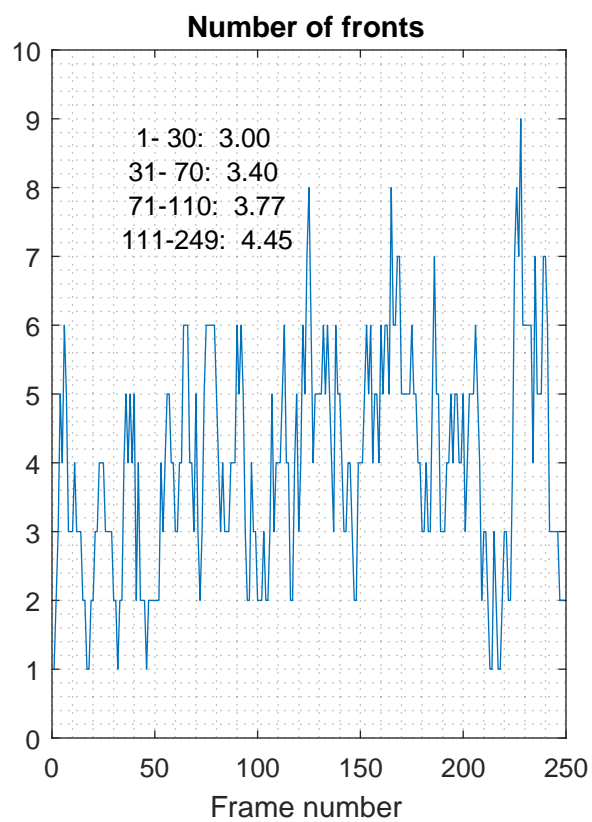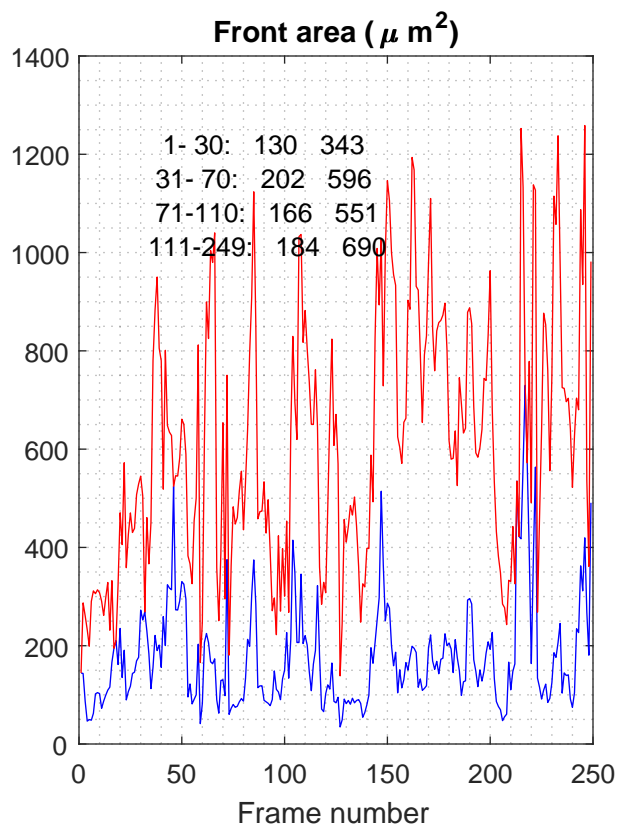

Supplement: Supplementary file 22 — Source Data for Figure 4 [file MSB-15-e8585-s020.zip › Source_data_for_Figure_4/Fig_4H/6.pdf]

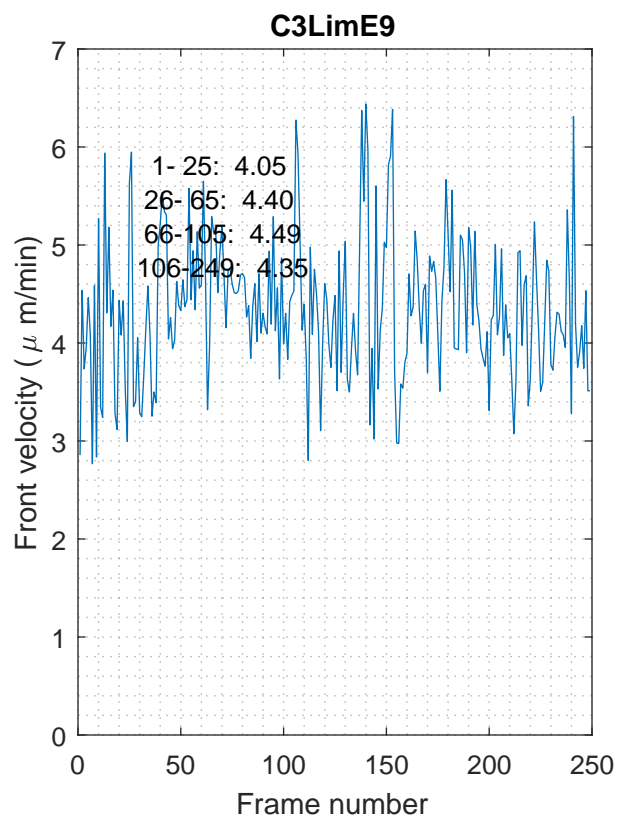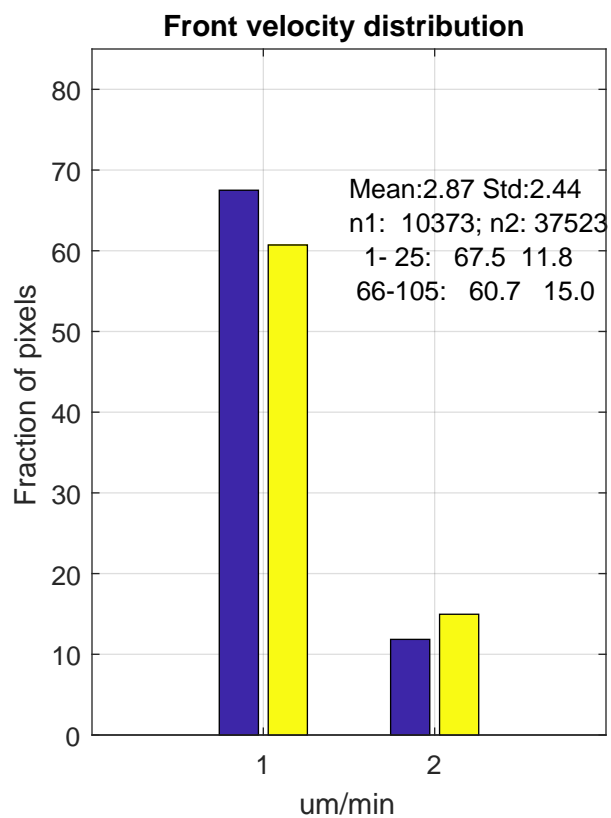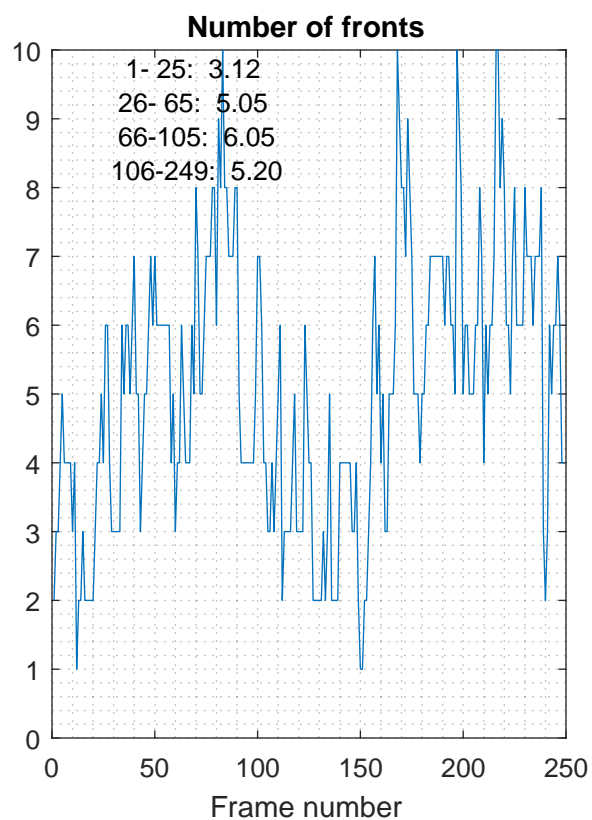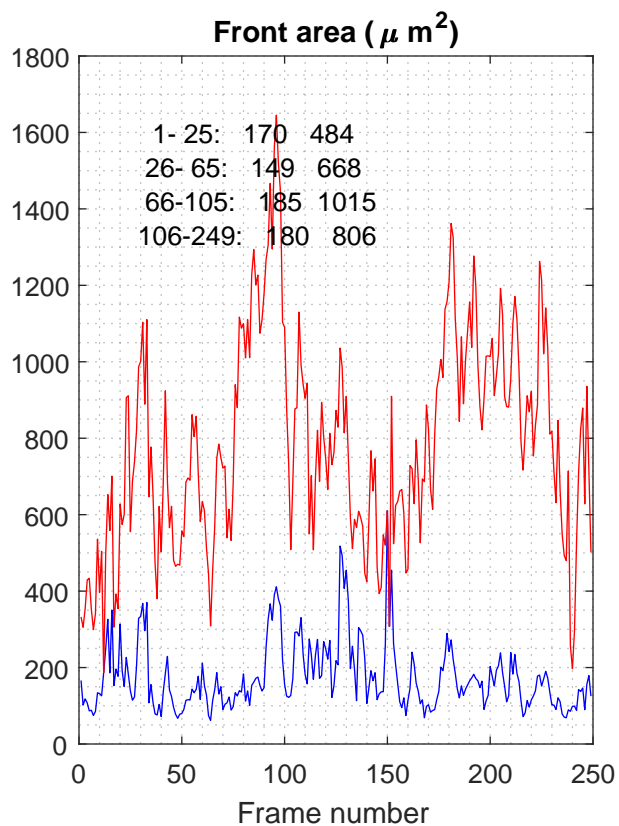

Supplement: Supplementary file 22 — Source Data for Figure 4 [file MSB-15-e8585-s020.zip › Source_data_for_Figure_4/Fig_4H/7.pdf]

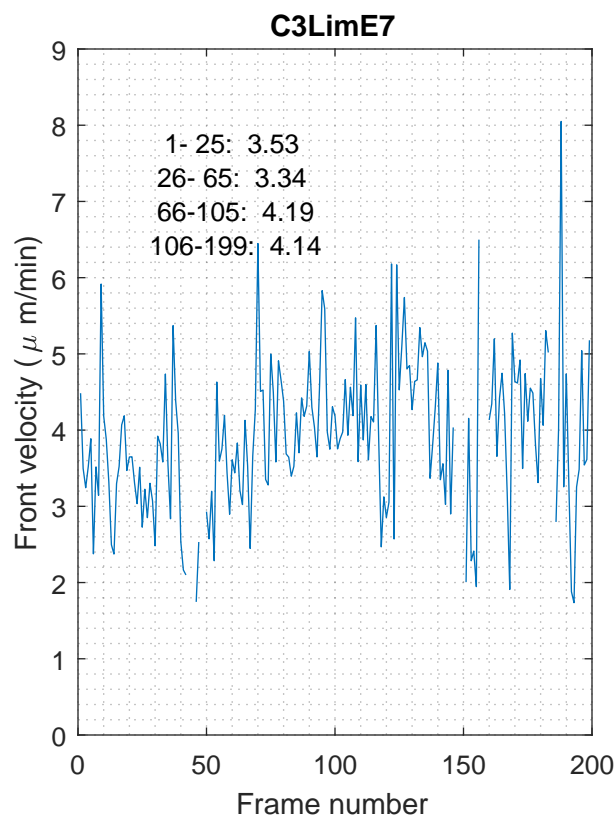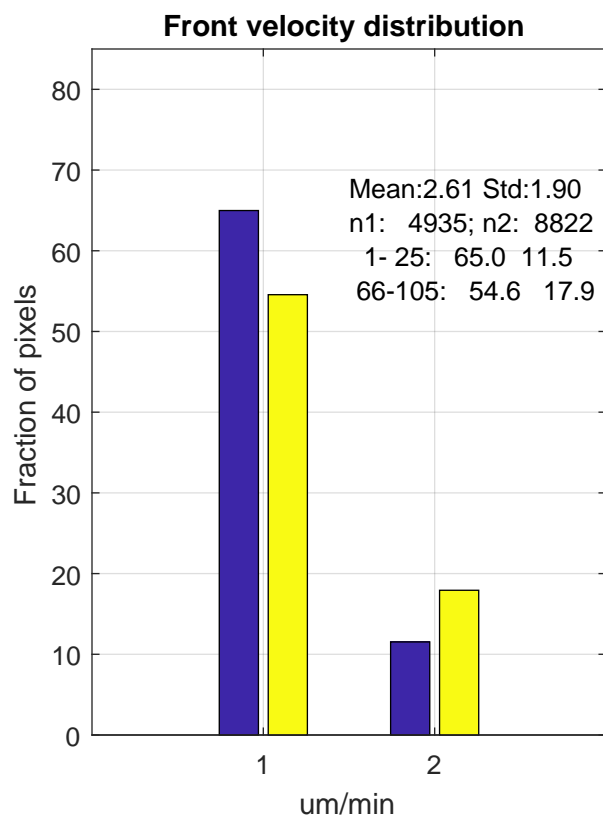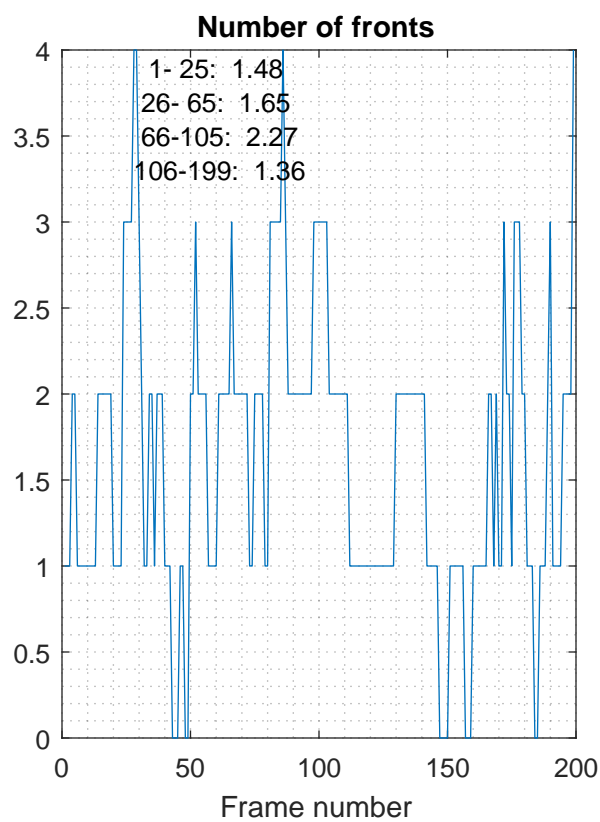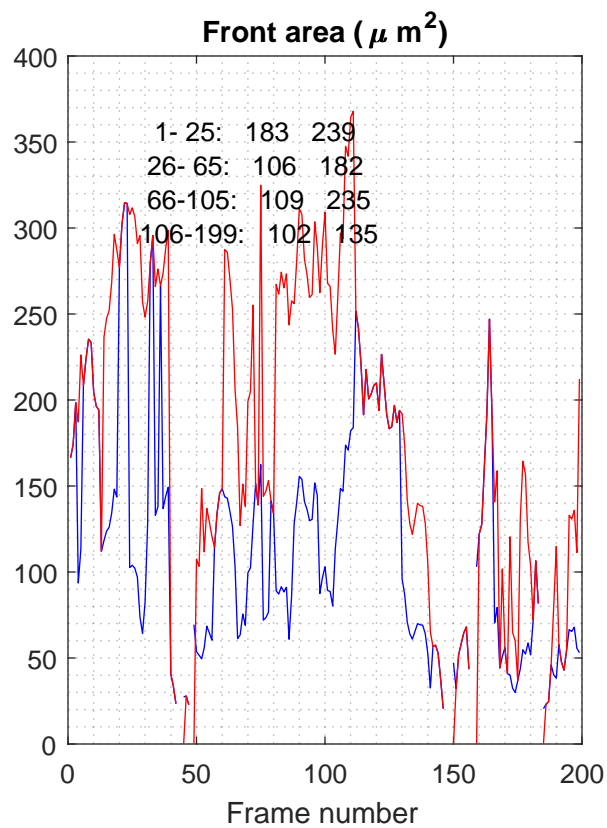

Supplement: Supplementary file 22 — Source Data for Figure 4 [file MSB-15-e8585-s020.zip › Source_data_for_Figure_4/Fig_4H/5.pdf]

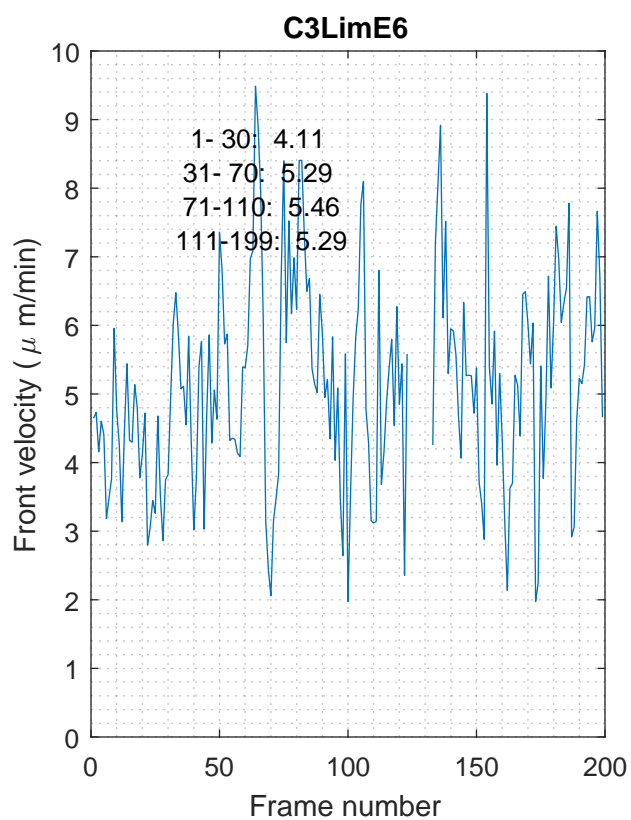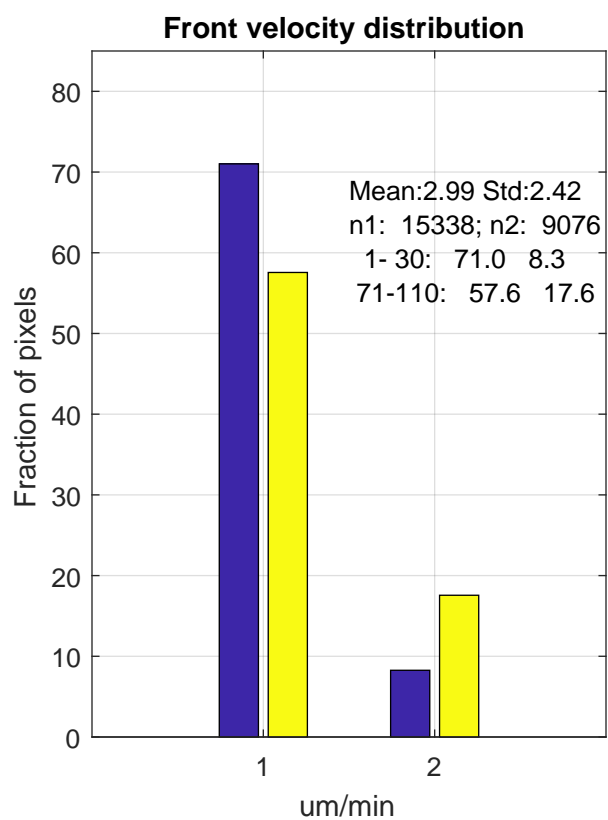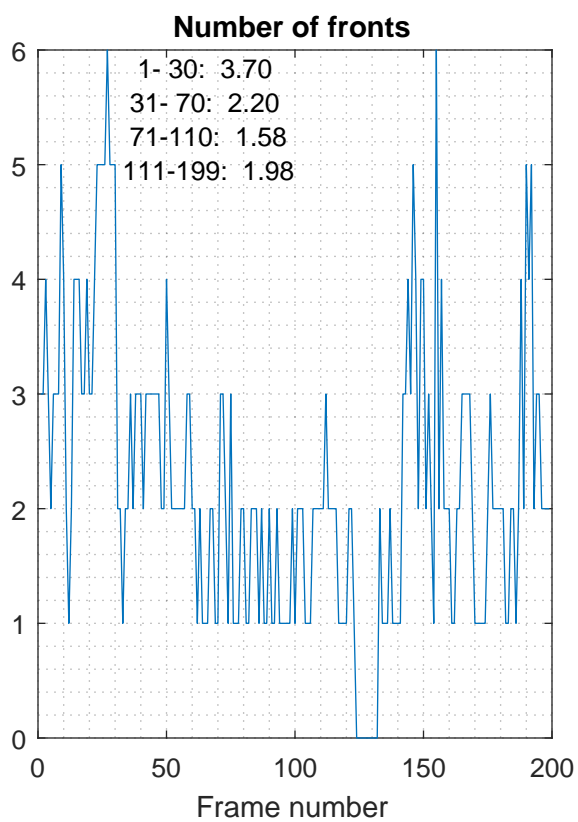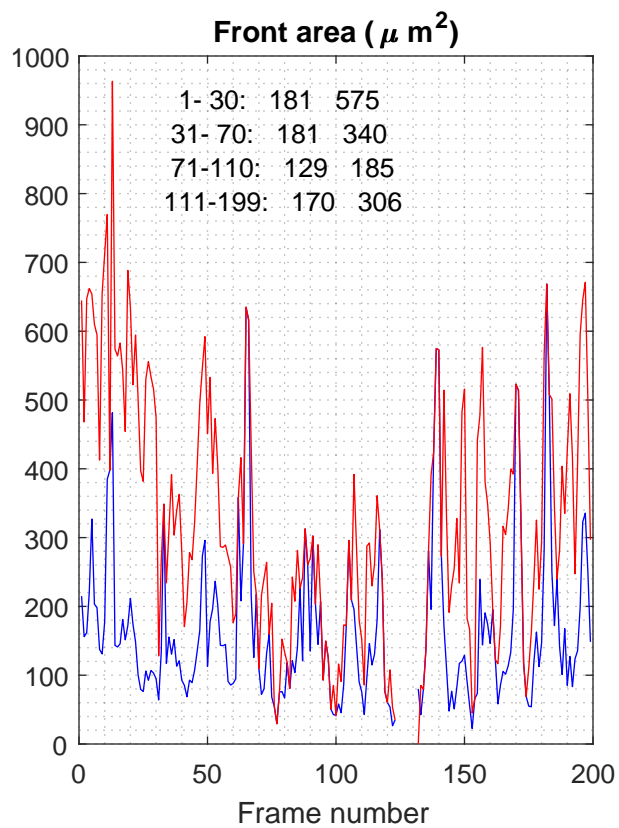

Supplement: Supplementary file 22 — Source Data for Figure 4 [file MSB-15-e8585-s020.zip › Source_data_for_Figure_4/Fig_4H/4.pdf]

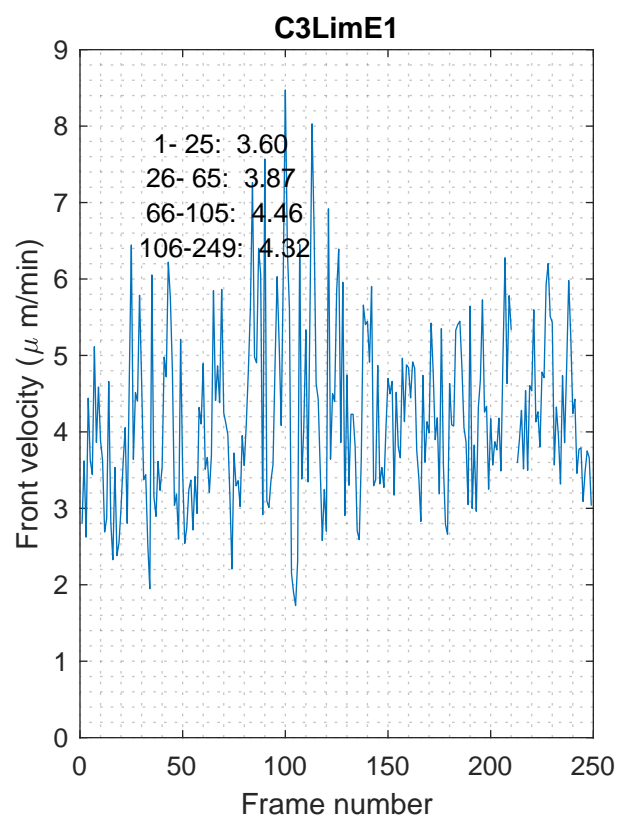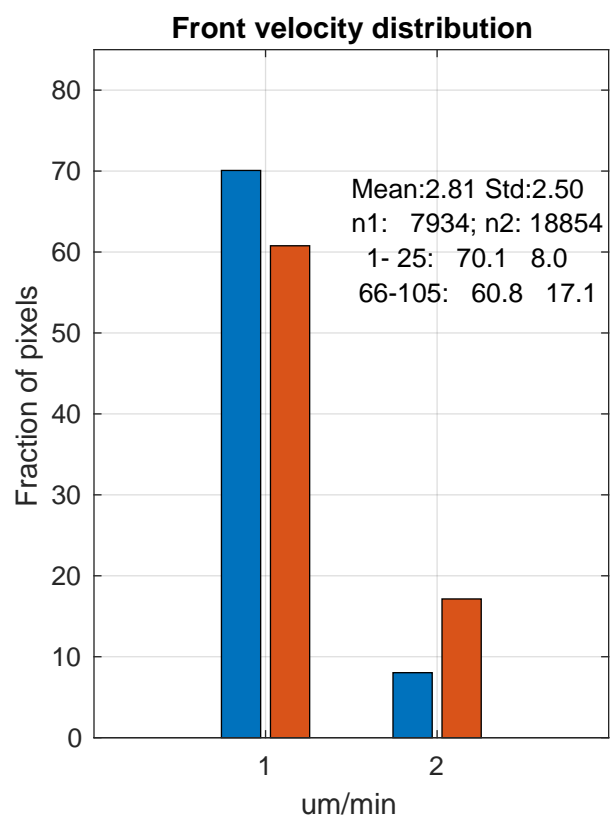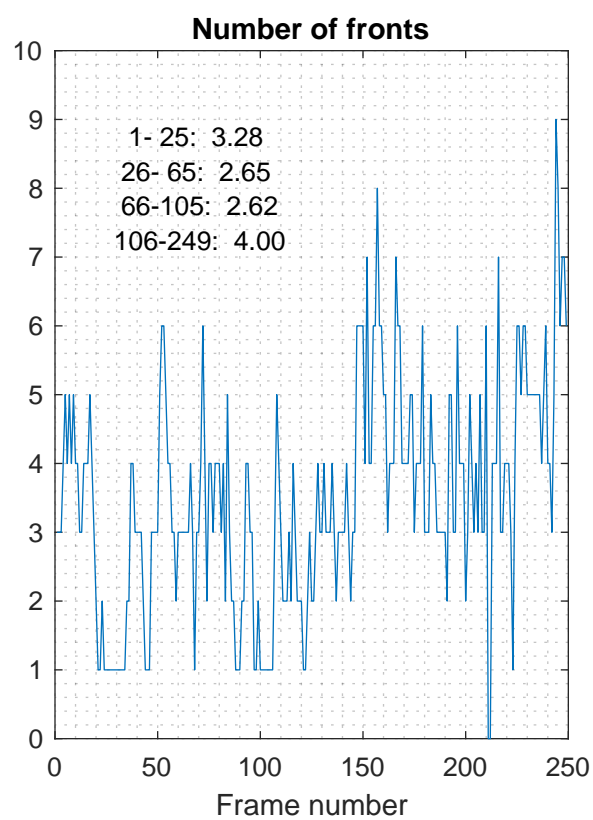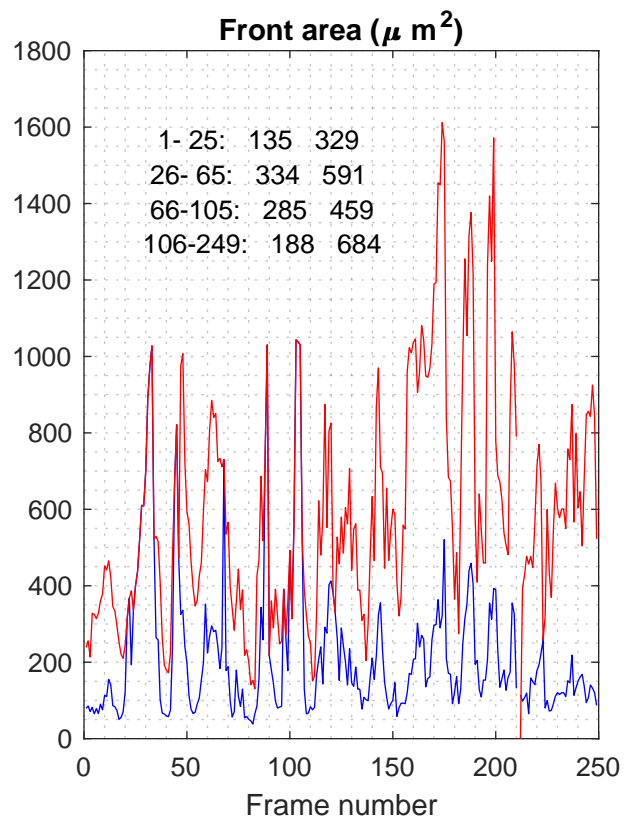

Supplement: Supplementary file 22 — Source Data for Figure 4 [file MSB-15-e8585-s020.zip › Source_data_for_Figure_4/Fig_4H/1.pdf]

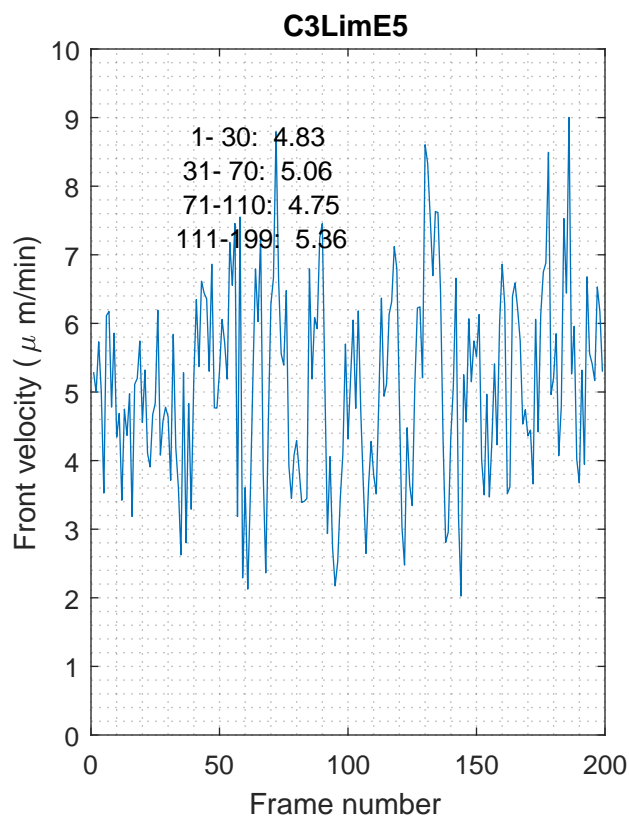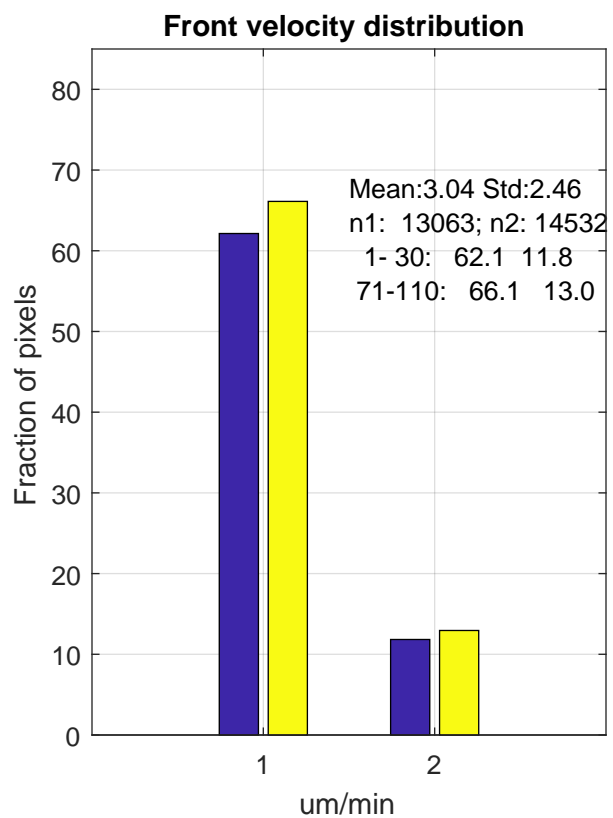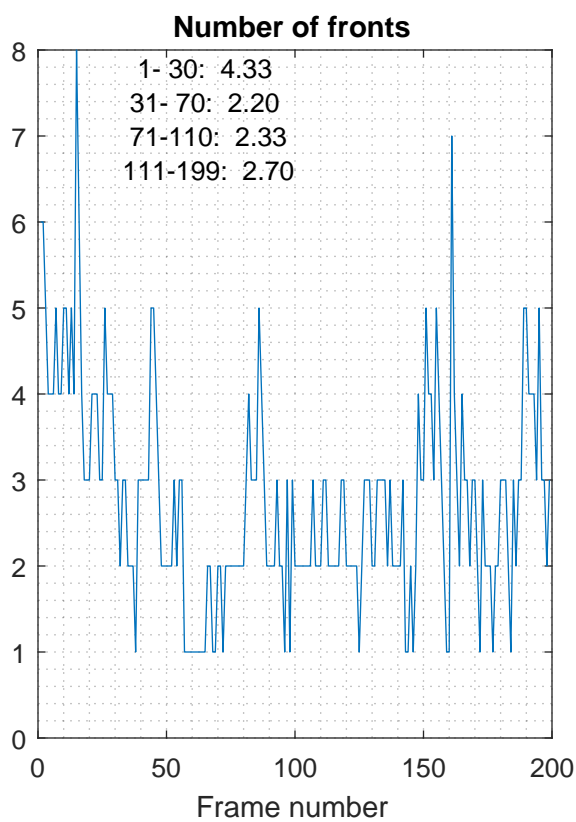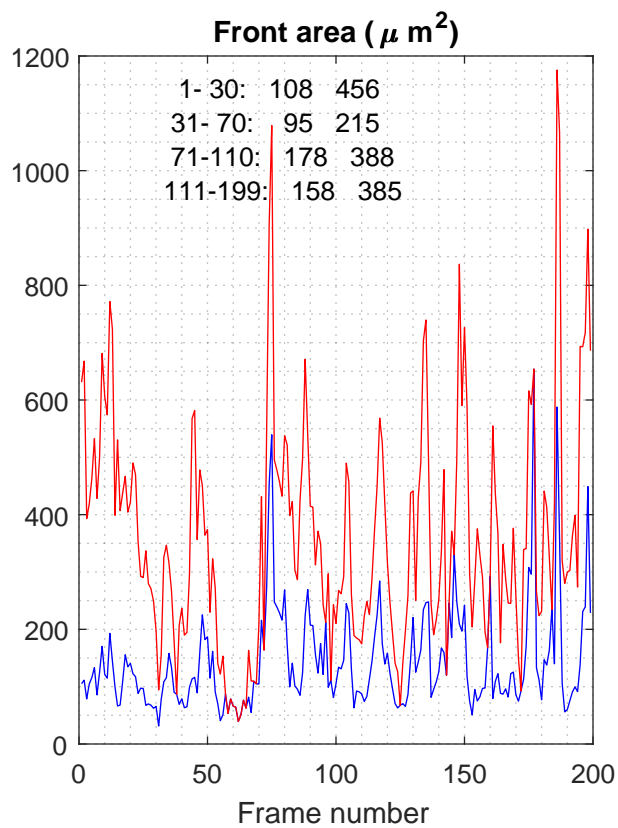

Supplement: Supplementary file 22 — Source Data for Figure 4 [file MSB-15-e8585-s020.zip › Source_data_for_Figure_4/Fig_4H/3.pdf]

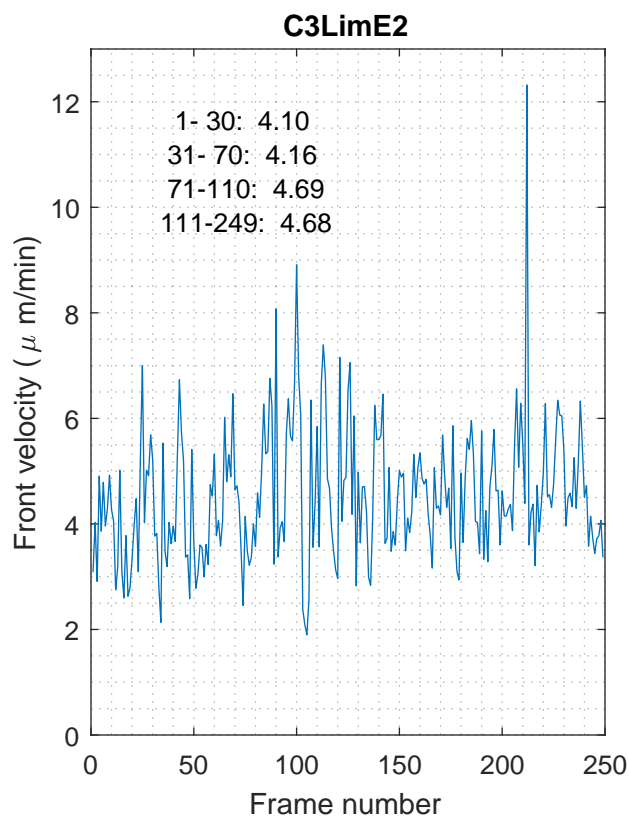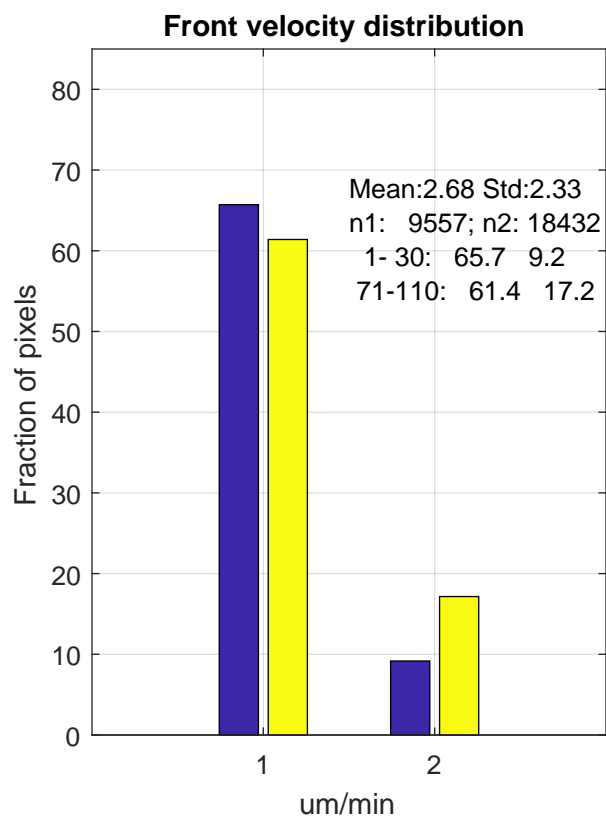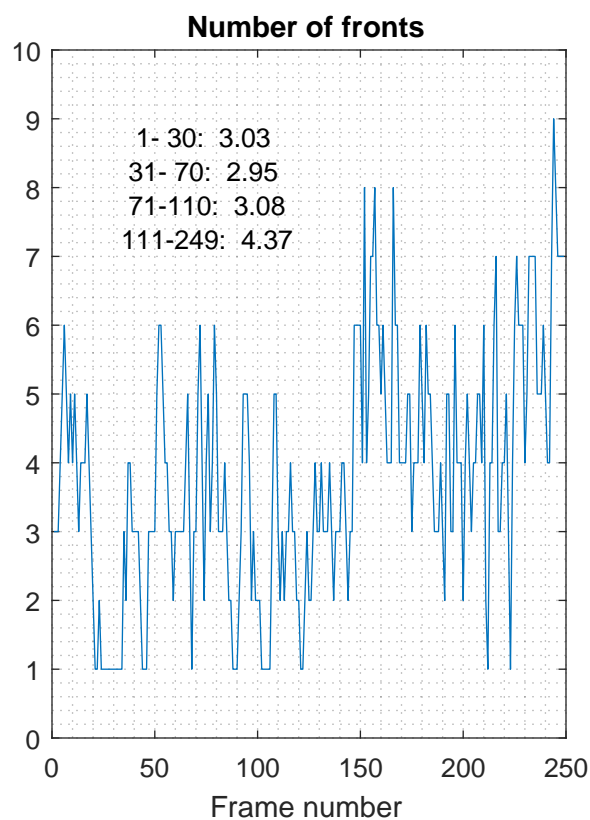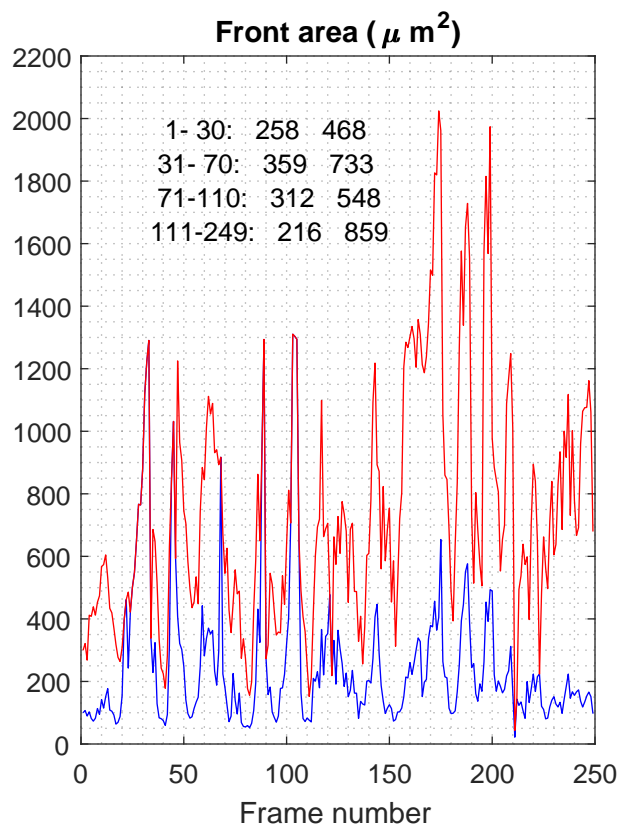

Supplement: Supplementary file 22 — Source Data for Figure 4 [file MSB-15-e8585-s020.zip › Source_data_for_Figure_4/Fig_4H/2.pdf]

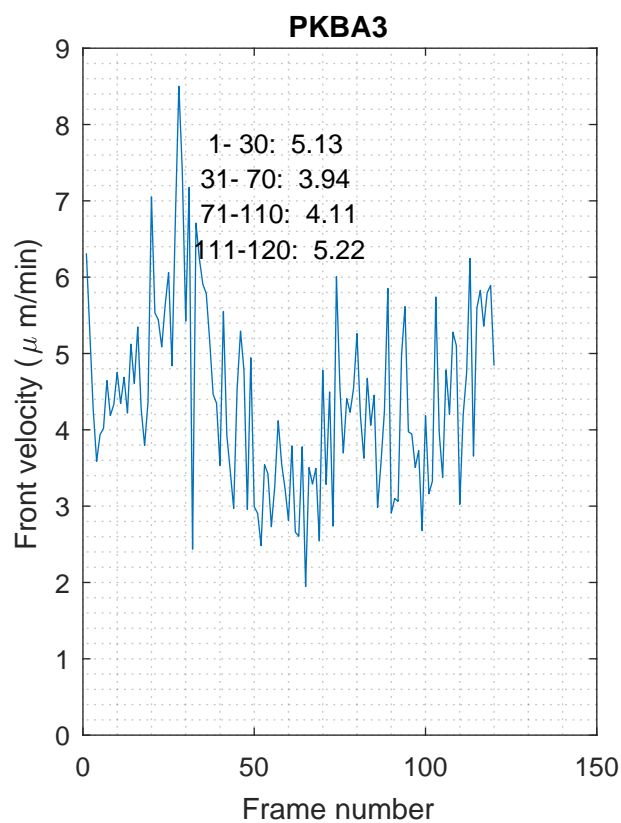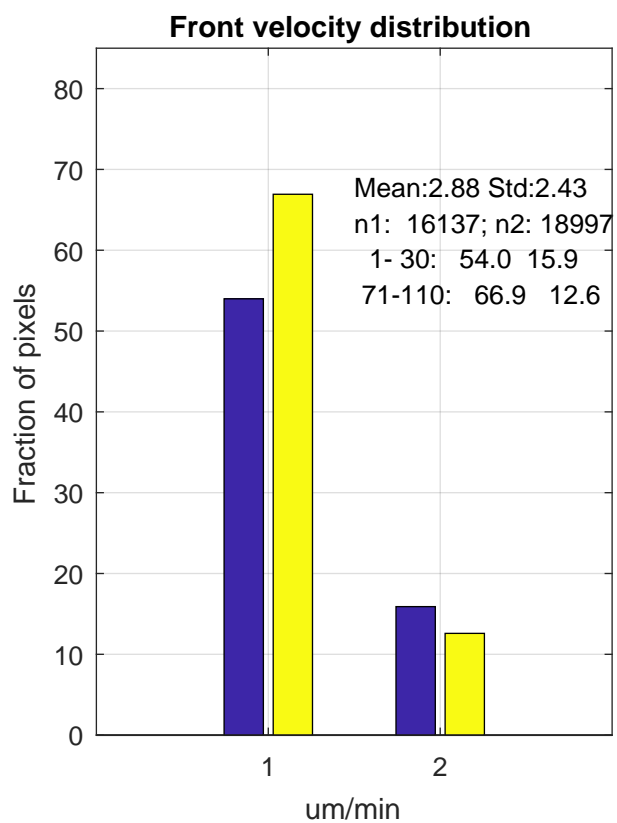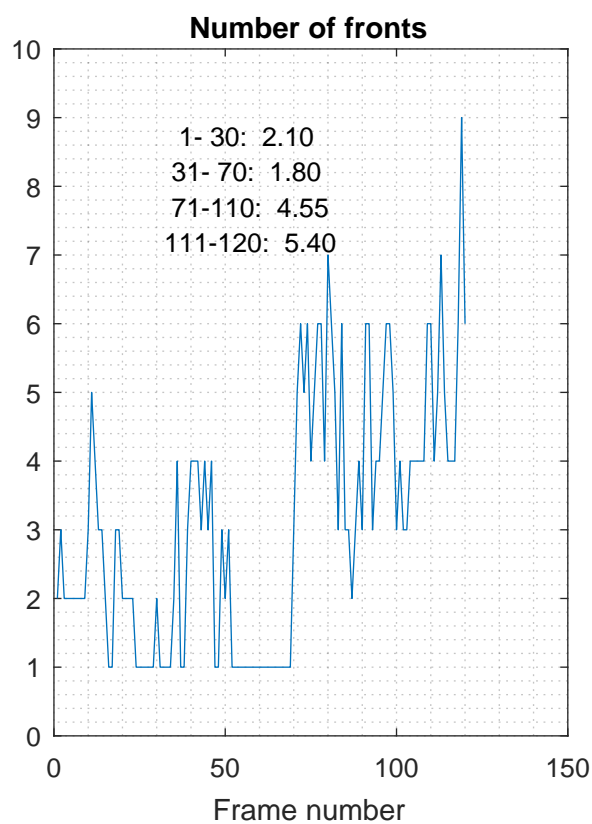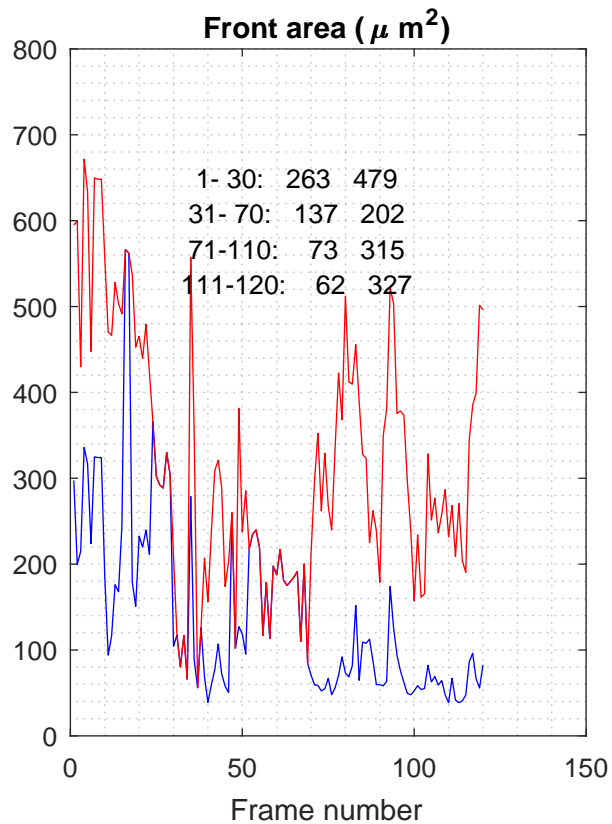

Supplement: Supplementary file 23 — Source Data for Figure 5 [file MSB-15-e8585-s021.zip › Source_data_for_Figure_5/Fig_5C/PKBA3.pdf]

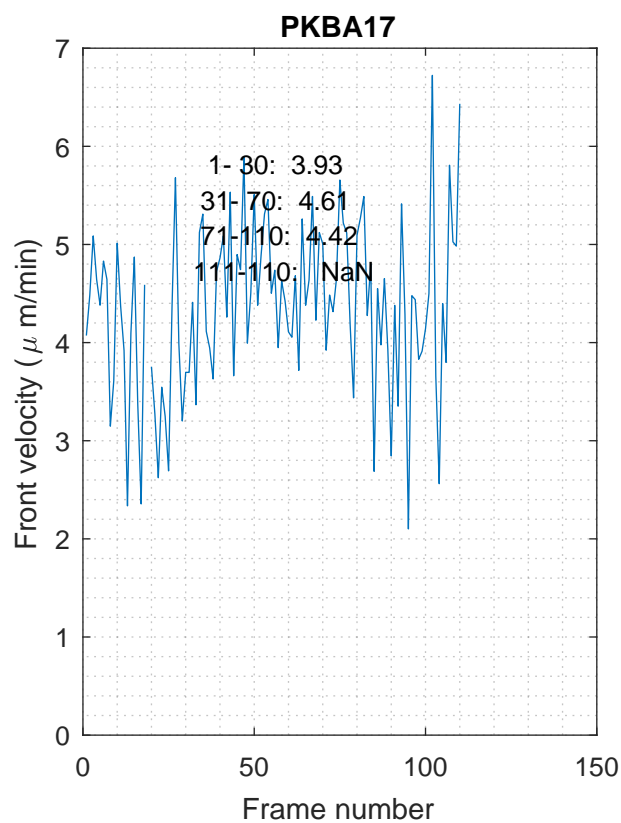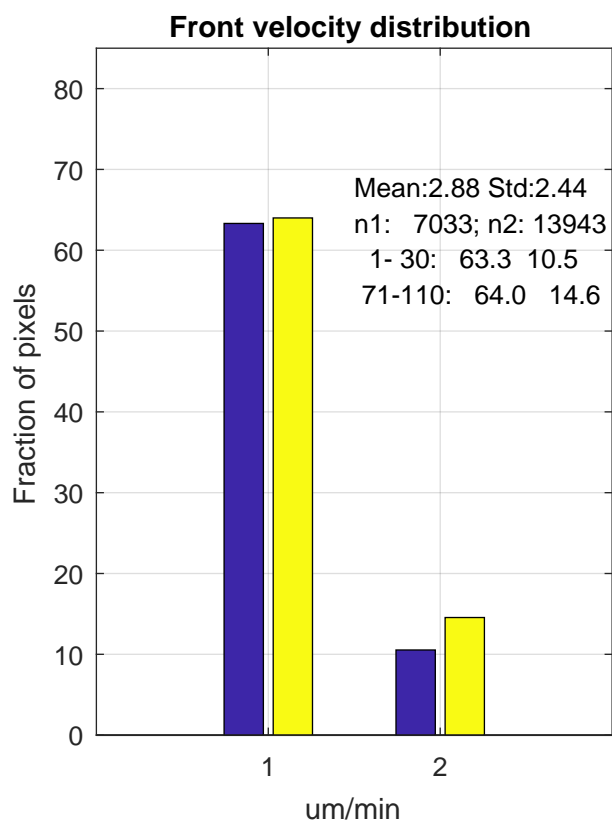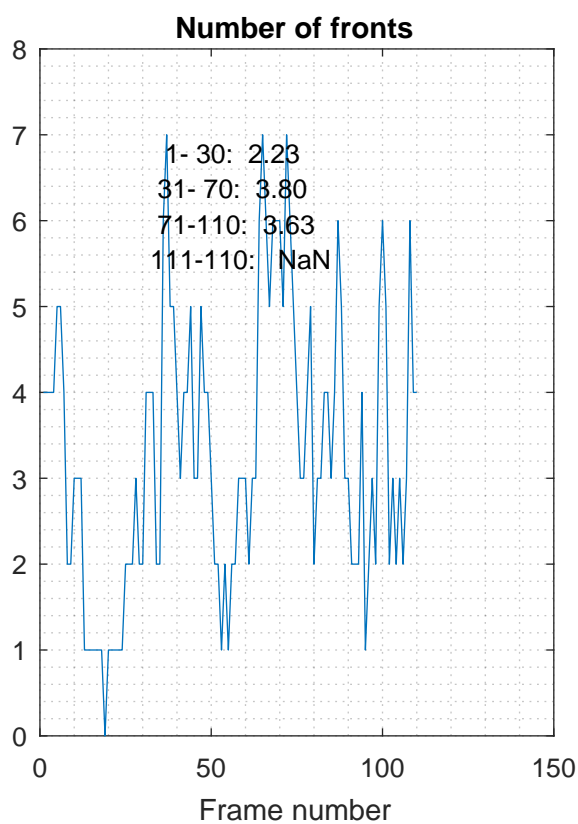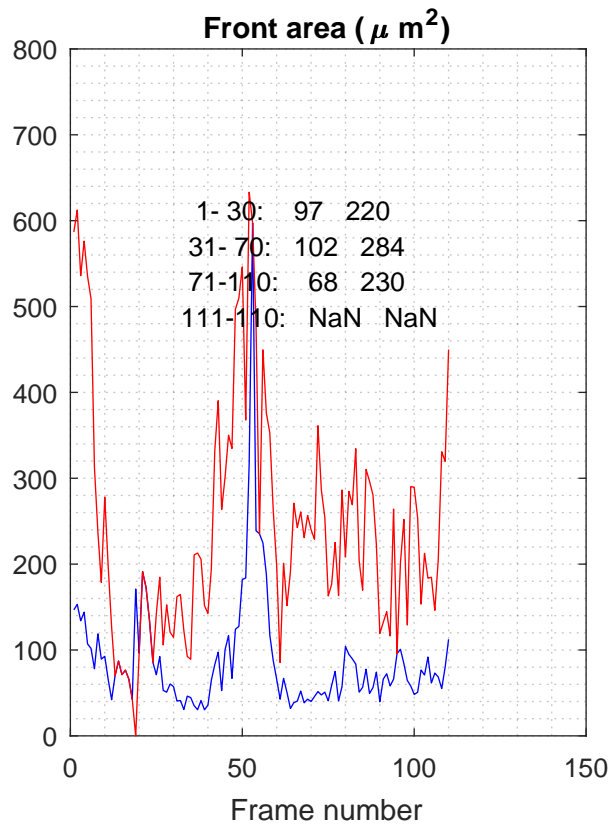

Supplement: Supplementary file 23 — Source Data for Figure 5 [file MSB-15-e8585-s021.zip › Source_data_for_Figure_5/Fig_5C/PKBA17.pdf]

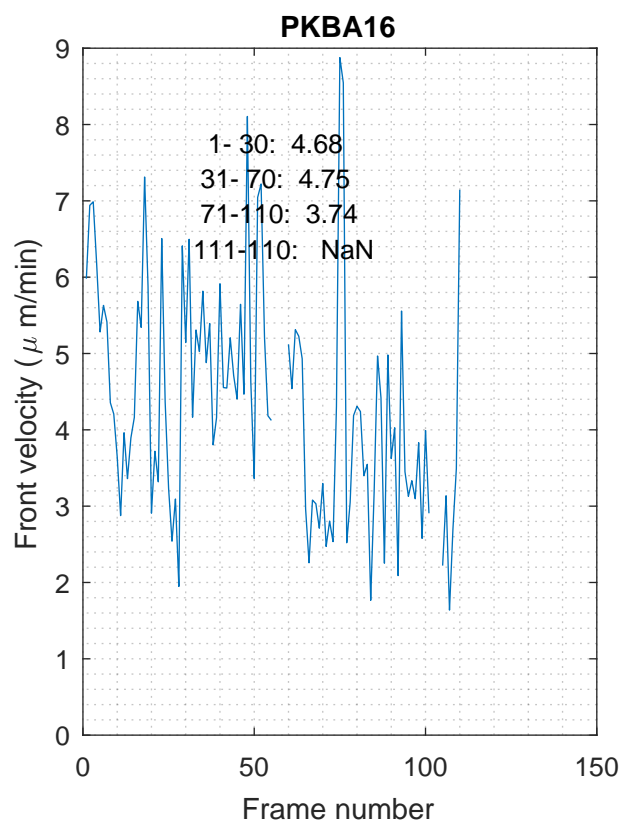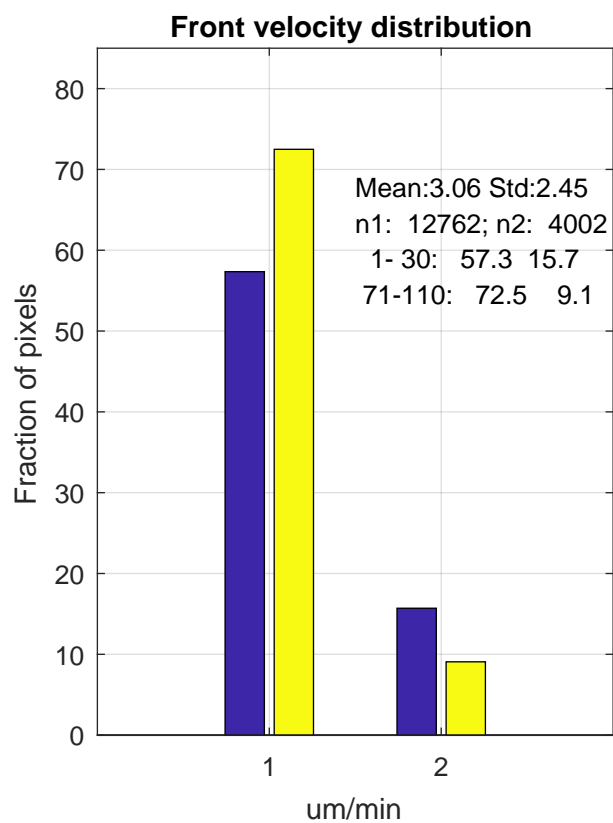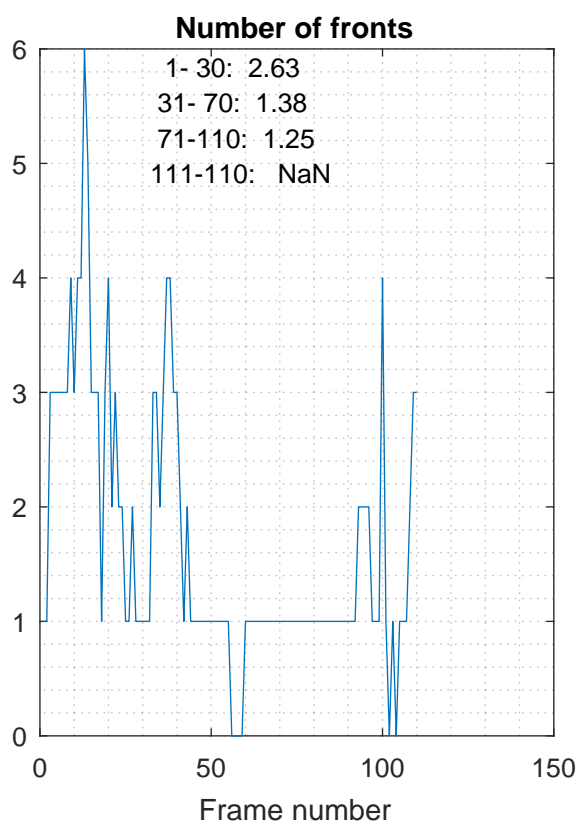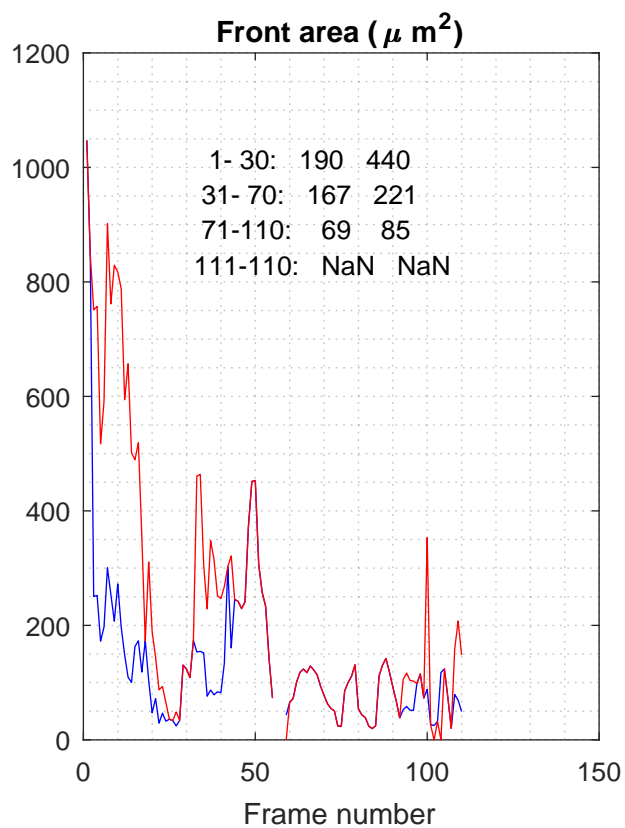

Supplement: Supplementary file 23 — Source Data for Figure 5 [file MSB-15-e8585-s021.zip › Source_data_for_Figure_5/Fig_5C/PKBA16.pdf]

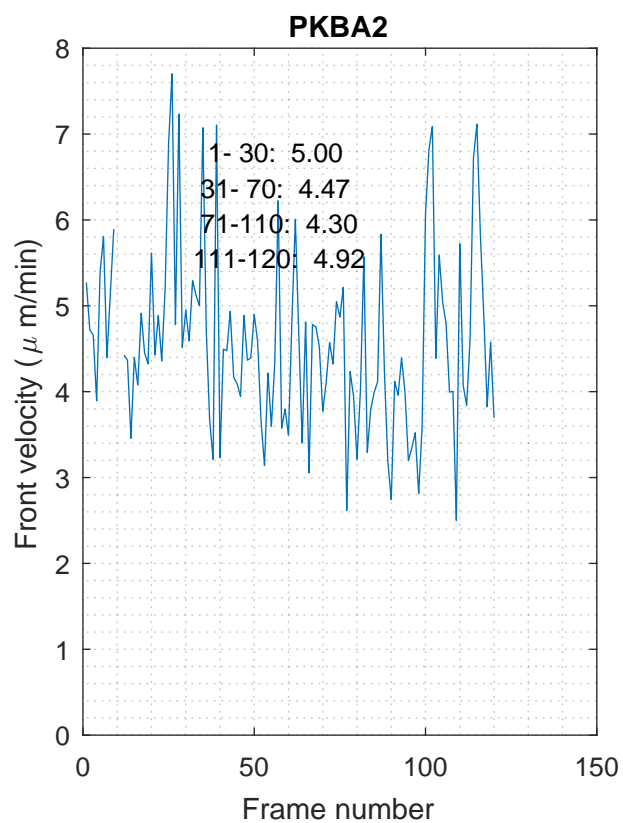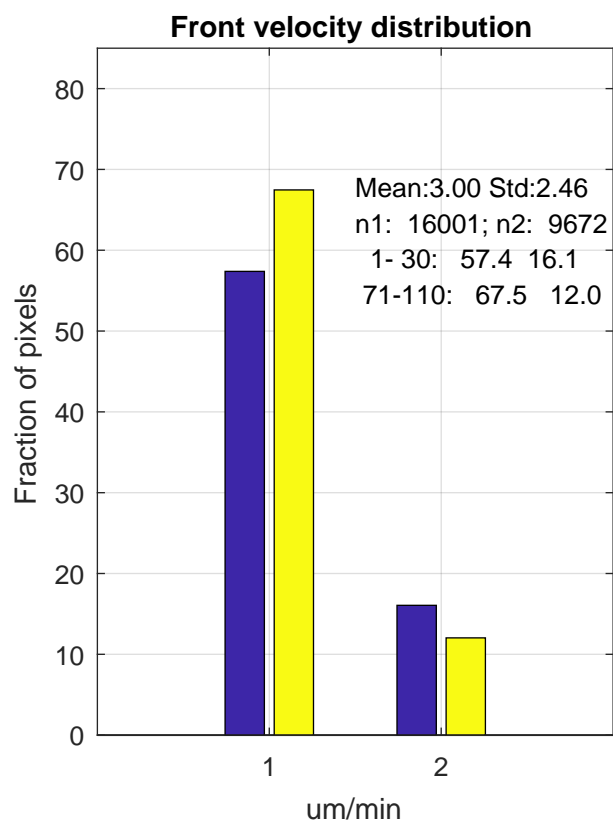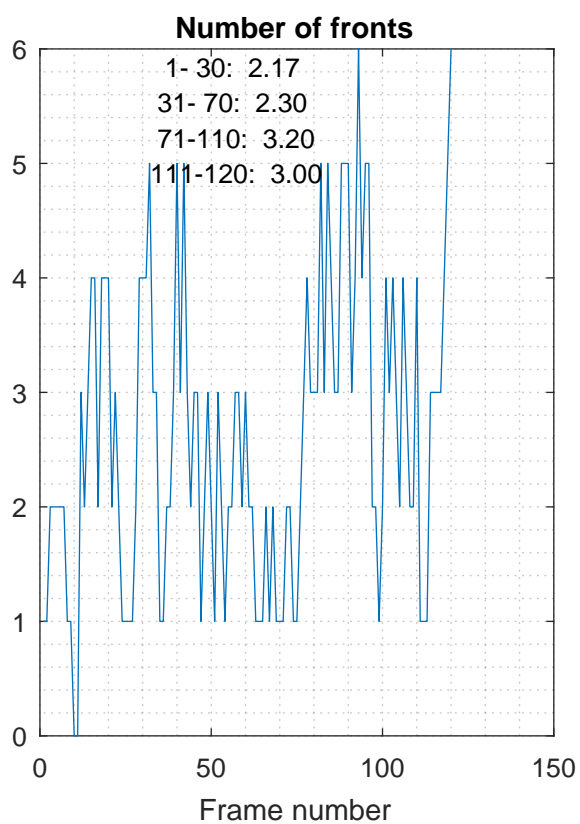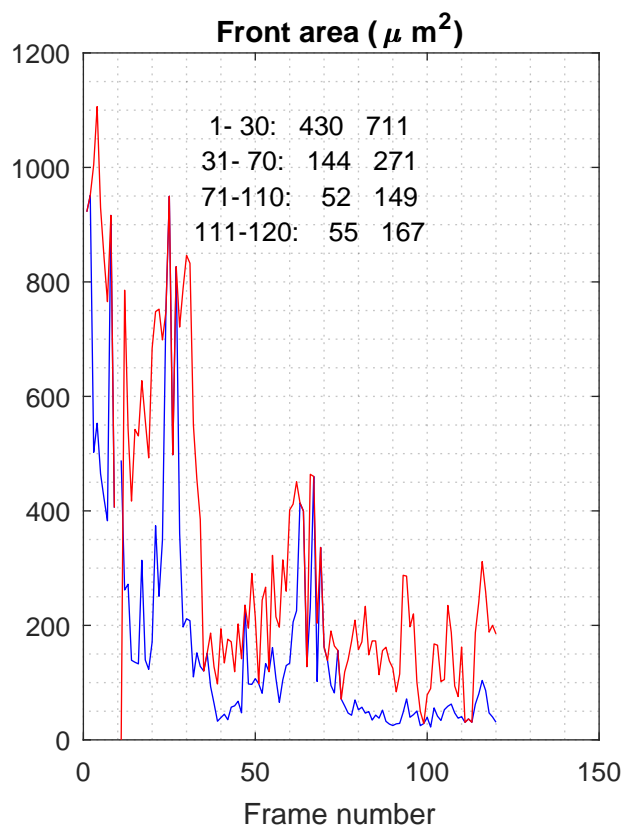

Supplement: Supplementary file 23 — Source Data for Figure 5 [file MSB-15-e8585-s021.zip › Source_data_for_Figure_5/Fig_5C/PKBA2.pdf]

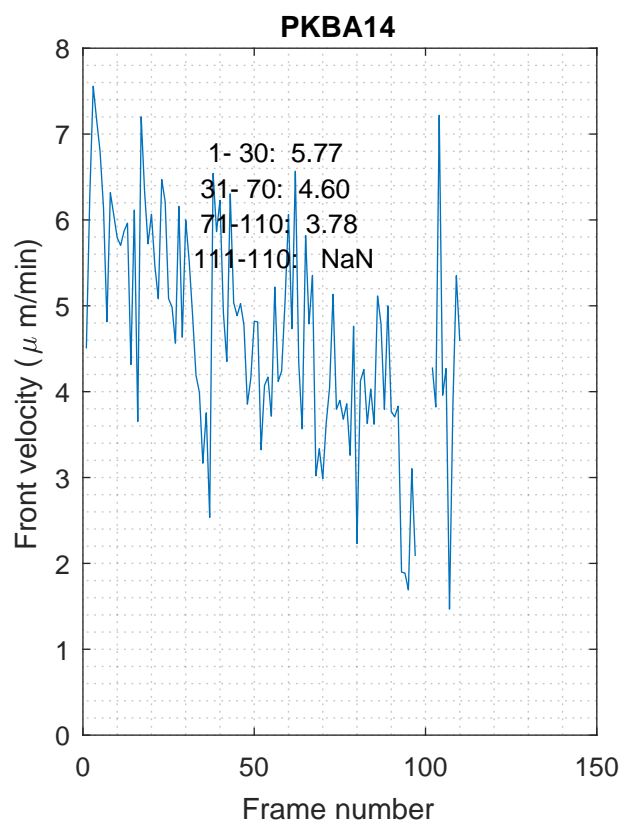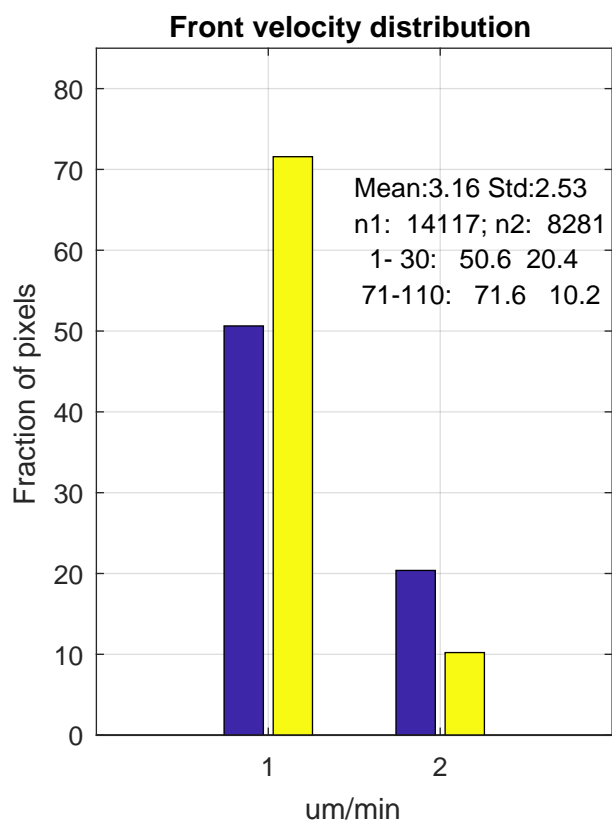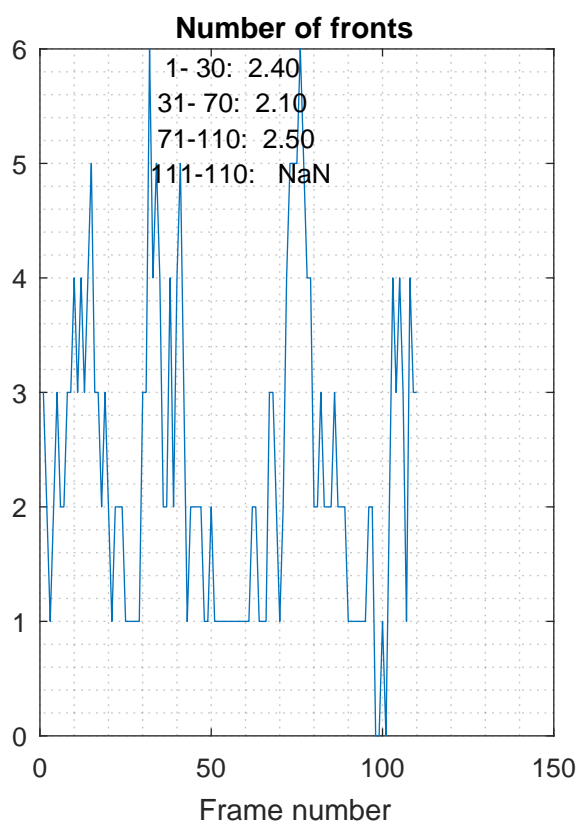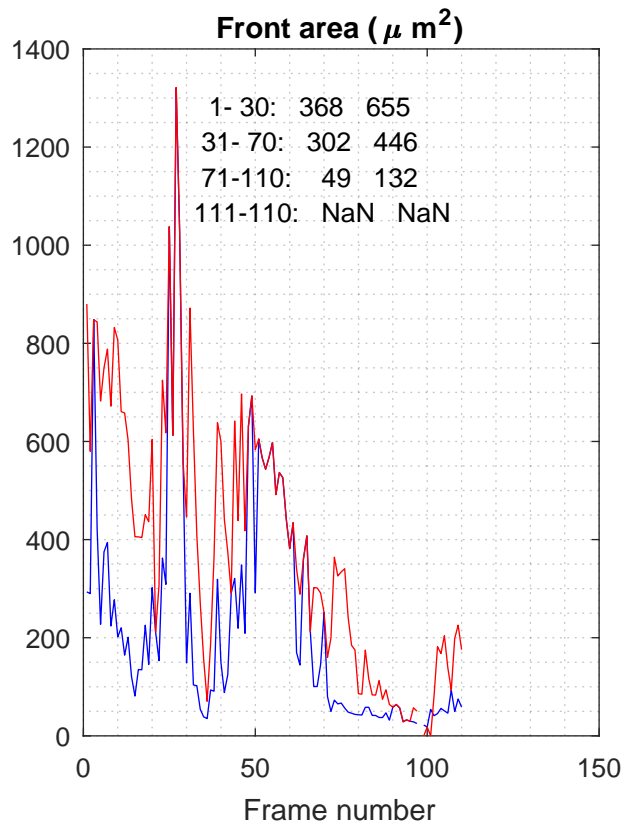

Supplement: Supplementary file 23 — Source Data for Figure 5 [file MSB-15-e8585-s021.zip › Source_data_for_Figure_5/Fig_5C/PKBA14.pdf]

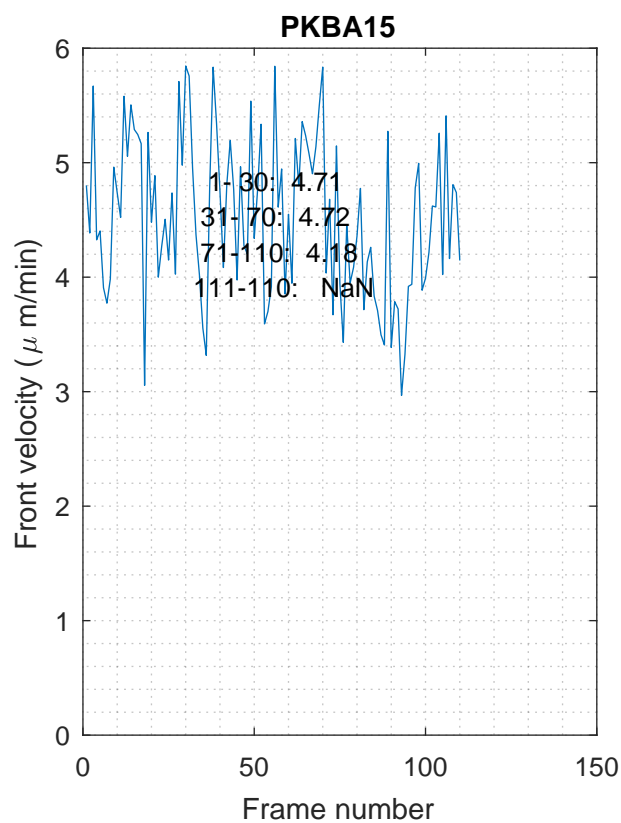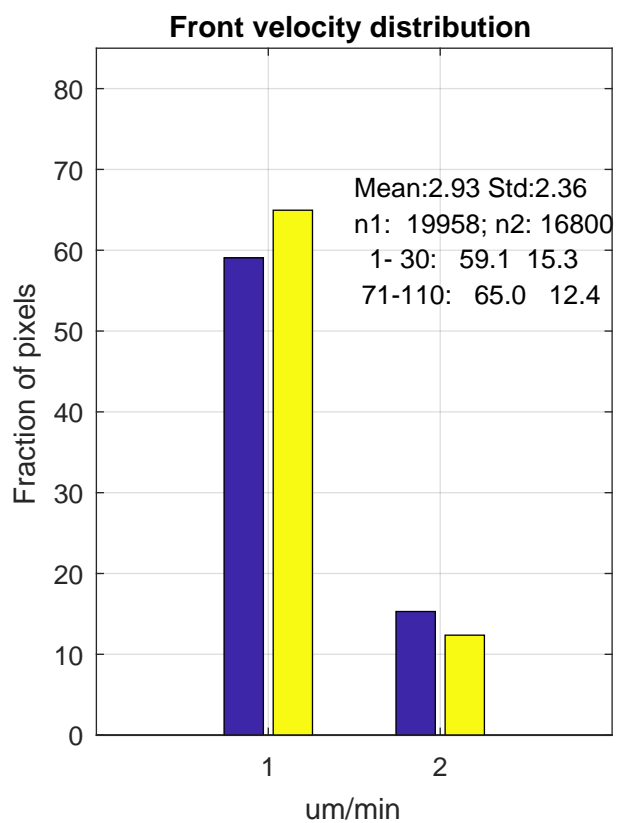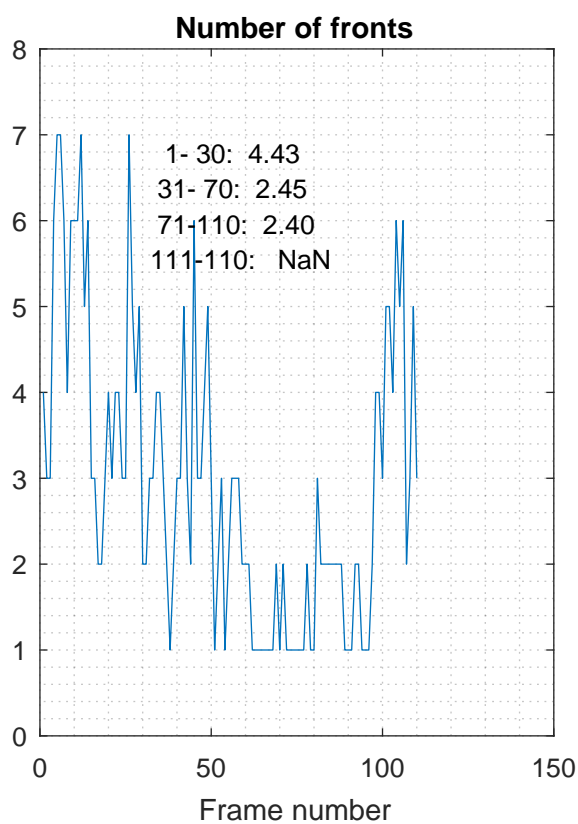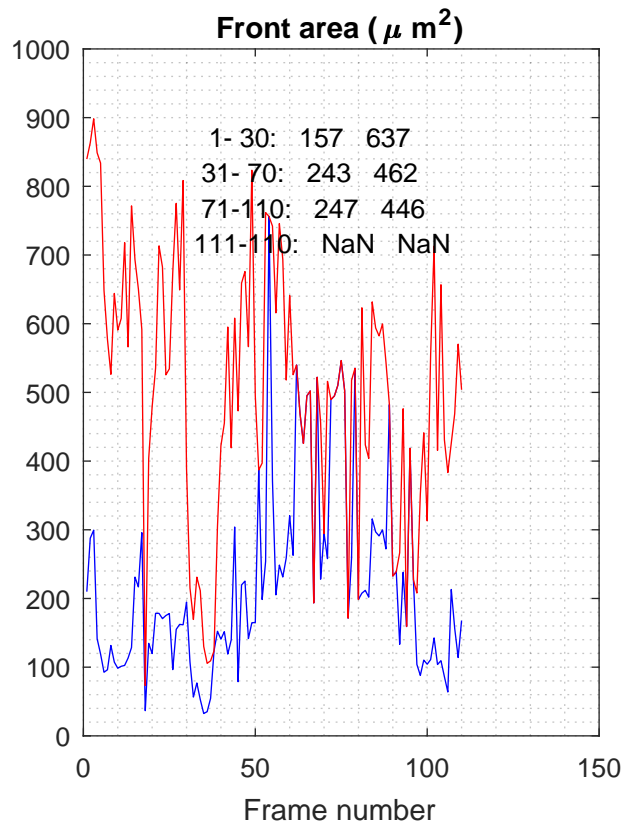

Supplement: Supplementary file 23 — Source Data for Figure 5 [file MSB-15-e8585-s021.zip › Source_data_for_Figure_5/Fig_5C/PKBA15.pdf]

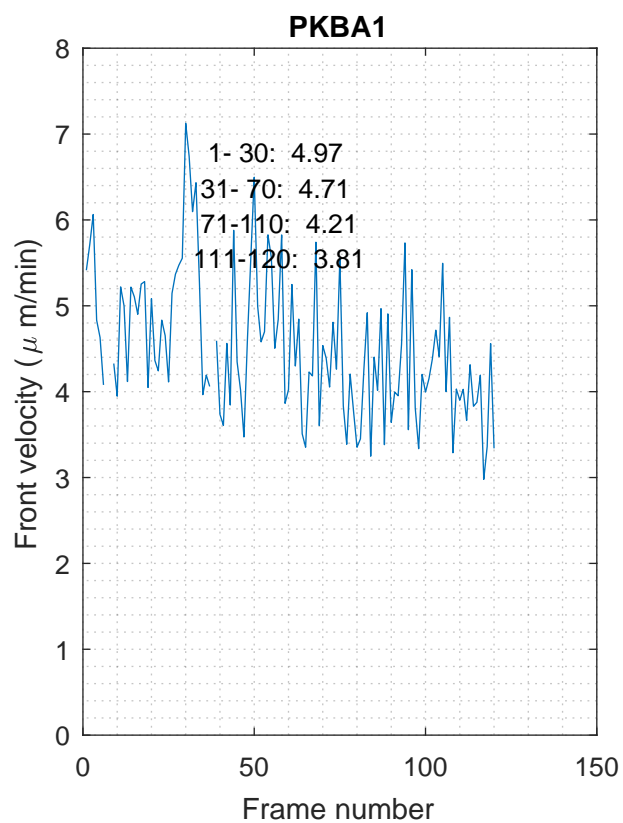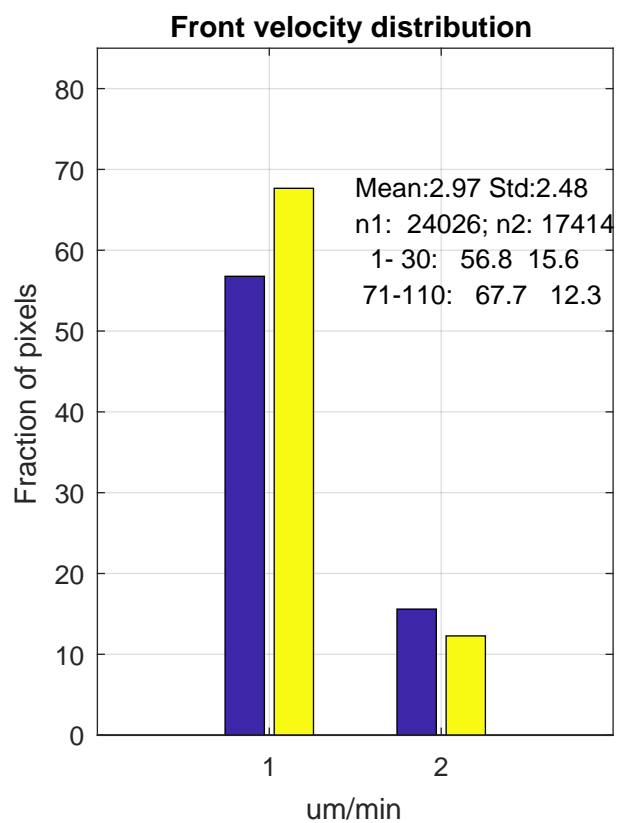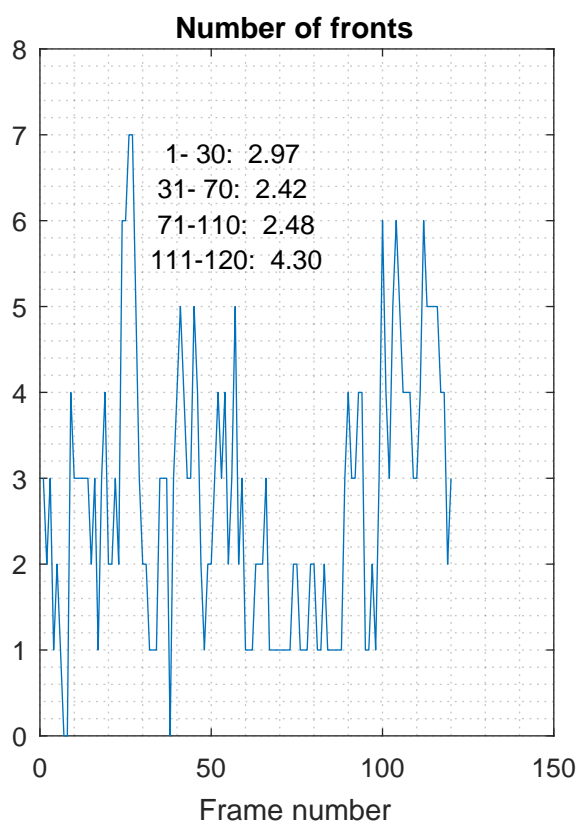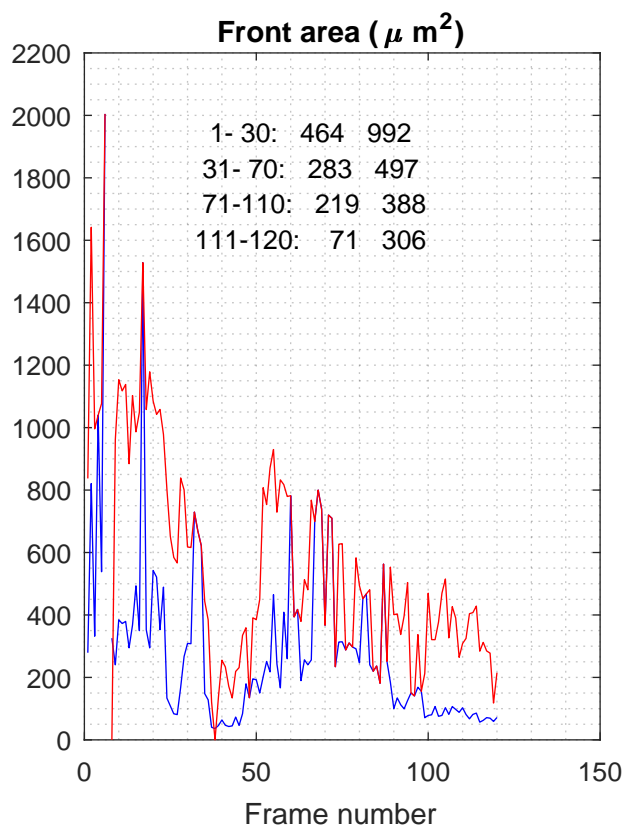

Supplement: Supplementary file 23 — Source Data for Figure 5 [file MSB-15-e8585-s021.zip › Source_data_for_Figure_5/Fig_5C/PKBA1.pdf]

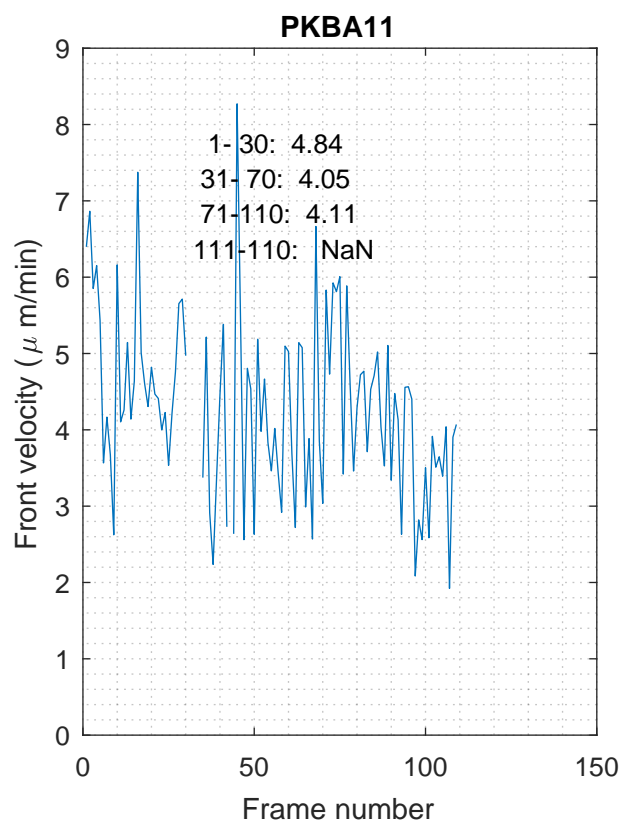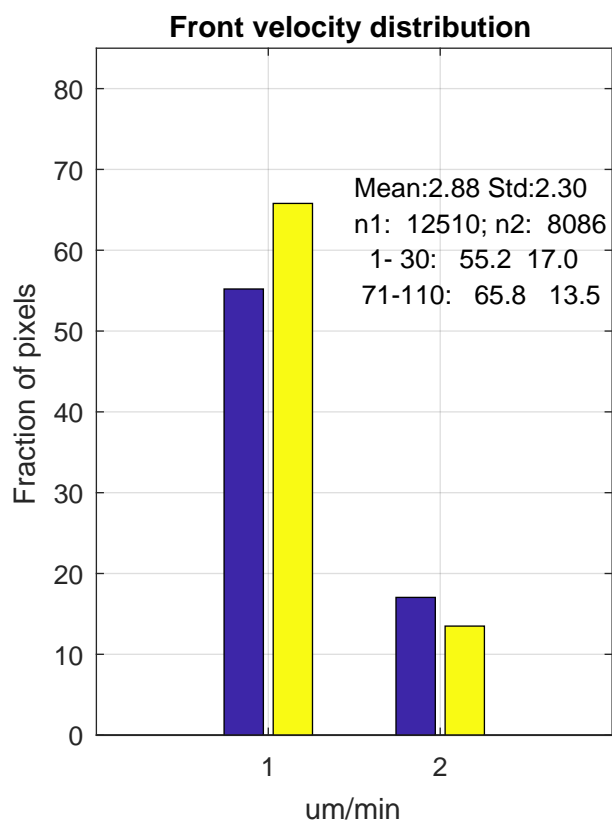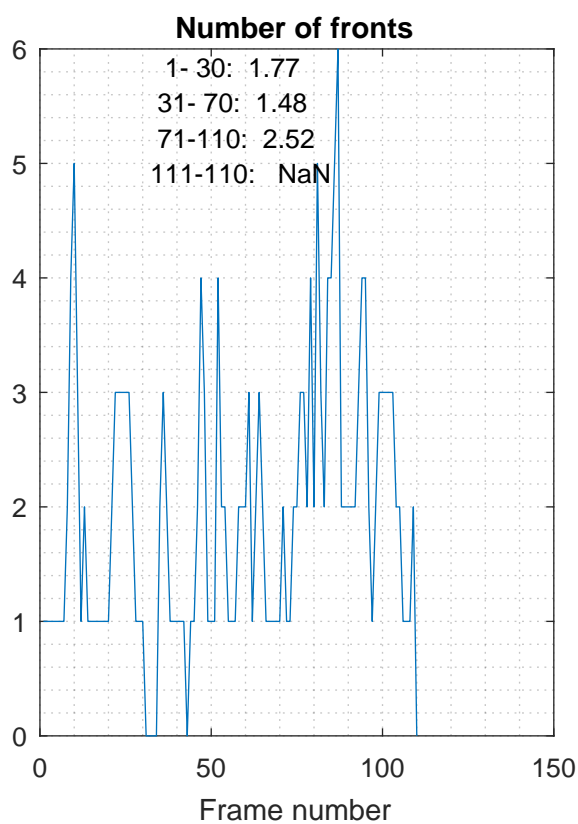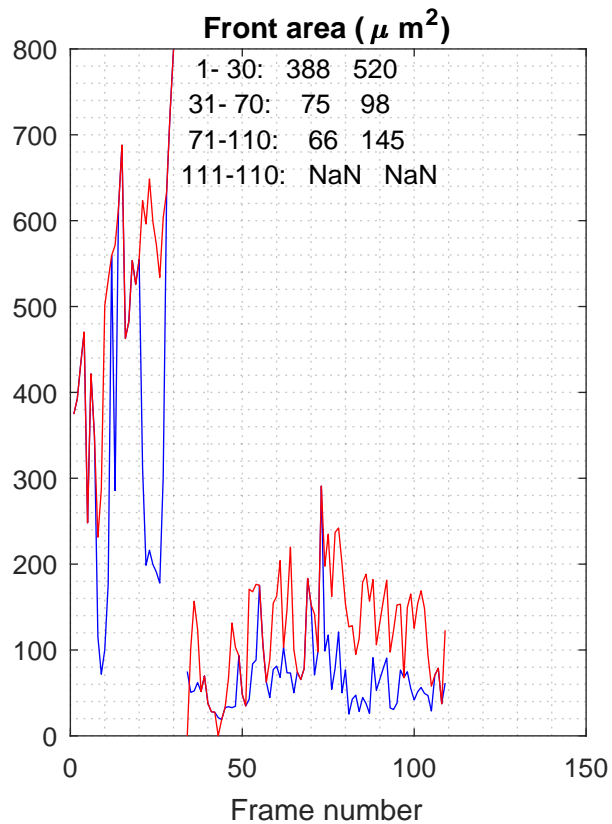

Supplement: Supplementary file 23 — Source Data for Figure 5 [file MSB-15-e8585-s021.zip › Source_data_for_Figure_5/Fig_5C/PKBA11.pdf]

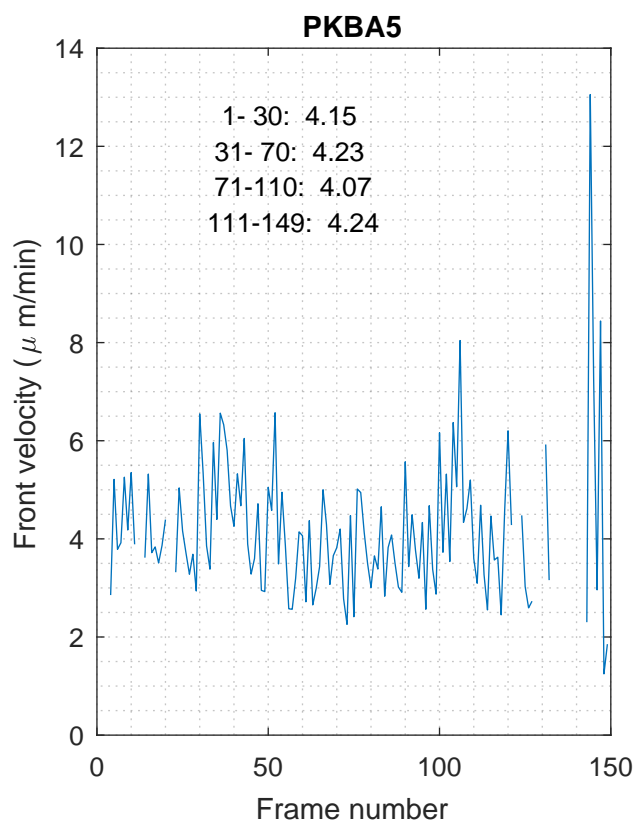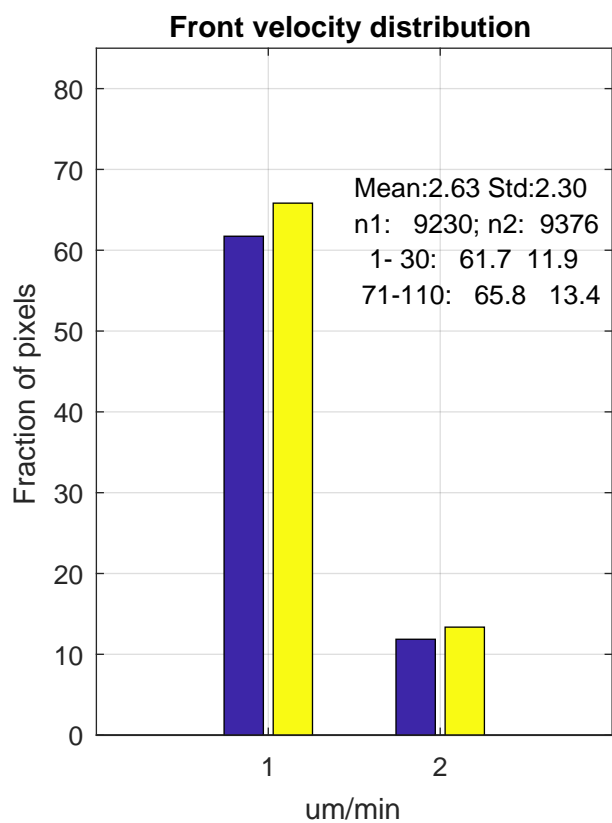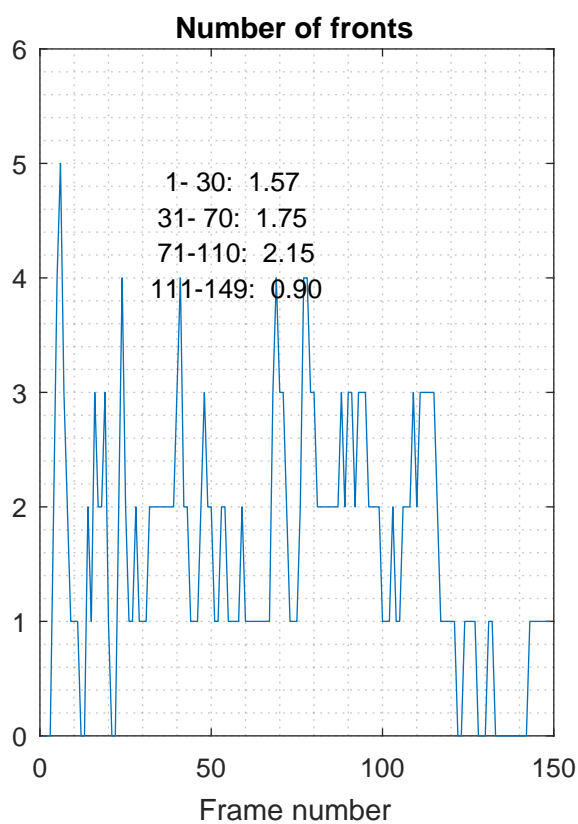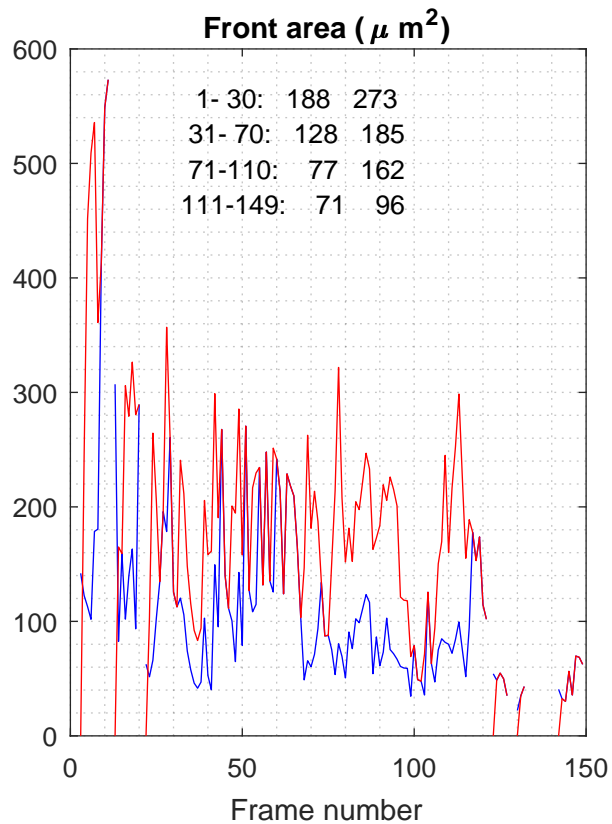

Supplement: Supplementary file 23 — Source Data for Figure 5 [file MSB-15-e8585-s021.zip › Source_data_for_Figure_5/Fig_5C/PKBA5.pdf]

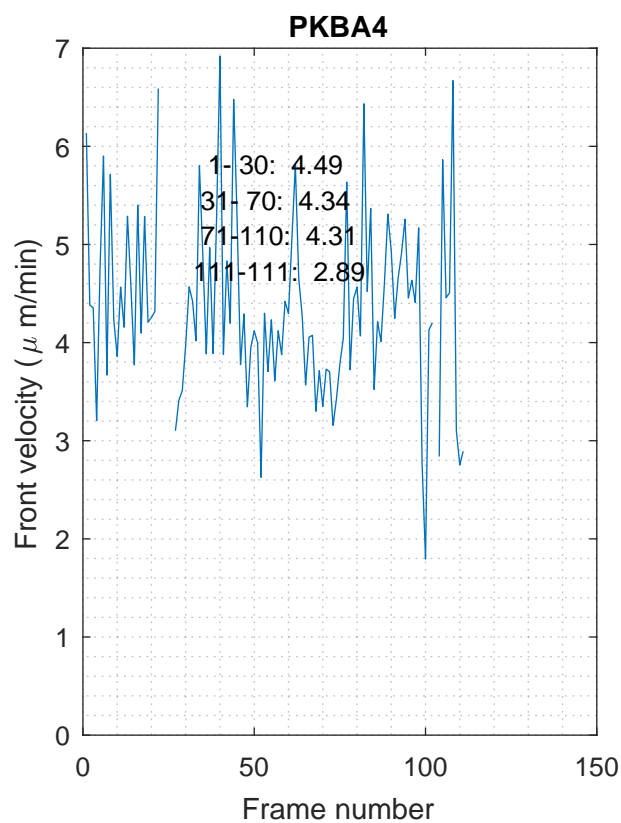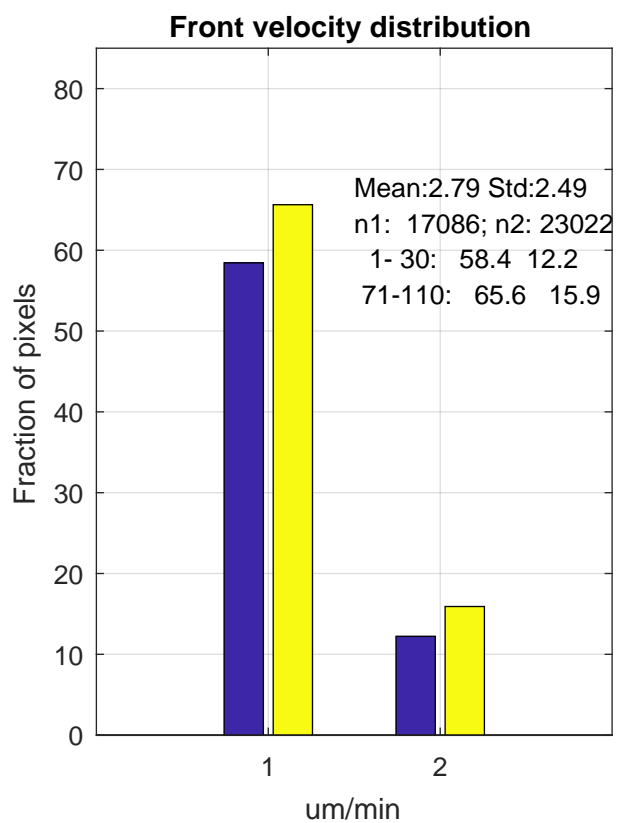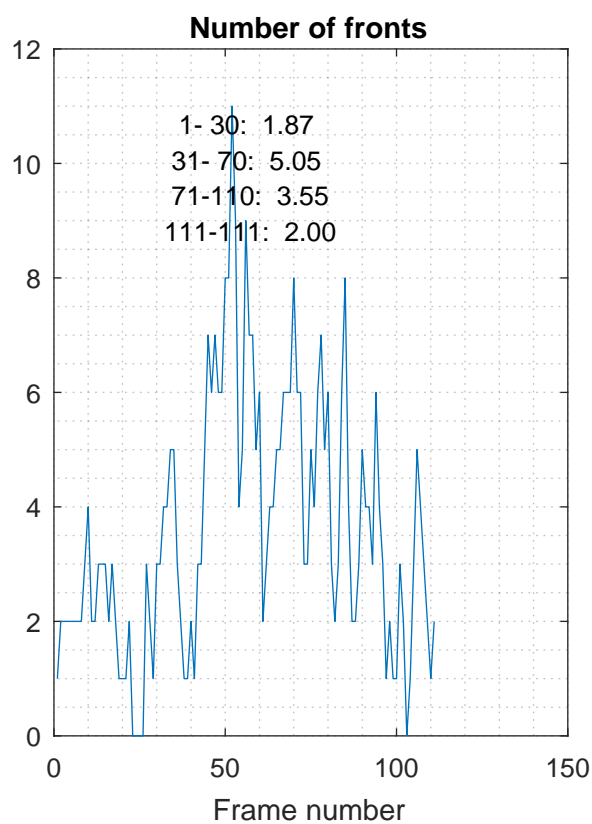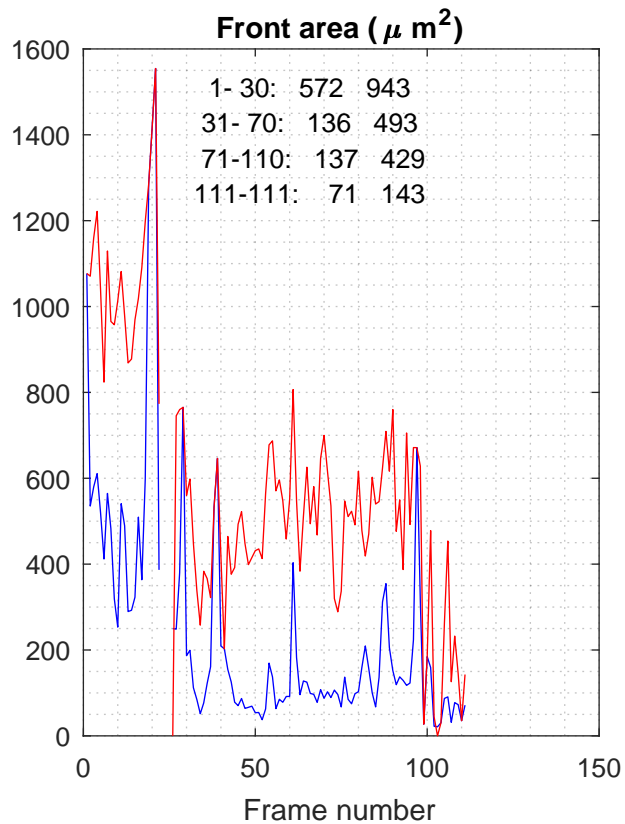

Supplement: Supplementary file 23 — Source Data for Figure 5 [file MSB-15-e8585-s021.zip › Source_data_for_Figure_5/Fig_5C/PKBA4.pdf]

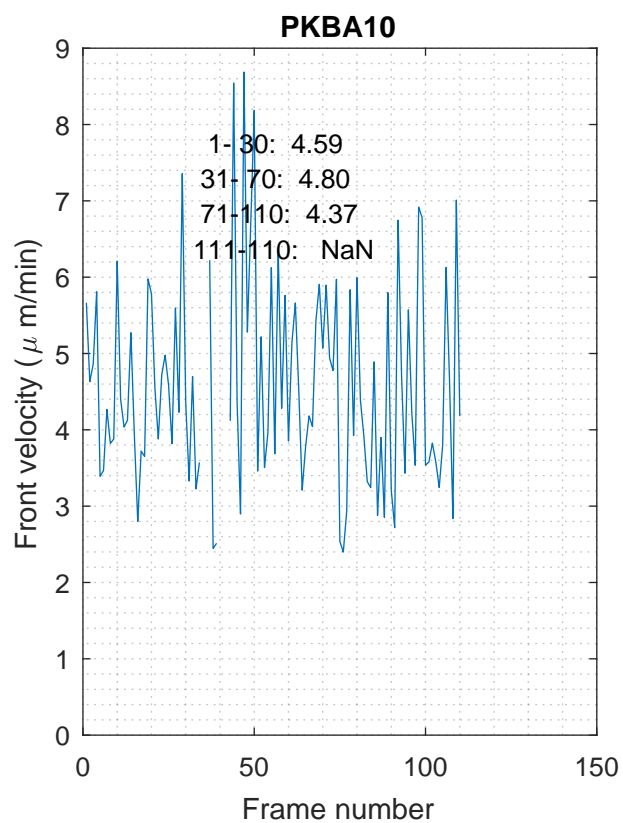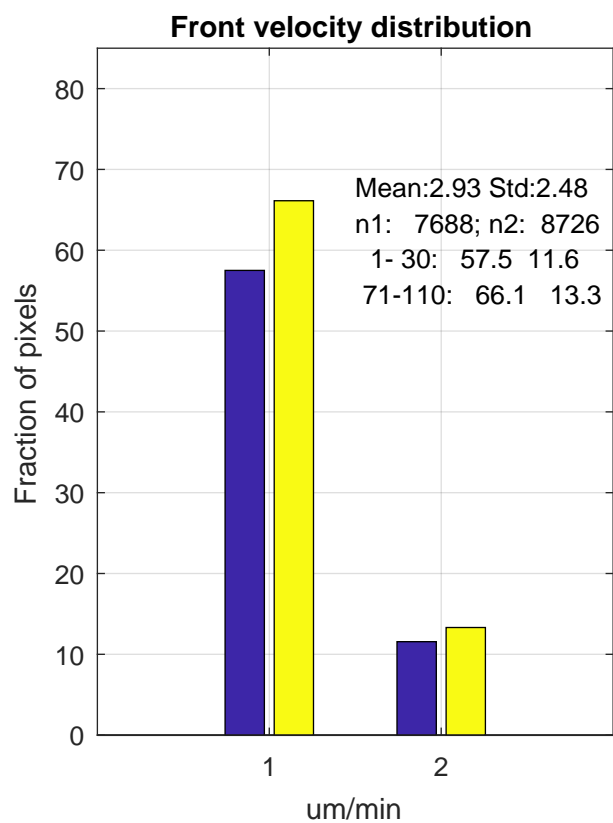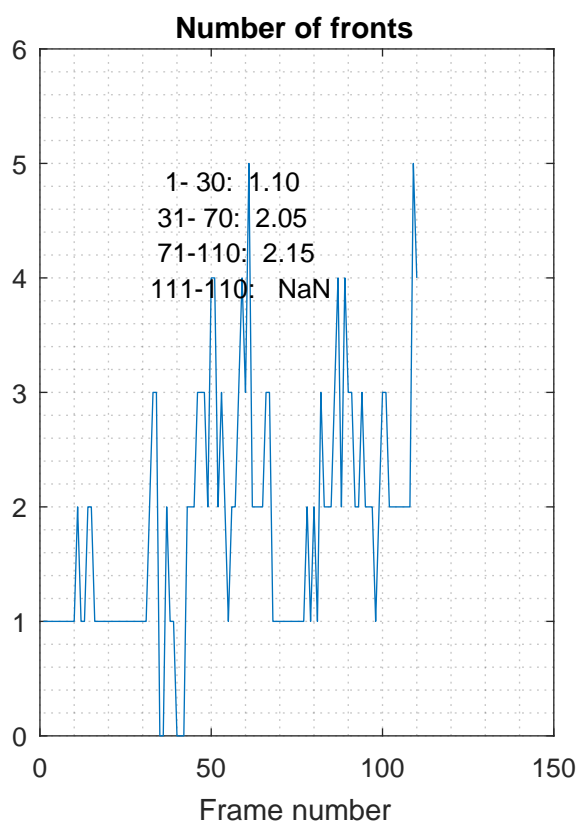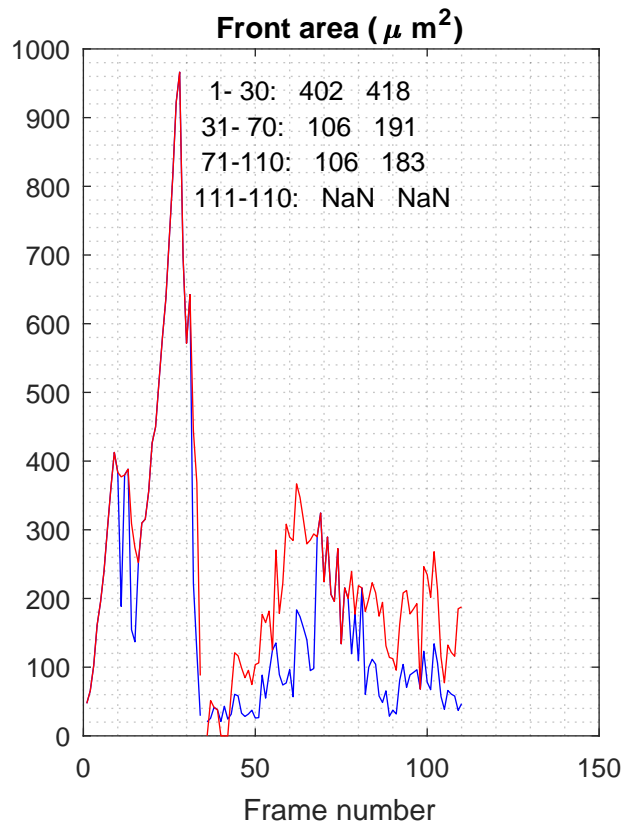

Supplement: Supplementary file 23 — Source Data for Figure 5 [file MSB-15-e8585-s021.zip › Source_data_for_Figure_5/Fig_5C/PKBA10.pdf]

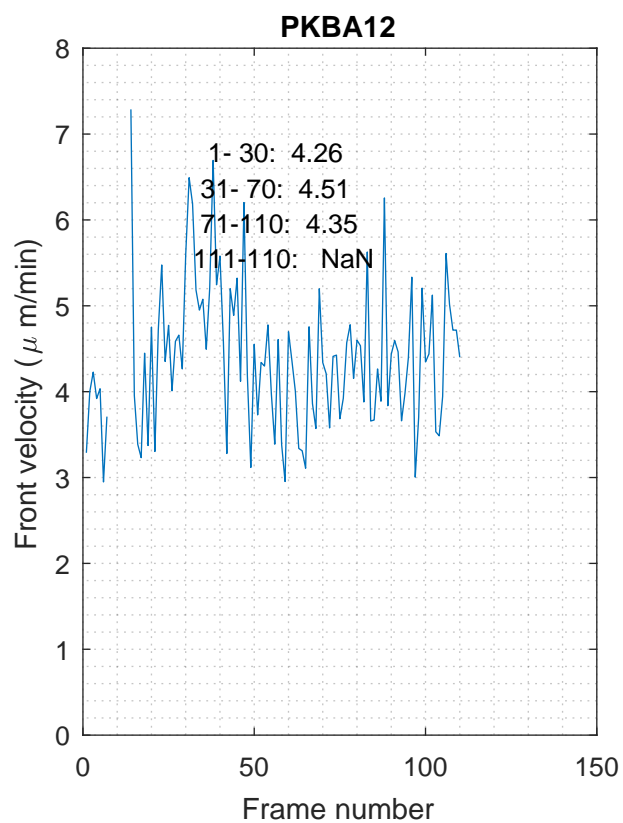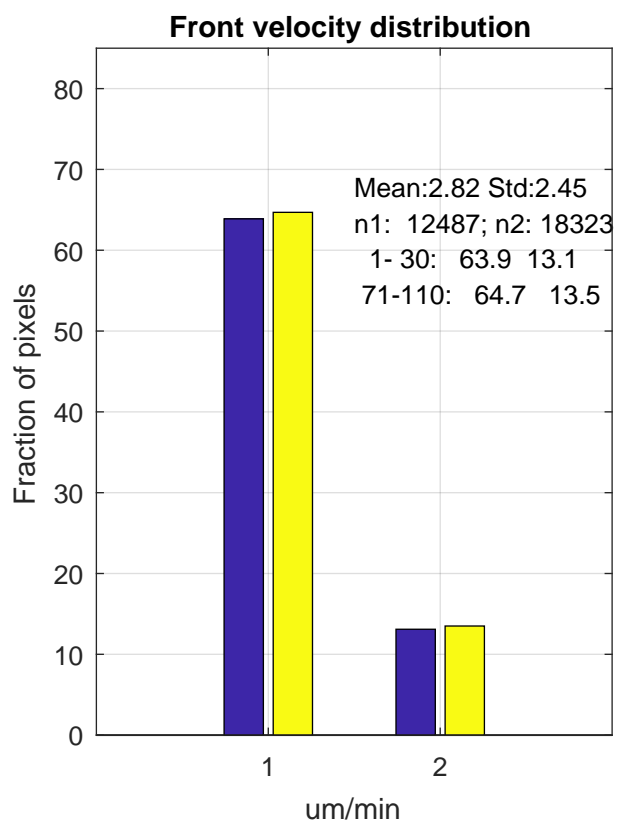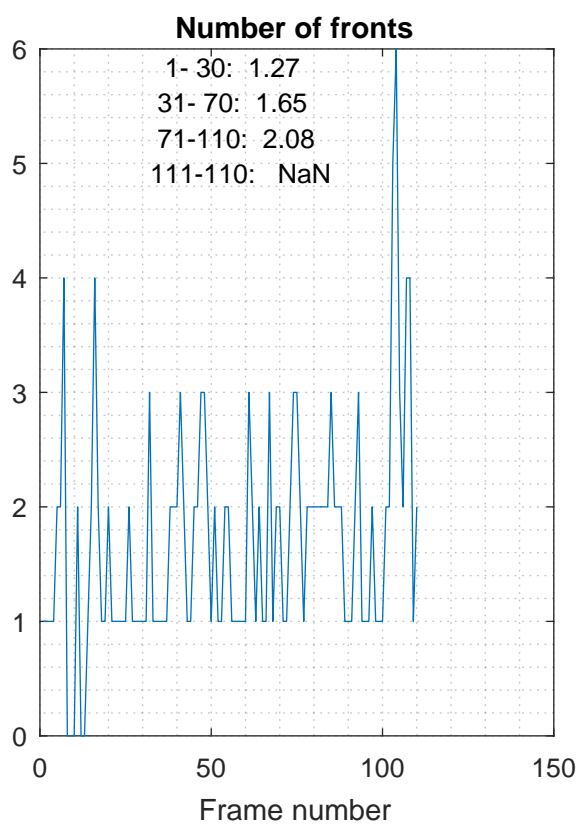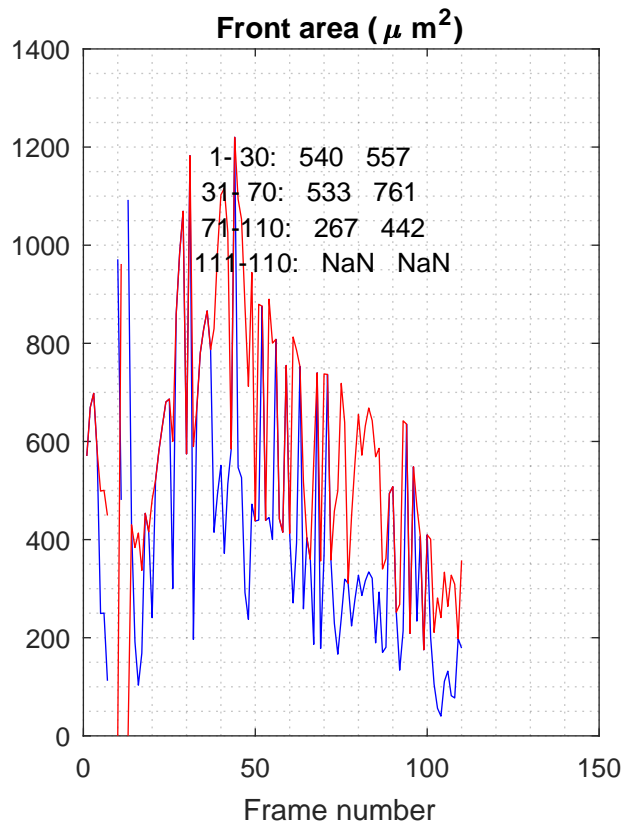

Supplement: Supplementary file 23 — Source Data for Figure 5 [file MSB-15-e8585-s021.zip › Source_data_for_Figure_5/Fig_5C/PKBA12.pdf]

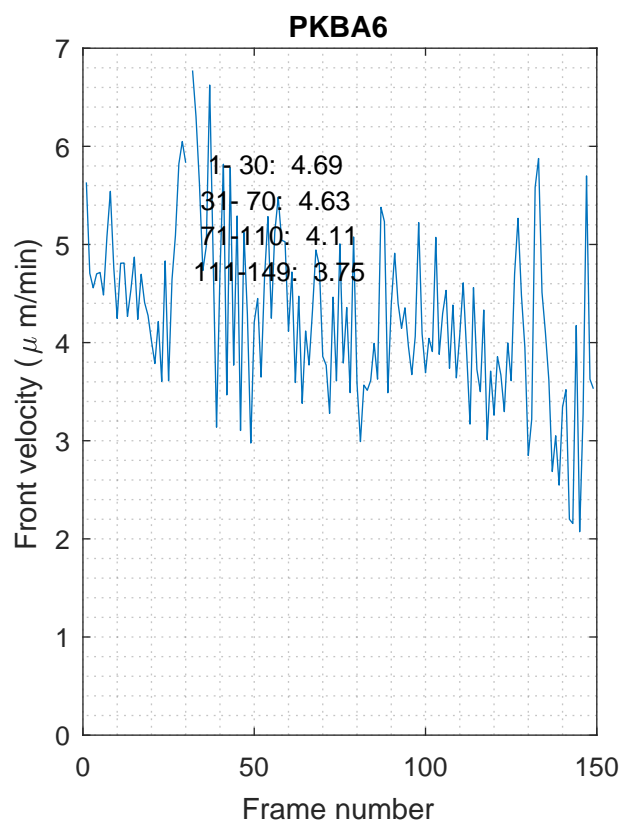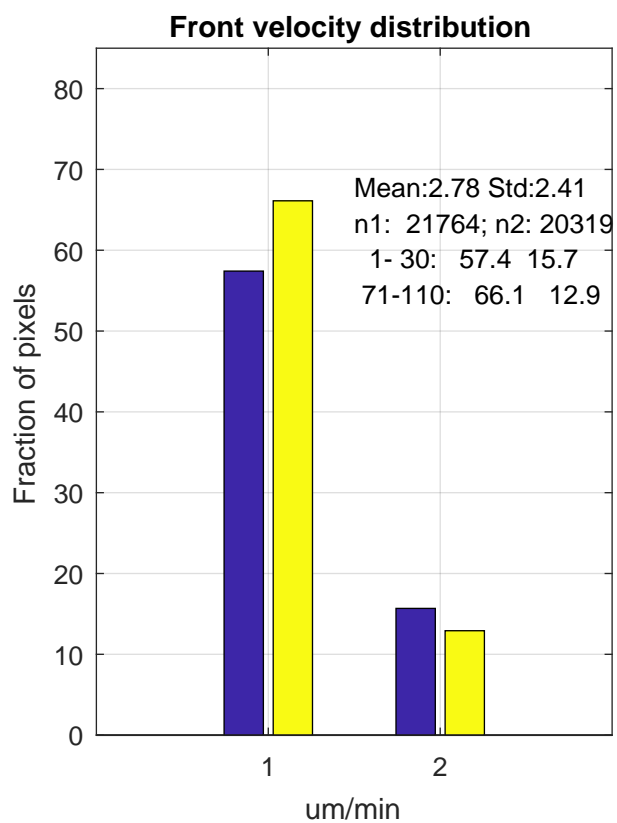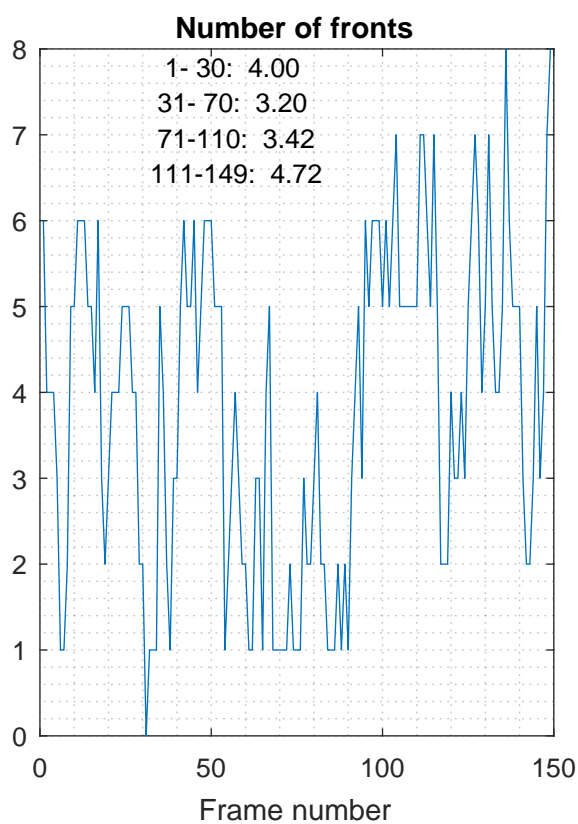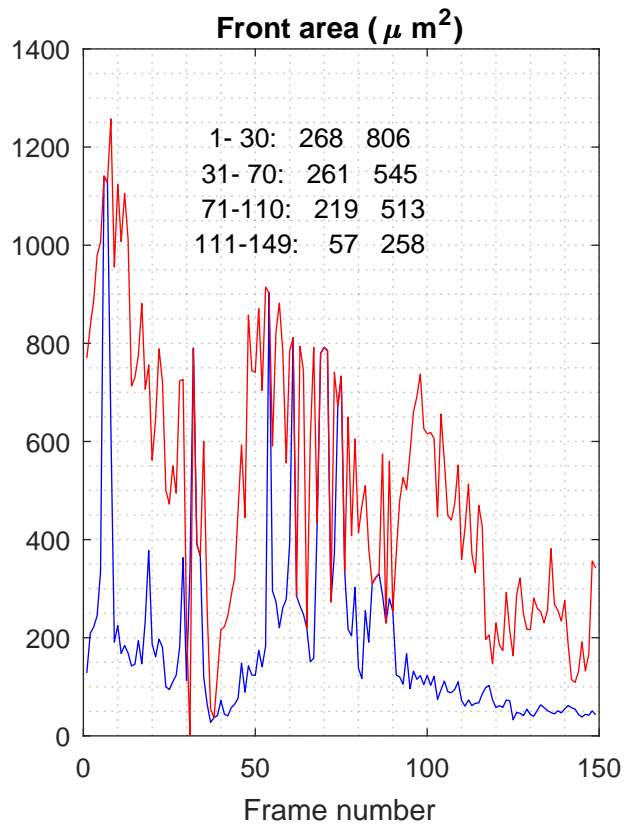

Supplement: Supplementary file 23 — Source Data for Figure 5 [file MSB-15-e8585-s021.zip › Source_data_for_Figure_5/Fig_5C/PKBA6.pdf]

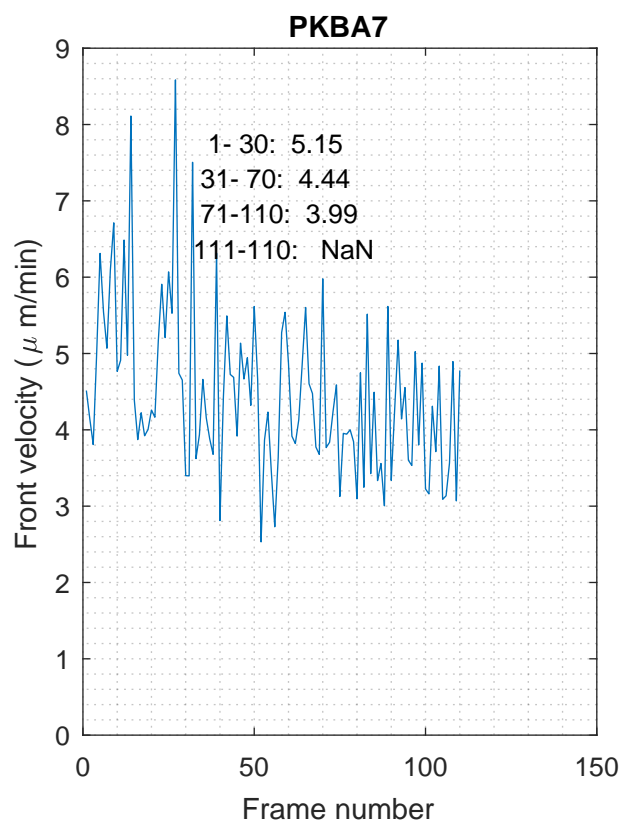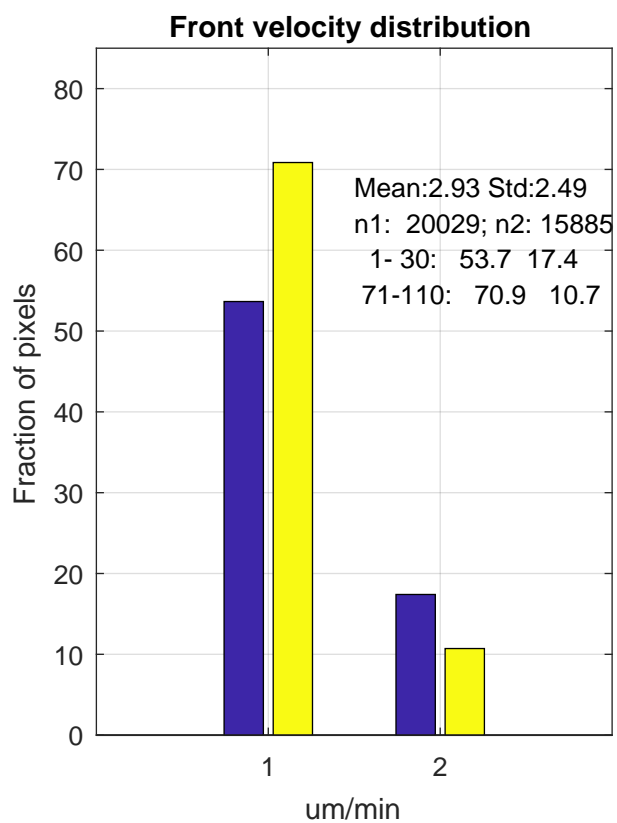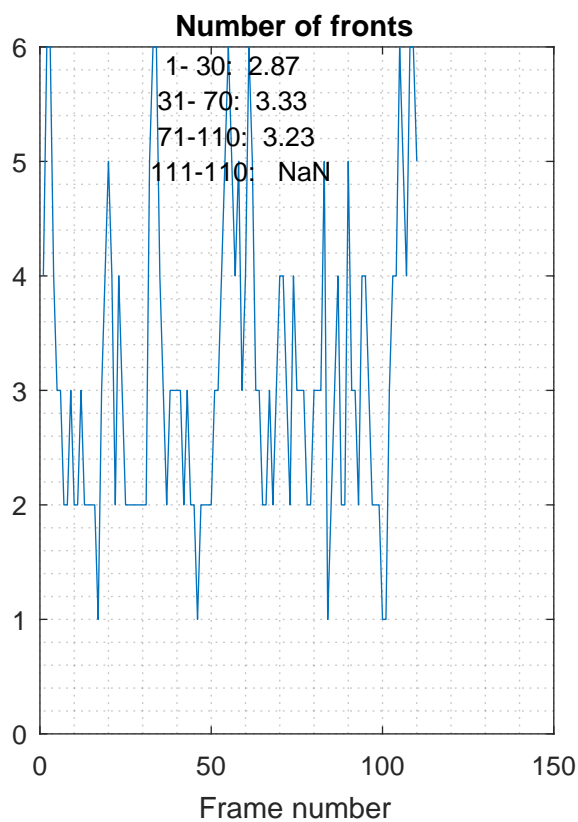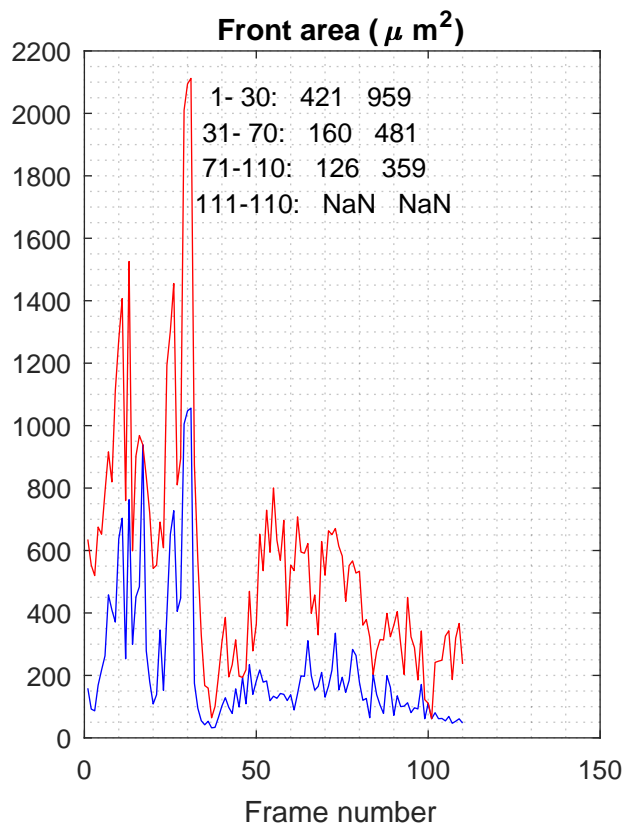

Supplement: Supplementary file 23 — Source Data for Figure 5 [file MSB-15-e8585-s021.zip › Source_data_for_Figure_5/Fig_5C/PKBA7.pdf]

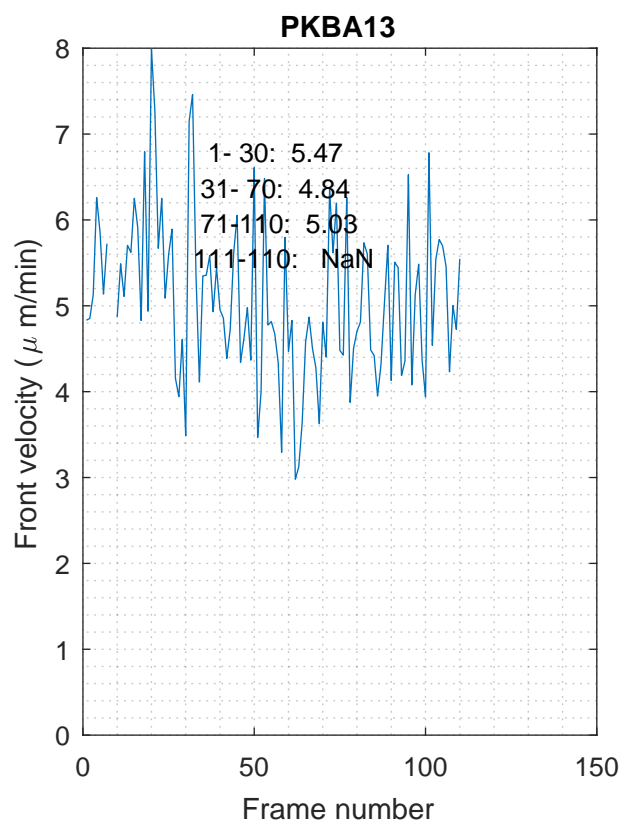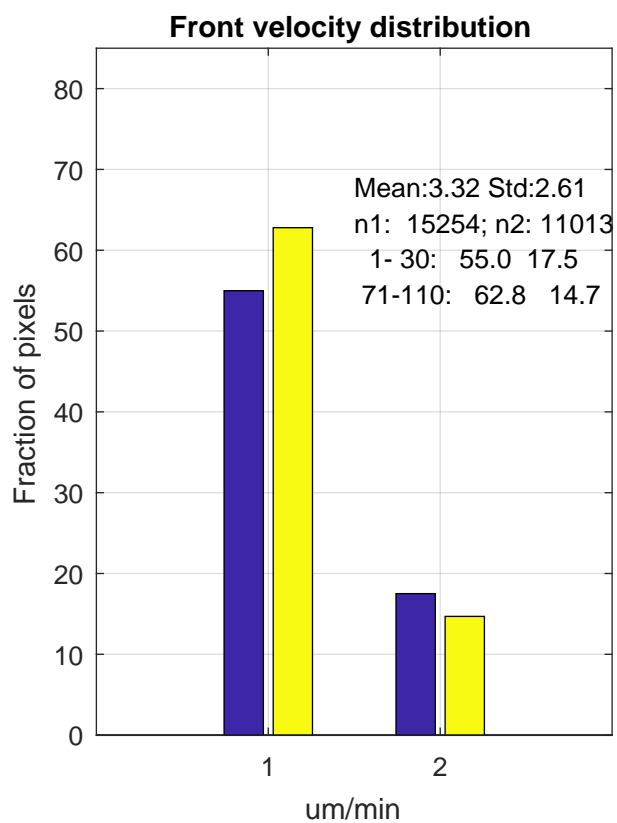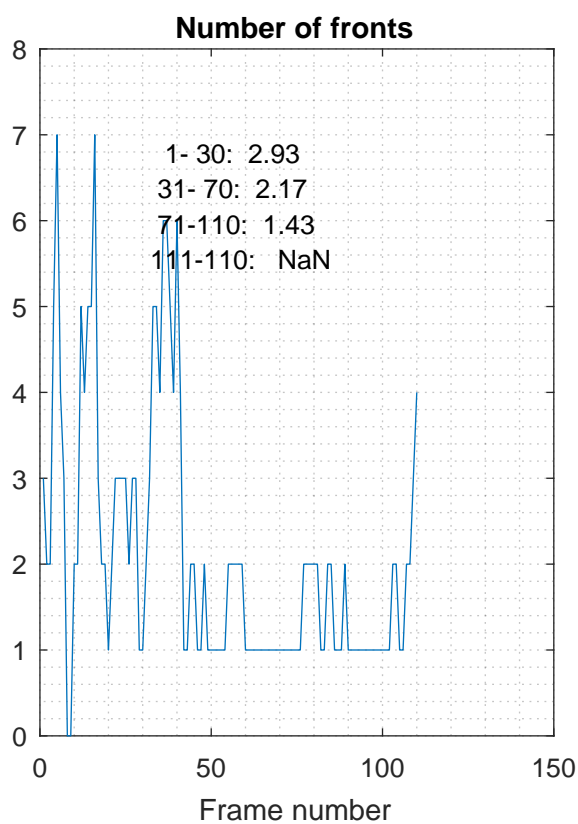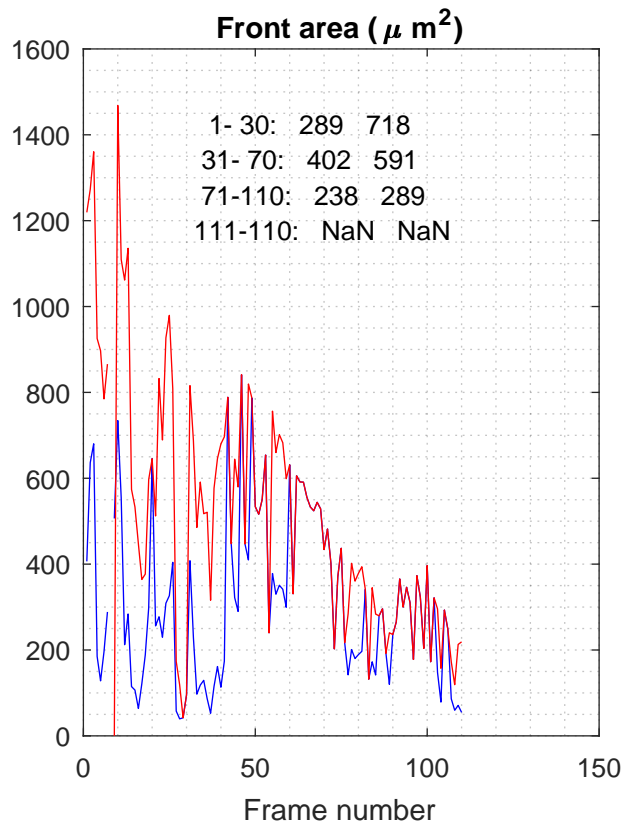

Supplement: Supplementary file 23 — Source Data for Figure 5 [file MSB-15-e8585-s021.zip › Source_data_for_Figure_5/Fig_5C/PKBA13.pdf]

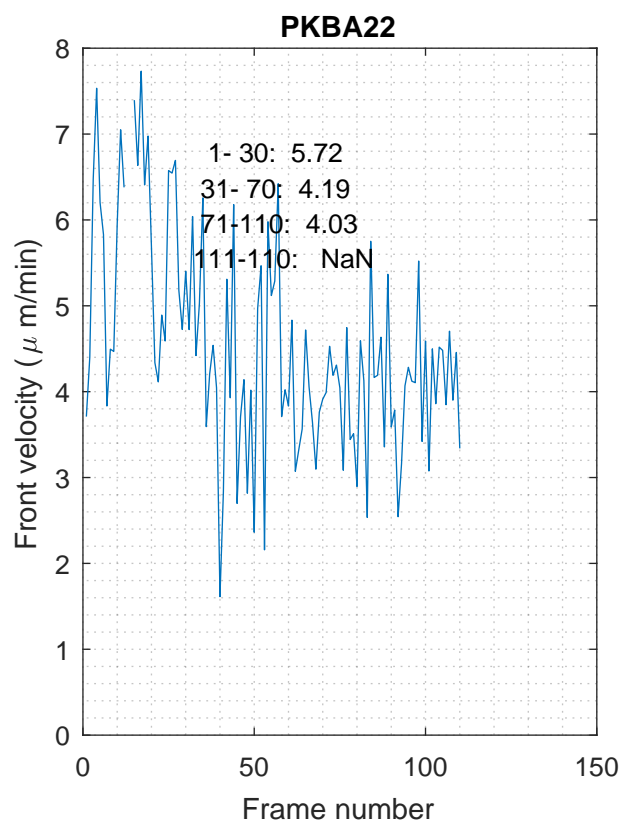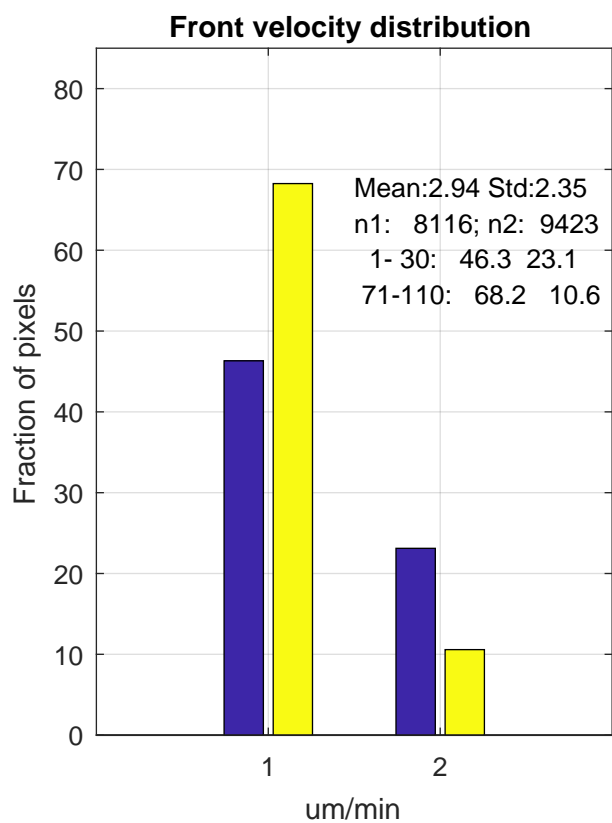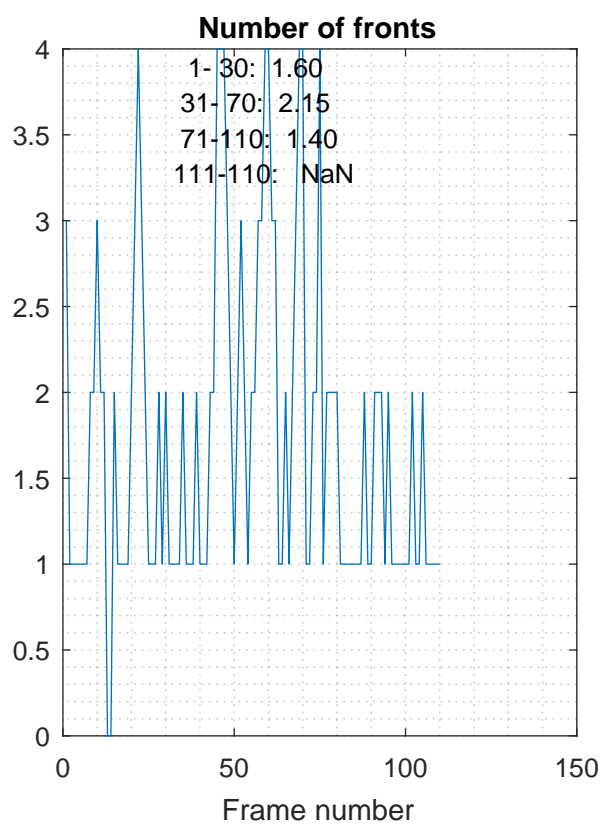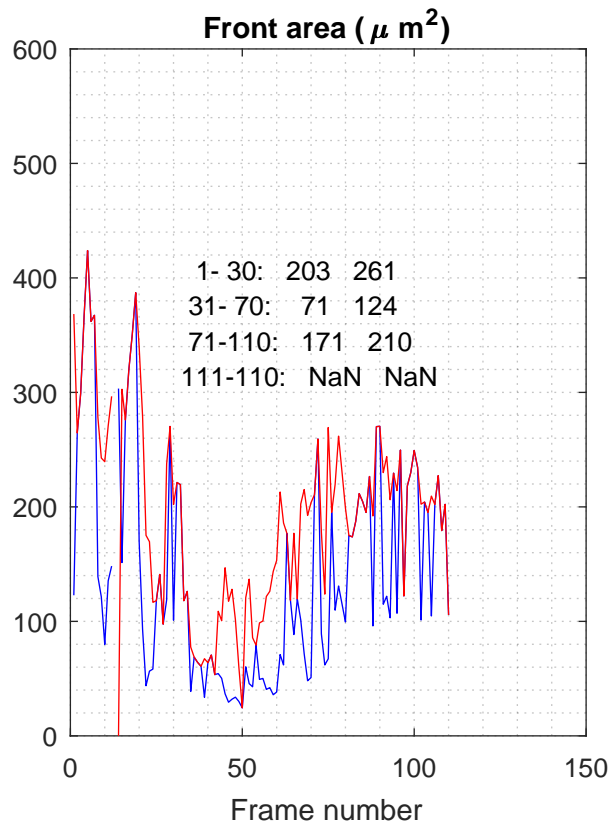

Supplement: Supplementary file 23 — Source Data for Figure 5 [file MSB-15-e8585-s021.zip › Source_data_for_Figure_5/Fig_5C/PKBA22.pdf]

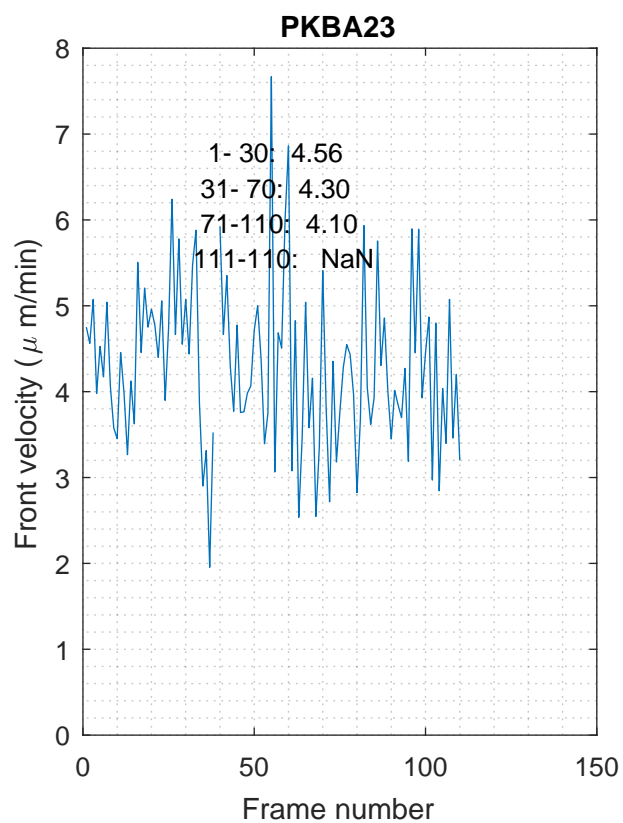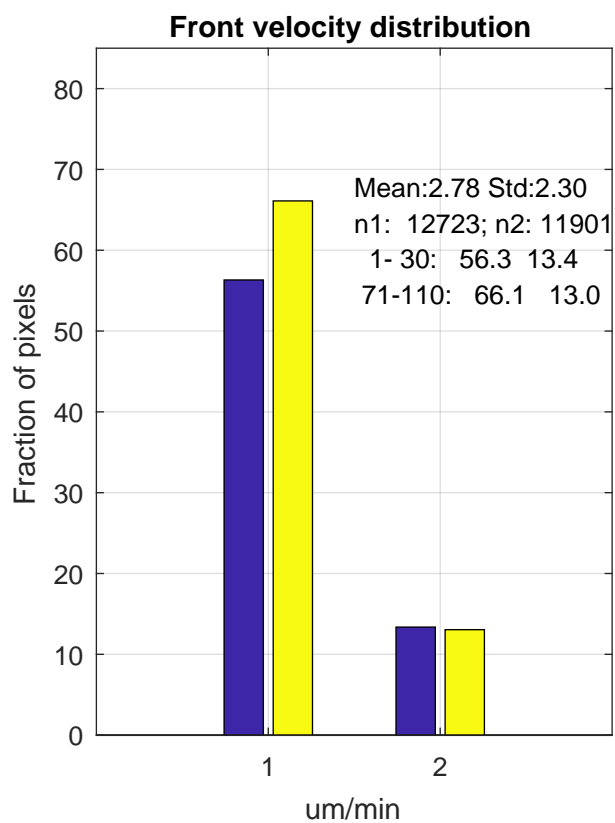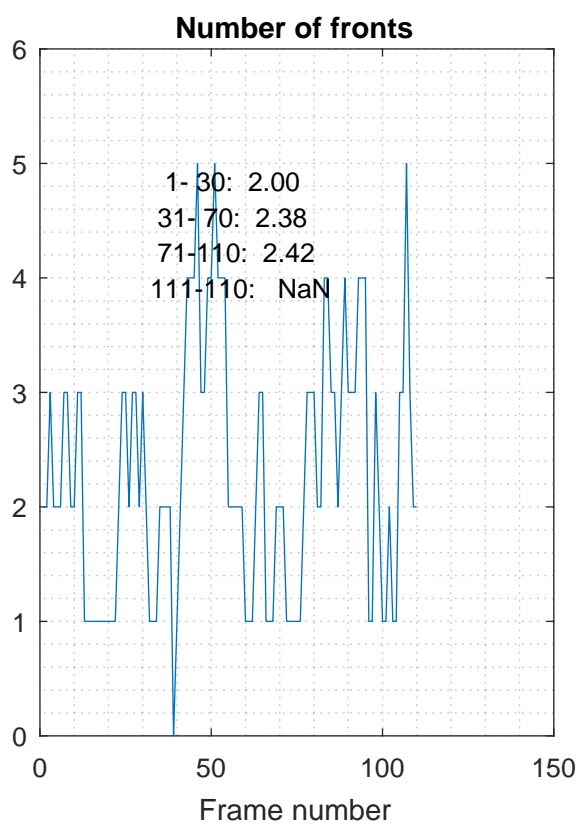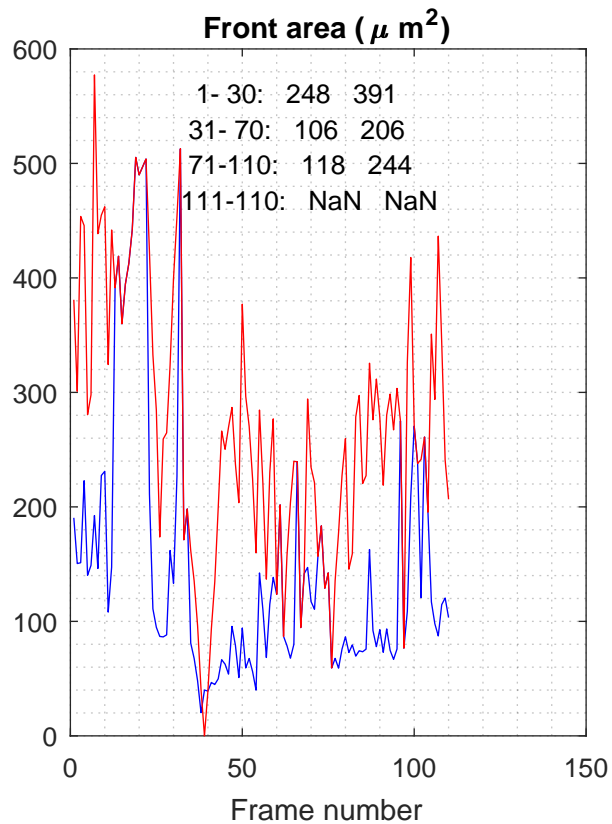

Supplement: Supplementary file 23 — Source Data for Figure 5 [file MSB-15-e8585-s021.zip › Source_data_for_Figure_5/Fig_5C/PKBA23.pdf]

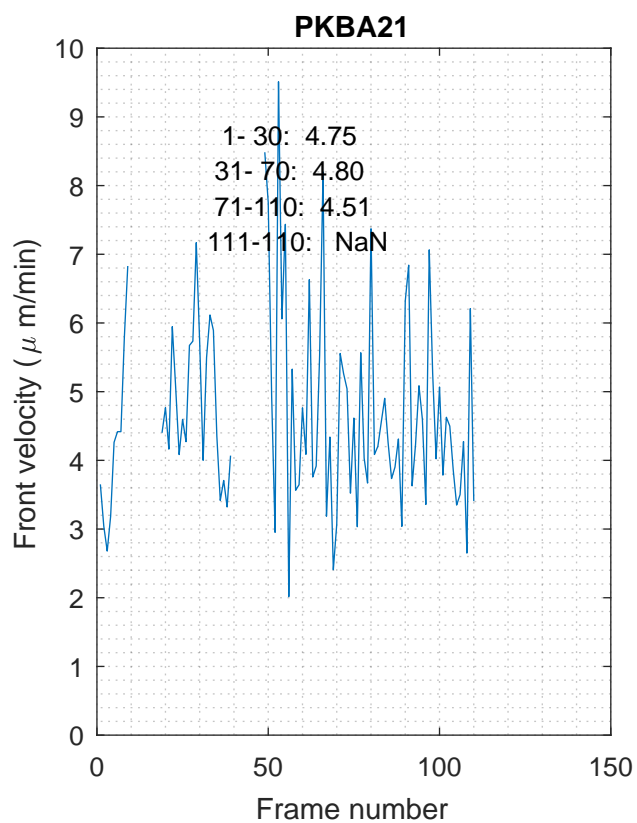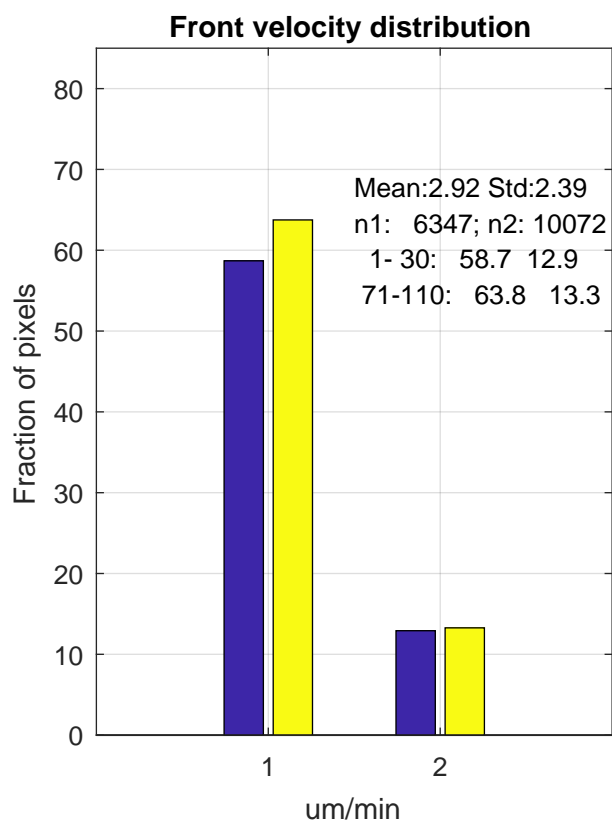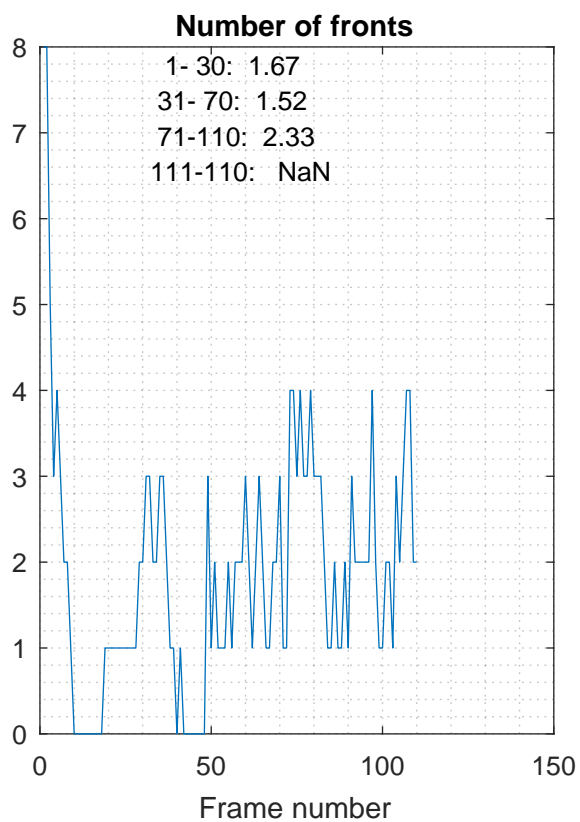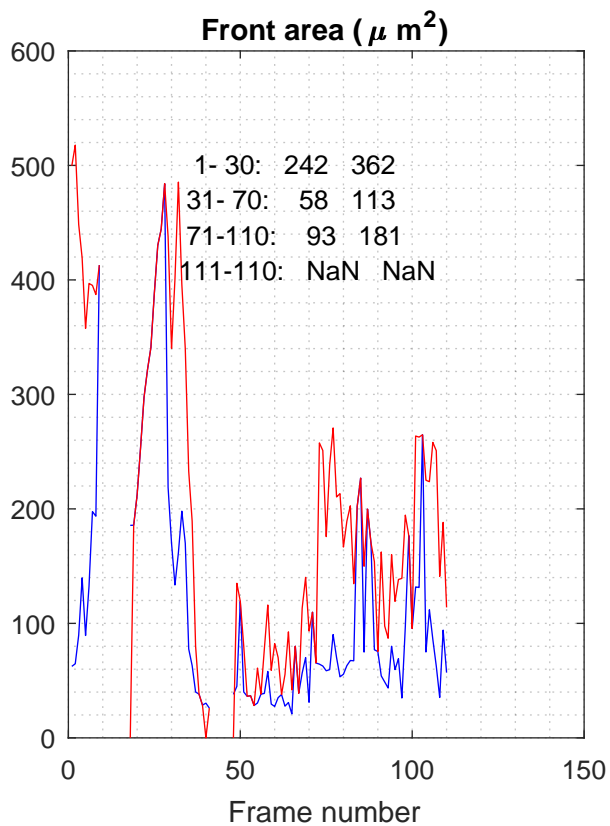

Supplement: Supplementary file 23 — Source Data for Figure 5 [file MSB-15-e8585-s021.zip › Source_data_for_Figure_5/Fig_5C/PKBA21.pdf]

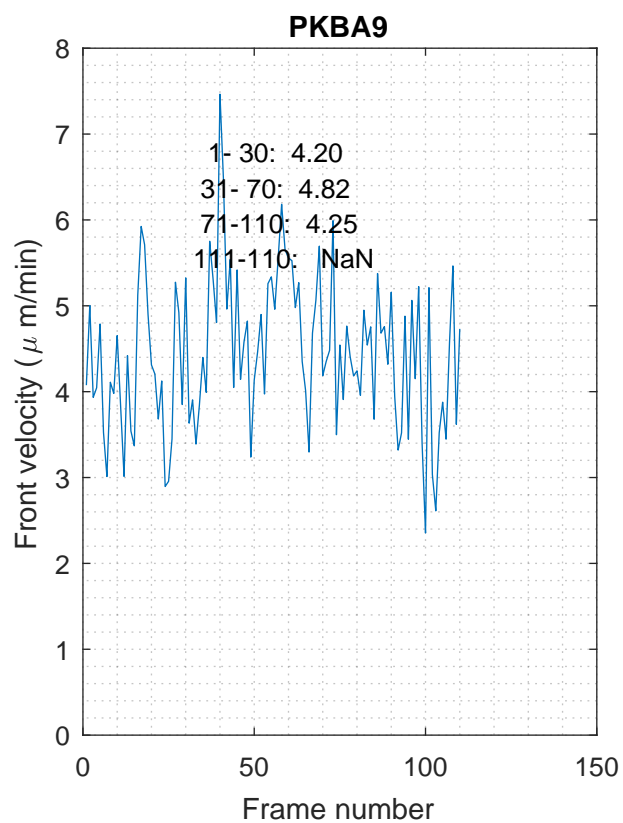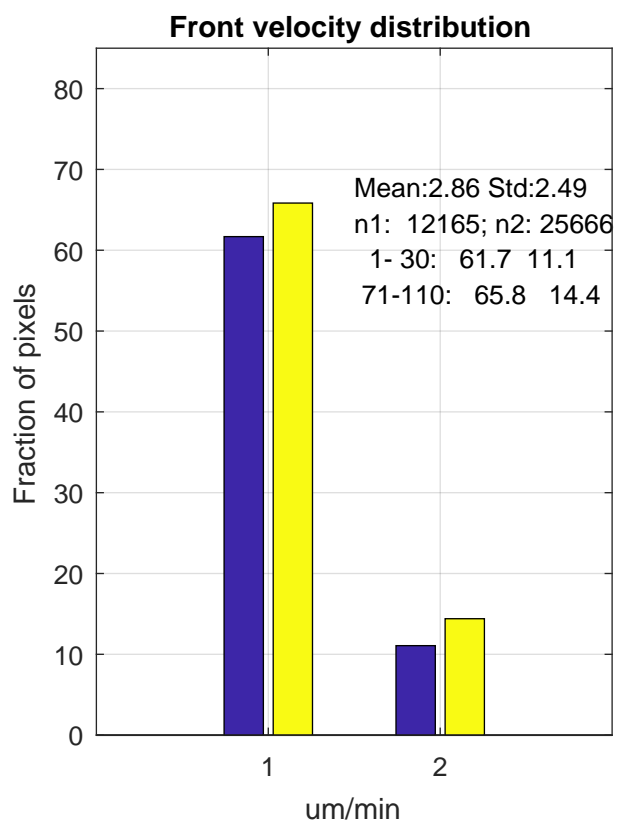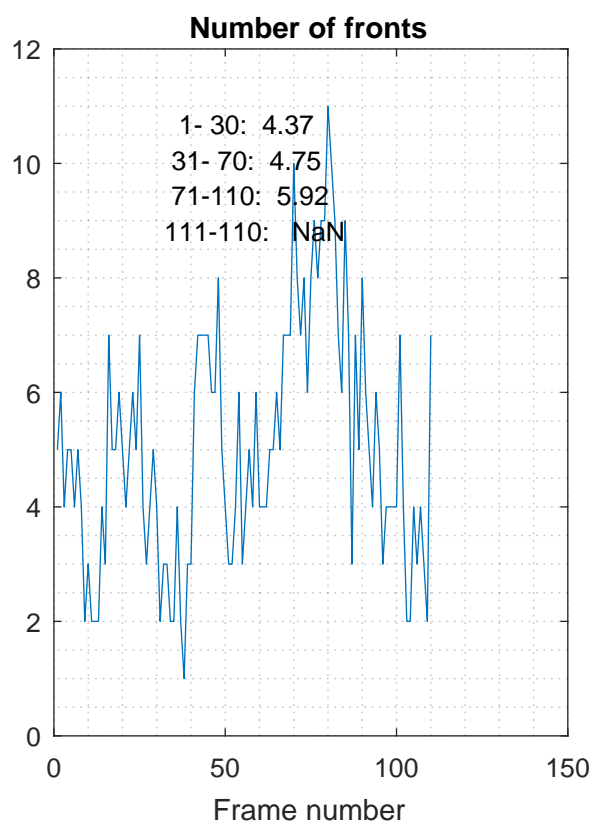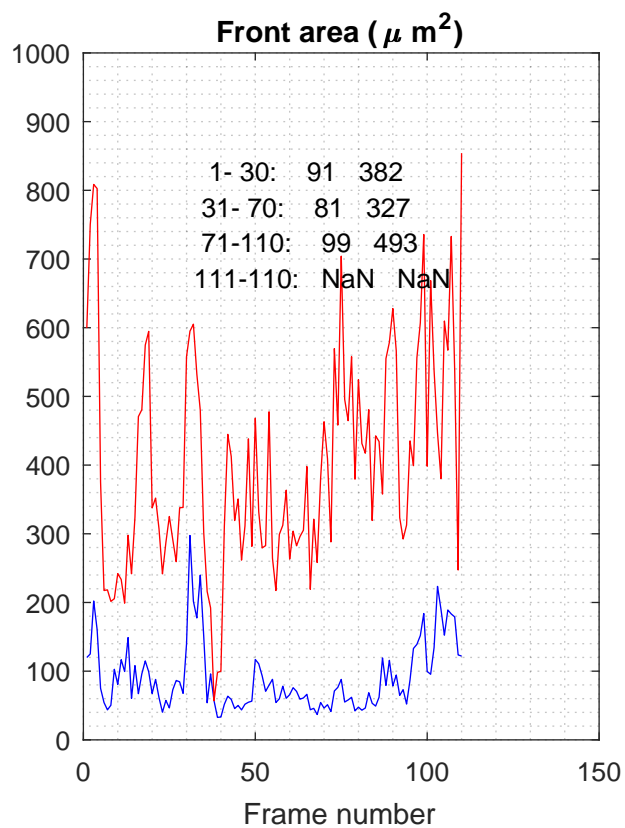

Supplement: Supplementary file 23 — Source Data for Figure 5 [file MSB-15-e8585-s021.zip › Source_data_for_Figure_5/Fig_5C/PKBA9.pdf]

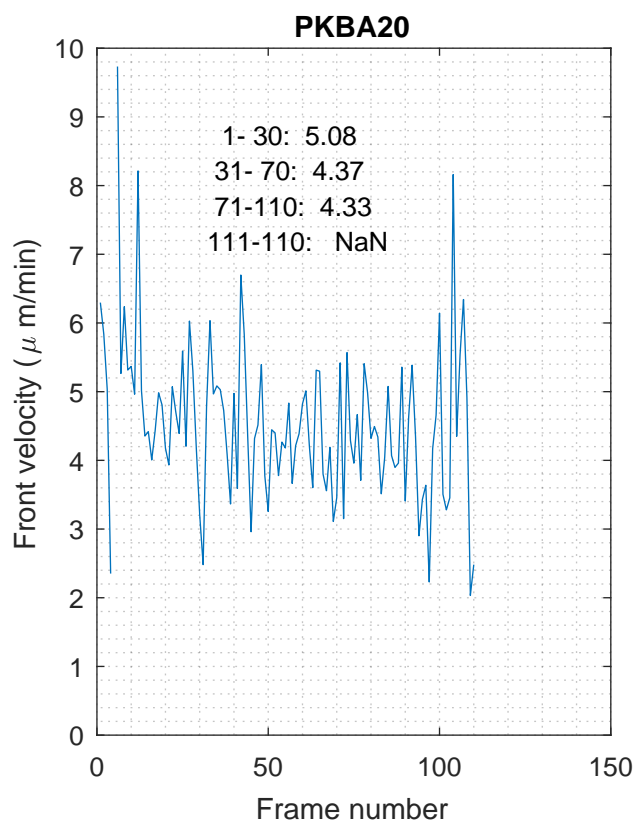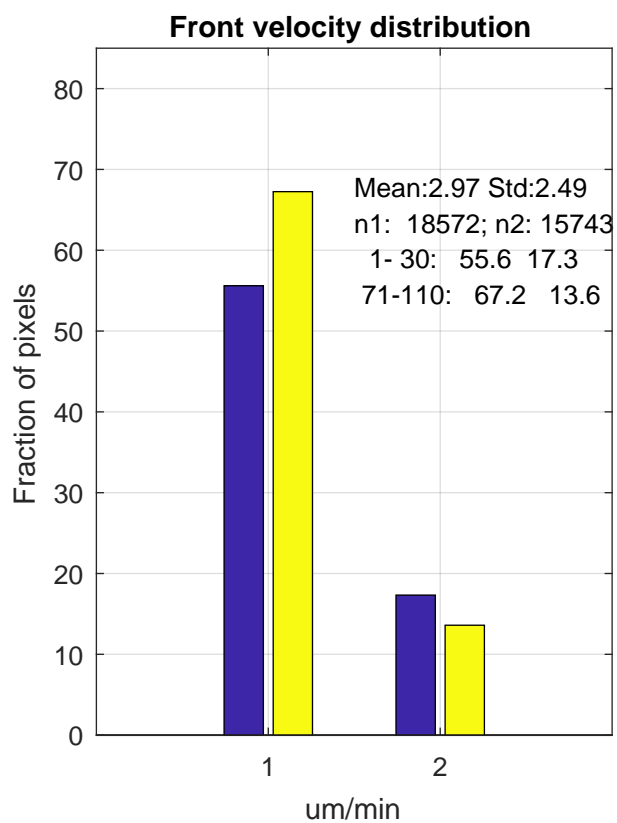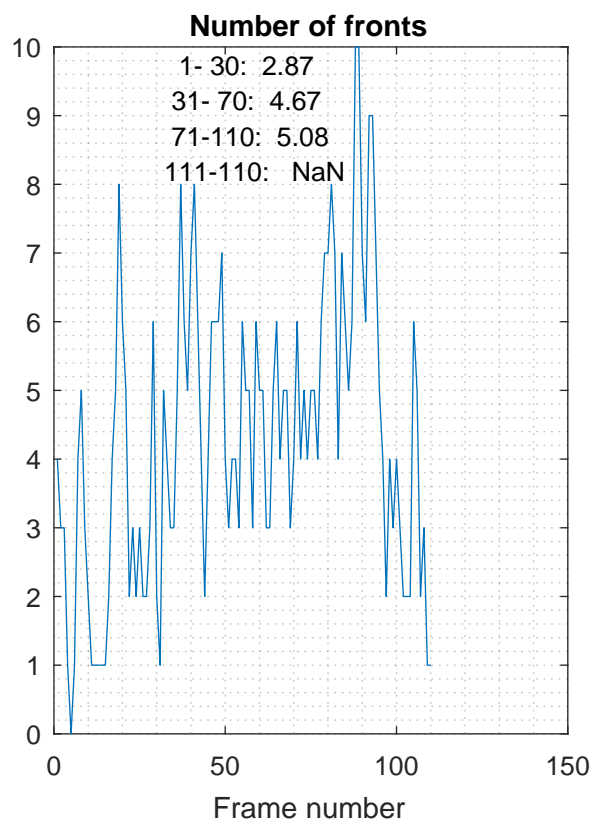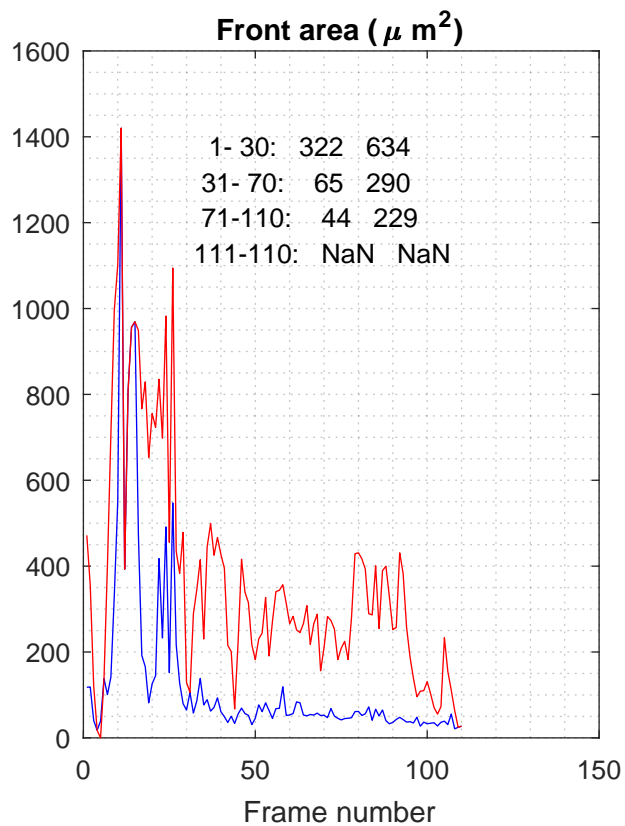

Supplement: Supplementary file 23 — Source Data for Figure 5 [file MSB-15-e8585-s021.zip › Source_data_for_Figure_5/Fig_5C/PKBA20.pdf]

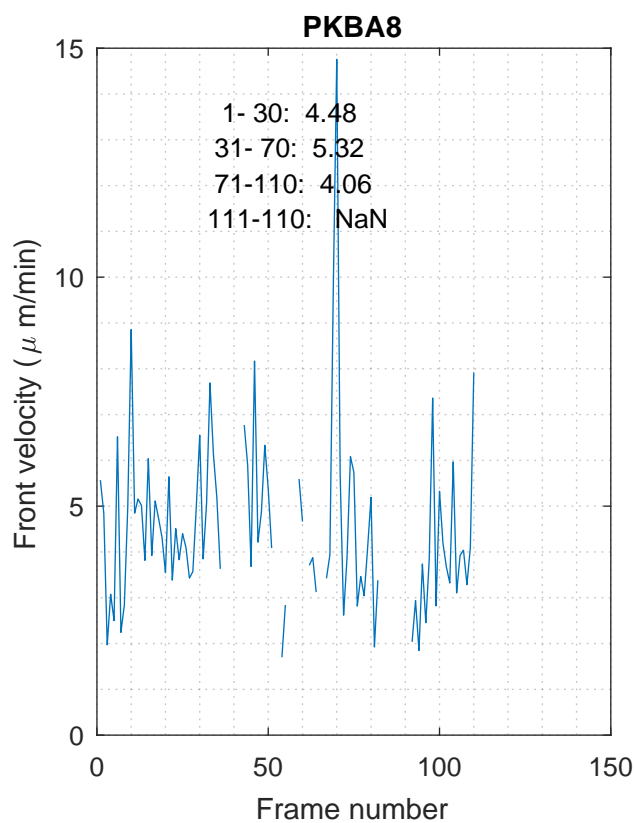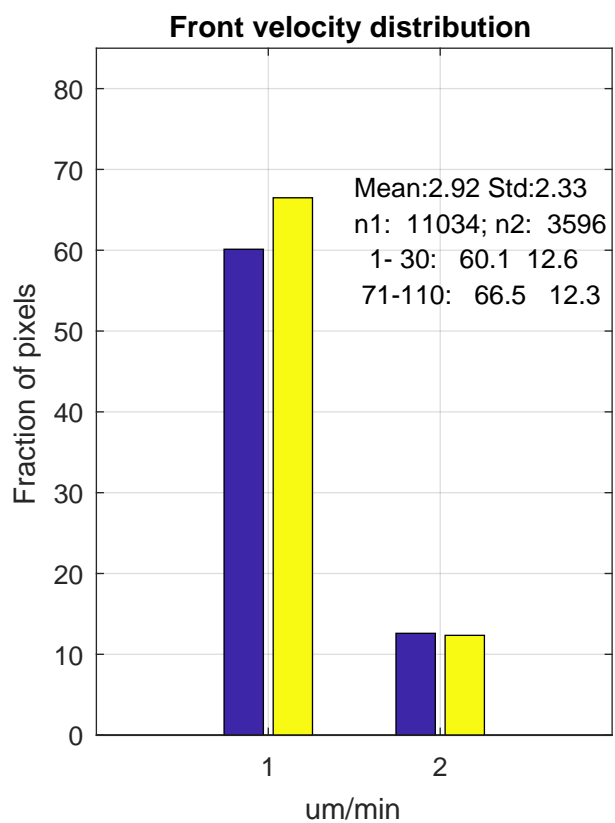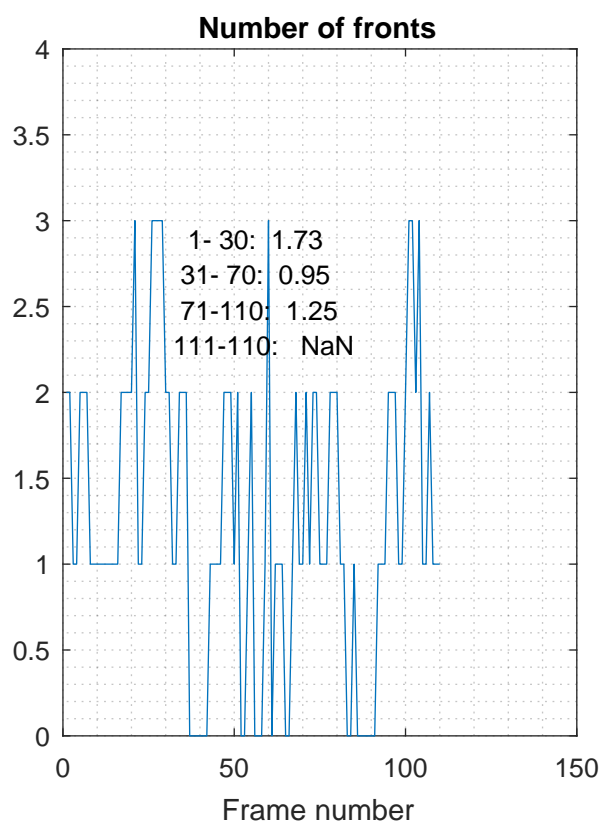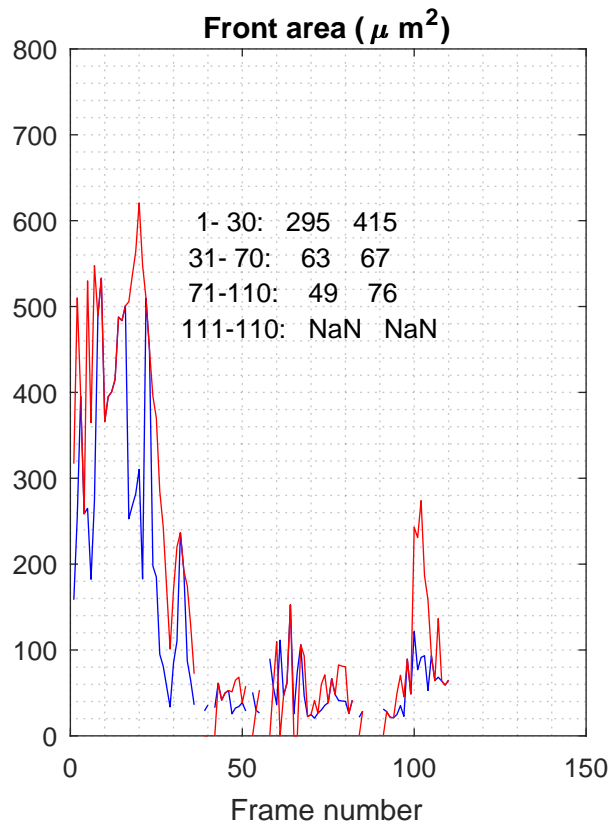

Supplement: Supplementary file 23 — Source Data for Figure 5 [file MSB-15-e8585-s021.zip › Source_data_for_Figure_5/Fig_5C/PKBA8.pdf]

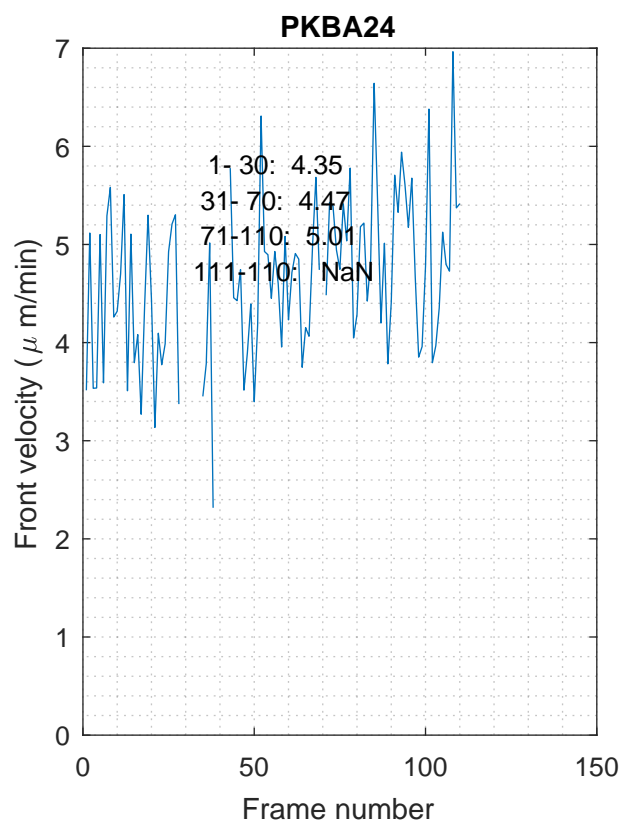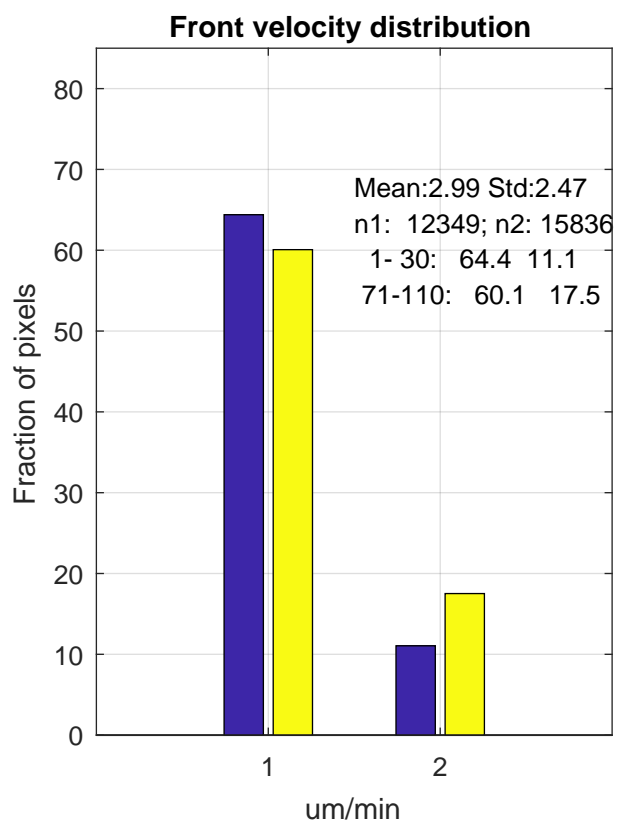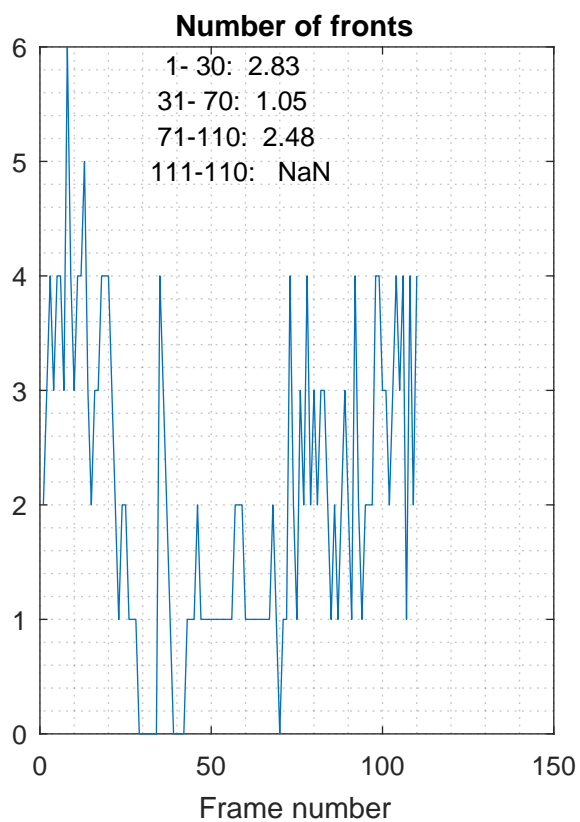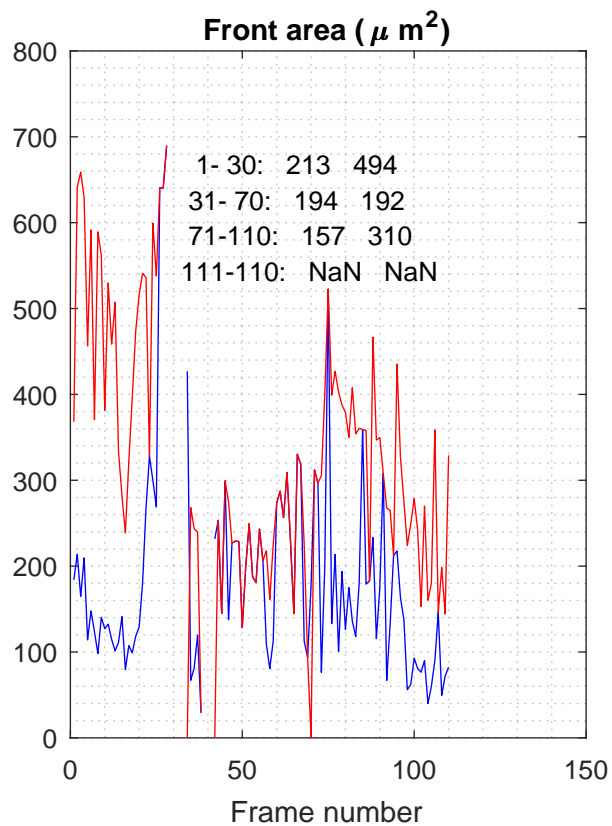

Supplement: Supplementary file 23 — Source Data for Figure 5 [file MSB-15-e8585-s021.zip › Source_data_for_Figure_5/Fig_5C/PKBA24.pdf]

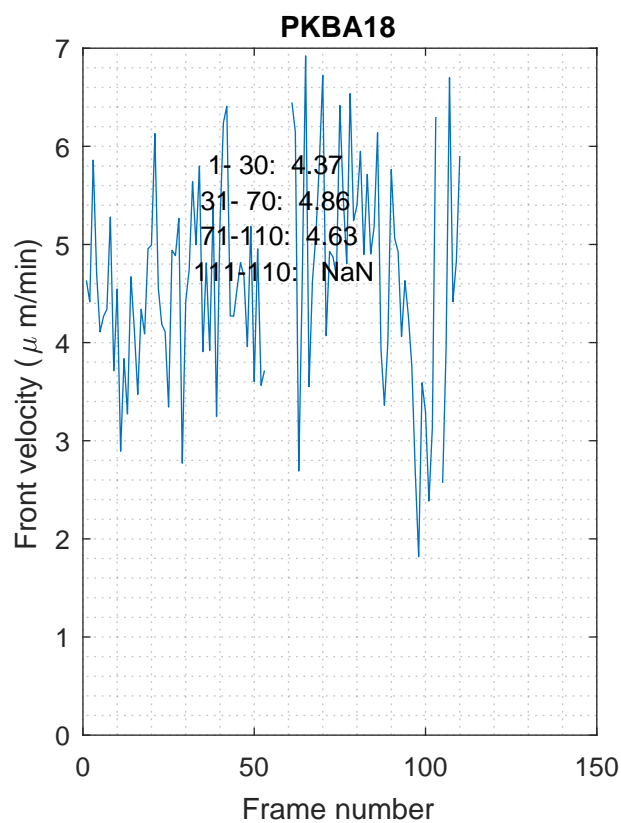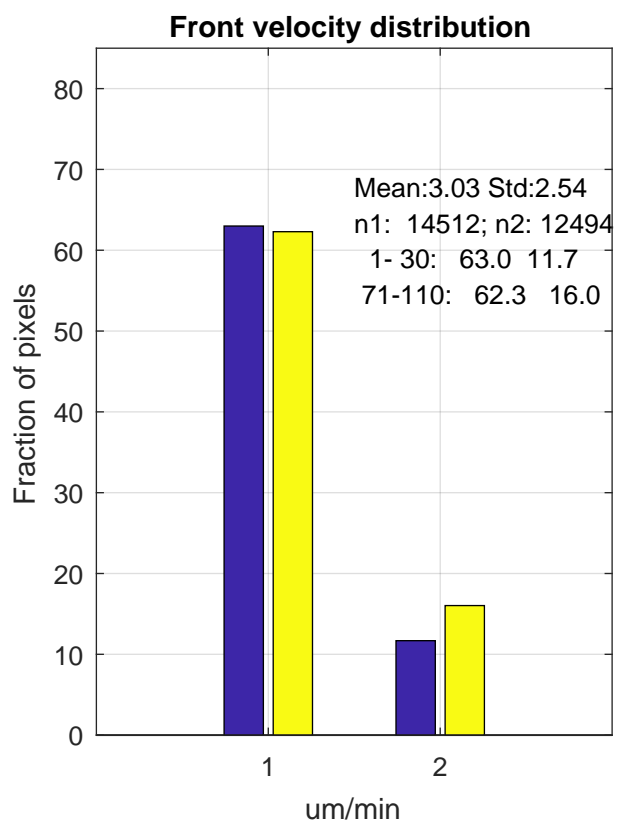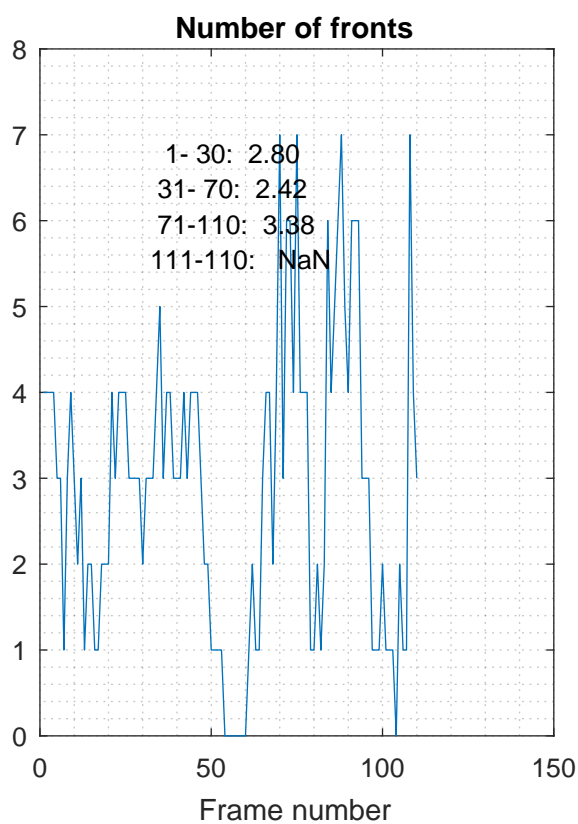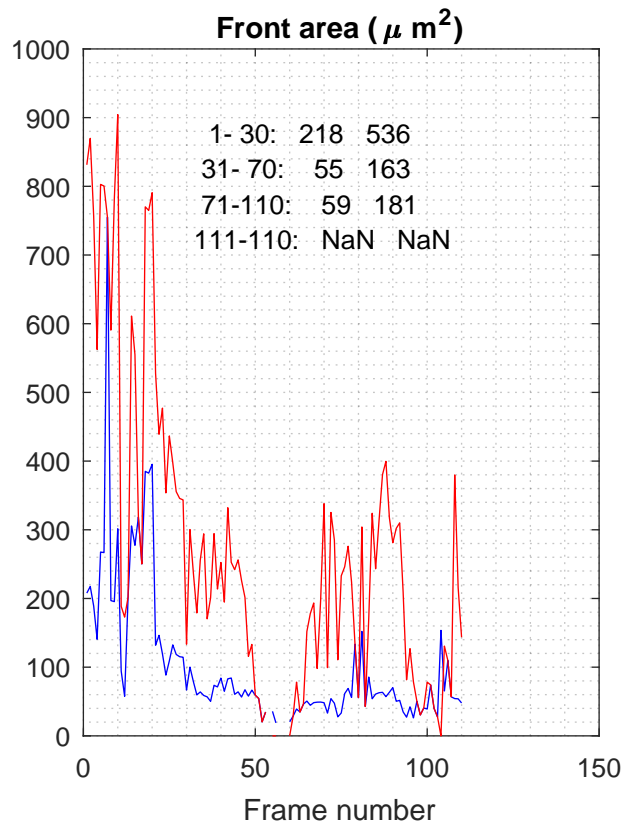

Supplement: Supplementary file 23 — Source Data for Figure 5 [file MSB-15-e8585-s021.zip › Source_data_for_Figure_5/Fig_5C/PKBA18.pdf]

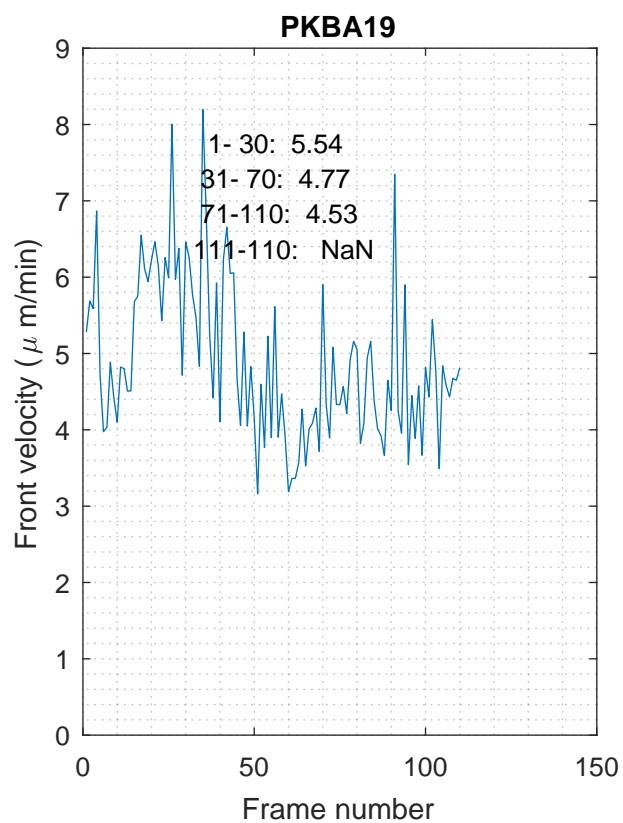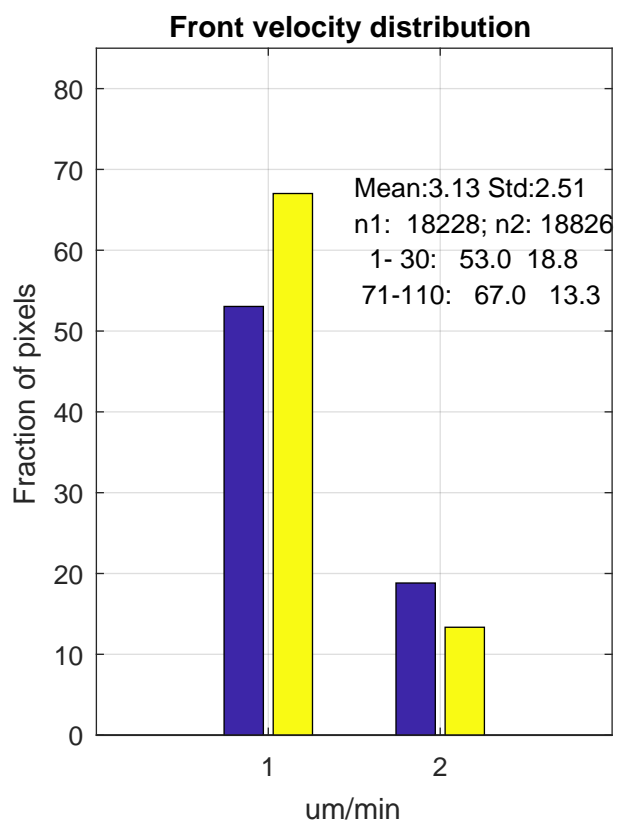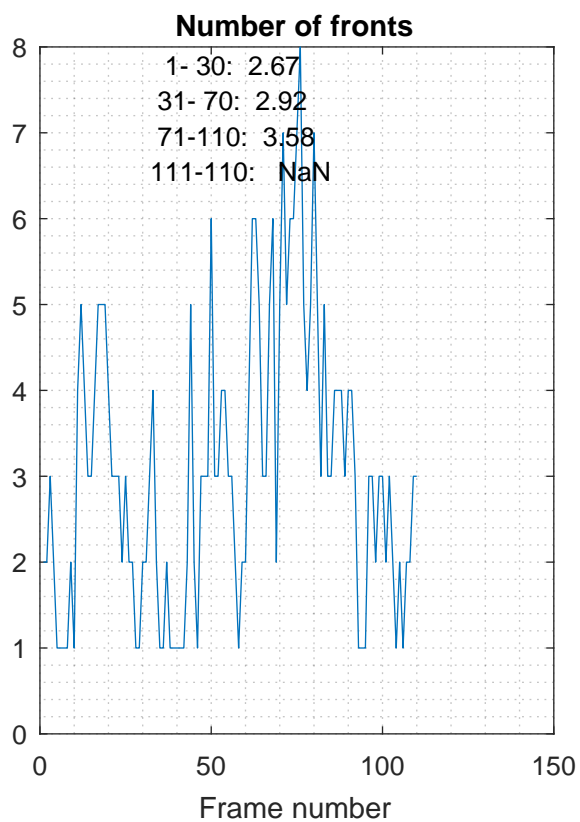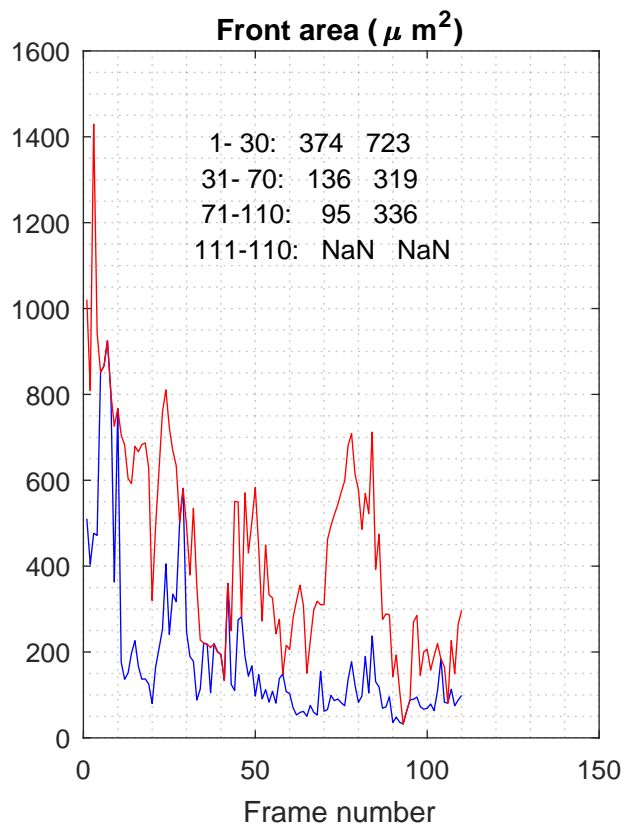

Supplement: Supplementary file 23 — Source Data for Figure 5 [file MSB-15-e8585-s021.zip › Source_data_for_Figure_5/Fig_5C/PKBA19.pdf]
